# 1,3,4-Oxadiazole-naphthalene hybrids as potential VEGFR-2 inhibitors: design, synthesis, antiproliferative activity, apoptotic effect, and *in silico* studies

**DOI:** 10.1080/14756366.2021.2015342

**Published:** 2021-12-20

**Authors:** Mohamed Hagras, Marwa A. Saleh, Rogy R. Ezz Eldin, Abdelrahman A. Abuelkhir, Emad Gamil Khidr, Ahmed A. El-Husseiny, Hesham A. El-Mahdy, Eslam B. Elkaeed, Ibrahim H. Eissa

**Affiliations:** a Pharmaceutical Organic Chemistry, Faculty of Pharmacy (Boys), Al-Azhar University, Cairo, Egypt; b Pharmaceutical Organic Chemistry, Faculty of Pharmacy (Girls), Al-Azhar University, Cairo, Egypt; c Department of Pharmaceutical Organic Chemistry, Faculty of Pharmacy, Port Said University, Port Said, Egypt; d Biochemistry and Molecular Biology Department, Faculty of Pharmacy (Boys), Al-Azhar University, Cairo, Egypt; e Department of Pharmaceutical Sciences, College of Pharmacy, AlMaarefa University, Riyadh, Saudi Arabia; f Pharmaceutical Medicinal Chemistry & Drug Design Department, Faculty of Pharmacy (Boys), Al-Azhar University, Cairo, Egypt

**Keywords:** Anticancer, apoptosis, docking, 1,3,4-oxadiazole, VEGFR-2 inhibitors

## Abstract

In the current work, some 1,3,4-oxadiazole-naphthalene hybrids were designed and synthesised as VEGFR-2 inhibitors. The synthesised compounds were evaluated *in vitro* for their antiproliferative activity against two human cancer cell lines namely, HepG-2 and MCF-7. Compounds that exhibited promising cytotoxicity (**5**, **8**, **15**, **16**, **17**, and **18**) were further evaluated for their VEGFR-2 inhibitory activities. Compound **5** showed good antiproliferative activity against both cell lines and inhibitory effect on VEGFR-2. Besides, it induced apoptosis by 22.86% compared to 0.51% in the control (HepG2) cells. This apoptotic effect was supported by a 5.61-fold increase in the level of caspase-3 compared to the control cells. Moreover, it arrested the HepG2 cell growth mostly at the Pre-G1 phase. Several *in silico* studies were performed including docking, ADMET, and toxicity studies to predict binding mode against VEGFR-2 and to anticipate pharmacokinetic, drug-likeness, and toxicity of the synthesised compounds.

## 1. Introduction

Although there are many advances in drug discovery for cancer control and treatment, still cancer is one of the most serious diseases responsible for a huge number of deaths^1^. According to global statistics, cancer is a leading cause of death worldwide, accounting for nearly 10 million deaths in 2020^2^. The cancer problem is localised not only in its widespread and metastasis but also in the lack of selectivity of anticancer drugs which leads to many side effects^3^. Moreover, the problem increases due to the continuous mutations in many targets of anticancer drugs which produce some sort of resistance^4^. Recently, medicinal chemists tried to develop new anticancer agents with high selectivity and can overcome the generated drug resistance^5–7^.

Vascular endothelial growth factor receptors (VEGFRs) are a group of tyrosine kinases that caught the attention of scientists for the discovery of new anticancer agents^8^^,^^9^. VEGFRs group is subdivided into three categories. (i) VEGFR-1 is responsible for the control of angiogenesis in embryos^10^. (ii) VEGFR-2 is the key element in tumour growth due to its crucial role in the formation of new vasculatures and angiogenesis^11^. (iii) VEGFR-3 controls the process of lymphangiogenesis^12^. VEGFR-2 took more interest due to its important role in tumour growth^13^.

In embryonic vasculogenesis, VEGFR1 plays a critical function. VEGFR2 is a protein that controls embryonic and tumour angiogenesis. VEGFR2 has mostly overexpressed in tumour vasculature endothelial cells, with reduced expression in normal endothelial cells^14^. Overexpressed VEGFR-2 is found in a variety of malignancies, including hepatocellular carcinoma and breast cancer^15–17^. Blocking VEGFR2 is a viable method for the identification of novel therapies for angiogenesis-dependent cancers^18^. VEGFR2 is now the most significant target of antiangiogenic therapy. During development, VEGFR3 is found in all endothelium, but it is only found in the lymphatic endothelium in adults^19^. It is up-regulated in the microvasculature of tumours and wounds^20^.

Till now, there are many drugs approved by the FDA targeting VEGFR-2 for the treatment of cancer^13^. Sorafenib **1** was approved for the treatment of thyroid cancer, metastatic renal cell cancer, and hepatocellular carcinoma. It is associated with many adverse effects as diarrhoea, renal dysfunction, and cardiovascular problems^21^. Sunitinib **II** is an oral VEGFR-2 inhibitor that exhibits potent antiangiogenic and antitumor activities. It exhibits a high bioavailability and potency against VEGFR in the nanomolar range. Small-cell lung cancer, GI stromal tumours, breast cancer, acute myelogenous leukaemia, multiple endocrine neoplasia types 2A and 2B, and familial medullary thyroid carcinoma were all treated with sunitinib^22^. Vorolanib III is a multi-target tyrosine kinase inhibitor that has successfully completed phase I studies. At a dose of 200 mg (once daily), it had an acceptable safety profile and a favourable therapeutic benefit for patients with advanced solid tumours^23^. Tivozanib IV is a powerful and highly selective orally accessible tyrosine kinase inhibitor with a long half-life (4 days) that targets VEGFR-1, VEGFR-2, and VEGF-3 at very low dosages^24^.

**Figure 1.**
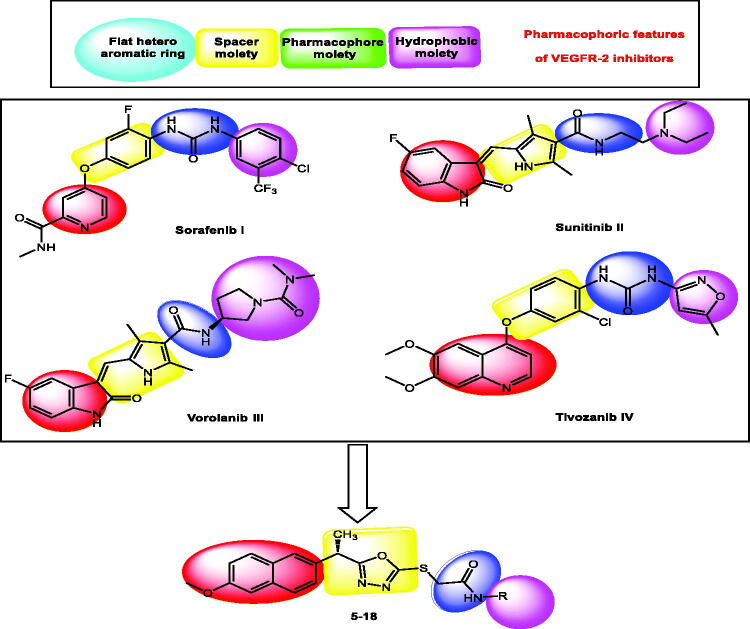
Some reported VEGFR-2 inhibitors (I, II, III, and IV) and the target compounds (**5–18**) having the same pharmacophoric features.

Four key pharmacophoric features of VEGFR-2 inhibitors have been identified^25–30^. Each feature has its own binding area in VEGFR-2's active site. The hinge region is occupied by a flat heteroaromatic moiety that forms one hydrogen bond with Cys917^26^. The second distinguishing feature is a linker moiety that sits between the hinge region and the DFG domain^31^. The pharmacophore moiety, which occupies the DFG domain and forms two crucial hydrogen bonding interactions with Glu883 and Asp1044, is the third feature. At least one H-bond acceptor (HBA) and one H-bond donor (HBD) group (e.g. amide or urea) must be present in the pharmacophore moiety^32^. The fourth feature is a terminal hydrophobic moiety that occupies the allosteric pocket, resulting in numerous hydrophobic interactions^33^ (Figure 1).

As an extension of our efforts to reach potent anti-VEGFR-2 agents^29^^,^^34–39^, a new series of 1,3,4-oxadiazole-naphthalene hybrids was synthesised as a modified form of the reported VEGFR-2 inhibitors. The synthesised hybrids were designed to have the main features of VEGFR-2 inhibitors and evaluated to confirm their VEGFR-2 inhibitory activities.

### 1.1. Rationale of molecular design

The 1,3,4-oxadiazole moiety has various biological activities. The wide and potent activity of this moiety made it an important pharmacological scaffold for drug design especially in the field of cancer disease^5^^,^^40^^,^^41^. Additionally, naphthalene is a main building block in many anticancer agents^42^^,^^43^. The molecular hybridisation approach is one of the most interesting and efficient method for the design and discovery of new bioactive agents^44^^,^^45^. Depending on this approach and in continuation of our activities in the discovery of VEGFR-2 inhibitors, we synthesised a new series 1,3,4-oxadiazole-naphthalene hybrids.

The 2-methoxynaphthalene moiety was used to occupy the hinge region of the VEGFR-2 binding site to validate the main pharmacophoric features of VEGFR-2 inhibitors. The naphthalene moiety's bicyclic structure is well-suited to the large size space of the hinge region^46^. Furthermore, the methoxy group serves as a hydrogen-bond acceptor, facilitating hydrogen bonding interactions in the hinge region. As a linker group, the 2-ethyl-1,3,4-oxadiazole moiety was used. In the linker region, the 1,3,4-oxadiazole moiety contains two nitrogen atoms that can form extra hydrogen bonds. As a pharmacophore moiety, we used an amide group to occupy the DFG region. Finally, different aromatic derivatives can occupy the allosteric hydrophobic region to investigate structure-activity relationships (Figure 1).

## 2. Results and discussion

### 2.1. Chemistry

**Scheme 1.**
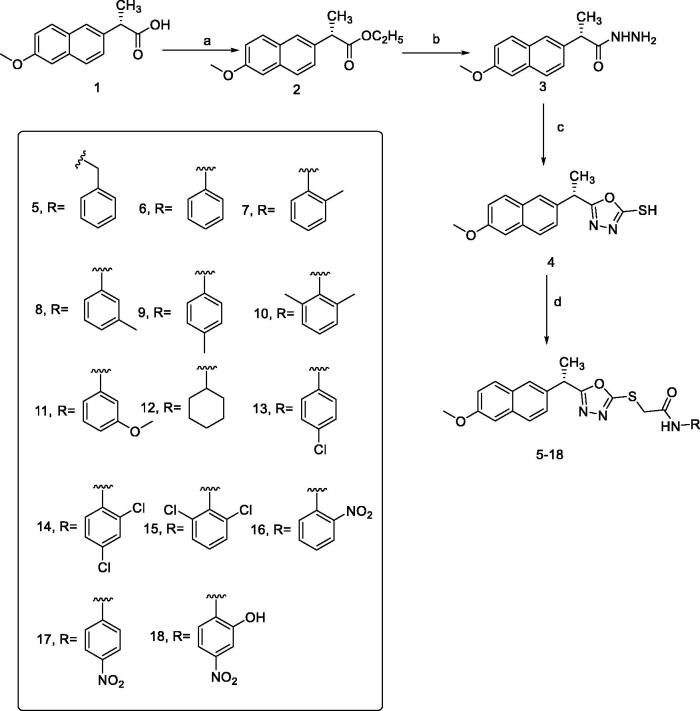
Synthesis of the target compounds **5–18**. Reagents and conditions: (a) C_2_H_5_OH/H_2_SO_4_; (b) NH_2_NH_2_·H_2_O/C_2_H_5_OH; (c) CS2\KOH, EtOH; (d) RX/EtOH, NaOAc.

Different synthetic pathways were described in Scheme 1 for the preparation of final designed compounds. Firstly, the commercially available S(+)naproxen **1** ((S)-2–(6-methoxynaphthalen-2-yl)propanoic acid) was esterified using absolute ethanol and conc. H_2_SO_4_ to produce the ester form **2**. Then, the ester derivative **2** was heated in ethanol and hydrazine hydrate to produce the corresponding hydrazide derivative **3**.

The synthesis of the key starting material 1,3,4-oxadiazolyl scaffold **4** was achieved by the reaction of acid hydrazide **3** with carbon disulphide in an alcoholic potassium hydroxide solution. Different chloroacetinilides were obtained by the treatment of aromatic amines with chloroacetyl chloride^47^. The potassium salt of **4** was allowed to react with the substituted chloroacetinilides. Unfortunately, the required products couldn't be separated from the reaction mixture with the desirable purity. The presence of more than one product for each reaction was attributed to the high reactivity and/or basicity of the potassium salt. Alternatively, a less basic condition was adopted and the mercapto-containing structure **4** was directly allowed to react with chloroacetinilides in the presence of sodium acetate. This alternative route successfully afforded the final products in satisfying yields and reasonable purities.

The spectral and elemental analytical data of this group of novel compounds confirmed their structures (see experimental section). In all cases, the characteristic thiol stretching band at 2624 cm^−1^ vanished in all IR spectra and was replaced by a higher frequency one distinctive band of the primary amide NH moiety. Furthermore, in all IR spectra, typical carbonyl stretching bands between 1655 and 1675 cm^−1^ were observed. These findings support the idea that the acetanilide moiety is linked to the oxadiazole nucleus *via* S-linkage. The later NH group revealed a broad singlet, corresponding to one proton around 10.4 ppm. As an example, the ^1^H NMR spectrum of compound **7** revealed a broad singlet signal, equivalent to one proton, at 10.36 ppm due to NH, and a multiplet signal, equivalent to ten protons, at 7.74–7.15 ppm due to aromatic protons. Finally, the ethyl linker between the naphthalene and oxadiazole rings produces a multiplet for one proton due (CH) and a doublet for three protons due to (CH_3_) at 4.53 and 1.78 ppm, respectively. Aliphatic (S-CH2) protons were detected as a singlet signal at 4.01 ppm, a singlet signal equivalent to three protons at 3.81 ppm due to OCH3, and a singlet signal of three protons at 1.95 ppm due to the benzylic methyl moiety. The ^13^C NMR spectrum of compound **7** revealed 24 signals.

The ^1^H NMR spectra of compounds **12**, showed, in addition to naphthalene aromatic protons, broad singlet due to NH group at 8.12 ppm, the ethyl group appeared as multiplet and doublet signals at 4.57 and 1.66 ppm, a singlet for two protons at 4.94 ppm due S-CH_2_ group, another singlet for three protons at 3.83 ppm due to O-CH_3_. While the cyclohexyl side chain is represented by a multiplet signal between 1.66 and 1.04 ppm.

In general, mass spectra of compounds-containing halogen atoms showed the typical isotopic distribution patterns. The parent peak intensity of compounds **14** and **15** makes it easy to recognise the expected isotopic pattern of the two chlorine atoms; however, an anticipated isotopic ratio was observed with some fragments-containing chlorine; i.e. the anilinium fragment showed M^+^, (M + 2)^+^, and (M + 4)^+^ isotopic peaks as detailed in the experimental section.

### 2.2. Biological testing

#### 2.2.1. In vitro cytotoxic activities

MTT assay was used to assess the cytotoxic activities of the synthesised compounds against MCF-7 (human breast cancer cell line) and HepG2 (human hepatocellular carcinoma cell line). And Sorafenib was used as a control drug (Table 1). When compared to sorafenib (IC_50_ = 10.8 and 10.2 µM against MCF-7 and HepG2 cells, respectively), the target compounds **5**, **8**, **15**, **16**, **17**, and **18** showed promising cytotoxicity against the two cell lines with IC_50_ values ranging from 8.4 to 10.4 µM. Compound **5** demonstrated superior activity against MCF-7 and HepG2 cells, with IC_50_ values of 9.7 and 8.8 M, respectively. With IC_50_ values of 8.4 M, compound 15 demonstrated promising activity against HepG2 cells. Compounds **5**, **8**, **15**, **16**, **17**, and **18** demonstrated excellent activity against HepG2 cells, with IC_50_ values of 8.8, 9.5, 8.4, 8.7, 9.2, and 8.7 µM, respectively. Compounds **6**, **7**, **10**, **11**, **12**, **13**, and **14** also demonstrated moderate activity against the tested cells, with IC_50_ values ranging from 10.4 M to 25.5 µM. Compound **9** on the other hand demonstrated weak cytotoxic activity against the two cell lines.

**Table 1.** The assessed compounds' *in vitro* cytotoxic activities (IC_50_) against MCF-7 and HepG2 cell lines.

Comp.	Structure	IC_50_ (µM)^a^		
		MCF-7	HepG2	
Sorafenib	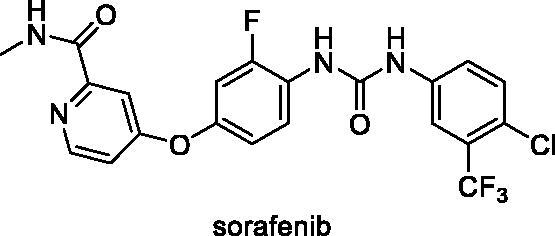	10.8 ± 1.01	10.2 ± 0.97	
5	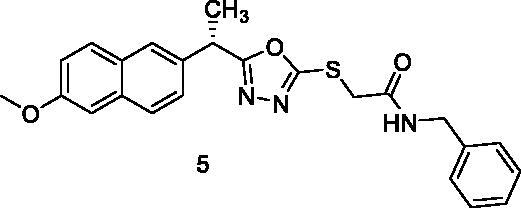	9.7 ± 0.75	8.8 ± 0.69	
6	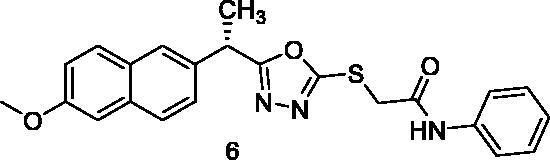	13.2 ± 1.12	12.3 ± 0.87	
7	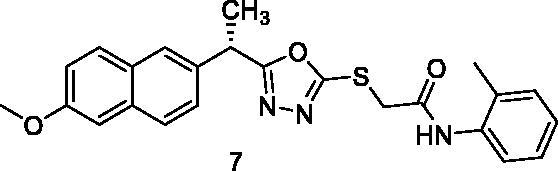	13.6 ± 1.09	12.5 ± 1.09	
8	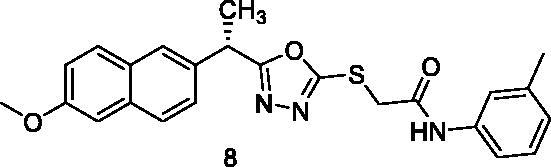	10.4 ± 0.92	9.5 ± 0.87	
9	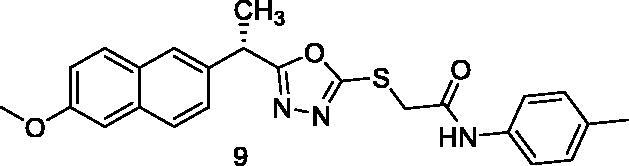	83.5 ± 7.79	83.7 ± 6.52	
10	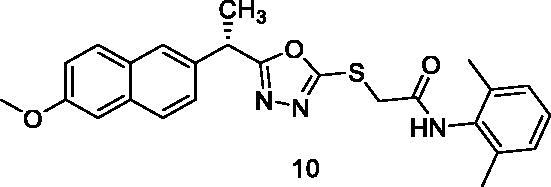	12.5 ± 0.74	11.8 ± 1.04	
11	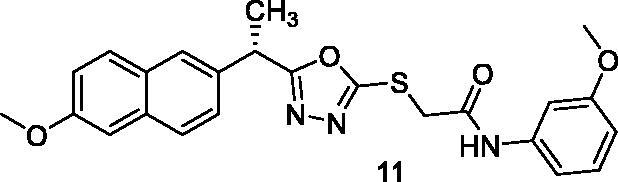	11.4 ± 0.81	10.7 ± 0.58	
12	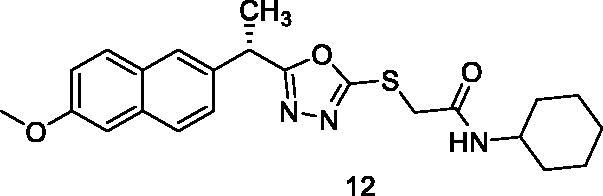	25.5 ± 2.02	24.6 ± 2.81	
13	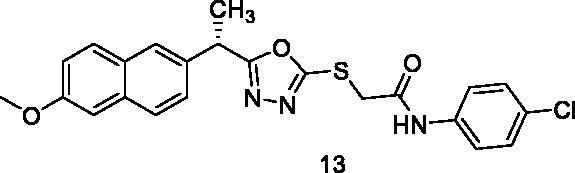	11.4 ± 0.99	10.5 ± 0.93	
14	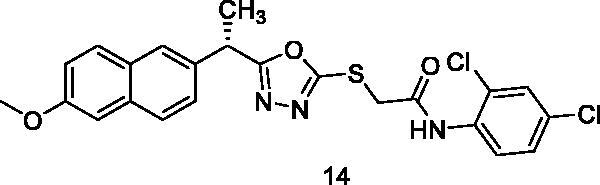	11.2 ± 0.69	10.4 ± 0.81	
15	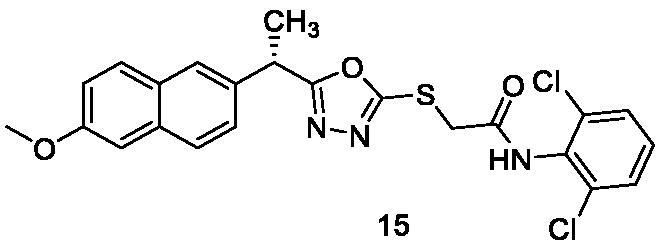	9.8 ± 0.46	8.4 ± 0.72	
16	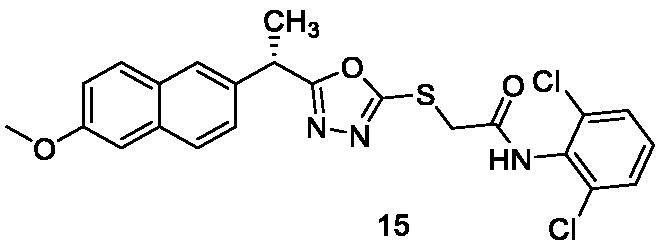	9.8 ± 0.80	8.7 ± 0.72	
17	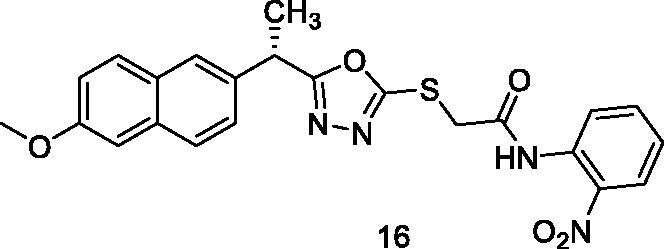	10.4 ± 0.82	9.2 ± 0.76	
18	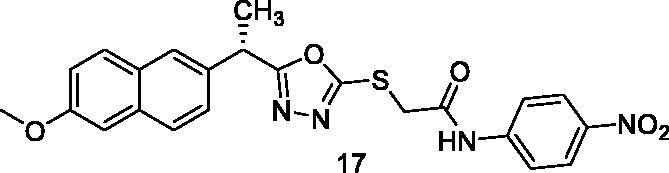	9.4 ± 0.64	8.7 ± 0.75	

^a^The mean ± SEM of at least three different experiments is used to calculate all IC_50_ values.

#### 2.2.2. VEGFR-2 inhibitory assay

**Figure 2.**
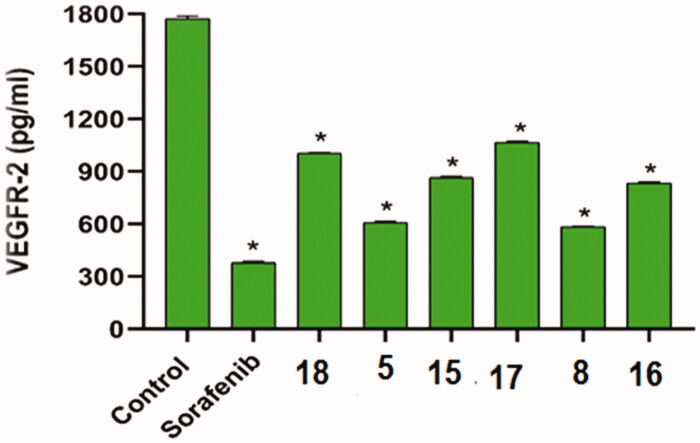
The inhibitory effects of the compounds tested on VEGFR-2 in HepG2 cells compared to sorafenib. The data are presented as the mean ± SEM of three different experiments. *Significant from control group at *p* < 0.001 using unpaired *t*-test.

The inhibitory effect of the most cytotoxic compounds **5**, **8**, **15**, **16**, **17**, and **18** on VEGFR-2 was studied using sorafenib as a control drug. VEGFR-2 concentrations after inhibition by the aforementioned compounds against HepG2 cells were summarised in Table 2 and Figure 2.

**Table 2.** The inhibitory effects of the assessed compounds on VEGFR-2 in HepG2 cells compared to sorafenib.

Comp.	VEGFR-2 (pg/ml)^a^
	HepG2
Control	1773 ± 15.01
Sorafenib	378.7 ± 7.22
5	610.4 ± 3.46
8	583.7 ± 1.93
15	864.5 ± 5.92
16	834.9 ± 5.17
17	1067 ± 4.91
18	1004 ± 5.49

^a^The data is presented as the mean ± SEM of three different experiments.

Compounds **5** and **8** inhibited VEGFR-2 well (concentrations of 610.4 and 583.7 pg/ml, respectively) when compared to sorafenib (378.7 pg/ml). Compounds **15** and **16** also had moderate effects (864.5 and 834.9 pg/ml, respectively). Compounds **17** and **18**, on the other hand, had low effects (1067 and 1004 pg/ml, respectively).

#### 2.2.3. In vitro cytotoxicity against normal cell

The cytotoxic effects of the most active compounds **5**, **8**, **15**, **16**, **17**, and **18** against normal adult liver epithelial cells (Transformed Human Liver Epithelial-2, THLE-2 cells) were assessed *in vitro* (Table 3). The findings revealed that these compounds have low cytotoxicity against the normal THLE-2 cells with IC_50_ values of 33.7, 16.7, 34.9, 29.7, 22.8, and 28.6 µM, respectively. While IC_50_ value of sorafenib as a reference drug was 27.8 µM. The results showed that these synthesised compounds have low cytotoxicity against the normal cells in comparison to their cytotoxicity against cancer cell lines.

**Table 3.** *In vitro* cytotoxicity of the assessed compounds and sorafenib against normal adult liver epithelial cells (THLE-2 cells).

Comp.	THLE-2 IC_50_ (µM)^a^
Sorafenib	27.8 ± 1.74
**5**	33.7 ± 1.35
**8**	16.7 ± 0.73
**15**	34.9 ± 1.86
**16**	29.7 ± 2.23
**17**	22.8 ± 1.22
**18**	28.6 ± 2.1

^a^The mean ± SEM of at least three different experiments is used to calculate all IC_50_ values.

#### 2.2.4. Structure–activity relationship (SAR)

The results of different biological analyses (cytotoxic activity and VEGFR-2 inhibitory assay) gave a valuable SAR. Comparing the cytotoxic activity of compounds **5** (incorporating benzyl moiety as a hydrophobic tail) and **6** (incorporating phenyl moiety as a hydrophobic tail), indicated that benzyl moiety enhances the cytotoxic activity. Modification of compound **6** by insertion of the methyl group at ortho-position (compound **7**) gave a mild change in activity. While insertion of the methyl group at meta-position (compound **8**) afforded a significant increase in the cytotoxic activity. On the other hand, the methyl substitution of phenyl moiety at para-position (compound **9**) produced a dramatic decrease in the cytotoxic activity. Insertion of the methyl group at the two ortho-positions of phenyl ring (compound **10**) did not produce a significant change in the cytotoxic activity. Comparing the cytotoxicity of compound **8** (with 3-methylphenyl moiety) and compound **11** (with 3-methoxyphenyl moiety), indicated that methyl moiety is more advantageous than the methoxy group. Insertion of the electron-withdrawing group at para-position (compound **13**) gave a mild increase in the cytotoxic activity. Insertion of chloro atom at both 2 and 4 positions of phenyl ring (compound **14**) did not produce a significant change in the cytotoxic activity. While insertion of the chloro atom at both 2 and 6 positions of the phenyl ring (compound **15**) produced a significant change in the cytotoxic activity. Insertion of the nitro group at ortho-position of phenyl ring (compound **16**) or para-position (compound **17**) gave a dramatic increase in the biological activity. Modification of compound **17** by insertion of the hydroxyl group at position −2 of the phenyl ring to give compound **18** afforded a mild decrease in activity. Changing the phenyl ring of compound **6** by cyclohexyl moiety (compound **12**), produced a dramatic decrease in the cytotoxic activity. This indicated that aromatic hydrophobic moiety is more efficient than not aromatic one.

#### 2.2.5. Cell cycle analysis

**Figure 3.**
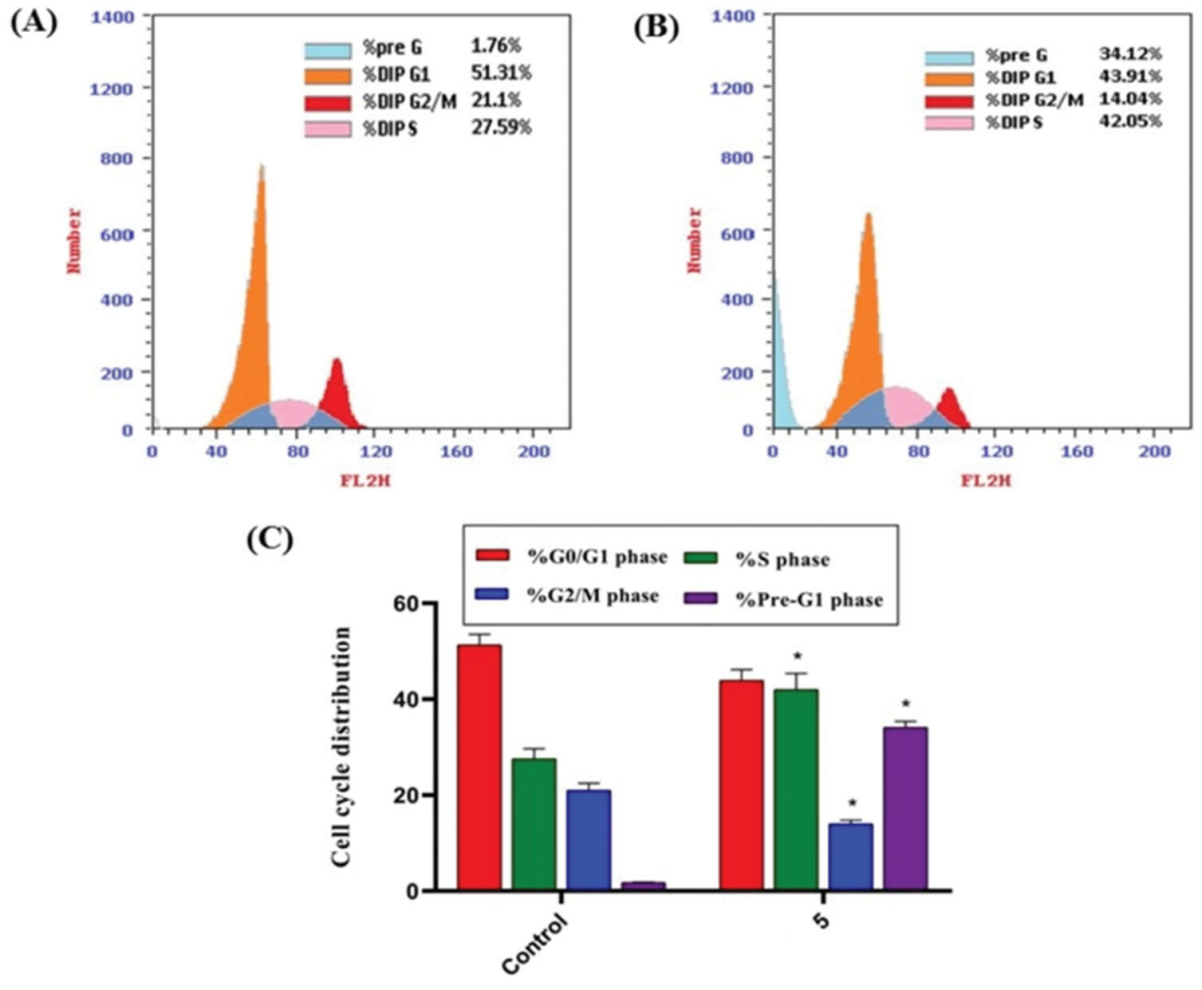
Flow cytometric analysis of cell cycle phases after compound **5** treatment of HepG2 cells. (A) The representative histogram depicts the cell cycle distribution of control (HepG2) cells; (B) the representative histogram depicts the cell cycle distribution of compound **5**-treated cells. (C) A column graph depicts the percentage of cells in each phase of the cell cycle in both control (HepG2) and compound **5** treated cells. The percentages are given as the mean SEM of three different experiments. Using unpaired *t*-tests, *p < 0.05 indicates statistically significant differences from the untreated control (HepG2) group.

Because compound **5** effectively inhibited the growth of HepG2 cells, it was assumed that this inhibitory effect was due to its ability to obstruct cell cycle progression. As a result, the cell cycle process was investigated after HepG2 cells were exposed to compound **5** at a concentration of 8.8 µM (IC_50_ value of compound **5**). As a control, HepG2 cells were not treated with compound **5**. According to flow cytometry data, the percentage of cells arrested in the S phase increased from 27.59% (in control cells) to 42.05% (in compound **5** treated cells). Furthermore, the percentage of HepG2 cells increased from 1.76 to 34.12% during the Pre-G1 phase. In contrast, the percentage of HepG2 cells decreased slightly during the G0/G1 phase, from 51.31 to 43.91%. These findings revealed that compound **5** primarily inhibited HepG2 cell growth during the Pre-G1 phase (Figure 3).

#### 2.2.6. Apoptosis analysis

**Figure 4.**
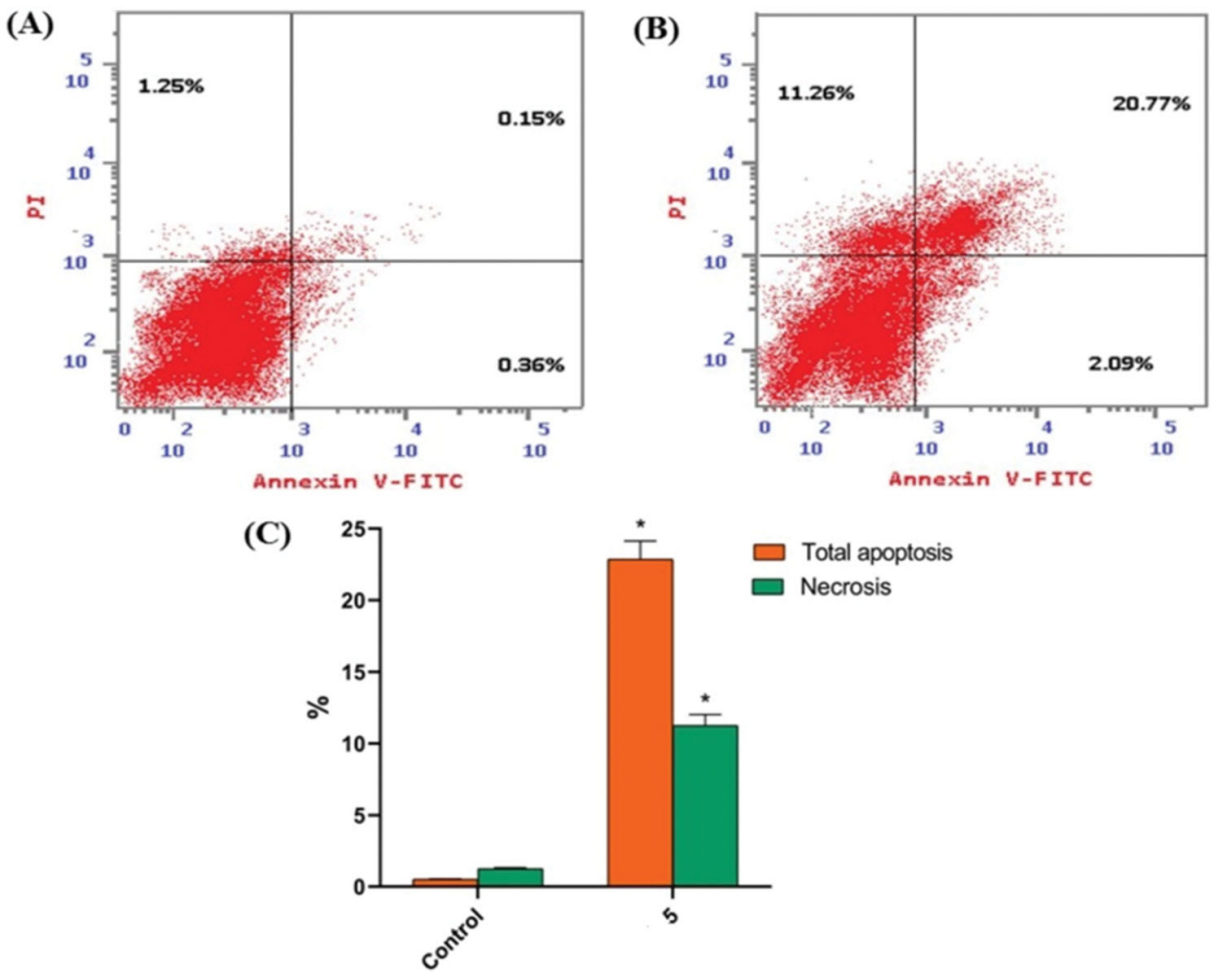
In HepG2 cells, compound **5** caused apoptosis. (A) Control, (B) Compound **5**, and (C) represent the graphical representation of the percent of apoptotic and necrotic cells in control (HepG2) cells and compound **5** treated cells. The percentages are given as the mean SEM of three different experiments. Using unpaired *t*-tests, *p < 0.05 indicates statistically significant differences from the untreated control (HepG2) group.

The apoptosis induced by compound **5** was quantified using an Annexin-V/propidium iodide (PI) staining assay. Compound **5** was applied to HepG2 cells at a concentration of 8.8 µM. As shown in Table 4 and Figure 4, compound **5** had a significantly higher apoptotic effect in HepG2 cells (22.86%) than in control cells (0.51%).

**Table 4.** Effect of compound **5** on the cell death process in HepG2 cells.

Sample	Apoptosis (%)	Necrosis (%)
HepG2	0.51 ± 0.03	1.25 ± 0.08
Compound 5/HepG2	22.86 ± 1.28*	11.26 ± 0.77*

Percentages equal mean ± SEM of three experiments. *p < 0.05 indicates a statistically significant difference from the corresponding control (HepG2) group using unpaired *t*-tests.

#### 2.2.7. Caspase-3 determination

**Figure 5.**
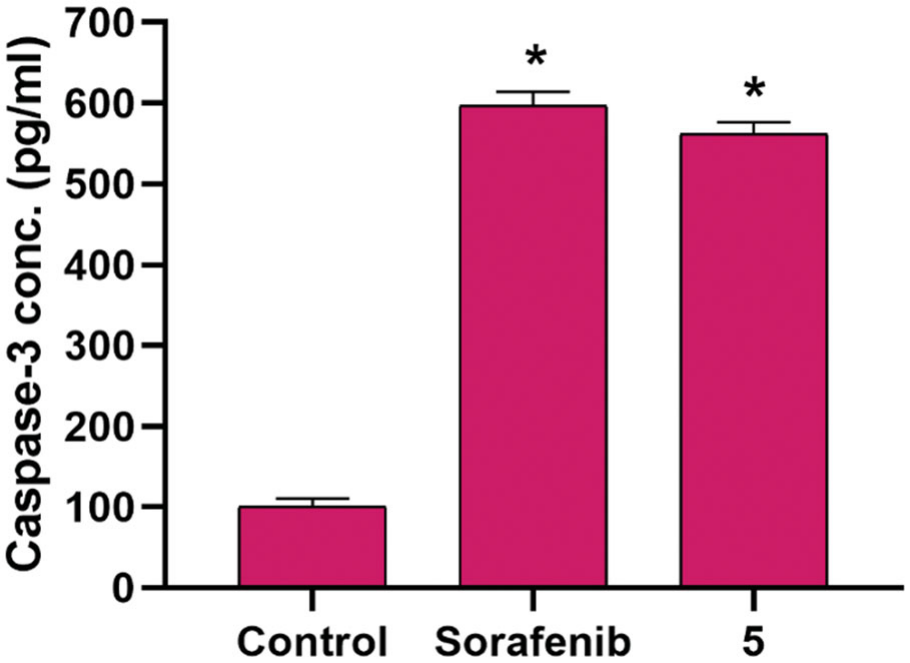
Effects of compound 5 on Caspase 3 level in HepG2 cells. Values are reported as mean ± SEM of three different experiments. *p < 0.001 indicates statistically significant differences from the untreated control (HepG2) group using unpaired *t*-test.

To test the effect of the synthesised compounds on caspase-3 levels, the most promising member **5**, at a concentration of 8.8 µM, was applied to the most sensitive cells (HepG2) for 24 h. The results showed that compound **5** significantly increased the level of caspase-3 (5.61-fold) when compared to control cells (Figure 5).

### 2.3. In silico studies

#### 2.3.1. Docking studies

Docking studies were performed in this study to gain a better understanding of the binding modes of the synthesised compounds into the VEGFR-2 binding site (PDB ID: 2OH4). Sorafenib was used as a control drug. Table 5 displays the binding free energies (Δ*G*). Asp1044 and Glu883 have been identified as key amino acids involved in the binding of VEGFR-2 inhibitors^33^^,^^48^.

**Table 5.** Results of docking scores.

Comp.	Δ*G* (kcal/mol)	Comp.	Δ*G* (kcal/mol)
**5**	−23.66	**13**	−22.88
**6**	−23.51	**14**	−23.51
**7**	−24.74	**15**	−23.38
**8**	−24.98	**16**	−23.91
**9**	−24.44	**17**	−23.78
**10**	−24.93	**18**	−23.40
**11**	−25.31	Sorafenib	−22.46
**12**	−24.96		

**Figure 6.**
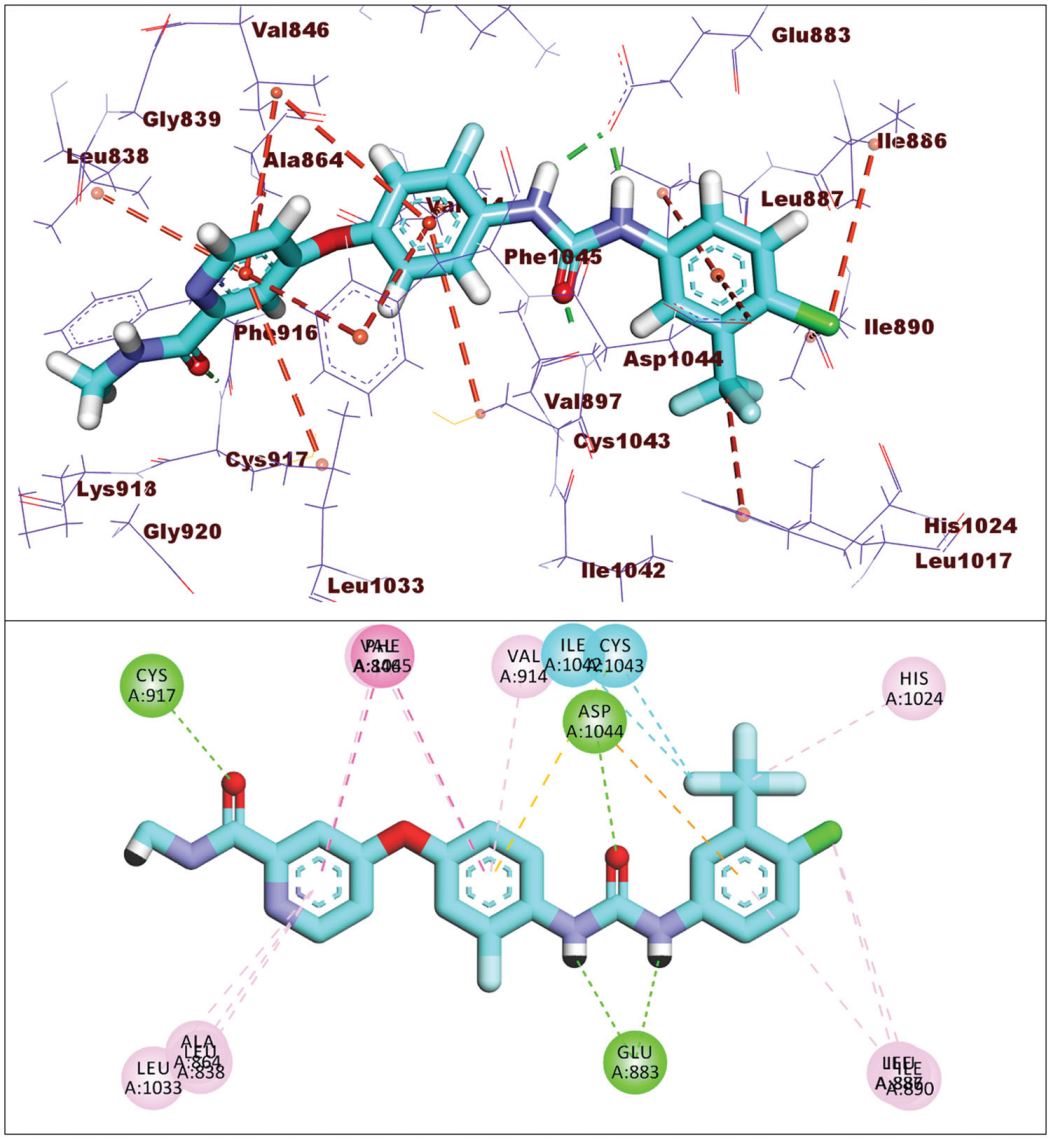
Interaction of sorafenib with VEGFR-2.

Sorafenib had a binding affinity of −22.46 kcal/mol. Three hydrogen bonding interactions occurred between the urea moiety and Glu883 and Asp1044. Four hydrophobic interactions were formed by the central phenyl group with Val914, Val846, Cys1043, and Phe1045. The hinge region was occupied by the *N*-methylpicolinamide moiety, which formed one hydrogen bond with Cys917 and five hydrophobic interactions with Val846, Leu838, Leu1033, Phe1045, and Ala864. The allosteric binding pocket was occupied by the terminal 1-chloro-2-(trifluoromethyl)benzene moiety, which formed five hydrophobic interactions with His1024, Ile890, Ile886, and Leu887. It also had one electrostatic interaction with Asp1044 (Figure 6).

The findings revealed that the synthesised compounds have a binding mode similar to that of sorafenib. Table 5 summarises the generated Δ*G* (binding energies) against VEGFR-2.

**Figure 7.**
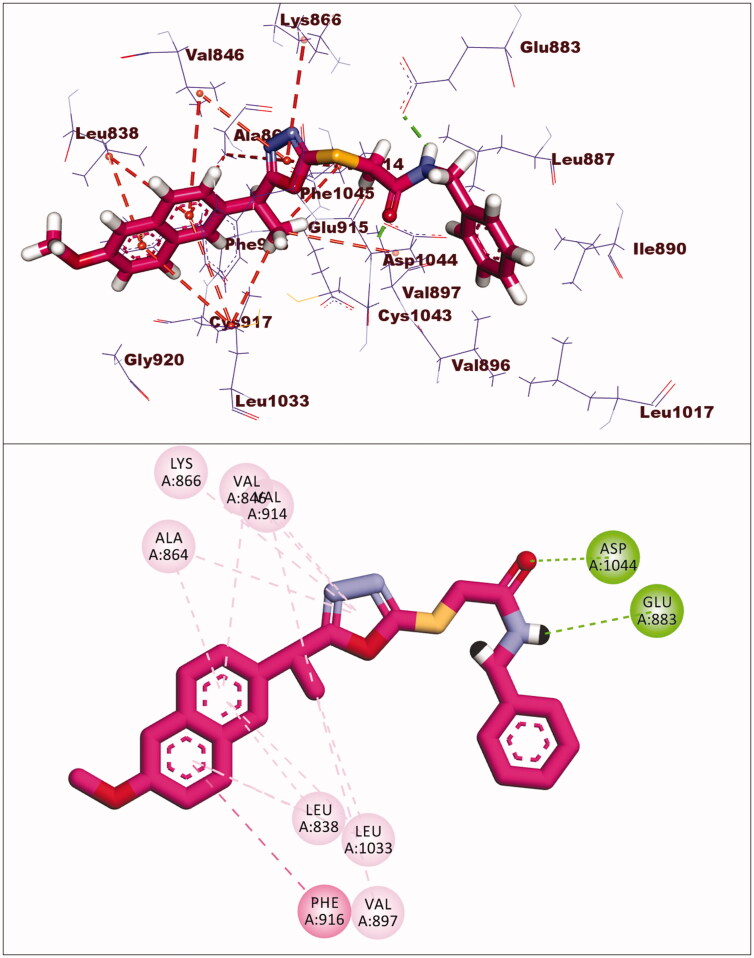
Interaction of compound **5** with VEGFR-2.

The docking score for compound **5** was −23.66 kcal/mol. Through its amide group, it formed two hydrogen bonds with Glu883 and Asp1044. The spacer moiety (2-ethyl-1,3,4-oxadiazole) interacted hydrophobically with Lys886, Val914, Val846, Val864, Val897, and Leu1033. Many hydrophobic interactions in the hinge region occurred between the 2-methoxynaphthalene moiety and different amino acid residues including Leu1033, Leu838, Val846, Val864, and Phe916. Furthermore, the hydrophobic group was positioned in the allosteric pocket, in close proximity to Ile890 and Leu887 (Figure 7).

**Figure 8.**
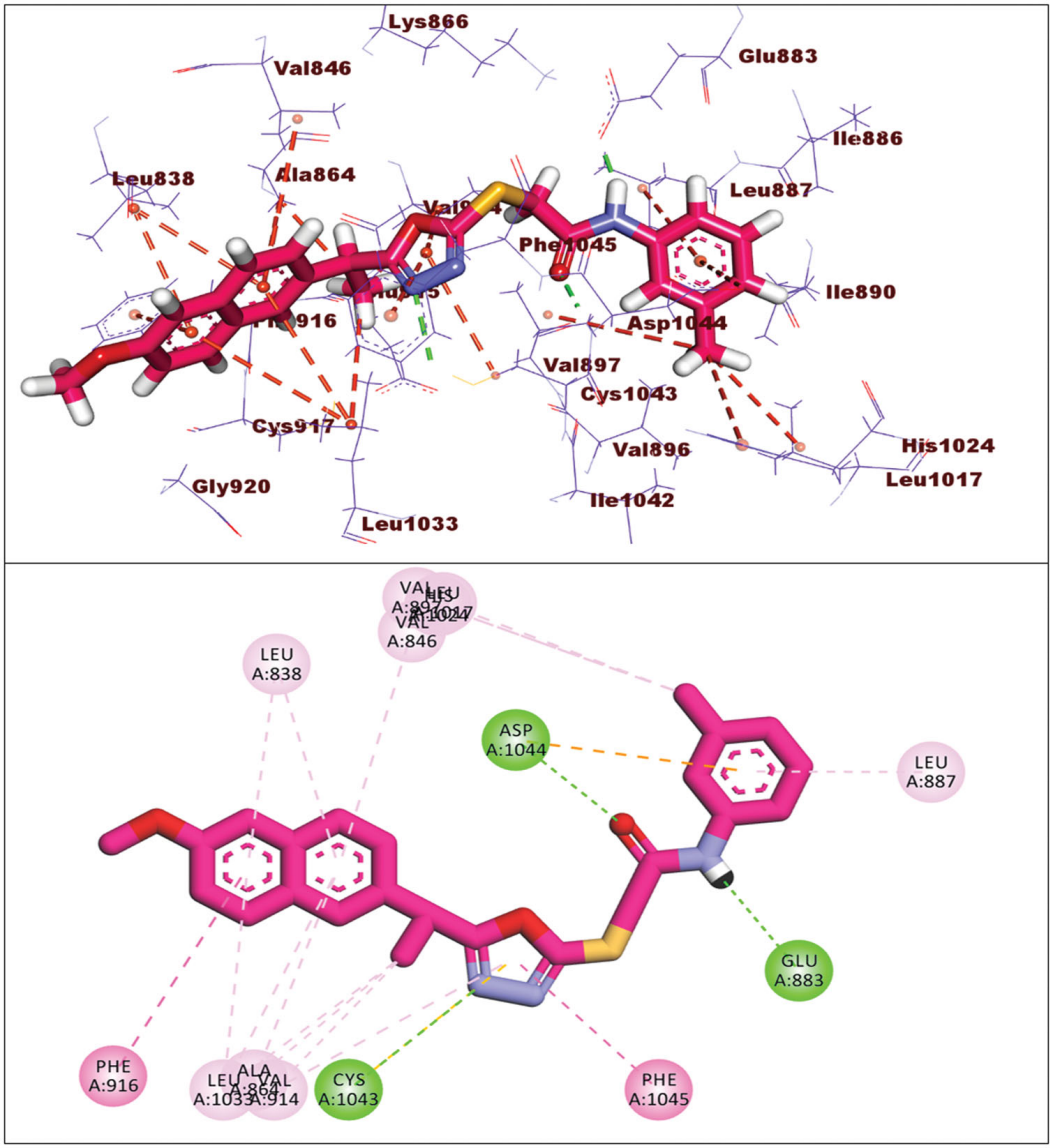
Interaction of compound **8** with VEGFR-2.

The binding energy of compound **8** was −24.98 kcal/mol. Two hydrogen bonds were formed between the pharmacophore (amide) group and Glu883 and Asp1044. Six hydrophobic interactions were formed by the 2-ethyl-1,3,4-oxadiazole moiety with Val914, Cys1043, Val864, and Leu1033. Furthermore, it formed an additional hydrogen bond with Cys1043. The 2-methoxynaphthalene moiety formed seven hydrophobic bonds, which were Leu1033, Leu838, Val846, Val864, and Phe916. In the allosteric pocket, the m-tolyl group formed numerous hydrophobic bonds with Leu1017, His1024, Val897, and Leu887. It also had an electrostatic interaction with Asp1044 (Figure 8).

**Figure 9.**
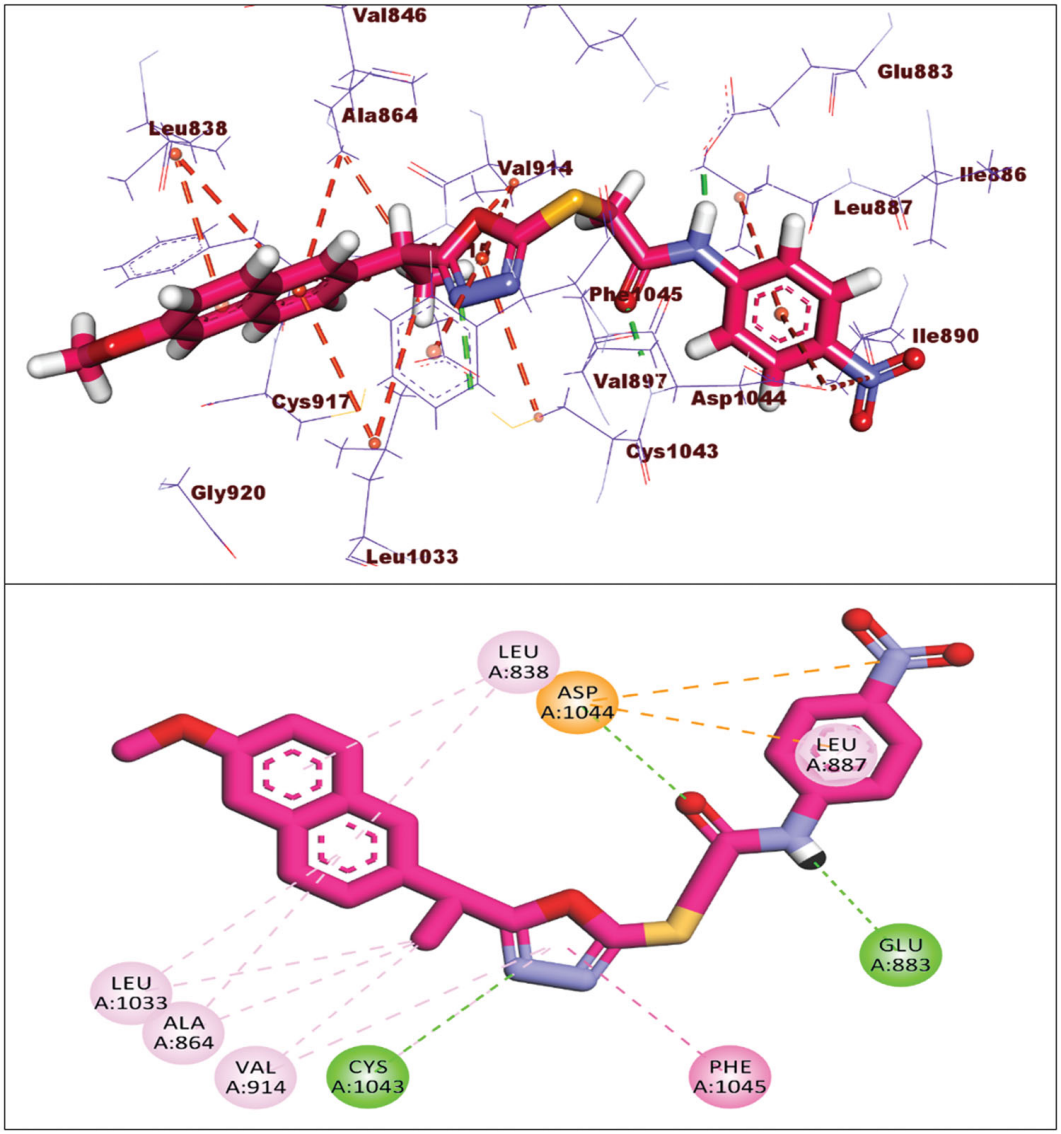
Interaction of compound **17** with VEGFR-2.

**Figure 10.**
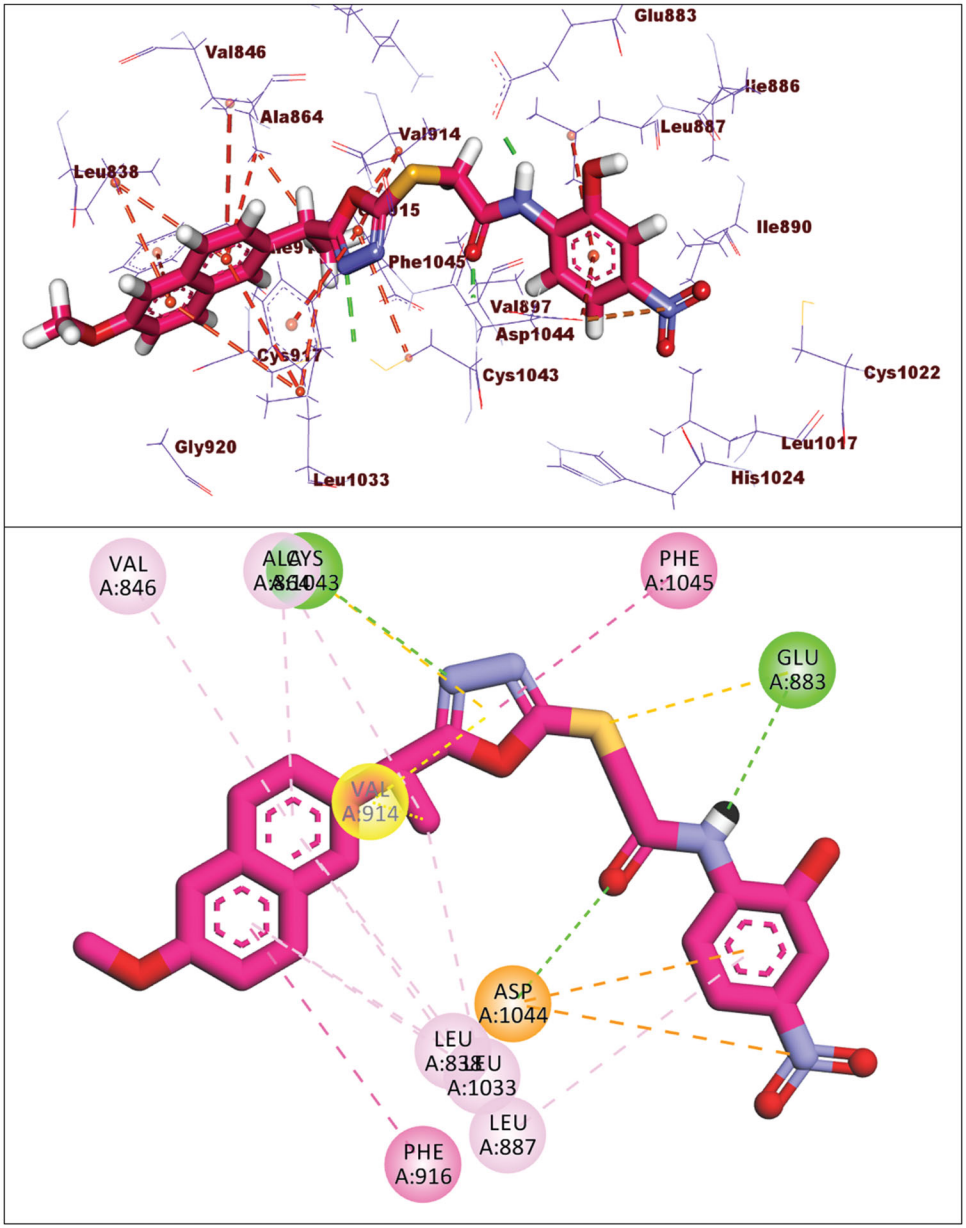
Predicted binding mode of compound **18** with the active site of VEGFR-2.

Compounds **17** and **18** exhibited binding energies of −23.78 and −23.40 kcal/mol, respectively. The pharmacophore groups in each compound formed two hydrogen bonds with Glu883 and Asp1044. The 2-ethyl-1,3,4-oxadiazole moiety in each compound formed many hydrophobic interactions in the linker region with extra hydrogen bonding with Cys1043. The 2-methoxynaphthalene moiety of compounds **17** and **18** formed four and seven hydrophobic bonds, respectively. The *p*-nitrophenyl of compound **17** and 2-hydroxy-4-nitrophenyl of compound **18** were oriented into the allosteric binding pocket-forming two electrostatic interactions with Asp1044 and one hydrophobic interaction with Leu887 (Figures 9 and 10).

#### 2.3.2. In silico ADMET studies

Discovery studio 4.0 was used to investigate the pharmacokinetic properties (ADMET studies) of the synthesised compounds. As a control molecule, sorafenib was used. Table 6 summarises the ADMET parameter values.

**Table 6.** Predicted ADMET for the designed compounds and reference drug.

Comp.	BBB level	Solubility level	Absorption level	CYP2D6 prediction	PPB prediction
**5**	Medium	Low	Good	Non-inhibitor	More than 90%
**6**	Medium	Low	Good	Non-inhibitor	More than 90%
**7**	High	Low	Good	Non-inhibitor	More than 90%
**8**	High	Low	Good	Non-inhibitor	More than 90%
**9**	High	Low	Good	Non-inhibitor	More than 90%
**10**	High	Very low	Good	Non-inhibitor	More than 90%
**11**	Medium	Low	Good	Non-inhibitor	More than 90%
**12**	High	Low	Good	Non-inhibitor	More than 90%
**13**	High	Very low	Good	Non-inhibitor	More than 90%
**14**	Very low	Very low	Moderate	Non-inhibitor	More than 90%
**15**	Very low	Very low	Moderate	Non-inhibitor	More than 90%
**16**	Very low	Low	Moderate	Non-inhibitor	More than 90%
**17**	Very low	Low	Moderate	Non-inhibitor	More than 90%
**18**	Very low	Low	Poor	Non-inhibitor	More than 90%
Sorafenib	Very low	Very low	Good	Non-inhibitor	More than 90%

**Figure 11.**
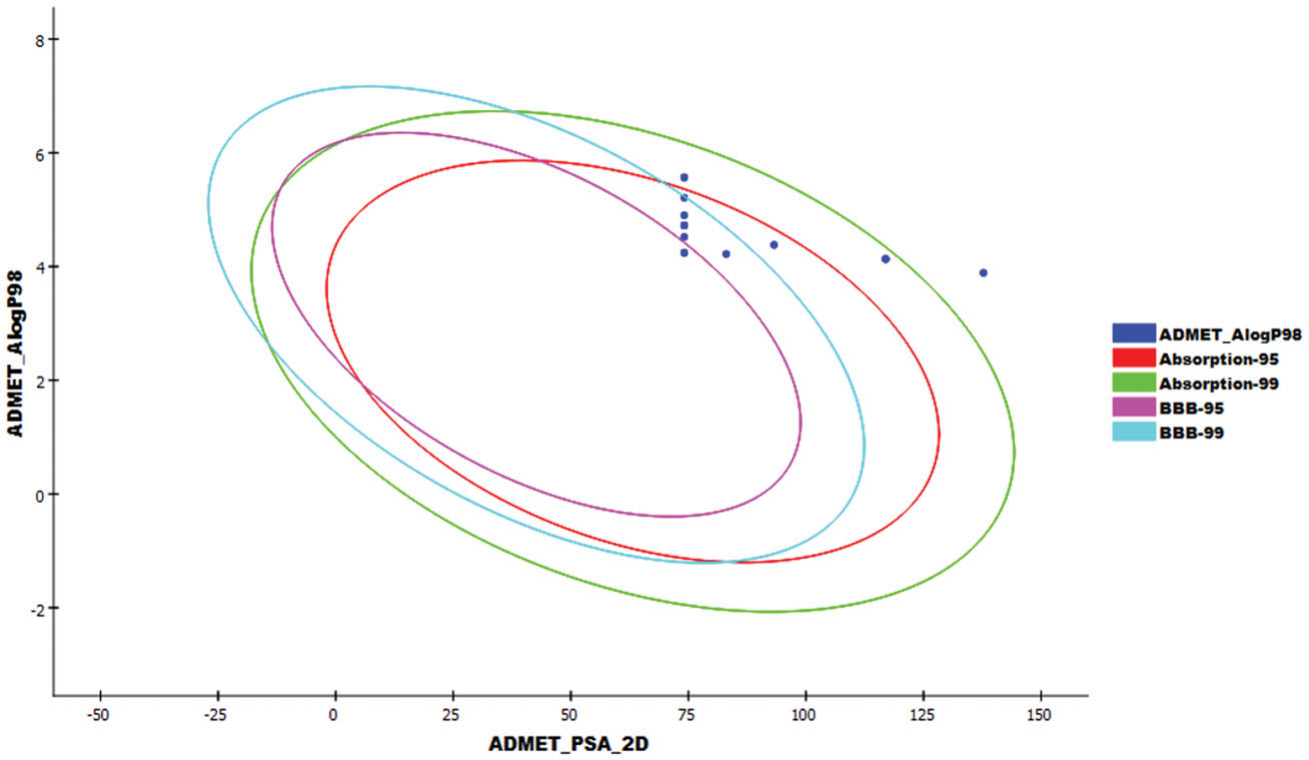
The expected ADMET studies.

Compounds **14–18** exhibited a very low level of BBB penetration, while compounds **5–13** had medium to high levels. The aqueous solubility of the synthesised compounds ranged from very low to very low. For absorption parameters, compounds **5–13** showed good levels, while compounds **14–17** were expected to have moderate absorption levels. Compound **18** showed poor absorption. In addition, all the tested compounds were anticipated to have a non-inhibitory effect against CYP2D6, and plasma protein binding ability of more than 90% (Figure 11).

#### 2.3.3. In silico toxicity studies

The synthesised compounds were examined *in silico* to reach the expected toxicity profile using Discovery Studio software^49^^,^^50^. As a control molecule, sorafenib was used. The toxicity studies include seven models: FDA rodent carcinogenicity, carcinogenic potency TD50, and carcinogenic potency TD_50_^51^, rat maximum tolerated dose^52^^,^^53^, rat oral LD_50_^54^, rat chronic LOAEL^55^^,^^56^, ocular irritancy^57^, and skin irritancy^57^ (Table 7).

**Table 7.** Toxicity properties of the synthesised compounds.

Comp.	FDA rodent carcinogenicity (mouse-female)	Carcinogenic potency TD_50_ (mouse)^a^	Rat maximum tolerated dose (feed)^b^	Rat oral LD_50_^b^	Rat chronic LOAEL^b^	Skin irritancy	Ocular irritancy
**5**	Non-carcinogen	15.349	0.042	0.700	0.032	Non-irritant	Mild
**6**	Non-carcinogen	20.304	0.054	0.876	0.031	Non-irritant	Mild
**7**	Multi-carcinogen	23.695	0.045	0.386	0.041	Non-irritant	Mild
**8**	Non-carcinogen	18.535	0.045	0.991	0.020	Non-irritant	Mild
**9**	Non-carcinogen	11.722	0.045	1.412	0.017	Non-irritant	Mild
**10**	Non-carcinogen	13.868	0.043	0.368	0.016	Non-irritant	Mild
**11**	Non-carcinogen	14.625	0.052	1.614	0.020	Non-irritant	Mild
**12**	Non-carcinogen	9.203	0.042	0.246	0.026	Non-irritant	Mild
**13**	Non-carcinogen	6.298	0.066	0.915	0.012	Non-irritant	Mild
**14**	Non-carcinogen	4.964	0.053	0.473	0.014	Non-irritant	Mild
**15**	Non-carcinogen	6.520	0.053	0.660	0.015	Non-irritant	Mild
**16**	Non-carcinogen	22.826	0.040	2.753	0.028	Non-irritant	Mild
**17**	Non-carcinogen	9.752	0.040	1.832	0.014	Non-irritant	Mild
**18**	Non-carcinogen	21.184	0.132	1.669	0.032	Non-irritant	Mild
Sorafenib	Non-carcinogen	17.535	0.077	0.890	0.004	Non-irritant	Mild

^a^Unit: mg/kg body weight/day.

^b^Unit: g/kg body weight.

Except for compound **7**, all compounds were predicted to be non-carcinogenic. TD_50_ values for compounds **6**, **7**, **8**, **16**, and **18** were 20.304, 23.695, 18.535, 22.826, and 21.184 mg/kg body weight/day, respectively, when compared to sorafenib (TD_50_ = 17.535 mg/kg body weight/day). Except for compound **18** (rat maximum tolerated dose = 0.132 g/kg body weight), all compounds had lower rat maximum tolerated doses (ranging from 0.042 to 0.053 g/kg body weight) than sorafenib. The rat chronic LOAEL ranged from 0.012 to 0.041 g/kg body weight, which was higher than sorafenib (0.004 g/kg body weight). Furthermore, all compounds were predicted to be non-irritants in skin irritancy models and mild irritants in ocular irritancy models.

## 3. Conclusion

Fourteen 1,3,4-oxadiazole-naphthalene hybrids were synthesised and tested for their *in vitro* antiproliferative activity against two human cancer cell lines MCF-7 and HepG-2. Compounds **5**, **8**, **15**, **16**, **17**, and **18** exhibited promising cytotoxicity ranging from 8.4 to 10.4 µM, comparing to sorafenib (IC_50_ = 10.8 µM and 10.2 µM against MCF-7 and HepG2 cells, respectively). Compounds **5**, **8**, **15**, **16**, **17**, and **18** showed VEGFR-2 inhibitory effects with concentrations of 610.4, 583.7, 864.5, 834.9, 1067, and 1004 pg/ml, respectively. SAR study revealed that substitution at the hydrophobic tail with electron-withdrawing is more beneficial than substitution with the electron-donating group for cytotoxicity. Compound **5**, the most active counterpart, induced a significant increase in apoptosis (22.86% compared to 0.51% in the control) and arrested the HepG2 cell growth mostly at the Pre-G1 phase. Additionally, compound **5** exerted a significant increase in the level of caspase-3 (5.61-fold). Docking studies revealed that the new derivatives have the same binding mode of sorafenib against the VEGFR-2 active site. ADMET and toxicity studies appeared that the new members have a high degree of drug-likeness profile.

## 4. Experimental

### 4.1. Chemistry

#### 4.1.1. General

Reagents, solvent, and apparatus used in chemical synthesis were shown in Supplementary Data. Compounds **2**, **3**, and **4** were prepared according to the reported procedures^58^^,^^59^.

#### 4.1.2. General procedure for synthesis of compounds (5–18)

The appropriate chloroacetanilide (0.7 mmol) was added to a suspension of 5-(1-(6-methoxynaphthalen-2-yl)ethyl)-1,3,4-oxadiazole-2-thiol 4 (0.2 g, 0.7 mmol) and anhydrous sodium acetate (0.1 g, 1.1 mmol) in absolute ethanol (30 ml). For 2–4 h, the reaction mixture was heated under reflux. The precipitate was collected and recrystallized from absolute ethanol after cooling to room temperature to provide the desired products (**5–18**).

##### 4.1.2.1. N-Benzyl-2-{[5-(1-(6-methoxynaphthalen-2-yl)ethyl)-1,3,4-oxadiazol-2-yl]thio}acetamide (5)

Yellow solid (0.25 g–83%); mp = 192–194 °C; IR (KBr) cm^−1^: 3295 (NH), 3082 (CH aromatic), 2925 (CH aliphatic), 1675 (C=O amide), 1628 (C=N); ^1^H NMR (400 MHz, DMSO-*d*_6_) δ:8.76 (brs, 1H, NH), 7.78–7.12 (m, 11H, Ar-H), 4.56–4.54 (m, 1H, CH_3_-CH), 4.22 (s, 2H, S-CH_2_), 4.04 (s, 2H, Ph-CH_2_), 3.83 (s, 3H, O-CH_3_), 1.66 (d, *J* = 4.0 Hz, 3H, CH-CH_3_). ^13^C NMR (101 MHz, DMSO) δ: 170.48, 166.12, 162.66, 157.48, 139.15, 135.71, 133.93, 129.78, 128.77, 128.53, 128.0, 127.79, 127.27, 126.77, 126.26, 119.65, 106.16, 55.33, 42.71, 37.11, 36.21, 19.77; MS (*m/z*) 433; Anal. Calc. for: (C_24_H_23_N_3_O_3_S, Mwt = 433): C, 66.49; H, 5.35; N, 9.69; Found: C, 66.56; H, 5.41; N, 9.76%.

##### 4.1.2.2. 2-{[5-(1-(6-Methoxynaphthalen-2-yl)ethyl)-1,3,4-oxadiazol-2-yl]thio}-N-phenylacetamide (6)

Light yellow solid (0.23 g–80%) mp = 187–189 °C; IR (KBr) cm^−1^: 3300 (NH), 3075 (CH aromatic), 2922 (CH aliphatic), 1685 (C=O amide), 1633 (C=N); ^1^H NMR (400 MHz, DMSO-*d*_6_) δ:10.76 (brs, 1H, NH), 7.76–7.04 (m, 11H, Ar-H), 4.55–4.54 (m, 1H, CH_3_-CH), 4.22 (s, 2H, S-CH_2_), 3.83 (s, 3H, O-CH_3_), 1.70 (d, *J* = 8.0 Hz, 3H, CH-CH_3_). ^13^C NMR (101 MHz, DMSO) δ: 170.10, 166.57, 160.70, 158.27, 135.85, 133.98, 129.86, 129.75, 129.41, 129.36, 127.93, 127.70, 126.60, 126.32, 126.09, 119.65, 106.15, 55.82, 42.33, 37.11, 19.77; MS (*m/z*) 419; Anal. Calc. for: (C_23_H_21_N_3_O_3_S, Mwt = 419): C, 65.85; H, 5.05; N, 10.02; Found: C, 65.93; H, 5.09; N, 10.09%.

##### 4.1.2.3. 2-{[5-(1-(6-Methoxynaphthalen-2-yl)ethyl)-1,3,4-oxadiazol-2-yl]thio}-N-(o-tolyl)acetamide (7)

Light yellow solid (0.23 g–77%) mp = 190–192 °C; IR (KBr) cm^−1^: 3293 (NH), 3089 (CH aromatic), 2954 (CH aliphatic), 1685 (C=O amide), 1638 (C=N); ^1^H NMR (400 MHz, DMSO-*d*_6_) δ:10.36 (brs, 1H, NH), 7.74–7.15 (m, 10H, Ar-H), 4.53–4.51 (m, 1H, CH_3_-CH), 4.01 (s, 2H, S-CH_2_), 3.81 (s, 3H, O-CH_3_), 1.95 (s, 3H, Ph-CH_3_), 1.78 (d, *J* = 4.0 Hz, 3H, CH-CH_3_). ^13^C NMR (101 MHz, DMSO) δ: 171.77, 164.09, 157.53, 147.73, 136.45, 134.69, 133.66, 131.67, 131.23, 129.67, 129.62, 129.16, 128.74, 126.95, 125.82, 123.23, 122.12, 119.08, 106.31, 55.80, 44.0, 33.18, 18.64, 17.39; MS (*m/z*) 433; Anal. Calc. for: (C_24_H_23_N_3_O_3_S, Mwt = 433): C, 66.49; H, 5.35; N, 9.69; Found: C, 66.57; H, 5.43; N, 9.77%.

##### 4.1.2.4. 2-{[5-(1-(6-Methoxynaphthalen-2-yl)ethyl)-1,3,4-oxadiazol-2-yl]thio}-N-(m-tolyl) acetamide (8)

Light yellow solid (0.24 g–82%) mp = 193–195 °C; IR (KBr) cm^−1^: 3288 (NH), 3010 (CH aromatic), 2925 (CH aliphatic), 1682 (C=O amide), 1638 (C=N) ^1^H NMR (400 MHz, DMSO-*d*_6_) δ:10.36 (brs, 1H, NH), 7.75–7.09 (m, 10H, Ar-H), 4.96–4.93 (m, 1H, CH_3_-CH), 4.08 (s, 2H, S-CH_2_), 3.82 (s, 3H, O-CH_3_), 2.28 (s, 3H, Ph-CH_3_), 1.62 (d, *J* = 8.0 Hz, 3H, CH-CH_3_). ^13^C NMR (101 MHz, DMSO) δ: 172.60, 169.99, 160.92, 157.47, 139.16, 136.99, 135.32, 133.54, 133.49, 129.77, 129.20, 128.89, 128.81, 128.32, 127.07, 126.64, 125.76, 119.26, 106.15, 55.82, 43.60, 33.17, 21.05, 18.88; MS (*m/z*) 433; Anal. Calc. for: (C_24_H_23_N_3_O_3_S, Mwt = 433): C, 66.49; H, 5.35; N, 9.69; Found: C, 66.53; H, 5.39; N, 9.73%.

##### 4.1.2.5. 2-{[5-(1-(6-Methoxynaphthalen-2-yl)ethyl)-1,3,4-oxadiazol-2-yl]thio}-N-(p-tolyl)acetamide (9)

Light yellow solid (0.24 g–82%) mp = 198–200 °C; IR (KBr) cm^−1^: 3309 (NH), 3122 (CH aromatic), 2900 (CH aliphatic), 1665 (C=O amide), 1633 (C=N); ^1^H NMR (400 MHz, DMSO-*d*_6_) δ:10.49 (brs, 1H, NH), 7.83–7.01 (m, 10H, Ar-H), 4.53–4.48 (m, 1H, CH_3_-CH), 4.05 (s, 2H, S-CH_2_), 3.85 (s, 3H, O-CH_3_), 2.30 (s, 3H, Ph-CH_3_), 1.78 (d, *J* = 12.0 Hz, 3H, CH-CH_3_). ^13^C NMR (101 MHz, DMSO) δ: 172.22, 165.94, 158.70, 157.67, 137.51, 134.05, 133.02, 130.57, 129.17, 128.45, 128.14, 127.43, 126.41, 126.01, 123.65, 119.80, 106.96, 55.92, 44.43, 34.04, 20.81, 18.37; MS (*m/z*) 433; Anal. Calc. for: (C_24_H_23_N_3_O_3_S, Mwt = 433): C, 66.49; H, 5.35; N, 9.69; Found: C, 66.58; H, 5.46; N, 9.73%.

##### 4.1.2.6. N-(2,6-Dimethylphenyl)-2-{[5-(1-(6-methoxynaphthalen-2-yl)ethyl)-1,3,4-oxadiazol-2-yl]thio}acetamide (10)

Light yellow solid (0.26 g–85%) mp = 206–208 °C; IR (KBr) cm^−1^: 3295 (NH), 3082 (CH aromatic), 2925 (CH aliphatic), 1675 (C=O amide), 1628 (C=N); ^1^H NMR (400 MHz, DMSO-*d*_6_) δ:9.65 (brs, 1H, NH), 7.77–7.01 (m, 10H, Ar-H), 4.58–4.56 (m, 1H, CH_3_-CH), 4.22 (s, 2H, S-CH_2_), 3.83 (s, 3H, O-CH_3_), 2.05 (s, 6H, Ph-2CH_3_), 1.67 (d, *J* = 4.0 Hz, 3H, CH-CH_3_). ^13^C NMR (101 MHz, DMSO) δ: 171.53, 166.33, 161.83, 153.10, 137.83, 134.04, 133.02, 130.18, 129.17, 128.45, 128.14, 127.75, 127.42, 126.01, 123.96, 119.47, 106.42, 55.92, 44.75, 33.33, 22.54, 18.76; MS (*m/z*) 447; Anal. Calc. for: (C_25_H_25_N_3_O_3_S, Mwt = 447): C, 67.09; H, 5.63; N, 9.39; Found: C, 67.16; H, 5.69; N, 9.47%.

##### 4.1.2.7. 2-{[5-(1-(6-Methoxynaphthalen-2-yl)ethyl)-1,3,4-oxadiazol-2-yl]thio}-N-(3-methoxyphenyl)acetamide (11)

Brownish yellow solid (0.25 g–82%) mp = 209–212 °C; IR (KBr) cm^−1^: 3295 (NH), 3080 (CH aromatic), 2900 (CH aliphatic), 1688 (C=O amide), 1631 (C=N); ^1^H NMR (400 MHz, DMSO-*d*_6_) δ:10.37 (brs, 1H, NH), 7.76–7.06 (m, 10H, Ar-H), 4.56–4.54 (m, 1H, CH_3_-CH), 4.21 (s, 2H, S-CH_2_), 3.83 (s, 3H, O-CH_3_), 3.68 (s, 3H, O-CH_3_), 1.66 (d, *J* = 8.0 Hz, 3H, CH-CH_3_). ^13^C NMR (101 MHz, DMSO) δ: 170.47, 165.32, 163.93, 158.81, 157.77, 140.44, 135.40, 133.93, 129.92, 129.48, 128.68, 127.71, 126.45, 126.18, 119.26, 112.01, 109.56, 106.35, 105.59, 55.74, 55.60, 42.88, 36.91, 19.71; MS (*m/z*) 449; Anal. Calc. for: (C_24_H_23_N_3_O_4_S, Mwt = 449): C, 64.13; H, 5.16; N, 9.35; Found: C, 64.15; H, 5.21; N, 9.39%.

##### 4.1.2.8. N-Cyclohexyl-2-{[5-(1-(6-methoxynaphthalen-2-yl)ethyl)-1,3,4-oxadiazol-2-yl]thio}acetamide (12)

Yellow solid (0.23 g–78%) mp = 188–191 °C; IR (KBr) cm^−1^: 3261 (NH), 3071 (CH aromatic), 2944 (CH aliphatic), 1681 (C=O amide), 1630 (C=N); ^1^H NMR (400 MHz, DMSO-*d*_6_) δ:8.12 (brs, 1H, NH), 7.78–7.13 (m, 6H, Ar-H), 4.57–4.49 (m, 1H, CH_3_-CH), 4.94 (s, 2H, S-CH_2_), 3.83 (s, 3H, O-CH_3_), 1.66–1.04 (m, 14H, CH-CH_3_ and cyclohexyl 11H). ^13^C NMR (101 MHz, DMSO) δ: 170.15, 165.34, 163.72, 157.87, 135.87, 129.75, 128.88, 127.93, 126.44, 126.12, 126.0, 119.66, 106.15, 105.59, 55.78, 48.68, 36.81, 36.39, 32.50, 25.53, 24.85, 19.69; MS (*m/z*) 425; Anal. Calc. for: (C_23_H_27_N_3_O_3_S, Mwt = 425): C, 64.92; H, 6.40; N, 9.87; Found: C, 64.99; H, 6.47; N, 9.95%.

##### 4.1.2.9. N-(4-Chlorophenyl)-2-{[5-(1-(6-methoxynaphthalen-2-yl)ethyl)-1,3,4-oxadiazol-2-yl]thio}acetamide (13)

Brownish yellow solid (0.23 g–78%) mp = 214–216 °C; IR (KBr) cm^−1^: 3321 (NH), 3088 (CH aromatic), 2954 (CH aliphatic), 1687 (C=O amide), 1644 (C=N); ^1^H NMR (400 MHz, DMSO-*d*_6_) δ:10.49 (brs, 1H, NH), 7.75–7.11 (m, 10H, Ar-H), 4.56–4.54 (m, 1H, CH_3_-CH), 4.21 (s, 2H, S-CH_2_), 3.83 (s, 3H, O-CH_3_), 1.65 (d, *J* = 8.0 Hz, 3H, CH-CH_3_). ^13^C NMR (101 MHz, DMSO) δ: 173.20, 164.66, 157.86, 134.0, 131.78, 131.08, 130.50, 129.77, 129.32, 129.21, 127.91, 127.20, 126.90, 126.34, 126.07, 121.33, 119.44, 106.39, 55.69, 43.51, 37.17, 19.41; MS (*m/z*) 453; Anal. Calc. for: (C_23_H_20_ClN_3_O_3_S, Mwt = 453): C, 60.86; H, 4.44; N, 9.26; Found: C, 60.94; H, 4.52; N, 9.31%.

##### 4.1.2.10. N-(2,4-Dichlorophenyl)-2-{[5-(1-(6-methoxynaphthalen-2-yl)ethyl)-1,3,4-oxadiazol-2-yl]thio}acetamide (14)

Brownish yellow solid (0.24 g–72%) mp = 226–228 °C; IR (KBr) cm^−1^: 3298 (NH), 3068 (CH aromatic), 2914 (CH aliphatic), 1688 (C=O amide), 1635 (C=N); ^1^H NMR (400 MHz, DMSO-*d*_6_) δ:10.58 (brs, 1H, NH), 8.36 (s, 1H, Ph-H3), 7.71–7.12 (m, 8H, Ar-H), 4.56–4.54 (m, 1H, CH_3_-CH), 4.24 (s, 2H, S-CH_2_), 3.82 (s, 3H, O-CH_3_), 1.69 (d, *J* = 8.0 Hz, 3H, CH-CH_3_). ^13^C NMR (101 MHz, DMSO) δ: 170.47, 163.57, 159.60, 157.48, 152.58, 147.60, 141.82, 136.99, 134.43, 133.54, 131.76, 131.37, 129.59, 128.32, 127.04, 125.75, 118.76, 116.20, 106.15, 55.83, 44.09, 34.55, 18.88; MS (*m/z*) 487; Anal. Calc. for: (C_23_H_19_Cl_2_N_3_O_3_S, Mwt = 487): C, 56.56; H, 3.92; N, 8.60; Found: C, 56.61; H, 3.97; N, 8.64%.

##### 4.1.2.11. N-(2,6-Dichlorophenyl)-2-{[5-(1-(6-methoxynaphthalen-2-yl)ethyl)-1,3,4-oxadiazol-2-yl]thio}acetamide (15)

Brownish yellow solid (0.23 g–68%) mp = 223–225 °C; IR (KBr) cm^−1^: 3311 (NH), 3082 (CH aromatic), 2923 (CH aliphatic), 1687 (C=O amide), 1630 (C=N); ^1^H NMR (400 MHz, DMSO-*d*_6_) δ:10.57 (brs, 1H, NH), 7.71–7.10 (m, 9H, Ar-H), 4.62–4.57 (m, 1H, CH_3_-CH), 4.34 (s, 2H, S-CH_2_), 3.79 (s, 3H, O-CH_3_), 1.38 (d, *J* = 12.0 Hz, 3H, CH-CH_3_). ^13^C NMR (101 MHz, DMSO) δ: 170.28, 160.10, 157.38, 137.44, 134.26, 133.75, 132.52, 129.79, 129.54, 128.84, 128.77, 127.13, 126.83, 125.88, 125.37, 119.24, 106.17, 55.46, 43.61, 33.0, 18.77; MS (*m/z*) 487; Anal. Calc. for: (C_23_H_19_Cl_2_N_3_O_3_S, Mwt = 487): C, 56.56; H, 3.92; N, 8.60; Found: C, 56.59; H, 3.97; N, 8.64%.

##### 4.1.2.12. 2-{[5-(1-(6-Methoxynaphthalen-2-yl)ethyl)-1,3,4-oxadiazol-2-yl]thio}-N-(2-nitrophenyl) acetamide (16)

Yellow solid (0.19 g–60%) mp = 228–230 °C; IR (KBr) cm^−1^: 3333 (NH), 3069 (CH aromatic), 2974 (CH aliphatic), 1688 (C=O amide), 1628 (C=N); ^1^H NMR (400 MHz, DMSO-*d*_6_) δ:10.22 (brs, 1H, NH), 8.0–7.11 (m, 10H, Ar-H), 4.54–4.52 (m, 1H, CH_3_-CH), 4.25 (s, 2H, S-CH_2_), 3.82 (s, 3H, O-CH_3_), 1.56 (d, *J* = 8.0 Hz, 3H, CH-CH_3_). ^13^C NMR (101 MHz, DMSO) δ: 170.50, 166.33, 162.86, 159.0, 157.28, 153.81, 146.16, 134.04, 133.33, 131.92, 130.58, 129.48, 127.12, 126.40, 123.96, 118.76, 116.31, 106.95, 55.54, 43.41, 33.64, 18.77; MS (*m/z*) 464; Anal. Calc. for: (C_23_H_20_N_4_O_5_S, Mwt = 464): C, 59.47; H, 4.34; N, 12.06; Found: C, 59.54; H, 4.41; N, 12.11%.

##### 4.1.2.13. 2-{[5-(1-(6-Methoxynaphthalen-2-yl)ethyl)-1,3,4-oxadiazol-2-yl]thio}-N-(4-nitrophenyl) acetamide (17)

Yellow solid (0.20 g–62%) mp = 232–234 °C; IR (KBr) cm^−1^: 3298 (NH), 3060 (CH aromatic), 2931 (CH aliphatic), 1671 (C=O amide), 1632 (C=N); ^1^H NMR (400 MHz, DMSO-*d*_6_) δ:10.37 (brs, 1H, NH), 8.0 (d, *J* = 8.0 Hz, 2H, Ph-H3,H5), 7.76–7.10 (m, 8H, Ar-H), 4.52–4.48 (m, 1H, CH_3_-CH), 4.27 (s, 2H, S-CH_2_), 3.84 (s, 3H, O-CH_3_), 1.64 (d, *J* = 8 Hz, 3H, CH-CH_3_). ^13^C NMR (101 MHz, DMSO) δ: 173.26, 167.36, 163.58, 159.0, 154.83, 150.35, 137.12, 134.04, 133.02, 130.19, 129.16, 128.45, 126.41, 125.71, 123.97, 119.08, 106.56, 55.53, 43.27, 33.65, 18.76; MS (*m/z*) 464; Anal. Calc. for: (C_23_H_20_N_4_O_5_S, Mwt = 464): C, 59.47; H, 4.34; N, 12.06; Found: C, 59.56; H, 4.40; N, 12.11%.

##### 4.1.2.14. N-(2-Hydroxy-4-nitrophenyl)-2-{[5-(1-(6-methoxynaphthalen-2-yl)ethyl)-1,3,4-oxadiazol-2-yl]thio}acetamide (18)

Yellow solid (0.20 g–62%) mp = 237–240 °C; IR (KBr) cm^−1^: 3435 (OH), 3300 (NH), 3075 (CH aromatic), 2922 (CH aliphatic), 1685 (C=O amide), 1633 (C=N); ^1^H NMR (400 MHz, DMSO-*d*_6_) δ:10.41 (brs, 1H, OH), 10.29 (brs, 1H, NH), 8.29–7.14 (m, 9H, Ar-H), 4.27–4.25 (m, 1H, CH_3_-CH), 4.24 (s, 2H, S-CH_2_), 3.83 (s, 3H, O-CH_3_), 1.66 (d, *J* = 12.0 Hz, 3H, CH-CH_3_). ^13^C NMR (101 MHz, DMSO) δ: 172.24, 166.33, 162.16, 160.11, 153.49, 147.20, 134.36, 133.02, 132.63, 129.87, 129.16, 128.46, 128.13, 127.74, 127.43, 126.41, 123.97, 119.48, 106.57, 55.53, 43.72, 33.33, 18.76; MS (*m/z*) 480; Anal. Calc. for: (C_23_H_20_N_4_O_6_S, Mwt = 480): C, 57.49; H, 4.20; N, 11.66; Found: C, 57.57; H, 4.28; N, 11.72%.

### 4.2. Biological testing

#### 4.2.1. Cell culture

The MCF-7, HepG2, and THLE-2 cell lines were purchased from the American Type Culture Collection (ATCC, Rockville, MD, USA). MCF-7 and HepG2 cells were grown in a medium that contains Roswell Park Memorial Institute (RPMI) 1640 with L-glutamine, 10% foetal bovine serum (FBS), streptomycin (100 μg/ml) and penicillin (100 U/ml). While THLE-2 cells were cultivated in Bronchial Epithelial Growth Media (BEGM) with 10% FBS, 5 ng/ml EGF, and 70 ng/ml phosphoethanolamine. All cells were maintained in a humidified incubator and 5% (v/v) CO_2_ atmosphere at 37 °C^60^.

#### 4.2.2. In vitro cytotoxic activity

The MCF-7, HepG2, and THLE-2 cells were tested for cytotoxicity using the 3–(4, 5-dimethylthiazol-2-yl)-2,5-diphenyltetrazolium bromide (MTT) assay^61–65^. The ability of living cells to reduce the yellow product MTT to a blue product, formazan, *via* a reduction reaction that occurs in the mitochondria is used to assess cell population growth. In this assay, 5000 cells/well were plated in a 96-well plate and allowed to grow for 24 h before being treated with over-mentioned suitable media containing increased concentrations of tested compounds (0, 0.1, 1, 10, 100, and 1000 µM). Each experiment was conducted in triplicate. After removing the media, 100 µL of MTT was added to each well and incubated for 4 h. Following that, 100 µL of dimethyl sulfoxide (DMSO) solution was added to solubilise the resulting formazan product, and absorbance at 570 nm was measured using an ELISA microplate reader (Epoc-2 C micro-plate reader, Bio Tek, VT, USA). The IC_50_ values [the concentration required to inhibit cell viability by 50%] were calculated.

#### 4.2.3. Assessment of VEGFR-2 level

*In vitro* VEGFR-2 concentration was evaluated using Enzyme-Linked Immunosorbent Assay (ELISA) kit (Cat. NO. EK0544) (AVIVA System Biology, USA) according to manufacturer instructions^66^.

#### 4.2.4. Cell cycle analysis using flow cytometry

The effect of compound **5** on the cell cycle phases was investigated using propidium iodide staining and flow cytometric analysis according to the cell cycle kit (PN C03551). Briefly, HepG2 cells were allowed to grow in 25 cm^3^ flask, then treated with compound **5** for 48 h. After that, the cells were harvested and cell fixation was performed, cells were centrifuged at 2000 rpm for 5 min then, the supernatant was aspirated. The pellet of fixed cells was resuspended in a 0.5 ml cell cycle kit, vortexed, and incubated at 25 °C for 15 min. Finally, DNA was stained with 50 µg/ml propidium iodide for 30 min. Flow cytometric analysis of cell cycle performed on a COULTER^®^ EPICS^®^ XL™ Flow Cytometer (USA)^67–70^.

#### 4.2.5. Flow cytometric analysis for detection of apoptosis

To assess the effect of compound **5** on cell apoptosis, Annexin V–FITC Kit was used according to the kit protocol (PN IM3546), followed by flow cytometric analysis. In brief, HepG2 cells were allowed to grow in 25 cm^3^ flask, after that treated for 48 h. then, washed in phosphate-buffered saline and suspended to 5 × 10^5^–5 × 10^6^ cells/mL in 1X binding buffer. Then we added to 100 µL of the cell suspensions, 5 µL of dissolved PI, and 1 µL of annexin VFITC solution and incubated in the dark for 15 min. Next to that, we added 400 µL of ice-cold 1X binding buffer and mixed gently. Apoptotic cells were determined by flow cytometric analysis on a COULTER^®^ EPICS^®^ XL™ Flow Cytometer (USA)^67^^,^^71^^,^^72^.

#### 4.2.6. Caspase-3 determination

The effect of compound **5** on the Caspase-3 level was assessed using an ELISA kit (Catalog # KHO1091) according to manufacturer instructions.

### 4.3. In silico studies

#### 4.3.1. Docking studies

Docking studies were performed on VEGFR-2 [PDB ID: PDB ID: 2OH4, resolution: 2.05] using the reported procedure^45^^,^^73–76^ as described in Supplementary Data.

#### 4.3.2. ADMET studies

The ADMET descriptors were calculated using Discovery Studio 4.0 in accordance with the reported method^73^^,^^74^^,^^77^ (Supplementary Data).

#### 4.3.3. Toxicity studies

The toxicity potential of the synthesised compounds was predicted using the Discovery studio 4.0 software, as reported in Supplementary Data^78^.

## Supplementary Material

Supplemental Material
