# Supplementary material for: 1,3,4-Oxadiazole-naphthalene hybrids as potential VEGFR-2 inhibitors: design, synthesis, antiproliferative activity, apoptotic effect, and in silico studies
Source: J Enzyme Inhib Med Chem. 2021 Dec 20;37(1):380–96. doi: 10.1080/14756366.2021.2015342 (PMC8725909; doi:10.1080/14756366.2021.2015342)
Supplement: Supplemental Material [file IENZ_A_2015342_SM4095.pdf]

1,3,4-oxadiazole-naphthalene hybrids as potential VEGFR-2 inhibitors: Design, synthesis, antiproliferative activity, apoptotic effect, and *in silico* studies

Mohamed Hagras*[a], Marwa A. Saleh[b], Rogy R. Ezz Eldin[c], Abdelrahman A. Abuelkhir[a], Emad Gamil Khidr[d], Ahmed A. El-Husseiny[d], Hesham A. El-Mahdy[d], Eslam B. Elkaeed[e], Ibrahim H. Eissa*[f]

[a] Pharmaceutical Organic Chemistry, Faculty of Pharmacy (Boys), Al-Azhar University, Cairo 11884, Egypt

[b] Pharmaceutical Organic Chemistry, Faculty of Pharmacy (Girls), Al-Azhar University, Cairo 11884, Egypt

[c] Department of Pharmaceutical Organic Chemistry, Faculty of Pharmacy, Port Said University, Port Said, Egypt

[d] Biochemistry and Molecular Biology Department, Faculty of Pharmacy (Boys), Al-Azhar University, Cairo 11884, Egypt.

[e] Department of Pharmaceutical Sciences, College of Pharmacy, AlMaarefa University, Ad Diriyah 13713, Riyadh, Saudi Arabia

[f] Pharmaceutical Medicinal Chemistry & Drug Design Department, Faculty of Pharmacy (Boys), Al-Azhar University, Cairo 11884, Egypt

*** Corresponding authors:**

Ibrahim H. Eissa

Pharmaceutical Medicinal Chemistry & Drug Design Department, Faculty of Pharmacy (Boys), Al-Azhar University, Cairo, 11884, Egypt

Email: Ibrahimeissa@azhar.edu.eg

Mohamed Hagras

Pharmaceutical Organic Chemistry, Faculty of Pharmacy (Boys), Al-Azhar University, Cairo, 11884, Egypt

E-mail: m.hagrs@azhar.edu.eg

Content

**1**	The used chemicals, reagents, and different apparatus
**2**	*In silico* studies procedures
**3**	Spectral data
**4**	*In silico* toxicity data

The used chemicals, reagents, and different apparatus

^{1}H NMR spectra were run at 400 MHz and ^{13}C spectra were determined at 100 MHz in deuterated dimethyl sulfoxide (DMSO-d_6) on a Varian Mercury VX-400 NMR spectrometer. Chemical shifts are given in parts per million (ppm) on the delta (δ) scale. Chemical shifts were calibrated relative to those of the solvents. The progress of reactions was monitored with Merck silica gel IB2-F plates (0.25 mm thickness). The infrared spectra were recorded in potassium bromide disks on pye Unicam SP 3300 and Shimadzu FT IR 8101 PC infrared spectrophotometer at Faculty of Pharmacy-Ain Shams University. Mass spectra were recorded on Hewlett Packard 5988 spectrometer at Regional Center for Mycology and Biotechnology, Al-Azhar University. Elemental analyses were performed on a Thermo Scientific Flash 2000 elemental analyzer at the Regional Center for Mycology and Biotechnology, Al-Azhar University. Melting points were determined using capillary tubes with a Stuart SMP30 apparatus and are uncorrected. All yields reported refer to isolated yields.

***In silico* studies procedures**

1- Molecular docking

The crystal structure of VEGFR-2 was downloaded from the Protein Data Bank, http://www.rcsb.org/pdb (PDB ID: 2OH4, resolution: 2.05 Å). Discovery Studio 4.0 software was used in the docking studies. At first, the target molecule was prepared by removal of the water molecules. Any crystallographic disorders and unfilled valence atoms were corrected using alternate conformations and valence monitor options. Then, the protein structure was subjected to energy minimization by applying CHARMm force fields for charge, and MMFF94 force field for partial charge. Inflexibility of structure was obtained by creating fixed atom constraint. The binding site of the protein was defined and prepared for docking. Sorafenib and the designed compounds 2D structures were sketched using ChemBioDraw Ultra 14.0 and saved in MDL-SD file format. Next, the SD file was opened, 3D structures were protonated, and energy was minimized by applying CHARMm force fields for charge andMMFF94 force field for partial charge and then prepared for docking by optimization of the parameters. Docking process was accomplished using CDOCKER-CHARMm-based technique in the interface of Accelry's Discovery Studio 4.0. A maximum of 10 conformers was considered for each molecule in the docking analysis. After that the docking scores (CDOCKER interaction energy) of the best -fitted conformation of each of the docked molecules with the amino acids at the VEGFR-2 binding pocket were recorded.

2- ADMET studies

ADMET descriptors (absorption, distribution, metabolism, excretion, and toxicity) of the compounds were determined using Discovery studio 4.0. At first, the CHARMM force field was applied then the tested compounds were prepared and minimized according to the preparation of small molecule protocol. Then ADMET descriptors protocol was applied to carry out these studies.

3- Toxicity studies

The toxicity parameters of the synthesized compounds were calculated using Discovery studio 4.0. Sorafenib was used as a reference drug. At first, the CHARMM force field was applied then the compounds were prepared and minimized according to the preparation of small molecule protocol. Then different parameters were calculated from toxicity prediction (extensible) protocol.

1H NMR of compound 5

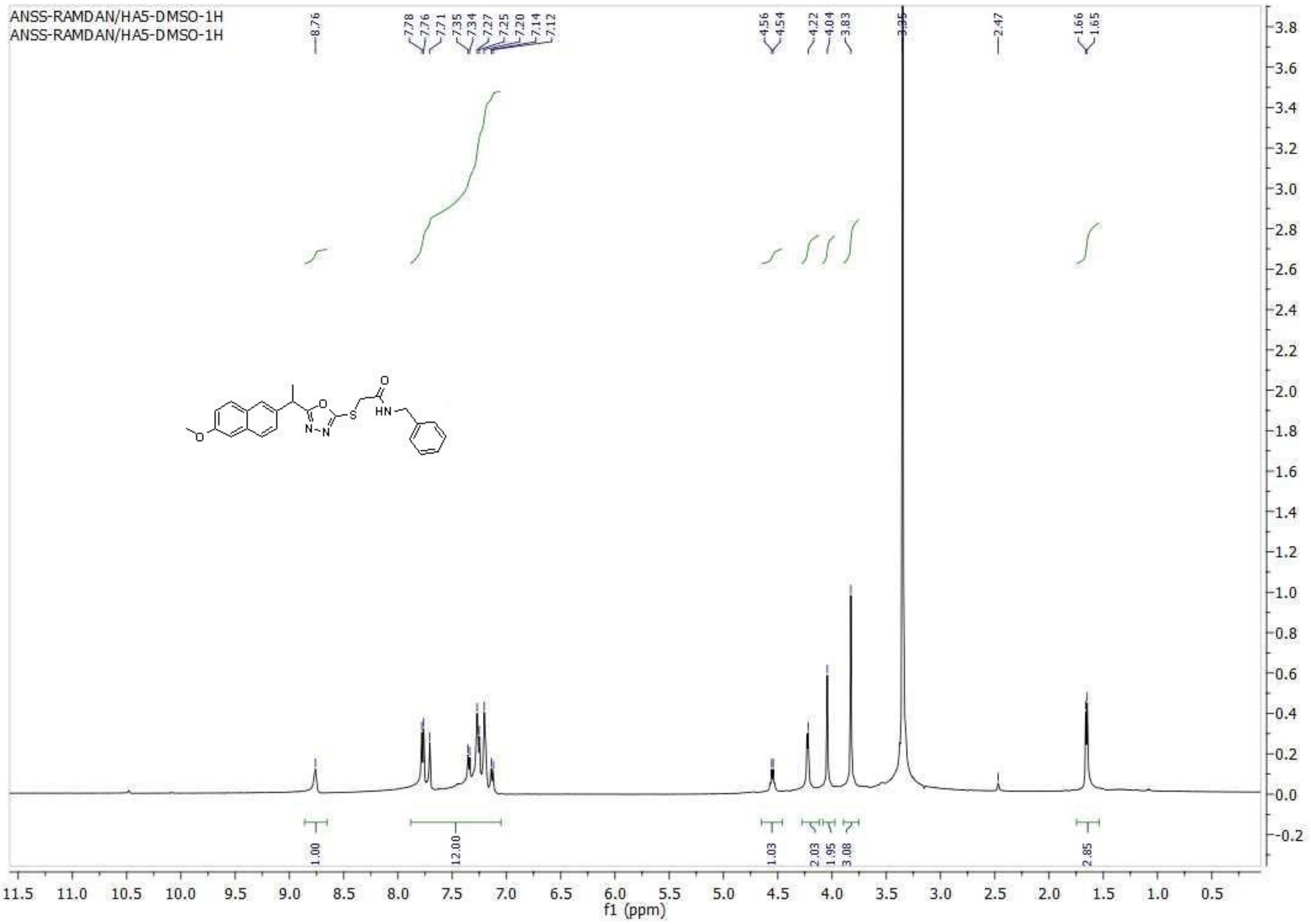

1H NMR of compound 5

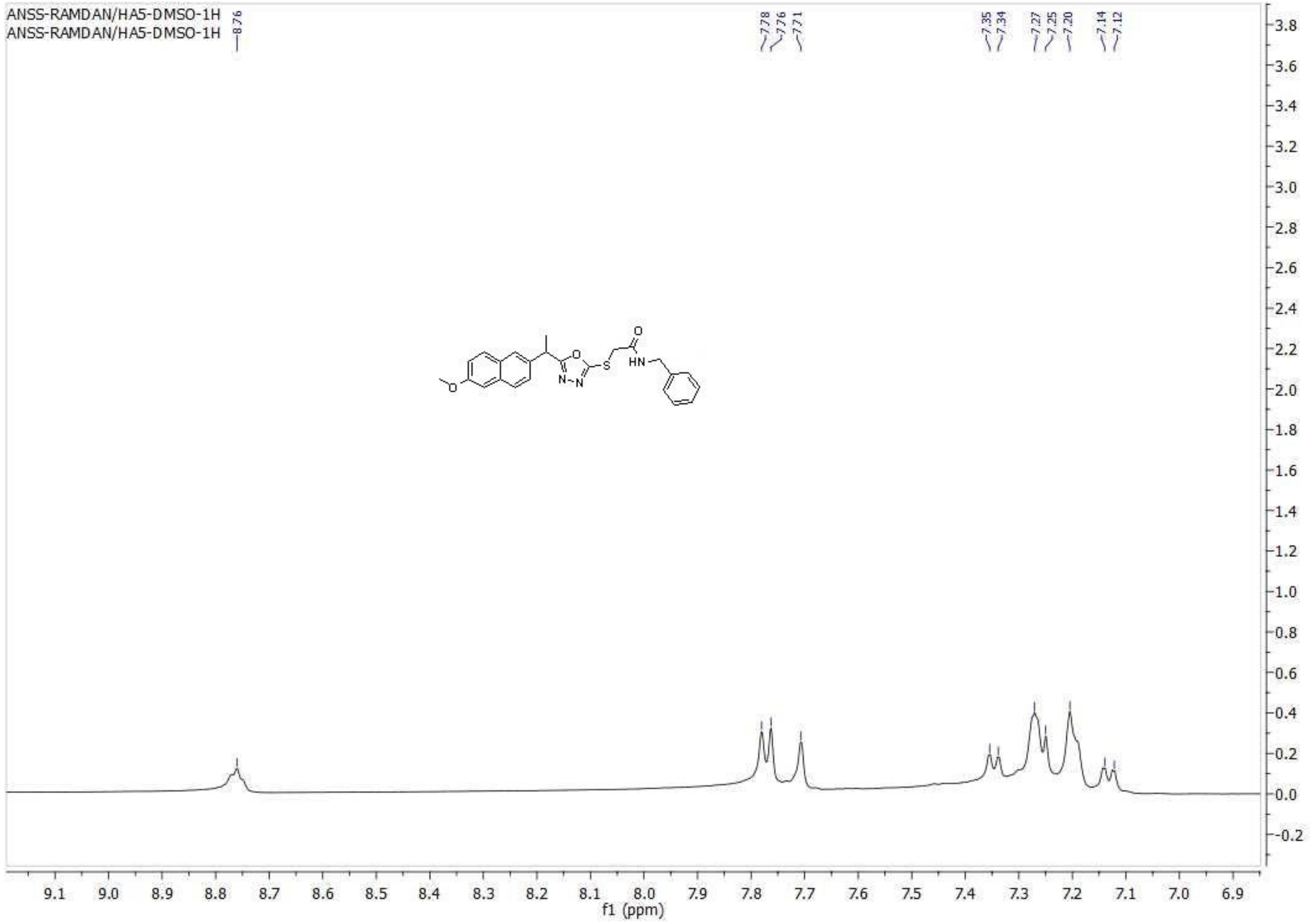

1H NMR of compound 5

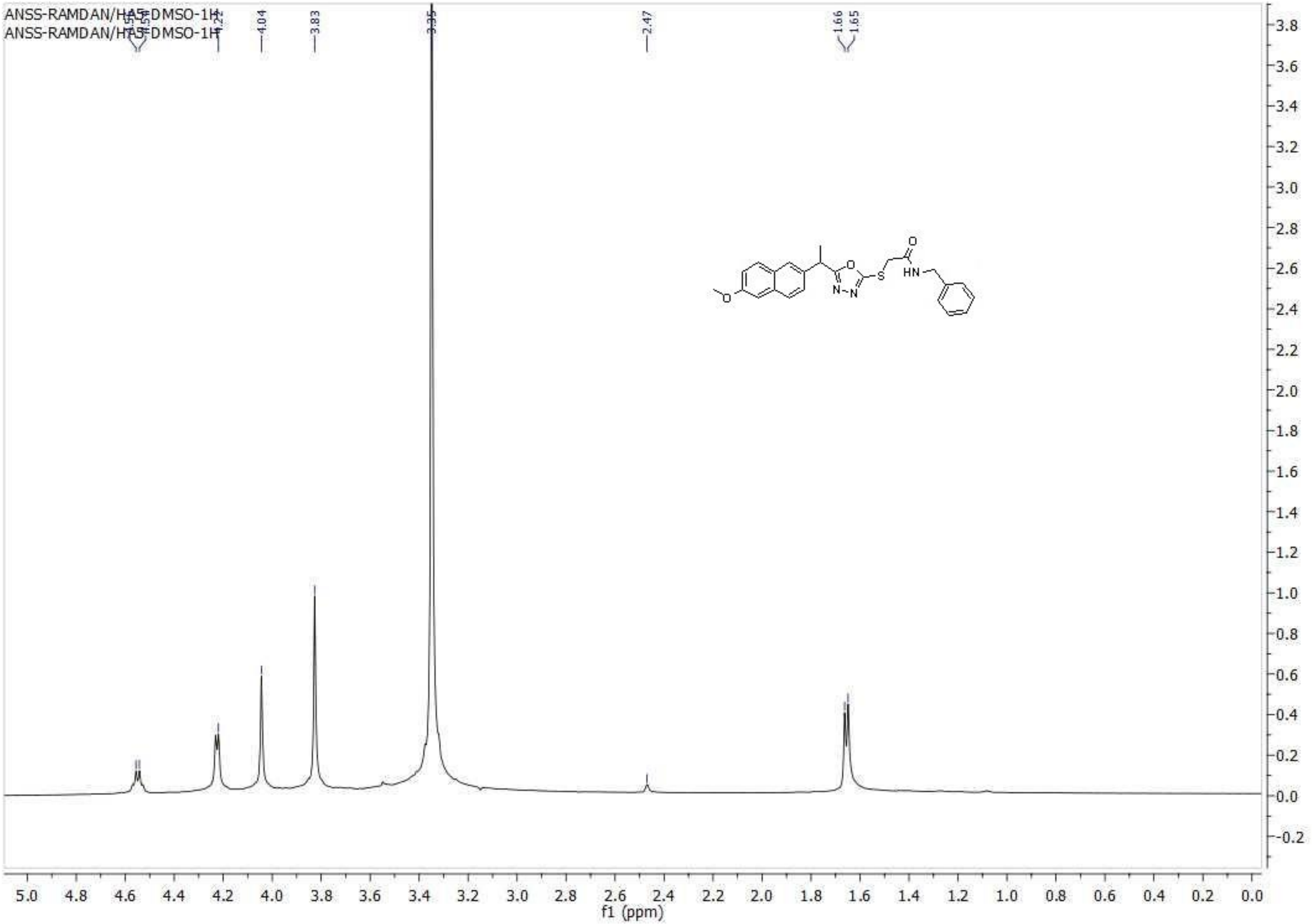

13C NMR of compound 5

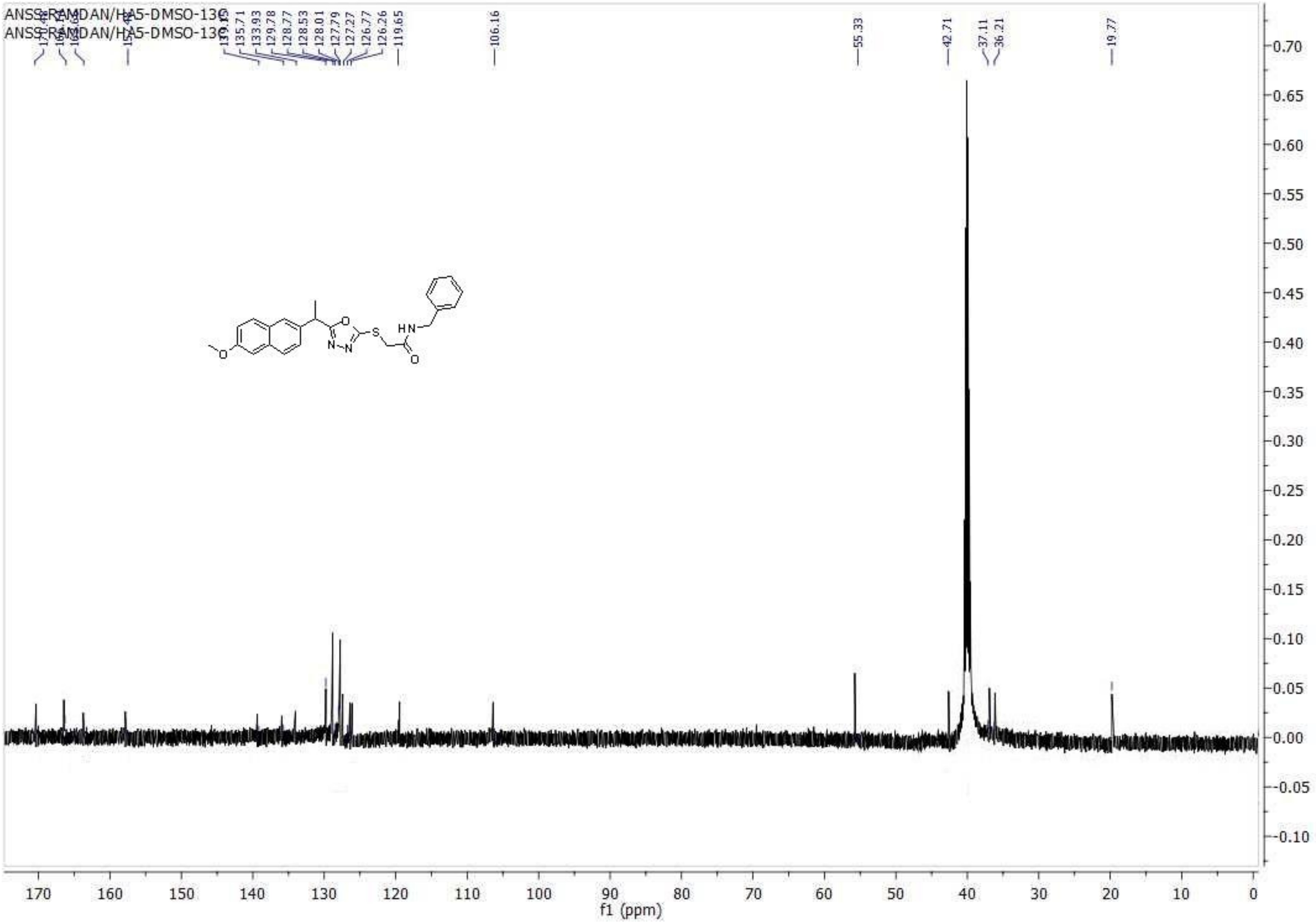

1H NMR of compound 6

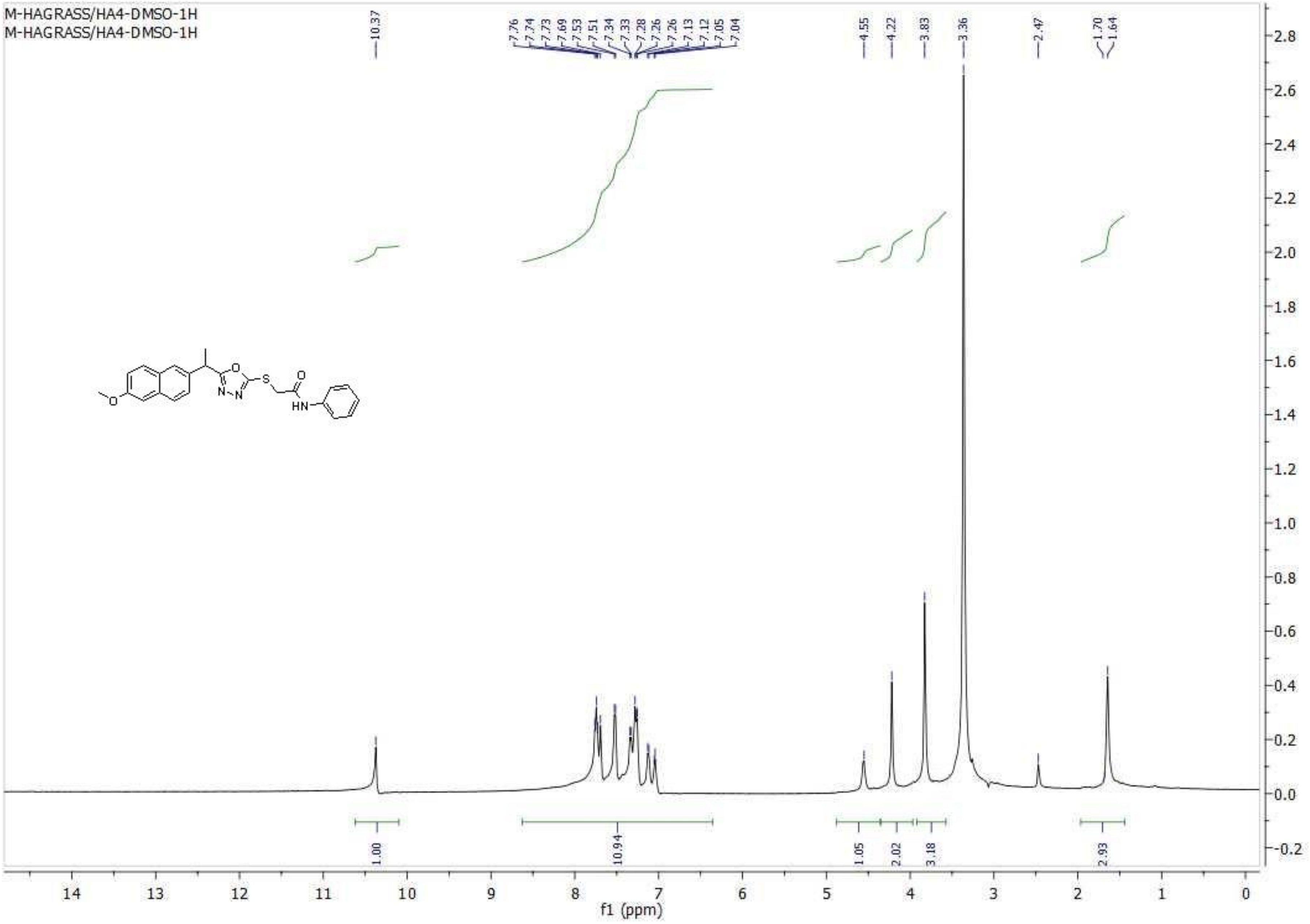

1H NMR of compound 6

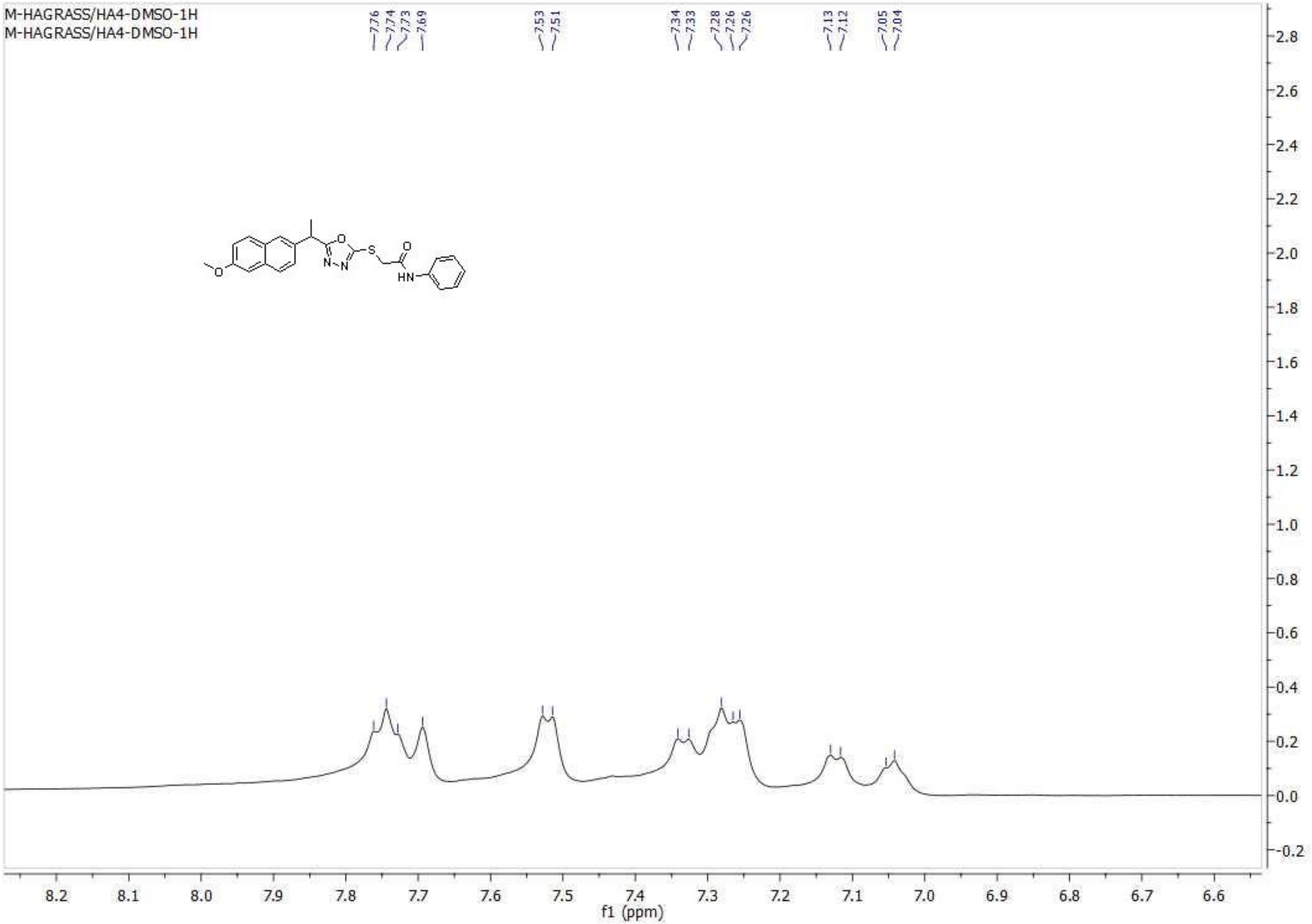

1H NMR of compound 6

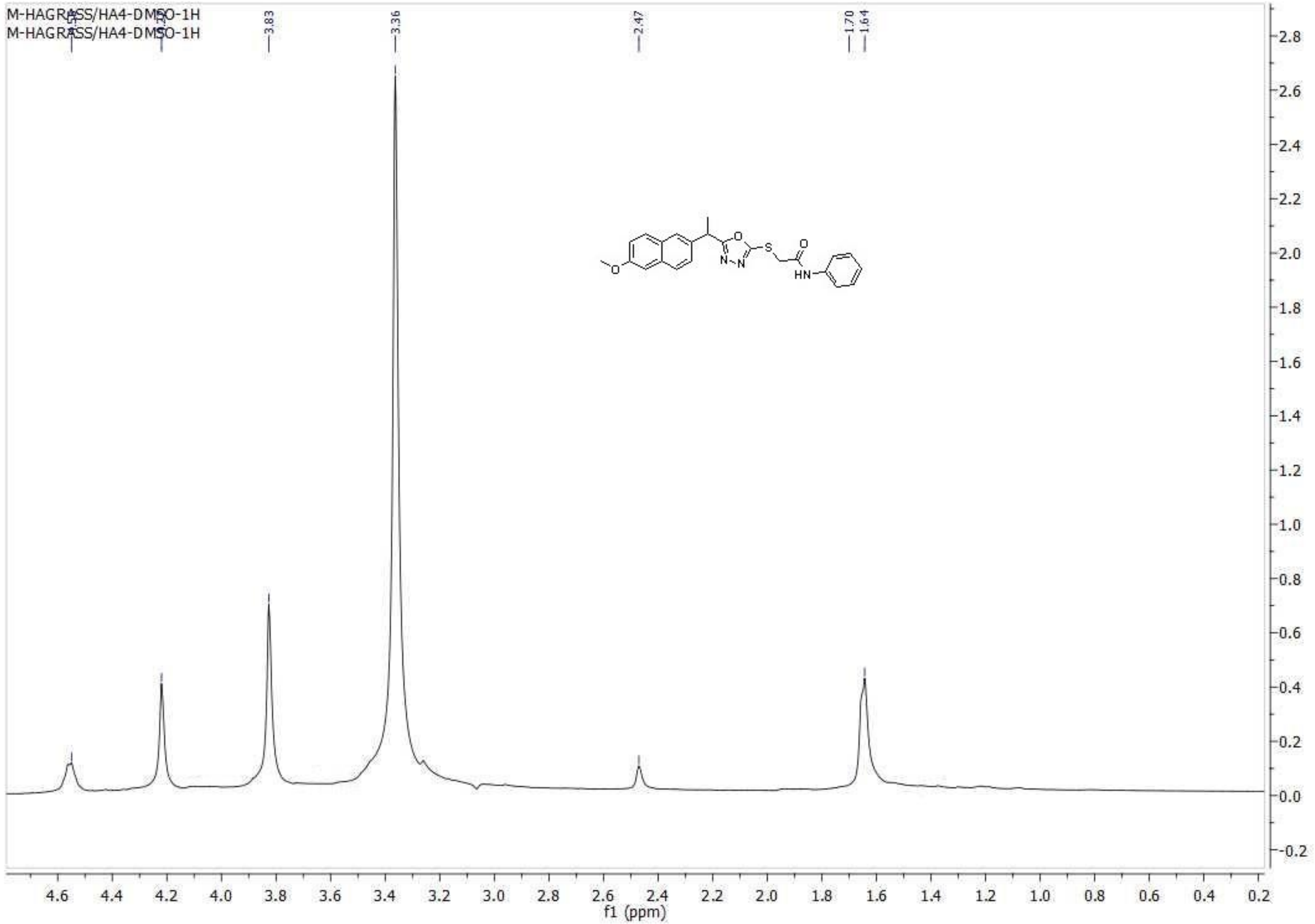

13C NMR of compound 6

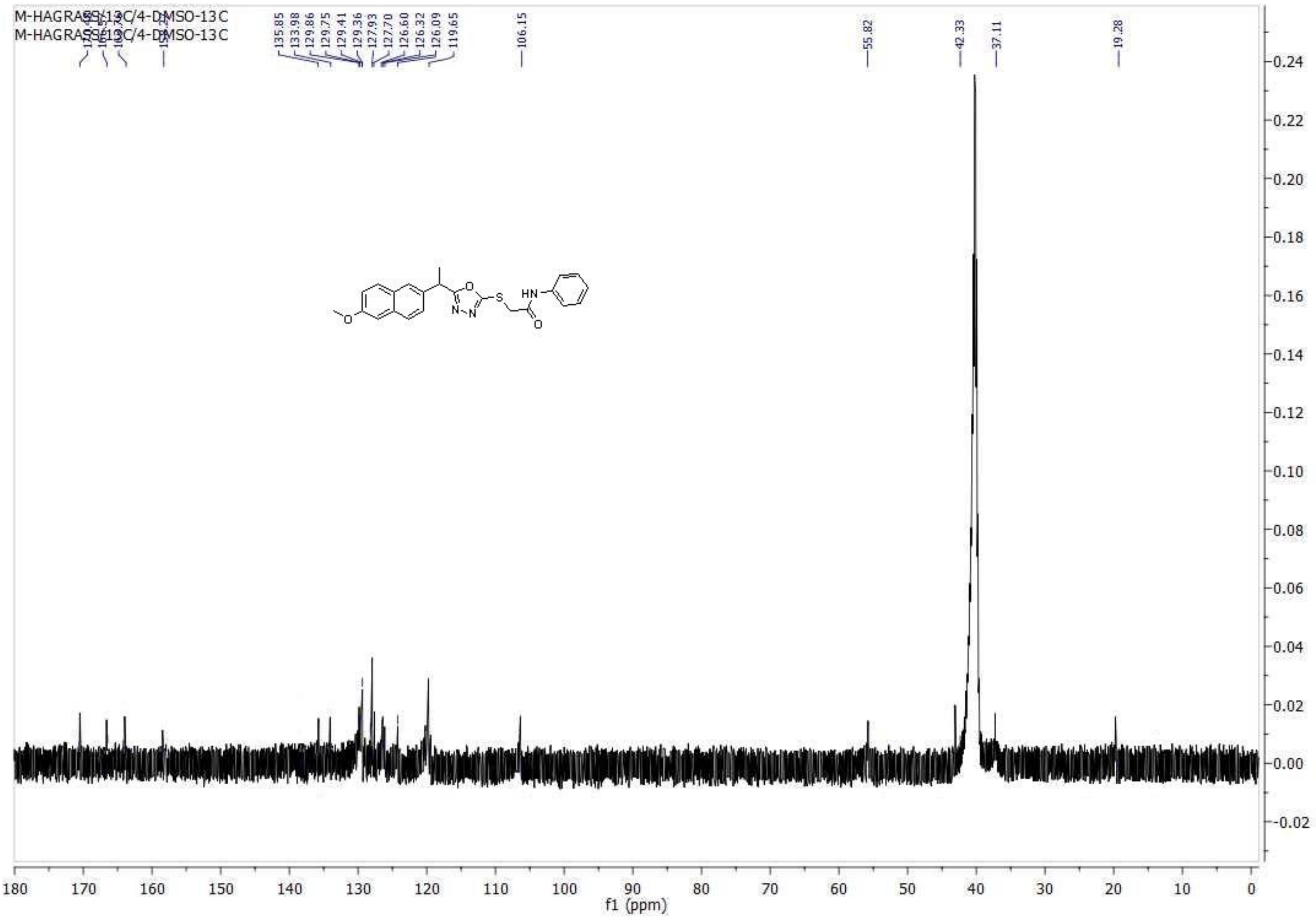

1H NMR of compound 7

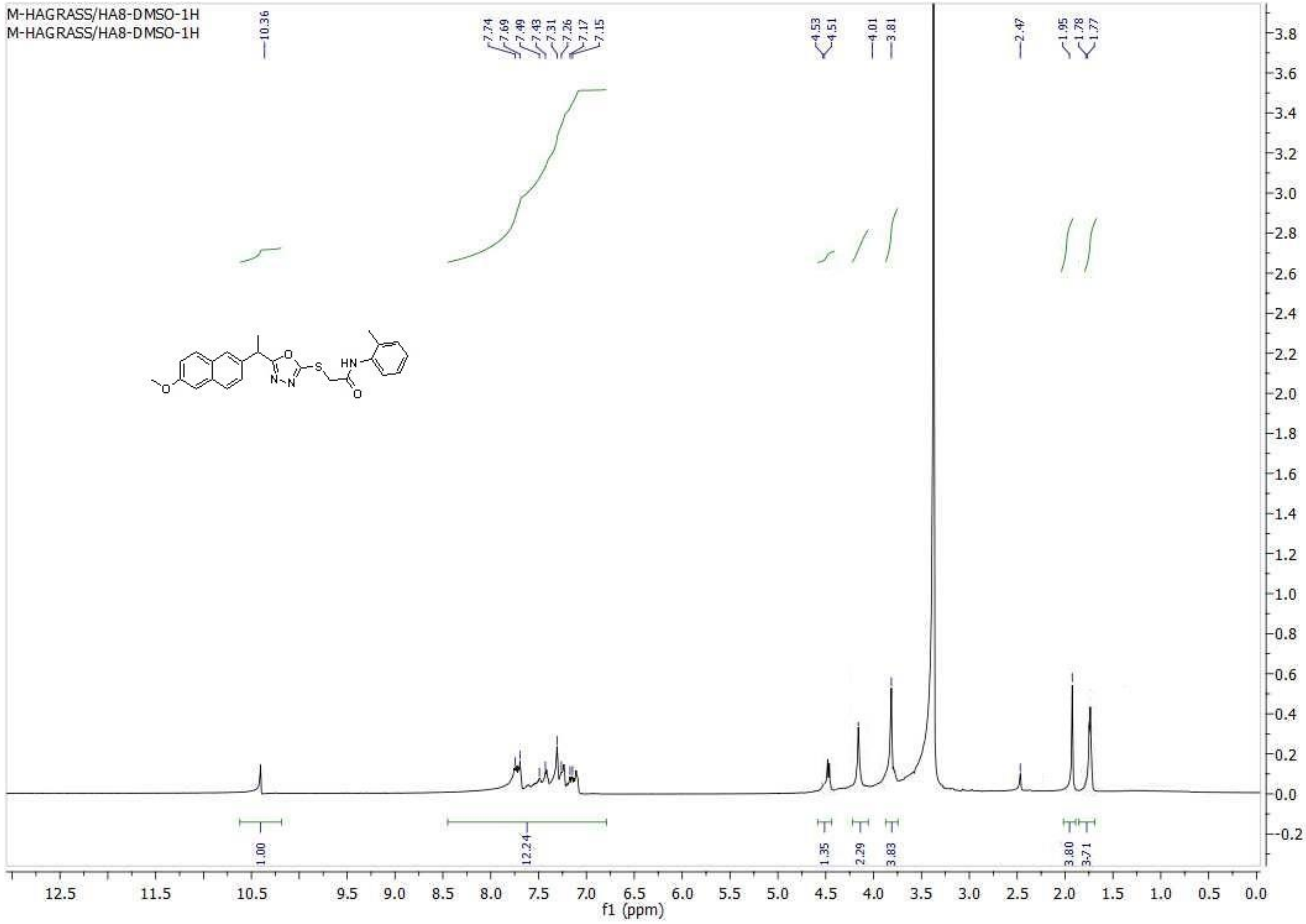

13C NMR of compound 7

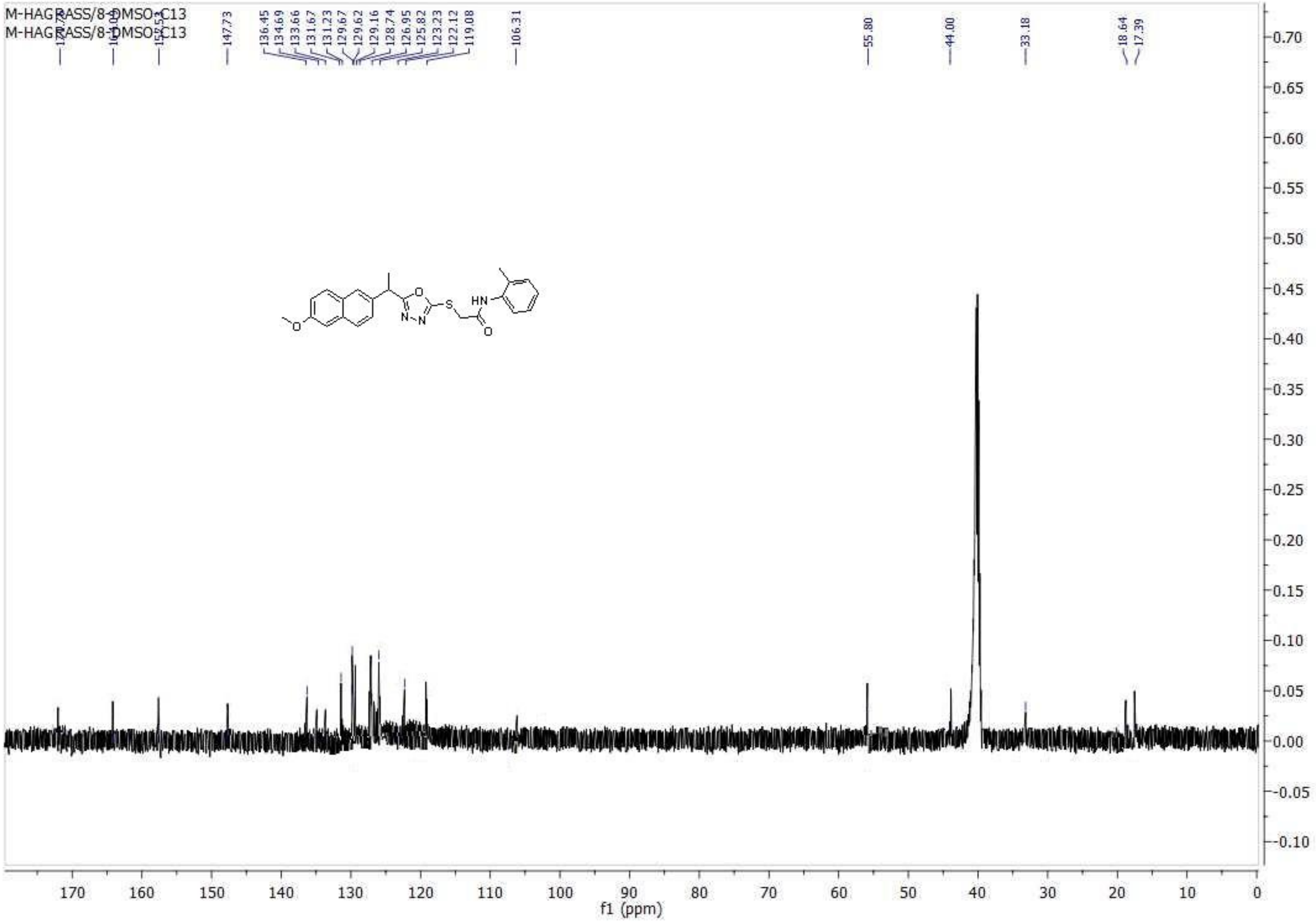

1H NMR of compound 8

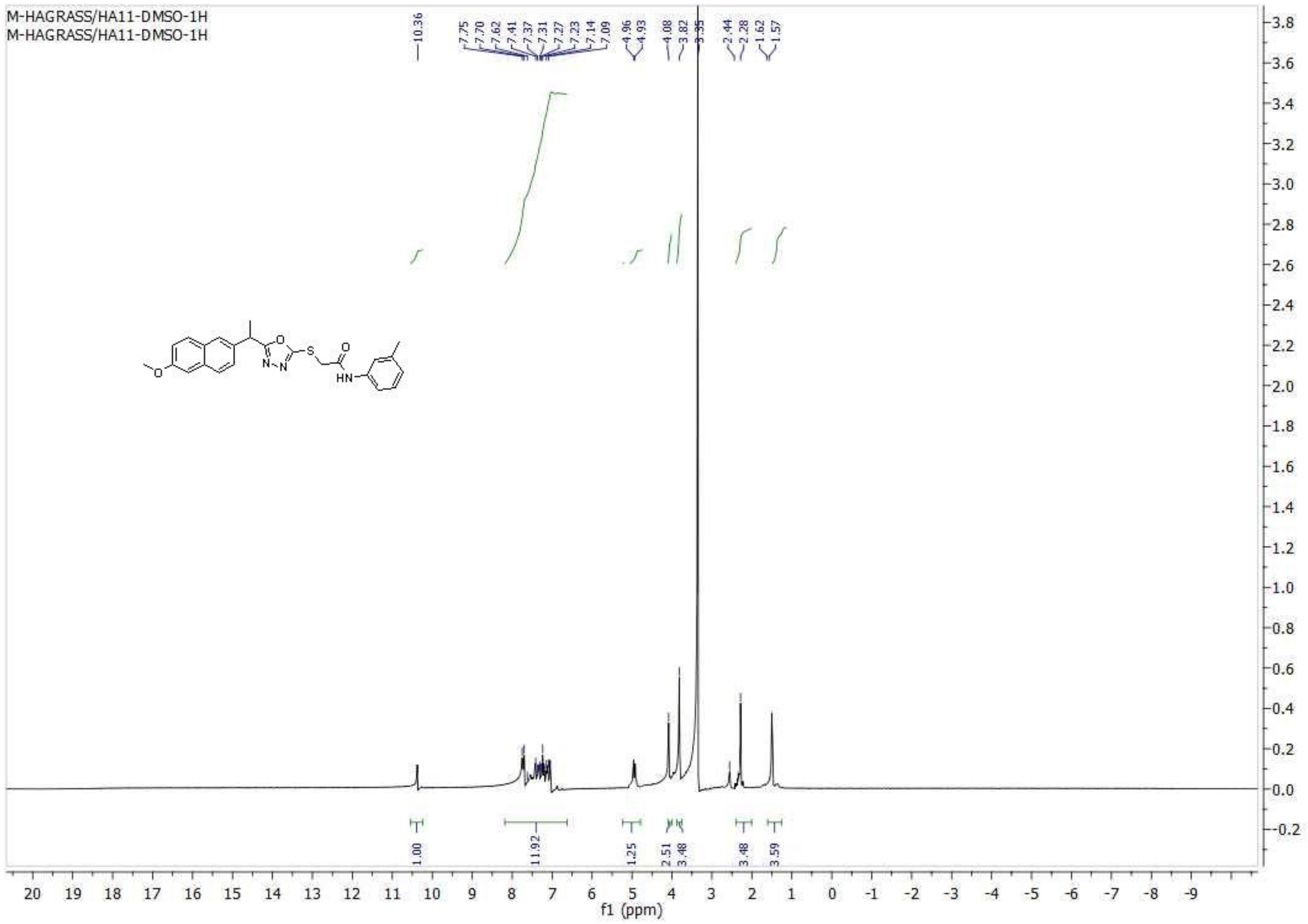

13C NMR of compound 8

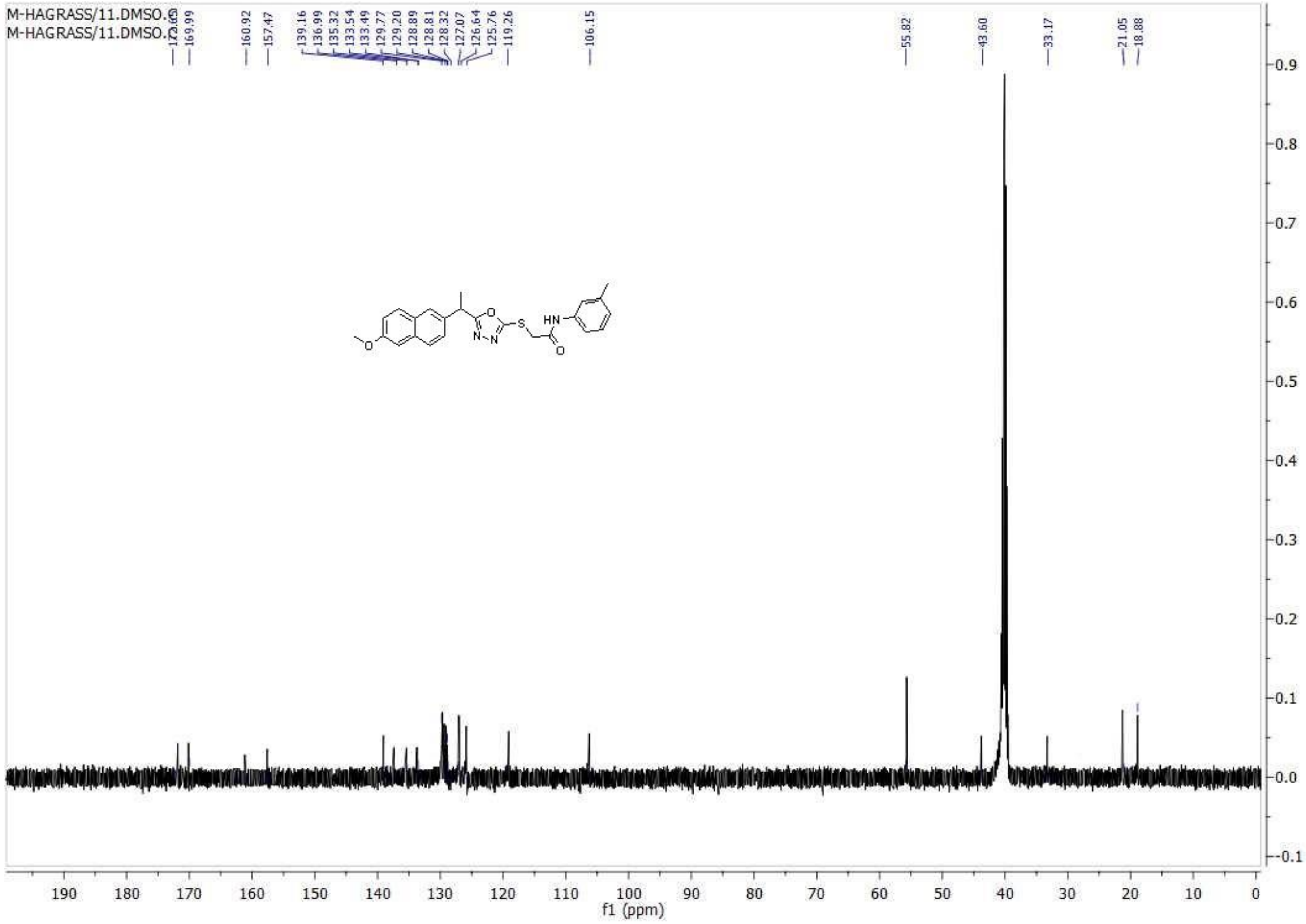

1H NMR of compound 9

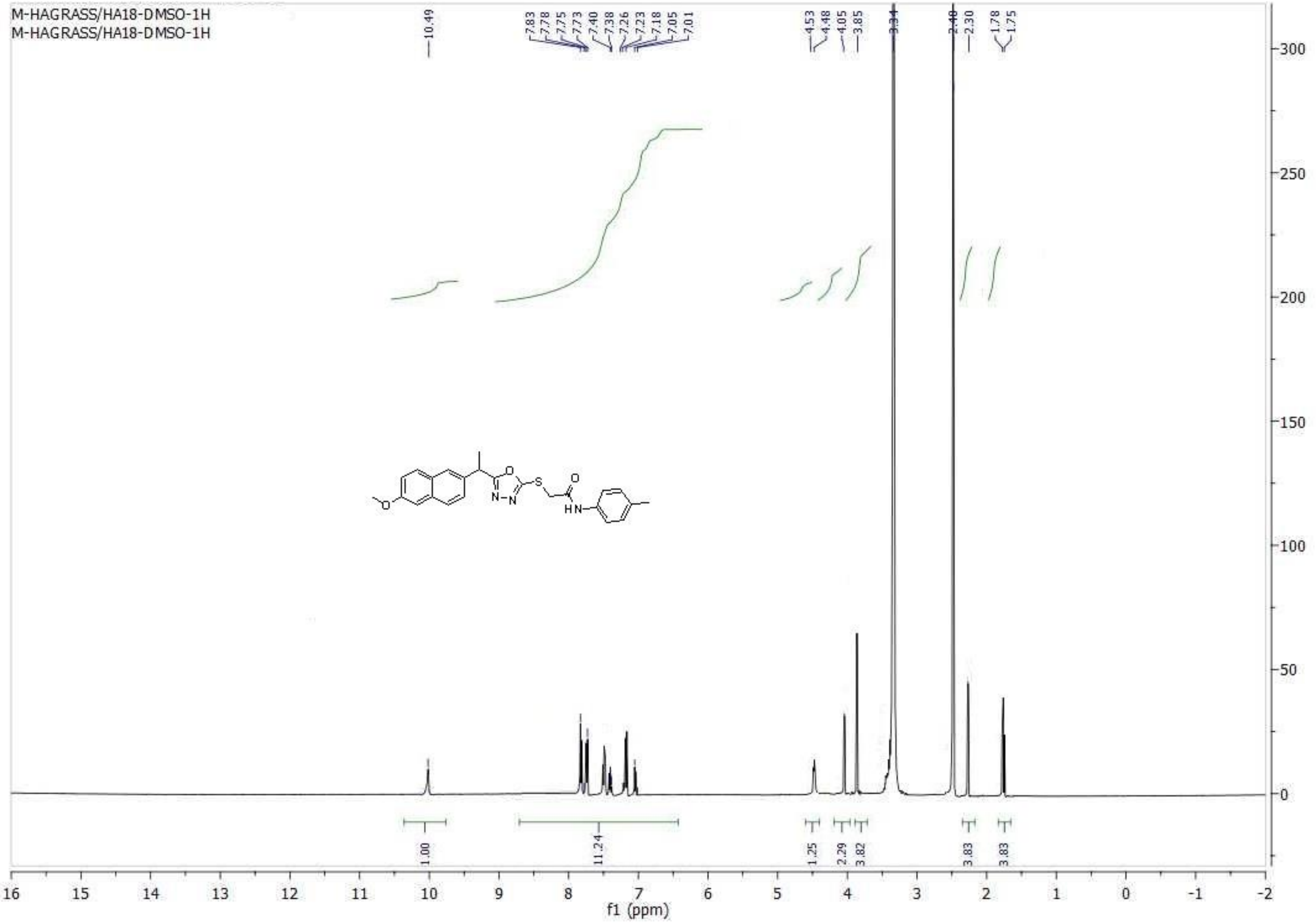

13C NMR of compound 9

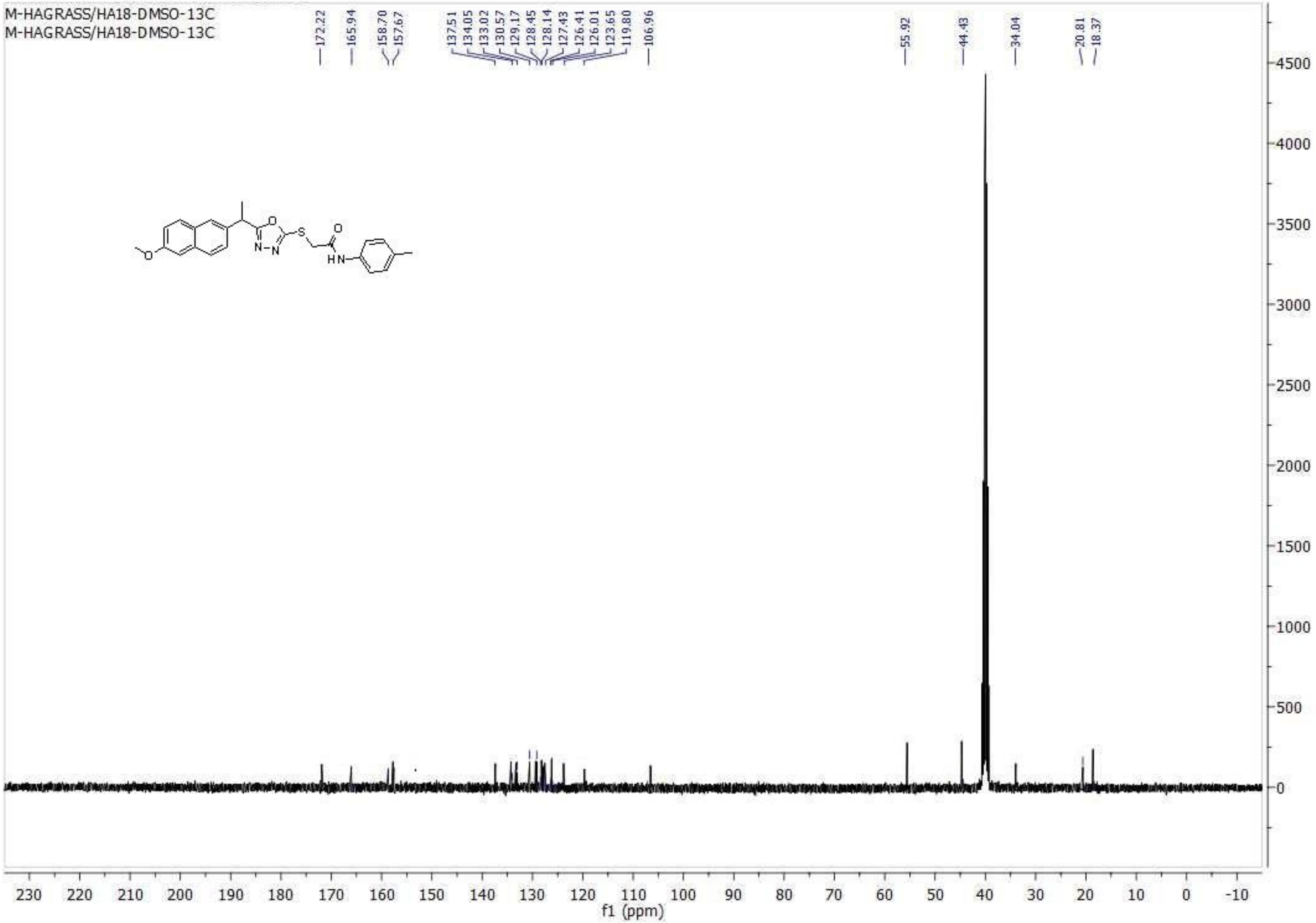

1H NMR of compound 10

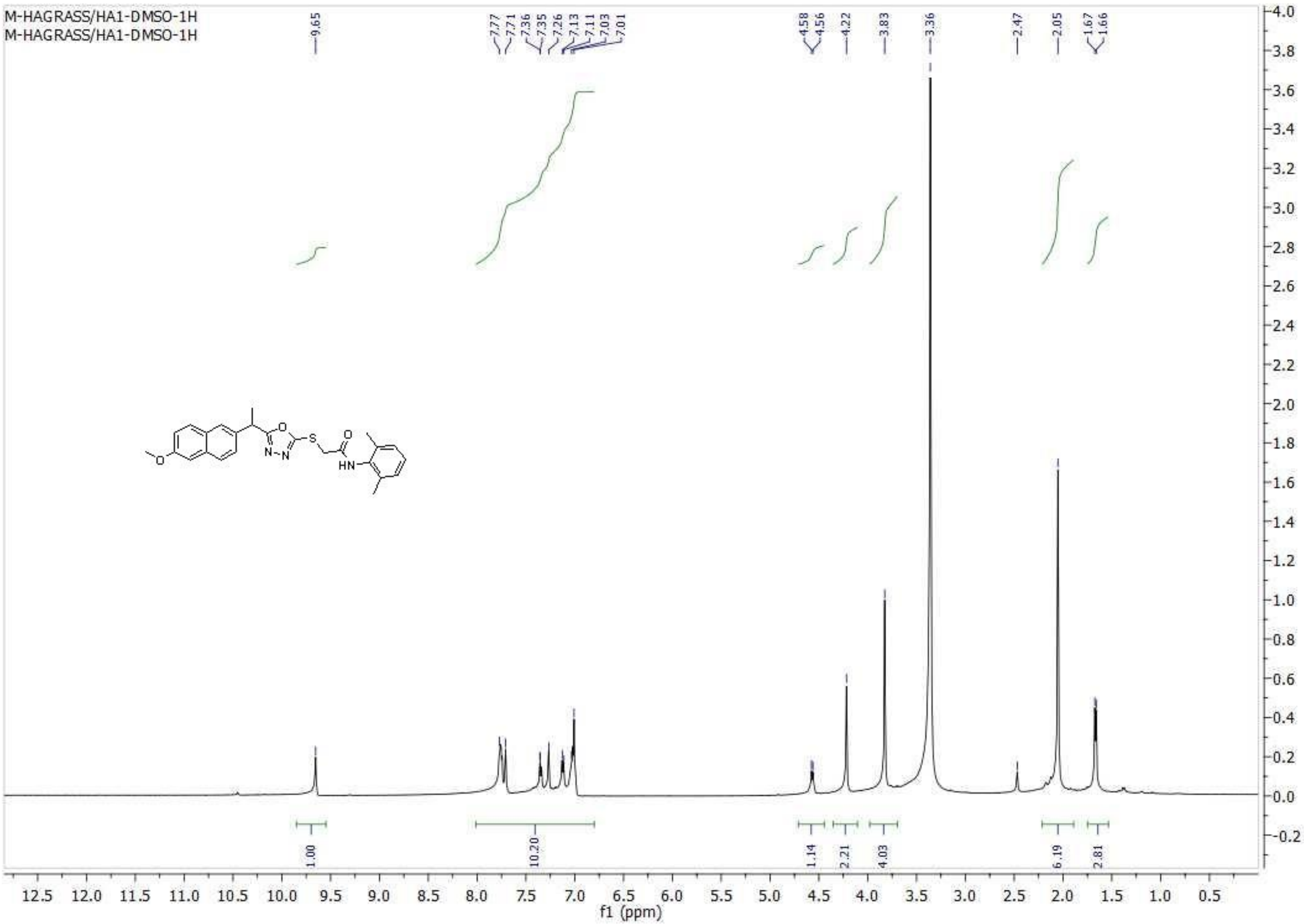

13C NMR of compound 10

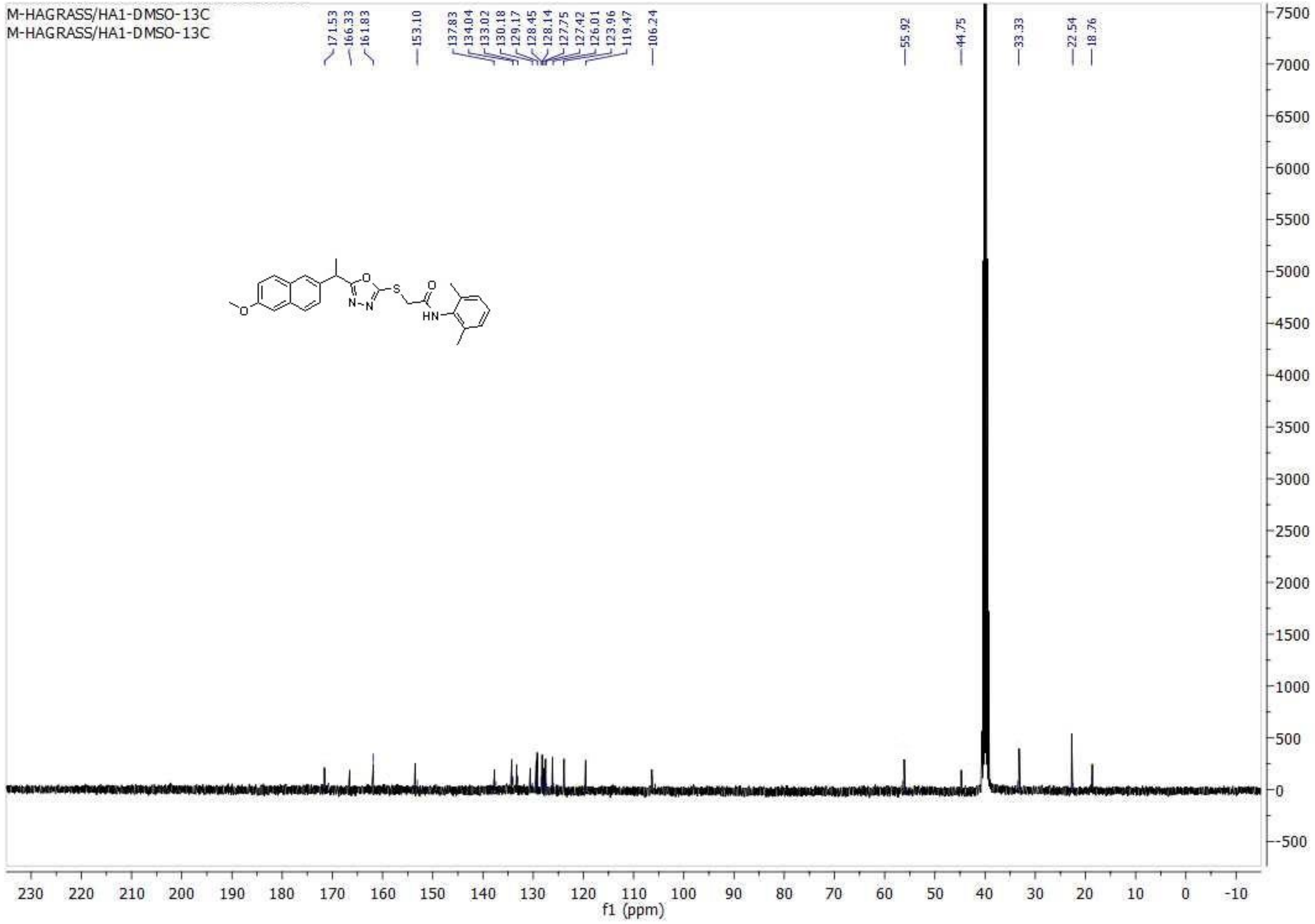

1H NMR of compound 11

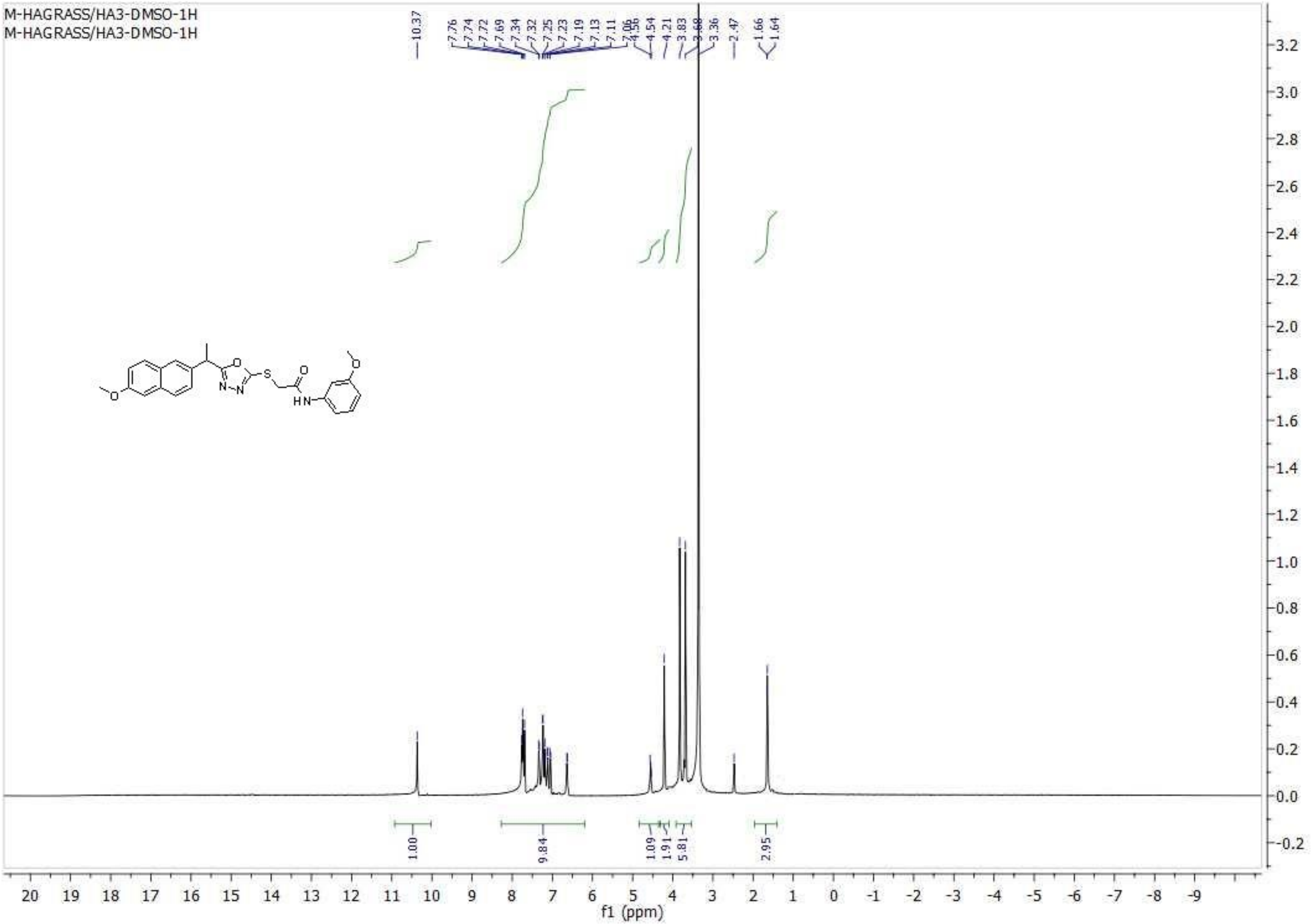

13C NMR of compound 11

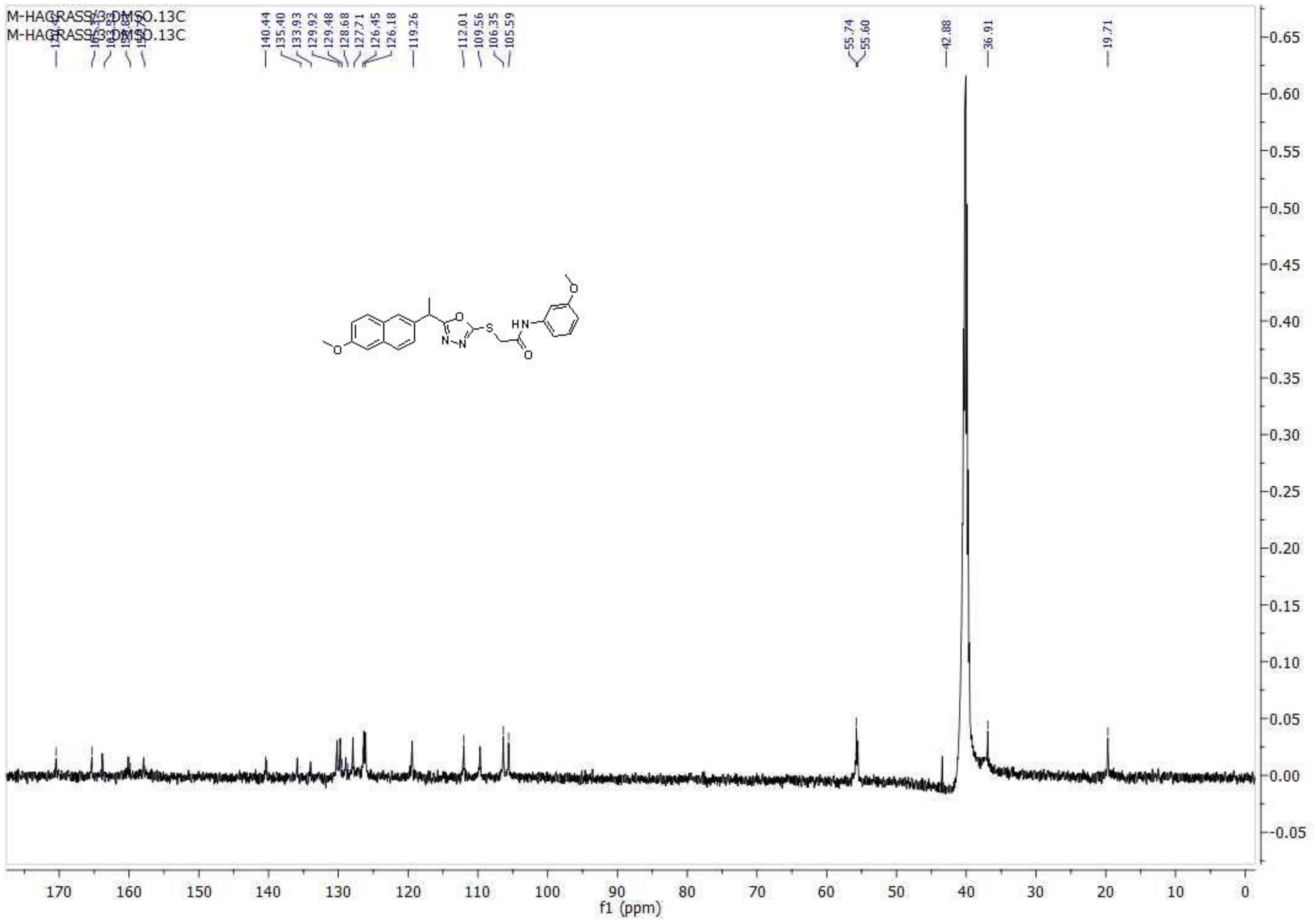

1H NMR of compound 12

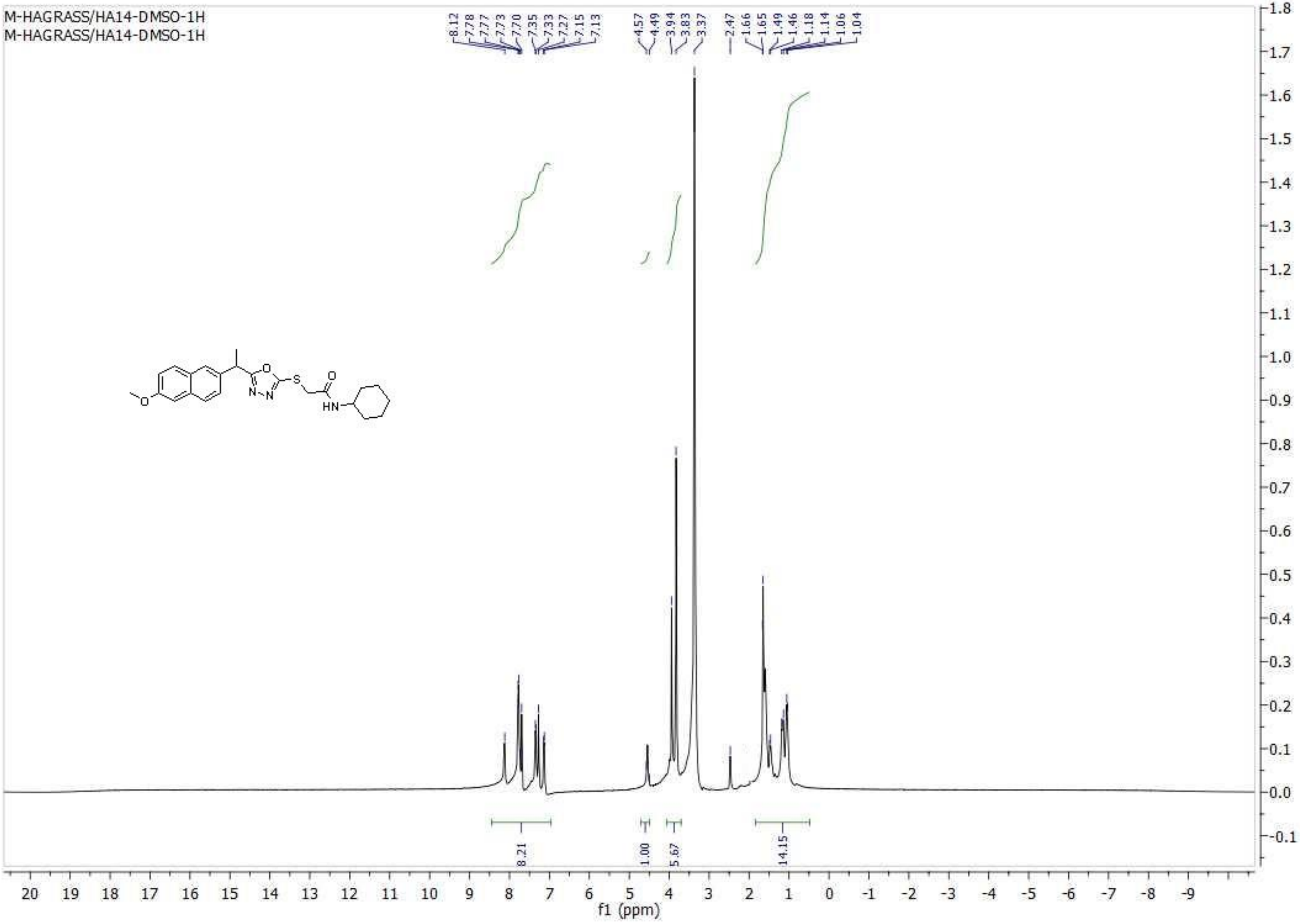

13C NMR of compound 12

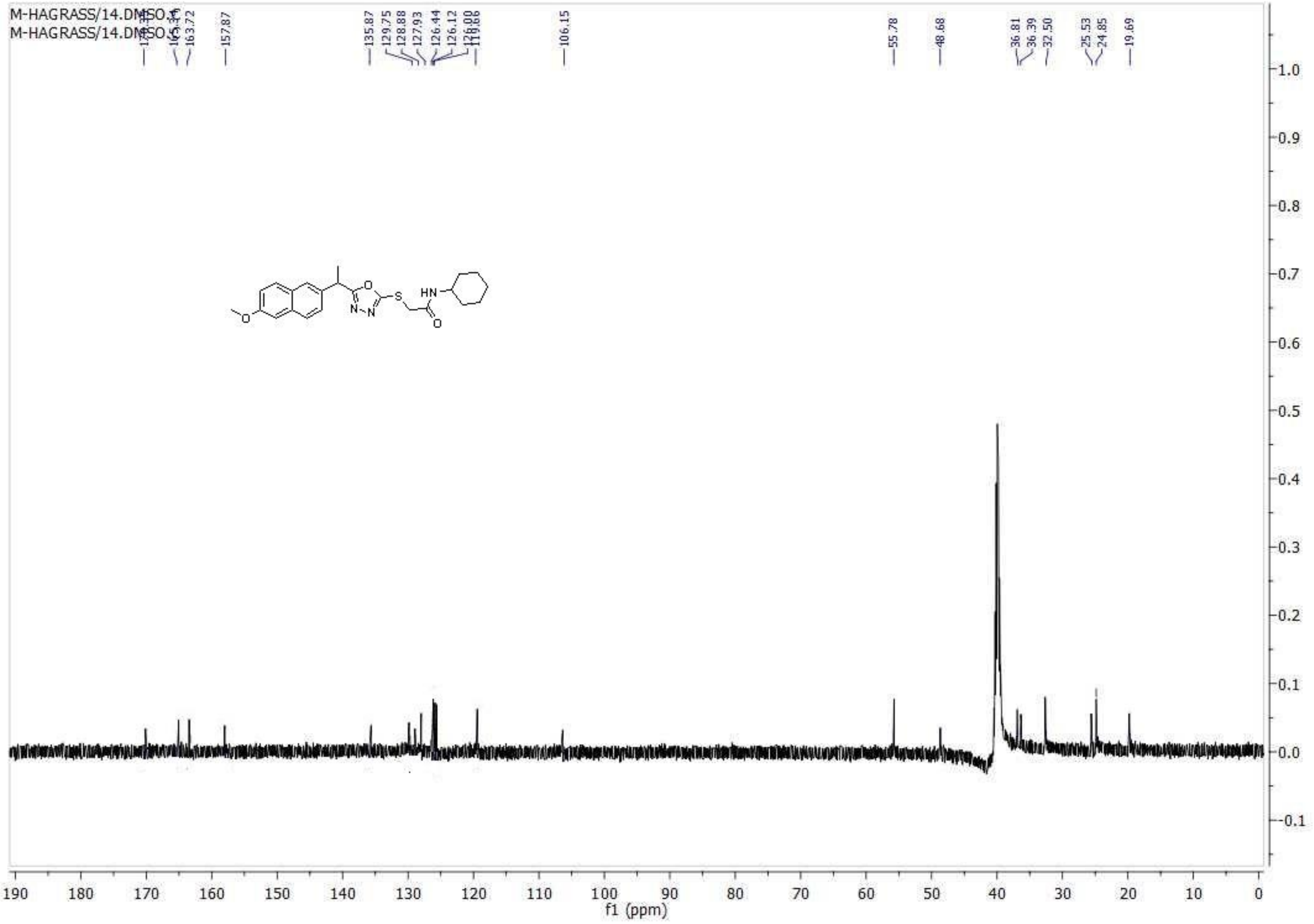

1H NMR of compound 13

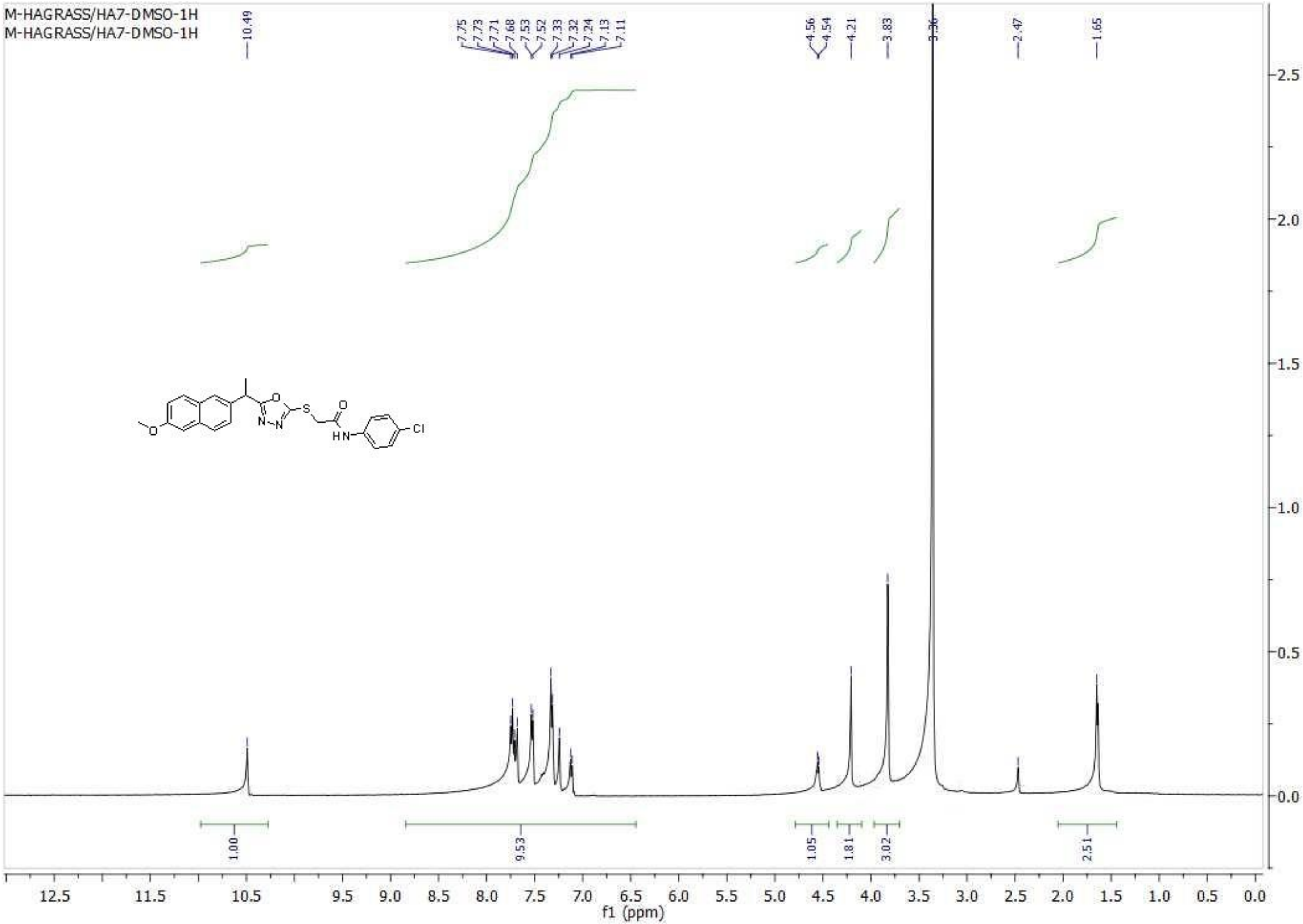

13C NMR of compound 13

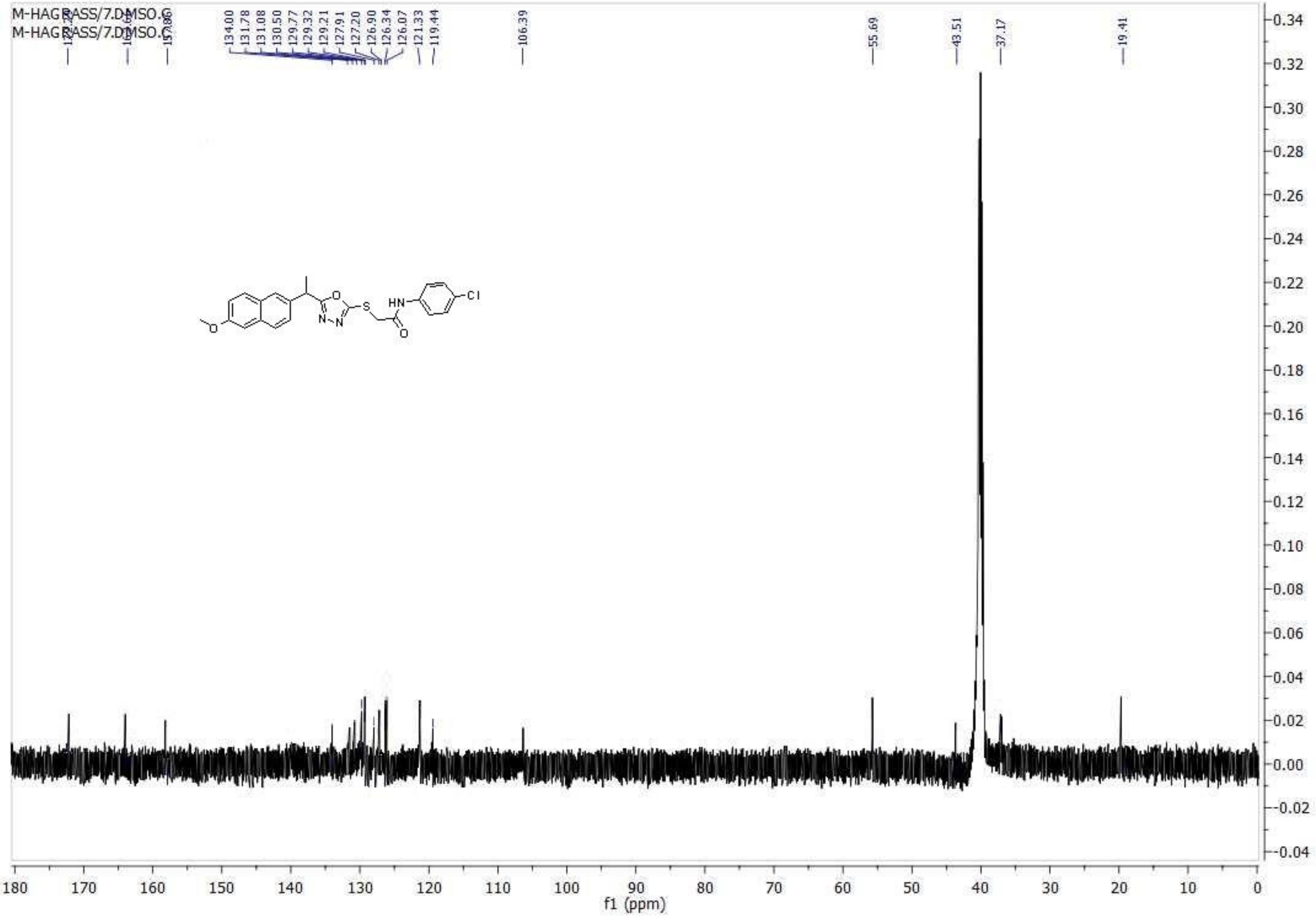

1H NMR of compound 17

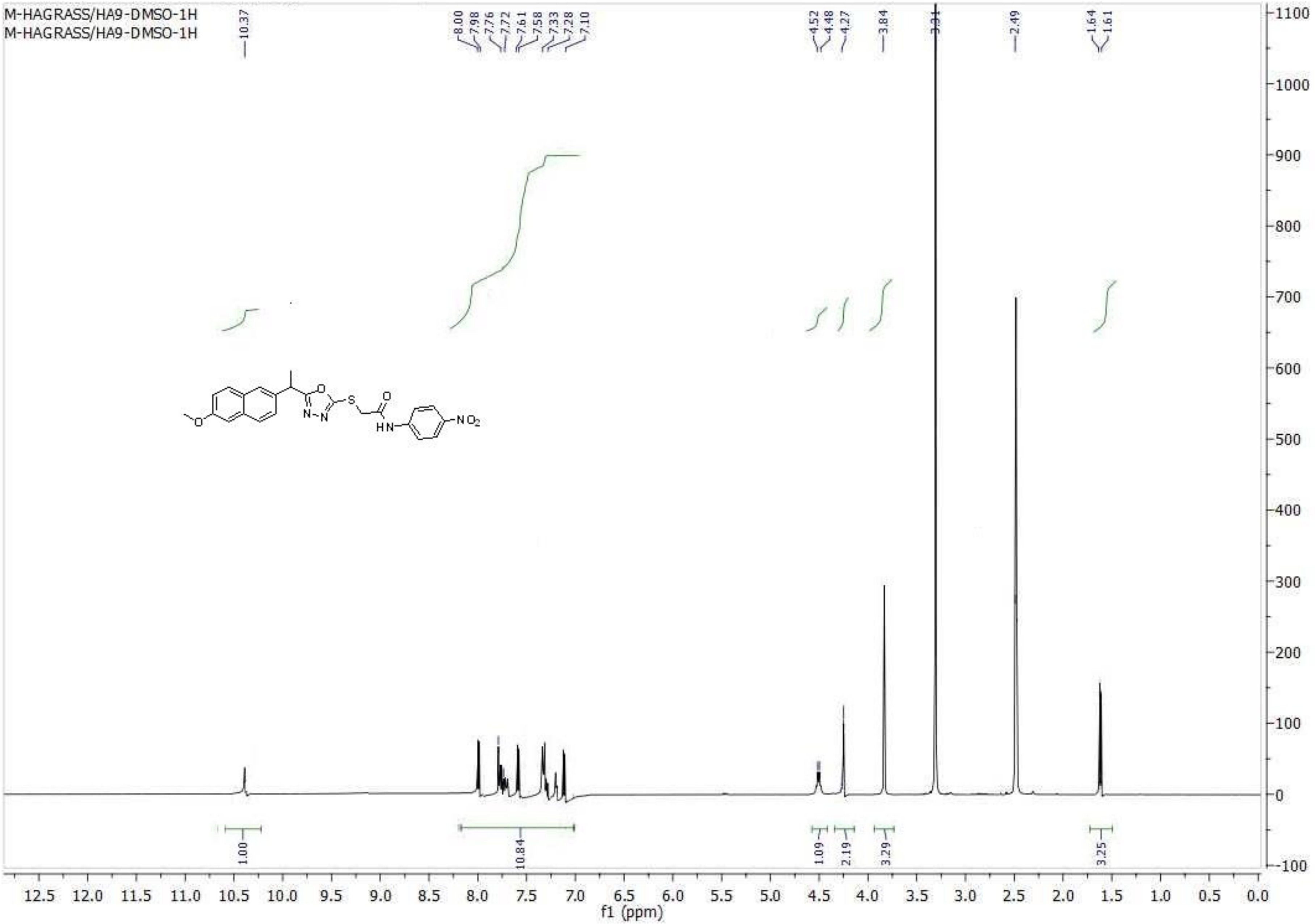

13C NMR of compound 17

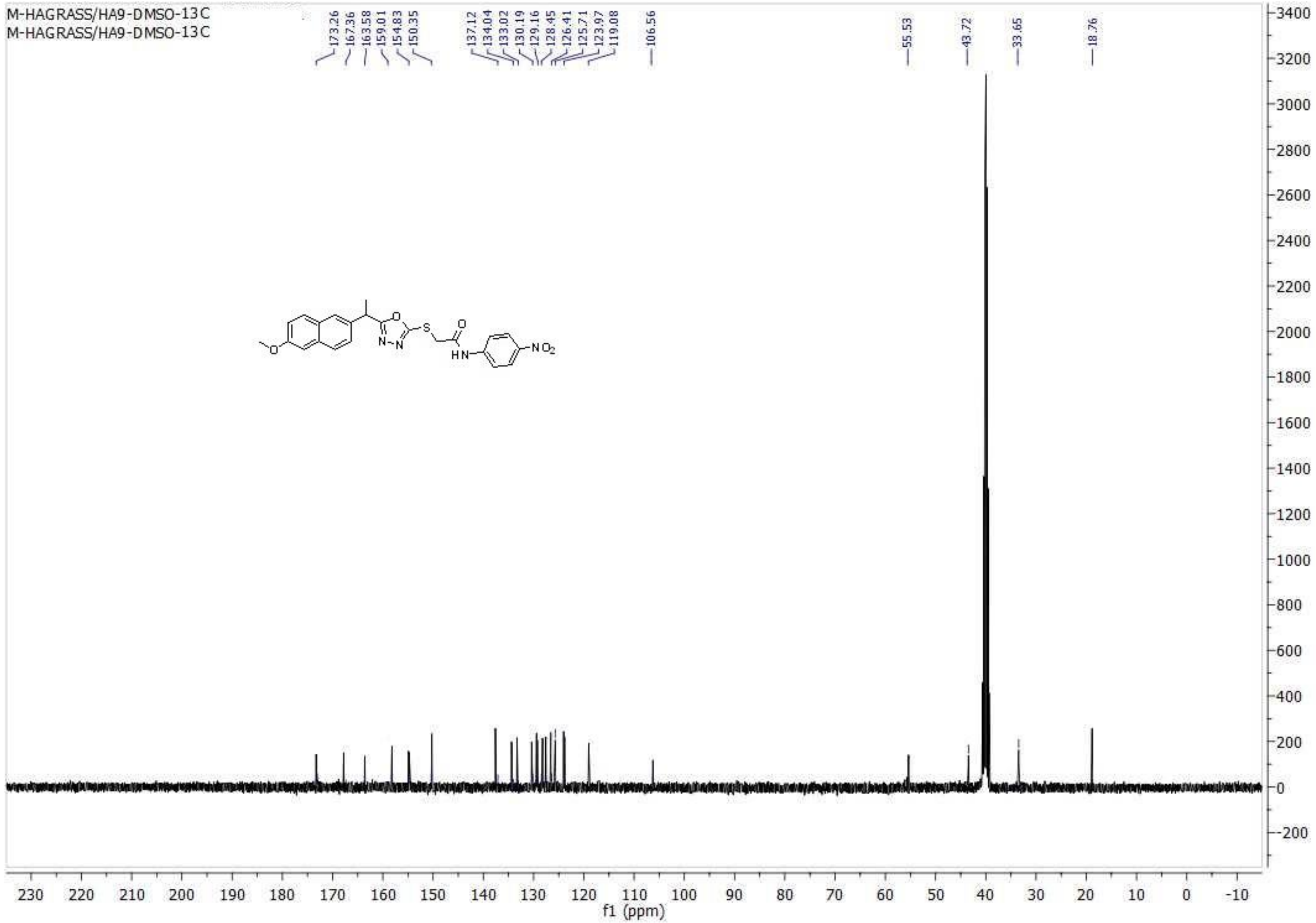

1H NMR of compound 18

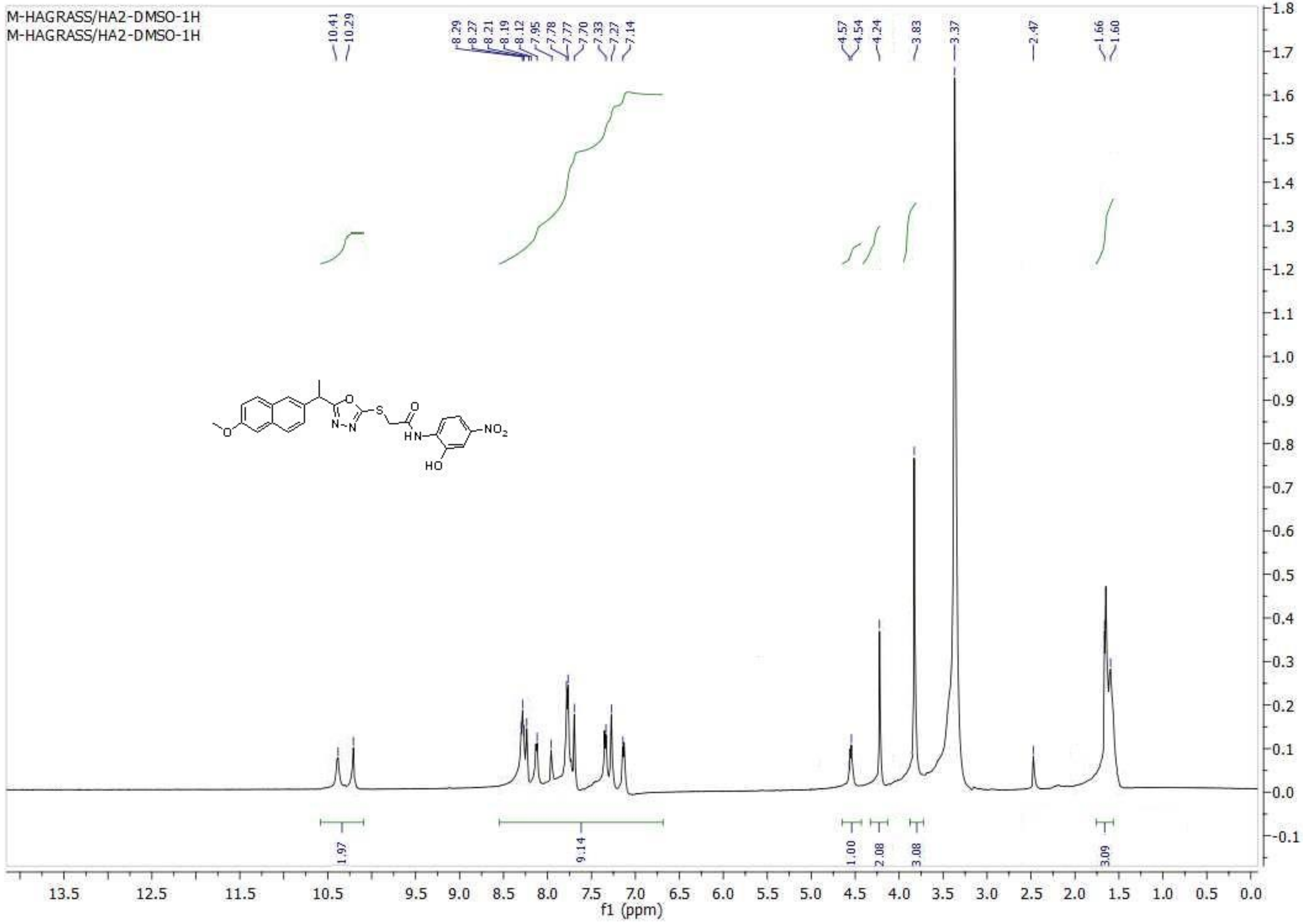

13C NMR of compound 18

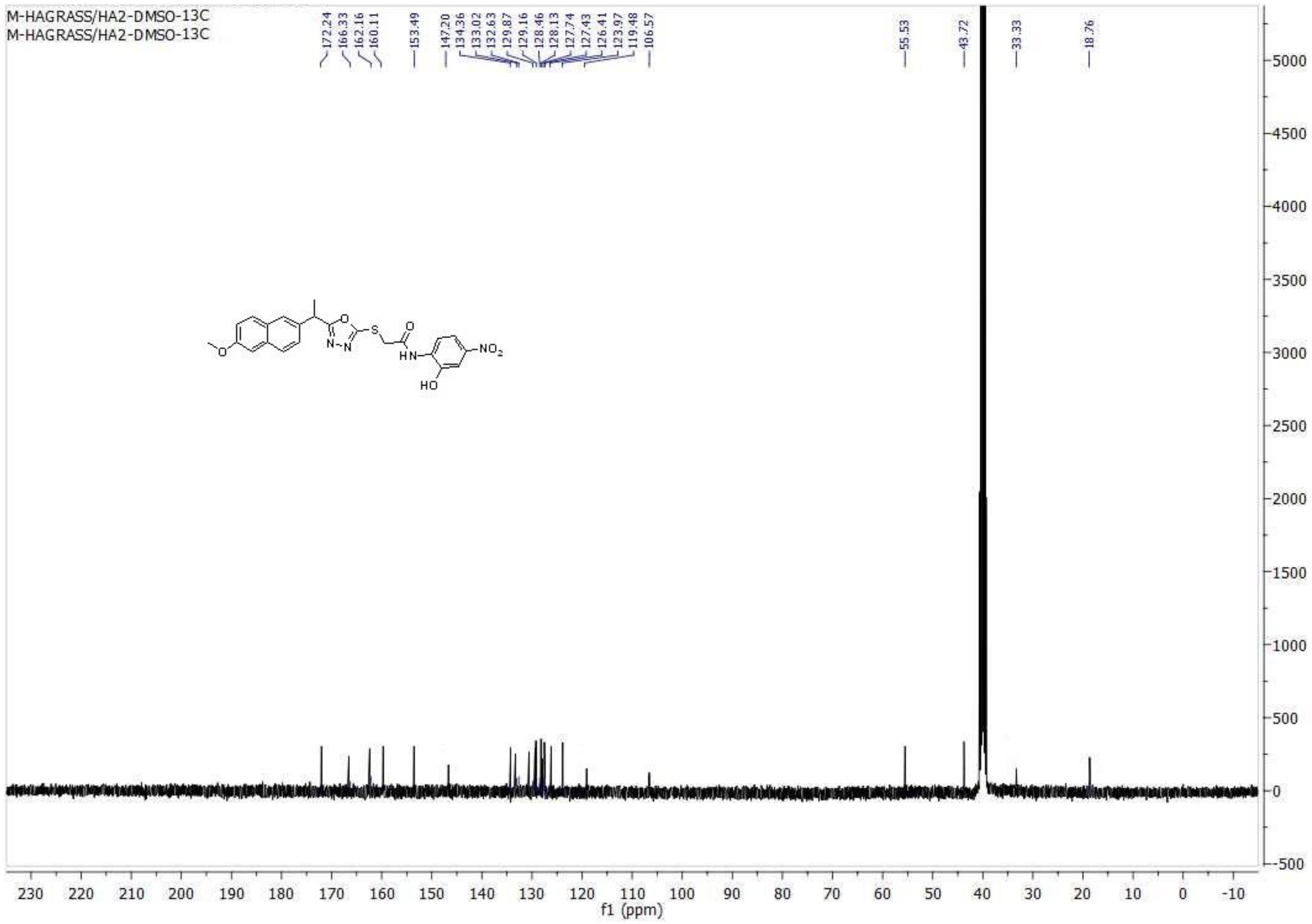

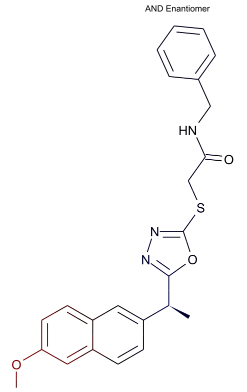

$C_{24}H_{23}N_3O_3S$
Molecular Weight: 433.52272
ALogP: 4.245
Rotatable Bonds: 8
Acceptors: 5
Donors: 1

Structural Similar Compounds

Name	Acemetacin	Nicardipine	Suxibuzone
Structure	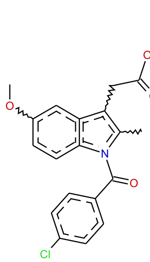	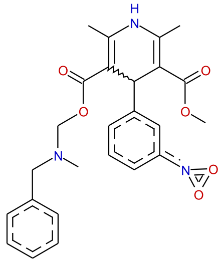	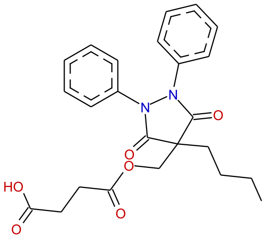
Actual Endpoint	Non-Toxic	Non-Toxic	Toxic
Predicted Endpoint	Non-Toxic	Non-Toxic	Toxic
Distance	0.520	0.599	0.643
Reference	Oyo Yakuri 22(6):777-786; 1981	Kiso to Rinsho 13:1149-1159; 1979	Oyo Yakuri 20:377-386; 1980

Model Prediction

Prediction: Non-Toxic
Probability: 0.513
Enrichment: 0.974
Bayesian Score: -0.947
Mahalanobis Distance: 10.646
Mahalanobis Distance p-value: 0.00339

Prediction: Positive if the Bayesian score is above the estimated best cutoff value from minimizing the false positive and false negative rate.
Probability: The esimated probability that the sample is in the positive category. This assumes that the Bayesian score follows a normal distribution and is different from the prediction using a cutoff.
Enrichment: An estimate of enrichment, that is, the increased likelihood (versus random) of this sample being in the category.
Bayesian Score: The standard Laplacian-modified Bayesian score.
Mahalanobis Distance: The Mahalanobis distance (MD) is the distance to the center of the training data. The larger the MD, the less trustworthy the prediction.
Mahalanobis Distance p-value: The p-value gives the fraction of training data with an MD greater than or equal to the one for the given sample, assuming normally distributed data. The smaller the p-value, the less trustworthy the prediciton. For highly non-normal X properties (e.g., fingerprints), the MD p-value is wildly inaccurate.

Model Applicability

Unknown features are fingerprint features in the query molecule, but not found in the training set.

1. All properties and OPS components are within expected ranges.

Feature Contribution

Top features for positive contribution

Fingerprint	Bit/Smiles	Feature Structure	Score	Toxic in training set
SCFP_6	1237755852	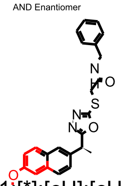 [*]:[c]1:[*]:[cH]:[cH]:[c](OC):[cH]:1	0.453	8 out of 9

SCFP_6	591469355	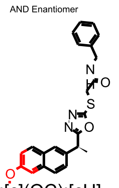 [*]:[cH]:[c](OC):[cH]:[*]	0.411	10 out of 12
SCFP_6	-1889730273	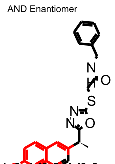 [*]O[c]1:[cH]:[cH]:[c]2:[cH]:[c]([*]):[*]:[cH]:[c]:2:[cH]:1	0.271	1 out of 1
**Top Features for negative contribution**				
**Fingerprint**	**Bit/Smiles**	**Feature Structure**	**Score**	**Toxic in training set**
SCFP_6	149212520	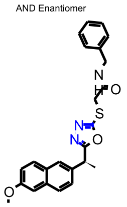 [*][c]1:[*]:[*]:n:n:1	-0.448	5 out of 16
SCFP_6	1424234162	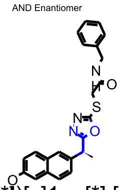 [*]C([*])[c]1:o:[*]:[*]:n:1	-0.422	0 out of 1
SCFP_6	-109786778	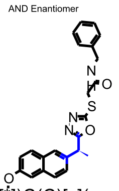 [*]:[c](:[*])C(C)[c](:[*]):[*]	-0.422	0 out of 1

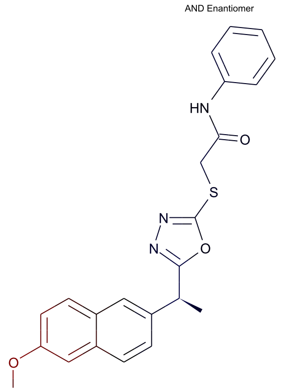

$C_{23}H_{21}N_3O_3S$
Molecular Weight: 419.49614
ALogP: 4.238
Rotatable Bonds: 7
Acceptors: 5
Donors: 1

Model Prediction

Prediction: Non-Toxic
Probability: 0.509
Enrichment: 0.969
Bayesian Score: -1.031
Mahalanobis Distance: 11.230
Mahalanobis Distance p-value: 0.000502

Prediction: Positive if the Bayesian score is above the estimated best cutoff value from minimizing the false positive and false negative rate.
Probability: The esimated probability that the sample is in the positive category. This assumes that the Bayesian score follows a normal distribution and is different from the prediction using a cutoff.
Enrichment: An estimate of enrichment, that is, the increased likelihood (versus random) of this sample being in the category.
Bayesian Score: The standard Laplacian-modified Bayesian score.
Mahalanobis Distance: The Mahalanobis distance (MD) is the distance to the center of the training data. The larger the MD, the less trustworthy the prediction.
Mahalanobis Distance p-value: The p-value gives the fraction of training data with an MD greater than or equal to the one for the given sample, assuming normally distributed data. The smaller the p-value, the less trustworthy the prediciton. For highly non-normal X properties (e.g., fingerprints), the MD p-value is wildly inaccurate.

Structural Similar Compounds

Name	Acemetacin	Amsacrine	Perphenazine
Structure	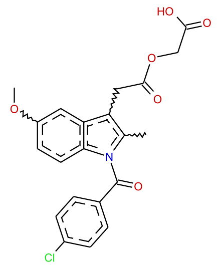	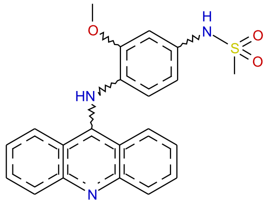	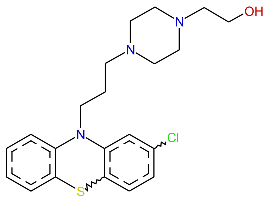
Actual Endpoint	Non-Toxic	Toxic	Toxic
Predicted Endpoint	Non-Toxic	Toxic	Toxic
Distance	0.498	0.610	0.632
Reference	Oyo Yakuri 22(6):777-786; 1981	Fundam Appl Toxicol 7(2):214-20; 1986	Toxicol Appl Pharmacol 21(2):230-6; 1972

Model Applicability

Unknown features are fingerprint features in the query molecule, but not found in the training set.

1. All properties and OPS components are within expected ranges.

Feature Contribution

Top features for positive contribution				
**Fingerprint**	**Bit/Smiles**	**Feature Structure**	**Score**	**Toxic in training set**
SCFP_6	1237755852	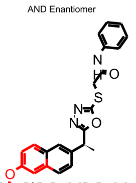 [*]:[c]1:[*]:[cH]:[cH]:[c](OC):[cH]:1	0.453	8 out of 9

SCFP_6	591469355	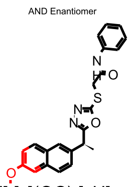<br>[*]:[cH]:[c](OC):[cH]:[*]	0.411	10 out of 12
SCFP_6	-2079162439	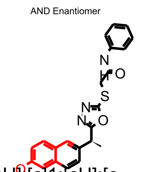<br>[*]:[cH]:[c]1:[cH]:[cH]:[c](OC):[cH]:[c]:1:[*]	0.271	1 out of 1
**Top Features for negative contribution**				
**Fingerprint**	**Bit/Smiles**	**Feature Structure**	**Score**	**Toxic in training set**
SCFP_6	149212520	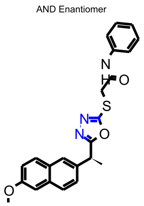<br>[*][c]1:[*]:[*]:n:n:1	-0.448	5 out of 16
SCFP_6	2097618059	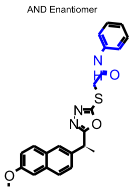<br>[*]CC(=O)N[c](:[cH]:[*]):[cH]:[*]	-0.422	0 out of 1
SCFP_6	1424234162	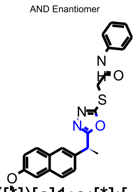<br>[*]C([*])[c]1:o:[*]:[*]:n:1	-0.422	0 out of 1

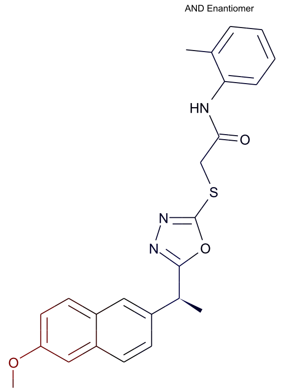

$C_{24}H_{23}N_3O_3S$
Molecular Weight: 433.52272
ALogP: 4.724
Rotatable Bonds: 7
Acceptors: 5
Donors: 1

Model Prediction

Prediction: Non-Toxic
Probability: 0.521
Enrichment: 0.991
Bayesian Score: -0.710
Mahalanobis Distance: 12.242
Mahalanobis Distance p-value: 1.1e-005

Prediction: Positive if the Bayesian score is above the estimated best cutoff value from minimizing the false positive and false negative rate.
Probability: The esimated probability that the sample is in the positive category. This assumes that the Bayesian score follows a normal distribution and is different from the prediction using a cutoff.
Enrichment: An estimate of enrichment, that is, the increased likelihood (versus random) of this sample being in the category.
Bayesian Score: The standard Laplacian-modified Bayesian score.
Mahalanobis Distance: The Mahalanobis distance (MD) is the distance to the center of the training data. The larger the MD, the less trustworthy the prediction.
Mahalanobis Distance p-value: The p-value gives the fraction of training data with an MD greater than or equal to the one for the given sample, assuming normally distributed data. The smaller the p-value, the less trustworthy the prediciton. For highly non-normal X properties (e.g., fingerprints), the MD p-value is wildly inaccurate.

Structural Similar Compounds

Name	Acemetacin	Amsacrine	Perphenazine
Structure	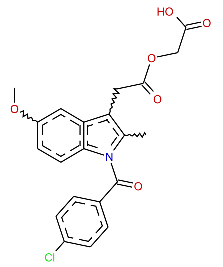	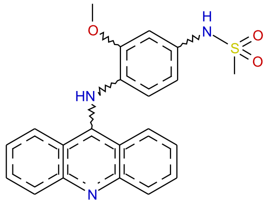	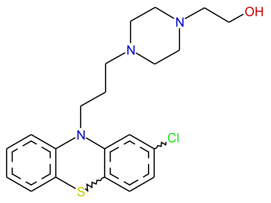
Actual Endpoint	Non-Toxic	Toxic	Toxic
Predicted Endpoint	Non-Toxic	Toxic	Toxic
Distance	0.522	0.630	0.630
Reference	Oyo Yakuri 22(6):777-786; 1981	Fundam Appl Toxicol 7(2):214-20; 1986	Toxicol Appl Pharmacol 21(2):230-6; 1972

Model Applicability

Unknown features are fingerprint features in the query molecule, but not found in the training set.

1. All properties and OPS components are within expected ranges.

Feature Contribution

Top features for positive contribution

Fingerprint	Bit/Smiles	Feature Structure	Score	Toxic in training set
SCFP_6	1237755852	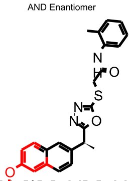 [*]:[c]1:[*]:[cH]:[cH]:[c](OC):[cH]:1	0.453	8 out of 9

SCFP_6	591469355	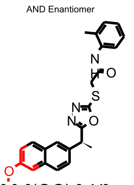 [*]:[cH]:[c](OC):[cH]:[*]	0.411	10 out of 12
SCFP_6	-2079162439	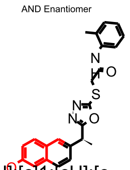 [*]:[cH]:[c]1:[cH]:[cH]:[c](OC):[cH]:[c]:1:[*]	0.271	1 out of 1

Top Features for negative contribution

Fingerprint	Bit/Smiles	Feature Structure	Score	Toxic in training set
SCFP_6	149212520	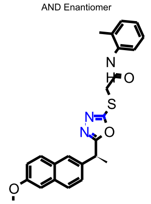 [*][c]1:[*]:[*]:n:n:1	-0.448	5 out of 16
SCFP_6	2097618059	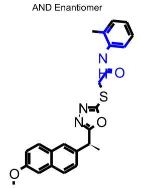 [*]CC(=O)N[c](:[cH]:[*]):[cH]:[*]	-0.422	0 out of 1
SCFP_6	-109786778	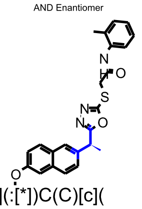 [*]:[c](:[*])C(C)[c](:[*]):[*]	-0.422	0 out of 1

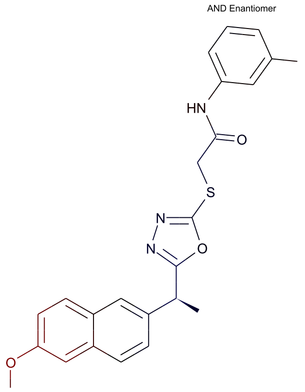

$C_{24}H_{23}N_3O_3S$
Molecular Weight: 433.52272
ALogP: 4.724
Rotatable Bonds: 7
Acceptors: 5
Donors: 1

Structural Similar Compounds

Name	Acemetacin	Amsacrine	Perphenazine
Structure	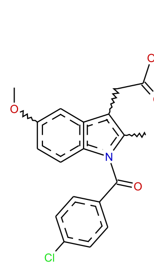	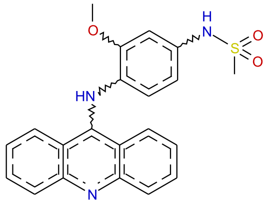	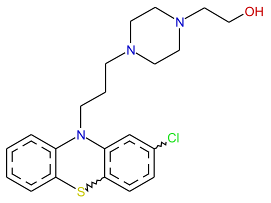
Actual Endpoint	Non-Toxic	Toxic	Toxic
Predicted Endpoint	Non-Toxic	Toxic	Toxic
Distance	0.522	0.630	0.631
Reference	Oyo Yakuri 22(6):777-786; 1981	Fundam Appl Toxicol 7(2):214-20; 1986	Toxicol Appl Pharmacol 21(2):230-6; 1972

Model Applicability

Unknown features are fingerprint features in the query molecule, but not found in the training set.

1. All properties and OPS components are within expected ranges.

Model Prediction

Prediction: Toxic

Probability: 0.549

Enrichment: 1.044

Bayesian Score: 0.040

Mahalanobis Distance: 11.245

Mahalanobis Distance p-value: 0.000478

Prediction: Positive if the Bayesian score is above the estimated best cutoff value from minimizing the false positive and false negative rate.
Probability: The esimated probability that the sample is in the positive category. This assumes that the Bayesian score follows a normal distribution and is different from the prediction using a cutoff.
Enrichment: An estimate of enrichment, that is, the increased likelihood (versus random) of this sample being in the category.
Bayesian Score: The standard Laplacian-modified Bayesian score.
Mahalanobis Distance: The Mahalanobis distance (MD) is the distance to the center of the training data. The larger the MD, the less trustworthy the prediction.
Mahalanobis Distance p-value: The p-value gives the fraction of training data with an MD greater than or equal to the one for the given sample, assuming normally distributed data. The smaller the p-value, the less trustworthy the prediciton. For highly non-normal X properties (e.g., fingerprints), the MD p-value is wildly inaccurate.

Feature Contribution

Top features for positive contribution

Fingerprint	Bit/Smiles	Feature Structure	Score	Toxic in training set
SCFP_6	1237755852	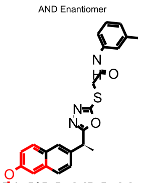 [*]:[c]1:[*]:[cH]:[cH]:[c](OC):[cH]:1	0.453	8 out of 9

SCFP_6	591469355	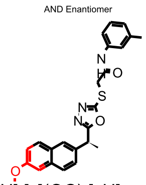[*]:[cH]:[c](OC):[cH]<br>:[*]	0.411	10 out of 12
SCFP_6	-347281112	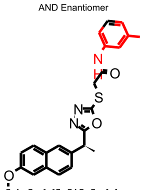[*]N[c]1:[cH]:[*]:[cH<br>]:[c](C):[cH]:1	0.381	2 out of 2

Top Features for negative contribution

Fingerprint	Bit/Smiles	Feature Structure	Score	Toxic in training set
SCFP_6	149212520	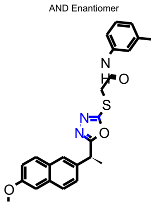[*][c]1:[*]:[*]:n:n:1	-0.448	5 out of 16
SCFP_6	-109786778	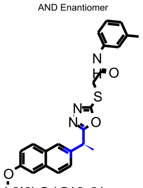[*]:[c](:[*])C(C)[c](<br>:[*]):[*]	-0.422	0 out of 1
SCFP_6	1424234162	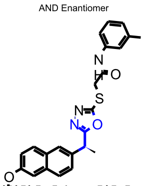[*]C([*])[c]1:o:[*]:[<br>*]:n:1	-0.422	0 out of 1

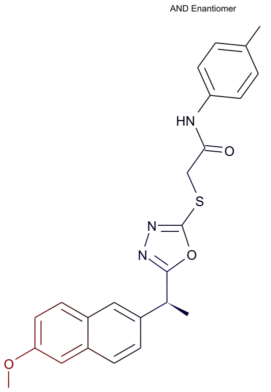

$C_{24}H_{23}N_3O_3S$
Molecular Weight: 433.52272
ALogP: 4.724
Rotatable Bonds: 7
Acceptors: 5
Donors: 1

Model Prediction

Prediction: Toxic
Probability: 0.545
Enrichment: 1.037
Bayesian Score: -0.059
Mahalanobis Distance: 10.601
Mahalanobis Distance p-value: 0.0039

Prediction: Positive if the Bayesian score is above the estimated best cutoff value from minimizing the false positive and false negative rate.
Probability: The esimated probability that the sample is in the positive category. This assumes that the Bayesian score follows a normal distribution and is different from the prediction using a cutoff.
Enrichment: An estimate of enrichment, that is, the increased likelihood (versus random) of this sample being in the category.
Bayesian Score: The standard Laplacian-modified Bayesian score.
Mahalanobis Distance: The Mahalanobis distance (MD) is the distance to the center of the training data. The larger the MD, the less trustworthy the prediction.
Mahalanobis Distance p-value: The p-value gives the fraction of training data with an MD greater than or equal to the one for the given sample, assuming normally distributed data. The smaller the p-value, the less trustworthy the prediciton. For highly non-normal X properties (e.g., fingerprints), the MD p-value is wildly inaccurate.

Structural Similar Compounds

Name	Acemetacin	Amsacrine	Perphenazine
Structure	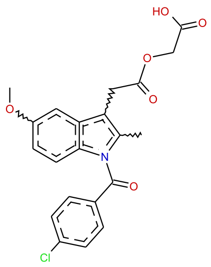	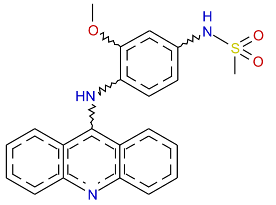	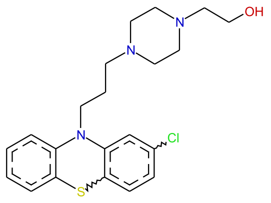
Actual Endpoint	Non-Toxic	Toxic	Toxic
Predicted Endpoint	Non-Toxic	Toxic	Toxic
Distance	0.520	0.634	0.634
Reference	Oyo Yakuri 22(6):777-786; 1981	Fundam Appl Toxicol 7(2):214-20; 1986	Toxicol Appl Pharmacol 21(2):230-6; 1972

Model Applicability

Unknown features are fingerprint features in the query molecule, but not found in the training set.

1. All properties and OPS components are within expected ranges.

Feature Contribution

Top features for positive contribution				
**Fingerprint**	**Bit/Smiles**	**Feature Structure**	**Score**	**Toxic in training set**
SCFP_6	1237755852	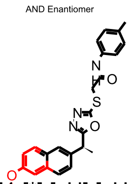 [*]:[c]1:[*]:[cH]:[cH]:[c](OC):[cH]:1	0.453	8 out of 9

SCFP_6	591469355	AND Enantiomer<br>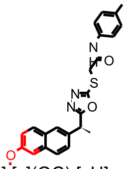<br>[*]:[cH]:[c](OC):[cH]<br>:[*]	0.411	10 out of 12
SCFP_6	795925860	AND Enantiomer<br>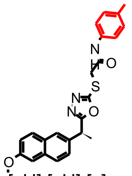<br>[*][c]1:[cH]:[cH]:[c]<br>(C):[cH]:[cH]:1	0.271	1 out of 1
**Top Features for negative contribution**				
**Fingerprint**	**Bit/Smiles**	**Feature Structure**	**Score**	**Toxic in training set**
SCFP_6	149212520	AND Enantiomer<br>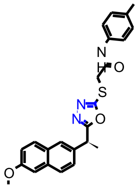<br>[*][c]1:[*]:[*]:n:n:1	-0.448	5 out of 16
SCFP_6	1424234162	AND Enantiomer<br>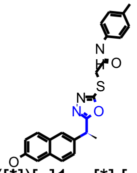<br>[*]C([*])[c]1:o:[*]:[<br>*]:n:1	-0.422	0 out of 1
SCFP_6	2097618059	AND Enantiomer<br>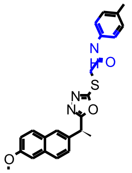<br>[*]CC(=O)N[c](:[cH]:[<br>*]):[cH]:[*]	-0.422	0 out of 1

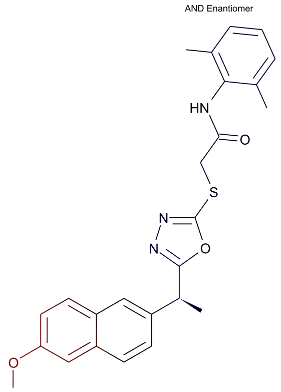

$C_{25}H_{25}N_3O_3S$
Molecular Weight: 447.5493
ALogP: 5.21
Rotatable Bonds: 7
Acceptors: 5
Donors: 1

Model Prediction

Prediction: Non-Toxic
Probability: 0.516
Enrichment: 0.982
Bayesian Score: -0.838
Mahalanobis Distance: 11.292
Mahalanobis Distance p-value: 0.000405

Prediction: Positive if the Bayesian score is above the estimated best cutoff value from minimizing the false positive and false negative rate.
Probability: The esimated probability that the sample is in the positive category. This assumes that the Bayesian score follows a normal distribution and is different from the prediction using a cutoff.
Enrichment: An estimate of enrichment, that is, the increased likelihood (versus random) of this sample being in the category.
Bayesian Score: The standard Laplacian-modified Bayesian score.
Mahalanobis Distance: The Mahalanobis distance (MD) is the distance to the center of the training data. The larger the MD, the less trustworthy the prediction.
Mahalanobis Distance p-value: The p-value gives the fraction of training data with an MD greater than or equal to the one for the given sample, assuming normally distributed data. The smaller the p-value, the less trustworthy the prediciton. For highly non-normal X properties (e.g., fingerprints), the MD p-value is wildly inaccurate.

Structural Similar Compounds

Name	Acemetacin	Estramustine Phosphate Disodium (Free acid form)	Brovanexine .HCl (Free base form)
Structure	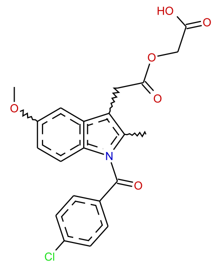	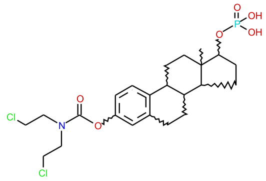	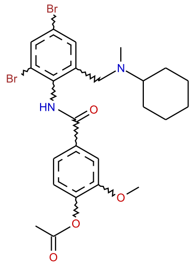
Actual Endpoint	Non-Toxic	Non-Toxic	Toxic
Predicted Endpoint	Non-Toxic	Non-Toxic	Toxic
Distance	0.561	0.635	0.646
Reference	Oyo Yakuri 22(6):777-786; 1981	Oyo Yakuri 20(6):1219-1236; 1980	Kiso to Rinsho 16(13):7179-7195; 1982

Model Applicability

Unknown features are fingerprint features in the query molecule, but not found in the training set.

1. All properties and OPS components are within expected ranges.

Feature Contribution

Top features for positive contribution

Fingerprint	Bit/Smiles	Feature Structure	Score	Toxic in training set
SCFP_6	1237755852	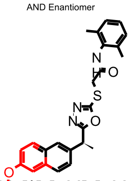 [*]:[c]1:[*]:[cH]:[cH]:[c](OC):[cH]:1	0.453	8 out of 9

SCFP_6	591469355	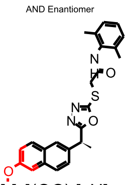 [*]:[cH]:[c](OC):[cH]:[*]	0.411	10 out of 12
SCFP_6	-1889730273	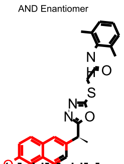 [*]O[c]1:[cH]:[cH]:[c]2:[cH]:[c]([*]):[*]:[cH]:[c]:2:[cH]:1	0.271	1 out of 1

Top Features for negative contribution

Fingerprint	Bit/Smiles	Feature Structure	Score	Toxic in training set
SCFP_6	149212520	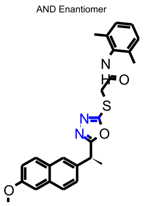 [*][c]1:[*]:[*]:n:n:1	-0.448	5 out of 16
SCFP_6	2097618059	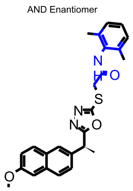 [*]CC(=O)N[c](:[cH]:[*]):[cH]:[*]	-0.422	0 out of 1
SCFP_6	-1630745304	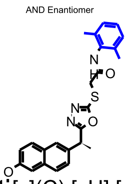 [*][c]1:[c](C):[cH]:[cH]:[cH]:[c]:1C	-0.422	0 out of 1

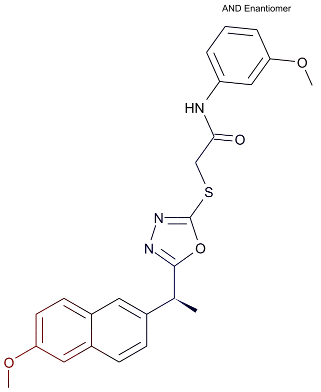

$C_{24}H_{23}N_3O_4S$
Molecular Weight: 449.52212
ALogP: 4.221
Rotatable Bonds: 8
Acceptors: 6
Donors: 1

Model Prediction

Prediction: Non-Toxic
Probability: 0.521
Enrichment: 0.990
Bayesian Score: -0.725
Mahalanobis Distance: 10.658
Mahalanobis Distance p-value: 0.00327

Prediction: Positive if the Bayesian score is above the estimated best cutoff value from minimizing the false positive and false negative rate.
Probability: The esimated probability that the sample is in the positive category. This assumes that the Bayesian score follows a normal distribution and is different from the prediction using a cutoff.
Enrichment: An estimate of enrichment, that is, the increased likelihood (versus random) of this sample being in the category.
Bayesian Score: The standard Laplacian-modified Bayesian score.
Mahalanobis Distance: The Mahalanobis distance (MD) is the distance to the center of the training data. The larger the MD, the less trustworthy the prediction.
Mahalanobis Distance p-value: The p-value gives the fraction of training data with an MD greater than or equal to the one for the given sample, assuming normally distributed data. The smaller the p-value, the less trustworthy the prediciton. For highly non-normal X properties (e.g., fingerprints), the MD p-value is wildly inaccurate.

Structural Similar Compounds

Name	Acemetacin	Nicardipine	Hydrocortisone-17-butyrate-21-propionate
Structure	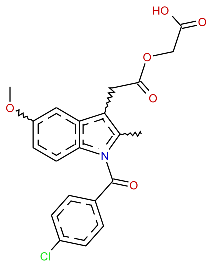	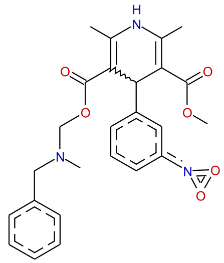	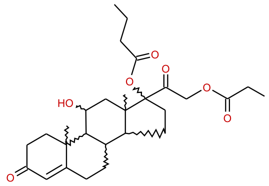
Actual Endpoint	Non-Toxic	Non-Toxic	Toxic
Predicted Endpoint	Non-Toxic	Non-Toxic	Toxic
Distance	0.505	0.593	0.597
Reference	Oyo Yakuri 22(6):777-786; 1981	Kiso to Rinsho 13:1149-1159; 1979	Oyo Yakuri 21:441-466; 1981

Model Applicability

Unknown features are fingerprint features in the query molecule, but not found in the training set.

1. All properties and OPS components are within expected ranges.

Feature Contribution

Top features for positive contribution				
**Fingerprint**	**Bit/Smiles**	**Feature Structure**	**Score**	**Toxic in training set**
SCFP_6	1237755852	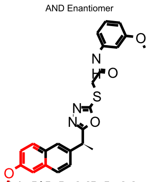 [*]:[c]1:[*]:[cH]:[cH]:[c](OC):[cH]:1	0.453	8 out of 9

SCFP_6	591469355	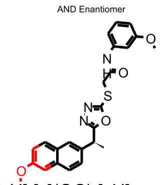 [*]:[cH]:[c](OC):[cH]:[*]	0.411	10 out of 12
SCFP_6	-1889730273	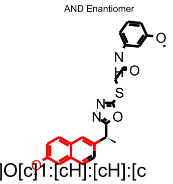 [*]O[c]1:[cH]:[cH]:[c]2:[cH]:[c]([*]):[*]:[cH]:[c]:2:[cH]:1	0.271	1 out of 1
**Top Features for negative contribution**				
**Fingerprint**	**Bit/Smiles**	**Feature Structure**	**Score**	**Toxic in training set**
SCFP_6	149212520	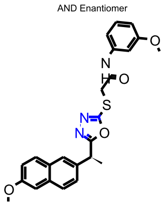 [*][c]1:[*]:[*]:n:n:1	-0.448	5 out of 16
SCFP_6	1424234162	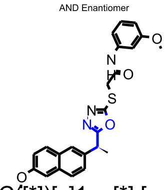 [*]C([*])[c]1:o:[*]:[*]:n:1	-0.422	0 out of 1
SCFP_6	-109786778	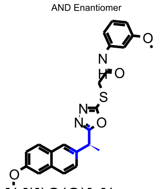 [*]:[c](:[*])C(C)[c](:[*]):[*]	-0.422	0 out of 1

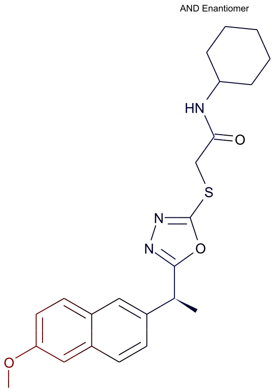

$C_{23}H_{27}N_3O_3S$

Molecular Weight: 425.54378

ALogP: 4.52

Rotatable Bonds: 7

Acceptors: 5

Donors: 1

Model Prediction

Prediction: Non-Toxic

Probability: 0.498

Enrichment: 0.946

Bayesian Score: -1.358

Mahalanobis Distance: 11.981

Mahalanobis Distance p-value: 3.12e-005

Prediction: Positive if the Bayesian score is above the estimated best cutoff value from minimizing the false positive and false negative rate.
Probability: The esimated probability that the sample is in the positive category. This assumes that the Bayesian score follows a normal distribution and is different from the prediction using a cutoff.
Enrichment: An estimate of enrichment, that is, the increased likelihood (versus random) of this sample being in the category.
Bayesian Score: The standard Laplacian-modified Bayesian score.
Mahalanobis Distance: The Mahalanobis distance (MD) is the distance to the center of the training data. The larger the MD, the less trustworthy the prediction.
Mahalanobis Distance p-value: The p-value gives the fraction of training data with an MD greater than or equal to the one for the given sample, assuming normally distributed data. The smaller the p-value, the less trustworthy the prediciton. For highly non-normal X properties (e.g., fingerprints), the MD p-value is wildly inaccurate.

Structural Similar Compounds

Name	Acemetacin	Perphenazine	Amsacrine
Structure	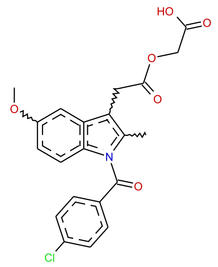	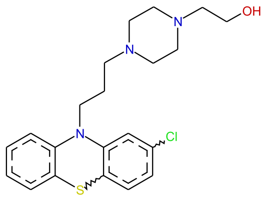	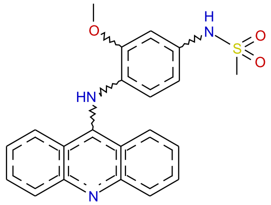
Actual Endpoint	Non-Toxic	Toxic	Toxic
Predicted Endpoint	Non-Toxic	Toxic	Toxic
Distance	0.511	0.628	0.634
Reference	Oyo Yakuri 22(6):777-786; 1981	Toxicol Appl Pharmacol 21(2):230-6; 1972	Fundam Appl Toxicol 7(2):214-20; 1986

Model Applicability

Unknown features are fingerprint features in the query molecule, but not found in the training set.

1. OPS PC12 out of range. Value: 4.1681. Training min, max, SD, explained variance: -3.7514, 3.3159, 1.318, 0.0255.

Feature Contribution

Top features for positive contribution				
**Fingerprint**	**Bit/Smiles**	**Feature Structure**	**Score**	**Toxic in training set**
SCFP_6	1237755852	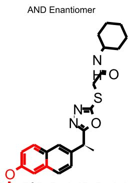 [*]:[c]1:[*]:[cH]:[cH]:[c](OC):[cH]:1	0.453	8 out of 9

SCFP_6	591469355	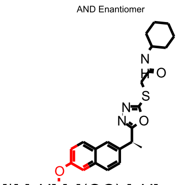 [*]:[cH]:[c](OC):[cH]:[*]	0.411	10 out of 12
SCFP_6	-1889730273	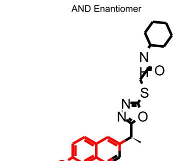 [*]O[c]1:[cH]:[cH]:[c]2:[cH]:[c]([*]):[*]:[cH]:[c]:2:[cH]:1	0.271	1 out of 1

Top Features for negative contribution

Fingerprint	Bit/Smiles	Feature Structure	Score	Toxic in training set
SCFP_6	149212520	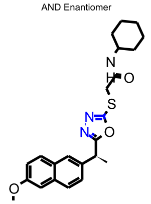 [*][c]1:[*]:[*]:n:n:1	-0.448	5 out of 16
SCFP_6	796516404	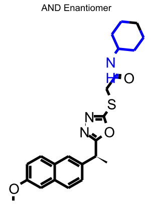 [*]C(=[*])NC1CC[*]CC1	-0.422	0 out of 1
SCFP_6	1424234162	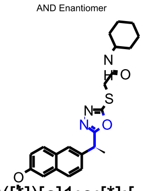 [*]C([*])[c]1:o:[*]:[*]:n:1	-0.422	0 out of 1

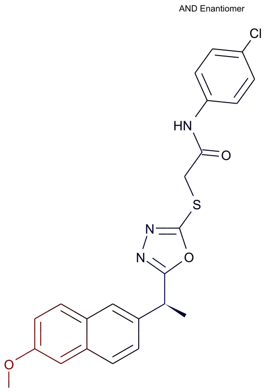

$C_{23}H_{20}ClN_3O_3S$
Molecular Weight: 453.9412
ALogP: 4.902
Rotatable Bonds: 7
Acceptors: 5
Donors: 1

Model Prediction

Prediction: Toxic
Probability: 0.543
Enrichment: 1.033
Bayesian Score: -0.108
Mahalanobis Distance: 10.608
Mahalanobis Distance p-value: 0.0038

Prediction: Positive if the Bayesian score is above the estimated best cutoff value from minimizing the false positive and false negative rate.
Probability: The esimated probability that the sample is in the positive category. This assumes that the Bayesian score follows a normal distribution and is different from the prediction using a cutoff.
Enrichment: An estimate of enrichment, that is, the increased likelihood (versus random) of this sample being in the category.
Bayesian Score: The standard Laplacian-modified Bayesian score.
Mahalanobis Distance: The Mahalanobis distance (MD) is the distance to the center of the training data. The larger the MD, the less trustworthy the prediction.
Mahalanobis Distance p-value: The p-value gives the fraction of training data with an MD greater than or equal to the one for the given sample, assuming normally distributed data. The smaller the p-value, the less trustworthy the prediciton. For highly non-normal X properties (e.g., fingerprints), the MD p-value is wildly inaccurate.

Structural Similar Compounds

Name	Acemetacin	Perphenazine	Estramustine Phosphate Disodium (Free acid form)
Structure	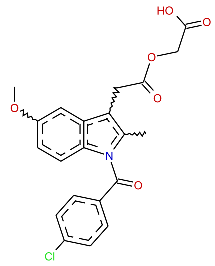	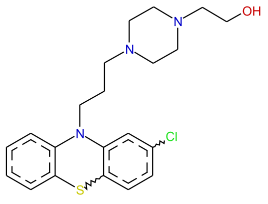	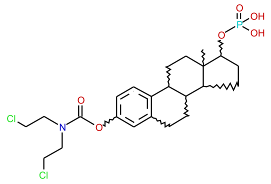
Actual Endpoint	Non-Toxic	Toxic	Non-Toxic
Predicted Endpoint	Non-Toxic	Toxic	Non-Toxic
Distance	0.518	0.629	0.632
Reference	Oyo Yakuri 22(6):777-786; 1981	Toxicol Appl Pharmacol 21(2):230-6; 1972	Oyo Yakuri 20(6):1219-1236; 1980

Model Applicability

Unknown features are fingerprint features in the query molecule, but not found in the training set.

1. All properties and OPS components are within expected ranges.

Feature Contribution

Top features for positive contribution				
Fingerprint	Bit/Smiles	Feature Structure	Score	Toxic in training set
SCFP_6	1237755852	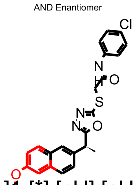 [*]:[c]1:[*]:[cH]:[cH]:[c](OC):[cH]:1	0.453	8 out of 9

SCFP_6	591469355	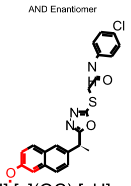 [*]:[cH]:[c](OC):[cH] :[*]	0.411	10 out of 12
SCFP_6	-1889730273	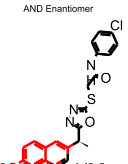 [*]O[c]1:[cH]:[cH]:[c]2:[cH]:[c]([*]):[*] :[cH]:[c]:2:[cH]:1	0.271	1 out of 1

Top Features for negative contribution

Fingerprint	Bit/Smiles	Feature Structure	Score	Toxic in training set
SCFP_6	149212520	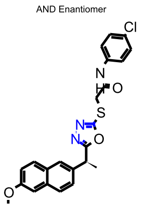 [*][c]1:[*]:[*]:n:n:1	-0.448	5 out of 16
SCFP_6	-109786778	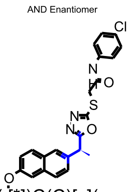 [*]:[c](:[*])C(C)[c](:[*]):[*]	-0.422	0 out of 1
SCFP_6	1424234162	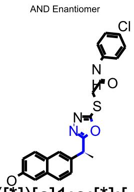 [*]C([*])[c]1:o:[*]:[*]:n:1	-0.422	0 out of 1

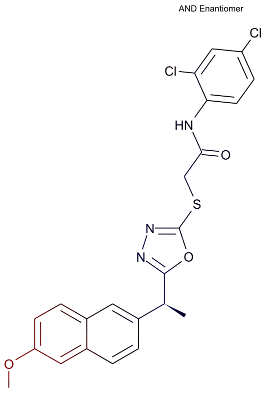

$C_{23}H_{19}Cl_2N_3O_3S$
Molecular Weight: 488.38626
ALogP: 5.567
Rotatable Bonds: 7
Acceptors: 5
Donors: 1

Model Prediction

Prediction: Toxic
Probability: 0.540
Enrichment: 1.027
Bayesian Score: -0.203
Mahalanobis Distance: 10.680
Mahalanobis Distance p-value: 0.00306

Prediction: Positive if the Bayesian score is above the estimated best cutoff value from minimizing the false positive and false negative rate.
Probability: The esimated probability that the sample is in the positive category. This assumes that the Bayesian score follows a normal distribution and is different from the prediction using a cutoff.
Enrichment: An estimate of enrichment, that is, the increased likelihood (versus random) of this sample being in the category.
Bayesian Score: The standard Laplacian-modified Bayesian score.
Mahalanobis Distance: The Mahalanobis distance (MD) is the distance to the center of the training data. The larger the MD, the less trustworthy the prediction.
Mahalanobis Distance p-value: The p-value gives the fraction of training data with an MD greater than or equal to the one for the given sample, assuming normally distributed data. The smaller the p-value, the less trustworthy the prediciton. For highly non-normal X properties (e.g., fingerprints), the MD p-value is wildly inaccurate.

Structural Similar Compounds

Name	Acemetacin	Brovanexine .HCl (Free base form)	Estramustine Phosphate Disodium (Free acid form)
Structure	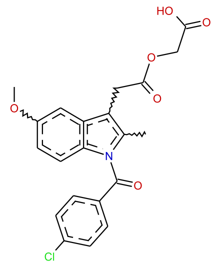	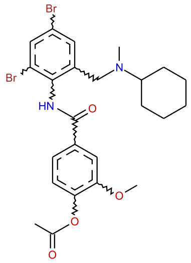	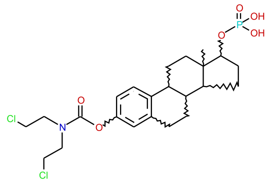
Actual Endpoint	Non-Toxic	Toxic	Non-Toxic
Predicted Endpoint	Non-Toxic	Toxic	Non-Toxic
Distance	0.594	0.605	0.613
Reference	Oyo Yakuri 22(6):777-786; 1981	Kiso to Rinsho 16(13):7179-7195; 1982	Oyo Yakuri 20(6):1219-1236; 1980

Model Applicability

Unknown features are fingerprint features in the query molecule, but not found in the training set.

1. All properties and OPS components are within expected ranges.

Feature Contribution

Top features for positive contribution				
**Fingerprint**	**Bit/Smiles**	**Feature Structure**	**Score**	**Toxic in training set**
SCFP_6	1237755852	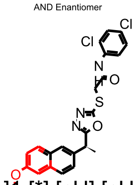 [*]:[c]1:[*]:[cH]:[cH]:[c](OC):[cH]:1	0.453	8 out of 9

SCFP_6	591469355	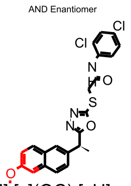<br>[*]:[cH]:[c](OC):[cH]:[*]	0.411	10 out of 12
SCFP_6	1908972582	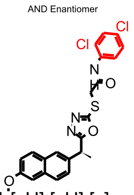<br>[*][c]1:[cH]:[cH]:[c](Cl):[cH]:[c]:1Cl	0.381	2 out of 2
**Top Features for negative contribution**				
**Fingerprint**	**Bit/Smiles**	**Feature Structure**	**Score**	**Toxic in training set**
SCFP_6	149212520	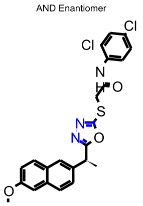<br>[*][c]1:[*]:[*]:n:n:1	-0.448	5 out of 16
SCFP_6	1424234162	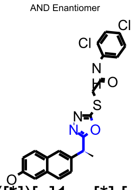<br>[*]C([*])[c]1:o:[*]:[*]:n:1	-0.422	0 out of 1
SCFP_6	-1212269302	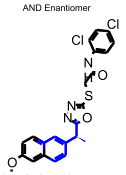<br>[*]:[c](:[*])[C@@H](C)[c]1:[cH]:[cH]:[*]:[c](:[*]):[cH]:1	-0.422	0 out of 1

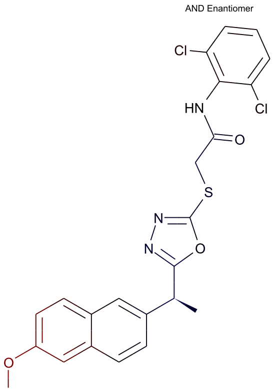

$C_{23}H_{19}Cl_2N_3O_3S$
Molecular Weight: 488.38626
ALogP: 5.567
Rotatable Bonds: 7
Acceptors: 5
Donors: 1

Model Prediction

Prediction: Toxic
Probability: 0.551
Enrichment: 1.048
Bayesian Score: 0.098
Mahalanobis Distance: 11.593
Mahalanobis Distance p-value: 0.000137

Prediction: Positive if the Bayesian score is above the estimated best cutoff value from minimizing the false positive and false negative rate.
Probability: The esimated probability that the sample is in the positive category. This assumes that the Bayesian score follows a normal distribution and is different from the prediction using a cutoff.
Enrichment: An estimate of enrichment, that is, the increased likelihood (versus random) of this sample being in the category.
Bayesian Score: The standard Laplacian-modified Bayesian score.
Mahalanobis Distance: The Mahalanobis distance (MD) is the distance to the center of the training data. The larger the MD, the less trustworthy the prediction.
Mahalanobis Distance p-value: The p-value gives the fraction of training data with an MD greater than or equal to the one for the given sample, assuming normally distributed data. The smaller the p-value, the less trustworthy the prediciton. For highly non-normal X properties (e.g., fingerprints), the MD p-value is wildly inaccurate.

Structural Similar Compounds

Name	Acemetacin	Brovanexine .HCl (Free base form)	Estramustine Phosphate Disodium (Free acid form)
Structure	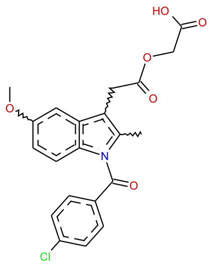	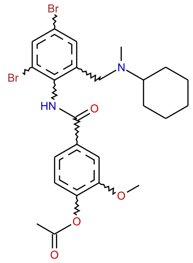	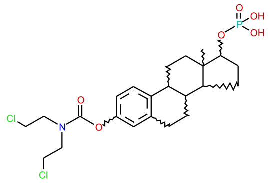
Actual Endpoint	Non-Toxic	Toxic	Non-Toxic
Predicted Endpoint	Non-Toxic	Toxic	Non-Toxic
Distance	0.596	0.603	0.612
Reference	Oyo Yakuri 22(6):777-786; 1981	Kiso to Rinsho 16(13):7179-7195; 1982	Oyo Yakuri 20(6):1219-1236; 1980

Model Applicability

Unknown features are fingerprint features in the query molecule, but not found in the training set.

1. All properties and OPS components are within expected ranges.

Feature Contribution

Top features for positive contribution				
**Fingerprint**	**Bit/Smiles**	**Feature Structure**	**Score**	**Toxic in training set**
SCFP_6	1237755852	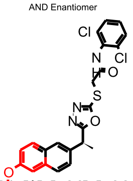 [*]:[c]1:[*]:[cH]:[cH]:[c](OC):[cH]:1	0.453	8 out of 9

SCFP_6	591469355	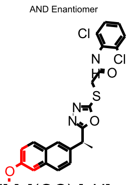 [*]:[cH]:[c](OC):[cH]:[*]	0.411	10 out of 12
SCFP_6	1062412764	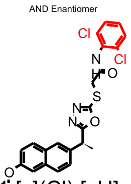 [*][c]1:[c](Cl):[cH]:[cH]:[cH]:[c]:1Cl	0.381	2 out of 2

Top Features for negative contribution

Fingerprint	Bit/Smiles	Feature Structure	Score	Toxic in training set
SCFP_6	149212520	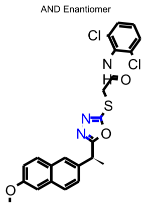 [*][c]1:[*]:[*]:n:n:1	-0.448	5 out of 16
SCFP_6	2097618059	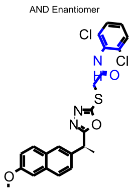 [*]CC(=O)N[c](:[cH]:[*]):[cH]:[*]	-0.422	0 out of 1
SCFP_6	1424234162	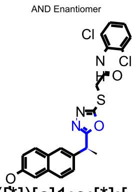 [*]C([*])[c]1:o:[*]:[*]:n:1	-0.422	0 out of 1

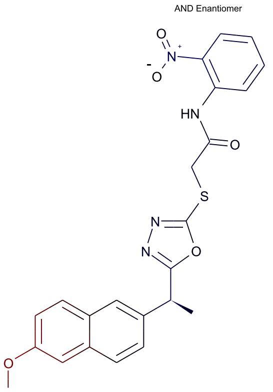

$C_{23}H_{20}N_4O_5S$

Molecular Weight: 464.4937

ALogP: 4.132

Rotatable Bonds: 8

Acceptors: 7

Donors: 1

Structural Similar Compounds

Name	Acemetacin	Hydrocortisone-17-butyrate-21-propionate	Beclomethasone Dipropionate
Structure	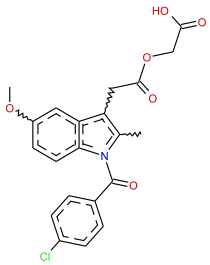	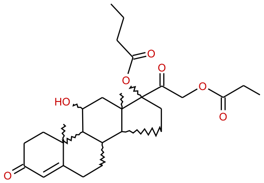	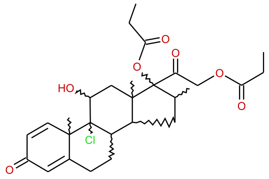
Actual Endpoint	Non-Toxic	Toxic	Toxic
Predicted Endpoint	Non-Toxic	Toxic	Toxic
Distance	0.595	0.649	0.659
Reference	Oyo Yakuri 22(6):777-786; 1981	Oyo Yakuri 21:441-466; 1981	Oyo Yakuri 18(6):1021-1038; 1979

Model Prediction

Prediction: Non-Toxic

Probability: 0.468

Enrichment: 0.889

Bayesian Score: -2.227

Mahalanobis Distance: 11.200

Mahalanobis Distance p-value: 0.000556

Prediction: Positive if the Bayesian score is above the estimated best cutoff value from minimizing the false positive and false negative rate.
Probability: The esimated probability that the sample is in the positive category. This assumes that the Bayesian score follows a normal distribution and is different from the prediction using a cutoff.
Enrichment: An estimate of enrichment, that is, the increased likelihood (versus random) of this sample being in the category.
Bayesian Score: The standard Laplacian-modified Bayesian score.
Mahalanobis Distance: The Mahalanobis distance (MD) is the distance to the center of the training data. The larger the MD, the less trustworthy the prediction.
Mahalanobis Distance p-value: The p-value gives the fraction of training data with an MD greater than or equal to the one for the given sample, assuming normally distributed data. The smaller the p-value, the less trustworthy the prediciton. For highly non-normal X properties (e.g., fingerprints), the MD p-value is wildly inaccurate.

Model Applicability

Unknown features are fingerprint features in the query molecule, but not found in the training set.

1. All properties and OPS components are within expected ranges.

Feature Contribution

Top features for positive contribution

Fingerprint	Bit/Smiles	Feature Structure	Score	Toxic in training set
SCFP_6	1237755852	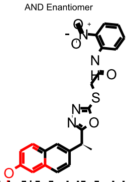 [*]:[c]1:[*]:[cH]:[cH]:[c](OC):[cH]:1	0.453	8 out of 9

SCFP_6	591469355	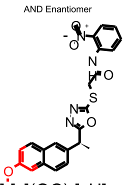 [*]:[cH]:[c](OC):[cH]:[*]	0.411	10 out of 12
SCFP_6	-1889730273	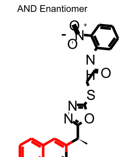 [*]O[c]1:[cH]:[cH]:[c]2:[cH]:[c]([*]):[*]:[cH]:[c]:2:[cH]:1	0.271	1 out of 1

Top Features for negative contribution

Fingerprint	Bit/Smiles	Feature Structure	Score	Toxic in training set
SCFP_6	-1380909229	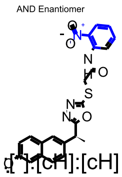 [*][c]1:[*]:[cH]:[cH]:[cH]:[c]:1[N+](=[*])[*]	-0.449	6 out of 19
SCFP_6	149212520	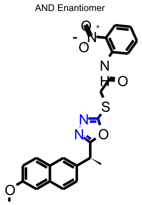 [*][c]1:[*]:[*]:n:n:1	-0.448	5 out of 16
SCFP_6	1311339974	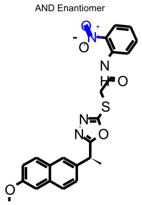 [*][N+](=O)[*]	-0.446	3 out of 10

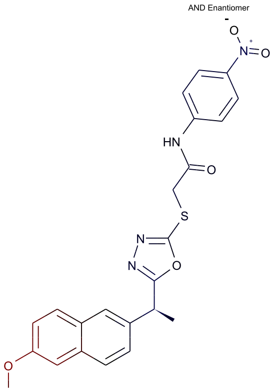

$C_{23}H_{20}N_4O_5S$
Molecular Weight: 464.4937
ALogP: 4.132
Rotatable Bonds: 8
Acceptors: 7
Donors: 1

Model Prediction

Prediction: Non-Toxic
Probability: 0.483
Enrichment: 0.918
Bayesian Score: -1.789
Mahalanobis Distance: 10.800
Mahalanobis Distance p-value: 0.0021

Prediction: Positive if the Bayesian score is above the estimated best cutoff value from minimizing the false positive and false negative rate.
Probability: The esimated probability that the sample is in the positive category. This assumes that the Bayesian score follows a normal distribution and is different from the prediction using a cutoff.
Enrichment: An estimate of enrichment, that is, the increased likelihood (versus random) of this sample being in the category.
Bayesian Score: The standard Laplacian-modified Bayesian score.
Mahalanobis Distance: The Mahalanobis distance (MD) is the distance to the center of the training data. The larger the MD, the less trustworthy the prediction.
Mahalanobis Distance p-value: The p-value gives the fraction of training data with an MD greater than or equal to the one for the given sample, assuming normally distributed data. The smaller the p-value, the less trustworthy the prediciton. For highly non-normal X properties (e.g., fingerprints), the MD p-value is wildly inaccurate.

Structural Similar Compounds

**Name**	**Acemetacin**	**Hydrocortisone-17-butyrate-21-propionate**	**Beclomethasone Dipropionate**
Structure	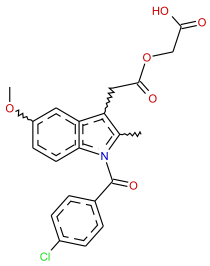	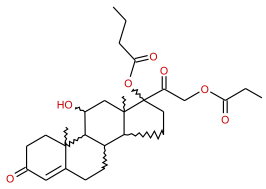	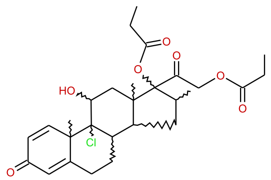
Actual Endpoint	Non-Toxic	Toxic	Toxic
Predicted Endpoint	Non-Toxic	Toxic	Toxic
Distance	0.593	0.649	0.658
Reference	Oyo Yakuri 22(6):777-786; 1981	Oyo Yakuri 21:441-466; 1981	Oyo Yakuri 18(6):1021-1038; 1979

Model Applicability

Unknown features are fingerprint features in the query molecule, but not found in the training set.

1. All properties and OPS components are within expected ranges.

Feature Contribution

Top features for positive contribution

Fingerprint	Bit/Smiles	Feature Structure	Score	Toxic in training set
SCFP_6	1237755852	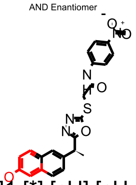 [*]:[c]1:[*]:[cH]:[cH]:[c](OC):[cH]:1	0.453	8 out of 9

SCFP_6	591469355	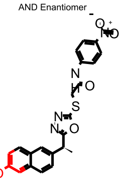 [*]:[cH]:[c](OC):[cH]:[*]	0.411	10 out of 12
SCFP_6	-1889730273	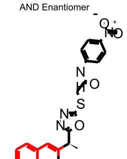 [*]O[c]1:[cH]:[cH]:[c]2:[cH]:[c]([*]):[*]:[cH]:[c]:2:[cH]:1	0.271	1 out of 1
**Top Features for negative contribution**				
**Fingerprint**	**Bit/Smiles**	**Feature Structure**	**Score**	**Toxic in training set**
SCFP_6	-1380909229	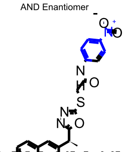 [*][c]1:[*]:[cH]:[cH]:[cH]:[c]:1[N+](=[*])[*]	-0.449	6 out of 19
SCFP_6	149212520	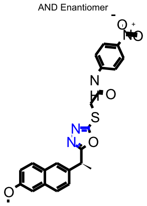 [*][c]1:[*]:[*]:n:n:1	-0.448	5 out of 16
SCFP_6	1311339974	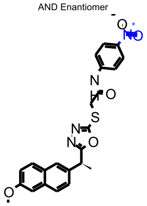 [*][N+](=O)[*]	-0.446	3 out of 10

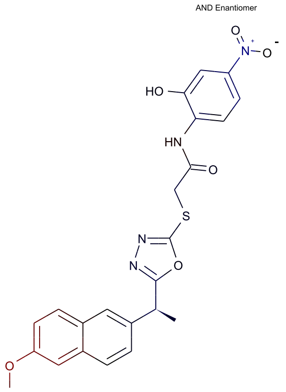

$C_{23}H_{20}N_4O_6S$
Molecular Weight: 480.4931
ALogP: 3.89
Rotatable Bonds: 8
Acceptors: 8
Donors: 2

Model Prediction

Prediction: Non-Toxic
Probability: 0.467
Enrichment: 0.889
Bayesian Score: -2.237
Mahalanobis Distance: 11.749
Mahalanobis Distance p-value: 7.63e-005

Prediction: Positive if the Bayesian score is above the estimated best cutoff value from minimizing the false positive and false negative rate.
Probability: The esimated probability that the sample is in the positive category. This assumes that the Bayesian score follows a normal distribution and is different from the prediction using a cutoff.
Enrichment: An estimate of enrichment, that is, the increased likelihood (versus random) of this sample being in the category.
Bayesian Score: The standard Laplacian-modified Bayesian score.
Mahalanobis Distance: The Mahalanobis distance (MD) is the distance to the center of the training data. The larger the MD, the less trustworthy the prediction.
Mahalanobis Distance p-value: The p-value gives the fraction of training data with an MD greater than or equal to the one for the given sample, assuming normally distributed data. The smaller the p-value, the less trustworthy the prediciton. For highly non-normal X properties (e.g., fingerprints), the MD p-value is wildly inaccurate.

Structural Similar Compounds

Name	Acemetacin	Reserpate	Bacampicillin .HCl (Free base form)
Structure	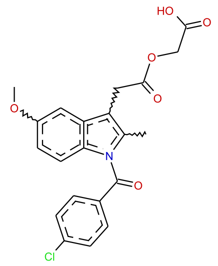	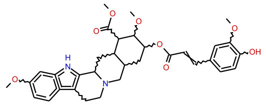	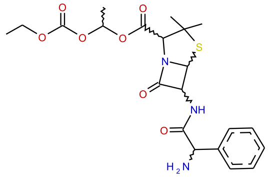
Actual Endpoint	Non-Toxic	Toxic	Toxic
Predicted Endpoint	Non-Toxic	Toxic	Non-Toxic
Distance	0.702	0.715	0.718
Reference	Oyo Yakuri 22(6):777-786; 1981	Oyo Yakuri 18:105-124; 1979	Chemotherapy 27:30-35; 1979

Model Applicability

Unknown features are fingerprint features in the query molecule, but not found in the training set.

1. All properties and OPS components are within expected ranges.

Feature Contribution

Top features for positive contribution

Fingerprint	Bit/Smiles	Feature Structure	Score	Toxic in training set
SCFP_6	1237755852	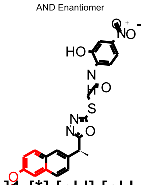 [*]:[c]1:[*]:[cH]:[cH]:[c](OC):[cH]:1	0.453	8 out of 9

SCFP_6	591469355	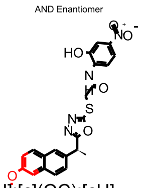 [*]:[cH]:[c](OC):[cH]:[*]	0.411	10 out of 12
SCFP_6	-1928055023	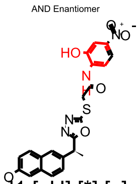 [*]N[c]1:[cH]:[*]:[c]([*]):[cH]:[c]:1O	0.381	2 out of 2
**Top Features for negative contribution**				
**Fingerprint**	**Bit/Smiles**	**Feature Structure**	**Score**	**Toxic in training set**
SCFP_6	124239044	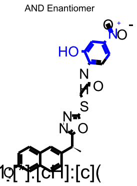 [*][c]1:[*]:[cH]:[c](:[cH]:[c]:1O)[N+](=[*])[*]	-1.131	0 out of 4
SCFP_6	-1380909229	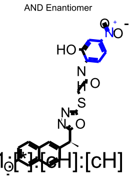 [*][c]1:[*]:[cH]:[cH]:[cH]:[c]:1[N+](=[*])[*]	-0.449	6 out of 19
SCFP_6	149212520	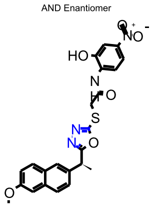 [*][c]1:[*]:[*]:n:n:1	-0.448	5 out of 16

Sorafenib

TOPKAT_Developmental_Toxicity_Potential

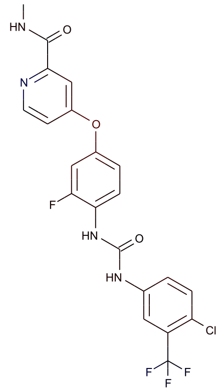

$C_{21}H_{15}ClF_{4}N_{4}O_{3}$

Molecular Weight: 482.81541

ALogP: 4.381

Rotatable Bonds: 6

Acceptors: 4

Donors: 3

Model Prediction

Prediction: Toxic

Probability: 0.585

Enrichment: 1.113

Bayesian Score: 0.984

Mahalanobis Distance: 12.647

Mahalanobis Distance p-value: 2.05e-006

Prediction: Positive if the Bayesian score is above the estimated best cutoff value from minimizing the false positive and false negative rate.
Probability: The esimated probability that the sample is in the positive category. This assumes that the Bayesian score follows a normal distribution and is different from the prediction using a cutoff.
Enrichment: An estimate of enrichment, that is, the increased likelihood (versus random) of this sample being in the category.
Bayesian Score: The standard Laplacian-modified Bayesian score.
Mahalanobis Distance: The Mahalanobis distance (MD) is the distance to the center of the training data. The larger the MD, the less trustworthy the prediction.
Mahalanobis Distance p-value: The p-value gives the fraction of training data with an MD greater than or equal to the one for the given sample, assuming normally distributed data. The smaller the p-value, the less trustworthy the prediciton. For highly non-normal X properties (e.g., fingerprints), the MD p-value is wildly inaccurate.

Structural Similar Compounds

Name	Chenodiol	Amsacrine	Ochratoxin a
Structure	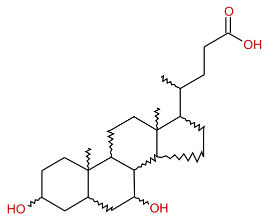	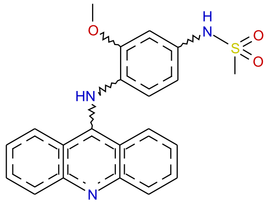	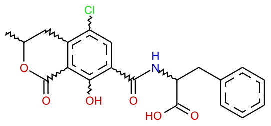
Actual Endpoint	Toxic	Toxic	Toxic
Predicted Endpoint	Toxic	Toxic	Toxic
Distance	0.649	0.658	0.669
Reference	Arch Int Pharm 246:149-158; 1980	Fundam Appl Toxicol 7(2):214-20; 1986	Toxicol Appl Pharmacol 37(2):331-8; 1976

Model Applicability

Unknown features are fingerprint features in the query molecule, but not found in the training set.

1. All properties and OPS components are within expected ranges.

Feature Contribution

Top features for positive contribution

Fingerprint	Bit/Smiles	Feature Structure	Score	Toxic in training set
SCFP_6	1559190850	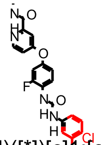 [*]C([*])([*])[c]1:[cH]:[*]:[cH]:[cH]:[c]:1Cl	0.441	3 out of 3

SCFP_6	-488587948	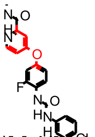 [*][c]1:[*]:[cH]:[cH]:[c](O[c](:[*]):[*]):[cH]:1	0.381	2 out of 2
SCFP_6	-347281112	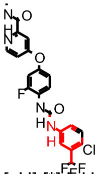 [*]N[c]1:[cH]:[*]:[cH]:[c](C):[cH]:1	0.381	2 out of 2

Top Features for negative contribution

Fingerprint	Bit/Smiles	Feature Structure	Score	Toxic in training set
SCFP_6	-1794974220	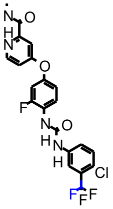 [*]C([*])([*])F	-0.550	2 out of 8
SCFP_6	-937094999	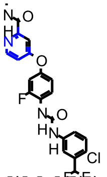 [*][c]1:[*]:[c]([*]):n:[cH]:[cH]:1	-0.358	3 out of 9
SCFP_6	-496201075	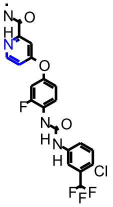 [*]:[cH]:[cH]:n:[*]	-0.289	8 out of 21

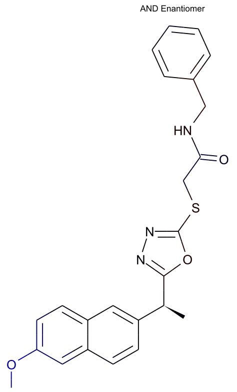

$C_{24}H_{23}N_3O_3S$
Molecular Weight: 433.52272
ALogP: 4.245
Rotatable Bonds: 8
Acceptors: 5
Donors: 1

Model Prediction

Prediction: Non-Carcinogen
Probability: 0.230
Enrichment: 0.718
Bayesian Score: -2.045
Mahalanobis Distance: 14.035
Mahalanobis Distance p-value: 1.29e-005

Prediction: Positive if the Bayesian score is above the estimated best cutoff value from minimizing the false positive and false negative rate.
Probability: The esimated probability that the sample is in the positive category. This assumes that the Bayesian score follows a normal distribution and is different from the prediction using a cutoff.
Enrichment: An estimate of enrichment, that is, the increased likelihood (versus random) of this sample being in the category.
Bayesian Score: The standard Laplacian-modified Bayesian score.
Mahalanobis Distance: The Mahalanobis distance (MD) is the distance to the center of the training data. The larger the MD, the less trustworthy the prediction.
Mahalanobis Distance p-value: The p-value gives the fraction of training data with an MD greater than or equal to the one for the given sample, assuming normally distributed data. The smaller the p-value, the less trustworthy the prediciton. For highly non-normal X properties (e.g., fingerprints), the MD p-value is wildly inaccurate.

Structural Similar Compounds

Name	Lovastatin	Felodipine	Simvastatin
Structure	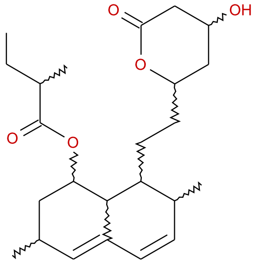	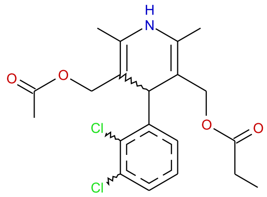	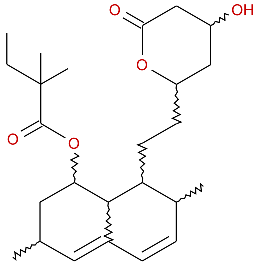
Actual Endpoint	Carcinogen	Non-Carcinogen	Carcinogen
Predicted Endpoint	Carcinogen	Non-Carcinogen	Carcinogen
Distance	0.575	0.581	0.591
Reference	US FDA (Centre for Drug Eval.& Res./Off. Testing & Res.) Sept. 1997	US FDA (Centre for Drug Eval.& Res./Off. Testing & Res.) Sept. 1997	US FDA (Centre for Drug Eval.& Res./Off. Testing & Res.) Sept. 1997

Model Applicability

Unknown features are fingerprint features in the query molecule, but not found in the training set.

1. All properties and OPS components are within expected ranges.
2. Unknown ECFP_2 feature: -955816473: [*]SCC(=[*])[*]
3. Unknown ECFP_2 feature: 1093109320: [*]S[c]1:o:[*]:[*]:n:1
4. Unknown ECFP_2 feature: 1427820655: [*]CS[c](:[*]):[*]
5. Unknown ECFP_2 feature: -1841325949: [*]:[c](:[*])C(C)[c](:[*]):[*]
6. Unknown ECFP_2 feature: 1092541557: [*]C([*])[c]1:o:[*]:[*]:n:1

Feature Contribution

Top features for positive contribution

Fingerprint	Bit/Smiles	Feature Structure	Score	Carcinogen in training set
ECFP_6	769925792	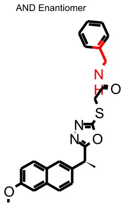 [*]NC[c](:[*]):[*]	0.617	2 out of 2

ECFP_6	-830332112	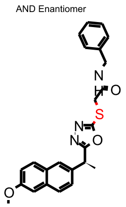<br>[*]S[*]	0.546	5 out of 8
ECFP_6	-178525456	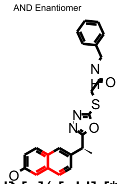<br>[*]:[cH]:[c](:[cH]:[*])):[c](:[*]):[*]	0.457	4 out of 7

Top Features for negative contribution

Fingerprint	Bit/Smiles	Feature Structure	Score	Carcinogen in training set
ECFP_6	1731843802	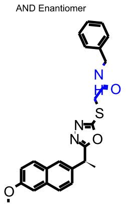<br>[*]CC(=O)N[*]	-0.657	0 out of 3
ECFP_6	1307307440	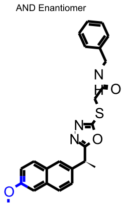<br>[*]:[c](:[*])OC	-0.558	4 out of 25
ECFP_6	864909220	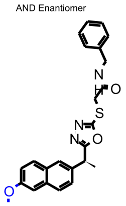<br>[*]OC	-0.466	7 out of 38

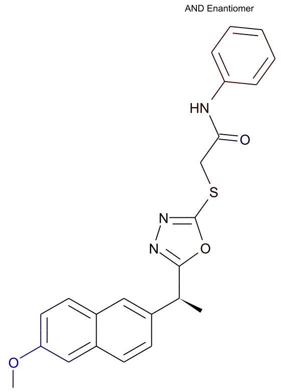

$C_{23}H_{21}N_3O_3S$
Molecular Weight: 419.49614
ALogP: 4.238
Rotatable Bonds: 7
Acceptors: 5
Donors: 1

Model Prediction

Prediction: Non-Carcinogen
Probability: 0.245
Enrichment: 0.765
Bayesian Score: -0.989
Mahalanobis Distance: 11.820
Mahalanobis Distance p-value: 0.0172

Prediction: Positive if the Bayesian score is above the estimated best cutoff value from minimizing the false positive and false negative rate.
Probability: The esimated probability that the sample is in the positive category. This assumes that the Bayesian score follows a normal distribution and is different from the prediction using a cutoff.
Enrichment: An estimate of enrichment, that is, the increased likelihood (versus random) of this sample being in the category.
Bayesian Score: The standard Laplacian-modified Bayesian score.
Mahalanobis Distance: The Mahalanobis distance (MD) is the distance to the center of the training data. The larger the MD, the less trustworthy the prediction.
Mahalanobis Distance p-value: The p-value gives the fraction of training data with an MD greater than or equal to the one for the given sample, assuming normally distributed data. The smaller the p-value, the less trustworthy the prediciton. For highly non-normal X properties (e.g., fingerprints), the MD p-value is wildly inaccurate.

Structural Similar Compounds

Name	Lovastatin	Simvastatin	Moricizine
Structure	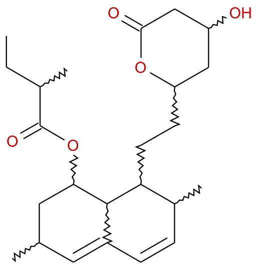	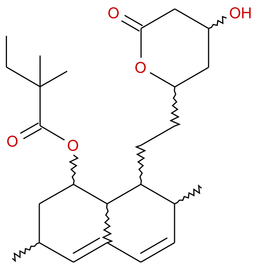	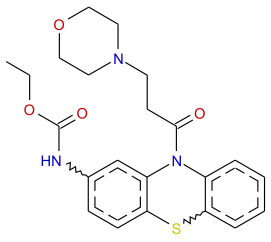
Actual Endpoint	Carcinogen	Carcinogen	Carcinogen
Predicted Endpoint	Carcinogen	Carcinogen	Carcinogen
Distance	0.560	0.584	0.595
Reference	US FDA (Centre for Drug Eval.& Res./Off. Testing & Res.) Sept. 1997	US FDA (Centre for Drug Eval.& Res./Off. Testing & Res.) Sept. 1997	US FDA (Centre for Drug Eval.& Res./Off. Testing & Res.) Sept. 1997

Model Applicability

Unknown features are fingerprint features in the query molecule, but not found in the training set.

1. All properties and OPS components are within expected ranges.
2. Unknown ECFP_2 feature: -955816473: [*]SCC(=[*])[*]
3. Unknown ECFP_2 feature: 1093109320: [*]S[c]1:o:[*]:[*]:n:1
4. Unknown ECFP_2 feature: 1427820655: [*]CS[c](:[*]):[*]
5. Unknown ECFP_2 feature: -1841325949: [*]:[c](:[*])C(C)[c](:[*]):[*]
6. Unknown ECFP_2 feature: 1092541557: [*]C([*])[c]1:o:[*]:[*]:n:1

Feature Contribution

Top features for positive contribution

Fingerprint	Bit/Smiles	Feature Structure	Score	Carcinogen in training set
ECFP_6	738938915	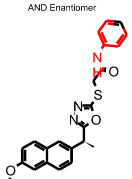 [*]C(=[*])N[c]1:[cH]:[cH]:[*]:[cH]:[cH]:1	0.617	2 out of 2

ECFP_6	-830332112	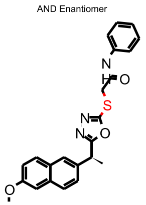 [*]S[*]	0.546	5 out of 8
ECFP_6	-178525456	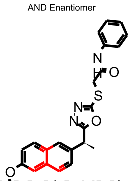 [*]:[cH]:[c](:[cH]:[*]):[c](:[*]):[*]	0.457	4 out of 7
**Top Features for negative contribution**				
**Fingerprint**	**Bit/Smiles**	**Feature Structure**	**Score**	**Carcinogen in training set**
ECFP_6	1731843802	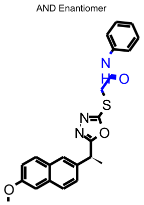 [*]CC(=O)N[*]	-0.657	0 out of 3
ECFP_6	1307307440	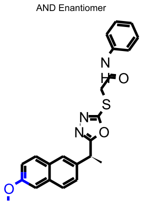 [*]:[c](:[*])OC	-0.558	4 out of 25
ECFP_6	864909220	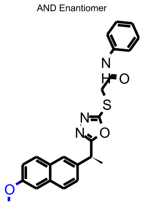 [*]OC	-0.466	7 out of 38

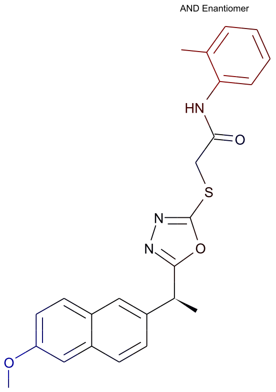

$C_{24}H_{23}N_3O_3S$
Molecular Weight: 433.52272
ALogP: 4.724
Rotatable Bonds: 7
Acceptors: 5
Donors: 1

Model Prediction

Prediction: Carcinogen
Probability: 0.270
Enrichment: 0.842
Bayesian Score: 0.336
Mahalanobis Distance: 11.979
Mahalanobis Distance p-value: 0.0114

Prediction: Positive if the Bayesian score is above the estimated best cutoff value from minimizing the false positive and false negative rate.
Probability: The esimated probability that the sample is in the positive category. This assumes that the Bayesian score follows a normal distribution and is different from the prediction using a cutoff.
Enrichment: An estimate of enrichment, that is, the increased likelihood (versus random) of this sample being in the category.
Bayesian Score: The standard Laplacian-modified Bayesian score.
Mahalanobis Distance: The Mahalanobis distance (MD) is the distance to the center of the training data. The larger the MD, the less trustworthy the prediction.
Mahalanobis Distance p-value: The p-value gives the fraction of training data with an MD greater than or equal to the one for the given sample, assuming normally distributed data. The smaller the p-value, the less trustworthy the prediciton. For highly non-normal X properties (e.g., fingerprints), the MD p-value is wildly inaccurate.

Structural Similar Compounds

Name	Simvastatin	Lovastatin	Felodipine
Structure	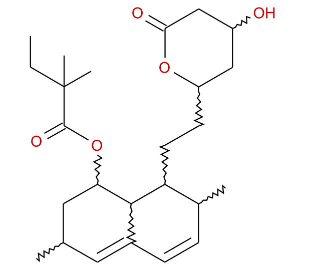	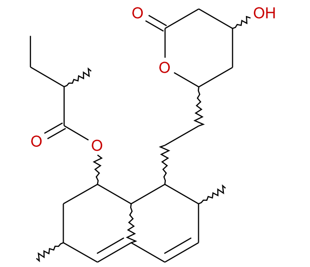	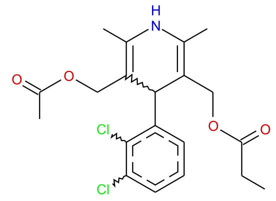
Actual Endpoint	Carcinogen	Carcinogen	Non-Carcinogen
Predicted Endpoint	Carcinogen	Carcinogen	Non-Carcinogen
Distance	0.567	0.568	0.613
Reference	US FDA (Centre for Drug Eval.& Res./Off. Testing & Res.) Sept. 1997	US FDA (Centre for Drug Eval.& Res./Off. Testing & Res.) Sept. 1997	US FDA (Centre for Drug Eval.& Res./Off. Testing & Res.) Sept. 1997

Model Applicability

Unknown features are fingerprint features in the query molecule, but not found in the training set.

1. All properties and OPS components are within expected ranges.
2. Unknown ECFP_2 feature: -955816473: [*]SCC(=[*])[*]
3. Unknown ECFP_2 feature: 1093109320: [*]S[c]1:o:[*]:[*]:n:1
4. Unknown ECFP_2 feature: 1427820655: [*]CS[c](:[*]):[*]
5. Unknown ECFP_2 feature: -1841325949: [*]:[c](:[*])C(C)[c](:[*]):[*]
6. Unknown ECFP_2 feature: 1092541557: [*]C([*])[c]1:o:[*]:[*]:n:1

Feature Contribution

Top features for positive contribution

Fingerprint	Bit/Smiles	Feature Structure	Score	Carcinogen in training set
ECFP_6	-830332112	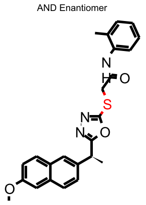 [*]S[*]	0.546	5 out of 8

ECFP_6	-178525456	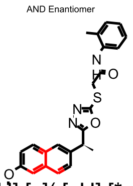 [*]:[cH]:[c](:[cH]:[*]):[c](:[*]):[*]	0.457	4 out of 7
ECFP_6	-907895376	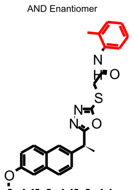 [*][c]1:[cH]:[cH]:[cH]:[cH]:[c]:1C	0.451	3 out of 5
**Top Features for negative contribution**				
**Fingerprint**	**Bit/Smiles**	**Feature Structure**	**Score**	**Carcinogen in training set**
ECFP_6	1731843802	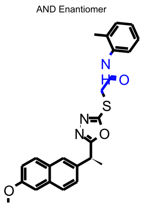 [*]CC(=O)N[*]	-0.657	0 out of 3
ECFP_6	1307307440	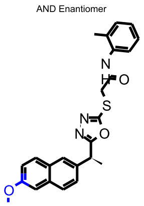 [*]:[c](:[*])OC	-0.558	4 out of 25
ECFP_6	864909220	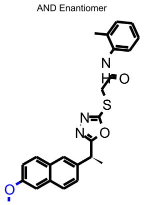 [*]OC	-0.466	7 out of 38

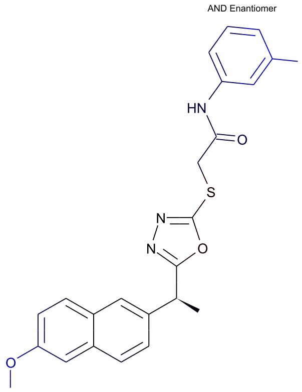

$C_{24}H_{23}N_3O_3S$
Molecular Weight: 433.52272
ALogP: 4.724
Rotatable Bonds: 7
Acceptors: 5
Donors: 1

Model Prediction

Prediction: Non-Carcinogen
Probability: 0.205
Enrichment: 0.639
Bayesian Score: -5.896
Mahalanobis Distance: 12.220
Mahalanobis Distance p-value: 0.0059

Prediction: Positive if the Bayesian score is above the estimated best cutoff value from minimizing the false positive and false negative rate.
Probability: The esimated probability that the sample is in the positive category. This assumes that the Bayesian score follows a normal distribution and is different from the prediction using a cutoff.
Enrichment: An estimate of enrichment, that is, the increased likelihood (versus random) of this sample being in the category.
Bayesian Score: The standard Laplacian-modified Bayesian score.
Mahalanobis Distance: The Mahalanobis distance (MD) is the distance to the center of the training data. The larger the MD, the less trustworthy the prediction.
Mahalanobis Distance p-value: The p-value gives the fraction of training data with an MD greater than or equal to the one for the given sample, assuming normally distributed data. The smaller the p-value, the less trustworthy the prediciton. For highly non-normal X properties (e.g., fingerprints), the MD p-value is wildly inaccurate.

Structural Similar Compounds

Name	Simvastatin	Lovastatin	Felodipine
Structure	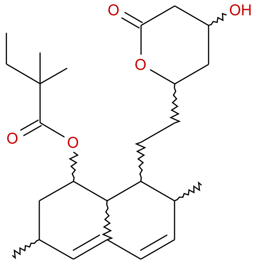	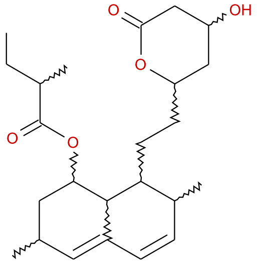	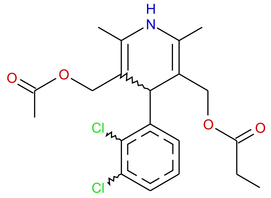
Actual Endpoint	Carcinogen	Carcinogen	Non-Carcinogen
Predicted Endpoint	Carcinogen	Carcinogen	Non-Carcinogen
Distance	0.567	0.568	0.609
Reference	US FDA (Centre for Drug Eval.& Res./Off. Testing & Res.) Sept. 1997	US FDA (Centre for Drug Eval.& Res./Off. Testing & Res.) Sept. 1997	US FDA (Centre for Drug Eval.& Res./Off. Testing & Res.) Sept. 1997

Model Applicability

Unknown features are fingerprint features in the query molecule, but not found in the training set.

1. All properties and OPS components are within expected ranges.
2. Unknown ECFP_2 feature: -955816473: [*]SCC(=[*])[*]
3. Unknown ECFP_2 feature: 1093109320: [*]S[c]1:o:[*]:[*]:n:1
4. Unknown ECFP_2 feature: 1427820655: [*]CS[c](:[*]):[*]
5. Unknown ECFP_2 feature: -1841325949: [*]:[c](:[*])C(C)[c](:[*]):[*]
6. Unknown ECFP_2 feature: 1092541557: [*]C([*])[c]1:o:[*]:[*]:n:1

Feature Contribution

Top features for positive contribution				
**Fingerprint**	**Bit/Smiles**	**Feature Structure**	**Score**	**Carcinogen in training set**
ECFP_6	-830332112	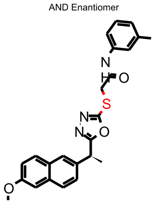 [*]S[*]	0.546	5 out of 8

ECFP_6	-178525456	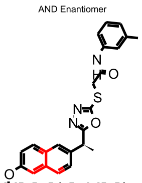<br>[*]:[cH]:[c](:[cH]:[*<br>]):[c](:[*]):[*]	0.457	4 out of 7
ECFP_6	710652510	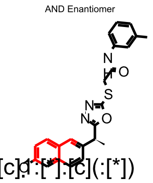<br>[*][c]1:[*]:[c](:[*])<br>:[c](:[cH]:[*]):[cH]<br>:[cH]:1	0.442	2 out of 3
**Top Features for negative contribution**				
**Fingerprint**	**Bit/Smiles**	**Feature Structure**	**Score**	**Carcinogen in training set**
ECFP_6	-179515162	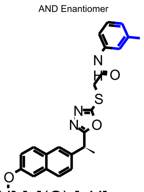<br>[*]:[cH]:[c](C):[cH]:<br>[*]	-1.409	0 out of 10
ECFP_6	1731843802	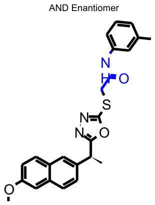<br>[*]CC(=O)N[*]	-0.657	0 out of 3
ECFP_6	-317125107	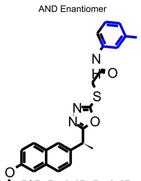<br>[*][c]1:[*]:[cH]:[cH]<br>:[c](C):[cH]:1	-0.657	0 out of 3

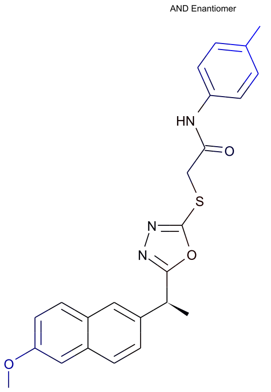

$C_{24}H_{23}N_3O_3S$
Molecular Weight: 433.52272
ALogP: 4.724
Rotatable Bonds: 7
Acceptors: 5
Donors: 1

Model Prediction

Prediction: Non-Carcinogen
Probability: 0.205
Enrichment: 0.639
Bayesian Score: -5.943
Mahalanobis Distance: 11.534
Mahalanobis Distance p-value: 0.0342

Prediction: Positive if the Bayesian score is above the estimated best cutoff value from minimizing the false positive and false negative rate.
Probability: The esimated probability that the sample is in the positive category. This assumes that the Bayesian score follows a normal distribution and is different from the prediction using a cutoff.
Enrichment: An estimate of enrichment, that is, the increased likelihood (versus random) of this sample being in the category.
Bayesian Score: The standard Laplacian-modified Bayesian score.
Mahalanobis Distance: The Mahalanobis distance (MD) is the distance to the center of the training data. The larger the MD, the less trustworthy the prediction.
Mahalanobis Distance p-value: The p-value gives the fraction of training data with an MD greater than or equal to the one for the given sample, assuming normally distributed data. The smaller the p-value, the less trustworthy the prediciton. For highly non-normal X properties (e.g., fingerprints), the MD p-value is wildly inaccurate.

Structural Similar Compounds

Name	Simvastatin	Lovastatin	Felodipine
Structure	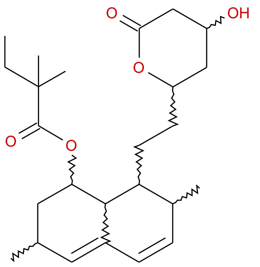	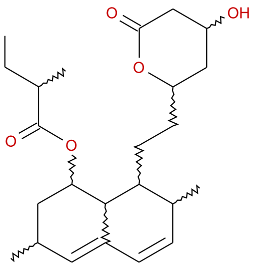	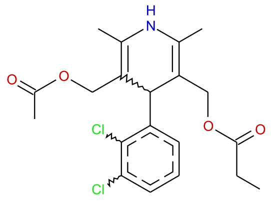
Actual Endpoint	Carcinogen	Carcinogen	Non-Carcinogen
Predicted Endpoint	Carcinogen	Carcinogen	Non-Carcinogen
Distance	0.566	0.567	0.616
Reference	US FDA (Centre for Drug Eval.& Res./Off. Testing & Res.) Sept. 1997	US FDA (Centre for Drug Eval.& Res./Off. Testing & Res.) Sept. 1997	US FDA (Centre for Drug Eval.& Res./Off. Testing & Res.) Sept. 1997

Model Applicability

Unknown features are fingerprint features in the query molecule, but not found in the training set.

1. All properties and OPS components are within expected ranges.
2. Unknown ECFP_2 feature: -955816473: [*]SCC(=[*])[*]
3. Unknown ECFP_2 feature: 1093109320: [*]S[c]1:o:[*]:[*]:n:1
4. Unknown ECFP_2 feature: 1427820655: [*]CS[c](:[*]):[*]
5. Unknown ECFP_2 feature: -1841325949: [*]:[c](:[*])C(C)[c](:[*]):[*]
6. Unknown ECFP_2 feature: 1092541557: [*]C([*])[c]1:o:[*]:[*]:n:1

Feature Contribution

Top features for positive contribution				
Fingerprint	Bit/Smiles	Feature Structure	Score	Carcinogen in training set
ECFP_6	738938915	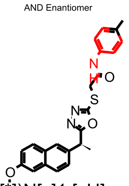 [*]C(=[*])N[c]1:[cH]:[cH]:[*]:[cH]:[cH]:1	0.617	2 out of 2

ECFP_6	-830332112	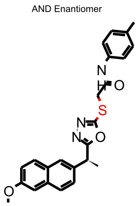 [*]S[*]	0.546	5 out of 8
ECFP_6	-178525456	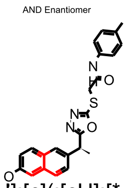 [*]:[cH]:[c](:[cH]:[*]):[c](:[*]):[*]	0.457	4 out of 7
**Top Features for negative contribution**				
**Fingerprint**	**Bit/Smiles**	**Feature Structure**	**Score**	**Carcinogen in training set**
ECFP_6	-179515162	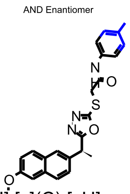 [*]:[cH]:[c](C):[cH]:[*]	-1.409	0 out of 10
ECFP_6	-210573707	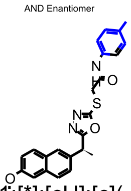 [*][c]1:[*]:[cH]:[c](C):[cH]:[cH]:1	-1.246	0 out of 8
ECFP_6	-533780882	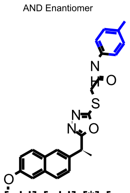 C[c]1:[cH]:[cH]:[*]:[cH]:[cH]:1	-1.049	0 out of 6

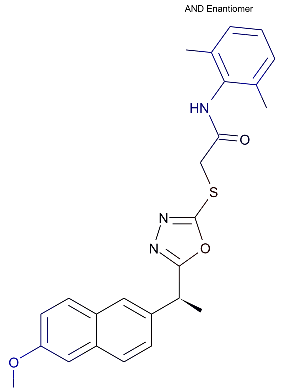

$C_{25}H_{25}N_3O_3S$
Molecular Weight: 447.5493
ALogP: 5.21
Rotatable Bonds: 7
Acceptors: 5
Donors: 1

Model Prediction

Prediction: Non-Carcinogen
Probability: 0.207
Enrichment: 0.646
Bayesian Score: -4.980
Mahalanobis Distance: 12.255
Mahalanobis Distance p-value: 0.00534

Prediction: Positive if the Bayesian score is above the estimated best cutoff value from minimizing the false positive and false negative rate.
Probability: The esimated probability that the sample is in the positive category. This assumes that the Bayesian score follows a normal distribution and is different from the prediction using a cutoff.
Enrichment: An estimate of enrichment, that is, the increased likelihood (versus random) of this sample being in the category.
Bayesian Score: The standard Laplacian-modified Bayesian score.
Mahalanobis Distance: The Mahalanobis distance (MD) is the distance to the center of the training data. The larger the MD, the less trustworthy the prediction.
Mahalanobis Distance p-value: The p-value gives the fraction of training data with an MD greater than or equal to the one for the given sample, assuming normally distributed data. The smaller the p-value, the less trustworthy the prediciton. For highly non-normal X properties (e.g., fingerprints), the MD p-value is wildly inaccurate.

Structural Similar Compounds

Name	Simvastatin	Lovastatin	Felodipine
Structure	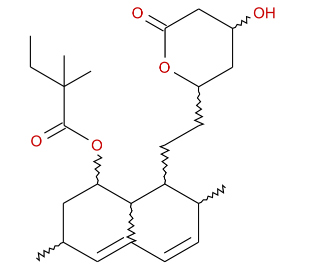	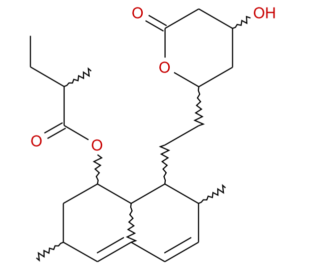	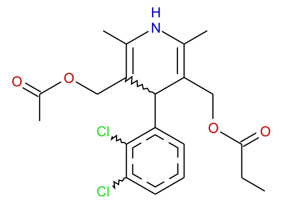
Actual Endpoint	Carcinogen	Carcinogen	Non-Carcinogen
Predicted Endpoint	Carcinogen	Carcinogen	Non-Carcinogen
Distance	0.578	0.598	0.638
Reference	US FDA (Centre for Drug Eval.& Res./Off. Testing & Res.) Sept. 1997	US FDA (Centre for Drug Eval.& Res./Off. Testing & Res.) Sept. 1997	US FDA (Centre for Drug Eval.& Res./Off. Testing & Res.) Sept. 1997

Model Applicability

Unknown features are fingerprint features in the query molecule, but not found in the training set.

1. All properties and OPS components are within expected ranges.
2. Unknown ECFP_2 feature: -955816473: [*]SCC(=[*])[*]
3. Unknown ECFP_2 feature: 1093109320: [*]S[c]1:o:[*]:[*]:n:1
4. Unknown ECFP_2 feature: 1427820655: [*]CS[c](:[*]):[*]
5. Unknown ECFP_2 feature: -1841325949: [*]:[c](:[*])C(C)[c](:[*]):[*]
6. Unknown ECFP_2 feature: 1092541557: [*]C([*])[c]1:o:[*]:[*]:n:1

Feature Contribution

Top features for positive contribution

Fingerprint	Bit/Smiles	Feature Structure	Score	Carcinogen in training set
ECFP_6	-830332112	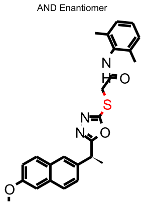 [*]S[*]	0.546	5 out of 8

ECFP_6	-178525456	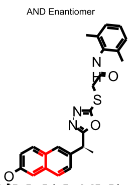 [*]:[cH]:[c](:[cH]:[*]):[c](:[*]):[*]	0.457	4 out of 7
ECFP_6	710652510	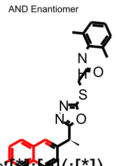 [*][c]1:[*]:[c](:[*]) :[c](:[cH]:[*]):[cH] :[cH]:1	0.442	2 out of 3

Top Features for negative contribution

Fingerprint	Bit/Smiles	Feature Structure	Score	Carcinogen in training set
ECFP_6	1731843802	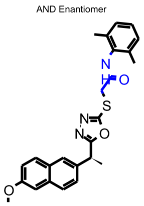 [*]CC(=O)N[*]	-0.657	0 out of 3
ECFP_6	2007300961	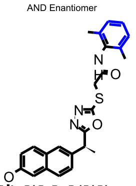 [*][c]1:[*]:[c]([*]): [cH]:[cH]:[cH]:1	-0.652	5 out of 34
ECFP_6	1307307440	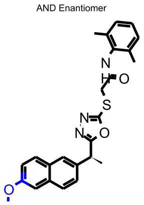 [*]:[c](:[*])OC	-0.558	4 out of 25

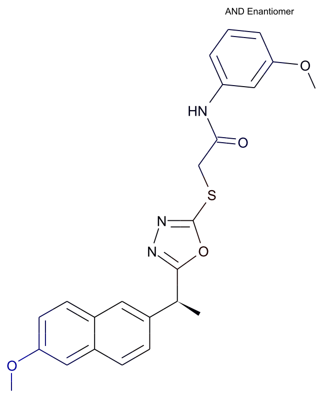

$C_{24}H_{23}N_3O_4S$
Molecular Weight: 449.52212
ALogP: 4.221
Rotatable Bonds: 8
Acceptors: 6
Donors: 1

Structural Similar Compounds

Name	Moricizine	Felodipine	Lovastatin
Structure	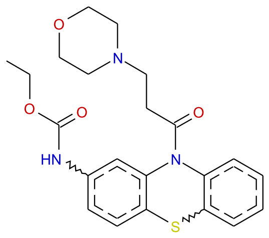	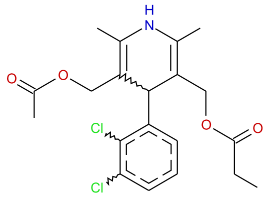	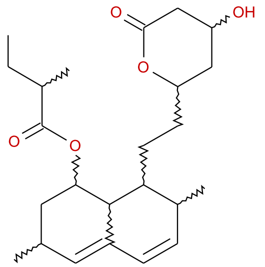
Actual Endpoint	Carcinogen	Non-Carcinogen	Carcinogen
Predicted Endpoint	Carcinogen	Non-Carcinogen	Carcinogen
Distance	0.613	0.622	0.624
Reference	US FDA (Centre for Drug Eval.& Res./Off. Testing & Res.) Sept. 1997	US FDA (Centre for Drug Eval.& Res./Off. Testing & Res.) Sept. 1997	US FDA (Centre for Drug Eval.& Res./Off. Testing & Res.) Sept. 1997

Model Prediction

Prediction: Non-Carcinogen
Probability: 0.214
Enrichment: 0.666
Bayesian Score: -3.754
Mahalanobis Distance: 12.133
Mahalanobis Distance p-value: 0.00751

Prediction: Positive if the Bayesian score is above the estimated best cutoff value from minimizing the false positive and false negative rate.
Probability: The esimated probability that the sample is in the positive category. This assumes that the Bayesian score follows a normal distribution and is different from the prediction using a cutoff.
Enrichment: An estimate of enrichment, that is, the increased likelihood (versus random) of this sample being in the category.
Bayesian Score: The standard Laplacian-modified Bayesian score.
Mahalanobis Distance: The Mahalanobis distance (MD) is the distance to the center of the training data. The larger the MD, the less trustworthy the prediction.
Mahalanobis Distance p-value: The p-value gives the fraction of training data with an MD greater than or equal to the one for the given sample, assuming normally distributed data. The smaller the p-value, the less trustworthy the prediciton. For highly non-normal X properties (e.g., fingerprints), the MD p-value is wildly inaccurate.

Model Applicability

Unknown features are fingerprint features in the query molecule, but not found in the training set.

1. All properties and OPS components are within expected ranges.
2. Unknown ECFP_2 feature: -955816473: [*]SCC(=[*])[*]
3. Unknown ECFP_2 feature: 1093109320: [*]S[c]1:o:[*]:[*]:n:1
4. Unknown ECFP_2 feature: 1427820655: [*]CS[c](:[*]):[*]
5. Unknown ECFP_2 feature: -1841325949: [*]:[c](:[*])C(C)[c](:[*]):[*]
6. Unknown ECFP_2 feature: 1092541557: [*]C([*])[c]1:o:[*]:[*]:n:1

Feature Contribution

Top features for positive contribution				
**Fingerprint**	**Bit/Smiles**	**Feature Structure**	**Score**	**Carcinogen in training set**
ECFP_6	-830332112	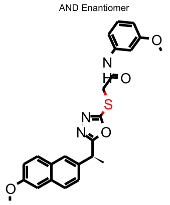 [*]S[*]	0.546	5 out of 8

ECFP_6	-178525456	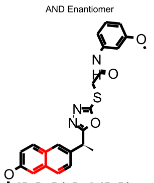<br>[*]:[cH]:[c](:[cH]:[*]):[c](:[*]):[*]	0.457	4 out of 7
ECFP_6	710652510	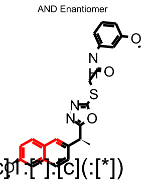<br>[*][c]1:[*]:[c](:[*]):[c](:[cH]:[*]):[cH]:[cH]:1	0.442	2 out of 3

Top Features for negative contribution

Fingerprint	Bit/Smiles	Feature Structure	Score	Carcinogen in training set
ECFP_6	1731843802	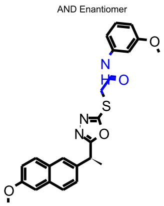<br>[*]CC(=O)N[*]	-0.657	0 out of 3
ECFP_6	2007300961	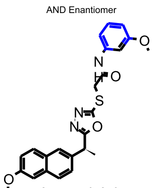<br>[*][c]1:[*]:[c]([*]):[cH]:[cH]:[cH]:1	-0.652	5 out of 34
ECFP_6	1307307440	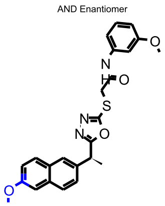<br>[*]:[c](:[*])OC	-0.558	4 out of 25

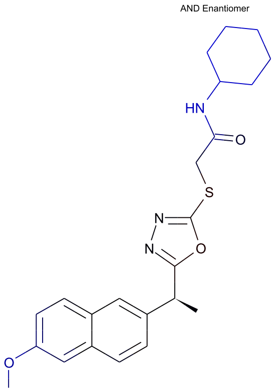

$C_{23}H_{27}N_3O_3S$
Molecular Weight: 425.54378
ALogP: 4.52
Rotatable Bonds: 7
Acceptors: 5
Donors: 1

Structural Similar Compounds

Name	Lovastatin	Simvastatin	Felodipine
Structure	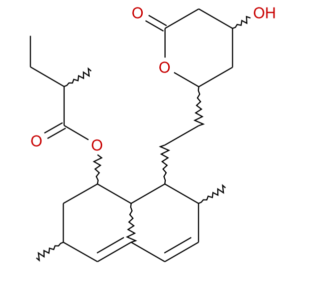	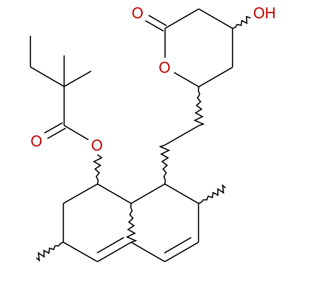	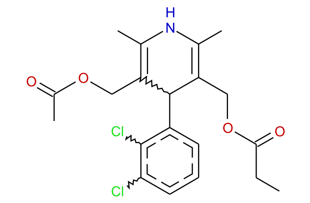
Actual Endpoint	Carcinogen	Carcinogen	Non-Carcinogen
Predicted Endpoint	Carcinogen	Carcinogen	Non-Carcinogen
Distance	0.552	0.563	0.608
Reference	US FDA (Centre for Drug Eval.& Res./Off. Testing & Res.) Sept. 1997	US FDA (Centre for Drug Eval.& Res./Off. Testing & Res.) Sept. 1997	US FDA (Centre for Drug Eval.& Res./Off. Testing & Res.) Sept. 1997

Model Prediction

Prediction: Non-Carcinogen
Probability: 0.205
Enrichment: 0.640
Bayesian Score: -6.779
Mahalanobis Distance: 12.092
Mahalanobis Distance p-value: 0.0084

Prediction: Positive if the Bayesian score is above the estimated best cutoff value from minimizing the false positive and false negative rate.
Probability: The esimated probability that the sample is in the positive category. This assumes that the Bayesian score follows a normal distribution and is different from the prediction using a cutoff.
Enrichment: An estimate of enrichment, that is, the increased likelihood (versus random) of this sample being in the category.
Bayesian Score: The standard Laplacian-modified Bayesian score.
Mahalanobis Distance: The Mahalanobis distance (MD) is the distance to the center of the training data. The larger the MD, the less trustworthy the prediction.
Mahalanobis Distance p-value: The p-value gives the fraction of training data with an MD greater than or equal to the one for the given sample, assuming normally distributed data. The smaller the p-value, the less trustworthy the prediciton. For highly non-normal X properties (e.g., fingerprints), the MD p-value is wildly inaccurate.

Model Applicability

Unknown features are fingerprint features in the query molecule, but not found in the training set.

1. OPS PC28 out of range. Value: -3.286. Training min, max, SD, explained variance: -2.8298, 3.1935, 1.043, 0.0111.
2. Unknown ECFP_2 feature: -955816473: [*]SCC(=[*])[*]
3. Unknown ECFP_2 feature: 1093109320: [*]S[c]1:o:[*]:[*]:n:1
4. Unknown ECFP_2 feature: 1427820655: [*]CS[c](:[*]):[*]
5. Unknown ECFP_2 feature: -1841325949: [*]:[c](:[*])C(C)[c](:[*]):[*]
6. Unknown ECFP_2 feature: 1092541557: [*]C([*])[c]1:o:[*]:[*]:n:1

Feature Contribution

Top features for positive contribution				
Fingerprint	Bit/Smiles	Feature Structure	Score	Carcinogen in training set

ECFP_6	-830332112	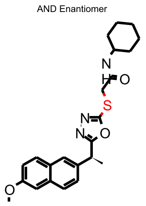 [*]S[*]	0.546	5 out of 8
ECFP_6	-178525456	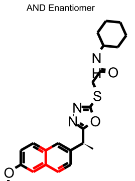 [*]:[cH]:[c](:[cH]:[*]):[c](:[*]):[*]	0.457	4 out of 7
ECFP_6	710652510	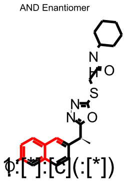 [*][c]1:[*]:[c](:[*]) :[c](:[cH]:[*]):[cH] :[cH]:1	0.442	2 out of 3
**Top Features for negative contribution**				
**Fingerprint**	**Bit/Smiles**	**Feature Structure**	**Score**	**Carcinogen in training set**
ECFP_6	-1607899848	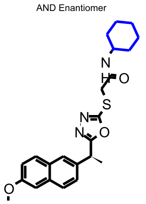 [*]C1CCCCC1	-0.805	0 out of 4
ECFP_6	662850656	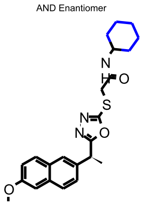 [*]1CCCCC1	-0.716	1 out of 10

ECFP_6	-676350260	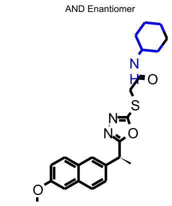 [*]NC1C[*]CCC1	-0.657	0 out of 3

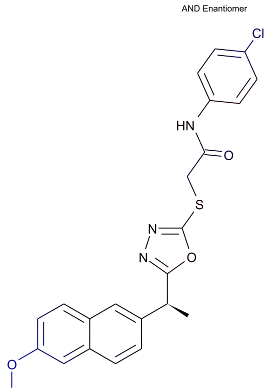

$C_{23}H_{20}ClN_3O_3S$
Molecular Weight: 453.9412
ALogP: 4.902
Rotatable Bonds: 7
Acceptors: 5
Donors: 1

Structural Similar Compounds

Name	Simvastatin	Lovastatin	Fluticasone
Structure	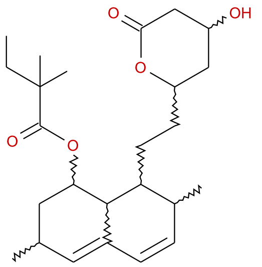	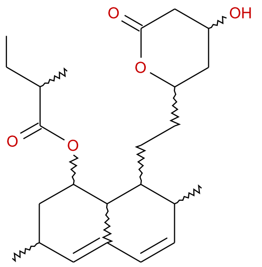	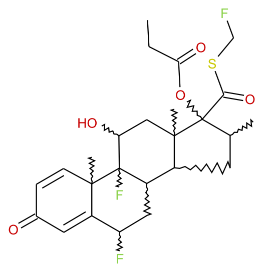
Actual Endpoint	Carcinogen	Carcinogen	Non-Carcinogen
Predicted Endpoint	Carcinogen	Carcinogen	Non-Carcinogen
Distance	0.585	0.596	0.624
Reference	US FDA (Centre for Drug Eval.& Res./Off. Testing & Res.) Sept. 1997	US FDA (Centre for Drug Eval.& Res./Off. Testing & Res.) Sept. 1997	US FDA (Centre for Drug Eval.& Res./Off. Testing & Res.) Sept. 1997

Model Prediction

Prediction: Non-Carcinogen
Probability: 0.221
Enrichment: 0.689
Bayesian Score: -2.891
Mahalanobis Distance: 13.215
Mahalanobis Distance p-value: 0.000261

Prediction: Positive if the Bayesian score is above the estimated best cutoff value from minimizing the false positive and false negative rate.
Probability: The esimated probability that the sample is in the positive category. This assumes that the Bayesian score follows a normal distribution and is different from the prediction using a cutoff.
Enrichment: An estimate of enrichment, that is, the increased likelihood (versus random) of this sample being in the category.
Bayesian Score: The standard Laplacian-modified Bayesian score.
Mahalanobis Distance: The Mahalanobis distance (MD) is the distance to the center of the training data. The larger the MD, the less trustworthy the prediction.
Mahalanobis Distance p-value: The p-value gives the fraction of training data with an MD greater than or equal to the one for the given sample, assuming normally distributed data. The smaller the p-value, the less trustworthy the prediciton. For highly non-normal X properties (e.g., fingerprints), the MD p-value is wildly inaccurate.

Model Applicability

Unknown features are fingerprint features in the query molecule, but not found in the training set.

1. OPS PC28 out of range. Value: -2.93. Training min, max, SD, explained variance: -2.8298, 3.1935, 1.043, 0.0111.
2. Unknown ECFP_2 feature: -955816473: [*]SCC(=[*])[*]
3. Unknown ECFP_2 feature: 1093109320: [*]S[c]1:o:[*]:[*]:n:1
4. Unknown ECFP_2 feature: 1427820655: [*]CS[c](:[*]):[*]
5. Unknown ECFP_2 feature: -1841325949: [*]:[c](:[*])C(C)[c](:[*]):[*]
6. Unknown ECFP_2 feature: 1092541557: [*]C([*])[c]1:o:[*]:[*]:n:1

Feature Contribution

Top features for positive contribution				
Fingerprint	Bit/Smiles	Feature Structure	Score	Carcinogen in training set

ECFP_6	738938915	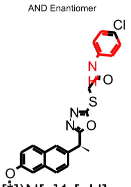 [*]C(=[*])N[c]1:[cH]:[cH]:[*]:[cH]:[cH]:1	0.617	2 out of 2
ECFP_6	-830332112	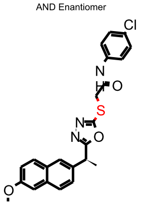 [*]S[*]	0.546	5 out of 8
ECFP_6	-178525456	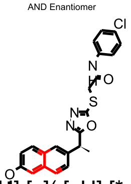 [*]:[cH]:[c](:[cH]:[*]):[c](:[*]):[*]	0.457	4 out of 7

Top Features for negative contribution

Fingerprint	Bit/Smiles	Feature Structure	Score	Carcinogen in training set
ECFP_6	1731843802	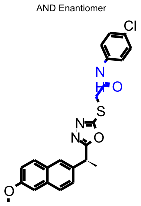 [*]CC(=O)N[*]	-0.657	0 out of 3
ECFP_6	1307307440	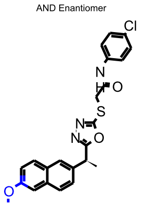 [*]:[c](:[*])OC	-0.558	4 out of 25

ECFP_6	-176494269	AND Enantiomer 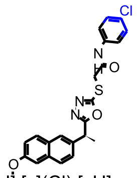 [*]:[cH]:[c](Cl):[cH]:[*]	-0.476	5 out of 28

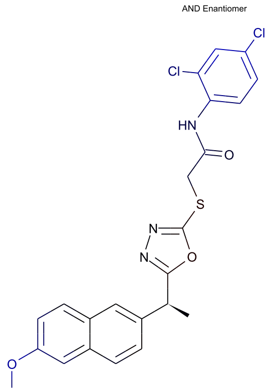

$C_{23}H_{19}Cl_2N_3O_3S$
Molecular Weight: 488.38626
ALogP: 5.567
Rotatable Bonds: 7
Acceptors: 5
Donors: 1

Structural Similar Compounds

Name	Simvastatin	Fluticasone	Emetine
Structure	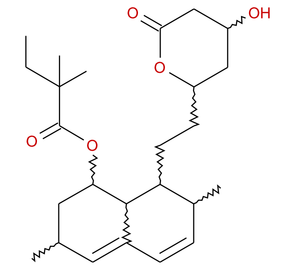	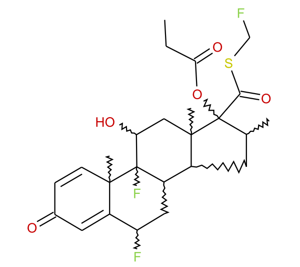	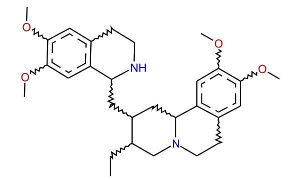
Actual Endpoint	Carcinogen	Non-Carcinogen	Non-Carcinogen
Predicted Endpoint	Carcinogen	Non-Carcinogen	Non-Carcinogen
Distance	0.639	0.645	0.658
Reference	US FDA (Centre for Drug Eval.& Res./Off. Testing & Res.) Sept. 1997	US FDA (Centre for Drug Eval.& Res./Off. Testing & Res.) Sept. 1997	US FDA (Centre for Drug Eval.& Res./Off. Testing & Res.) Sept. 1997

Model Prediction

Prediction: Non-Carcinogen
Probability: 0.205
Enrichment: 0.639
Bayesian Score: -6.708
Mahalanobis Distance: 12.616
Mahalanobis Distance p-value: 0.00184

Prediction: Positive if the Bayesian score is above the estimated best cutoff value from minimizing the false positive and false negative rate.
Probability: The esimated probability that the sample is in the positive category. This assumes that the Bayesian score follows a normal distribution and is different from the prediction using a cutoff.
Enrichment: An estimate of enrichment, that is, the increased likelihood (versus random) of this sample being in the category.
Bayesian Score: The standard Laplacian-modified Bayesian score.
Mahalanobis Distance: The Mahalanobis distance (MD) is the distance to the center of the training data. The larger the MD, the less trustworthy the prediction.
Mahalanobis Distance p-value: The p-value gives the fraction of training data with an MD greater than or equal to the one for the given sample, assuming normally distributed data. The smaller the p-value, the less trustworthy the prediciton. For highly non-normal X properties (e.g., fingerprints), the MD p-value is wildly inaccurate.

Model Applicability

Unknown features are fingerprint features in the query molecule, but not found in the training set.

1. OPS PC28 out of range. Value: -2.9066. Training min, max, SD, explained variance: -2.8298, 3.1935, 1.043, 0.0111.
2. Unknown ECFP_2 feature: -955816473: [*]SCC(=[*])[*]
3. Unknown ECFP_2 feature: 1093109320: [*]S[c]1:o:[*]:[*]:n:1
4. Unknown ECFP_2 feature: 1427820655: [*]CS[c](:[*]):[*]
5. Unknown ECFP_2 feature: -1841325949: [*]:[c](:[*])C(C)[c](:[*]):[*]
6. Unknown ECFP_2 feature: 1092541557: [*]C([*])[c]1:o:[*]:[*]:n:1

Feature Contribution

Top features for positive contribution

Fingerprint	Bit/Smiles	Feature Structure	Score	Carcinogen in training set

ECFP_6	-830332112	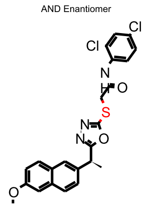<br>[*]S[*]	0.546	5 out of 8
ECFP_6	-178525456	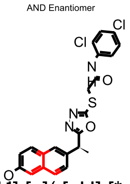<br>[*]:[cH]:[c](:[cH]:[*]):[c](:[*]):[*]	0.457	4 out of 7
ECFP_6	710652510	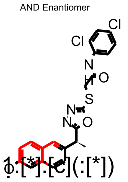<br>[*][c]1:[*]:[c](:[*]):[c](:[cH]:[*]):[cH]:[cH]:1	0.442	2 out of 3
**Top Features for negative contribution**				
**Fingerprint**	**Bit/Smiles**	**Feature Structure**	**Score**	**Carcinogen in training set**
ECFP_6	1335691903	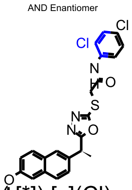<br>[*][c](:[*]):[c](Cl):[cH]:[*]	-0.669	3 out of 22
ECFP_6	1731843802	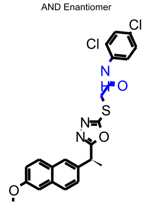<br>[*]CC(=O)N[*]	-0.657	0 out of 3

ECFP_6	577592657	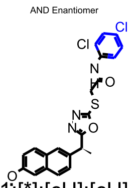 [*][c]1:[*]:[cH]:[cH]:[c](Cl):[cH]:1	-0.586	3 out of 20

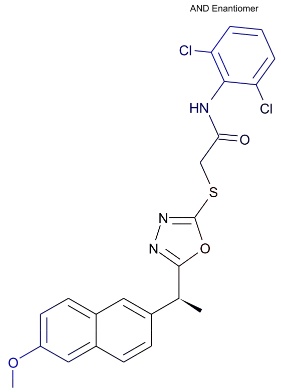

$C_{23}H_{19}Cl_2N_3O_3S$
Molecular Weight: 488.38626
ALogP: 5.567
Rotatable Bonds: 7
Acceptors: 5
Donors: 1

Model Prediction

Prediction: Non-Carcinogen
Probability: 0.205
Enrichment: 0.639
Bayesian Score: -6.361
Mahalanobis Distance: 12.878
Mahalanobis Distance p-value: 0.000803

Prediction: Positive if the Bayesian score is above the estimated best cutoff value from minimizing the false positive and false negative rate.
Probability: The esimated probability that the sample is in the positive category. This assumes that the Bayesian score follows a normal distribution and is different from the prediction using a cutoff.
Enrichment: An estimate of enrichment, that is, the increased likelihood (versus random) of this sample being in the category.
Bayesian Score: The standard Laplacian-modified Bayesian score.
Mahalanobis Distance: The Mahalanobis distance (MD) is the distance to the center of the training data. The larger the MD, the less trustworthy the prediction.
Mahalanobis Distance p-value: The p-value gives the fraction of training data with an MD greater than or equal to the one for the given sample, assuming normally distributed data. The smaller the p-value, the less trustworthy the prediciton. For highly non-normal X properties (e.g., fingerprints), the MD p-value is wildly inaccurate.

Structural Similar Compounds

Name	Simvastatin	Fluticasone	Emetine
Structure	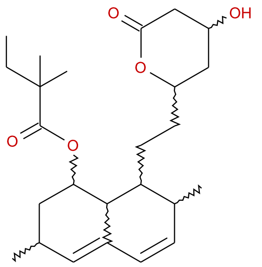	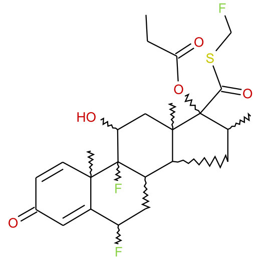	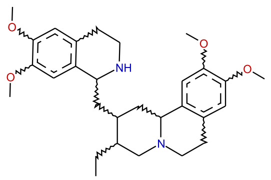
Actual Endpoint	Carcinogen	Non-Carcinogen	Non-Carcinogen
Predicted Endpoint	Carcinogen	Non-Carcinogen	Non-Carcinogen
Distance	0.638	0.645	0.657
Reference	US FDA (Centre for Drug Eval.& Res./Off. Testing & Res.) Sept. 1997	US FDA (Centre for Drug Eval.& Res./Off. Testing & Res.) Sept. 1997	US FDA (Centre for Drug Eval.& Res./Off. Testing & Res.) Sept. 1997

Model Applicability

Unknown features are fingerprint features in the query molecule, but not found in the training set.

1. All properties and OPS components are within expected ranges.
2. Unknown ECFP_2 feature: -955816473: [*]SCC(=[*])[*]
3. Unknown ECFP_2 feature: 1093109320: [*]S[c]1:o:[*]:[*]:n:1
4. Unknown ECFP_2 feature: 1427820655: [*]CS[c](:[*]):[*]
5. Unknown ECFP_2 feature: -1841325949: [*]:[c](:[*])C(C)[c](:[*]):[*]
6. Unknown ECFP_2 feature: 1092541557: [*]C([*])[c]1:o:[*]:[*]:n:1

Feature Contribution

Top features for positive contribution

Fingerprint	Bit/Smiles	Feature Structure	Score	Carcinogen in training set
ECFP_6	-830332112	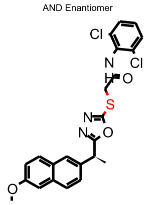 [*]S[*]	0.546	5 out of 8

ECFP_6	-178525456	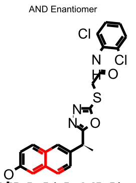 [*]:[cH]:[c](:[cH]:[*]):[c](:[*]):[*]	0.457	4 out of 7
ECFP_6	710652510	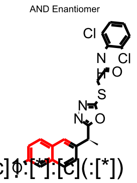 [*][c]1:[*]:[c](:[*]):[c](:[cH]:[*]):[cH]:[cH]:1	0.442	2 out of 3

Top Features for negative contribution

Fingerprint	Bit/Smiles	Feature Structure	Score	Carcinogen in training set
ECFP_6	1641317964	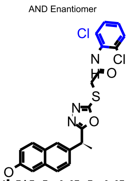 [*][c]1:[*]:[cH]:[cH]:[cH]:[c]:1Cl	-0.789	1 out of 11
ECFP_6	1335691903	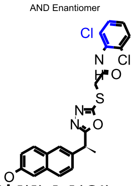 [*][c](:[*]):[c](Cl):[cH]:[*]	-0.669	3 out of 22
ECFP_6	1731843802	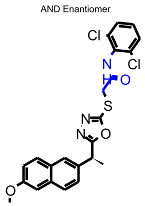 [*]CC(=O)N[*]	-0.657	0 out of 3

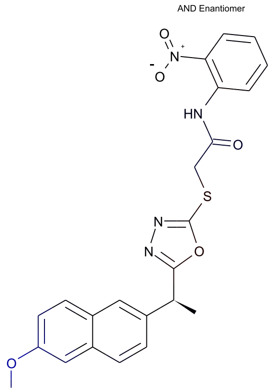

$C_{23}H_{20}N_4O_5S$
Molecular Weight: 464.4937
ALogP: 4.132
Rotatable Bonds: 8
Acceptors: 7
Donors: 1

Structural Similar Compounds

Name	Moricizine	Nisoldipine	Fluticasone
Structure	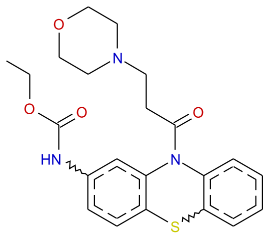	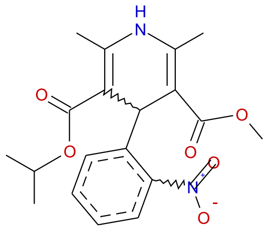	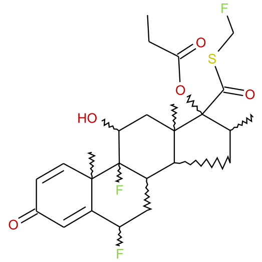
Actual Endpoint	Carcinogen	Non-Carcinogen	Non-Carcinogen
Predicted Endpoint	Carcinogen	Non-Carcinogen	Non-Carcinogen
Distance	0.678	0.684	0.685
Reference	US FDA (Centre for Drug Eval.& Res./Off. Testing & Res.) Sept. 1997	US FDA (Centre for Drug Eval.& Res./Off. Testing & Res.) Sept. 1997	US FDA (Centre for Drug Eval.& Res./Off. Testing & Res.) Sept. 1997

Model Prediction

Prediction: Non-Carcinogen
Probability: 0.215
Enrichment: 0.671
Bayesian Score: -3.553
Mahalanobis Distance: 12.191
Mahalanobis Distance p-value: 0.0064

Prediction: Positive if the Bayesian score is above the estimated best cutoff value from minimizing the false positive and false negative rate.
Probability: The esimated probability that the sample is in the positive category. This assumes that the Bayesian score follows a normal distribution and is different from the prediction using a cutoff.
Enrichment: An estimate of enrichment, that is, the increased likelihood (versus random) of this sample being in the category.
Bayesian Score: The standard Laplacian-modified Bayesian score.
Mahalanobis Distance: The Mahalanobis distance (MD) is the distance to the center of the training data. The larger the MD, the less trustworthy the prediction.
Mahalanobis Distance p-value: The p-value gives the fraction of training data with an MD greater than or equal to the one for the given sample, assuming normally distributed data. The smaller the p-value, the less trustworthy the prediciton. For highly non-normal X properties (e.g., fingerprints), the MD p-value is wildly inaccurate.

Model Applicability

Unknown features are fingerprint features in the query molecule, but not found in the training set.

1. All properties and OPS components are within expected ranges.
2. Unknown ECFP_2 feature: 1043790491: [*][N+](=[*])[*]
3. Unknown ECFP_2 feature: 781519895: [*][O-]
4. Unknown ECFP_2 feature: -955816473: [*]SCC(=[*])[*]
5. Unknown ECFP_2 feature: 1093109320: [*]S[c]1:o:[*]:[*]:n:1
6. Unknown ECFP_2 feature: 1427820655: [*]CS[c](:[*]):[*]
7. Unknown ECFP_2 feature: -1841325949: [*]:[c](:[*])C(C)[c](:[*]):[*]
8. Unknown ECFP_2 feature: 1092541557: [*]C([*])[c]1:o:[*]:[*]:n:1
9. Unknown ECFP_2 feature: -1956535100: [*][c](:[*]):[c]([N+](=[*])[*]):c:[*]
10. Unknown ECFP_2 feature: -215026467: [*]:[c](:[*])[N+](=O)[O-]
11. Unknown ECFP_2 feature: 2104376220: [*][N+](=O)[*]
12. Unknown ECFP_2 feature: -659271057: [*][N+](=[*])[O-]

Feature Contribution

Top features for positive contribution

Fingerprint	Bit/Smiles	Feature Structure	Score	Carcinogen in training set

ECFP_6	-830332112	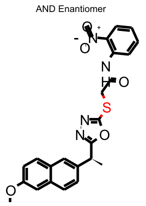 [*]S[*]	0.546	5 out of 8
ECFP_6	-178525456	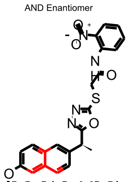 [*]:[cH]:[c](:[cH]:[*]):[c](:[*]):[*]	0.457	4 out of 7
ECFP_6	710652510	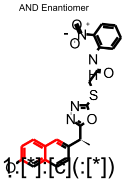 [*][c]1:[*]:[*]:[c](:[*]):[c](:[cH]:[*]):[cH]:[cH]:1	0.442	2 out of 3

Top Features for negative contribution

Fingerprint	Bit/Smiles	Feature Structure	Score	Carcinogen in training set
ECFP_6	1731843802	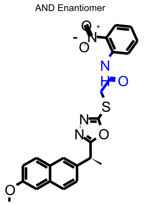 [*]CC(=O)N[*]	-0.657	0 out of 3
ECFP_6	1307307440	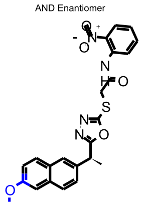 [*]:[c](:[*])OC	-0.558	4 out of 25

ECFP_6	864909220	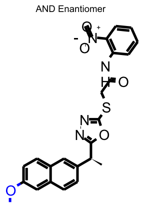 [*]OC	-0.466	7 out of 38

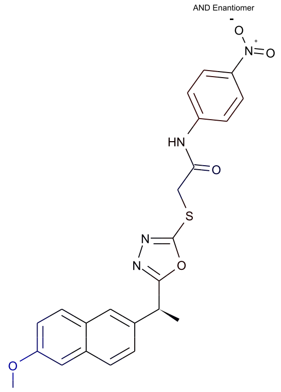

$C_{23}H_{20}N_4O_5S$
Molecular Weight: 464.4937
ALogP: 4.132
Rotatable Bonds: 8
Acceptors: 7
Donors: 1

Model Prediction

Prediction: Non-Carcinogen
Probability: 0.223
Enrichment: 0.695
Bayesian Score: -2.697
Mahalanobis Distance: 11.651
Mahalanobis Distance p-value: 0.026

Prediction: Positive if the Bayesian score is above the estimated best cutoff value from minimizing the false positive and false negative rate.
Probability: The esimated probability that the sample is in the positive category. This assumes that the Bayesian score follows a normal distribution and is different from the prediction using a cutoff.
Enrichment: An estimate of enrichment, that is, the increased likelihood (versus random) of this sample being in the category.
Bayesian Score: The standard Laplacian-modified Bayesian score.
Mahalanobis Distance: The Mahalanobis distance (MD) is the distance to the center of the training data. The larger the MD, the less trustworthy the prediction.
Mahalanobis Distance p-value: The p-value gives the fraction of training data with an MD greater than or equal to the one for the given sample, assuming normally distributed data. The smaller the p-value, the less trustworthy the prediciton. For highly non-normal X properties (e.g., fingerprints), the MD p-value is wildly inaccurate.

Structural Similar Compounds

Name	Moricizine	Fluticasone	Nisoldipine
Structure	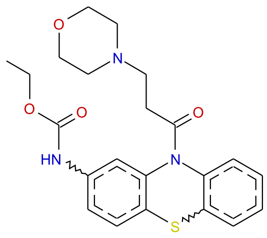	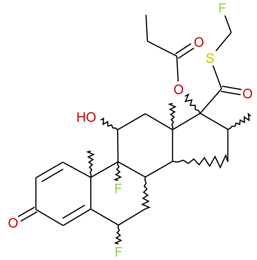	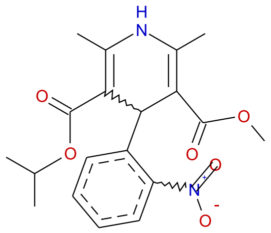
Actual Endpoint	Carcinogen	Non-Carcinogen	Non-Carcinogen
Predicted Endpoint	Carcinogen	Non-Carcinogen	Non-Carcinogen
Distance	0.676	0.684	0.691
Reference	US FDA (Centre for Drug Eval.& Res./Off. Testing & Res.) Sept. 1997	US FDA (Centre for Drug Eval.& Res./Off. Testing & Res.) Sept. 1997	US FDA (Centre for Drug Eval.& Res./Off. Testing & Res.) Sept. 1997

Model Applicability

Unknown features are fingerprint features in the query molecule, but not found in the training set.

1. All properties and OPS components are within expected ranges.
2. Unknown ECFP_2 feature: 1043790491: [*][N+](=[*])[*]
3. Unknown ECFP_2 feature: 781519895: [*][O-]
4. Unknown ECFP_2 feature: -955816473: [*]SCC(=[*])[*]
5. Unknown ECFP_2 feature: 1093109320: [*]S[c]1:o:[*]:[*]:n:1
6. Unknown ECFP_2 feature: 1427820655: [*]CS[c](:[*]):[*]
7. Unknown ECFP_2 feature: -1841325949: [*]:[c](:[*])C(C)[c](:[*]):[*]
8. Unknown ECFP_2 feature: 1092541557: [*]C([*])[c]1:o:[*]:[*]:n:1
9. Unknown ECFP_2 feature: -179073144: [*][N+](=[*])[c](:c:[*]):c:[*]
10. Unknown ECFP_2 feature: -215026467: [*]:[c](:[*])[N+](=O)[O-]
11. Unknown ECFP_2 feature: 2104376220: [*][N+](=O)[*]
12. Unknown ECFP_2 feature: -659271057: [*][N+](=[*])[O-]

Feature Contribution

Top features for positive contribution				
**Fingerprint**	**Bit/Smiles**	**Feature Structure**	**Score**	**Carcinogen in training set**

ECFP_6	738938915	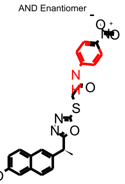 [*]C(=[*])N[c]1:[cH]:[cH]:[*]:[cH]:[cH]:1	0.617	2 out of 2
ECFP_6	-830332112	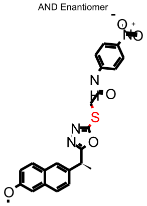 [*]S[*]	0.546	5 out of 8
ECFP_6	-178525456	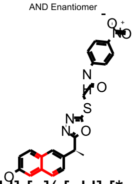 [*]:[cH]:[c](:[cH]:[*]):[c](:[*]):[*]	0.457	4 out of 7

Top Features for negative contribution

Fingerprint	Bit/Smiles	Feature Structure	Score	Carcinogen in training set
ECFP_6	1731843802	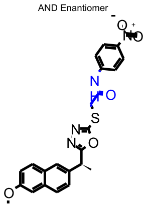 [*]CC(=O)N[*]	-0.657	0 out of 3
ECFP_6	1307307440	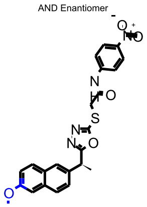 [*]:[c](:[*])OC	-0.558	4 out of 25

ECFP_6	864909220	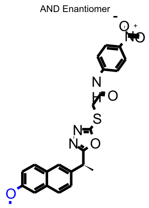 [*]OC	-0.466	7 out of 38

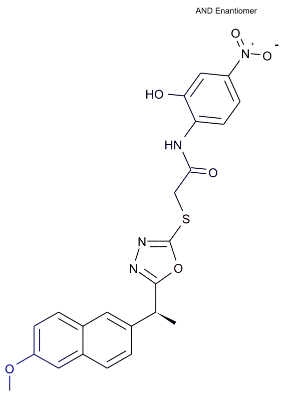

$C_{23}H_{20}N_4O_6S$
Molecular Weight: 480.4931
ALogP: 3.89
Rotatable Bonds: 8
Acceptors: 8
Donors: 2

Model Prediction

Prediction: Non-Carcinogen
Probability: 0.209
Enrichment: 0.652
Bayesian Score: -4.526
Mahalanobis Distance: 13.239
Mahalanobis Distance p-value: 0.00024

Prediction: Positive if the Bayesian score is above the estimated best cutoff value from minimizing the false positive and false negative rate.
Probability: The esimated probability that the sample is in the positive category. This assumes that the Bayesian score follows a normal distribution and is different from the prediction using a cutoff.
Enrichment: An estimate of enrichment, that is, the increased likelihood (versus random) of this sample being in the category.
Bayesian Score: The standard Laplacian-modified Bayesian score.
Mahalanobis Distance: The Mahalanobis distance (MD) is the distance to the center of the training data. The larger the MD, the less trustworthy the prediction.
Mahalanobis Distance p-value: The p-value gives the fraction of training data with an MD greater than or equal to the one for the given sample, assuming normally distributed data. The smaller the p-value, the less trustworthy the prediciton. For highly non-normal X properties (e.g., fingerprints), the MD p-value is wildly inaccurate.

Structural Similar Compounds

Name	Sulfasalazine	Bacampicillin	Nimodipine
Structure	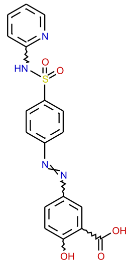	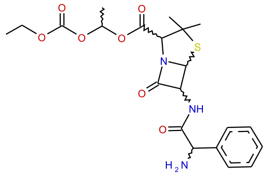	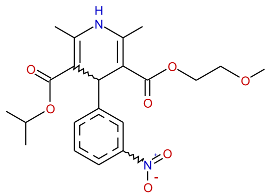
Actual Endpoint	Carcinogen	Non-Carcinogen	Non-Carcinogen
Predicted Endpoint	Carcinogen	Non-Carcinogen	Non-Carcinogen
Distance	0.702	0.734	0.739
Reference	US FDA (Centre for Drug Eval.& Res./Off. Testing & Res.) Sept. 1997	US FDA (Centre for Drug Eval.& Res./Off. Testing & Res.) Sept. 1997	US FDA (Centre for Drug Eval.& Res./Off. Testing & Res.) Sept. 1997

Model Applicability

Unknown features are fingerprint features in the query molecule, but not found in the training set.

1. All properties and OPS components are within expected ranges.
2. Unknown ECFP_2 feature: 1043790491: [*][N+](=[*])[*]
3. Unknown ECFP_2 feature: 781519895: [*][O-]
4. Unknown ECFP_2 feature: -955816473: [*]SCC(=[*])[*]
5. Unknown ECFP_2 feature: 1093109320: [*]S[c]1:o:[*]:[*]:n:1
6. Unknown ECFP_2 feature: 1427820655: [*]CS[c](:[*]):[*]
7. Unknown ECFP_2 feature: -1841325949: [*]:[c](:[*])C(C)[c](:[*]):[*]
8. Unknown ECFP_2 feature: 1092541557: [*]C([*])[c]1:o:[*]:[*]:n:1
9. Unknown ECFP_2 feature: -179073144: [*][N+](=[*])[c](:c:[*]):c:[*]
10. Unknown ECFP_2 feature: -215026467: [*]:[c](:[*])[N+](=O)[O-]
11. Unknown ECFP_2 feature: 2104376220: [*][N+](=O)[*]
12. Unknown ECFP_2 feature: -659271057: [*][N+](=[*])[O-]

Feature Contribution

Top features for positive contribution				
Fingerprint	Bit/Smiles	Feature Structure	Score	Carcinogen in training set

ECFP_6	-830332112	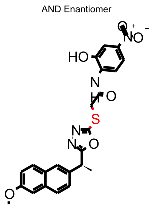 [*]S[*]	0.546	5 out of 8
ECFP_6	-178525456	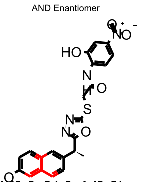 [*]:[cH]:[c](:[cH]:[*]):[c](:[*]):[*]	0.457	4 out of 7
ECFP_6	710652510	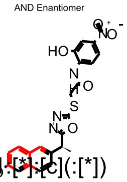 [*][c]1:[*]:[c](:[*]):[c](:[cH]:[*]):[cH]:[cH]:1	0.442	2 out of 3

Top Features for negative contribution

Fingerprint	Bit/Smiles	Feature Structure	Score	Carcinogen in training set
ECFP_6	1731843802	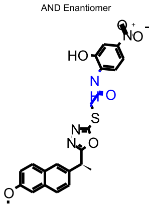 [*]CC(=O)N[*]	-0.657	0 out of 3
ECFP_6	1307307440	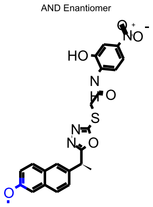 [*]:[c](:[*])OC	-0.558	4 out of 25

ECFP_6	1334400011	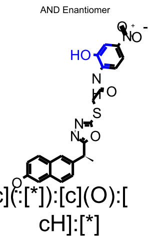 [*][c](:[*]):[c](O):[cH]:[*]	-0.496	3 out of 18

Sorafenib

TOPKAT_Mouse_Female_FDA_None_vs_Carcinogen

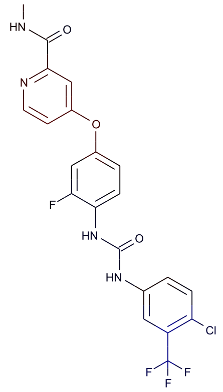

$C_{21}H_{15}ClF_{4}N_{4}O_{3}$
Molecular Weight: 482.81541
ALogP: 4.381
Rotatable Bonds: 6
Acceptors: 4
Donors: 3

Model Prediction

Prediction: Non-Carcinogen
Probability: 0.222
Enrichment: 0.692
Bayesian Score: -2.773
Mahalanobis Distance: 14.948
Mahalanobis Distance p-value: 3.1e-007

Prediction: Positive if the Bayesian score is above the estimated best cutoff value from minimizing the false positive and false negative rate.
Probability: The esimated probability that the sample is in the positive category. This assumes that the Bayesian score follows a normal distribution and is different from the prediction using a cutoff.
Enrichment: An estimate of enrichment, that is, the increased likelihood (versus random) of this sample being in the category.
Bayesian Score: The standard Laplacian-modified Bayesian score.
Mahalanobis Distance: The Mahalanobis distance (MD) is the distance to the center of the training data. The larger the MD, the less trustworthy the prediction.
Mahalanobis Distance p-value: The p-value gives the fraction of training data with an MD greater than or equal to the one for the given sample, assuming normally distributed data. The smaller the p-value, the less trustworthy the prediciton. For highly non-normal X properties (e.g., fingerprints), the MD p-value is wildly inaccurate.

Structural Similar Compounds

Name	Glimepride	Glyburide	Fluvastatin
Structure	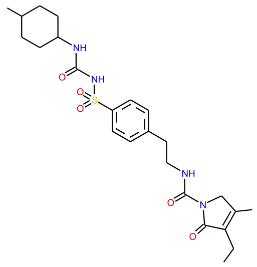	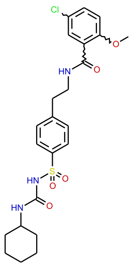	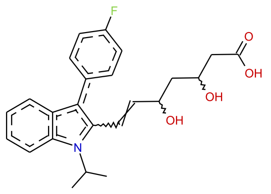
Actual Endpoint	Carcinogen	Non-Carcinogen	Non-Carcinogen
Predicted Endpoint	Carcinogen	Non-Carcinogen	Non-Carcinogen
Distance	0.610	0.615	0.643
Reference	US FDA (Centre for Drug Eval.& Res./Off. Testing & Res.) Sept. 1997	US FDA (Centre for Drug Eval.& Res./Off. Testing & Res.) Sept. 1997	US FDA (Centre for Drug Eval.& Res./Off. Testing & Res.) Sept. 1997

Model Applicability

Unknown features are fingerprint features in the query molecule, but not found in the training set.

1. OPS PC20 out of range. Value: -3.3157. Training min, max, SD, explained variance: -3.1862, 4.4571, 1.28, 0.0167.

Feature Contribution

Top features for positive contribution

Fingerprint	Bit/Smiles	Feature Structure	Score	Carcinogen in training set
ECFP_6	1338334141	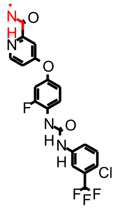 [*]C(=[*])NC	0.442	2 out of 3

ECFP_6	-834094296	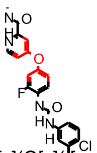 [*]:[cH]:[c](O[c](:[cH]:[*]):[cH]:[*]):[cH]:[*]	0.424	1 out of 1
ECFP_6	143734695	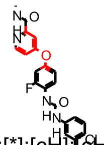 [*][c]1:[*]:[cH]:[cH]:[c](O[c](:[*]):[*]):[cH]:1	0.424	1 out of 1

Top Features for negative contribution

Fingerprint	Bit/Smiles	Feature Structure	Score	Carcinogen in training set
ECFP_6	1335691903	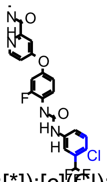 [*][c](:[*]):[c](Cl):[cH]:[*]	-0.669	3 out of 22
ECFP_6	-1952889961	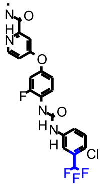 [*]:[c](:[*])C(F)(F)F	-0.657	0 out of 3
ECFP_6	1336678434	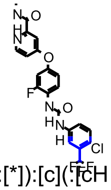 [*][c](:[*]):[c](:[cH]:[*])C([*])([*])[*]	-0.657	0 out of 3

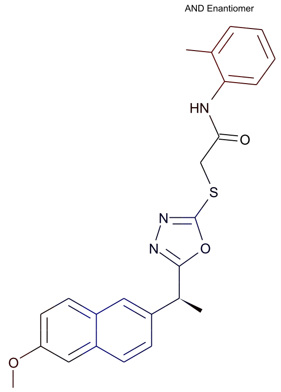

$C_{24}H_{23}N_3O_3S$
Molecular Weight: 433.52272
ALogP: 4.724
Rotatable Bonds: 7
Acceptors: 5
Donors: 1

Structural Similar Compounds

Name	Lovastatin	Simvastatin	Moricizine
Structure	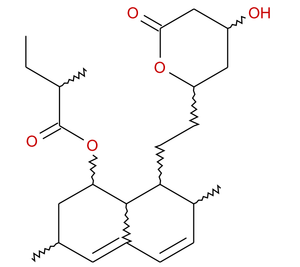	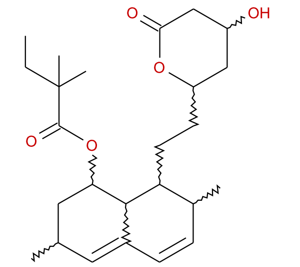	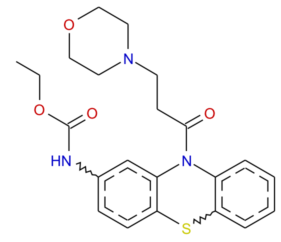
Actual Endpoint	Multiple-Carcinogen	Multiple-Carcinogen	Single-Carcinogen
Predicted Endpoint	Multiple-Carcinogen	Multiple-Carcinogen	Single-Carcinogen
Distance	0.553	0.557	0.610
Reference	US FDA (Centre for Drug Eval.& Res./Off. Testing & Res.) Sept. 1997	US FDA (Centre for Drug Eval.& Res./Off. Testing & Res.) Sept. 1997	US FDA (Centre for Drug Eval.& Res./Off. Testing & Res.) Sept. 1997

Model Prediction

Prediction: Single-Carcinogen
Probability: 0.322
Enrichment: 0.786
Bayesian Score: -2.319
Mahalanobis Distance: 10.949
Mahalanobis Distance p-value: 0.00302

Prediction: Positive if the Bayesian score is above the estimated best cutoff value from minimizing the false positive and false negative rate.
Probability: The esimated probability that the sample is in the positive category. This assumes that the Bayesian score follows a normal distribution and is different from the prediction using a cutoff.
Enrichment: An estimate of enrichment, that is, the increased likelihood (versus random) of this sample being in the category.
Bayesian Score: The standard Laplacian-modified Bayesian score.
Mahalanobis Distance: The Mahalanobis distance (MD) is the distance to the center of the training data. The larger the MD, the less trustworthy the prediction.
Mahalanobis Distance p-value: The p-value gives the fraction of training data with an MD greater than or equal to the one for the given sample, assuming normally distributed data. The smaller the p-value, the less trustworthy the prediciton. For highly non-normal X properties (e.g., fingerprints), the MD p-value is wildly inaccurate.

Model Applicability

Unknown features are fingerprint features in the query molecule, but not found in the training set.

1. All properties and OPS components are within expected ranges.
2. Unknown ECFP_2 feature: -955816473: [*]SCC(=[*])[*]
3. Unknown ECFP_2 feature: 1731843802: [*]CC(=O)N[*]
4. Unknown ECFP_2 feature: 1093109320: [*]S[c]1:o:[*]:[*]:n:1
5. Unknown ECFP_2 feature: 1427820655: [*]CS[c](:[*]):[*]
6. Unknown ECFP_2 feature: -1841325949: [*]:[c](:[*])C(C)[c](:[*]):[*]
7. Unknown ECFP_2 feature: 1092541557: [*]C([*])[c]1:o:[*]:[*]:n:1

Feature Contribution

Top features for positive contribution

Fingerprint	Bit/Smiles	Feature Structure	Score	Multiple-Carcinogen in training set

ECFP_4	1760125606	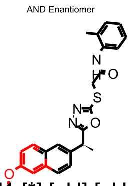 [*]:[c]1:[*]:[cH]:[cH]:[c](OC):[cH]:1	0.501	2 out of 2
ECFP_4	2055803015	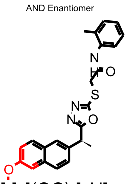 [*]:[cH]:[c](OC):[cH] :[*]	0.501	2 out of 2
ECFP_4	-1660962600	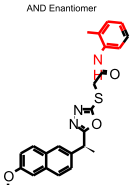 [*]N[c]1:[cH]:[*]:[cH]:[cH]:[c]:1C	0.351	1 out of 1
**Top Features for negative contribution**				
**Fingerprint**	**Bit/Smiles**	**Feature Structure**	**Score**	**Multiple-Carcinogen in training set**
ECFP_4	-178525456	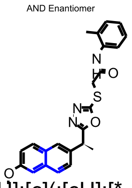 [*]:[cH]:[c](:[cH]:[*]):[c](:[*]):[*]	-0.968	0 out of 4
ECFP_4	1203316083	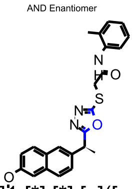 [*][c]1:[*]:[*]:[c]([*]):o:1	-0.800	0 out of 3

ECFP_4	911256832	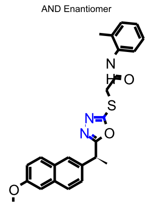<br>[*][c]1:[*]:[*]:n:n:1	-0.597	0 out of 2

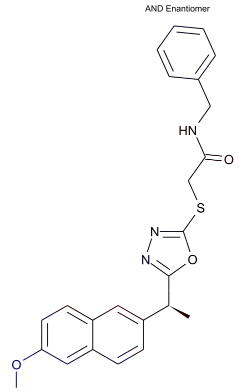

$C_{24}H_{23}N_3O_3S$
Molecular Weight: 433.52272
ALogP: 4.245
Rotatable Bonds: 8
Acceptors: 5
Donors: 1

Structural Similar Compounds

Name	Felodipine	Lovastatin	Simvastatin
Structure	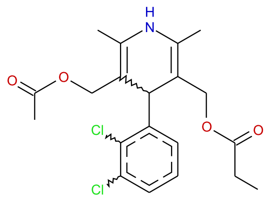	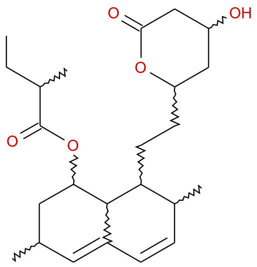	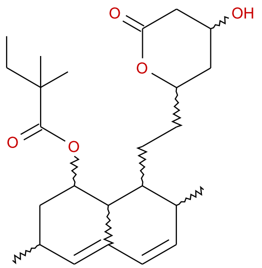
Actual Endpoint	Non-Carcinogen	Carcinogen	Carcinogen
Predicted Endpoint	Non-Carcinogen	Carcinogen	Carcinogen
Distance	0.561	0.591	0.599
Reference	US FDA (Centre for Drug Eval.& Res./Off. Testing & Res.) Sept. 1997	US FDA (Centre for Drug Eval.& Res./Off. Testing & Res.) Sept. 1997	US FDA (Centre for Drug Eval.& Res./Off. Testing & Res.) Sept. 1997

Model Prediction

Prediction: Non-Carcinogen
Probability: 0.256
Enrichment: 0.871
Bayesian Score: -2.001
Mahalanobis Distance: 15.623
Mahalanobis Distance p-value: 5.39e-009

Prediction: Positive if the Bayesian score is above the estimated best cutoff value from minimizing the false positive and false negative rate.
Probability: The esimated probability that the sample is in the positive category. This assumes that the Bayesian score follows a normal distribution and is different from the prediction using a cutoff.
Enrichment: An estimate of enrichment, that is, the increased likelihood (versus random) of this sample being in the category.
Bayesian Score: The standard Laplacian-modified Bayesian score.
Mahalanobis Distance: The Mahalanobis distance (MD) is the distance to the center of the training data. The larger the MD, the less trustworthy the prediction.
Mahalanobis Distance p-value: The p-value gives the fraction of training data with an MD greater than or equal to the one for the given sample, assuming normally distributed data. The smaller the p-value, the less trustworthy the prediciton. For highly non-normal X properties (e.g., fingerprints), the MD p-value is wildly inaccurate.

Model Applicability

Unknown features are fingerprint features in the query molecule, but not found in the training set.

1. All properties and OPS components are within expected ranges.

Feature Contribution

Top features for positive contribution

Fingerprint	Bit/Smiles	Feature Structure	Score	Carcinogen in training set
FCFP_6	-328193675	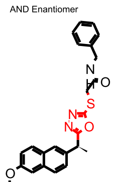 [*]S[c]1:o:[c]([*]):n:n:1	0.460	1 out of 1

FCFP_6	566058135	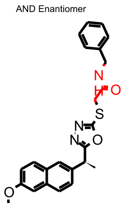 [*]CC(=O)N[*]	0.447	17 out of 40
FCFP_6	-105186863	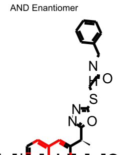 [*][c]1:[*]:[cH]:[c]2:[cH]:[*]:[cH]:[cH]:[c]:2:[cH]:1	0.380	2 out of 4
**Top Features for negative contribution**				
**Fingerprint**	**Bit/Smiles**	**Feature Structure**	**Score**	**Carcinogen in training set**
FCFP_6	1674955425	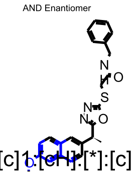 [*]O[c]1:[cH]:[*]:[c](:[*]):[c](:[cH]:[*]):[cH]:1	-0.719	0 out of 4
FCFP_6	86586436	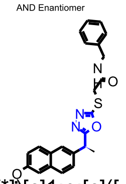 [*]C([*])[c]1:o:[c]([*]):n:n:1	-0.719	0 out of 4
FCFP_6	1514013367	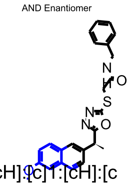 [*]:[cH]:[c]1:[cH]:[c](OC):[cH]:[cH]:[c]:1:[*]	-0.423	0 out of 2

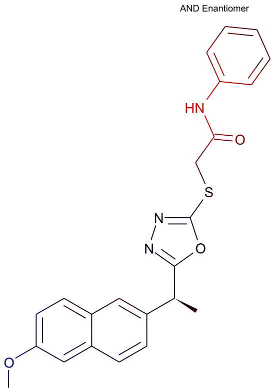

$C_{23}H_{21}N_3O_3S$
Molecular Weight: 419.49614
ALogP: 4.238
Rotatable Bonds: 7
Acceptors: 5
Donors: 1

Structural Similar Compounds

Name	Lovastatin	Moricizine	Felodipine
Structure	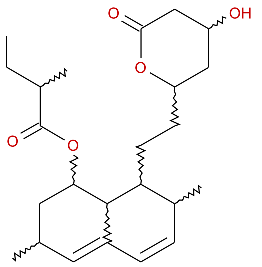	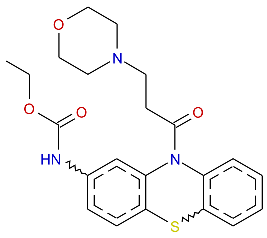	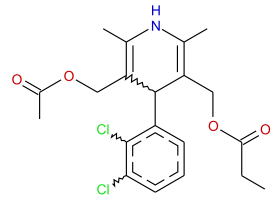
Actual Endpoint	Carcinogen	Non-Carcinogen	Non-Carcinogen
Predicted Endpoint	Carcinogen	Non-Carcinogen	Non-Carcinogen
Distance	0.576	0.587	0.588
Reference	US FDA (Centre for Drug Eval.& Res./Off. Testing & Res.) Sept. 1997	US FDA (Centre for Drug Eval.& Res./Off. Testing & Res.) Sept. 1997	US FDA (Centre for Drug Eval.& Res./Off. Testing & Res.) Sept. 1997

Model Prediction

Prediction: Carcinogen
Probability: 0.380
Enrichment: 1.291
Bayesian Score: 2.432
Mahalanobis Distance: 15.961
Mahalanobis Distance p-value: 1.08e-009

Prediction: Positive if the Bayesian score is above the estimated best cutoff value from minimizing the false positive and false negative rate.
Probability: The esimated probability that the sample is in the positive category. This assumes that the Bayesian score follows a normal distribution and is different from the prediction using a cutoff.
Enrichment: An estimate of enrichment, that is, the increased likelihood (versus random) of this sample being in the category.
Bayesian Score: The standard Laplacian-modified Bayesian score.
Mahalanobis Distance: The Mahalanobis distance (MD) is the distance to the center of the training data. The larger the MD, the less trustworthy the prediction.
Mahalanobis Distance p-value: The p-value gives the fraction of training data with an MD greater than or equal to the one for the given sample, assuming normally distributed data. The smaller the p-value, the less trustworthy the prediciton. For highly non-normal X properties (e.g., fingerprints), the MD p-value is wildly inaccurate.

Model Applicability

Unknown features are fingerprint features in the query molecule, but not found in the training set.

1. All properties and OPS components are within expected ranges.

Feature Contribution

Top features for positive contribution

Fingerprint	Bit/Smiles	Feature Structure	Score	Carcinogen in training set
FCFP_6	-451043714	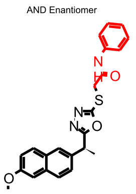 [*]CC(=O)N[c]1:[cH]:[cH]:[cH]:[cH]:[cH]:1	0.676	2 out of 2

FCFP_6	1175665944	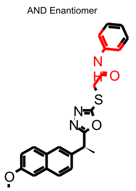 [*]CC(=O)N[c](:[cH]:[*]):[cH]:[*]	0.655	7 out of 12
FCFP_6	-1838187238	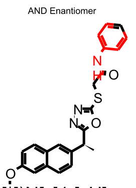 [*]C(=[*])N[c]1:[cH]: [cH]:[*]:[cH]:[cH]:1	0.565	4 out of 7
**Top Features for negative contribution**				
**Fingerprint**	**Bit/Smiles**	**Feature Structure**	**Score**	**Carcinogen in training set**
FCFP_6	1674955425	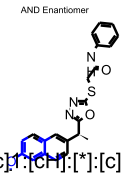 [*]O[c]1:[cH]:[*]:[c] (:[*]):[c](:[cH]:[*]):[cH]:1	-0.719	0 out of 4
FCFP_6	86586436	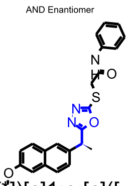 [*]C([*])[c]1:o:[c]([*]):n:n:1	-0.719	0 out of 4
FCFP_6	1514013367	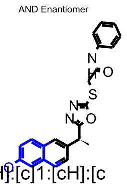 [*]:[cH]:[c]1:[cH]:[c](OC):[cH]:[cH]:[c]: 1:[*]	-0.423	0 out of 2

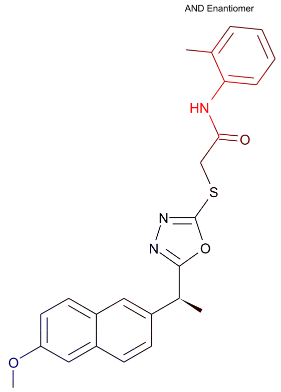

$C_{24}H_{23}N_3O_3S$
Molecular Weight: 433.52272
ALogP: 4.724
Rotatable Bonds: 7
Acceptors: 5
Donors: 1

Model Prediction

Prediction: Carcinogen
Probability: 0.427
Enrichment: 1.451
Bayesian Score: 3.765
Mahalanobis Distance: 15.333
Mahalanobis Distance p-value: 2.06e-008

Prediction: Positive if the Bayesian score is above the estimated best cutoff value from minimizing the false positive and false negative rate.
Probability: The esimated probability that the sample is in the positive category. This assumes that the Bayesian score follows a normal distribution and is different from the prediction using a cutoff.
Enrichment: An estimate of enrichment, that is, the increased likelihood (versus random) of this sample being in the category.
Bayesian Score: The standard Laplacian-modified Bayesian score.
Mahalanobis Distance: The Mahalanobis distance (MD) is the distance to the center of the training data. The larger the MD, the less trustworthy the prediction.
Mahalanobis Distance p-value: The p-value gives the fraction of training data with an MD greater than or equal to the one for the given sample, assuming normally distributed data. The smaller the p-value, the less trustworthy the prediciton. For highly non-normal X properties (e.g., fingerprints), the MD p-value is wildly inaccurate.

Structural Similar Compounds

Name	Simvastatin	Lovastatin	Felodipine
Structure	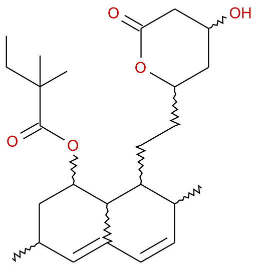	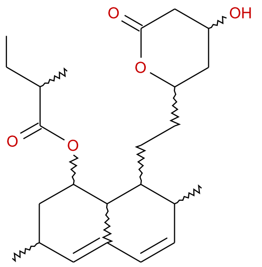	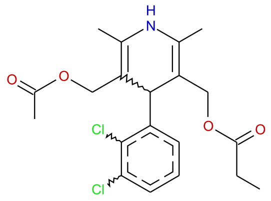
Actual Endpoint	Carcinogen	Carcinogen	Non-Carcinogen
Predicted Endpoint	Carcinogen	Carcinogen	Non-Carcinogen
Distance	0.576	0.583	0.604
Reference	US FDA (Centre for Drug Eval.& Res./Off. Testing & Res.) Sept. 1997	US FDA (Centre for Drug Eval.& Res./Off. Testing & Res.) Sept. 1997	US FDA (Centre for Drug Eval.& Res./Off. Testing & Res.) Sept. 1997

Model Applicability

Unknown features are fingerprint features in the query molecule, but not found in the training set.

1. OPS PC19 out of range. Value: -3.0531. Training min, max, SD, explained variance: -2.8152, 4.6113, 1.185, 0.0147.

Feature Contribution

Top features for positive contribution				
**Fingerprint**	**Bit/Smiles**	**Feature Structure**	**Score**	**Carcinogen in training set**
FCFP_6	1175665944	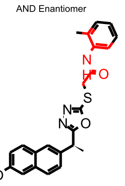 [*]CC(=O)N[c](:[cH]:[*]):[cH]:[*]	0.655	7 out of 12

FCFP_6	755520106	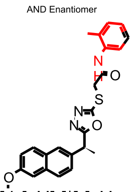 [*]N[c]1:[cH]:[*]:[cH]:[cH]:[c]:1C	0.517	2 out of 3
FCFP_6	1396506317	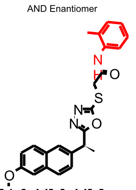 [*]N[c]1:[cH]:[cH]:[cH]:[cH]:[c]:1C	0.517	2 out of 3
**Top Features for negative contribution**				
**Fingerprint**	**Bit/Smiles**	**Feature Structure**	**Score**	**Carcinogen in training set**
FCFP_6	1674955425	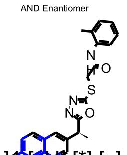 [*]O[c]1:[cH]:[*]:[c](:[*]):[c](:[cH]:[*]):[cH]:1	-0.719	0 out of 4
FCFP_6	86586436	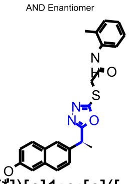 [*]C([*])[c]1:o:[c]([*]):n:n:1	-0.719	0 out of 4
FCFP_6	1514013367	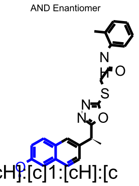 [*]:[cH]:[c]1:[cH]:[c](OC):[cH]:[cH]:[c]:1:[*]	-0.423	0 out of 2

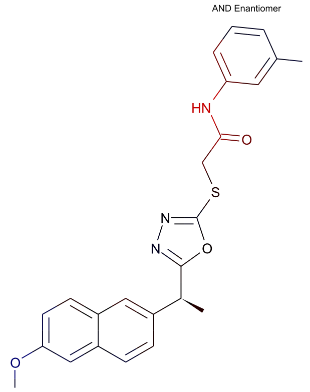

$C_{24}H_{23}N_3O_3S$
Molecular Weight: 433.52272
ALogP: 4.724
Rotatable Bonds: 7
Acceptors: 5
Donors: 1

Model Prediction

Prediction: Carcinogen
Probability: 0.344
Enrichment: 1.171
Bayesian Score: 1.330
Mahalanobis Distance: 14.846
Mahalanobis Distance p-value: 1.84e-007

Prediction: Positive if the Bayesian score is above the estimated best cutoff value from minimizing the false positive and false negative rate.
Probability: The esimated probability that the sample is in the positive category. This assumes that the Bayesian score follows a normal distribution and is different from the prediction using a cutoff.
Enrichment: An estimate of enrichment, that is, the increased likelihood (versus random) of this sample being in the category.
Bayesian Score: The standard Laplacian-modified Bayesian score.
Mahalanobis Distance: The Mahalanobis distance (MD) is the distance to the center of the training data. The larger the MD, the less trustworthy the prediction.
Mahalanobis Distance p-value: The p-value gives the fraction of training data with an MD greater than or equal to the one for the given sample, assuming normally distributed data. The smaller the p-value, the less trustworthy the prediciton. For highly non-normal X properties (e.g., fingerprints), the MD p-value is wildly inaccurate.

Structural Similar Compounds

Name	Simvastatin	Lovastatin	Felodipine
Structure	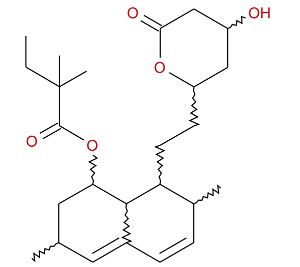	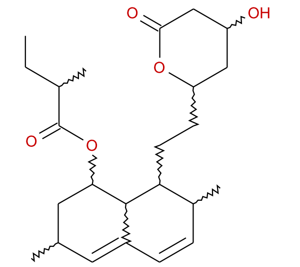	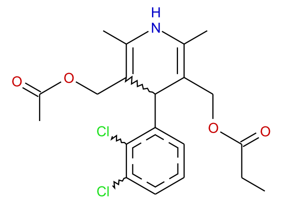
Actual Endpoint	Carcinogen	Carcinogen	Non-Carcinogen
Predicted Endpoint	Carcinogen	Carcinogen	Non-Carcinogen
Distance	0.576	0.583	0.604
Reference	US FDA (Centre for Drug Eval.& Res./Off. Testing & Res.) Sept. 1997	US FDA (Centre for Drug Eval.& Res./Off. Testing & Res.) Sept. 1997	US FDA (Centre for Drug Eval.& Res./Off. Testing & Res.) Sept. 1997

Model Applicability

Unknown features are fingerprint features in the query molecule, but not found in the training set.

1. OPS PC19 out of range. Value: -3.3263. Training min, max, SD, explained variance: -2.8152, 4.6113, 1.185, 0.0147.

Feature Contribution

Top features for positive contribution				
**Fingerprint**	**Bit/Smiles**	**Feature Structure**	**Score**	**Carcinogen in training set**
FCFP_6	1175665944	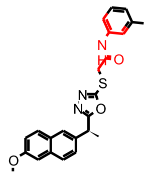 AND Enantiomer [*]CC(=O)N[c](:[cH]:[*]):[cH]:[*]	0.655	7 out of 12

FCFP_6	-1838187238	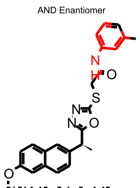 [*]C(=[*])N[c]1:[cH]:[cH]:[*]:[cH]:[cH]:1	0.565	4 out of 7
FCFP_6	-453277354	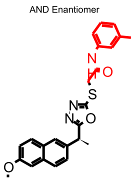 [*]CC(=O)N[c]1:[cH]:[cH]:[cH]:[c](C):[cH]:1	0.517	2 out of 3
**Top Features for negative contribution**				
**Fingerprint**	**Bit/Smiles**	**Feature Structure**	**Score**	**Carcinogen in training set**
FCFP_6	-1773728142	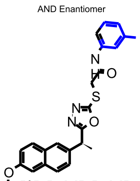 [*][c]1:[*]:[cH]:[cH]:[c](C):[cH]:1	-1.289	0 out of 10
FCFP_6	86586436	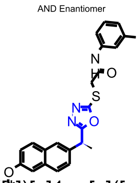 [*]C([*])[c]1:o:[c]([*]):n:n:1	-0.719	0 out of 4
FCFP_6	1674955425	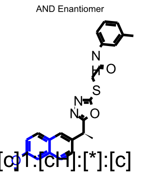 [*]O[c]1:[cH]:[*]:[c](:[*]):[c](:[cH]:[*]):[cH]:1	-0.719	0 out of 4

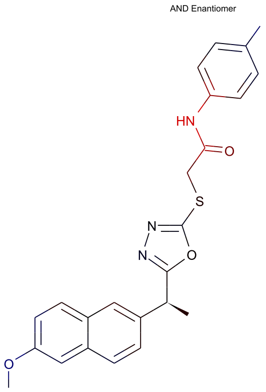

$C_{24}H_{23}N_3O_3S$
Molecular Weight: 433.52272
ALogP: 4.724
Rotatable Bonds: 7
Acceptors: 5
Donors: 1

Model Prediction

Prediction: Carcinogen
Probability: 0.337
Enrichment: 1.147
Bayesian Score: 1.101
Mahalanobis Distance: 14.531
Mahalanobis Distance p-value: 7.18e-007

Prediction: Positive if the Bayesian score is above the estimated best cutoff value from minimizing the false positive and false negative rate.
Probability: The esimated probability that the sample is in the positive category. This assumes that the Bayesian score follows a normal distribution and is different from the prediction using a cutoff.
Enrichment: An estimate of enrichment, that is, the increased likelihood (versus random) of this sample being in the category.
Bayesian Score: The standard Laplacian-modified Bayesian score.
Mahalanobis Distance: The Mahalanobis distance (MD) is the distance to the center of the training data. The larger the MD, the less trustworthy the prediction.
Mahalanobis Distance p-value: The p-value gives the fraction of training data with an MD greater than or equal to the one for the given sample, assuming normally distributed data. The smaller the p-value, the less trustworthy the prediciton. For highly non-normal X properties (e.g., fingerprints), the MD p-value is wildly inaccurate.

Structural Similar Compounds

Name	Simvastatin	Lovastatin	Felodipine
Structure	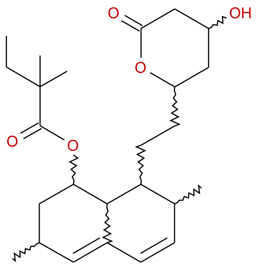	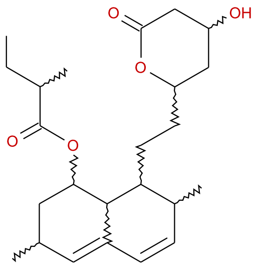	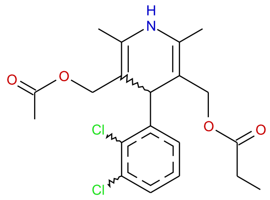
Actual Endpoint	Carcinogen	Carcinogen	Non-Carcinogen
Predicted Endpoint	Carcinogen	Carcinogen	Non-Carcinogen
Distance	0.575	0.583	0.608
Reference	US FDA (Centre for Drug Eval.& Res./Off. Testing & Res.) Sept. 1997	US FDA (Centre for Drug Eval.& Res./Off. Testing & Res.) Sept. 1997	US FDA (Centre for Drug Eval.& Res./Off. Testing & Res.) Sept. 1997

Model Applicability

Unknown features are fingerprint features in the query molecule, but not found in the training set.

1. OPS PC19 out of range. Value: -3.548. Training min, max, SD, explained variance: -2.8152, 4.6113, 1.185, 0.0147.

Feature Contribution

Top features for positive contribution

Fingerprint	Bit/Smiles	Feature Structure	Score	Carcinogen in training set
FCFP_6	-451043714	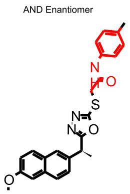 [*]CC(=O)N[c]1:[cH]:[cH]:[cH]:[cH]:[cH]:1	0.676	2 out of 2

FCFP_6	1175665944	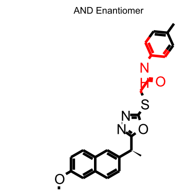 [*]CC(=O)N[c](:[cH]:[*]):[cH]:[*]	0.655	7 out of 12
FCFP_6	-1838187238	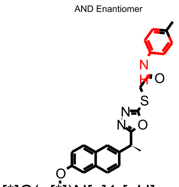 [*]C(=[*])N[c]1:[cH]: [cH]:[*]:[cH]:[cH]:1	0.565	4 out of 7
**Top Features for negative contribution**				
**Fingerprint**	**Bit/Smiles**	**Feature Structure**	**Score**	**Carcinogen in training set**
FCFP_6	-1773728142	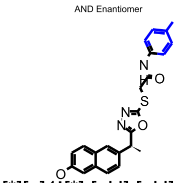 [*][c]1:[*]:[cH]:[cH] :[c](C):[cH]:1	-1.289	0 out of 10
FCFP_6	2109043264	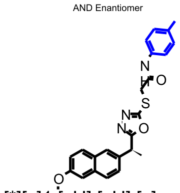 [*][c]1:[cH]:[cH]:[c] (C):[cH]:[cH]:1	-0.947	0 out of 6
FCFP_6	86586436	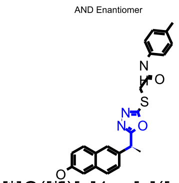 [*]C([*])[c]1:o:[c]([*]):n:n:1	-0.719	0 out of 4

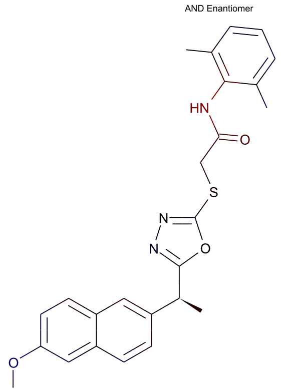

$C_{25}H_{25}N_3O_3S$
Molecular Weight: 447.5493
ALogP: 5.21
Rotatable Bonds: 7
Acceptors: 5
Donors: 1

Model Prediction

Prediction: Carcinogen
Probability: 0.337
Enrichment: 1.145
Bayesian Score: 1.083
Mahalanobis Distance: 15.130
Mahalanobis Distance p-value: 5.19e-008

Prediction: Positive if the Bayesian score is above the estimated best cutoff value from minimizing the false positive and false negative rate.
Probability: The esimated probability that the sample is in the positive category. This assumes that the Bayesian score follows a normal distribution and is different from the prediction using a cutoff.
Enrichment: An estimate of enrichment, that is, the increased likelihood (versus random) of this sample being in the category.
Bayesian Score: The standard Laplacian-modified Bayesian score.
Mahalanobis Distance: The Mahalanobis distance (MD) is the distance to the center of the training data. The larger the MD, the less trustworthy the prediction.
Mahalanobis Distance p-value: The p-value gives the fraction of training data with an MD greater than or equal to the one for the given sample, assuming normally distributed data. The smaller the p-value, the less trustworthy the prediciton. For highly non-normal X properties (e.g., fingerprints), the MD p-value is wildly inaccurate.

Structural Similar Compounds

Name	Simvastatin	Lovastatin	Felodipine
Structure	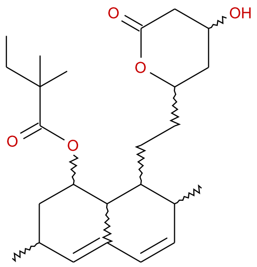	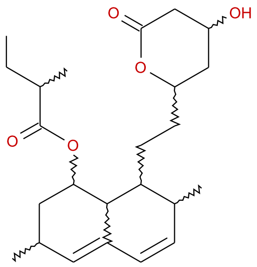	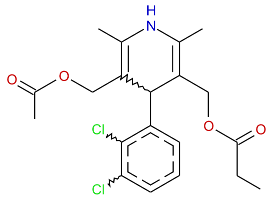
Actual Endpoint	Carcinogen	Carcinogen	Non-Carcinogen
Predicted Endpoint	Carcinogen	Carcinogen	Non-Carcinogen
Distance	0.587	0.614	0.634
Reference	US FDA (Centre for Drug Eval.& Res./Off. Testing & Res.) Sept. 1997	US FDA (Centre for Drug Eval.& Res./Off. Testing & Res.) Sept. 1997	US FDA (Centre for Drug Eval.& Res./Off. Testing & Res.) Sept. 1997

Model Applicability

Unknown features are fingerprint features in the query molecule, but not found in the training set.

1. OPS PC19 out of range. Value: -2.8814. Training min, max, SD, explained variance: -2.8152, 4.6113, 1.185, 0.0147.

Feature Contribution

Top features for positive contribution

Fingerprint	Bit/Smiles	Feature Structure	Score	Carcinogen in training set
FCFP_6	1175665944	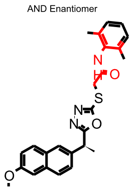 [*]CC(=O)N[c](:[cH]:[*]):[cH]:[*]	0.655	7 out of 12

FCFP_6	1396506317	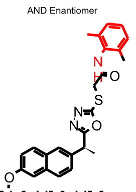 [*]N[c]1:[cH]:[cH]:[cH]:[cH]:[c]:1C	0.517	2 out of 3
FCFP_6	755520106	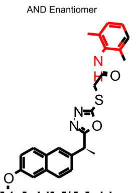 [*]N[c]1:[cH]:[*]:[cH]:[cH]:[c]:1C	0.517	2 out of 3
**Top Features for negative contribution**				
**Fingerprint**	**Bit/Smiles**	**Feature Structure**	**Score**	**Carcinogen in training set**
FCFP_6	-1696375691	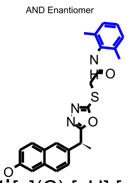 [*][c]1:[c](C):[cH]:[cH]:[cH]:[c]:1C	-0.839	0 out of 5
FCFP_6	1674955425	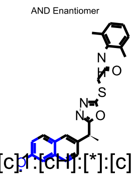 [*]O[c]1:[cH]:[*]:[c](:[*]):[c](:[cH]:[*]):[cH]:1	-0.719	0 out of 4
FCFP_6	86586436	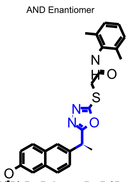 [*]C([*])[c]1:o:[c]([*]):n:n:1	-0.719	0 out of 4

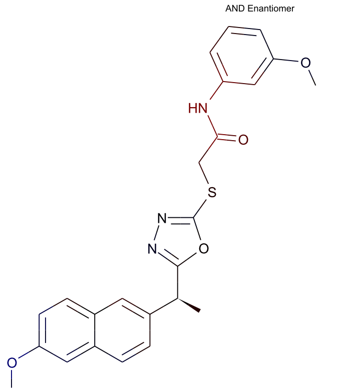

$C_{24}H_{23}N_3O_4S$

Molecular Weight: 449.52212

ALogP: 4.221

Rotatable Bonds: 8

Acceptors: 6

Donors: 1

Structural Similar Compounds

Name	Moricizine	Felodipine	Diltiazem
Structure	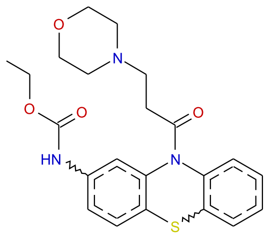	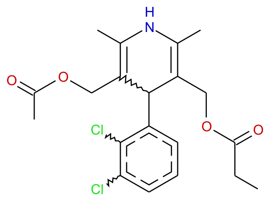	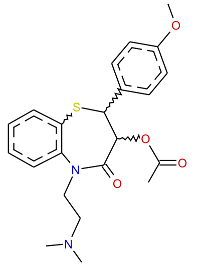
Actual Endpoint	Non-Carcinogen	Non-Carcinogen	Non-Carcinogen
Predicted Endpoint	Non-Carcinogen	Non-Carcinogen	Non-Carcinogen
Distance	0.600	0.613	0.635
Reference	US FDA (Centre for Drug Eval.& Res./Off. Testing & Res.) Sept. 1997	US FDA (Centre for Drug Eval.& Res./Off. Testing & Res.) Sept. 1997	US FDA (Centre for Drug Eval.& Res./Off. Testing & Res.) Sept. 1997

Model Prediction

Prediction: Carcinogen

Probability: 0.309

Enrichment: 1.050

Bayesian Score: 0.113

Mahalanobis Distance: 14.765

Mahalanobis Distance p-value: 2.62e-007

Prediction: Positive if the Bayesian score is above the estimated best cutoff value from minimizing the false positive and false negative rate.
Probability: The esimated probability that the sample is in the positive category. This assumes that the Bayesian score follows a normal distribution and is different from the prediction using a cutoff.
Enrichment: An estimate of enrichment, that is, the increased likelihood (versus random) of this sample being in the category.
Bayesian Score: The standard Laplacian-modified Bayesian score.
Mahalanobis Distance: The Mahalanobis distance (MD) is the distance to the center of the training data. The larger the MD, the less trustworthy the prediction.
Mahalanobis Distance p-value: The p-value gives the fraction of training data with an MD greater than or equal to the one for the given sample, assuming normally distributed data. The smaller the p-value, the less trustworthy the prediciton. For highly non-normal X properties (e.g., fingerprints), the MD p-value is wildly inaccurate.

Model Applicability

Unknown features are fingerprint features in the query molecule, but not found in the training set.

1. OPS PC19 out of range. Value: -2.8411. Training min, max, SD, explained variance: -2.8152, 4.6113, 1.185, 0.0147.

Feature Contribution

Top features for positive contribution				
**Fingerprint**	**Bit/Smiles**	**Feature Structure**	**Score**	**Carcinogen in training set**
FCFP_6	1175665944	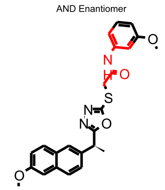 [*]CC(=O)N[c](:[cH]:[*]):[cH]:[*]	0.655	7 out of 12

FCFP_6	-1838187238	AND Enantiomer 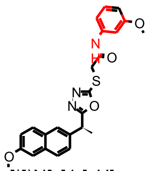 [*]C(=[*])N[c]1:[cH]:[cH]:[*]:[cH]:[cH]:1	0.565	4 out of 7
FCFP_6	-1947166985	AND Enantiomer 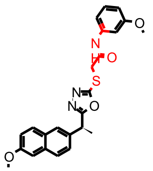 [*]SCC(=O)N[c](:[*]):[*]	0.460	1 out of 1
**Top Features for negative contribution**				
**Fingerprint**	**Bit/Smiles**	**Feature Structure**	**Score**	**Carcinogen in training set**
FCFP_6	1674955425	AND Enantiomer 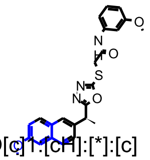 [*]O[c]1:[cH]:[*]:[c](:[*]):[c](:[cH]:[*]):[cH]:1	-0.719	0 out of 4
FCFP_6	86586436	AND Enantiomer 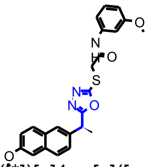 [*]C([*])[c]1:o:[c]([*]):n:n:1	-0.719	0 out of 4
FCFP_6	-768690632	AND Enantiomer 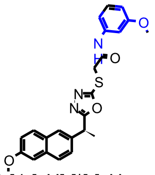 [*]N[c]1:[cH]:[*]:[cH]:[c](O[*]):[cH]:1	-0.582	0 out of 3

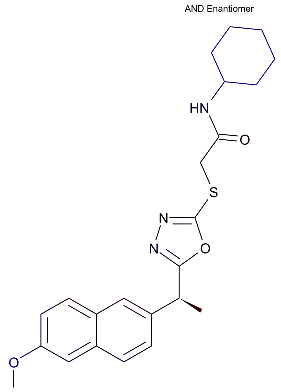

$C_{23}H_{27}N_3O_3S$
Molecular Weight: 425.54378
ALogP: 4.52
Rotatable Bonds: 7
Acceptors: 5
Donors: 1

Model Prediction

Prediction: Non-Carcinogen
Probability: 0.228
Enrichment: 0.774
Bayesian Score: -3.385
Mahalanobis Distance: 14.831
Mahalanobis Distance p-value: 1.96e-007

Prediction: Positive if the Bayesian score is above the estimated best cutoff value from minimizing the false positive and false negative rate.
Probability: The esimated probability that the sample is in the positive category. This assumes that the Bayesian score follows a normal distribution and is different from the prediction using a cutoff.
Enrichment: An estimate of enrichment, that is, the increased likelihood (versus random) of this sample being in the category.
Bayesian Score: The standard Laplacian-modified Bayesian score.
Mahalanobis Distance: The Mahalanobis distance (MD) is the distance to the center of the training data. The larger the MD, the less trustworthy the prediction.
Mahalanobis Distance p-value: The p-value gives the fraction of training data with an MD greater than or equal to the one for the given sample, assuming normally distributed data. The smaller the p-value, the less trustworthy the prediciton. For highly non-normal X properties (e.g., fingerprints), the MD p-value is wildly inaccurate.

Structural Similar Compounds

Name	Lovastatin	Simvastatin	Felodipine
Structure	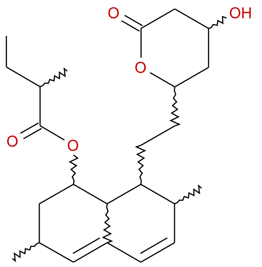	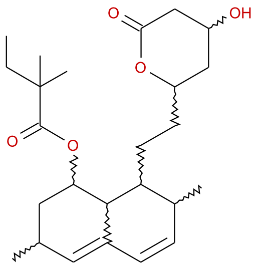	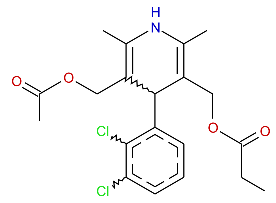
Actual Endpoint	Carcinogen	Carcinogen	Non-Carcinogen
Predicted Endpoint	Carcinogen	Carcinogen	Non-Carcinogen
Distance	0.566	0.570	0.589
Reference	US FDA (Centre for Drug Eval.& Res./Off. Testing & Res.) Sept. 1997	US FDA (Centre for Drug Eval.& Res./Off. Testing & Res.) Sept. 1997	US FDA (Centre for Drug Eval.& Res./Off. Testing & Res.) Sept. 1997

Model Applicability

Unknown features are fingerprint features in the query molecule, but not found in the training set.

1. All properties and OPS components are within expected ranges.

Feature Contribution

Top features for positive contribution

Fingerprint	Bit/Smiles	Feature Structure	Score	Carcinogen in training set
FCFP_6	-328193675	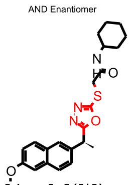 [*]S[c]1:o:[c]([*]):n :n:1	0.460	1 out of 1

FCFP_6	566058135	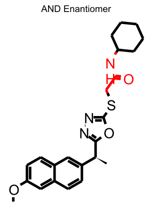 [*]CC(=O)N[*]	0.447	17 out of 40
FCFP_6	-105186863	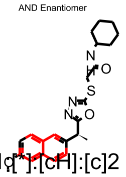 [*][c]1:[*]:[cH]:[c]2 :[cH]:[*]:[cH]:[cH]: [c]:2:[cH]:1	0.380	2 out of 4
**Top Features for negative contribution**				
**Fingerprint**	**Bit/Smiles**	**Feature Structure**	**Score**	**Carcinogen in training set**
FCFP_6	-1525101452	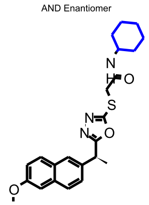 [*]C1CCCCC1	-1.133	0 out of 8
FCFP_6	86586436	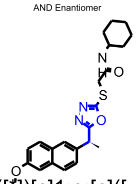 [*]C([*])[c]1:o:[c]([*]):n:n:1	-0.719	0 out of 4
FCFP_6	1674955425	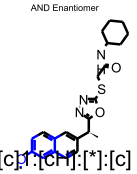 [*]O[c]1:[cH]:[*]:[c] (:[*]):[c](:[cH]:[*]):[cH]:1	-0.719	0 out of 4

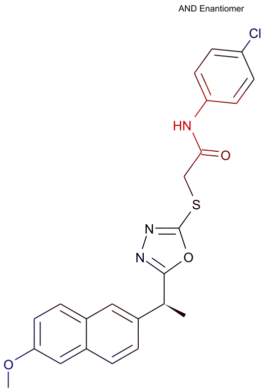

$C_{23}H_{20}ClN_3O_3S$
Molecular Weight: 453.9412
ALogP: 4.902
Rotatable Bonds: 7
Acceptors: 5
Donors: 1

Model Prediction

Prediction: Carcinogen
Probability: 0.336
Enrichment: 1.142
Bayesian Score: 1.059
Mahalanobis Distance: 14.801
Mahalanobis Distance p-value: 2.24e-007

Prediction: Positive if the Bayesian score is above the estimated best cutoff value from minimizing the false positive and false negative rate.
Probability: The esimated probability that the sample is in the positive category. This assumes that the Bayesian score follows a normal distribution and is different from the prediction using a cutoff.
Enrichment: An estimate of enrichment, that is, the increased likelihood (versus random) of this sample being in the category.
Bayesian Score: The standard Laplacian-modified Bayesian score.
Mahalanobis Distance: The Mahalanobis distance (MD) is the distance to the center of the training data. The larger the MD, the less trustworthy the prediction.
Mahalanobis Distance p-value: The p-value gives the fraction of training data with an MD greater than or equal to the one for the given sample, assuming normally distributed data. The smaller the p-value, the less trustworthy the prediciton. For highly non-normal X properties (e.g., fingerprints), the MD p-value is wildly inaccurate.

Structural Similar Compounds

Name	Simvastatin	Felodipine	Lovastatin
Structure	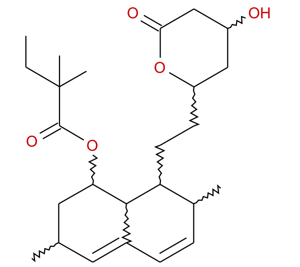	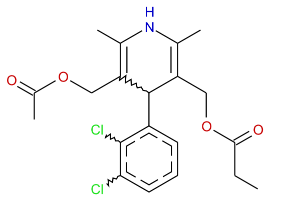	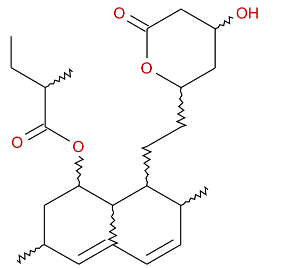
Actual Endpoint	Carcinogen	Non-Carcinogen	Carcinogen
Predicted Endpoint	Carcinogen	Non-Carcinogen	Carcinogen
Distance	0.594	0.611	0.612
Reference	US FDA (Centre for Drug Eval.& Res./Off. Testing & Res.) Sept. 1997	US FDA (Centre for Drug Eval.& Res./Off. Testing & Res.) Sept. 1997	US FDA (Centre for Drug Eval.& Res./Off. Testing & Res.) Sept. 1997

Model Applicability

Unknown features are fingerprint features in the query molecule, but not found in the training set.

1. OPS PC19 out of range. Value: -3.3341. Training min, max, SD, explained variance: -2.8152, 4.6113, 1.185, 0.0147.

Feature Contribution

Top features for positive contribution				
**Fingerprint**	**Bit/Smiles**	**Feature Structure**	**Score**	**Carcinogen in training set**
FCFP_6	-451043714	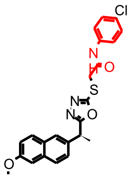 AND Enantiomer [*]CC(=O)N[c]1:[cH]:[cH]:[cH]:[cH]:[cH]:1	0.676	2 out of 2

FCFP_6	1175665944	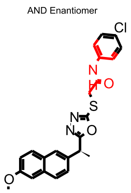<br>[*]CC(=O)N[c](:[cH]:[*]):[cH]:[*]	0.655	7 out of 12
FCFP_6	-1838187238	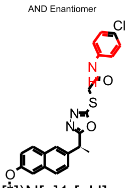<br>[*]C(=[*])N[c]1:[cH]:[cH]:[*]:[cH]:[cH]:1	0.565	4 out of 7

Top Features for negative contribution

Fingerprint	Bit/Smiles	Feature Structure	Score	Carcinogen in training set
FCFP_6	1674955425	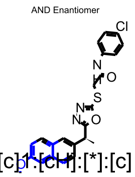<br>[*]O[c]1:[cH]:[*]:[c](:[*]):[c](:[cH]:[*]):[cH]:1	-0.719	0 out of 4
FCFP_6	86586436	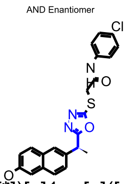<br>[*]C([*])[c]1:o:[c]([*]):n:n:1	-0.719	0 out of 4
FCFP_6	551850122	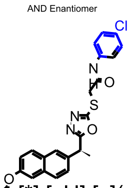<br>[*][c]1:[*]:[cH]:[c](Cl):[cH]:[cH]:1	-0.433	8 out of 49

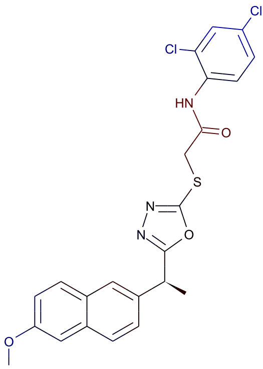

$C_{23}H_{19}Cl_2N_3O_3S$

Molecular Weight: 488.38626

ALogP: 5.567

Rotatable Bonds: 7

Acceptors: 5

Donors: 1

Model Prediction

Prediction: Non-Carcinogen

Probability: 0.258

Enrichment: 0.877

Bayesian Score: -1.916

Mahalanobis Distance: 14.866

Mahalanobis Distance p-value: 1.68e-007

Prediction: Positive if the Bayesian score is above the estimated best cutoff value from minimizing the false positive and false negative rate.
Probability: The esimated probability that the sample is in the positive category. This assumes that the Bayesian score follows a normal distribution and is different from the prediction using a cutoff.
Enrichment: An estimate of enrichment, that is, the increased likelihood (versus random) of this sample being in the category.
Bayesian Score: The standard Laplacian-modified Bayesian score.
Mahalanobis Distance: The Mahalanobis distance (MD) is the distance to the center of the training data. The larger the MD, the less trustworthy the prediction.
Mahalanobis Distance p-value: The p-value gives the fraction of training data with an MD greater than or equal to the one for the given sample, assuming normally distributed data. The smaller the p-value, the less trustworthy the prediciton. For highly non-normal X properties (e.g., fingerprints), the MD p-value is wildly inaccurate.

Structural Similar Compounds

Name	Astemizole	Emetine	Simvastatin
Structure	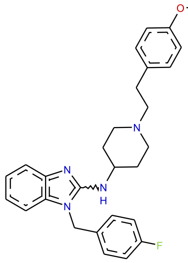	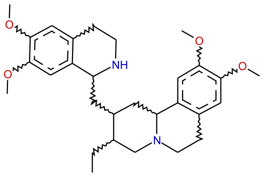	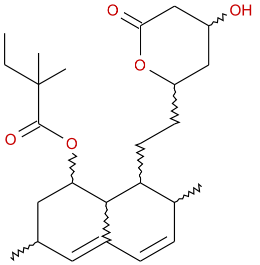
Actual Endpoint	Non-Carcinogen	Non-Carcinogen	Carcinogen
Predicted Endpoint	Non-Carcinogen	Non-Carcinogen	Carcinogen
Distance	0.623	0.641	0.648
Reference	US FDA (Centre for Drug Eval.& Res./Off. Testing & Res.) Sept. 1997	US FDA (Centre for Drug Eval.& Res./Off. Testing & Res.) Sept. 1997	US FDA (Centre for Drug Eval.& Res./Off. Testing & Res.) Sept. 1997

Model Applicability

Unknown features are fingerprint features in the query molecule, but not found in the training set.

1. OPS PC19 out of range. Value: -3.3695. Training min, max, SD, explained variance: -2.8152, 4.6113, 1.185, 0.0147.

Feature Contribution

Top features for positive contribution				
**Fingerprint**	**Bit/Smiles**	**Feature Structure**	**Score**	**Carcinogen in training set**
FCFP_6	1175665944	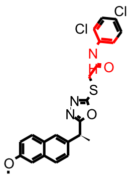 AND Enantiomer [*]CC(=O)N[c](:[cH]:[*]):[cH]:[*]	0.655	7 out of 12

FCFP_6	-328193675	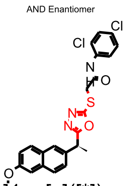 [*]S[c]1:o:[c]([*]):n :n:1	0.460	1 out of 1
FCFP_6	-1947166985	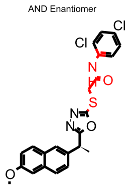 [*]SCC(=O)N[c](:[*]): [*]	0.460	1 out of 1

Top Features for negative contribution

**Fingerprint**	**Bit/Smiles**	**Feature Structure**	**Score**	**Carcinogen in training set**
FCFP_6	555188808	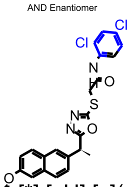 [*][c]1:[*]:[cH]:[c](Cl):[cH]:[c]:1Cl	-0.839	0 out of 5
FCFP_6	1783756416	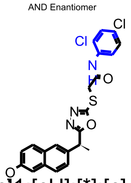 [*]N[c]1:[cH]:[*]:[c] ([*]):[cH]:[c]:1Cl	-0.719	0 out of 4
FCFP_6	73264552	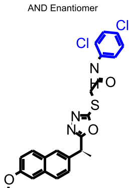 [*][c]1:[cH]:[cH]:[c] (Cl):[cH]:[c]:1Cl	-0.719	0 out of 4

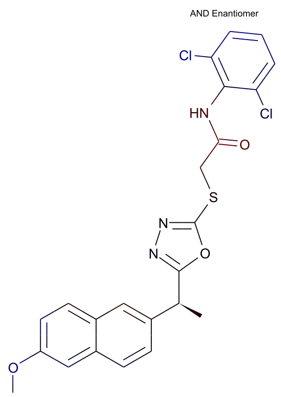

$C_{23}H_{19}Cl_2N_3O_3S$
Molecular Weight: 488.38626
ALogP: 5.567
Rotatable Bonds: 7
Acceptors: 5
Donors: 1

Model Prediction

Prediction: Carcinogen
Probability: 0.268
Enrichment: 0.911
Bayesian Score: -1.480
Mahalanobis Distance: 16.993
Mahalanobis Distance p-value: 6.7e-012

Prediction: Positive if the Bayesian score is above the estimated best cutoff value from minimizing the false positive and false negative rate.
Probability: The esimated probability that the sample is in the positive category. This assumes that the Bayesian score follows a normal distribution and is different from the prediction using a cutoff.
Enrichment: An estimate of enrichment, that is, the increased likelihood (versus random) of this sample being in the category.
Bayesian Score: The standard Laplacian-modified Bayesian score.
Mahalanobis Distance: The Mahalanobis distance (MD) is the distance to the center of the training data. The larger the MD, the less trustworthy the prediction.
Mahalanobis Distance p-value: The p-value gives the fraction of training data with an MD greater than or equal to the one for the given sample, assuming normally distributed data. The smaller the p-value, the less trustworthy the prediciton. For highly non-normal X properties (e.g., fingerprints), the MD p-value is wildly inaccurate.

Structural Similar Compounds

Name	Astemizole	Emetine	Simvastatin
Structure	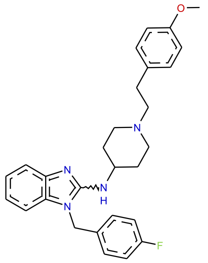	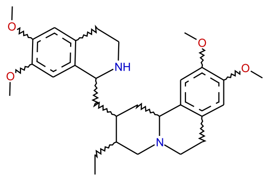	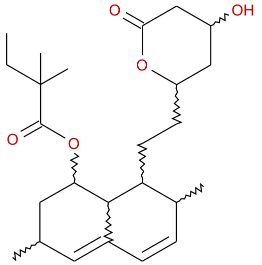
Actual Endpoint	Non-Carcinogen	Non-Carcinogen	Carcinogen
Predicted Endpoint	Non-Carcinogen	Non-Carcinogen	Carcinogen
Distance	0.621	0.640	0.648
Reference	US FDA (Centre for Drug Eval.& Res./Off. Testing & Res.) Sept. 1997	US FDA (Centre for Drug Eval.& Res./Off. Testing & Res.) Sept. 1997	US FDA (Centre for Drug Eval.& Res./Off. Testing & Res.) Sept. 1997

Model Applicability

Unknown features are fingerprint features in the query molecule, but not found in the training set.

1. All properties and OPS components are within expected ranges.

Feature Contribution

Top features for positive contribution

Fingerprint	Bit/Smiles	Feature Structure	Score	Carcinogen in training set
FCFP_6	1175665944	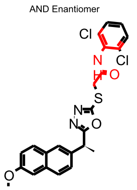 [*]CC(=O)N[c](:[cH]:[*]):[cH]:[*]	0.655	7 out of 12

FCFP_6	-328193675	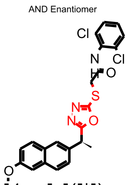 [*]S[c]1:o:[c]([*]):n:n:1	0.460	1 out of 1
FCFP_6	-1947166985	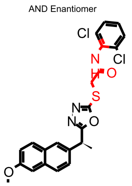 [*]SCC(=O)N[c](:[*]):[*]	0.460	1 out of 1
**Top Features for negative contribution**				
**Fingerprint**	**Bit/Smiles**	**Feature Structure**	**Score**	**Carcinogen in training set**
FCFP_6	1783756416	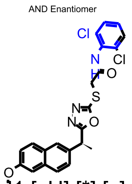 [*]N[c]1:[cH]:[*]:[c]([*]):[cH]:[c]:1Cl	-0.719	0 out of 4
FCFP_6	1674955425	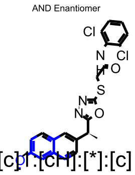 [*]O[c]1:[cH]:[*]:[c](:[*]):[c](:[cH]:[*]):[cH]:1	-0.719	0 out of 4
FCFP_6	1161767339	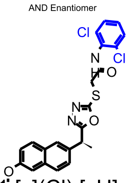 [*][c]1:[c](Cl):[cH]:[cH]:[cH]:[c]:1Cl	-0.719	0 out of 4

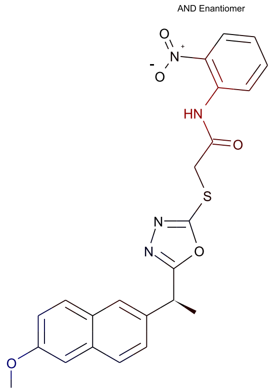

$C_{23}H_{20}N_4O_5S$
Molecular Weight: 464.4937
ALogP: 4.132
Rotatable Bonds: 8
Acceptors: 7
Donors: 1

Model Prediction

Prediction: Carcinogen
Probability: 0.332
Enrichment: 1.128
Bayesian Score: 0.917
Mahalanobis Distance: 14.786
Mahalanobis Distance p-value: 2.38e-007

Prediction: Positive if the Bayesian score is above the estimated best cutoff value from minimizing the false positive and false negative rate.
Probability: The esimated probability that the sample is in the positive category. This assumes that the Bayesian score follows a normal distribution and is different from the prediction using a cutoff.
Enrichment: An estimate of enrichment, that is, the increased likelihood (versus random) of this sample being in the category.
Bayesian Score: The standard Laplacian-modified Bayesian score.
Mahalanobis Distance: The Mahalanobis distance (MD) is the distance to the center of the training data. The larger the MD, the less trustworthy the prediction.
Mahalanobis Distance p-value: The p-value gives the fraction of training data with an MD greater than or equal to the one for the given sample, assuming normally distributed data. The smaller the p-value, the less trustworthy the prediciton. For highly non-normal X properties (e.g., fingerprints), the MD p-value is wildly inaccurate.

Structural Similar Compounds

Name	Moricizine	Nisoldipine	Fluticasone
Structure	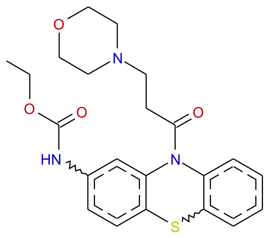	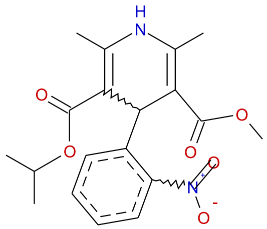	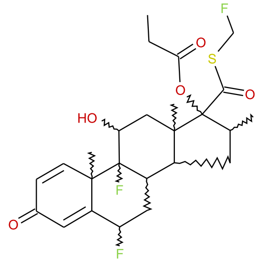
Actual Endpoint	Non-Carcinogen	Non-Carcinogen	Non-Carcinogen
Predicted Endpoint	Non-Carcinogen	Non-Carcinogen	Carcinogen
Distance	0.672	0.683	0.694
Reference	US FDA (Centre for Drug Eval.& Res./Off. Testing & Res.) Sept. 1997	US FDA (Centre for Drug Eval.& Res./Off. Testing & Res.) Sept. 1997	US FDA (Centre for Drug Eval.& Res./Off. Testing & Res.) Sept. 1997

Model Applicability

Unknown features are fingerprint features in the query molecule, but not found in the training set.

1. OPS PC19 out of range. Value: -2.8607. Training min, max, SD, explained variance: -2.8152, 4.6113, 1.185, 0.0147.
2. Unknown FCFP_2 feature: 5: [*][O-]
3. Unknown FCFP_2 feature: -828984032: [*][c](:[*]):[c]([N+](=[*])[*]):c:[*]
4. Unknown FCFP_2 feature: -1338588315: [*]:[c](:[*])[N+](=O)[O-]
5. Unknown FCFP_2 feature: 1872392852: [*][N+](=O)[*]
6. Unknown FCFP_2 feature: 260476081: [*][N+](=[*])[O-]

Feature Contribution

Top features for positive contribution				
Fingerprint	Bit/Smiles	Feature Structure	Score	Carcinogen in training set

FCFP_6	1175665944	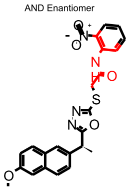 [*]CC(=O)N[c](:[cH]:[*]):[cH]:[*]	0.655	7 out of 12
FCFP_6	-328193675	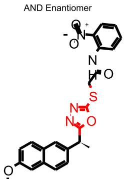 [*]S[c]1:o:[c]([*]):n:n:1	0.460	1 out of 1
FCFP_6	-1947166985	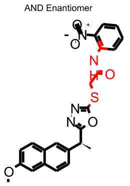 [*]SCC(=O)N[c](:[*]):[*]	0.460	1 out of 1

Top Features for negative contribution

Fingerprint	Bit/Smiles	Feature Structure	Score	Carcinogen in training set
FCFP_6	86586436	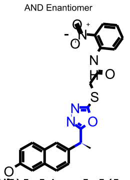 [*]C([*])[c]1:o:[c]([*]):n:n:1	-0.719	0 out of 4
FCFP_6	1674955425	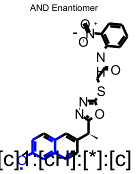 [*]O[c]1:[cH]:[*]:[c](:[*]):[c](:[cH]:[*]):[cH]:1	-0.719	0 out of 4

FCFP_6	1514013367	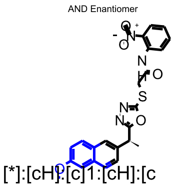 [*]:[cH]:[c]1:[cH]:[c](OC):[cH]:[cH]:[c]:1:[*]	-0.423	0 out of 2

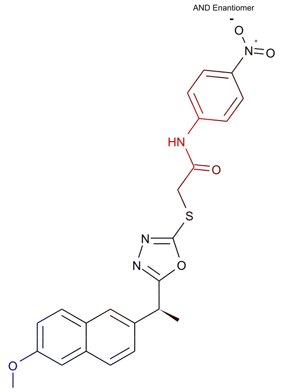

$C_{23}H_{20}N_4O_5S$
Molecular Weight: 464.4937
ALogP: 4.132
Rotatable Bonds: 8
Acceptors: 7
Donors: 1

Model Prediction

Prediction: Carcinogen
Probability: 0.369
Enrichment: 1.254
Bayesian Score: 2.105
Mahalanobis Distance: 14.331
Mahalanobis Distance p-value: 1.67e-006

Prediction: Positive if the Bayesian score is above the estimated best cutoff value from minimizing the false positive and false negative rate.
Probability: The esimated probability that the sample is in the positive category. This assumes that the Bayesian score follows a normal distribution and is different from the prediction using a cutoff.
Enrichment: An estimate of enrichment, that is, the increased likelihood (versus random) of this sample being in the category.
Bayesian Score: The standard Laplacian-modified Bayesian score.
Mahalanobis Distance: The Mahalanobis distance (MD) is the distance to the center of the training data. The larger the MD, the less trustworthy the prediction.
Mahalanobis Distance p-value: The p-value gives the fraction of training data with an MD greater than or equal to the one for the given sample, assuming normally distributed data. The smaller the p-value, the less trustworthy the prediciton. For highly non-normal X properties (e.g., fingerprints), the MD p-value is wildly inaccurate.

Structural Similar Compounds

Name	Moricizine	Nisoldipine	Fluticasone
Structure	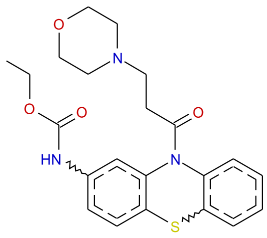	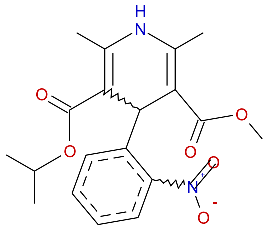	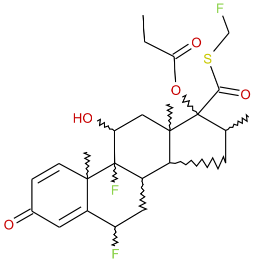
Actual Endpoint	Non-Carcinogen	Non-Carcinogen	Non-Carcinogen
Predicted Endpoint	Non-Carcinogen	Non-Carcinogen	Carcinogen
Distance	0.670	0.687	0.694
Reference	US FDA (Centre for Drug Eval.& Res./Off. Testing & Res.) Sept. 1997	US FDA (Centre for Drug Eval.& Res./Off. Testing & Res.) Sept. 1997	US FDA (Centre for Drug Eval.& Res./Off. Testing & Res.) Sept. 1997

Model Applicability

Unknown features are fingerprint features in the query molecule, but not found in the training set.

1. OPS PC19 out of range. Value: -3.0823. Training min, max, SD, explained variance: -2.8152, 4.6113, 1.185, 0.0147.
2. Unknown FCFP_2 feature: 5: [*][O-]
3. Unknown FCFP_2 feature: -828984032: [*][c](:[*]):[c]([N+](=[*])[*]):c:[*]
4. Unknown FCFP_2 feature: -1338588315: [*]:[c](:[*])[N+](=O)[O-]
5. Unknown FCFP_2 feature: 1872392852: [*][N+](=O)[*]
6. Unknown FCFP_2 feature: 260476081: [*][N+](=[*])[O-]

Feature Contribution

Top features for positive contribution				
Fingerprint	Bit/Smiles	Feature Structure	Score	Carcinogen in training set

FCFP_6	-451043714	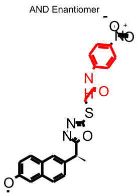 [*]CC(=O)N[c]1:[cH]:[cH]:[cH]:[cH]:[cH]:1	0.676	2 out of 2
FCFP_6	1175665944	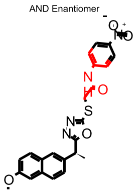 [*]CC(=O)N[c](:[cH]:[*]):[cH]:[*]	0.655	7 out of 12
FCFP_6	-1838187238	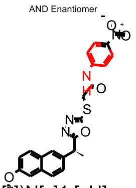 [*]C(=[*])N[c]1:[cH]: [cH]:[*]:[cH]:[cH]:1	0.565	4 out of 7
**Top Features for negative contribution**				
**Fingerprint**	**Bit/Smiles**	**Feature Structure**	**Score**	**Carcinogen in training set**
FCFP_6	1674955425	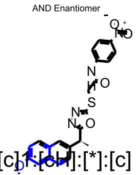 [*]O[c]1:[cH]:[*]:[c] (:[*]):[c](:[cH]:[*]):[cH]:1	-0.719	0 out of 4
FCFP_6	86586436	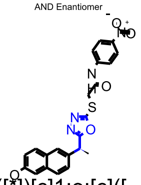 [*]C([*])[c]1:o:[c]([*]):n:n:1	-0.719	0 out of 4

FCFP_6	1514013367	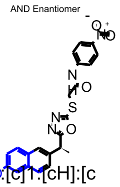<br>[*]:[cH]:[c]1:[cH]:[c<br>](OC):[cH]:[cH]:[c]:<br>1:[*]	-0.423	0 out of 2

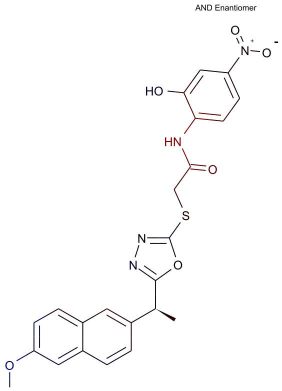

$C_{23}H_{20}N_4O_6S$
Molecular Weight: 480.4931
ALogP: 3.89
Rotatable Bonds: 8
Acceptors: 8
Donors: 2

Model Prediction

Prediction: Carcinogen
Probability: 0.296
Enrichment: 1.007
Bayesian Score: -0.355
Mahalanobis Distance: 17.375
Mahalanobis Distance p-value: 9.56e-013

Prediction: Positive if the Bayesian score is above the estimated best cutoff value from minimizing the false positive and false negative rate.
Probability: The esimated probability that the sample is in the positive category. This assumes that the Bayesian score follows a normal distribution and is different from the prediction using a cutoff.
Enrichment: An estimate of enrichment, that is, the increased likelihood (versus random) of this sample being in the category.
Bayesian Score: The standard Laplacian-modified Bayesian score.
Mahalanobis Distance: The Mahalanobis distance (MD) is the distance to the center of the training data. The larger the MD, the less trustworthy the prediction.
Mahalanobis Distance p-value: The p-value gives the fraction of training data with an MD greater than or equal to the one for the given sample, assuming normally distributed data. The smaller the p-value, the less trustworthy the prediciton. For highly non-normal X properties (e.g., fingerprints), the MD p-value is wildly inaccurate.

Structural Similar Compounds

Name	Sulfasalazine	Bacampicillin	Nimodipine
Structure	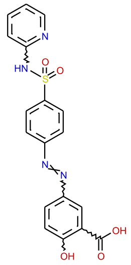	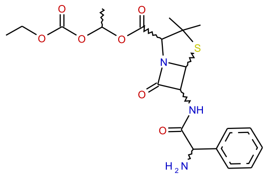	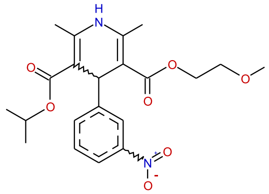
Actual Endpoint	Carcinogen	Non-Carcinogen	Non-Carcinogen
Predicted Endpoint	Carcinogen	Non-Carcinogen	Non-Carcinogen
Distance	0.678	0.735	0.739
Reference	US FDA (Centre for Drug Eval.& Res./Off. Testing & Res.) Sept. 1997	US FDA (Centre for Drug Eval.& Res./Off. Testing & Res.) Sept. 1997	US FDA (Centre for Drug Eval.& Res./Off. Testing & Res.) Sept. 1997

Model Applicability

Unknown features are fingerprint features in the query molecule, but not found in the training set.

1. OPS PC19 out of range. Value: -2.9766. Training min, max, SD, explained variance: -2.8152, 4.6113, 1.185, 0.0147.
2. Unknown FCFP_2 feature: 5: [*][O-]
3. Unknown FCFP_2 feature: -828984032: [*][c](:[*]):[c]([N+](=[*])[*]):c:[*]
4. Unknown FCFP_2 feature: -1338588315: [*]:[c](:[*])[N+](=O)[O-]
5. Unknown FCFP_2 feature: 1872392852: [*][N+](=O)[*]
6. Unknown FCFP_2 feature: 260476081: [*][N+](=[*])[O-]

Feature Contribution

Top features for positive contribution				
**Fingerprint**	**Bit/Smiles**	**Feature Structure**	**Score**	**Carcinogen in training set**

FCFP_6	1175665944	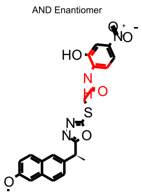 [*]CC(=O)N[c](:[cH]:[*]):[cH]:[*]	0.655	7 out of 12
FCFP_6	-328193675	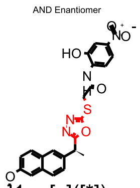 [*]S[c]1:o:[c]([*]):n :n:1	0.460	1 out of 1
FCFP_6	-1947166985	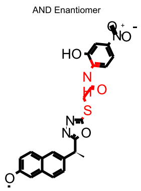 [*]SCC(=O)N[c](:[*]): [*]	0.460	1 out of 1
**Top Features for negative contribution**				
**Fingerprint**	**Bit/Smiles**	**Feature Structure**	**Score**	**Carcinogen in training set**
FCFP_6	1674955425	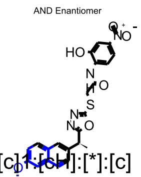 [*]O[c]1:[cH]:[*]:[c] (:[*]):[c](:[cH]:[*]):[cH]:1	-0.719	0 out of 4
FCFP_6	86586436	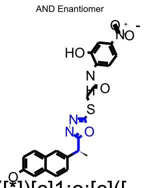 [*]C([*])[c]1:o:[c]([*]):n:n:1	-0.719	0 out of 4

FCFP_6	1514013367	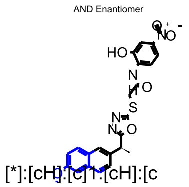 [*]:[cH]:[c]1:[cH]:[c](OC):[cH]:[cH]:[c]:1:[*]	-0.423	0 out of 2

Sorafenib

TOPKAT_Mouse_Male_FDA_None_vs_Carcinogen

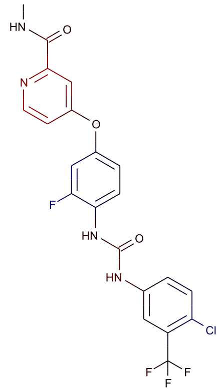

$C_{21}H_{15}ClF_4N_4O_3$
Molecular Weight: 482.81541
ALogP: 4.381
Rotatable Bonds: 6
Acceptors: 4
Donors: 3

Model Prediction

Prediction: Carcinogen
Probability: 0.356
Enrichment: 1.210
Bayesian Score: 1.698
Mahalanobis Distance: 20.300
Mahalanobis Distance p-value: 1.44e-019

Prediction: Positive if the Bayesian score is above the estimated best cutoff value from minimizing the false positive and false negative rate.
Probability: The esimated probability that the sample is in the positive category. This assumes that the Bayesian score follows a normal distribution and is different from the prediction using a cutoff.
Enrichment: An estimate of enrichment, that is, the increased likelihood (versus random) of this sample being in the category.
Bayesian Score: The standard Laplacian-modified Bayesian score.
Mahalanobis Distance: The Mahalanobis distance (MD) is the distance to the center of the training data. The larger the MD, the less trustworthy the prediction.
Mahalanobis Distance p-value: The p-value gives the fraction of training data with an MD greater than or equal to the one for the given sample, assuming normally distributed data. The smaller the p-value, the less trustworthy the prediciton. For highly non-normal X properties (e.g., fingerprints), the MD p-value is wildly inaccurate.

Structural Similar Compounds

Name	Glyburide	Glimepride	Fluvastatin
Structure	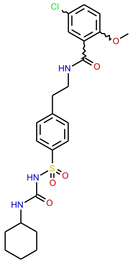	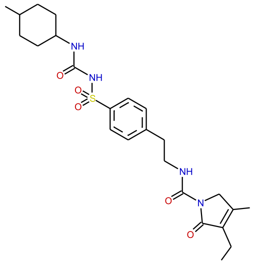	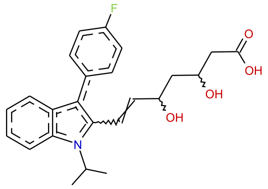
Actual Endpoint	Non-Carcinogen	Carcinogen	Non-Carcinogen
Predicted Endpoint	Non-Carcinogen	Carcinogen	Non-Carcinogen
Distance	0.595	0.603	0.624
Reference	US FDA (Centre for Drug Eval.& Res./Off. Testing & Res.) Sept. 1997	US FDA (Centre for Drug Eval.& Res./Off. Testing & Res.) Sept. 1997	US FDA (Centre for Drug Eval.& Res./Off. Testing & Res.) Sept. 1997

Model Applicability

Unknown features are fingerprint features in the query molecule, but not found in the training set.

1. All properties and OPS components are within expected ranges.

Feature Contribution

Top features for positive contribution				
**Fingerprint**	**Bit/Smiles**	**Feature Structure**	**Score**	**Carcinogen in training set**
FCFP_6	71953198	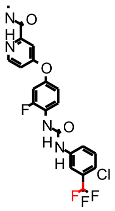 [*]C([*])([*])F	0.612	12 out of 23

FCFP_6	-1838187238	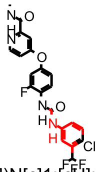 [*]C(=[*])N[c]1:[cH]:[cH]:[*]:[cH]:[cH]:1	0.565	4 out of 7
FCFP_6	140656626	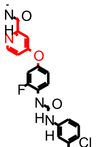 [*]O[c]1:[cH]:[cH]:n:[c](:[cH]:1)C(=[*])[*]	0.460	1 out of 1

Top Features for negative contribution

Fingerprint	Bit/Smiles	Feature Structure	Score	Carcinogen in training set
FCFP_6	2104062943	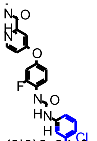 [*]C([*])([*])[c]1:[cH]:[*]:[cH]:[cH]:[c]:1Cl	-1.006	1 out of 17
FCFP_6	1783756416	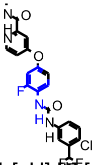 [*]N[c]1:[cH]:[*]:[c]([*]):[cH]:[c]:1Cl	-0.719	0 out of 4
FCFP_6	1677789694	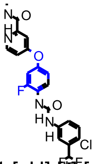 [*]O[c]1:[cH]:[*]:[c]([*]):[c](F):[cH]:1	-0.719	0 out of 4

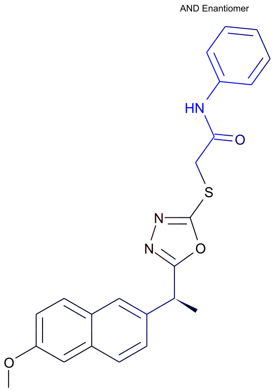

$C_{23}H_{21}N_3O_3S$
Molecular Weight: 419.49614
ALogP: 4.238
Rotatable Bonds: 7
Acceptors: 5
Donors: 1

Model Prediction

Prediction: Single-Carcinogen
Probability: 0.153
Enrichment: 0.507
Bayesian Score: -12.046
Mahalanobis Distance: 16.332
Mahalanobis Distance p-value: 2.27e-007

Prediction: Positive if the Bayesian score is above the estimated best cutoff value from minimizing the false positive and false negative rate.
Probability: The esimated probability that the sample is in the positive category. This assumes that the Bayesian score follows a normal distribution and is different from the prediction using a cutoff.
Enrichment: An estimate of enrichment, that is, the increased likelihood (versus random) of this sample being in the category.
Bayesian Score: The standard Laplacian-modified Bayesian score.
Mahalanobis Distance: The Mahalanobis distance (MD) is the distance to the center of the training data. The larger the MD, the less trustworthy the prediction.
Mahalanobis Distance p-value: The p-value gives the fraction of training data with an MD greater than or equal to the one for the given sample, assuming normally distributed data. The smaller the p-value, the less trustworthy the prediciton. For highly non-normal X properties (e.g., fingerprints), the MD p-value is wildly inaccurate.

Structural Similar Compounds

Name	Lovastatin	Simvastatin	Lansoprazole
Structure	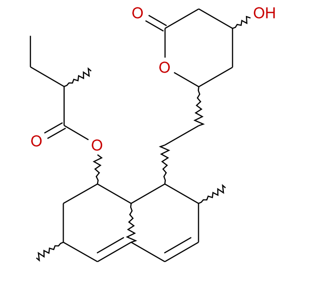	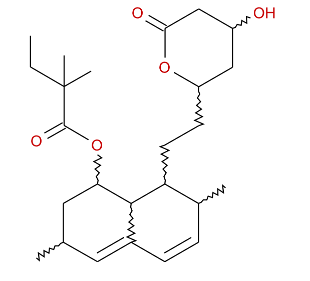	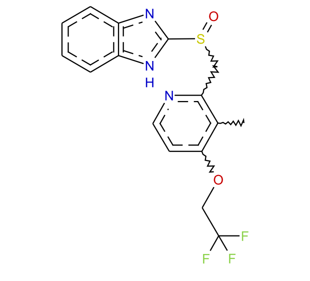
Actual Endpoint	Multiple-Carcinogen	Multiple-Carcinogen	Single-Carcinogen
Predicted Endpoint	Multiple-Carcinogen	Multiple-Carcinogen	Single-Carcinogen
Distance	0.579	0.594	0.632
Reference	US FDA (Centre for Drug Eval.& Res./Off. Testing & Res.) Sept. 1997	US FDA (Centre for Drug Eval.& Res./Off. Testing & Res.) Sept. 1997	US FDA (Centre for Drug Eval.& Res./Off. Testing & Res.) Sept. 1997

Model Applicability

Unknown features are fingerprint features in the query molecule, but not found in the training set.

1. OPS PC2 out of range. Value: 4.8785. Training min, max, SD, explained variance: -5.2888, 4.2744, 2.566, 0.1229.

Feature Contribution

Top features for positive contribution

Fingerprint	Bit/Smiles	Feature Structure	Score	Multiple-Carcinogen in training set
FCFP_12	-328193675	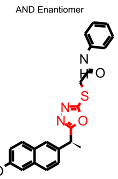 [*]S[c]1:o:[c]([*]):n :n:1	0.400	1 out of 1

FCFP_12	277509858	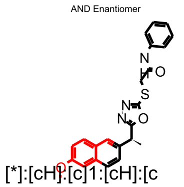 [*]:[cH]:[c]1:[cH]:[cH]:[c](OC):[cH]:[c]:1:[*]	0.400	1 out of 1
FCFP_12	-1410079687	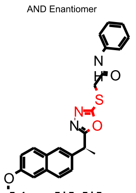 [*]S[c]1:o:[*]:[*]:n:1	0.395	2 out of 3
**Top Features for negative contribution**				
**Fingerprint**	**Bit/Smiles**	**Feature Structure**	**Score**	**Multiple-Carcinogen in training set**
FCFP_12	1294255210	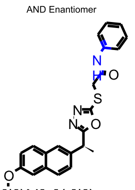 [*]C(=[*])N[c](:[*]):[*]	-1.626	0 out of 12
FCFP_12	1175665944	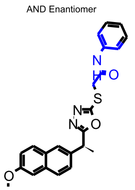 [*]CC(=O)N[c](:[cH]:[*]):[cH]:[*]	-1.219	0 out of 7
FCFP_12	590925877	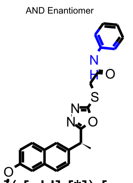 [*]N[c](:[cH]:[*]):[cH]:[*]	-0.998	1 out of 13

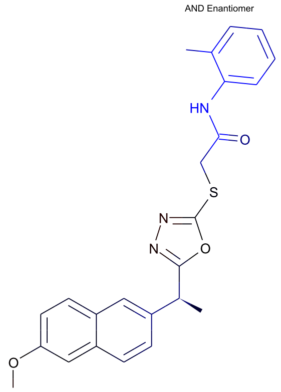

$C_{24}H_{23}N_3O_3S$
Molecular Weight: 433.52272
ALogP: 4.724
Rotatable Bonds: 7
Acceptors: 5
Donors: 1

Structural Similar Compounds

Name	Simvastatin	Lovastatin	Lansoprazole
Structure	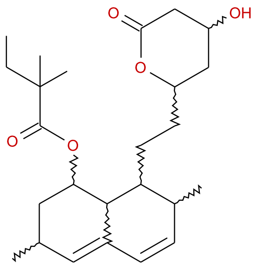	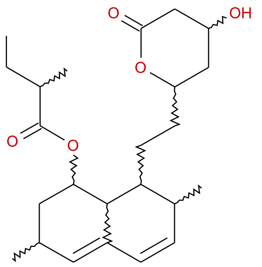	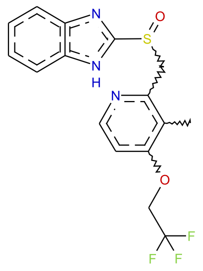
Actual Endpoint	Multiple-Carcinogen	Multiple-Carcinogen	Single-Carcinogen
Predicted Endpoint	Multiple-Carcinogen	Multiple-Carcinogen	Single-Carcinogen
Distance	0.579	0.587	0.665
Reference	US FDA (Centre for Drug Eval.& Res./Off. Testing & Res.) Sept. 1997	US FDA (Centre for Drug Eval.& Res./Off. Testing & Res.) Sept. 1997	US FDA (Centre for Drug Eval.& Res./Off. Testing & Res.) Sept. 1997

Model Prediction

Prediction: Single-Carcinogen
Probability: 0.147
Enrichment: 0.488
Bayesian Score: -13.822
Mahalanobis Distance: 17.096
Mahalanobis Distance p-value: 5.87e-008

Prediction: Positive if the Bayesian score is above the estimated best cutoff value from minimizing the false positive and false negative rate.
Probability: The esimated probability that the sample is in the positive category. This assumes that the Bayesian score follows a normal distribution and is different from the prediction using a cutoff.
Enrichment: An estimate of enrichment, that is, the increased likelihood (versus random) of this sample being in the category.
Bayesian Score: The standard Laplacian-modified Bayesian score.
Mahalanobis Distance: The Mahalanobis distance (MD) is the distance to the center of the training data. The larger the MD, the less trustworthy the prediction.
Mahalanobis Distance p-value: The p-value gives the fraction of training data with an MD greater than or equal to the one for the given sample, assuming normally distributed data. The smaller the p-value, the less trustworthy the prediciton. For highly non-normal X properties (e.g., fingerprints), the MD p-value is wildly inaccurate.

Model Applicability

Unknown features are fingerprint features in the query molecule, but not found in the training set.

1. OPS PC2 out of range. Value: 5.3258. Training min, max, SD, explained variance: -5.2888, 4.2744, 2.566, 0.1229.
2. OPS PC6 out of range. Value: -3.289. Training min, max, SD, explained variance: -3.1949, 6.05, 1.653, 0.0510.

Feature Contribution

Top features for positive contribution

Fingerprint	Bit/Smiles	Feature Structure	Score	Multiple-Carcinogen in training set
FCFP_12	-328193675	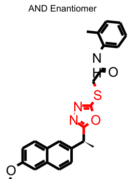 [*]S[c]1:o:[c]([*]):n:n:1	0.400	1 out of 1

FCFP_12	277509858	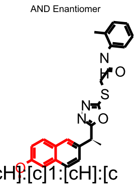 [*]:[cH]:[c]1:[cH]:[cH]:[c](OC):[cH]:[c]:1:[*]	0.400	1 out of 1
FCFP_12	-1410079687	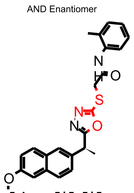 [*]S[c]1:o:[*]:[*]:n:1	0.395	2 out of 3

Top Features for negative contribution

Fingerprint	Bit/Smiles	Feature Structure	Score	Multiple-Carcinogen in training set
FCFP_12	1294255210	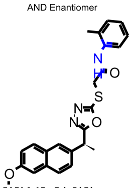 [*]C(=[*])N[c](:[*]):[*]	-1.626	0 out of 12
FCFP_12	1175665944	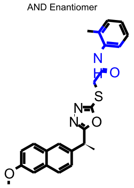 [*]CC(=O)N[c](:[cH]:[*]):[cH]:[*]	-1.219	0 out of 7
FCFP_12	590925877	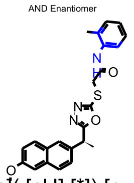 [*]N[c](:[cH]:[*]):[cH]:[*]	-0.998	1 out of 13

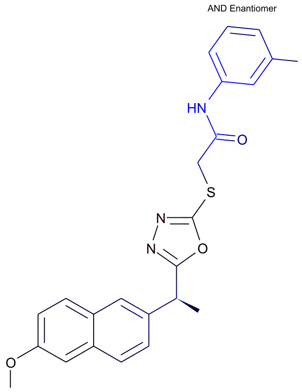

$C_{24}H_{23}N_3O_3S$
Molecular Weight: 433.52272
ALogP: 4.724
Rotatable Bonds: 7
Acceptors: 5
Donors: 1

Model Prediction

Prediction: Single-Carcinogen
Probability: 0.155
Enrichment: 0.514
Bayesian Score: -12.969
Mahalanobis Distance: 17.093
Mahalanobis Distance p-value: 5.9e-008

Prediction: Positive if the Bayesian score is above the estimated best cutoff value from minimizing the false positive and false negative rate.
Probability: The esimated probability that the sample is in the positive category. This assumes that the Bayesian score follows a normal distribution and is different from the prediction using a cutoff.
Enrichment: An estimate of enrichment, that is, the increased likelihood (versus random) of this sample being in the category.
Bayesian Score: The standard Laplacian-modified Bayesian score.
Mahalanobis Distance: The Mahalanobis distance (MD) is the distance to the center of the training data. The larger the MD, the less trustworthy the prediction.
Mahalanobis Distance p-value: The p-value gives the fraction of training data with an MD greater than or equal to the one for the given sample, assuming normally distributed data. The smaller the p-value, the less trustworthy the prediciton. For highly non-normal X properties (e.g., fingerprints), the MD p-value is wildly inaccurate.

Structural Similar Compounds

Name	Simvastatin	Lovastatin	Lansoprazole
Structure	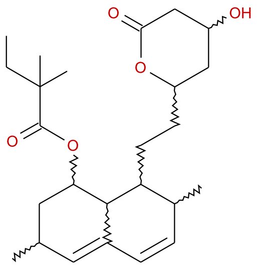	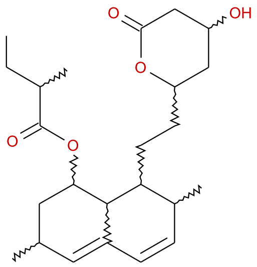	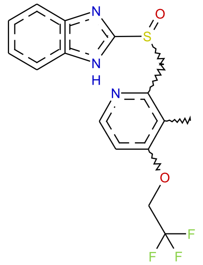
Actual Endpoint	Multiple-Carcinogen	Multiple-Carcinogen	Single-Carcinogen
Predicted Endpoint	Multiple-Carcinogen	Multiple-Carcinogen	Single-Carcinogen
Distance	0.579	0.587	0.665
Reference	US FDA (Centre for Drug Eval.& Res./Off. Testing & Res.) Sept. 1997	US FDA (Centre for Drug Eval.& Res./Off. Testing & Res.) Sept. 1997	US FDA (Centre for Drug Eval.& Res./Off. Testing & Res.) Sept. 1997

Model Applicability

Unknown features are fingerprint features in the query molecule, but not found in the training set.

1. OPS PC2 out of range. Value: 4.8195. Training min, max, SD, explained variance: -5.2888, 4.2744, 2.566, 0.1229.
2. OPS PC6 out of range. Value: -3.414. Training min, max, SD, explained variance: -3.1949, 6.05, 1.653, 0.0510.

Feature Contribution

Top features for positive contribution

Fingerprint	Bit/Smiles	Feature Structure	Score	Multiple-Carcinogen in training set
FCFP_12	277509858	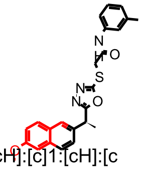 AND Enantiomer [*]:[cH]:[c]1:[cH]:[cH]:[c](OC):[cH]:[c]:1:[*]	0.400	1 out of 1

FCFP_12	-328193675	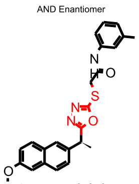 [*]S[c]1:o:[c]([*]):n:n:1	0.400	1 out of 1
FCFP_12	-1410079687	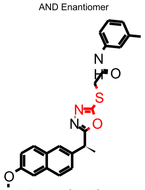 [*]S[c]1:o:[*]:[*]:n:1	0.395	2 out of 3

Top Features for negative contribution

**Fingerprint**	**Bit/Smiles**	**Feature Structure**	**Score**	**Multiple-Carcinogen in training set**
FCFP_12	1294255210	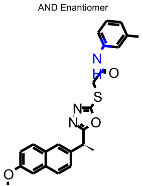 [*]C(=[*])N[c](:[*]):[*]	-1.626	0 out of 12
FCFP_12	1175665944	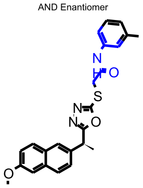 [*]CC(=O)N[c](:[cH]:[*]):[cH]:[*]	-1.219	0 out of 7
FCFP_12	590925877	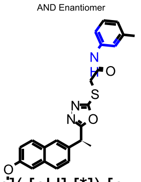 [*]N[c](:[cH]:[*]):[cH]:[*]	-0.998	1 out of 13

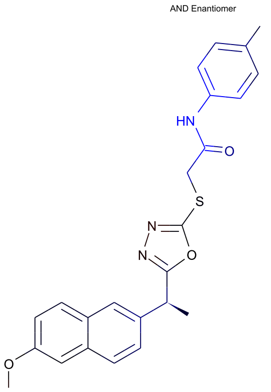

$C_{24}H_{23}N_3O_3S$
Molecular Weight: 433.52272
ALogP: 4.724
Rotatable Bonds: 7
Acceptors: 5
Donors: 1

Structural Similar Compounds

Name	Simvastatin	Lovastatin	Lansoprazole
Structure	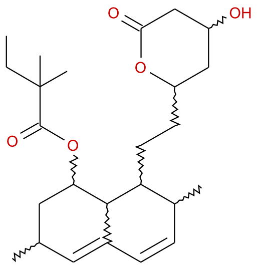	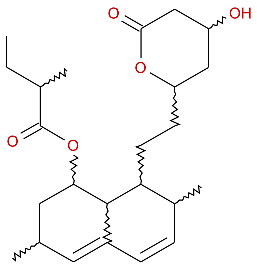	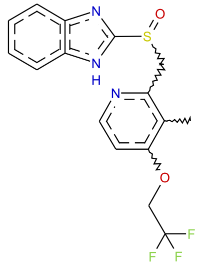
Actual Endpoint	Multiple-Carcinogen	Multiple-Carcinogen	Single-Carcinogen
Predicted Endpoint	Multiple-Carcinogen	Multiple-Carcinogen	Single-Carcinogen
Distance	0.579	0.587	0.668
Reference	US FDA (Centre for Drug Eval.& Res./Off. Testing & Res.) Sept. 1997	US FDA (Centre for Drug Eval.& Res./Off. Testing & Res.) Sept. 1997	US FDA (Centre for Drug Eval.& Res./Off. Testing & Res.) Sept. 1997

Model Prediction

Prediction: Single-Carcinogen
Probability: 0.153
Enrichment: 0.509
Bayesian Score: -12.181
Mahalanobis Distance: 16.248
Mahalanobis Distance p-value: 2.64e-007

Prediction: Positive if the Bayesian score is above the estimated best cutoff value from minimizing the false positive and false negative rate.
Probability: The esimated probability that the sample is in the positive category. This assumes that the Bayesian score follows a normal distribution and is different from the prediction using a cutoff.
Enrichment: An estimate of enrichment, that is, the increased likelihood (versus random) of this sample being in the category.
Bayesian Score: The standard Laplacian-modified Bayesian score.
Mahalanobis Distance: The Mahalanobis distance (MD) is the distance to the center of the training data. The larger the MD, the less trustworthy the prediction.
Mahalanobis Distance p-value: The p-value gives the fraction of training data with an MD greater than or equal to the one for the given sample, assuming normally distributed data. The smaller the p-value, the less trustworthy the prediciton. For highly non-normal X properties (e.g., fingerprints), the MD p-value is wildly inaccurate.

Model Applicability

Unknown features are fingerprint features in the query molecule, but not found in the training set.

1. OPS PC2 out of range. Value: 4.3669. Training min, max, SD, explained variance: -5.2888, 4.2744, 2.566, 0.1229.
2. OPS PC6 out of range. Value: -3.3379. Training min, max, SD, explained variance: -3.1949, 6.05, 1.653, 0.0510.

Feature Contribution

Top features for positive contribution

Fingerprint	Bit/Smiles	Feature Structure	Score	Multiple-Carcinogen in training set
FCFP_12	-328193675	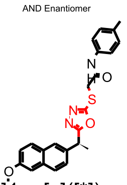 [*]S[c]1:o:[c]([*]):n:n:1	0.400	1 out of 1

FCFP_12	277509858	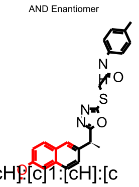 [*]:[cH]:[c]1:[cH]:[cH]:[c](OC):[cH]:[c]:1:[*]	0.400	1 out of 1
FCFP_12	-1410079687	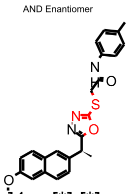 [*]S[c]1:o:[*]:[*]:n:1	0.395	2 out of 3
**Top Features for negative contribution**				
**Fingerprint**	**Bit/Smiles**	**Feature Structure**	**Score**	**Multiple-Carcinogen in training set**
FCFP_12	1294255210	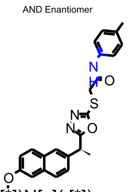 [*]C(=[*])N[c](:[*]):[*]	-1.626	0 out of 12
FCFP_12	1175665944	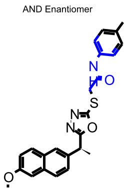 [*]CC(=O)N[c](:[cH]:[*]):[cH]:[*]	-1.219	0 out of 7
FCFP_12	590925877	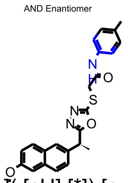 [*]N[c](:[cH]:[*]):[cH]:[*]	-0.998	1 out of 13

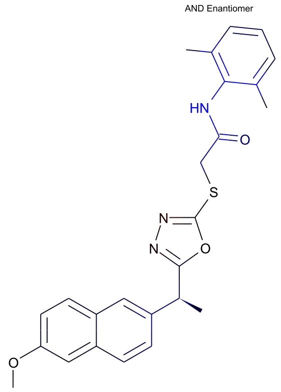

$C_{25}H_{25}N_3O_3S$
Molecular Weight: 447.5493
ALogP: 5.21
Rotatable Bonds: 7
Acceptors: 5
Donors: 1

Model Prediction

Prediction: Single-Carcinogen
Probability: 0.150
Enrichment: 0.498
Bayesian Score: -11.475
Mahalanobis Distance: 17.153
Mahalanobis Distance p-value: 5.3e-008

Prediction: Positive if the Bayesian score is above the estimated best cutoff value from minimizing the false positive and false negative rate.
Probability: The esimated probability that the sample is in the positive category. This assumes that the Bayesian score follows a normal distribution and is different from the prediction using a cutoff.
Enrichment: An estimate of enrichment, that is, the increased likelihood (versus random) of this sample being in the category.
Bayesian Score: The standard Laplacian-modified Bayesian score.
Mahalanobis Distance: The Mahalanobis distance (MD) is the distance to the center of the training data. The larger the MD, the less trustworthy the prediction.
Mahalanobis Distance p-value: The p-value gives the fraction of training data with an MD greater than or equal to the one for the given sample, assuming normally distributed data. The smaller the p-value, the less trustworthy the prediciton. For highly non-normal X properties (e.g., fingerprints), the MD p-value is wildly inaccurate.

Structural Similar Compounds

Name	Simvastatin	Lovastatin	Lansoprazole
Structure	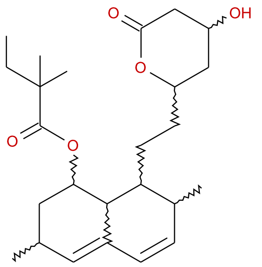	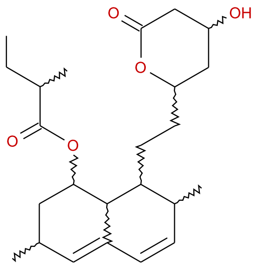	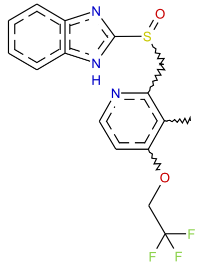
Actual Endpoint	Multiple-Carcinogen	Multiple-Carcinogen	Single-Carcinogen
Predicted Endpoint	Multiple-Carcinogen	Multiple-Carcinogen	Single-Carcinogen
Distance	0.590	0.616	0.704
Reference	US FDA (Centre for Drug Eval.& Res./Off. Testing & Res.) Sept. 1997	US FDA (Centre for Drug Eval.& Res./Off. Testing & Res.) Sept. 1997	US FDA (Centre for Drug Eval.& Res./Off. Testing & Res.) Sept. 1997

Model Applicability

Unknown features are fingerprint features in the query molecule, but not found in the training set.

1. OPS PC2 out of range. Value: 4.4526. Training min, max, SD, explained variance: -5.2888, 4.2744, 2.566, 0.1229.

Feature Contribution

Top features for positive contribution

Fingerprint	Bit/Smiles	Feature Structure	Score	Multiple-Carcinogen in training set
FCFP_12	277509858	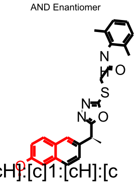 [*]:[cH]:[c]1:[cH]:[cH]:[c](OC):[cH]:[c]:1:[*]	0.400	1 out of 1

FCFP_12	-328193675	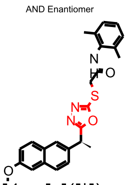 [*]S[c]1:o:[c]([*]):n :n:1	0.400	1 out of 1
FCFP_12	-1410079687	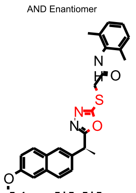 [*]S[c]1:o:[*]:[*]:n: 1	0.395	2 out of 3
**Top Features for negative contribution**				
**Fingerprint**	**Bit/Smiles**	**Feature Structure**	**Score**	**Multiple-Carcinogen in training set**
FCFP_12	1294255210	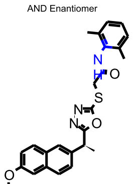 [*]C(=[*])N[c](:[*]): [*]	-1.626	0 out of 12
FCFP_12	1175665944	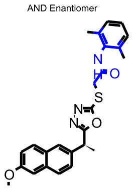 [*]CC(=O)N[c](:[cH]:[*]):[cH]:[*]	-1.219	0 out of 7
FCFP_12	590925877	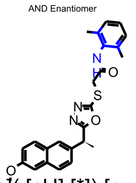 [*]N[c](:[cH]:[*]):[c H]:[*]	-0.998	1 out of 13

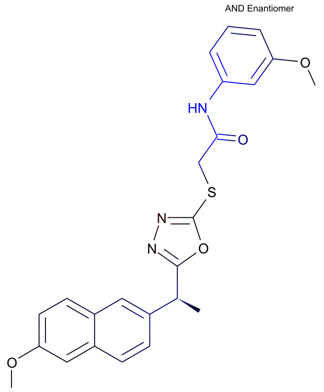

$C_{24}H_{23}N_3O_4S$
Molecular Weight: 449.52212
ALogP: 4.221
Rotatable Bonds: 8
Acceptors: 6
Donors: 1

Model Prediction

Prediction: Single-Carcinogen
Probability: 0.151
Enrichment: 0.500
Bayesian Score: -11.642
Mahalanobis Distance: 16.687
Mahalanobis Distance p-value: 1.21e-007

Prediction: Positive if the Bayesian score is above the estimated best cutoff value from minimizing the false positive and false negative rate.
Probability: The esimated probability that the sample is in the positive category. This assumes that the Bayesian score follows a normal distribution and is different from the prediction using a cutoff.
Enrichment: An estimate of enrichment, that is, the increased likelihood (versus random) of this sample being in the category.
Bayesian Score: The standard Laplacian-modified Bayesian score.
Mahalanobis Distance: The Mahalanobis distance (MD) is the distance to the center of the training data. The larger the MD, the less trustworthy the prediction.
Mahalanobis Distance p-value: The p-value gives the fraction of training data with an MD greater than or equal to the one for the given sample, assuming normally distributed data. The smaller the p-value, the less trustworthy the prediciton. For highly non-normal X properties (e.g., fingerprints), the MD p-value is wildly inaccurate.

Structural Similar Compounds

Name	Lovastatin	Simvastatin	Bicalutamide
Structure	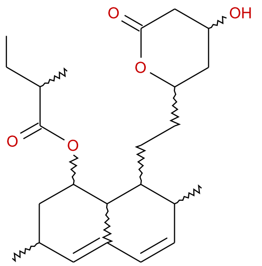	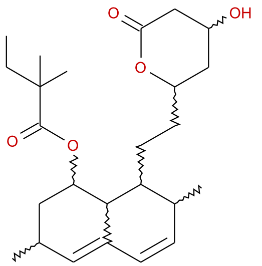	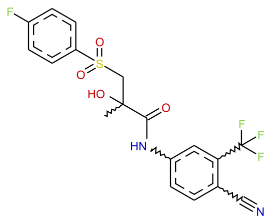
Actual Endpoint	Multiple-Carcinogen	Multiple-Carcinogen	Single-Carcinogen
Predicted Endpoint	Multiple-Carcinogen	Multiple-Carcinogen	Single-Carcinogen
Distance	0.658	0.659	0.739
Reference	US FDA (Centre for Drug Eval.& Res./Off. Testing & Res.) Sept. 1997	US FDA (Centre for Drug Eval.& Res./Off. Testing & Res.) Sept. 1997	US FDA (Centre for Drug Eval.& Res./Off. Testing & Res.) Sept. 1997

Model Applicability

Unknown features are fingerprint features in the query molecule, but not found in the training set.

1. OPS PC2 out of range. Value: 4.5216. Training min, max, SD, explained variance: -5.2888, 4.2744, 2.566, 0.1229.

Feature Contribution

Top features for positive contribution

Fingerprint	Bit/Smiles	Feature Structure	Score	Multiple-Carcinogen in training set
FCFP_12	277509858	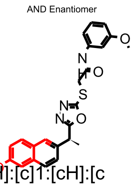 [*]:[cH]:[c]1:[cH]:[cH]:[c](OC):[cH]:[c]:1:[*]	0.400	1 out of 1

FCFP_12	-328193675	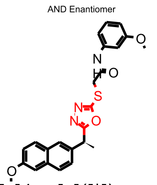 [*]S[c]1:o:[c]([*]):n:n:1	0.400	1 out of 1
FCFP_12	-1410079687	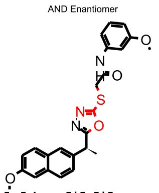 [*]S[c]1:o:[*]:[*]:n:1	0.395	2 out of 3

Top Features for negative contribution

Fingerprint	Bit/Smiles	Feature Structure	Score	Multiple-Carcinogen in training set
FCFP_12	1294255210	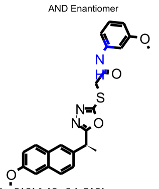 [*]C(=[*])N[c](:[*]):[*]	-1.626	0 out of 12
FCFP_12	1175665944	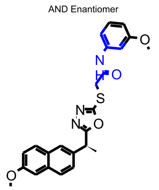 [*]CC(=O)N[c](:[cH]:[*]):[cH]:[*]	-1.219	0 out of 7
FCFP_12	590925877	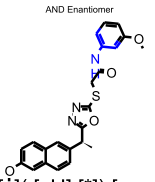 [*]N[c](:[cH]:[*]):[cH]:[*]	-0.998	1 out of 13

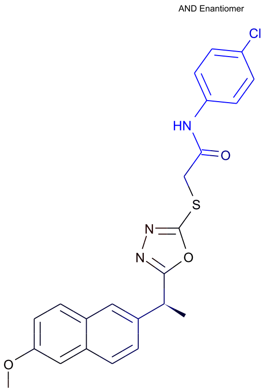

$C_{23}H_{20}ClN_3O_3S$
Molecular Weight: 453.9412
ALogP: 4.902
Rotatable Bonds: 7
Acceptors: 5
Donors: 1

Model Prediction

Prediction: Single-Carcinogen
Probability: 0.132
Enrichment: 0.438
Bayesian Score: -15.544
Mahalanobis Distance: 16.581
Mahalanobis Distance p-value: 1.46e-007

Prediction: Positive if the Bayesian score is above the estimated best cutoff value from minimizing the false positive and false negative rate.
Probability: The esimated probability that the sample is in the positive category. This assumes that the Bayesian score follows a normal distribution and is different from the prediction using a cutoff.
Enrichment: An estimate of enrichment, that is, the increased likelihood (versus random) of this sample being in the category.
Bayesian Score: The standard Laplacian-modified Bayesian score.
Mahalanobis Distance: The Mahalanobis distance (MD) is the distance to the center of the training data. The larger the MD, the less trustworthy the prediction.
Mahalanobis Distance p-value: The p-value gives the fraction of training data with an MD greater than or equal to the one for the given sample, assuming normally distributed data. The smaller the p-value, the less trustworthy the prediciton. For highly non-normal X properties (e.g., fingerprints), the MD p-value is wildly inaccurate.

Structural Similar Compounds

Name	Simvastatin	Lovastatin	Lansoprazole
Structure	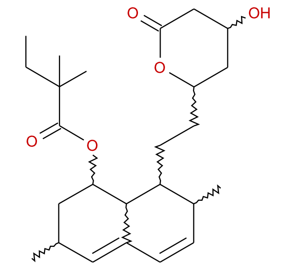	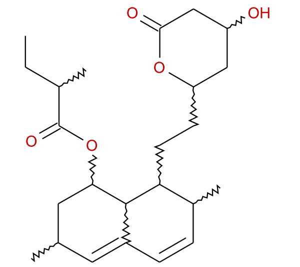	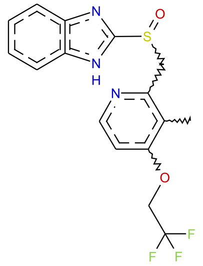
Actual Endpoint	Multiple-Carcinogen	Multiple-Carcinogen	Single-Carcinogen
Predicted Endpoint	Multiple-Carcinogen	Multiple-Carcinogen	Single-Carcinogen
Distance	0.599	0.617	0.699
Reference	US FDA (Centre for Drug Eval.& Res./Off. Testing & Res.) Sept. 1997	US FDA (Centre for Drug Eval.& Res./Off. Testing & Res.) Sept. 1997	US FDA (Centre for Drug Eval.& Res./Off. Testing & Res.) Sept. 1997

Model Applicability

Unknown features are fingerprint features in the query molecule, but not found in the training set.

1. OPS PC2 out of range. Value: 4.8826. Training min, max, SD, explained variance: -5.2888, 4.2744, 2.566, 0.1229.
2. OPS PC5 out of range. Value: 4.4391. Training min, max, SD, explained variance: -3.5268, 3.8048, 1.733, 0.0560.

Feature Contribution

Top features for positive contribution

Fingerprint	Bit/Smiles	Feature Structure	Score	Multiple-Carcinogen in training set
FCFP_12	-328193675	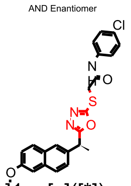 [*]S[c]1:o:[c]([*]):n:n:1	0.400	1 out of 1

FCFP_12	277509858	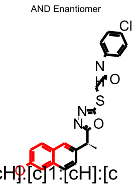 [*]:[cH]:[c]1:[cH]:[cH]:[c](OC):[cH]:[c]:1:[*]	0.400	1 out of 1
FCFP_12	-1410079687	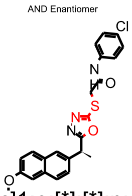 [*]S[c]1:o:[*]:[*]:n:1	0.395	2 out of 3

Top Features for negative contribution

Fingerprint	Bit/Smiles	Feature Structure	Score	Multiple-Carcinogen in training set
FCFP_12	1294255210	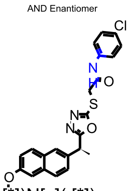 [*]C(=[*])N[c](:[*]):[*]	-1.626	0 out of 12
FCFP_12	1175665944	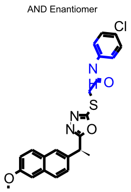 [*]CC(=O)N[c](:[cH]:[*]):[cH]:[*]	-1.219	0 out of 7
FCFP_12	590925877	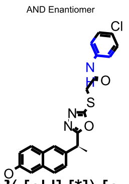 [*]N[c](:[cH]:[*]):[cH]:[*]	-0.998	1 out of 13

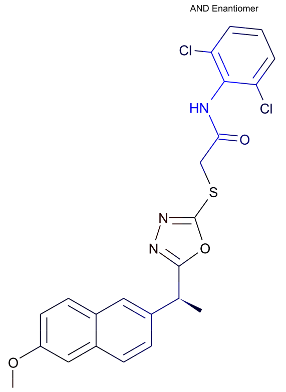

$C_{23}H_{19}Cl_2N_3O_3S$
Molecular Weight: 488.38626
ALogP: 5.567
Rotatable Bonds: 7
Acceptors: 5
Donors: 1

Structural Similar Compounds

Name	Simvastatin	Lovastatin	Lansoprazole
Structure	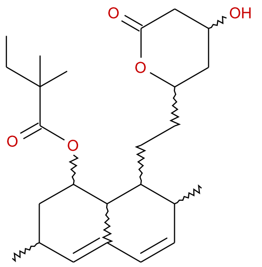	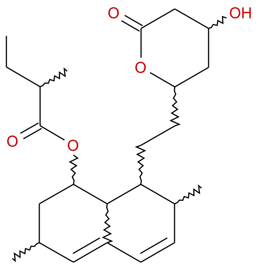	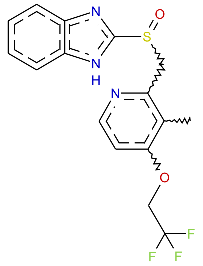
Actual Endpoint	Multiple-Carcinogen	Multiple-Carcinogen	Single-Carcinogen
Predicted Endpoint	Multiple-Carcinogen	Multiple-Carcinogen	Single-Carcinogen
Distance	0.653	0.685	0.770
Reference	US FDA (Centre for Drug Eval.& Res./Off. Testing & Res.) Sept. 1997	US FDA (Centre for Drug Eval.& Res./Off. Testing & Res.) Sept. 1997	US FDA (Centre for Drug Eval.& Res./Off. Testing & Res.) Sept. 1997

Model Prediction

Prediction: Single-Carcinogen
Probability: 0.155
Enrichment: 0.514
Bayesian Score: -12.416
Mahalanobis Distance: 19.840
Mahalanobis Distance p-value: 5.49e-010

Prediction: Positive if the Bayesian score is above the estimated best cutoff value from minimizing the false positive and false negative rate.
Probability: The esimated probability that the sample is in the positive category. This assumes that the Bayesian score follows a normal distribution and is different from the prediction using a cutoff.
Enrichment: An estimate of enrichment, that is, the increased likelihood (versus random) of this sample being in the category.
Bayesian Score: The standard Laplacian-modified Bayesian score.
Mahalanobis Distance: The Mahalanobis distance (MD) is the distance to the center of the training data. The larger the MD, the less trustworthy the prediction.
Mahalanobis Distance p-value: The p-value gives the fraction of training data with an MD greater than or equal to the one for the given sample, assuming normally distributed data. The smaller the p-value, the less trustworthy the prediciton. For highly non-normal X properties (e.g., fingerprints), the MD p-value is wildly inaccurate.

Model Applicability

Unknown features are fingerprint features in the query molecule, but not found in the training set.

1. OPS PC2 out of range. Value: 4.9692. Training min, max, SD, explained variance: -5.2888, 4.2744, 2.566, 0.1229.

Feature Contribution

Top features for positive contribution

Fingerprint	Bit/Smiles	Feature Structure	Score	Multiple-Carcinogen in training set
FCFP_12	-328193675	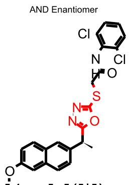 [*]S[c]1:o:[c]([*]):n :n:1	0.400	1 out of 1

FCFP_12	277509858	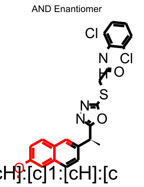 [*]:[cH]:[c]1:[cH]:[cH]:[c](OC):[cH]:[c]:1:[*]	0.400	1 out of 1
FCFP_12	-1410079687	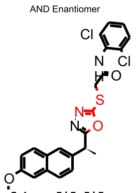 [*]S[c]1:o:[*]:[*]:n:1	0.395	2 out of 3

Top Features for negative contribution

Fingerprint	Bit/Smiles	Feature Structure	Score	Multiple-Carcinogen in training set
FCFP_12	1294255210	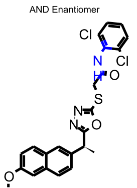 [*]C(=[*])N[c](:[*]):[*]	-1.626	0 out of 12
FCFP_12	1175665944	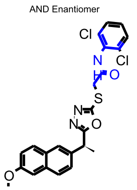 [*]CC(=O)N[c](:[cH]:[*]):[cH]:[*]	-1.219	0 out of 7
FCFP_12	590925877	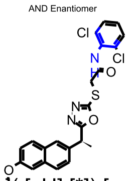 [*]N[c](:[cH]:[*]):[cH]:[*]	-0.998	1 out of 13

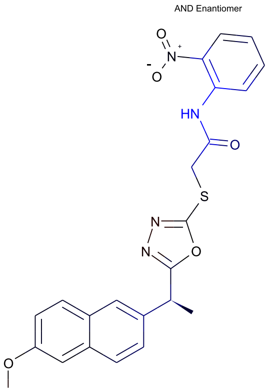

$C_{23}H_{20}N_4O_5S$
Molecular Weight: 464.4937
ALogP: 4.132
Rotatable Bonds: 8
Acceptors: 7
Donors: 1

Model Prediction

Prediction: Single-Carcinogen
Probability: 0.147
Enrichment: 0.487
Bayesian Score: -10.440
Mahalanobis Distance: 16.763
Mahalanobis Distance p-value: 1.06e-007

Prediction: Positive if the Bayesian score is above the estimated best cutoff value from minimizing the false positive and false negative rate.
Probability: The esimated probability that the sample is in the positive category. This assumes that the Bayesian score follows a normal distribution and is different from the prediction using a cutoff.
Enrichment: An estimate of enrichment, that is, the increased likelihood (versus random) of this sample being in the category.
Bayesian Score: The standard Laplacian-modified Bayesian score.
Mahalanobis Distance: The Mahalanobis distance (MD) is the distance to the center of the training data. The larger the MD, the less trustworthy the prediction.
Mahalanobis Distance p-value: The p-value gives the fraction of training data with an MD greater than or equal to the one for the given sample, assuming normally distributed data. The smaller the p-value, the less trustworthy the prediciton. For highly non-normal X properties (e.g., fingerprints), the MD p-value is wildly inaccurate.

Structural Similar Compounds

Name	Lovastatin	Simvastatin	Bicalutamide
Structure	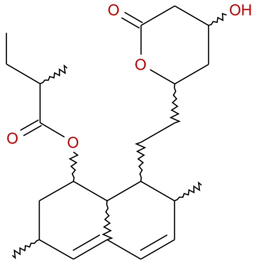	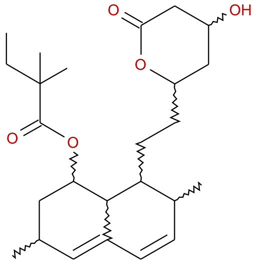	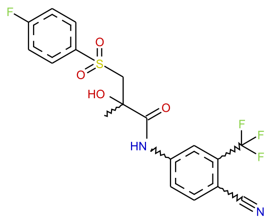
Actual Endpoint	Multiple-Carcinogen	Multiple-Carcinogen	Single-Carcinogen
Predicted Endpoint	Multiple-Carcinogen	Multiple-Carcinogen	Single-Carcinogen
Distance	0.791	0.794	0.795
Reference	US FDA (Centre for Drug Eval.& Res./Off. Testing & Res.) Sept. 1997	US FDA (Centre for Drug Eval.& Res./Off. Testing & Res.) Sept. 1997	US FDA (Centre for Drug Eval.& Res./Off. Testing & Res.) Sept. 1997

Model Applicability

Unknown features are fingerprint features in the query molecule, but not found in the training set.

1. OPS PC2 out of range. Value: 4.5771. Training min, max, SD, explained variance: -5.2888, 4.2744, 2.566, 0.1229.
2. Unknown FCFP_2 feature: 5: [*][O-]
3. Unknown FCFP_2 feature: -828984032: [*][c](:[*]):[c]([N+](=[*])[*]):c:[*]
4. Unknown FCFP_2 feature: -1338588315: [*]:[c](:[*])[N+](=O)[O-]
5. Unknown FCFP_2 feature: 1872392852: [*][N+](=O)[*]
6. Unknown FCFP_2 feature: 260476081: [*][N+](=[*])[O-]

Feature Contribution

Top features for positive contribution				
Fingerprint	Bit/Smiles	Feature Structure	Score	Multiple-Carcinogen in training set

FCFP_12	277509858	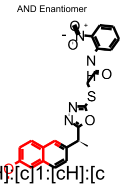 [*]:[cH]:[c]1:[cH]:[cH]:[c](OC):[cH]:[c]:1:[*]	0.400	1 out of 1
FCFP_12	-328193675	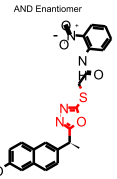 [*]S[c]1:o:[c]([*]):n:n:1	0.400	1 out of 1
FCFP_12	-1410079687	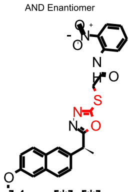 [*]S[c]1:o:[*]:[*]:n:1	0.395	2 out of 3

Top Features for negative contribution

Fingerprint	Bit/Smiles	Feature Structure	Score	Multiple-Carcinogen in training set
FCFP_12	1294255210	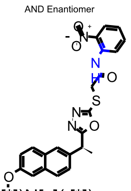 [*]C(=[*])N[c](:[*]):[*]	-1.626	0 out of 12
FCFP_12	1175665944	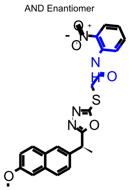 [*]CC(=O)N[c](:[cH]:[*]):[cH]:[*]	-1.219	0 out of 7

FCFP_12	590925877	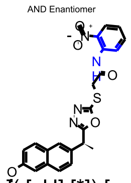 [*]N[c](:[cH]:[*]):[cH]:[*]	-0.998	1 out of 13

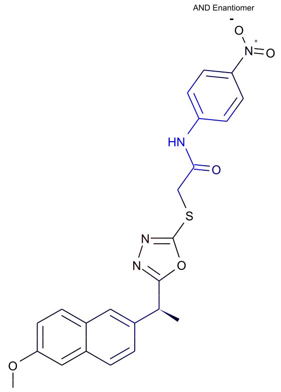

$C_{23}H_{20}N_4O_5S$

Molecular Weight: 464.4937

ALogP: 4.132

Rotatable Bonds: 8

Acceptors: 7

Donors: 1

Model Prediction

Prediction: Single-Carcinogen

Probability: 0.149

Enrichment: 0.495

Bayesian Score: -11.256

Mahalanobis Distance: 15.600

Mahalanobis Distance p-value: 8.47e-007

Prediction: Positive if the Bayesian score is above the estimated best cutoff value from minimizing the false positive and false negative rate.
Probability: The esimated probability that the sample is in the positive category. This assumes that the Bayesian score follows a normal distribution and is different from the prediction using a cutoff.
Enrichment: An estimate of enrichment, that is, the increased likelihood (versus random) of this sample being in the category.
Bayesian Score: The standard Laplacian-modified Bayesian score.
Mahalanobis Distance: The Mahalanobis distance (MD) is the distance to the center of the training data. The larger the MD, the less trustworthy the prediction.
Mahalanobis Distance p-value: The p-value gives the fraction of training data with an MD greater than or equal to the one for the given sample, assuming normally distributed data. The smaller the p-value, the less trustworthy the prediciton. For highly non-normal X properties (e.g., fingerprints), the MD p-value is wildly inaccurate.

Structural Similar Compounds

Name	Lovastatin	Bicalutamide	Simvastatin
Structure	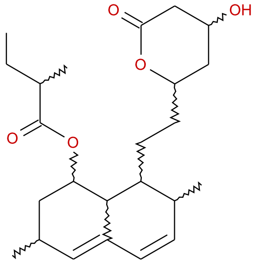	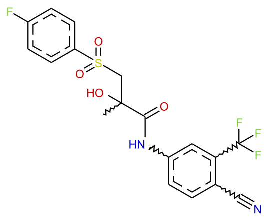	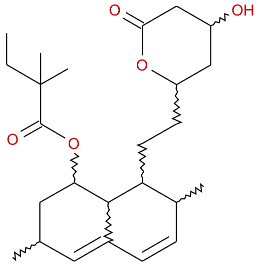
Actual Endpoint	Multiple-Carcinogen	Single-Carcinogen	Multiple-Carcinogen
Predicted Endpoint	Multiple-Carcinogen	Single-Carcinogen	Multiple-Carcinogen
Distance	0.791	0.793	0.794
Reference	US FDA (Centre for Drug Eval.& Res./Off. Testing & Res.) Sept. 1997	US FDA (Centre for Drug Eval.& Res./Off. Testing & Res.) Sept. 1997	US FDA (Centre for Drug Eval.& Res./Off. Testing & Res.) Sept. 1997

Model Applicability

Unknown features are fingerprint features in the query molecule, but not found in the training set.

1. All properties and OPS components are within expected ranges.
2. Unknown FCFP_2 feature: 5: [*][O-]
3. Unknown FCFP_2 feature: -828984032: [*][c](:[*]):[c]([N+](=[*])[*]):c:[*]
4. Unknown FCFP_2 feature: -1338588315: [*]:[c](:[*])[N+](=O)[O-]
5. Unknown FCFP_2 feature: 1872392852: [*][N+](=O)[*]
6. Unknown FCFP_2 feature: 260476081: [*][N+](=[*])[O-]

Feature Contribution

Top features for positive contribution

Fingerprint	Bit/Smiles	Feature Structure	Score	Multiple-Carcinogen in training set

FCFP_12	-328193675	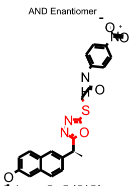 [*]S[c]1:o:[c]([*]):n :n:1	0.400	1 out of 1
FCFP_12	277509858	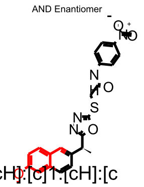 [*]:[cH]:[c]1:[cH]:[c H]:[c](OC):[cH]:[c]: 1:[*]	0.400	1 out of 1
FCFP_12	-1410079687	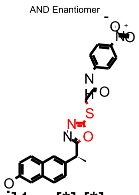 [*]S[c]1:o:[*]:[*]:n: 1	0.395	2 out of 3
**Top Features for negative contribution**				
**Fingerprint**	**Bit/Smiles**	**Feature Structure**	**Score**	**Multiple-Carcinogen in training set**
FCFP_12	1294255210	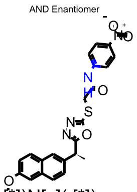 [*]C(=[*])N[c](:[*]): [*]	-1.626	0 out of 12
FCFP_12	1175665944	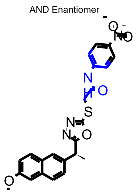 [*]CC(=O)N[c](:[cH]:[*]):[cH]:[*]	-1.219	0 out of 7

FCFP_12	590925877	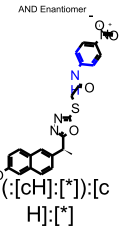 [*]N[c](:[cH]:[*]):[cH]:[*]	-0.998	1 out of 13

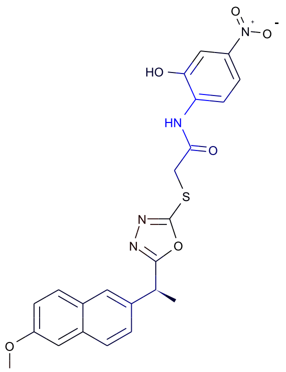

$C_{23}H_{20}N_4O_6S$
Molecular Weight: 480.4931
ALogP: 3.89
Rotatable Bonds: 8
Acceptors: 8
Donors: 2

Model Prediction

Prediction: Single-Carcinogen
Probability: 0.151
Enrichment: 0.503
Bayesian Score: -11.807
Mahalanobis Distance: 18.777
Mahalanobis Distance p-value: 3.23e-009

Prediction: Positive if the Bayesian score is above the estimated best cutoff value from minimizing the false positive and false negative rate.
Probability: The esimated probability that the sample is in the positive category. This assumes that the Bayesian score follows a normal distribution and is different from the prediction using a cutoff.
Enrichment: An estimate of enrichment, that is, the increased likelihood (versus random) of this sample being in the category.
Bayesian Score: The standard Laplacian-modified Bayesian score.
Mahalanobis Distance: The Mahalanobis distance (MD) is the distance to the center of the training data. The larger the MD, the less trustworthy the prediction.
Mahalanobis Distance p-value: The p-value gives the fraction of training data with an MD greater than or equal to the one for the given sample, assuming normally distributed data. The smaller the p-value, the less trustworthy the prediciton. For highly non-normal X properties (e.g., fingerprints), the MD p-value is wildly inaccurate.

Structural Similar Compounds

Name	Sulfasalazine	Bicalutamide	Glimepride
Structure	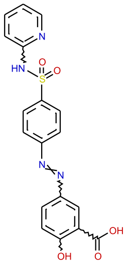	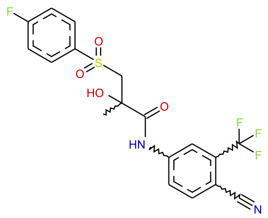	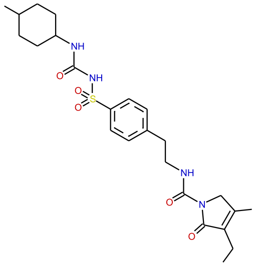
Actual Endpoint	Single-Carcinogen	Single-Carcinogen	Single-Carcinogen
Predicted Endpoint	Single-Carcinogen	Single-Carcinogen	Single-Carcinogen
Distance	0.740	0.830	0.860
Reference	US FDA (Centre for Drug Eval.& Res./Off. Testing & Res.) Sept. 1997	US FDA (Centre for Drug Eval.& Res./Off. Testing & Res.) Sept. 1997	US FDA (Centre for Drug Eval.& Res./Off. Testing & Res.) Sept. 1997

Model Applicability

Unknown features are fingerprint features in the query molecule, but not found in the training set.

1. OPS PC2 out of range. Value: 4.3332. Training min, max, SD, explained variance: -5.2888, 4.2744, 2.566, 0.1229.
2. Unknown FCFP_2 feature: 5: [*][O-]
3. Unknown FCFP_2 feature: -828984032: [*][c](:[*]):[c]([N+](=[*])[*]):c:[*]
4. Unknown FCFP_2 feature: -1338588315: [*]:[c](:[*])[N+](=O)[O-]
5. Unknown FCFP_2 feature: 1872392852: [*][N+](=O)[*]
6. Unknown FCFP_2 feature: 260476081: [*][N+](=[*])[O-]

Feature Contribution

Top features for positive contribution

Fingerprint	Bit/Smiles	Feature Structure	Score	Multiple-Carcinogen in training set

FCFP_12	277509858	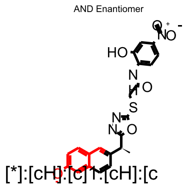 [*]:[cH]:[c]1:[cH]:[cH]:[c](OC):[cH]:[c]:1:[*]	0.400	1 out of 1
FCFP_12	-328193675	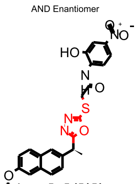 [*]S[c]1:o:[c]([*]):n:n:1	0.400	1 out of 1
FCFP_12	-1410079687	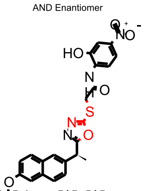 [*]S[c]1:o:[*]:[*]:n:1	0.395	2 out of 3
**Top Features for negative contribution**				
**Fingerprint**	**Bit/Smiles**	**Feature Structure**	**Score**	**Multiple-Carcinogen in training set**
FCFP_12	1294255210	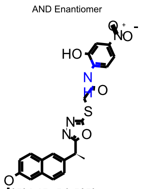 [*]C(=[*])N[c](:[*]):[*]	-1.626	0 out of 12
FCFP_12	1175665944	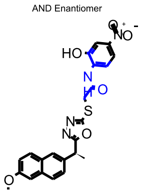 [*]CC(=O)N[c](:[cH]:[*]):[cH]:[*]	-1.219	0 out of 7

FCFP_12	590925877	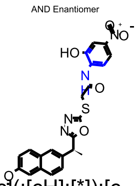 [*]N[c](:[cH]:[*]):[cH]:[*]	-0.998	1 out of 13

Sorafenib

TOPKAT_Mouse_Male_FDA_Single_vs_Multiple

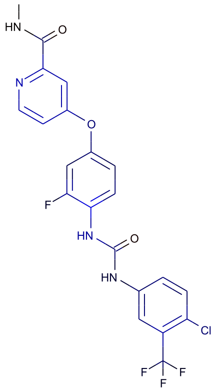

$C_{21}H_{15}ClF_4N_4O_3$

Molecular Weight: 482.81541

ALogP: 4.381

Rotatable Bonds: 6

Acceptors: 4

Donors: 3

Model Prediction

Prediction: Single-Carcinogen

Probability: 0.146

Enrichment: 0.485

Bayesian Score: -13.930

Mahalanobis Distance: 21.366

Mahalanobis Distance p-value: 4.72e-011

Prediction: Positive if the Bayesian score is above the estimated best cutoff value from minimizing the false positive and false negative rate.
Probability: The esimated probability that the sample is in the positive category. This assumes that the Bayesian score follows a normal distribution and is different from the prediction using a cutoff.
Enrichment: An estimate of enrichment, that is, the increased likelihood (versus random) of this sample being in the category.
Bayesian Score: The standard Laplacian-modified Bayesian score.
Mahalanobis Distance: The Mahalanobis distance (MD) is the distance to the center of the training data. The larger the MD, the less trustworthy the prediction.
Mahalanobis Distance p-value: The p-value gives the fraction of training data with an MD greater than or equal to the one for the given sample, assuming normally distributed data. The smaller the p-value, the less trustworthy the prediciton. For highly non-normal X properties (e.g., fingerprints), the MD p-value is wildly inaccurate.

Structural Similar Compounds

Name	Glimepride	Bicalutamide	Lansoprazole
Structure	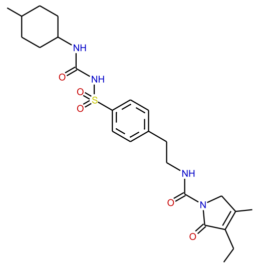	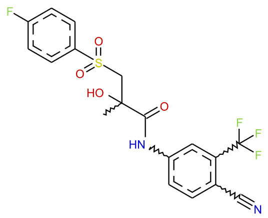	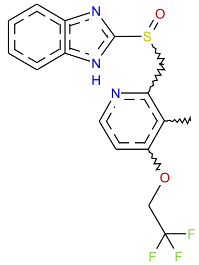
Actual Endpoint	Single-Carcinogen	Single-Carcinogen	Single-Carcinogen
Predicted Endpoint	Single-Carcinogen	Single-Carcinogen	Single-Carcinogen
Distance	0.627	0.721	0.885
Reference	US FDA (Centre for Drug Eval.& Res./Off. Testing & Res.) Sept. 1997	US FDA (Centre for Drug Eval.& Res./Off. Testing & Res.) Sept. 1997	US FDA (Centre for Drug Eval.& Res./Off. Testing & Res.) Sept. 1997

Model Applicability

Unknown features are fingerprint features in the query molecule, but not found in the training set.

1. All properties and OPS components are within expected ranges.

Feature Contribution

Top features for positive contribution

Fingerprint	Bit/Smiles	Feature Structure	Score	Multiple-Carcinogen in training set
FCFP_12	1499521844	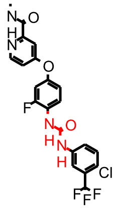 [*]NC(=O)N[*]	0.390	5 out of 9

FCFP_12	-904785030	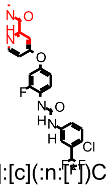 [*]:[cH]:[c](:n:[*])C(=O)NC	0.174	1 out of 2
FCFP_12	-1549103449	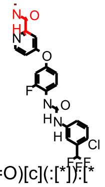 [*]NC(=O)[c](:[*]):[*]	0.168	3 out of 7
**Top Features for negative contribution**				
**Fingerprint**	**Bit/Smiles**	**Feature Structure**	**Score**	**Multiple-Carcinogen in training set**
FCFP_12	1294255210	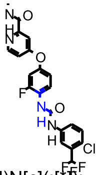 [*]C(=[*])N[c](:[*]):[*]	-1.626	0 out of 12
FCFP_12	590925877	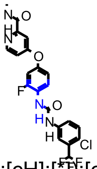 [*]N[c](:[cH]:[*]):[cH]:[*]	-0.998	1 out of 13
FCFP_12	-1462709112	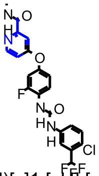 [*]C(=[*])[c]1:[cH]:[*]:[cH]:[cH]:n:1	-0.994	0 out of 5

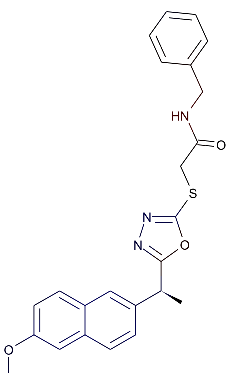

$C_{24}H_{23}N_3O_3S$
Molecular Weight: 433.52272
ALogP: 4.245
Rotatable Bonds: 8
Acceptors: 5
Donors: 1

Structural Similar Compounds

Name	1-BENZOYLAMINO-4-METHOXY-5-CHLORANTHRAQUINONE	ANTHRAQUINONE; 1;1'-IMINODI-	Cinchoninamide; 2-butoxy-N-(2-(diethylamino)ethyl)-; monohydrochloride
Structure	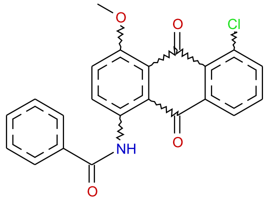	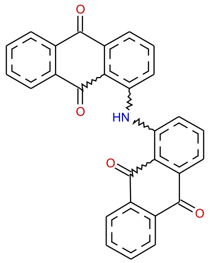	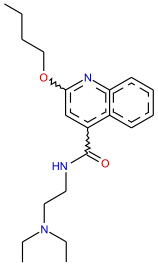
Actual Endpoint	Mild	Mild	Moderate_Severe
Predicted Endpoint	Mild	Mild	Moderate_Severe
Distance	0.642	0.672	0.686
Reference	28ZPAK-;90;72	28ZPAK-;125;72	Arzneimittel-Forschung 8;181;58

Model Prediction

Prediction: Mild
Probability: 0.718
Enrichment: 1.042
Bayesian Score: -3.192
Mahalanobis Distance: 10.369
Mahalanobis Distance p-value: 0.0368

Prediction: Positive if the Bayesian score is above the estimated best cutoff value from minimizing the false positive and false negative rate.
Probability: The esimated probability that the sample is in the positive category. This assumes that the Bayesian score follows a normal distribution and is different from the prediction using a cutoff.
Enrichment: An estimate of enrichment, that is, the increased likelihood (versus random) of this sample being in the category.
Bayesian Score: The standard Laplacian-modified Bayesian score.
Mahalanobis Distance: The Mahalanobis distance (MD) is the distance to the center of the training data. The larger the MD, the less trustworthy the prediction.
Mahalanobis Distance p-value: The p-value gives the fraction of training data with an MD greater than or equal to the one for the given sample, assuming normally distributed data. The smaller the p-value, the less trustworthy the prediciton. For highly non-normal X properties (e.g., fingerprints), the MD p-value is wildly inaccurate.

Model Applicability

Unknown features are fingerprint features in the query molecule, but not found in the training set.

1. All properties and OPS components are within expected ranges.
2. Unknown FCFP_2 feature: -928857652: [*]:[c](:[*])C(C)[c](:[*]):[*]

Feature Contribution

Top features for positive contribution				
**Fingerprint**	**Bit/Smiles**	**Feature Structure**	**Score**	**Moderate_Severe in training set**
FCFP_10	907096426	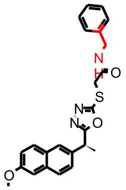 AND Enantiomer [*]NC[c](:[*]):[*]	0.332	5 out of 5

FCFP_10	427906732	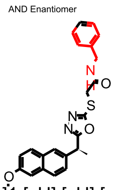 [*]NC[c]1:[cH]:[cH]:[*]:[cH]:[cH]:1	0.294	3 out of 3
FCFP_10	-1539162406	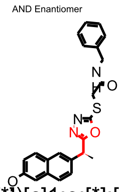 [*]C([*])[c]1:o:[*]:[*]:n:1	0.294	3 out of 3
**Top Features for negative contribution**				
**Fingerprint**	**Bit/Smiles**	**Feature Structure**	**Score**	**Moderate_Severe in training set**
FCFP_10	4427049	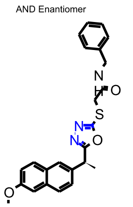 [*][c]1:[*]:[*]:n:n:1	-1.293	0 out of 4
FCFP_10	-1977641857	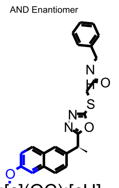 [*]:[cH]:[c](OC):[cH]:[*]	-0.780	4 out of 15
FCFP_10	-1371139928	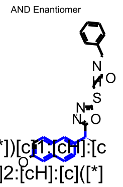 [*]C([*])[c]1:[cH]:[cH]:[c]2:[cH]:[c]([*]):[*]:[cH]:[c]:2:[cH]:1	-0.507	0 out of 1

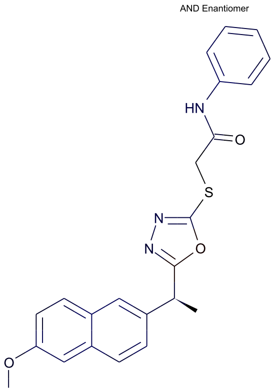

$C_{23}H_{21}N_3O_3S$
Molecular Weight: 419.49614
ALogP: 4.238
Rotatable Bonds: 7
Acceptors: 5
Donors: 1

Model Prediction

Prediction: Mild
Probability: 0.563
Enrichment: 0.817
Bayesian Score: -5.461
Mahalanobis Distance: 8.584
Mahalanobis Distance p-value: 0.727

Prediction: Positive if the Bayesian score is above the estimated best cutoff value from minimizing the false positive and false negative rate.
Probability: The esimated probability that the sample is in the positive category. This assumes that the Bayesian score follows a normal distribution and is different from the prediction using a cutoff.
Enrichment: An estimate of enrichment, that is, the increased likelihood (versus random) of this sample being in the category.
Bayesian Score: The standard Laplacian-modified Bayesian score.
Mahalanobis Distance: The Mahalanobis distance (MD) is the distance to the center of the training data. The larger the MD, the less trustworthy the prediction.
Mahalanobis Distance p-value: The p-value gives the fraction of training data with an MD greater than or equal to the one for the given sample, assuming normally distributed data. The smaller the p-value, the less trustworthy the prediciton. For highly non-normal X properties (e.g., fingerprints), the MD p-value is wildly inaccurate.

Structural Similar Compounds

Name	1-BENZOYLAMINO-4-METHOXY-5-CHLORANTHRAQUINONE	ANTHRAQUINONE; 1;1'-IMINODI-	N;S-DIBENZOYL-O-AMINOTHIOPHENOL
Structure	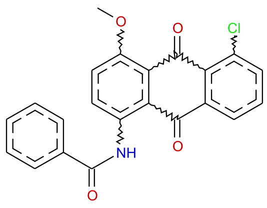	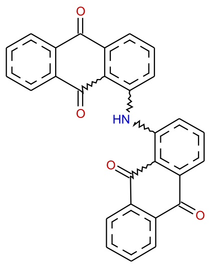	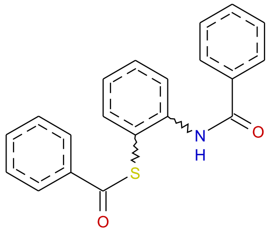
Actual Endpoint	Mild	Mild	Mild
Predicted Endpoint	Mild	Mild	Mild
Distance	0.610	0.648	0.688
Reference	28ZPAK-;90;72	28ZPAK-;125;72	28ZPAK-;175;72

Model Applicability

Unknown features are fingerprint features in the query molecule, but not found in the training set.

1. All properties and OPS components are within expected ranges.
2. Unknown FCFP_2 feature: -928857652: [*]:[c](:[*])C(C)[c](:[*]):[*]

Feature Contribution

Top features for positive contribution				
**Fingerprint**	**Bit/Smiles**	**Feature Structure**	**Score**	**Moderate_Severe in training set**
FCFP_10	-1539162406	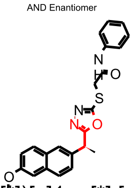 [*]C([*])[c]1:o:[*]:[*]:n:1	0.294	3 out of 3

FCFP_10	346218766	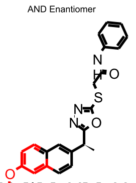 [*]:[c]1:[*]:[cH]:[cH]:[c](OC):[cH]:1	0.197	30 out of 37
FCFP_10	-1370111440	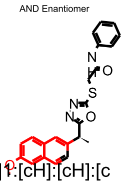 [*]O[c]1:[cH]:[cH]:[c]2:[cH]:[c]([*]):[*]:[cH]:[c]:2:[cH]:1	0.186	1 out of 1
**Top Features for negative contribution**				
**Fingerprint**	**Bit/Smiles**	**Feature Structure**	**Score**	**Moderate_Severe in training set**
FCFP_10	4427049	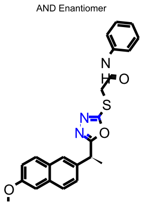 [*][c]1:[*]:[*]:n:n:1	-1.293	0 out of 4
FCFP_10	-1977641857	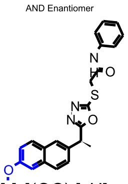 [*]:[cH]:[c](OC):[cH]:[*]	-0.780	4 out of 15
FCFP_10	-1371139928	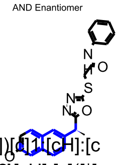 [*]C([*])[c]1:[cH]:[cH]:[c]2:[cH]:[c]([*]):[*]:[cH]:[c]:2:[cH]:1	-0.507	0 out of 1

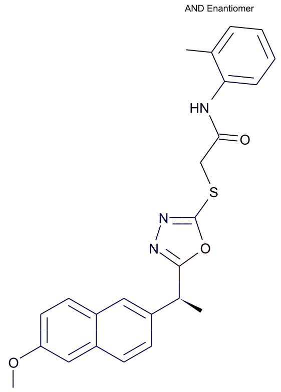

$C_{24}H_{23}N_3O_3S$

Molecular Weight: 433.52272

ALogP: 4.724

Rotatable Bonds: 7

Acceptors: 5

Donors: 1

Structural Similar Compounds

Name	ANTHRAQUINONE; 1;1'-IMINODI-	1-BENZOYLAMINO-4-METHOXY-5-CHLORANTHRAQUINONE	N;S-DIBENZOYL-O-AMINOTHIOPHENOL
Structure	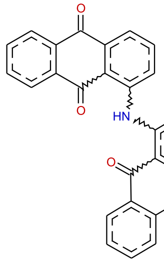	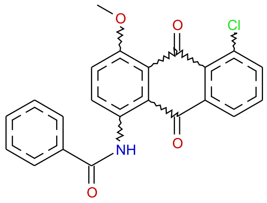	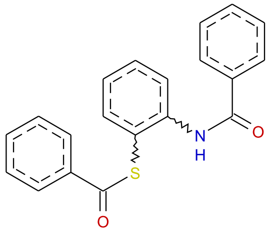
Actual Endpoint	Mild	Mild	Mild
Predicted Endpoint	Mild	Mild	Mild
Distance	0.615	0.618	0.701
Reference	28ZPAK-;125;72	28ZPAK-;90;72	28ZPAK-;175;72

Model Prediction

Prediction: Mild

Probability: 0.637

Enrichment: 0.925

Bayesian Score: -4.515

Mahalanobis Distance: 8.667

Mahalanobis Distance p-value: 0.687

Prediction: Positive if the Bayesian score is above the estimated best cutoff value from minimizing the false positive and false negative rate.
Probability: The esimated probability that the sample is in the positive category. This assumes that the Bayesian score follows a normal distribution and is different from the prediction using a cutoff.
Enrichment: An estimate of enrichment, that is, the increased likelihood (versus random) of this sample being in the category.
Bayesian Score: The standard Laplacian-modified Bayesian score.
Mahalanobis Distance: The Mahalanobis distance (MD) is the distance to the center of the training data. The larger the MD, the less trustworthy the prediction.
Mahalanobis Distance p-value: The p-value gives the fraction of training data with an MD greater than or equal to the one for the given sample, assuming normally distributed data. The smaller the p-value, the less trustworthy the prediciton. For highly non-normal X properties (e.g., fingerprints), the MD p-value is wildly inaccurate.

Model Applicability

Unknown features are fingerprint features in the query molecule, but not found in the training set.

1. All properties and OPS components are within expected ranges.
2. Unknown FCFP_2 feature: -928857652: [*]:[c](:[*])C(C)[c](:[*]):[*]

Feature Contribution

Top features for positive contribution

Fingerprint	Bit/Smiles	Feature Structure	Score	Moderate_Severe in training set
FCFP_10	1396506317	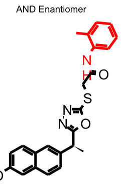 [*]N[c]1:[cH]:[cH]:[cH]:[cH]:[c]:1C	0.317	4 out of 4

FCFP_10	-1539162406	AND Enantiomer 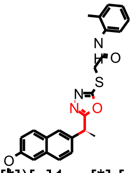 [*]C([*])[c]1:o:[*]:[*]:n:1	0.294	3 out of 3
FCFP_10	755520106	AND Enantiomer 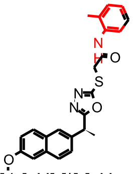 [*]N[c]1:[cH]:[*]:[cH]:[cH]:[c]:1C	0.273	9 out of 10
**Top Features for negative contribution**				
**Fingerprint**	**Bit/Smiles**	**Feature Structure**	**Score**	**Moderate_Severe in training set**
FCFP_10	4427049	AND Enantiomer 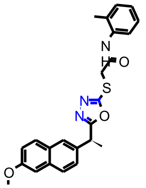 [*][c]1:[*]:[*]:n:n:1	-1.293	0 out of 4
FCFP_10	-1977641857	AND Enantiomer 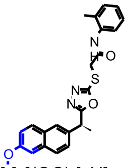 [*]:[cH]:[c](OC):[cH]:[*]	-0.780	4 out of 15
FCFP_10	-1371139928	AND Enantiomer 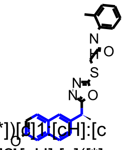 [*]C([*])[c]1:[cH]:[cH]:[c]2:[cH]:[c]([*]):[*]:[cH]:[c]:2:[cH]:1	-0.507	0 out of 1

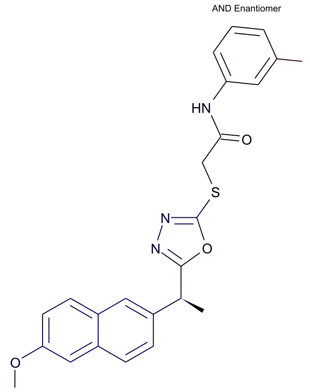

$C_{24}H_{23}N_3O_3S$

Molecular Weight: 433.52272

ALogP: 4.724

Rotatable Bonds: 7

Acceptors: 5

Donors: 1

Model Prediction

Prediction: Mild

Probability: 0.672

Enrichment: 0.975

Bayesian Score: -3.994

Mahalanobis Distance: 8.667

Mahalanobis Distance p-value: 0.687

Prediction: Positive if the Bayesian score is above the estimated best cutoff value from minimizing the false positive and false negative rate.
Probability: The esimated probability that the sample is in the positive category. This assumes that the Bayesian score follows a normal distribution and is different from the prediction using a cutoff.
Enrichment: An estimate of enrichment, that is, the increased likelihood (versus random) of this sample being in the category.
Bayesian Score: The standard Laplacian-modified Bayesian score.
Mahalanobis Distance: The Mahalanobis distance (MD) is the distance to the center of the training data. The larger the MD, the less trustworthy the prediction.
Mahalanobis Distance p-value: The p-value gives the fraction of training data with an MD greater than or equal to the one for the given sample, assuming normally distributed data. The smaller the p-value, the less trustworthy the prediciton. For highly non-normal X properties (e.g., fingerprints), the MD p-value is wildly inaccurate.

Structural Similar Compounds

Name	ANTHRAQUINONE; 1;1'-IMINODI-	1-BENZOYLAMINO-4-METHOXY-5-CHLORANTHRAQUINONE	N;S-DIBENZOYL-O-AMINOTHIOPHENOL
Structure	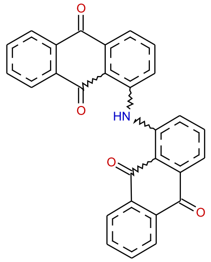	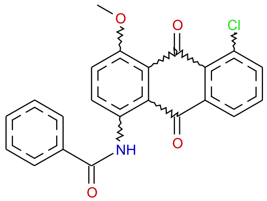	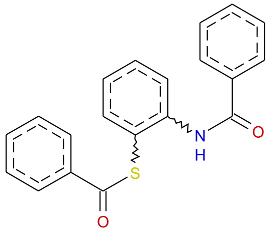
Actual Endpoint	Mild	Mild	Mild
Predicted Endpoint	Mild	Mild	Mild
Distance	0.620	0.625	0.709
Reference	28ZPAK-;125;72	28ZPAK-;90;72	28ZPAK-;175;72

Model Applicability

Unknown features are fingerprint features in the query molecule, but not found in the training set.

1. All properties and OPS components are within expected ranges.
2. Unknown FCFP_2 feature: -928857652: [*]:[c](:[*])C(C)[c](:[*]):[*]

Feature Contribution

Top features for positive contribution

Fingerprint	Bit/Smiles	Feature Structure	Score	Moderate_Severe in training set
FCFP_10	-1539162406	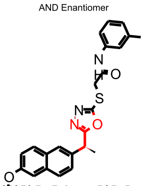 [*]C([*])[c]1:o:[*]:[*]:n:1	0.294	3 out of 3

FCFP_10	393262357	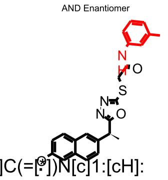 [*]C(=[*])N[c]1:[cH]:[cH]:[cH]:[c](C):[cH]:1	0.294	3 out of 3
FCFP_10	136120670	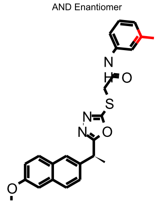 [*]:[c](:[*])C	0.206	53 out of 65

Top Features for negative contribution

**Fingerprint**	**Bit/Smiles**	**Feature Structure**	**Score**	**Moderate_Severe in training set**
FCFP_10	4427049	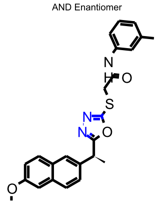 [*][c]1:[*]:[*]:n:n:1	-1.293	0 out of 4
FCFP_10	-1977641857	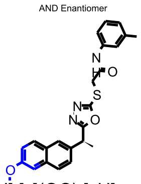 [*]:[cH]:[c](OC):[cH]:[*]	-0.780	4 out of 15
FCFP_10	-1371139928	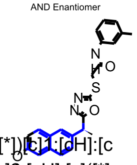 [*]C([*])[c]1:[cH]:[cH]:[c]2:[cH]:[c]([*]):[*]:[cH]:[c]:2:[cH]:1	-0.507	0 out of 1

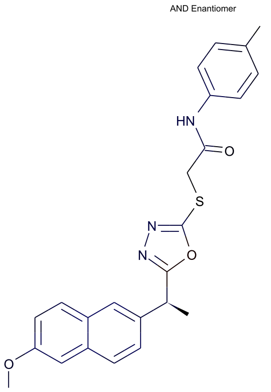

$C_{24}H_{23}N_3O_3S$

Molecular Weight: 433.52272

ALogP: 4.724

Rotatable Bonds: 7

Acceptors: 5

Donors: 1

Structural Similar Compounds

Name	ANTHRAQUINONE; 1;1'-IMINODI-	1-BENZOYLAMINO-4-METHOXY-5-CHLORANTHRAQUINONE	Cinchoninamide; 2-butoxy-N-(2-(diethylamino)ethyl)-; monohydrochloride
Structure	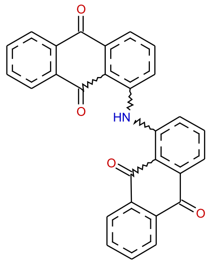	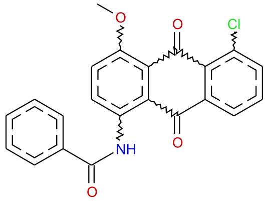	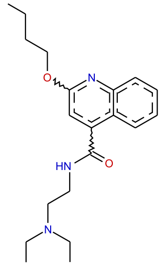
Actual Endpoint	Mild	Mild	Moderate_Severe
Predicted Endpoint	Mild	Mild	Moderate_Severe
Distance	0.623	0.628	0.712
Reference	28ZPAK-;125;72	28ZPAK-;90;72	Arzneimittel-Forschung 8;181;58

Model Prediction

Prediction: Mild

Probability: 0.619

Enrichment: 0.898

Bayesian Score: -4.770

Mahalanobis Distance: 8.667

Mahalanobis Distance p-value: 0.687

Prediction: Positive if the Bayesian score is above the estimated best cutoff value from minimizing the false positive and false negative rate.
Probability: The esimated probability that the sample is in the positive category. This assumes that the Bayesian score follows a normal distribution and is different from the prediction using a cutoff.
Enrichment: An estimate of enrichment, that is, the increased likelihood (versus random) of this sample being in the category.
Bayesian Score: The standard Laplacian-modified Bayesian score.
Mahalanobis Distance: The Mahalanobis distance (MD) is the distance to the center of the training data. The larger the MD, the less trustworthy the prediction.
Mahalanobis Distance p-value: The p-value gives the fraction of training data with an MD greater than or equal to the one for the given sample, assuming normally distributed data. The smaller the p-value, the less trustworthy the prediciton. For highly non-normal X properties (e.g., fingerprints), the MD p-value is wildly inaccurate.

Model Applicability

Unknown features are fingerprint features in the query molecule, but not found in the training set.

1. All properties and OPS components are within expected ranges.
2. Unknown FCFP_2 feature: -928857652: [*]:[c](:[*])C(C)[c](:[*]):[*]

Feature Contribution

Top features for positive contribution				
**Fingerprint**	**Bit/Smiles**	**Feature Structure**	**Score**	**Moderate_Severe in training set**
FCFP_10	-1539162406	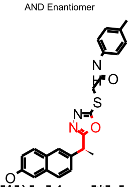 [*]C([*])[c]1:o:[*]:[*]:n:1	0.294	3 out of 3

FCFP_10	136120670	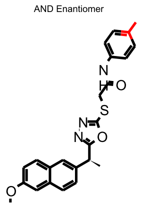 [*]:[c](:[*])C	0.206	53 out of 65
FCFP_10	346218766	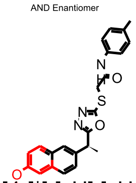 [*]:[c]1:[*]:[cH]:[cH]:[c](OC):[cH]:1	0.197	30 out of 37
**Top Features for negative contribution**				
**Fingerprint**	**Bit/Smiles**	**Feature Structure**	**Score**	**Moderate_Severe in training set**
FCFP_10	4427049	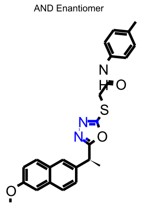 [*][c]1:[*]:[*]:n:n:1	-1.293	0 out of 4
FCFP_10	-1977641857	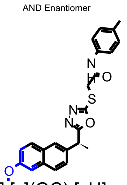 [*]:[cH]:[c](OC):[cH] :[*]	-0.780	4 out of 15
FCFP_10	-790336137	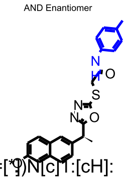 [*]C(=[*])N[c]1:[cH]:[cH]:[c](C):[cH]:[cH]:1	-0.507	0 out of 1

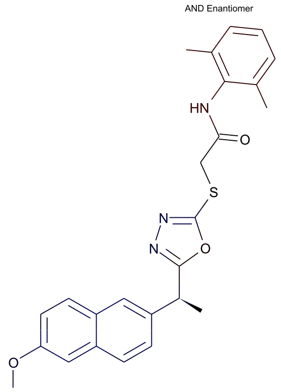

$C_{25}H_{25}N_3O_3S$
Molecular Weight: 447.5493
ALogP: 5.21
Rotatable Bonds: 7
Acceptors: 5
Donors: 1

Model Prediction

Prediction: Mild

Probability: 0.730

Enrichment: 1.059

Bayesian Score: -2.947

Mahalanobis Distance: 8.771

Mahalanobis Distance p-value: 0.635

Prediction: Positive if the Bayesian score is above the estimated best cutoff value from minimizing the false positive and false negative rate.
Probability: The esimated probability that the sample is in the positive category. This assumes that the Bayesian score follows a normal distribution and is different from the prediction using a cutoff.
Enrichment: An estimate of enrichment, that is, the increased likelihood (versus random) of this sample being in the category.
Bayesian Score: The standard Laplacian-modified Bayesian score.
Mahalanobis Distance: The Mahalanobis distance (MD) is the distance to the center of the training data. The larger the MD, the less trustworthy the prediction.
Mahalanobis Distance p-value: The p-value gives the fraction of training data with an MD greater than or equal to the one for the given sample, assuming normally distributed data. The smaller the p-value, the less trustworthy the prediciton. For highly non-normal X properties (e.g., fingerprints), the MD p-value is wildly inaccurate.

Structural Similar Compounds

Name	ANTHRAQUINONE; 1;1'-IMINODI-	1-BENZOYLAMINO-4-METHOXY-5-CHLORANTHRAQUINONE	2-(1'-ANTHRAQUINONYL)-AMINOBENZANTHRONE
Structure	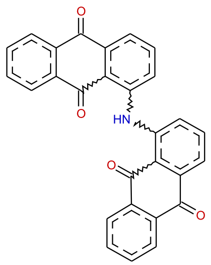	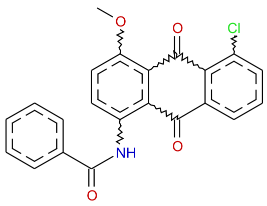	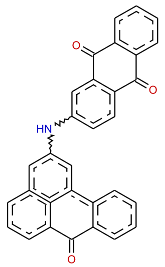
Actual Endpoint	Mild	Mild	Mild
Predicted Endpoint	Mild	Mild	Mild
Distance	0.612	0.655	0.690
Reference	28ZPAK-;125;72	28ZPAK-;90;72	28ZPAK-;126;72

Model Applicability

Unknown features are fingerprint features in the query molecule, but not found in the training set.

1. All properties and OPS components are within expected ranges.
2. Unknown FCFP_2 feature: -928857652: [*]:[c](:[*])C(C)[c](:[*]):[*]

Feature Contribution

Top features for positive contribution

Fingerprint	Bit/Smiles	Feature Structure	Score	Moderate_Severe in training set
FCFP_10	1396506317	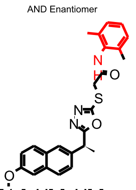 [*]N[c]1:[cH]:[cH]:[cH]:[cH]:[c]:1C	0.317	4 out of 4

FCFP_10	-1539162406	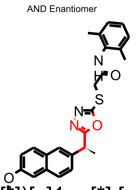 [*]C([*])[c]1:o:[*]:[*]:n:1	0.294	3 out of 3
FCFP_10	755520106	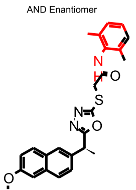 [*]N[c]1:[cH]:[*]:[cH]:[cH]:[c]:1C	0.273	9 out of 10

Top Features for negative contribution

**Fingerprint**	**Bit/Smiles**	**Feature Structure**	**Score**	**Moderate_Severe in training set**
FCFP_10	4427049	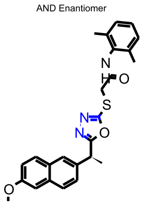 [*][c]1:[*]:[*]:n:n:1	-1.293	0 out of 4
FCFP_10	-1977641857	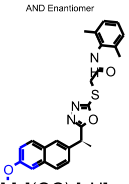 [*]:[cH]:[c](OC):[cH]:[*]	-0.780	4 out of 15
FCFP_10	-1371139928	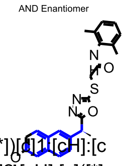 [*]C([*])[c]1:[cH]:[cH]:[c]2:[cH]:[c]([*]):[*]:[cH]:[c]:2:[cH]:1	-0.507	0 out of 1

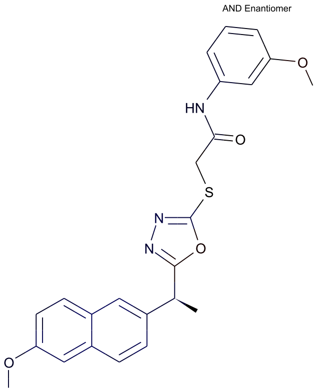

$C_{24}H_{23}N_3O_4S$
Molecular Weight: 449.52212
ALogP: 4.221
Rotatable Bonds: 8
Acceptors: 6
Donors: 1

Model Prediction

Prediction: Mild
Probability: 0.633
Enrichment: 0.919
Bayesian Score: -4.572
Mahalanobis Distance: 8.622
Mahalanobis Distance p-value: 0.709

Prediction: Positive if the Bayesian score is above the estimated best cutoff value from minimizing the false positive and false negative rate.
Probability: The esimated probability that the sample is in the positive category. This assumes that the Bayesian score follows a normal distribution and is different from the prediction using a cutoff.
Enrichment: An estimate of enrichment, that is, the increased likelihood (versus random) of this sample being in the category.
Bayesian Score: The standard Laplacian-modified Bayesian score.
Mahalanobis Distance: The Mahalanobis distance (MD) is the distance to the center of the training data. The larger the MD, the less trustworthy the prediction.
Mahalanobis Distance p-value: The p-value gives the fraction of training data with an MD greater than or equal to the one for the given sample, assuming normally distributed data. The smaller the p-value, the less trustworthy the prediciton. For highly non-normal X properties (e.g., fingerprints), the MD p-value is wildly inaccurate.

Structural Similar Compounds

Name	ANTHRAQUINONE; 1;1'-IMINODI-	1-BENZOYLAMINO-4-METHOXY-5-CHLORANTHRAQUINONE	COLCHICINE
Structure	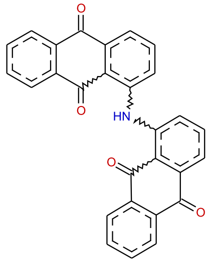	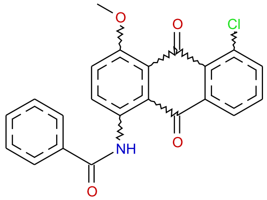	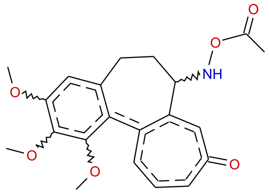
Actual Endpoint	Mild	Mild	Moderate_Severe
Predicted Endpoint	Mild	Mild	Moderate_Severe
Distance	0.694	0.711	0.720
Reference	28ZPAK-;125;72	28ZPAK-;90;72	AJOPAA 31;837;48

Model Applicability

Unknown features are fingerprint features in the query molecule, but not found in the training set.

1. All properties and OPS components are within expected ranges.
2. Unknown FCFP_2 feature: -928857652: [*]:[c](:[*])C(C)[c](:[*]):[*]

Feature Contribution

Top features for positive contribution

Fingerprint	Bit/Smiles	Feature Structure	Score	Moderate_Severe in training set
FCFP_10	-1539162406	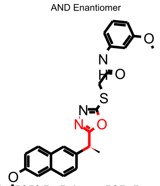 [*]C([*])[c]1:o:[*]:[*]:n:1	0.294	3 out of 3

FCFP_10	346218766	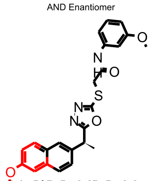[*]:[c]1:[*]:[cH]:[cH]:[c](OC):[cH]:1	0.197	30 out of 37
FCFP_10	-768690632	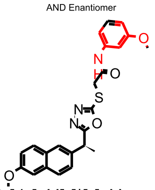[*]N[c]1:[cH]:[*]:[cH]:[c](O[*]):[cH]:1	0.186	1 out of 1

Top Features for negative contribution

Fingerprint	Bit/Smiles	Feature Structure	Score	Moderate_Severe in training set
FCFP_10	4427049	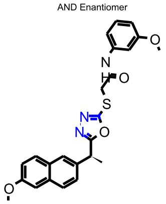[*][c]1:[*]:[*]:n:n:1	-1.293	0 out of 4
FCFP_10	-1977641857	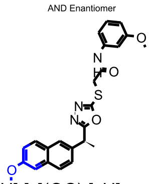[*]:[cH]:[c](OC):[cH]:[*]	-0.780	4 out of 15
FCFP_10	-1371139928	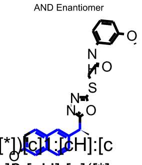[*]C([*])[c]1:[cH]:[cH]:[c]2:[cH]:[c]([*]):[*]:[cH]:[c]:2:[cH]:1	-0.507	0 out of 1

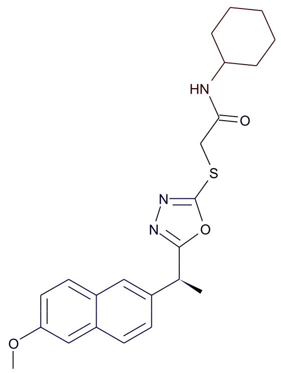

$C_{23}H_{27}N_3O_3S$
Molecular Weight: 425.54378
ALogP: 4.52
Rotatable Bonds: 7
Acceptors: 5
Donors: 1

Structural Similar Compounds

Name	1-BENZOYLAMINO-4-METHOXY-5-CHLORANTHRAQUINONE	ANTHRAQUINONE; 1;1'-IMINODI-	Cinchoninamide; 2-butoxy-N-(2-(diethylamino)ethyl)-; monohydrochloride
Structure	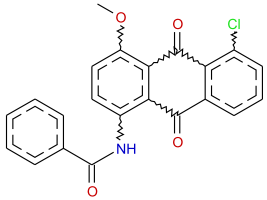	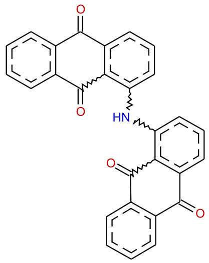	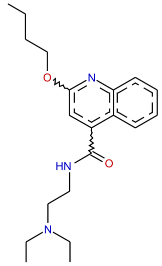
Actual Endpoint	Mild	Mild	Moderate_Severe
Predicted Endpoint	Mild	Mild	Moderate_Severe
Distance	0.630	0.641	0.694
Reference	28ZPAK-;90;72	28ZPAK-;125;72	Arzneimittel-Forschung 8;181;58

Model Prediction

Prediction: Mild
Probability: 0.754
Enrichment: 1.094
Bayesian Score: -2.399
Mahalanobis Distance: 12.354
Mahalanobis Distance p-value: 1.04e-005

Prediction: Positive if the Bayesian score is above the estimated best cutoff value from minimizing the false positive and false negative rate.
Probability: The esimated probability that the sample is in the positive category. This assumes that the Bayesian score follows a normal distribution and is different from the prediction using a cutoff.
Enrichment: An estimate of enrichment, that is, the increased likelihood (versus random) of this sample being in the category.
Bayesian Score: The standard Laplacian-modified Bayesian score.
Mahalanobis Distance: The Mahalanobis distance (MD) is the distance to the center of the training data. The larger the MD, the less trustworthy the prediction.
Mahalanobis Distance p-value: The p-value gives the fraction of training data with an MD greater than or equal to the one for the given sample, assuming normally distributed data. The smaller the p-value, the less trustworthy the prediciton. For highly non-normal X properties (e.g., fingerprints), the MD p-value is wildly inaccurate.

Model Applicability

Unknown features are fingerprint features in the query molecule, but not found in the training set.

1. All properties and OPS components are within expected ranges.
2. Unknown FCFP_2 feature: -928857652: [*]:[c](:[*])C(C)[c](:[*]):[*]

Feature Contribution

Top features for positive contribution				
**Fingerprint**	**Bit/Smiles**	**Feature Structure**	**Score**	**Moderate_Severe in training set**
FCFP_10	-796673622	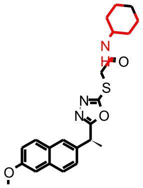 AND Enantiomer [*]C(=[*])NC1CC[*]CC1	0.317	4 out of 4

FCFP_10	-1539162406	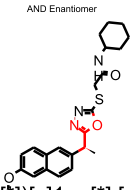 [*]C([*])[c]1:o:[*]:[*]:n:1	0.294	3 out of 3
FCFP_10	654530535	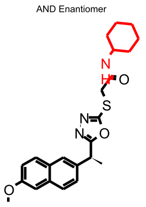 [*]C(=[*])NC1CCCCC1	0.294	3 out of 3

Top Features for negative contribution

Fingerprint	Bit/Smiles	Feature Structure	Score	Moderate_Severe in training set
FCFP_10	4427049	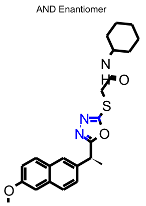 [*][c]1:[*]:[*]:n:n:1	-1.293	0 out of 4
FCFP_10	-1977641857	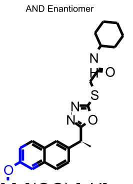 [*]:[cH]:[c](OC):[cH] :[*]	-0.780	4 out of 15
FCFP_10	-1371139928	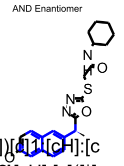 [*]C([*])[c]1:[cH]:[c H]:[c]2:[cH]:[c]([*]):[*]:[cH]:[c]:2:[cH]:1	-0.507	0 out of 1

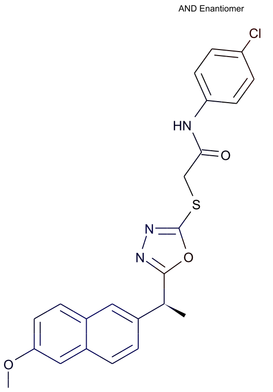

$C_{23}H_{20}ClN_3O_3S$

Molecular Weight: 453.9412

ALogP: 4.902

Rotatable Bonds: 7

Acceptors: 5

Donors: 1

Model Prediction

Prediction: Mild

Probability: 0.684

Enrichment: 0.992

Bayesian Score: -3.803

Mahalanobis Distance: 9.228

Mahalanobis Distance p-value: 0.389

Prediction: Positive if the Bayesian score is above the estimated best cutoff value from minimizing the false positive and false negative rate.
Probability: The esimated probability that the sample is in the positive category. This assumes that the Bayesian score follows a normal distribution and is different from the prediction using a cutoff.
Enrichment: An estimate of enrichment, that is, the increased likelihood (versus random) of this sample being in the category.
Bayesian Score: The standard Laplacian-modified Bayesian score.
Mahalanobis Distance: The Mahalanobis distance (MD) is the distance to the center of the training data. The larger the MD, the less trustworthy the prediction.
Mahalanobis Distance p-value: The p-value gives the fraction of training data with an MD greater than or equal to the one for the given sample, assuming normally distributed data. The smaller the p-value, the less trustworthy the prediciton. For highly non-normal X properties (e.g., fingerprints), the MD p-value is wildly inaccurate.

Structural Similar Compounds

Name	ANTHRAQUINONE; 1;1'-IMINODI-	1-BENZOYLAMINO-4-METHOXY-5-CHLORANTHRAQUINONE	2-(1'-ANTHRAQUINONYL)-AMINOBENZANTHRONE
Structure	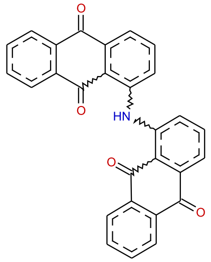	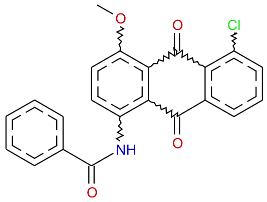	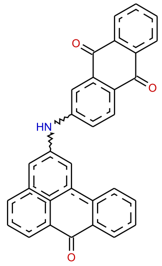
Actual Endpoint	Mild	Mild	Mild
Predicted Endpoint	Mild	Mild	Mild
Distance	0.624	0.638	0.715
Reference	28ZPAK-;125;72	28ZPAK-;90;72	28ZPAK-;126;72

Model Applicability

Unknown features are fingerprint features in the query molecule, but not found in the training set.

1. All properties and OPS components are within expected ranges.
2. Unknown FCFP_2 feature: -928857652: [*]:[c](:[*])C(C)[c](:[*]):[*]

Feature Contribution

Top features for positive contribution

Fingerprint	Bit/Smiles	Feature Structure	Score	Moderate_Severe in training set
FCFP_10	-1508180856	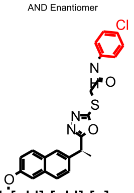 [*][c]1:[cH]:[cH]:[c](Cl):[cH]:[cH]:1	0.329	16 out of 17

FCFP_10	-745491832	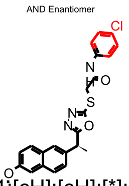 Cl[c]1:[cH]:[cH]:[*]:[cH]:[cH]:1	0.304	29 out of 32
FCFP_10	-1539162406	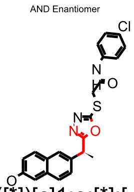 [*]C([*])[c]1:o:[*]:[*]:n:1	0.294	3 out of 3

Top Features for negative contribution

Fingerprint	Bit/Smiles	Feature Structure	Score	Moderate_Severe in training set
FCFP_10	4427049	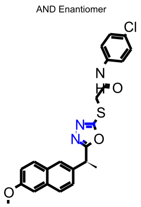 [*][c]1:[*]:[*]:n:n:1	-1.293	0 out of 4
FCFP_10	-1977641857	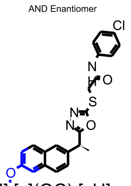 [*]:[cH]:[c](OC):[cH]:[*]	-0.780	4 out of 15
FCFP_10	-1371139928	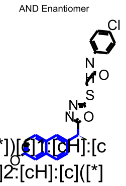 [*]C([*])[c]1:[cH]:[cH]:[c]2:[cH]:[c]([*]):[*]:[cH]:[c]:2:[cH]:1	-0.507	0 out of 1

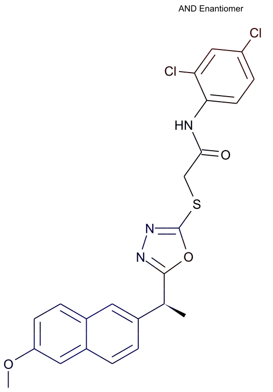

$C_{23}H_{19}Cl_2N_3O_3S$
Molecular Weight: 488.38626
ALogP: 5.567
Rotatable Bonds: 7
Acceptors: 5
Donors: 1

Model Prediction

Prediction: Mild
Probability: 0.714
Enrichment: 1.037
Bayesian Score: -3.258
Mahalanobis Distance: 9.434
Mahalanobis Distance p-value: 0.288

Prediction: Positive if the Bayesian score is above the estimated best cutoff value from minimizing the false positive and false negative rate.
Probability: The esimated probability that the sample is in the positive category. This assumes that the Bayesian score follows a normal distribution and is different from the prediction using a cutoff.
Enrichment: An estimate of enrichment, that is, the increased likelihood (versus random) of this sample being in the category.
Bayesian Score: The standard Laplacian-modified Bayesian score.
Mahalanobis Distance: The Mahalanobis distance (MD) is the distance to the center of the training data. The larger the MD, the less trustworthy the prediction.
Mahalanobis Distance p-value: The p-value gives the fraction of training data with an MD greater than or equal to the one for the given sample, assuming normally distributed data. The smaller the p-value, the less trustworthy the prediciton. For highly non-normal X properties (e.g., fingerprints), the MD p-value is wildly inaccurate.

Structural Similar Compounds

Name	ANTHRAQUINONE; 1;1'-IMINODI-	2-(1'-ANTHRAQUINONYL)-AMINOBENZANTHRONE	1-BENZOYLAMINO-4-METHOXY-5-CHLORANTHRAQUINONE
Structure	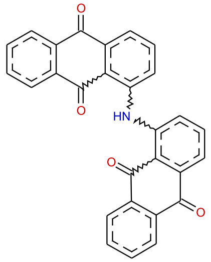	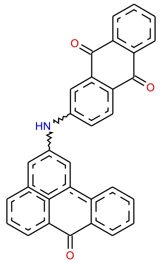	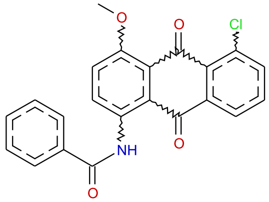
Actual Endpoint	Mild	Mild	Mild
Predicted Endpoint	Mild	Mild	Mild
Distance	0.644	0.684	0.702
Reference	28ZPAK-;125;72	28ZPAK-;126;72	28ZPAK-;90;72

Model Applicability

Unknown features are fingerprint features in the query molecule, but not found in the training set.

1. All properties and OPS components are within expected ranges.
2. Unknown FCFP_2 feature: -928857652: [*]:[c](:[*])C(C)[c](:[*]):[*]

Feature Contribution

Top features for positive contribution				
**Fingerprint**	**Bit/Smiles**	**Feature Structure**	**Score**	**Moderate_Severe in training set**
FCFP_10	73264552	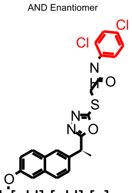 [*][c]1:[cH]:[cH]:[c](Cl):[cH]:[c]:1Cl	0.317	4 out of 4

FCFP_10	-745491832	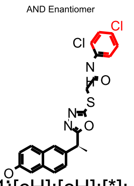 Cl[c]1:[cH]:[cH]:[*]:[cH]:[cH]:1	0.304	29 out of 32
FCFP_10	-1539162406	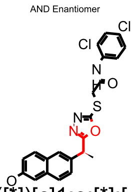 [*]C([*])[c]1:o:[*]:[*]:n:1	0.294	3 out of 3

Top Features for negative contribution

Fingerprint	Bit/Smiles	Feature Structure	Score	Moderate_Severe in training set
FCFP_10	4427049	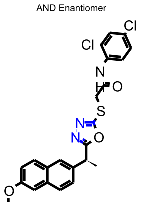 [*][c]1:[*]:[*]:n:n:1	-1.293	0 out of 4
FCFP_10	-1977641857	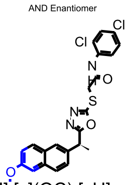 [*]:[cH]:[c](OC):[cH]:[*]	-0.780	4 out of 15
FCFP_10	-1371139928	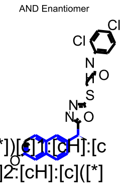 [*]C([*])[c]1:[cH]:[cH]:[c]2:[cH]:[c]([*]):[*]:[cH]:[c]:2:[cH]:1	-0.507	0 out of 1

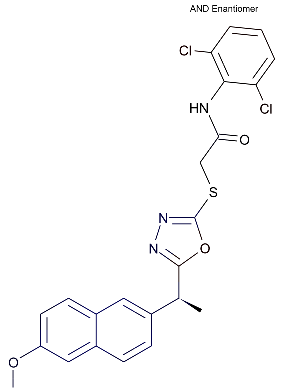

$C_{23}H_{19}Cl_2N_3O_3S$
Molecular Weight: 488.38626
ALogP: 5.567
Rotatable Bonds: 7
Acceptors: 5
Donors: 1

Model Prediction

Prediction: Mild

Probability: 0.684

Enrichment: 0.993

Bayesian Score: -3.792

Mahalanobis Distance: 9.434

Mahalanobis Distance p-value: 0.288

Prediction: Positive if the Bayesian score is above the estimated best cutoff value from minimizing the false positive and false negative rate.
Probability: The esimated probability that the sample is in the positive category. This assumes that the Bayesian score follows a normal distribution and is different from the prediction using a cutoff.
Enrichment: An estimate of enrichment, that is, the increased likelihood (versus random) of this sample being in the category.
Bayesian Score: The standard Laplacian-modified Bayesian score.
Mahalanobis Distance: The Mahalanobis distance (MD) is the distance to the center of the training data. The larger the MD, the less trustworthy the prediction.
Mahalanobis Distance p-value: The p-value gives the fraction of training data with an MD greater than or equal to the one for the given sample, assuming normally distributed data. The smaller the p-value, the less trustworthy the prediciton. For highly non-normal X properties (e.g., fingerprints), the MD p-value is wildly inaccurate.

Structural Similar Compounds

Name	ANTHRAQUINONE; 1;1'-IMINODI-	2-(1'-ANTHRAQUINONYL)-AMINOBENZANTHRONE	1-BENZOYLAMINO-4-METHOXY-5-CHLORANTHRAQUINONE
Structure	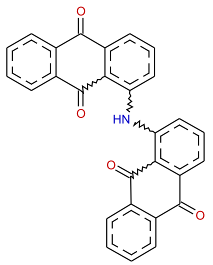	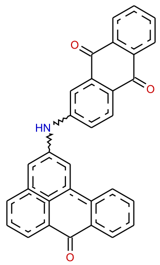	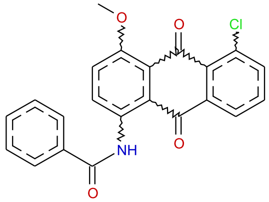
Actual Endpoint	Mild	Mild	Mild
Predicted Endpoint	Mild	Mild	Mild
Distance	0.643	0.684	0.700
Reference	28ZPAK-;125;72	28ZPAK-;126;72	28ZPAK-;90;72

Model Applicability

Unknown features are fingerprint features in the query molecule, but not found in the training set.

1. All properties and OPS components are within expected ranges.
2. Unknown FCFP_2 feature: -928857652: [*]:[c](:[*])C(C)[c](:[*]):[*]

Feature Contribution

Top features for positive contribution				
Fingerprint	Bit/Smiles	Feature Structure	Score	Moderate_Severe in training set
FCFP_10	-1539162406	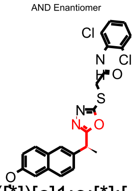 [*]C([*])[c]1:o:[*]:[*]:n:1	0.294	3 out of 3

FCFP_10	346218766	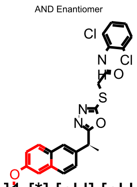 [*]:[c]1:[*]:[cH]:[cH]:[c](OC):[cH]:1	0.197	30 out of 37
FCFP_10	1161767339	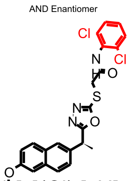 [*][c]1:[c](Cl):[cH]: [cH]:[cH]:[c]:1Cl	0.186	1 out of 1
**Top Features for negative contribution**				
**Fingerprint**	**Bit/Smiles**	**Feature Structure**	**Score**	**Moderate_Severe in training set**
FCFP_10	4427049	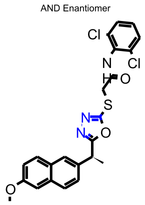 [*][c]1:[*]:[*]:n:n:1	-1.293	0 out of 4
FCFP_10	-1977641857	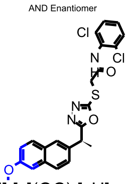 [*]:[cH]:[c](OC):[cH] :[*]	-0.780	4 out of 15
FCFP_10	-1371139928	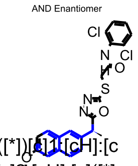 [*]C([*])[c]1:[cH]:[c H]:[c]2:[cH]:[c]([*]):[*]:[cH]:[c]:2:[cH]:1	-0.507	0 out of 1

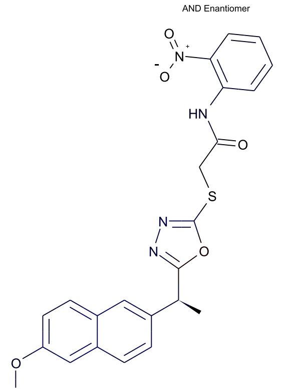

$C_{23}H_{20}N_4O_5S$
Molecular Weight: 464.4937
ALogP: 4.132
Rotatable Bonds: 8
Acceptors: 7
Donors: 1

Structural Similar Compounds

Name	COLCHICINE	ANTHRAQUINONE; 1;1'-IMINODI-	1;8;9-ANTHRACENETRIOL; TRIACETATE
Structure	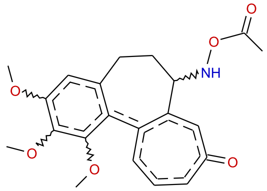	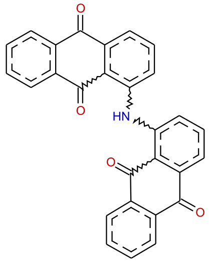	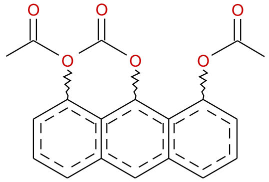
Actual Endpoint	Moderate_Severe	Mild	Moderate_Severe
Predicted Endpoint	Moderate_Severe	Mild	Moderate_Severe
Distance	0.753	0.790	0.820
Reference	AJOPAA 31;837;48	28ZPAK-;125;72	BJOPAL 53;819;69

Model Prediction

Prediction: Mild
Probability: 0.625
Enrichment: 0.907
Bayesian Score: -4.685
Mahalanobis Distance: 8.802
Mahalanobis Distance p-value: 0.618

Prediction: Positive if the Bayesian score is above the estimated best cutoff value from minimizing the false positive and false negative rate.
Probability: The esimated probability that the sample is in the positive category. This assumes that the Bayesian score follows a normal distribution and is different from the prediction using a cutoff.
Enrichment: An estimate of enrichment, that is, the increased likelihood (versus random) of this sample being in the category.
Bayesian Score: The standard Laplacian-modified Bayesian score.
Mahalanobis Distance: The Mahalanobis distance (MD) is the distance to the center of the training data. The larger the MD, the less trustworthy the prediction.
Mahalanobis Distance p-value: The p-value gives the fraction of training data with an MD greater than or equal to the one for the given sample, assuming normally distributed data. The smaller the p-value, the less trustworthy the prediciton. For highly non-normal X properties (e.g., fingerprints), the MD p-value is wildly inaccurate.

Model Applicability

Unknown features are fingerprint features in the query molecule, but not found in the training set.

1. All properties and OPS components are within expected ranges.
2. Unknown FCFP_2 feature: -928857652: [*]:[c](:[*])C(C)[c](:[*]):[*]
3. Unknown FCFP_2 feature: -828984032: [*][c](:[*]):[c]([N+](=[*])[*]):c:[*]
4. Unknown FCFP_2 feature: -1338588315: [*]:[c](:[*])[N+](=O)[O-]
5. Unknown FCFP_2 feature: 1872392852: [*][N+](=O)[*]
6. Unknown FCFP_2 feature: 260476081: [*][N+](=[*])[O-]

Feature Contribution

Top features for positive contribution

Fingerprint	Bit/Smiles	Feature Structure	Score	Moderate_Severe in training set
FCFP_10	-1539162406	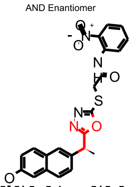 [*]C([*])[c]1:o:[*]:[*]:n:1	0.294	3 out of 3

FCFP_10	346218766	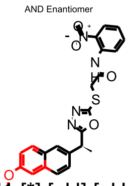<br>[*]:[c]1:[*]:[cH]:[cH]:[c](OC):[cH]:1	0.197	30 out of 37
FCFP_10	-1370111440	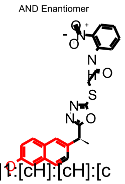<br>[*]O[c]1:[cH]:[cH]:[c]2:[cH]:[c]([*]):[*]:[cH]:[c]:2:[cH]:1	0.186	1 out of 1
**Top Features for negative contribution**				
**Fingerprint**	**Bit/Smiles**	**Feature Structure**	**Score**	**Moderate_Severe in training set**
FCFP_10	4427049	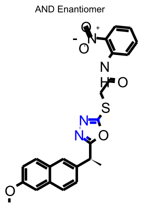<br>[*][c]1:[*]:[*]:n:n:1	-1.293	0 out of 4
FCFP_10	-1977641857	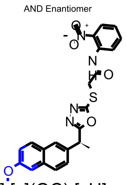<br>[*]:[cH]:[c](OC):[cH]:[*]	-0.780	4 out of 15
FCFP_10	-1371139928	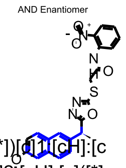<br>[*]C([*])[c]1:[cH]:[cH]:[c]2:[cH]:[c]([*]):[*]:[cH]:[c]:2:[cH]:1	-0.507	0 out of 1

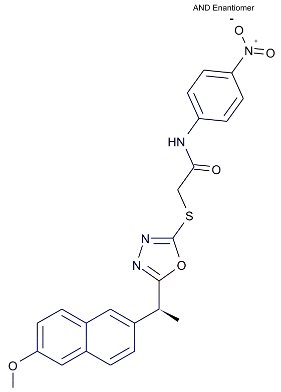

$C_{23}H_{20}N_4O_5S$
Molecular Weight: 464.4937
ALogP: 4.132
Rotatable Bonds: 8
Acceptors: 7
Donors: 1

Model Prediction

Prediction: Mild
Probability: 0.631
Enrichment: 0.916
Bayesian Score: -4.597
Mahalanobis Distance: 8.802
Mahalanobis Distance p-value: 0.618

Prediction: Positive if the Bayesian score is above the estimated best cutoff value from minimizing the false positive and false negative rate.
Probability: The esimated probability that the sample is in the positive category. This assumes that the Bayesian score follows a normal distribution and is different from the prediction using a cutoff.
Enrichment: An estimate of enrichment, that is, the increased likelihood (versus random) of this sample being in the category.
Bayesian Score: The standard Laplacian-modified Bayesian score.
Mahalanobis Distance: The Mahalanobis distance (MD) is the distance to the center of the training data. The larger the MD, the less trustworthy the prediction.
Mahalanobis Distance p-value: The p-value gives the fraction of training data with an MD greater than or equal to the one for the given sample, assuming normally distributed data. The smaller the p-value, the less trustworthy the prediciton. For highly non-normal X properties (e.g., fingerprints), the MD p-value is wildly inaccurate.

Structural Similar Compounds

Name	COLCHICINE	ANTHRAQUINONE; 1;1'-IMINODI-	1;8;9-ANTHRACENETRIOL; TRIACETATE
Structure	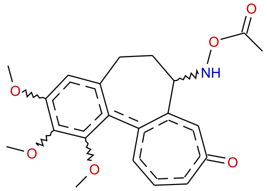	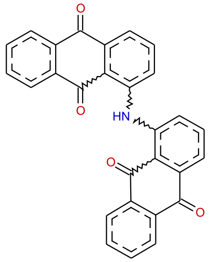	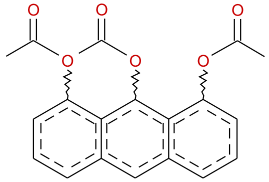
Actual Endpoint	Moderate_Severe	Mild	Moderate_Severe
Predicted Endpoint	Moderate_Severe	Mild	Moderate_Severe
Distance	0.753	0.794	0.824
Reference	AJOPAA 31;837;48	28ZPAK-;125;72	BJOPAL 53;819;69

Model Applicability

Unknown features are fingerprint features in the query molecule, but not found in the training set.

1. All properties and OPS components are within expected ranges.
2. Unknown FCFP_2 feature: -928857652: [*]:[c](:[*])C(C)[c](:[*]):[*]
3. Unknown FCFP_2 feature: -828984032: [*][c](:[*]):[c]([N+](=[*])[*]):c:[*]
4. Unknown FCFP_2 feature: -1338588315: [*]:[c](:[*])[N+](=O)[O-]
5. Unknown FCFP_2 feature: 1872392852: [*][N+](=O)[*]
6. Unknown FCFP_2 feature: 260476081: [*][N+](=[*])[O-]

Feature Contribution

Top features for positive contribution				
**Fingerprint**	**Bit/Smiles**	**Feature Structure**	**Score**	**Moderate_Severe in training set**
FCFP_10	-1539162406	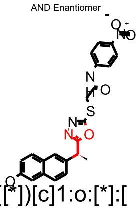 [*]C([*])[c]1:o:[*]:[*]:n:1	0.294	3 out of 3

FCFP_10	346218766	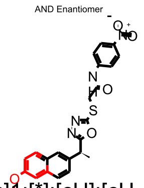 [*]:[c]1:[*]:[cH]:[cH]:[c](OC):[cH]:1	0.197	30 out of 37
FCFP_10	-1370111440	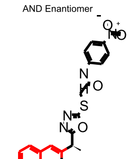 [*]O[c]1:[cH]:[cH]:[c]2:[cH]:[c]([*]):[*]:[cH]:[c]:2:[cH]:1	0.186	1 out of 1

Top Features for negative contribution

**Fingerprint**	**Bit/Smiles**	**Feature Structure**	**Score**	**Moderate_Severe in training set**
FCFP_10	4427049	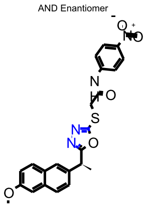 [*][c]1:[*]:[*]:n:n:1	-1.293	0 out of 4
FCFP_10	-1977641857	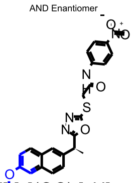 [*]:[cH]:[c](OC):[cH]:[*]	-0.780	4 out of 15
FCFP_10	-1371139928	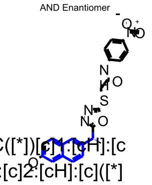 [*]C([*])[c]1:[cH]:[cH]:[c]2:[cH]:[c]([*]):[*]:[cH]:[c]:2:[cH]:1	-0.507	0 out of 1

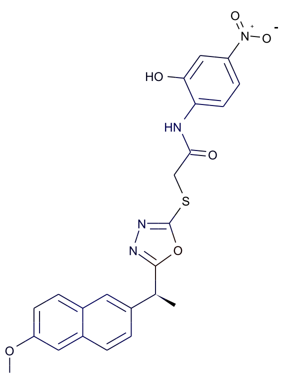

$C_{23}H_{20}N_4O_6S$
Molecular Weight: 480.4931
ALogP: 3.89
Rotatable Bonds: 8
Acceptors: 8
Donors: 2

Model Prediction

Prediction: Mild
Probability: 0.591
Enrichment: 0.858
Bayesian Score: -5.126
Mahalanobis Distance: 9.646
Mahalanobis Distance p-value: 0.2

Prediction: Positive if the Bayesian score is above the estimated best cutoff value from minimizing the false positive and false negative rate.
Probability: The esimated probability that the sample is in the positive category. This assumes that the Bayesian score follows a normal distribution and is different from the prediction using a cutoff.
Enrichment: An estimate of enrichment, that is, the increased likelihood (versus random) of this sample being in the category.
Bayesian Score: The standard Laplacian-modified Bayesian score.
Mahalanobis Distance: The Mahalanobis distance (MD) is the distance to the center of the training data. The larger the MD, the less trustworthy the prediction.
Mahalanobis Distance p-value: The p-value gives the fraction of training data with an MD greater than or equal to the one for the given sample, assuming normally distributed data. The smaller the p-value, the less trustworthy the prediciton. For highly non-normal X properties (e.g., fingerprints), the MD p-value is wildly inaccurate.

Structural Similar Compounds

Name	4;4'-DIAMINO-1;1'-DIANTHRIMIDE	2;7-NAPHTHALENE DISULFONIC ACID;4-AMINO-5-HYDROXY-;P-TOLUENE SULFONATE (ESTER)	COLCHICINE
Structure	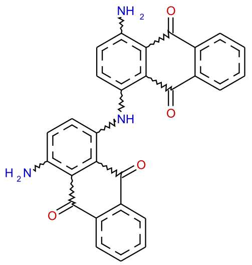	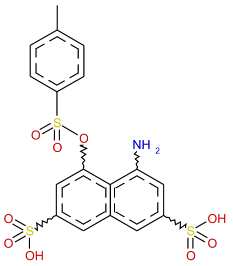	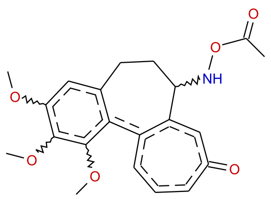
Actual Endpoint	Mild	Moderate_Severe	Moderate_Severe
Predicted Endpoint	Mild	Moderate_Severe	Moderate_Severe
Distance	0.776	0.859	0.869
Reference	28ZPAK-;125;72	28ZPAK-;194;72	AJOPAA 31;837;48

Model Applicability

Unknown features are fingerprint features in the query molecule, but not found in the training set.

1. All properties and OPS components are within expected ranges.
2. Unknown FCFP_2 feature: -928857652: [*]:[c](:[*])C(C)[c](:[*]):[*]
3. Unknown FCFP_2 feature: -828984032: [*][c](:[*]):[c]([N+](=[*])[*]):c:[*]
4. Unknown FCFP_2 feature: -1338588315: [*]:[c](:[*])[N+](=O)[O-]
5. Unknown FCFP_2 feature: 1872392852: [*][N+](=O)[*]
6. Unknown FCFP_2 feature: 260476081: [*][N+](=[*])[O-]

Feature Contribution

Top features for positive contribution

Fingerprint	Bit/Smiles	Feature Structure	Score	Moderate_Severe in training set

FCFP_10	-1539162406	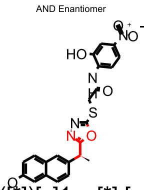 [*]C([*])[c]1:o:[*]:[*]:n:1	0.294	3 out of 3
FCFP_10	7	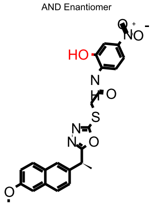 [*]O	0.219	117 out of 142
FCFP_10	346218766	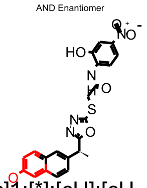 [*]:[c]1:[*]:[cH]:[cH]:[c](OC):[cH]:1	0.197	30 out of 37
**Top Features for negative contribution**				
**Fingerprint**	**Bit/Smiles**	**Feature Structure**	**Score**	**Moderate_Severe in training set**
FCFP_10	4427049	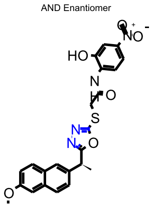 [*][c]1:[*]:[*]:n:n:1	-1.293	0 out of 4
FCFP_10	-1977641857	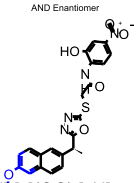 [*]:[cH]:[c](OC):[cH]:[*]	-0.780	4 out of 15

FCFP_10	-1883332927	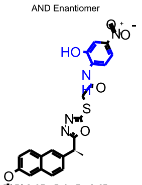 [*]C(=[*])N[c]1:[cH]:[cH]:[*]:[cH]:[c]:1O	-0.507	0 out of 1

Sorafenib

TOPKAT_Ocular_Irritancy_Mild_vs_Moderate_Severe

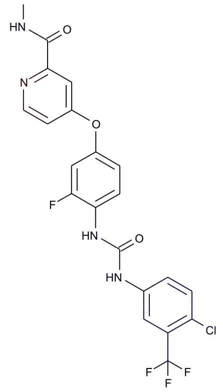

$C_{21}H_{15}ClF_4N_4O_3$
Molecular Weight: 482.81541
ALogP: 4.381
Rotatable Bonds: 6
Acceptors: 4
Donors: 3

Model Prediction

Prediction: Mild
Probability: 0.769
Enrichment: 1.116
Bayesian Score: -2.018
Mahalanobis Distance: 9.088
Mahalanobis Distance p-value: 0.464

Prediction: Positive if the Bayesian score is above the estimated best cutoff value from minimizing the false positive and false negative rate.
Probability: The esimated probability that the sample is in the positive category. This assumes that the Bayesian score follows a normal distribution and is different from the prediction using a cutoff.
Enrichment: An estimate of enrichment, that is, the increased likelihood (versus random) of this sample being in the category.
Bayesian Score: The standard Laplacian-modified Bayesian score.
Mahalanobis Distance: The Mahalanobis distance (MD) is the distance to the center of the training data. The larger the MD, the less trustworthy the prediction.
Mahalanobis Distance p-value: The p-value gives the fraction of training data with an MD greater than or equal to the one for the given sample, assuming normally distributed data. The smaller the p-value, the less trustworthy the prediciton. For highly non-normal X properties (e.g., fingerprints), the MD p-value is wildly inaccurate.

Structural Similar Compounds

Name	4;4'-DIAMINO-1;1'-DIANTHRIMIDE	ANTHRAQUINONE; 1;4-BIS(p-TOLYLAMINO)-	5-NORBORNENE-2;3-DICARBOXYLIC ACID; 1;4;5;6;7;7-HEXACHLORO-
Structure	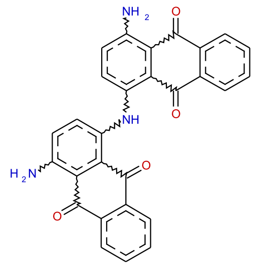	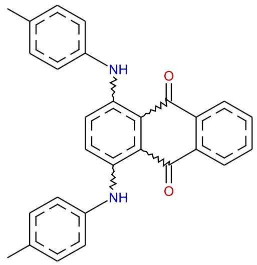	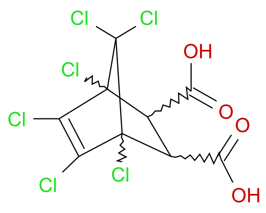
Actual Endpoint	Mild	Moderate_Severe	Moderate_Severe
Predicted Endpoint	Mild	Mild	Moderate_Severe
Distance	0.809	0.841	0.842
Reference	28ZPAK-;125;72	28ZPAK -;124;72	28ZPAK-;92;72

Model Applicability

Unknown features are fingerprint features in the query molecule, but not found in the training set.

1. All properties and OPS components are within expected ranges.

Feature Contribution

Top features for positive contribution				
**Fingerprint**	**Bit/Smiles**	**Feature Structure**	**Score**	**Moderate_Severe in training set**
FCFP_10	-1695756380	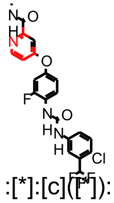 [*][c]1:[*]:[c]([*]):n:[cH]:[cH]:1	0.285	10 out of 11

FCFP_10	-124655670	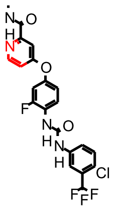 [*]:[cH]:[cH]:n:[*]	0.259	14 out of 16
FCFP_10	-885550502	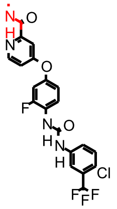 [*]CNC(=[*])[*]	0.239	54 out of 64

Top Features for negative contribution

Fingerprint	Bit/Smiles	Feature Structure	Score	Moderate_Severe in training set
FCFP_10	2104062943	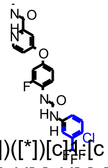 [*]C([*])([*])[c]1:[cH]:[*]:[cH]:[cH]:[c]:1Cl	-0.745	7 out of 24
FCFP_10	-174293376	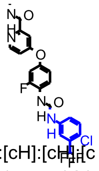 [*]N[c]1:[cH]:[cH]:[c](Cl):[c](:[cH]:1)C([*])([*])[*]	-0.507	0 out of 1
FCFP_10	-1549103449	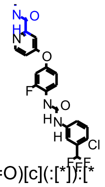 [*]NC(=O)[c](:[*]):[*]	-0.504	2 out of 6

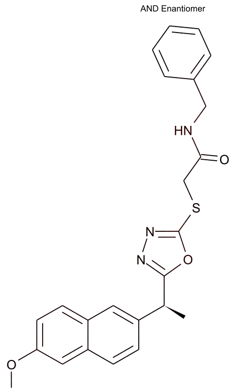

$C_{24}H_{23}N_3O_3S$
Molecular Weight: 433.52272
ALogP: 4.245
Rotatable Bonds: 8
Acceptors: 5
Donors: 1

Structural Similar Compounds

Name	1-BENZOYLAMINO-4-METHOXY-5-CHLORANTHRAQUINONE	ANTHRAQUINONE; 1;1'-IMINODI-	Cinchoninamide; 2-butoxy-N-(2-(diethylamino)ethyl)-; monohydrochloride
Structure	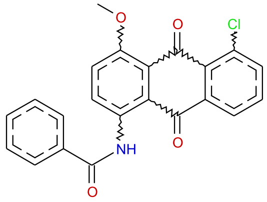	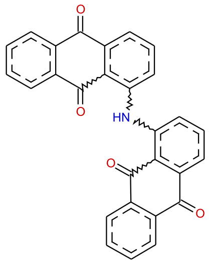	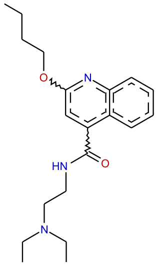
Actual Endpoint	Irritant	Irritant	Irritant
Predicted Endpoint	Irritant	Irritant	Irritant
Distance	0.634	0.654	0.683
Reference	28ZPAK-;90;72	28ZPAK-;125;72	Arzneimittel-Forschung 8;181;58

Model Prediction

Prediction: Irritant
Probability: 1.000
Enrichment: 1.176
Bayesian Score: 1.841
Mahalanobis Distance: 8.312
Mahalanobis Distance p-value: 0.839

Prediction: Positive if the Bayesian score is above the estimated best cutoff value from minimizing the false positive and false negative rate.
Probability: The esimated probability that the sample is in the positive category. This assumes that the Bayesian score follows a normal distribution and is different from the prediction using a cutoff.
Enrichment: An estimate of enrichment, that is, the increased likelihood (versus random) of this sample being in the category.
Bayesian Score: The standard Laplacian-modified Bayesian score.
Mahalanobis Distance: The Mahalanobis distance (MD) is the distance to the center of the training data. The larger the MD, the less trustworthy the prediction.
Mahalanobis Distance p-value: The p-value gives the fraction of training data with an MD greater than or equal to the one for the given sample, assuming normally distributed data. The smaller the p-value, the less trustworthy the prediciton. For highly non-normal X properties (e.g., fingerprints), the MD p-value is wildly inaccurate.

Model Applicability

Unknown features are fingerprint features in the query molecule, but not found in the training set.

1. All properties and OPS components are within expected ranges.
2. Unknown FCFP_2 feature: -928857652: [*]:[c](:[*])C(C)[c](:[*]):[*]

Feature Contribution

Top features for positive contribution				
**Fingerprint**	**Bit/Smiles**	**Feature Structure**	**Score**	**Irritant in training set**
FCFP_12	1747237384	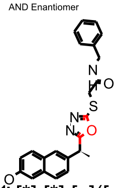 [*][c]1:[*]:[*]:[*]:[c]([*]):o:1	0.208	44 out of 44

FCFP_12	17	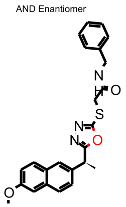 [*]:o:[*]	0.189	48 out of 49
FCFP_12	-1410079687	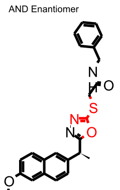 [*]S[c]1:o:[*]:[*]:n:1	0.187	8 out of 8
**Top Features for negative contribution**				
**Fingerprint**	**Bit/Smiles**	**Feature Structure**	**Score**	**Irritant in training set**
FCFP_12	-1698724694	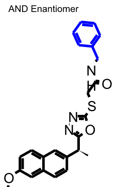 [*]C[c]1:[cH]:[cH]:[cH]:[cH]:1	-0.096	107 out of 146
FCFP_12	991735244	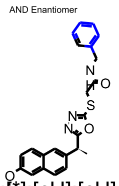 [*][c]1:[*]:[cH]:[cH]:[cH]:[cH]:1	0.000	237 out of 291
FCFP_12	-1977641857	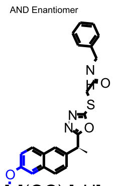 [*]:[cH]:[c](OC):[cH]:[*]	0.000	15 out of 19

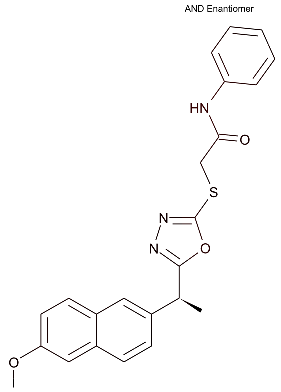

$C_{23}H_{21}N_3O_3S$
Molecular Weight: 419.49614
ALogP: 4.238
Rotatable Bonds: 7
Acceptors: 5
Donors: 1

Model Prediction

Prediction: Irritant
Probability: 1.000
Enrichment: 1.176
Bayesian Score: 2.175
Mahalanobis Distance: 8.252
Mahalanobis Distance p-value: 0.859

Prediction: Positive if the Bayesian score is above the estimated best cutoff value from minimizing the false positive and false negative rate.
Probability: The esimated probability that the sample is in the positive category. This assumes that the Bayesian score follows a normal distribution and is different from the prediction using a cutoff.
Enrichment: An estimate of enrichment, that is, the increased likelihood (versus random) of this sample being in the category.
Bayesian Score: The standard Laplacian-modified Bayesian score.
Mahalanobis Distance: The Mahalanobis distance (MD) is the distance to the center of the training data. The larger the MD, the less trustworthy the prediction.
Mahalanobis Distance p-value: The p-value gives the fraction of training data with an MD greater than or equal to the one for the given sample, assuming normally distributed data. The smaller the p-value, the less trustworthy the prediciton. For highly non-normal X properties (e.g., fingerprints), the MD p-value is wildly inaccurate.

Structural Similar Compounds

Name	1-BENZOYLAMINO-4-METHOXY-5-CHLORANTHRAQUINONE	ANTHRAQUINONE; 1;1'-IMINODI-	N;S-DIBENZOYL-O-AMINOTHIOPHENOL
Structure	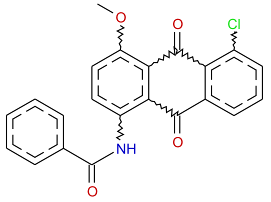	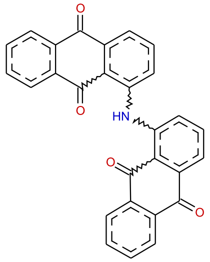	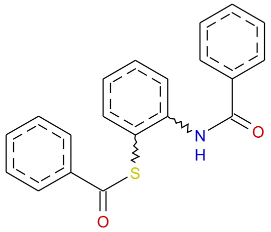
Actual Endpoint	Irritant	Irritant	Irritant
Predicted Endpoint	Irritant	Irritant	Irritant
Distance	0.605	0.633	0.683
Reference	28ZPAK-;90;72	28ZPAK-;125;72	28ZPAK-;175;72

Model Applicability

Unknown features are fingerprint features in the query molecule, but not found in the training set.

1. All properties and OPS components are within expected ranges.
2. Unknown FCFP_2 feature: -928857652: [*]:[c](:[*])C(C)[c](:[*]):[*]

Feature Contribution

Top features for positive contribution				
Fingerprint	Bit/Smiles	Feature Structure	Score	Irritant in training set
FCFP_12	1747237384	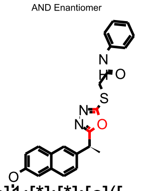 [*][c]1:[*]:[*]:[c]([*]):o:1	0.208	44 out of 44

FCFP_12	1175665944	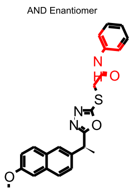 [*]CC(=O)N[c](:[cH]:[*]):[cH]:[*]	0.198	14 out of 14
FCFP_12	17	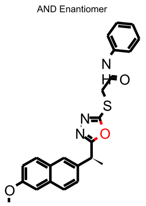 [*]:o:[*]	0.189	48 out of 49

Top Features for negative contribution

Fingerprint	Bit/Smiles	Feature Structure	Score	Irritant in training set
FCFP_12	346218766	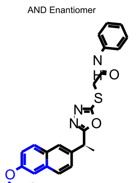 [*]:[c]1:[*]:[cH]:[cH]:[c](OC):[cH]:1	0.000	37 out of 45
FCFP_12	136627117	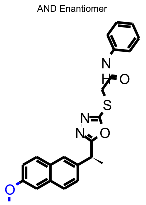 [*]OC	0.000	96 out of 113
FCFP_12	203677720	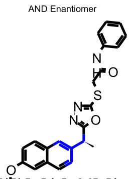 [*]C([*])[c](:[cH]:[*]):[cH]:[*]	0.000	319 out of 382

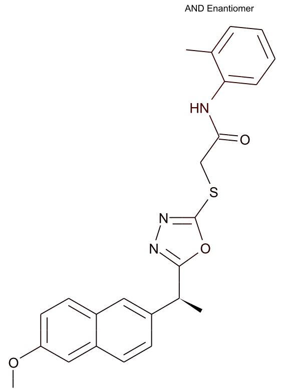

$C_{24}H_{23}N_3O_3S$
Molecular Weight: 433.52272
ALogP: 4.724
Rotatable Bonds: 7
Acceptors: 5
Donors: 1

Model Prediction

Prediction: Irritant
Probability: 1.000
Enrichment: 1.176
Bayesian Score: 2.569
Mahalanobis Distance: 8.331
Mahalanobis Distance p-value: 0.832

Prediction: Positive if the Bayesian score is above the estimated best cutoff value from minimizing the false positive and false negative rate.
Probability: The esimated probability that the sample is in the positive category. This assumes that the Bayesian score follows a normal distribution and is different from the prediction using a cutoff.
Enrichment: An estimate of enrichment, that is, the increased likelihood (versus random) of this sample being in the category.
Bayesian Score: The standard Laplacian-modified Bayesian score.
Mahalanobis Distance: The Mahalanobis distance (MD) is the distance to the center of the training data. The larger the MD, the less trustworthy the prediction.
Mahalanobis Distance p-value: The p-value gives the fraction of training data with an MD greater than or equal to the one for the given sample, assuming normally distributed data. The smaller the p-value, the less trustworthy the prediciton. For highly non-normal X properties (e.g., fingerprints), the MD p-value is wildly inaccurate.

Structural Similar Compounds

Name	ANTHRAQUINONE; 1;1'-IMINODI-	1-BENZOYLAMINO-4-METHOXY-5-CHLORANTHRAQUINONE	N;S-DIBENZOYL-O-AMINOTHIOPHENOL
Structure	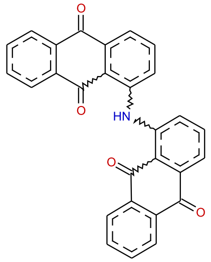	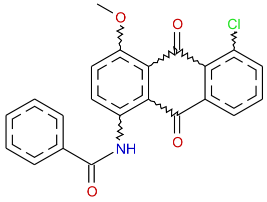	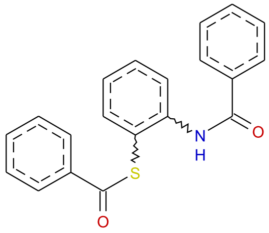
Actual Endpoint	Irritant	Irritant	Irritant
Predicted Endpoint	Irritant	Irritant	Irritant
Distance	0.605	0.611	0.695
Reference	28ZPAK-;125;72	28ZPAK-;90;72	28ZPAK-;175;72

Model Applicability

Unknown features are fingerprint features in the query molecule, but not found in the training set.

1. All properties and OPS components are within expected ranges.
2. Unknown FCFP_2 feature: -928857652: [*]:[c](:[*])C(C)[c](:[*]):[*]

Feature Contribution

Top features for positive contribution				
**Fingerprint**	**Bit/Smiles**	**Feature Structure**	**Score**	**Irritant in training set**
FCFP_12	1747237384	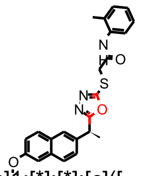 AND Enantiomer [*][c]1:[*]:[*]:[c]([*]):o:1	0.208	44 out of 44

FCFP_12	1175665944	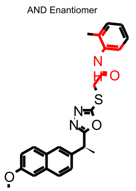 [*]CC(=O)N[c](:[cH]:[*]):[cH]:[*]	0.198	14 out of 14
FCFP_12	755520106	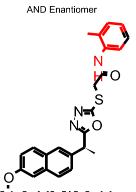 [*]N[c]1:[cH]:[*]:[cH]:[cH]:[c]:1C	0.192	10 out of 10

Top Features for negative contribution

Fingerprint	Bit/Smiles	Feature Structure	Score	Irritant in training set
FCFP_12	-1698724694	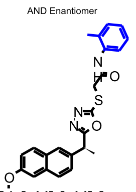 [*]C[c]1:[cH]:[cH]:[cH]:[cH]:1	-0.096	107 out of 146
FCFP_12	-773983804	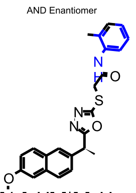 [*]N[c]1:[cH]:[*]:[cH]:[cH]:[cH]:1	0.000	102 out of 121
FCFP_12	-1977641857	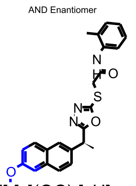 [*]:[cH]:[c](OC):[cH]:[*]	0.000	15 out of 19

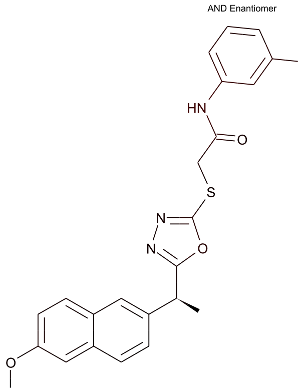

$C_{24}H_{23}N_3O_3S$
Molecular Weight: 433.52272
ALogP: 4.724
Rotatable Bonds: 7
Acceptors: 5
Donors: 1

Model Prediction

Prediction: Irritant
Probability: 1.000
Enrichment: 1.176
Bayesian Score: 2.599
Mahalanobis Distance: 8.331
Mahalanobis Distance p-value: 0.832

Prediction: Positive if the Bayesian score is above the estimated best cutoff value from minimizing the false positive and false negative rate.
Probability: The esimated probability that the sample is in the positive category. This assumes that the Bayesian score follows a normal distribution and is different from the prediction using a cutoff.
Enrichment: An estimate of enrichment, that is, the increased likelihood (versus random) of this sample being in the category.
Bayesian Score: The standard Laplacian-modified Bayesian score.
Mahalanobis Distance: The Mahalanobis distance (MD) is the distance to the center of the training data. The larger the MD, the less trustworthy the prediction.
Mahalanobis Distance p-value: The p-value gives the fraction of training data with an MD greater than or equal to the one for the given sample, assuming normally distributed data. The smaller the p-value, the less trustworthy the prediciton. For highly non-normal X properties (e.g., fingerprints), the MD p-value is wildly inaccurate.

Structural Similar Compounds

Name	ANTHRAQUINONE; 1;1'-IMINODI-	1-BENZOYLAMINO-4-METHOXY-5-CHLORANTHRAQUINONE	Cinchoninamide; 2-butoxy-N-(2-(diethylamino)ethyl)-; monohydrochloride
Structure	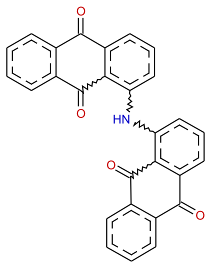	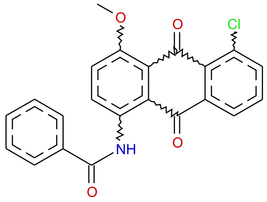	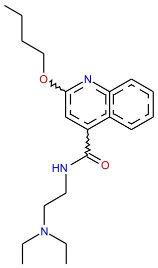
Actual Endpoint	Irritant	Irritant	Irritant
Predicted Endpoint	Irritant	Irritant	Irritant
Distance	0.608	0.618	0.701
Reference	28ZPAK-;125;72	28ZPAK-;90;72	Arzneimittel-Forschung 8;181;58

Model Applicability

Unknown features are fingerprint features in the query molecule, but not found in the training set.

1. All properties and OPS components are within expected ranges.
2. Unknown FCFP_2 feature: -928857652: [*]:[c](:[*])C(C)[c](:[*]):[*]

Feature Contribution

Top features for positive contribution				
Fingerprint	Bit/Smiles	Feature Structure	Score	Irritant in training set
FCFP_12	1747237384	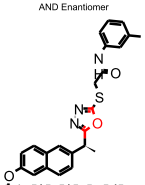 [*][c]1:[*]:[*]:[c]([*]):o:1	0.208	44 out of 44

FCFP_12	1175665944	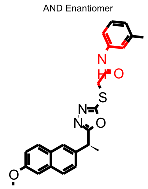<br>[*]CC(=O)N[c](:[cH]:[<br>*]):[cH]:[*]	0.198	14 out of 14
FCFP_12	17	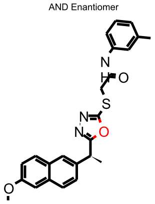<br>[*]:o:[*]	0.189	48 out of 49
**Top Features for negative contribution**				
**Fingerprint**	**Bit/Smiles**	**Feature Structure**	**Score**	**Irritant in training set**
FCFP_12	630418361	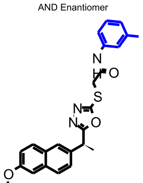<br>[*][c]1:[cH]:[cH]:[cH<br>]:[c](C):[cH]:1	0.000	4 out of 5
FCFP_12	-792685140	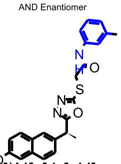<br>[*]C(=[*])N[c]1:[cH]:<br>[cH]:[cH]:[cH]:[cH]:<br>1	0.000	5 out of 6
FCFP_12	991735244	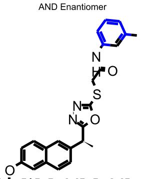<br>[*][c]1:[*]:[cH]:[cH]<br>:[cH]:[cH]:1	0.000	237 out of 291

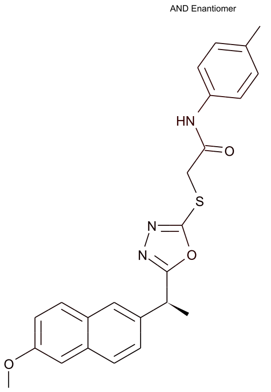

$C_{24}H_{23}N_3O_3S$
Molecular Weight: 433.52272
ALogP: 4.724
Rotatable Bonds: 7
Acceptors: 5
Donors: 1

Structural Similar Compounds

Name	ANTHRAQUINONE; 1;1'-IMINODI-	1-BENZOYLAMINO-4-METHOXY-5-CHLORANTHRAQUINONE	Cinchoninamide; 2-butoxy-N-(2-(diethylamino)ethyl)-; monohydrochloride
Structure	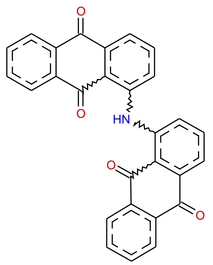	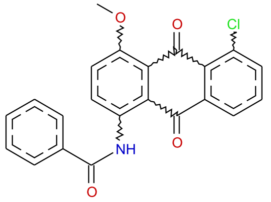	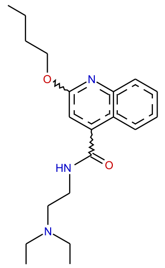
Actual Endpoint	Irritant	Irritant	Irritant
Predicted Endpoint	Irritant	Irritant	Irritant
Distance	0.612	0.621	0.704
Reference	28ZPAK-;125;72	28ZPAK-;90;72	Arzneimittel-Forschung 8;181;58

Model Prediction

Prediction: Irritant
Probability: 1.000
Enrichment: 1.176
Bayesian Score: 2.508
Mahalanobis Distance: 8.331
Mahalanobis Distance p-value: 0.832

Prediction: Positive if the Bayesian score is above the estimated best cutoff value from minimizing the false positive and false negative rate.
Probability: The esimated probability that the sample is in the positive category. This assumes that the Bayesian score follows a normal distribution and is different from the prediction using a cutoff.
Enrichment: An estimate of enrichment, that is, the increased likelihood (versus random) of this sample being in the category.
Bayesian Score: The standard Laplacian-modified Bayesian score.
Mahalanobis Distance: The Mahalanobis distance (MD) is the distance to the center of the training data. The larger the MD, the less trustworthy the prediction.
Mahalanobis Distance p-value: The p-value gives the fraction of training data with an MD greater than or equal to the one for the given sample, assuming normally distributed data. The smaller the p-value, the less trustworthy the prediciton. For highly non-normal X properties (e.g., fingerprints), the MD p-value is wildly inaccurate.

Model Applicability

Unknown features are fingerprint features in the query molecule, but not found in the training set.

1. All properties and OPS components are within expected ranges.
2. Unknown FCFP_2 feature: -928857652: [*]:[c](:[*])C(C)[c](:[*]):[*]

Feature Contribution

Top features for positive contribution				
Fingerprint	Bit/Smiles	Feature Structure	Score	Irritant in training set
FCFP_12	1747237384	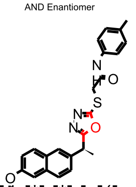 [*][c]1:[*]:[*]:[c]([*]):o:1	0.208	44 out of 44

FCFP_12	1175665944	AND Enantiomer 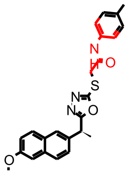 [*]CC(=O)N[c](:[cH]:[*]):[cH]:[*]	0.198	14 out of 14
FCFP_12	17	AND Enantiomer 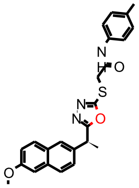 [*]:o:[*]	0.189	48 out of 49
**Top Features for negative contribution**				
**Fingerprint**	**Bit/Smiles**	**Feature Structure**	**Score**	**Irritant in training set**
FCFP_12	0	AND Enantiomer 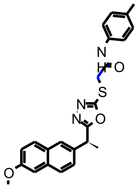 [*]C[*]	0.000	1184 out of 1397
FCFP_12	630139722	AND Enantiomer 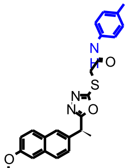 [*]N[c]1:[cH]:[cH]:[c](C):[cH]:[cH]:1	0.000	4 out of 5
FCFP_12	-773983804	AND Enantiomer 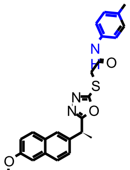 [*]N[c]1:[cH]:[*]:[cH]:[cH]:[cH]:1	0.000	102 out of 121

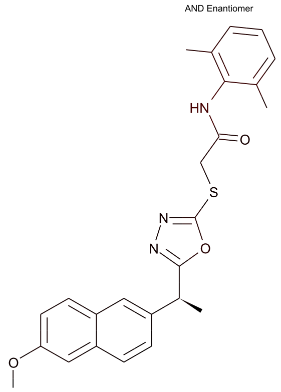

$C_{25}H_{25}N_3O_3S$
Molecular Weight: 447.5493
ALogP: 5.21
Rotatable Bonds: 7
Acceptors: 5
Donors: 1

Model Prediction

Prediction: Irritant
Probability: 1.000
Enrichment: 1.176
Bayesian Score: 2.274
Mahalanobis Distance: 8.429
Mahalanobis Distance p-value: 0.795

Prediction: Positive if the Bayesian score is above the estimated best cutoff value from minimizing the false positive and false negative rate.
Probability: The esimated probability that the sample is in the positive category. This assumes that the Bayesian score follows a normal distribution and is different from the prediction using a cutoff.
Enrichment: An estimate of enrichment, that is, the increased likelihood (versus random) of this sample being in the category.
Bayesian Score: The standard Laplacian-modified Bayesian score.
Mahalanobis Distance: The Mahalanobis distance (MD) is the distance to the center of the training data. The larger the MD, the less trustworthy the prediction.
Mahalanobis Distance p-value: The p-value gives the fraction of training data with an MD greater than or equal to the one for the given sample, assuming normally distributed data. The smaller the p-value, the less trustworthy the prediciton. For highly non-normal X properties (e.g., fingerprints), the MD p-value is wildly inaccurate.

Structural Similar Compounds

Name	ANTHRAQUINONE; 1;1'-IMINODI-	1-BENZOYLAMINO-4-METHOXY-5-CHLORANTHRAQUINONE	2-(1'-ANTHRAQUINONYL)-AMINOBENZANTHRONE
Structure	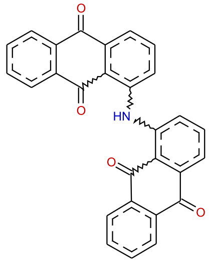	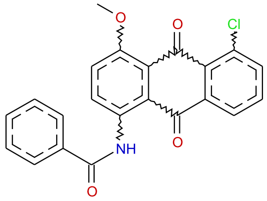	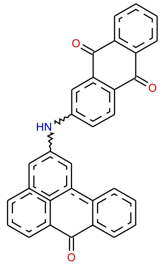
Actual Endpoint	Irritant	Irritant	Irritant
Predicted Endpoint	Irritant	Irritant	Irritant
Distance	0.602	0.643	0.676
Reference	28ZPAK-;125;72	28ZPAK-;90;72	28ZPAK-;126;72

Model Applicability

Unknown features are fingerprint features in the query molecule, but not found in the training set.

1. All properties and OPS components are within expected ranges.
2. Unknown FCFP_2 feature: -928857652: [*]:[c](:[*])C(C)[c](:[*]):[*]

Feature Contribution

Top features for positive contribution

Fingerprint	Bit/Smiles	Feature Structure	Score	Irritant in training set
FCFP_12	1747237384	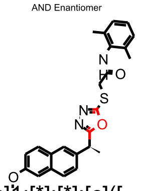 [*][c]1:[*]:[*]:[c]([*]):o:1	0.208	44 out of 44

FCFP_12	1175665944	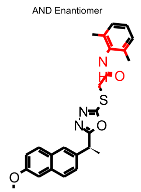 [*]CC(=O)N[c](:[cH]:[*]):[cH]:[*]	0.198	14 out of 14
FCFP_12	755520106	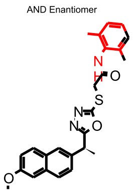 [*]N[c]1:[cH]:[*]:[cH]:[cH]:[c]:1C	0.192	10 out of 10
**Top Features for negative contribution**				
**Fingerprint**	**Bit/Smiles**	**Feature Structure**	**Score**	**Irritant in training set**
FCFP_12	-1696375691	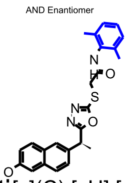 [*][c]1:[c](C):[cH]:[cH]:[cH]:[c]:1C	-0.055	16 out of 21
FCFP_12	307419094	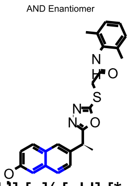 [*]:[cH]:[c](:[cH]:[*]):[c](:[*]):[*]	0.000	43 out of 52
FCFP_12	1872154524	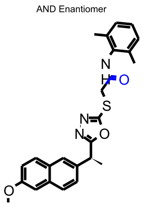 [*]C(=O)[*]	0.000	563 out of 690

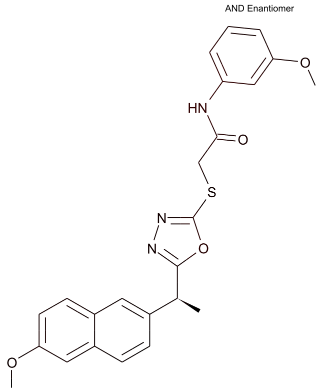

$C_{24}H_{23}N_3O_4S$

Molecular Weight: 449.52212

ALogP: 4.221

Rotatable Bonds: 8

Acceptors: 6

Donors: 1

Model Prediction

Prediction: Irritant

Probability: 1.000

Enrichment: 1.176

Bayesian Score: 2.312

Mahalanobis Distance: 8.316

Mahalanobis Distance p-value: 0.838

Prediction: Positive if the Bayesian score is above the estimated best cutoff value from minimizing the false positive and false negative rate.
Probability: The esimated probability that the sample is in the positive category. This assumes that the Bayesian score follows a normal distribution and is different from the prediction using a cutoff.
Enrichment: An estimate of enrichment, that is, the increased likelihood (versus random) of this sample being in the category.
Bayesian Score: The standard Laplacian-modified Bayesian score.
Mahalanobis Distance: The Mahalanobis distance (MD) is the distance to the center of the training data. The larger the MD, the less trustworthy the prediction.
Mahalanobis Distance p-value: The p-value gives the fraction of training data with an MD greater than or equal to the one for the given sample, assuming normally distributed data. The smaller the p-value, the less trustworthy the prediciton. For highly non-normal X properties (e.g., fingerprints), the MD p-value is wildly inaccurate.

Structural Similar Compounds

Name	ANTHRAQUINONE; 1;1'-IMINODI-	COLCHICINE	1-BENZOYLAMINO-4-METHOXY-5-CHLORANTHRAQUINONE
Structure	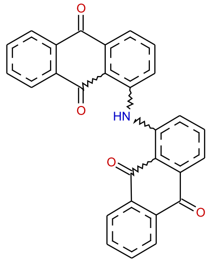	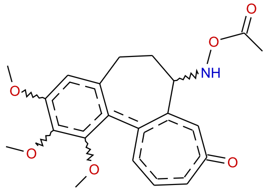	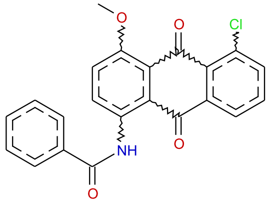
Actual Endpoint	Irritant	Irritant	Irritant
Predicted Endpoint	Irritant	Irritant	Irritant
Distance	0.678	0.700	0.702
Reference	28ZPAK-;125;72	AJOPAA 31;837;48	28ZPAK-;90;72

Model Applicability

Unknown features are fingerprint features in the query molecule, but not found in the training set.

1. All properties and OPS components are within expected ranges.
2. Unknown FCFP_2 feature: -928857652: [*]:[c](:[*])C(C)[c](:[*]):[*]

Feature Contribution

Top features for positive contribution				
**Fingerprint**	**Bit/Smiles**	**Feature Structure**	**Score**	**Irritant in training set**
FCFP_12	1747237384	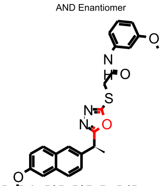 [*][c]1:[*]:[*]:[c]([*]):o:1	0.208	44 out of 44

FCFP_12	1175665944	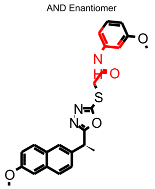 [*]CC(=O)N[c](:[cH]:[*]):[cH]:[*]	0.198	14 out of 14
FCFP_12	-1429257777	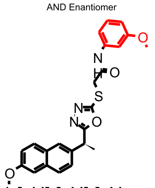 [*][c]1:[cH]:[cH]:[cH]:[c](OC):[cH]:1	0.190	9 out of 9

Top Features for negative contribution

Fingerprint	Bit/Smiles	Feature Structure	Score	Irritant in training set
FCFP_12	-453677277	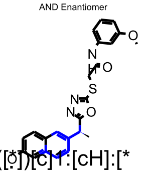 [*]C([*])[c]1:[cH]:[*]:[c](:[*]):[cH]:[cH]:1	0.000	264 out of 323
FCFP_12	-105186863	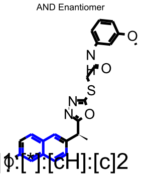 [*][c]1:[*]:[cH]:[c]2 :[cH]:[*]:[cH]:[cH]: [c]:2:[cH]:1	0.000	27 out of 32
FCFP_12	346218766	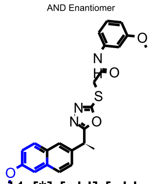 [*]:[c]1:[*]:[cH]:[cH]:[c](OC):[cH]:1	0.000	37 out of 45

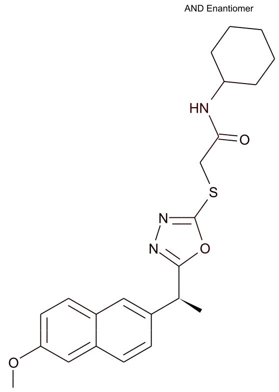

$C_{23}H_{27}N_3O_3S$
Molecular Weight: 425.54378
ALogP: 4.52
Rotatable Bonds: 7
Acceptors: 5
Donors: 1

Structural Similar Compounds

Name	1-BENZOYLAMINO-4-METHOXY-5-CHLORANTHRAQUINONE	ANTHRAQUINONE; 1;1'-IMINODI-	Cinchoninamide; 2-butoxy-N-(2-(diethylamino)ethyl)-; monohydrochloride
Structure	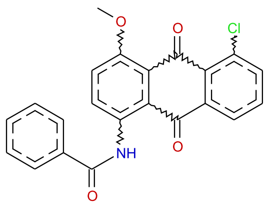	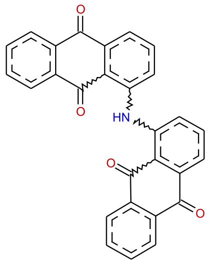	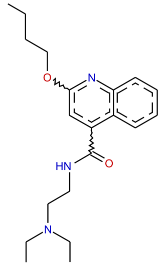
Actual Endpoint	Irritant	Irritant	Irritant
Predicted Endpoint	Irritant	Irritant	Irritant
Distance	0.623	0.628	0.689
Reference	28ZPAK-;90;72	28ZPAK-;125;72	Arzneimittel-Forschung 8;181;58

Model Prediction

Prediction: Irritant
Probability: 1.000
Enrichment: 1.176
Bayesian Score: 1.917
Mahalanobis Distance: 11.177
Mahalanobis Distance p-value: 0.00229

Prediction: Positive if the Bayesian score is above the estimated best cutoff value from minimizing the false positive and false negative rate.
Probability: The esimated probability that the sample is in the positive category. This assumes that the Bayesian score follows a normal distribution and is different from the prediction using a cutoff.
Enrichment: An estimate of enrichment, that is, the increased likelihood (versus random) of this sample being in the category.
Bayesian Score: The standard Laplacian-modified Bayesian score.
Mahalanobis Distance: The Mahalanobis distance (MD) is the distance to the center of the training data. The larger the MD, the less trustworthy the prediction.
Mahalanobis Distance p-value: The p-value gives the fraction of training data with an MD greater than or equal to the one for the given sample, assuming normally distributed data. The smaller the p-value, the less trustworthy the prediciton. For highly non-normal X properties (e.g., fingerprints), the MD p-value is wildly inaccurate.

Model Applicability

Unknown features are fingerprint features in the query molecule, but not found in the training set.

1. All properties and OPS components are within expected ranges.
2. Unknown FCFP_2 feature: -928857652: [*]:[c](:[*])C(C)[c](:[*]):[*]

Feature Contribution

Top features for positive contribution				
**Fingerprint**	**Bit/Smiles**	**Feature Structure**	**Score**	**Irritant in training set**
FCFP_12	1747237384	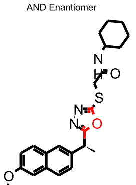 [*][c]1:[*]:[*]:[c]([*]):o:1	0.208	44 out of 44

FCFP_12	17	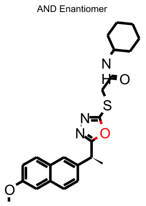<br>[*]:o:[*]	0.189	48 out of 49
FCFP_12	-1410079687	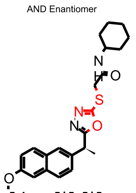<br>[*]S[c]1:o:[*]:[*]:n:1	0.187	8 out of 8

Top Features for negative contribution

Fingerprint	Bit/Smiles	Feature Structure	Score	Irritant in training set
FCFP_12	1175638033	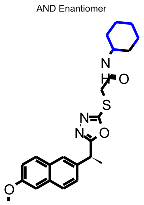<br>[*]C1[*]CCCC1	-0.133	207 out of 293
FCFP_12	-1525101452	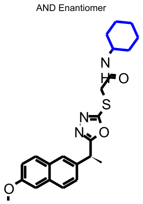<br>[*]C1CCCCC1	-0.127	108 out of 152
FCFP_12	-1317581692	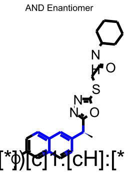<br>[*]C([*])[c]1:[cH]:[*]:[c](:[*]):[c](:[cH]:[*]):[cH]:1	0.000	11 out of 13

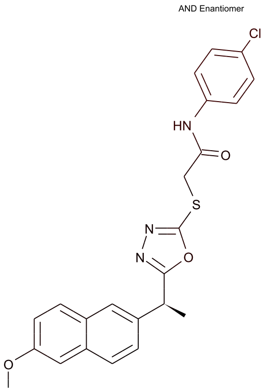

$C_{23}H_{20}ClN_3O_3S$
Molecular Weight: 453.9412
ALogP: 4.902
Rotatable Bonds: 7
Acceptors: 5
Donors: 1

Structural Similar Compounds

Name	ANTHRAQUINONE; 1;1'-IMINODI-	1-BENZOYLAMINO-4-METHOXY-5-CHLORANTHRAQUINONE	2-(1'-ANTHRAQUINONYL)-AMINOBENZANTHRONE
Structure	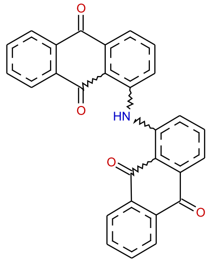	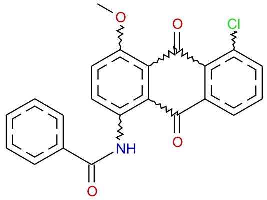	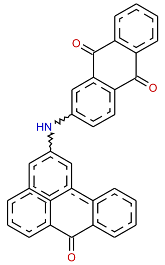
Actual Endpoint	Irritant	Irritant	Irritant
Predicted Endpoint	Irritant	Irritant	Irritant
Distance	0.613	0.630	0.698
Reference	28ZPAK-;125;72	28ZPAK-;90;72	28ZPAK-;126;72

Model Applicability

Unknown features are fingerprint features in the query molecule, but not found in the training set.

1. All properties and OPS components are within expected ranges.
2. Unknown FCFP_2 feature: -928857652: [*]:[c](:[*])C(C)[c](:[*]):[*]

Model Prediction

Prediction: Irritant
Probability: 1.000
Enrichment: 1.176
Bayesian Score: 3.218
Mahalanobis Distance: 8.489
Mahalanobis Distance p-value: 0.77

Prediction: Positive if the Bayesian score is above the estimated best cutoff value from minimizing the false positive and false negative rate.
Probability: The esimated probability that the sample is in the positive category. This assumes that the Bayesian score follows a normal distribution and is different from the prediction using a cutoff.
Enrichment: An estimate of enrichment, that is, the increased likelihood (versus random) of this sample being in the category.
Bayesian Score: The standard Laplacian-modified Bayesian score.
Mahalanobis Distance: The Mahalanobis distance (MD) is the distance to the center of the training data. The larger the MD, the less trustworthy the prediction.
Mahalanobis Distance p-value: The p-value gives the fraction of training data with an MD greater than or equal to the one for the given sample, assuming normally distributed data. The smaller the p-value, the less trustworthy the prediciton. For highly non-normal X properties (e.g., fingerprints), the MD p-value is wildly inaccurate.

Feature Contribution

Top features for positive contribution

Fingerprint	Bit/Smiles	Feature Structure	Score	Irritant in training set
FCFP_12	1747237384	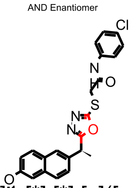 [*][c]1:[*]:[*]:[c]([*]):o:1	0.208	44 out of 44

FCFP_12	-1508180856	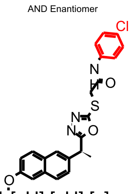 [*][c]1:[cH]:[cH]:[c](Cl):[cH]:[cH]:1	0.200	17 out of 17
FCFP_12	1175665944	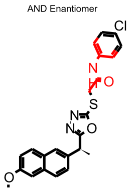 [*]CC(=O)N[c](:[cH]:[*]):[cH]:[*]	0.198	14 out of 14

Top Features for negative contribution

Fingerprint	Bit/Smiles	Feature Structure	Score	Irritant in training set
FCFP_12	-773983804	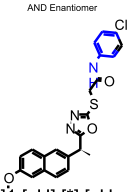 [*]N[c]1:[cH]:[*]:[cH]:[cH]:[cH]:1	0.000	102 out of 121
FCFP_12	-1977641857	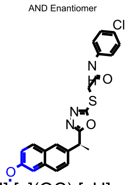 [*]:[cH]:[c](OC):[cH]:[*]	0.000	15 out of 19
FCFP_12	-1317581692	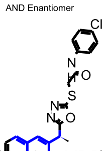 [*]C([*])[c]1:[cH]:[*]:[c](:[*]):[c](:[cH]:[*]):[cH]:1	0.000	11 out of 13

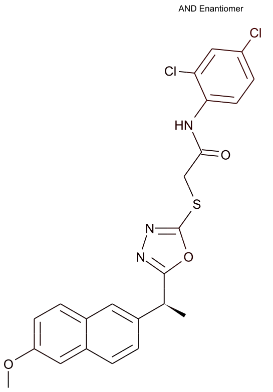

$C_{23}H_{19}Cl_2N_3O_3S$
Molecular Weight: 488.38626
ALogP: 5.567
Rotatable Bonds: 7
Acceptors: 5
Donors: 1

Structural Similar Compounds

Name	ANTHRAQUINONE; 1;1'-IMINODI-	2-(1'-ANTHRAQUINONYL)-AMINOBENZANTHRONE	1-BENZOYLAMINO-4-METHOXY-5-CHLORANTHRAQUINONE
Structure	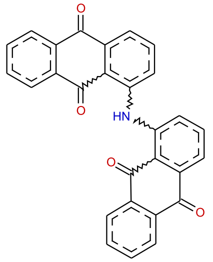	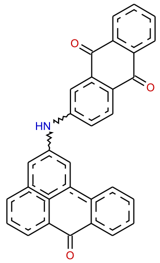	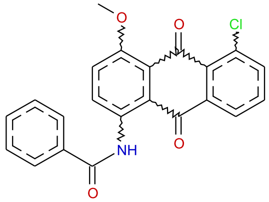
Actual Endpoint	Irritant	Irritant	Irritant
Predicted Endpoint	Irritant	Irritant	Irritant
Distance	0.633	0.673	0.687
Reference	28ZPAK-;125;72	28ZPAK-;126;72	28ZPAK-;90;72

Model Prediction

Prediction: Irritant
Probability: 1.000
Enrichment: 1.176
Bayesian Score: 2.722
Mahalanobis Distance: 8.772
Mahalanobis Distance p-value: 0.635

Prediction: Positive if the Bayesian score is above the estimated best cutoff value from minimizing the false positive and false negative rate.
Probability: The esimated probability that the sample is in the positive category. This assumes that the Bayesian score follows a normal distribution and is different from the prediction using a cutoff.
Enrichment: An estimate of enrichment, that is, the increased likelihood (versus random) of this sample being in the category.
Bayesian Score: The standard Laplacian-modified Bayesian score.
Mahalanobis Distance: The Mahalanobis distance (MD) is the distance to the center of the training data. The larger the MD, the less trustworthy the prediction.
Mahalanobis Distance p-value: The p-value gives the fraction of training data with an MD greater than or equal to the one for the given sample, assuming normally distributed data. The smaller the p-value, the less trustworthy the prediciton. For highly non-normal X properties (e.g., fingerprints), the MD p-value is wildly inaccurate.

Model Applicability

Unknown features are fingerprint features in the query molecule, but not found in the training set.

1. All properties and OPS components are within expected ranges.
2. Unknown FCFP_2 feature: -928857652: [*]:[c](:[*])C(C)[c](:[*]):[*]

Feature Contribution

Top features for positive contribution				
**Fingerprint**	**Bit/Smiles**	**Feature Structure**	**Score**	**Irritant in training set**
FCFP_12	1747237384	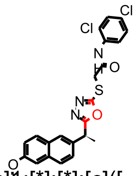 AND Enantiomer [*][c]1:[*]:[*]:[*]:[c]([*]):o:1	0.208	44 out of 44

FCFP_12	1175665944	AND Enantiomer 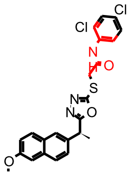 [*]CC(=O)N[c](:[cH]:[*]):[cH]:[*]	0.198	14 out of 14
FCFP_12	555188808	AND Enantiomer 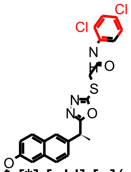 [*][c]1:[*]:[cH]:[c](Cl):[cH]:[c]:1Cl	0.195	12 out of 12

Top Features for negative contribution

Fingerprint	Bit/Smiles	Feature Structure	Score	Irritant in training set
FCFP_12	-1272768868	AND Enantiomer 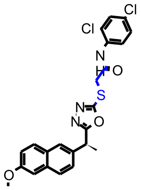 [*]SCC(=[*])[*]	0.000	396 out of 514
FCFP_12	1	AND Enantiomer 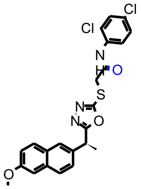 [*]=O	0.000	872 out of 1051
FCFP_12	0	AND Enantiomer 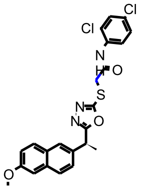 [*]C[*]	0.000	1184 out of 1397

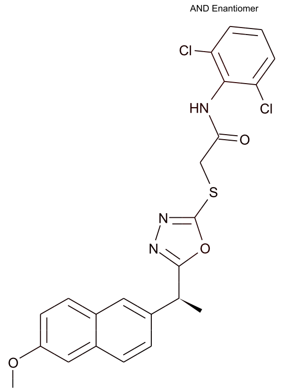

$C_{23}H_{19}Cl_2N_3O_3S$
Molecular Weight: 488.38626
ALogP: 5.567
Rotatable Bonds: 7
Acceptors: 5
Donors: 1

Model Prediction

Prediction: Irritant
Probability: 1.000
Enrichment: 1.176
Bayesian Score: 2.284
Mahalanobis Distance: 8.772
Mahalanobis Distance p-value: 0.635

Prediction: Positive if the Bayesian score is above the estimated best cutoff value from minimizing the false positive and false negative rate.
Probability: The esimated probability that the sample is in the positive category. This assumes that the Bayesian score follows a normal distribution and is different from the prediction using a cutoff.
Enrichment: An estimate of enrichment, that is, the increased likelihood (versus random) of this sample being in the category.
Bayesian Score: The standard Laplacian-modified Bayesian score.
Mahalanobis Distance: The Mahalanobis distance (MD) is the distance to the center of the training data. The larger the MD, the less trustworthy the prediction.
Mahalanobis Distance p-value: The p-value gives the fraction of training data with an MD greater than or equal to the one for the given sample, assuming normally distributed data. The smaller the p-value, the less trustworthy the prediciton. For highly non-normal X properties (e.g., fingerprints), the MD p-value is wildly inaccurate.

Structural Similar Compounds

Name	ANTHRAQUINONE; 1;1'-IMINODI-	2-(1'-ANTHRAQUINONYL)-AMINOBENZANTHRONE	1-BENZOYLAMINO-4-METHOXY-5-CHLORANTHRAQUINONE
Structure	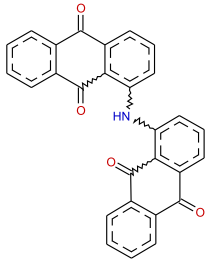	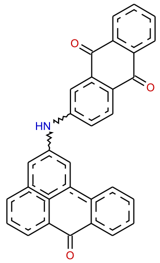	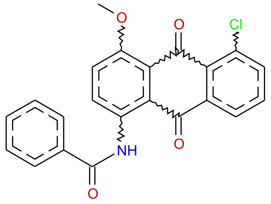
Actual Endpoint	Irritant	Irritant	Irritant
Predicted Endpoint	Irritant	Irritant	Irritant
Distance	0.632	0.672	0.686
Reference	28ZPAK-;125;72	28ZPAK-;126;72	28ZPAK-;90;72

Model Applicability

Unknown features are fingerprint features in the query molecule, but not found in the training set.

1. All properties and OPS components are within expected ranges.
2. Unknown FCFP_2 feature: -928857652: [*]:[c](:[*])C(C)[c](:[*]):[*]

Feature Contribution

Top features for positive contribution

Fingerprint	Bit/Smiles	Feature Structure	Score	Irritant in training set
FCFP_12	1747237384	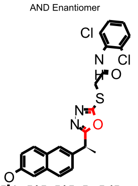 AND Enantiomer [*][c]1:[*]:[*]:[c]([*]):o:1	0.208	44 out of 44

FCFP_12	1175665944	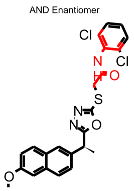 [*]CC(=O)N[c](:[cH]:[*]):[cH]:[*]	0.198	14 out of 14
FCFP_12	17	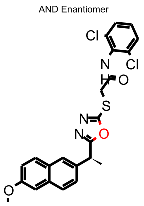 [*]:o:[*]	0.189	48 out of 49
**Top Features for negative contribution**				
**Fingerprint**	**Bit/Smiles**	**Feature Structure**	**Score**	**Irritant in training set**
FCFP_12	-1977641857	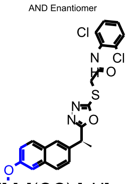 [*]:[cH]:[c](OC):[cH] :[*]	0.000	15 out of 19
FCFP_12	991735244	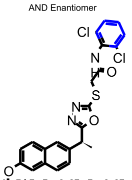 [*][c]1:[*]:[cH]:[cH] :[cH]:[cH]:1	0.000	237 out of 291
FCFP_12	1783756416	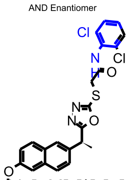 [*]N[c]1:[cH]:[*]:[c] ([*]):[cH]:[c]:1Cl	0.000	4 out of 5

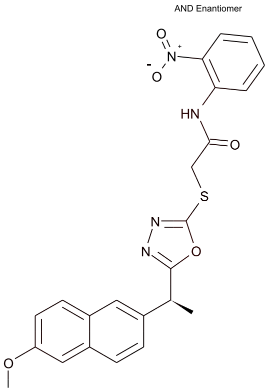

$C_{23}H_{20}N_4O_5S$

Molecular Weight: 464.4937

ALogP: 4.132

Rotatable Bonds: 8

Acceptors: 7

Donors: 1

Model Prediction

Prediction: Irritant

Probability: 1.000

Enrichment: 1.176

Bayesian Score: 1.939

Mahalanobis Distance: 8.559

Mahalanobis Distance p-value: 0.739

Prediction: Positive if the Bayesian score is above the estimated best cutoff value from minimizing the false positive and false negative rate.
Probability: The esimated probability that the sample is in the positive category. This assumes that the Bayesian score follows a normal distribution and is different from the prediction using a cutoff.
Enrichment: An estimate of enrichment, that is, the increased likelihood (versus random) of this sample being in the category.
Bayesian Score: The standard Laplacian-modified Bayesian score.
Mahalanobis Distance: The Mahalanobis distance (MD) is the distance to the center of the training data. The larger the MD, the less trustworthy the prediction.
Mahalanobis Distance p-value: The p-value gives the fraction of training data with an MD greater than or equal to the one for the given sample, assuming normally distributed data. The smaller the p-value, the less trustworthy the prediciton. For highly non-normal X properties (e.g., fingerprints), the MD p-value is wildly inaccurate.

Structural Similar Compounds

Name	COLCHICINE	ANTHRAQUINONE; 1;1'-IMINODI-	1;8;9-ANTHRACENETRIOL; TRIACETATE
Structure	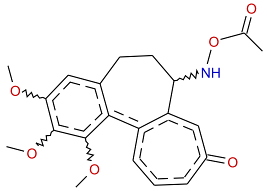	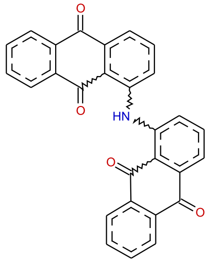	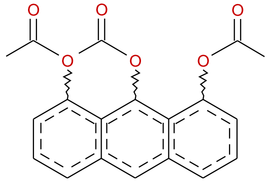
Actual Endpoint	Irritant	Irritant	Irritant
Predicted Endpoint	Irritant	Irritant	Irritant
Distance	0.738	0.776	0.813
Reference	AJOPAA 31;837;48	28ZPAK-;125;72	BJOPAL 53;819;69

Model Applicability

Unknown features are fingerprint features in the query molecule, but not found in the training set.

1. All properties and OPS components are within expected ranges.
2. Unknown FCFP_2 feature: -928857652: [*]:[c](:[*])C(C)[c](:[*]):[*]
3. Unknown FCFP_2 feature: -828984032: [*][c](:[*]):[c]([N+](=[*])[*]):c:[*]
4. Unknown FCFP_2 feature: -1338588315: [*]:[c](:[*])[N+](=O)[O-]
5. Unknown FCFP_2 feature: 1872392852: [*][N+](=O)[*]
6. Unknown FCFP_2 feature: 260476081: [*][N+](=[*])[O-]

Feature Contribution

Top features for positive contribution

Fingerprint	Bit/Smiles	Feature Structure	Score	Irritant in training set
FCFP_12	1747237384	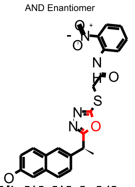 [*][c]1:[*]:[*]:[c]([*]):o:1	0.208	44 out of 44

FCFP_12	1175665944	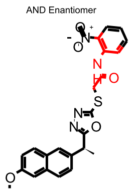 [*]CC(=O)N[c](:[cH]:[*]):[cH]:[*]	0.198	14 out of 14
FCFP_12	17	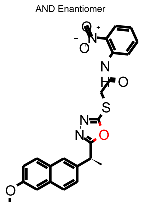 [*]:o:[*]	0.189	48 out of 49
**Top Features for negative contribution**				
**Fingerprint**	**Bit/Smiles**	**Feature Structure**	**Score**	**Irritant in training set**
FCFP_12	8	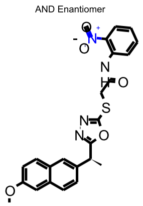 [*][N+](=[*])[*]	-0.056	3 out of 4
FCFP_12	136597326	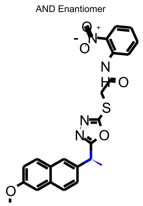 [*]C([*])C	0.000	612 out of 753
FCFP_12	136627117	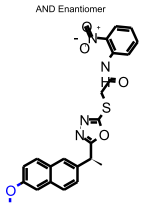 [*]OC	0.000	96 out of 113

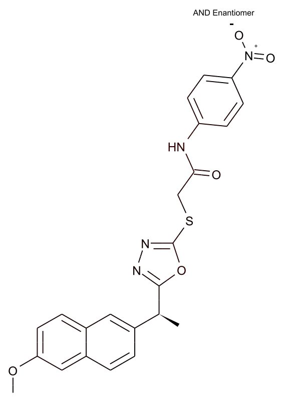

$C_{23}H_{20}N_4O_5S$
Molecular Weight: 464.4937
ALogP: 4.132
Rotatable Bonds: 8
Acceptors: 7
Donors: 1

Model Prediction

Prediction: Irritant
Probability: 1.000
Enrichment: 1.176
Bayesian Score: 2.121
Mahalanobis Distance: 8.559
Mahalanobis Distance p-value: 0.739

Prediction: Positive if the Bayesian score is above the estimated best cutoff value from minimizing the false positive and false negative rate.
Probability: The esimated probability that the sample is in the positive category. This assumes that the Bayesian score follows a normal distribution and is different from the prediction using a cutoff.
Enrichment: An estimate of enrichment, that is, the increased likelihood (versus random) of this sample being in the category.
Bayesian Score: The standard Laplacian-modified Bayesian score.
Mahalanobis Distance: The Mahalanobis distance (MD) is the distance to the center of the training data. The larger the MD, the less trustworthy the prediction.
Mahalanobis Distance p-value: The p-value gives the fraction of training data with an MD greater than or equal to the one for the given sample, assuming normally distributed data. The smaller the p-value, the less trustworthy the prediciton. For highly non-normal X properties (e.g., fingerprints), the MD p-value is wildly inaccurate.

Structural Similar Compounds

Name	COLCHICINE	ANTHRAQUINONE; 1;1'-IMINODI-	Benzoic acid; p-(N-butyl-2-(butylamino)acetamido)-; butyl ester;
Structure	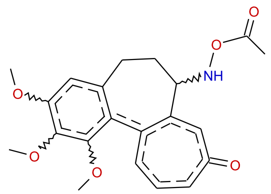	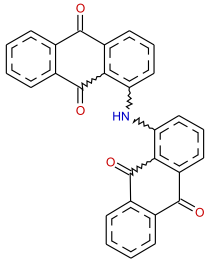	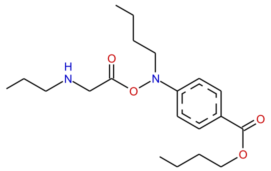
Actual Endpoint	Irritant	Irritant	Irritant
Predicted Endpoint	Irritant	Irritant	Non-Irritant
Distance	0.738	0.780	0.815
Reference	AJOPAA 31;837;48	28ZPAK-;125;72	Arzneimittel-Forschung 8;609;58

Model Applicability

Unknown features are fingerprint features in the query molecule, but not found in the training set.

1. All properties and OPS components are within expected ranges.
2. Unknown FCFP_2 feature: -928857652: [*]:[c](:[*])C(C)[c](:[*]):[*]
3. Unknown FCFP_2 feature: -828984032: [*][c](:[*]):[c]([N+](=[*])[*]):c:[*]
4. Unknown FCFP_2 feature: -1338588315: [*]:[c](:[*])[N+](=O)[O-]
5. Unknown FCFP_2 feature: 1872392852: [*][N+](=O)[*]
6. Unknown FCFP_2 feature: 260476081: [*][N+](=[*])[O-]

Feature Contribution

Top features for positive contribution				
Fingerprint	Bit/Smiles	Feature Structure	Score	Irritant in training set

FCFP_12	1747237384	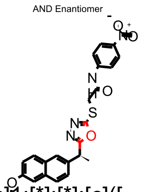 [*][c]1:[*]:[*]:[c]([*]):o:1	0.208	44 out of 44
FCFP_12	1175665944	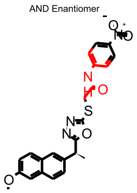 [*]CC(=O)N[c](:[cH]:[*]):[cH]:[*]	0.198	14 out of 14
FCFP_12	17	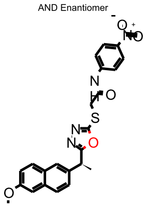 [*]:o:[*]	0.189	48 out of 49
**Top Features for negative contribution**				
**Fingerprint**	**Bit/Smiles**	**Feature Structure**	**Score**	**Irritant in training set**
FCFP_12	8	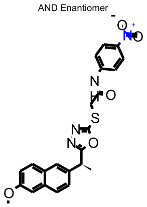 [*][N+](=[*])[*]	-0.056	3 out of 4
FCFP_12	-453677277	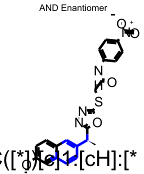 [*]C([*])[c]1:[cH]:[*]:[c](:[*]):[cH]:[cH]:1	0.000	264 out of 323

FCFP_12	0	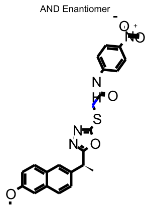<br>[*]C[*]	0.000	1184 out of 1397

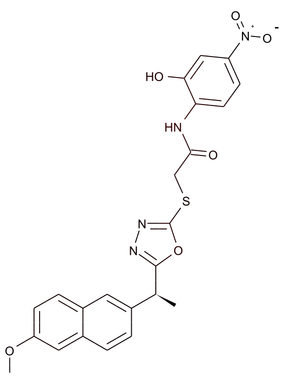

$C_{23}H_{20}N_4O_6S$
Molecular Weight: 480.4931
ALogP: 3.89
Rotatable Bonds: 8
Acceptors: 8
Donors: 2

Structural Similar Compounds

Name	4;4'-DIAMINO-1;1'-DIANTHRIMIDE	2;7-NAPHTHALENE DISULFONIC ACID;4-AMINO-5-HYDROXY-;P-TOLUENE SULFONATE (ESTER)	COLCHICINE
Structure	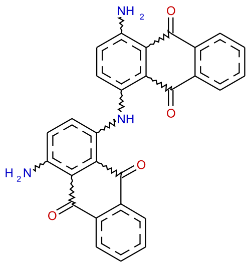	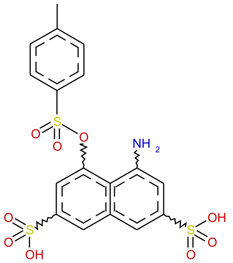	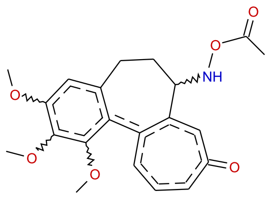
Actual Endpoint	Irritant	Irritant	Irritant
Predicted Endpoint	Irritant	Irritant	Irritant
Distance	0.770	0.851	0.861
Reference	28ZPAK-;125;72	28ZPAK-;194;72	AJOPAA 31;837;48

Model Prediction

Prediction: Irritant
Probability: 1.000
Enrichment: 1.176
Bayesian Score: 2.389
Mahalanobis Distance: 8.547
Mahalanobis Distance p-value: 0.745

Prediction: Positive if the Bayesian score is above the estimated best cutoff value from minimizing the false positive and false negative rate.
Probability: The esimated probability that the sample is in the positive category. This assumes that the Bayesian score follows a normal distribution and is different from the prediction using a cutoff.
Enrichment: An estimate of enrichment, that is, the increased likelihood (versus random) of this sample being in the category.
Bayesian Score: The standard Laplacian-modified Bayesian score.
Mahalanobis Distance: The Mahalanobis distance (MD) is the distance to the center of the training data. The larger the MD, the less trustworthy the prediction.
Mahalanobis Distance p-value: The p-value gives the fraction of training data with an MD greater than or equal to the one for the given sample, assuming normally distributed data. The smaller the p-value, the less trustworthy the prediciton. For highly non-normal X properties (e.g., fingerprints), the MD p-value is wildly inaccurate.

Model Applicability

Unknown features are fingerprint features in the query molecule, but not found in the training set.

1. All properties and OPS components are within expected ranges.
2. Unknown FCFP_2 feature: -928857652: [*]:[c](:[*])C(C)[c](:[*]):[*]
3. Unknown FCFP_2 feature: -828984032: [*][c](:[*]):[c]([N+](=[*])[*]):c:[*]
4. Unknown FCFP_2 feature: -1338588315: [*]:[c](:[*])[N+](=O)[O-]
5. Unknown FCFP_2 feature: 1872392852: [*][N+](=O)[*]
6. Unknown FCFP_2 feature: 260476081: [*][N+](=[*])[O-]

Feature Contribution

Top features for positive contribution				
**Fingerprint**	**Bit/Smiles**	**Feature Structure**	**Score**	**Irritant in training set**

FCFP_12	1747237384	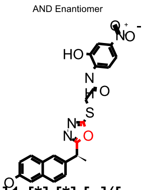 [*][c]1:[*]:[*]:[c]([*]):o:1	0.208	44 out of 44
FCFP_12	1175665944	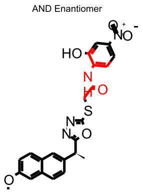 [*]CC(=O)N[c](:[cH]:[*]):[cH]:[*]	0.198	14 out of 14
FCFP_12	17	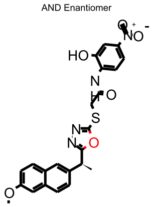 [*]:o:[*]	0.189	48 out of 49

Top Features for negative contribution

**Fingerprint**	**Bit/Smiles**	**Feature Structure**	**Score**	**Irritant in training set**
FCFP_12	8	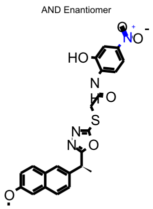 [*][N+](=[*])[*]	-0.056	3 out of 4
FCFP_12	307419094	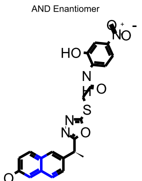 [*]:[cH]:[c](:[cH]:[*]):[c](:[*]):[*]	0.000	43 out of 52

FCFP_12	-1272768868	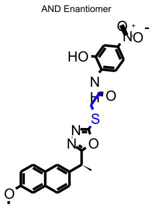<br>[*]SCC(=[*])[*]	0.000	396 out of 514

Sorafenib

TOPKAT_Ocular_Irritancy_None_vs_Irritant

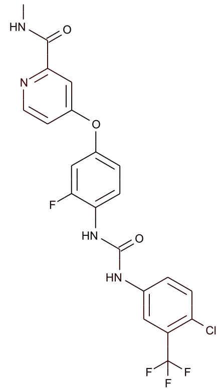

$C_{21}H_{15}ClF_4N_4O_3$
Molecular Weight: 482.81541
ALogP: 4.381
Rotatable Bonds: 6
Acceptors: 4
Donors: 3

Structural Similar Compounds

Name	BENZANILIDE;2';2'''-DITHIOBIS-	4;4'-DIAMINO-1;1'-DIANTHRIMIDE	ANTHRAQUINONE; 1;4-BIS(p-TOLYLAMINO)-
Structure	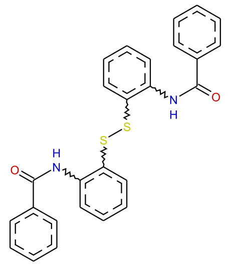	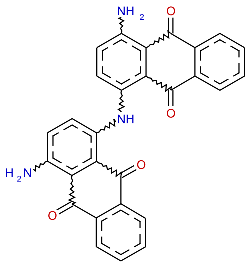	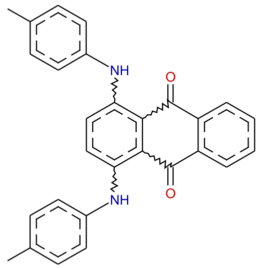
Actual Endpoint	Non-Irritant	Irritant	Irritant
Predicted Endpoint	Non-Irritant	Irritant	Non-Irritant
Distance	0.739	0.800	0.820
Reference	28ZPAK-;173;72	28ZPAK-;125;72	28ZPAK -;124;72

Model Applicability

Unknown features are fingerprint features in the query molecule, but not found in the training set.

1. All properties and OPS components are within expected ranges.

Model Prediction

Prediction: Irritant
Probability: 1.000
Enrichment: 1.176
Bayesian Score: 3.330
Mahalanobis Distance: 6.511
Mahalanobis Distance p-value: 1

Prediction: Positive if the Bayesian score is above the estimated best cutoff value from minimizing the false positive and false negative rate.
Probability: The esimated probability that the sample is in the positive category. This assumes that the Bayesian score follows a normal distribution and is different from the prediction using a cutoff.
Enrichment: An estimate of enrichment, that is, the increased likelihood (versus random) of this sample being in the category.
Bayesian Score: The standard Laplacian-modified Bayesian score.
Mahalanobis Distance: The Mahalanobis distance (MD) is the distance to the center of the training data. The larger the MD, the less trustworthy the prediction.
Mahalanobis Distance p-value: The p-value gives the fraction of training data with an MD greater than or equal to the one for the given sample, assuming normally distributed data. The smaller the p-value, the less trustworthy the prediciton. For highly non-normal X properties (e.g., fingerprints), the MD p-value is wildly inaccurate.

Feature Contribution

Top features for positive contribution

Fingerprint	Bit/Smiles	Feature Structure	Score	Irritant in training set
FCFP_12	1747237384	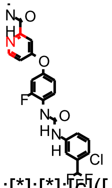 [*][c]1:[*]:[*]:[c]([*]):o:1	0.208	44 out of 44

FCFP_12	-124655670	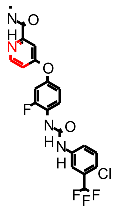 [*]:[cH]:[cH]:n:[*]	0.200	16 out of 16
FCFP_12	-1539132615	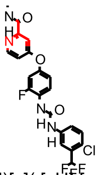 [*]C(=[*])[c](:[cH]:[*]):n:[*]	0.197	13 out of 13

Top Features for negative contribution

Fingerprint	Bit/Smiles	Feature Structure	Score	Irritant in training set
FCFP_12	-747629521	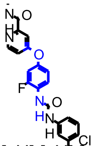 [*]N[c]1:[cH]:[cH]:[c](O[c](:[*]):[*]):[cH]:[c]:1[*]	-0.268	1 out of 2
FCFP_12	-1597477966	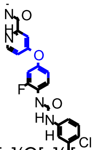 [*]:[cH]:[c](O[c](:[cH]:[*]):[cH]:[*]):[cH]:[*]	0.000	9 out of 11
FCFP_12	-773983804	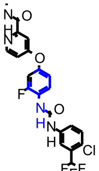 [*]N[c]1:[cH]:[*]:[cH]:[cH]:[cH]:1	0.000	102 out of 121

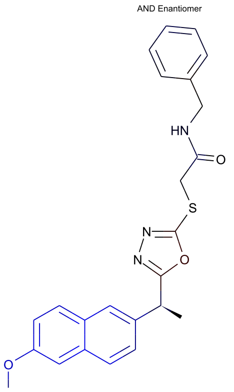

$C_{24}H_{23}N_3O_3S$
Molecular Weight: 433.52272
ALogP: 4.245
Rotatable Bonds: 8
Acceptors: 5
Donors: 1

Model Prediction

Prediction: Non-Carcinogen
Probability: 0.211
Enrichment: 0.654
Bayesian Score: -6.003
Mahalanobis Distance: 13.455
Mahalanobis Distance p-value: 2.39e-005

Prediction: Positive if the Bayesian score is above the estimated best cutoff value from minimizing the false positive and false negative rate.
Probability: The esimated probability that the sample is in the positive category. This assumes that the Bayesian score follows a normal distribution and is different from the prediction using a cutoff.
Enrichment: An estimate of enrichment, that is, the increased likelihood (versus random) of this sample being in the category.
Bayesian Score: The standard Laplacian-modified Bayesian score.
Mahalanobis Distance: The Mahalanobis distance (MD) is the distance to the center of the training data. The larger the MD, the less trustworthy the prediction.
Mahalanobis Distance p-value: The p-value gives the fraction of training data with an MD greater than or equal to the one for the given sample, assuming normally distributed data. The smaller the p-value, the less trustworthy the prediciton. For highly non-normal X properties (e.g., fingerprints), the MD p-value is wildly inaccurate.

Structural Similar Compounds

Name	Lovastatin	Felodipine	Simvastatin
Structure	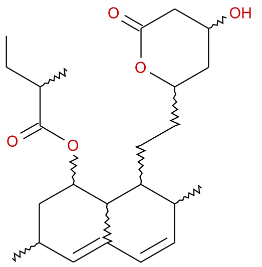	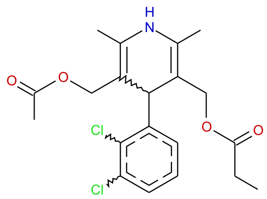	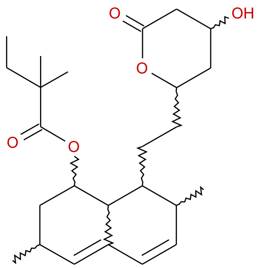
Actual Endpoint	Non-Carcinogen	Non-Carcinogen	Carcinogen
Predicted Endpoint	Carcinogen	Non-Carcinogen	Carcinogen
Distance	0.588	0.593	0.600
Reference	US FDA (Centre for Drug Eval.& Res./Off. Testing & Res.) Sept. 1997	US FDA (Centre for Drug Eval.& Res./Off. Testing & Res.) Sept. 1997	US FDA (Centre for Drug Eval.& Res./Off. Testing & Res.) Sept. 1997

Model Applicability

Unknown features are fingerprint features in the query molecule, but not found in the training set.

1. All properties and OPS components are within expected ranges.
2. Unknown ECFP_2 feature: -955816473: [*]SCC(=[*])[*]
3. Unknown ECFP_2 feature: 1093109320: [*]S[c]1:o:[*]:[*]:n:1
4. Unknown ECFP_2 feature: 1427820655: [*]CS[c](:[*]):[*]
5. Unknown ECFP_2 feature: -1841325949: [*]:[c](:[*])C(C)[c](:[*]):[*]
6. Unknown ECFP_2 feature: 1092541557: [*]C([*])[c]1:o:[*]:[*]:n:1

Feature Contribution

Top features for positive contribution				
**Fingerprint**	**Bit/Smiles**	**Feature Structure**	**Score**	**Carcinogen in training set**
ECFP_12	1203316083	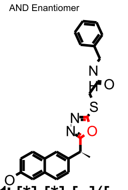 [*][c]1:[*]:[*]:[c]([*]):o:1	0.681	9 out of 13

ECFP_12	110318898	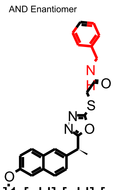 [*]NC[c]1:[cH]:[cH]:[*]:[cH]:[cH]:1	0.421	1 out of 1
ECFP_12	683445015	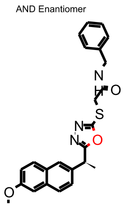 [*]:o:[*]	0.294	28 out of 66
**Top Features for negative contribution**				
**Fingerprint**	**Bit/Smiles**	**Feature Structure**	**Score**	**Carcinogen in training set**
ECFP_12	497523368	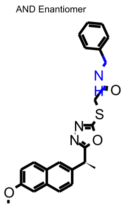 [*]CNC(=[*])[*]	-0.989	1 out of 14
ECFP_12	1571214559	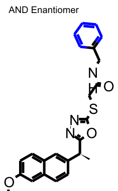 [*]1:[cH]:[cH]:[cH]:[cH]:[cH]:1	-0.560	11 out of 64
ECFP_12	-281505363	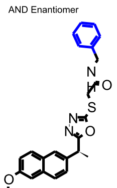 [*][c]1:[cH]:[cH]:[cH]:[cH]:[cH]:1	-0.560	11 out of 64

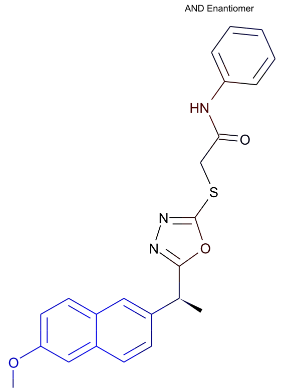

$C_{23}H_{21}N_3O_3S$

Molecular Weight: 419.49614

ALogP: 4.238

Rotatable Bonds: 7

Acceptors: 5

Donors: 1

Structural Similar Compounds

Name	Lovastatin	Simvastatin	Felodipine
Structure	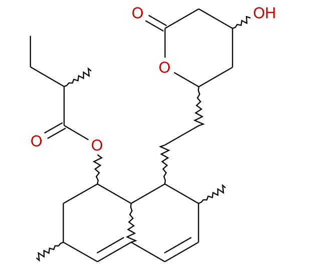	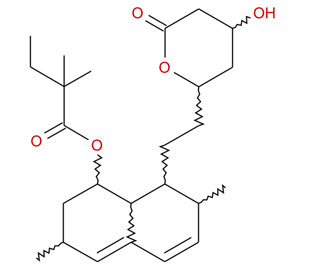	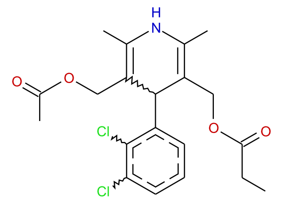
Actual Endpoint	Non-Carcinogen	Carcinogen	Non-Carcinogen
Predicted Endpoint	Carcinogen	Carcinogen	Non-Carcinogen
Distance	0.572	0.593	0.613
Reference	US FDA (Centre for Drug Eval.& Res./Off. Testing & Res.) Sept. 1997	US FDA (Centre for Drug Eval.& Res./Off. Testing & Res.) Sept. 1997	US FDA (Centre for Drug Eval.& Res./Off. Testing & Res.) Sept. 1997

Model Prediction

Prediction: Non-Carcinogen

Probability: 0.223

Enrichment: 0.691

Bayesian Score: -4.874

Mahalanobis Distance: 11.828

Mahalanobis Distance p-value: 0.008

Prediction: Positive if the Bayesian score is above the estimated best cutoff value from minimizing the false positive and false negative rate.
Probability: The esimated probability that the sample is in the positive category. This assumes that the Bayesian score follows a normal distribution and is different from the prediction using a cutoff.
Enrichment: An estimate of enrichment, that is, the increased likelihood (versus random) of this sample being in the category.
Bayesian Score: The standard Laplacian-modified Bayesian score.
Mahalanobis Distance: The Mahalanobis distance (MD) is the distance to the center of the training data. The larger the MD, the less trustworthy the prediction.
Mahalanobis Distance p-value: The p-value gives the fraction of training data with an MD greater than or equal to the one for the given sample, assuming normally distributed data. The smaller the p-value, the less trustworthy the prediciton. For highly non-normal X properties (e.g., fingerprints), the MD p-value is wildly inaccurate.

Model Applicability

Unknown features are fingerprint features in the query molecule, but not found in the training set.

1. All properties and OPS components are within expected ranges.
2. Unknown ECFP_2 feature: -955816473: [*]SCC(=[*])[*]
3. Unknown ECFP_2 feature: 1093109320: [*]S[c]1:o:[*]:[*]:n:1
4. Unknown ECFP_2 feature: 1427820655: [*]CS[c](:[*]):[*]
5. Unknown ECFP_2 feature: -1841325949: [*]:[c](:[*])C(C)[c](:[*]):[*]
6. Unknown ECFP_2 feature: 1092541557: [*]C([*])[c]1:o:[*]:[*]:n:1

Feature Contribution

Top features for positive contribution				
Fingerprint	Bit/Smiles	Feature Structure	Score	Carcinogen in training set
ECFP_12	1203316083	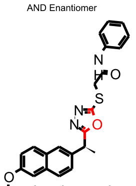 [*][c]1:[*]:[*]:[c]([*]):o:1	0.681	9 out of 13

ECFP_12	-177077903	AND Enantiomer 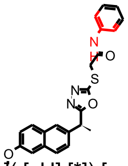 [*]N[c](:[cH]:[*]):[cH]:[*]	0.529	6 out of 10
ECFP_12	-1236483485	AND Enantiomer 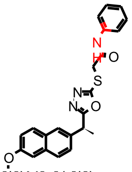 [*]C(=[*])N[c](:[*]):[*]	0.460	9 out of 17

Top Features for negative contribution

Fingerprint	Bit/Smiles	Feature Structure	Score	Carcinogen in training set
ECFP_12	1571214559	AND Enantiomer 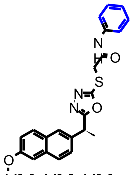 [*]1:[cH]:[cH]:[cH]:[cH]:[cH]:1	-0.560	11 out of 64
ECFP_12	-281505363	AND Enantiomer 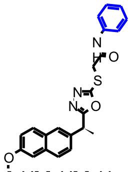 [*][c]1:[cH]:[cH]:[cH]:[cH]:[cH]:1	-0.560	11 out of 64
ECFP_12	168590984	AND Enantiomer 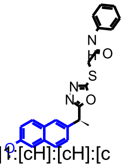 [*]O[c]1:[cH]:[cH]:[c]2:[cH]:[c]([*]):[cH]:[cH]:[c]:2:[cH]:1	-0.485	0 out of 2

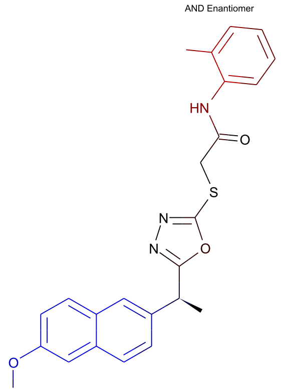

$C_{24}H_{23}N_3O_3S$
Molecular Weight: 433.52272
ALogP: 4.724
Rotatable Bonds: 7
Acceptors: 5
Donors: 1

Structural Similar Compounds

Name	Simvastatin	Lovastatin	Felodipine
Structure	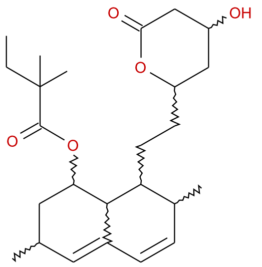	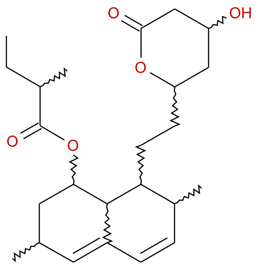	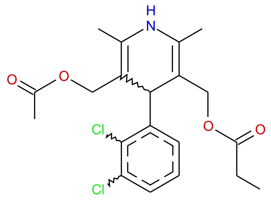
Actual Endpoint	Carcinogen	Non-Carcinogen	Non-Carcinogen
Predicted Endpoint	Carcinogen	Carcinogen	Non-Carcinogen
Distance	0.576	0.580	0.627
Reference	US FDA (Centre for Drug Eval.& Res./Off. Testing & Res.) Sept. 1997	US FDA (Centre for Drug Eval.& Res./Off. Testing & Res.) Sept. 1997	US FDA (Centre for Drug Eval.& Res./Off. Testing & Res.) Sept. 1997

Model Prediction

Prediction: Carcinogen
Probability: 0.271
Enrichment: 0.843
Bayesian Score: -1.444
Mahalanobis Distance: 11.665
Mahalanobis Distance p-value: 0.0128

Prediction: Positive if the Bayesian score is above the estimated best cutoff value from minimizing the false positive and false negative rate.
Probability: The esimated probability that the sample is in the positive category. This assumes that the Bayesian score follows a normal distribution and is different from the prediction using a cutoff.
Enrichment: An estimate of enrichment, that is, the increased likelihood (versus random) of this sample being in the category.
Bayesian Score: The standard Laplacian-modified Bayesian score.
Mahalanobis Distance: The Mahalanobis distance (MD) is the distance to the center of the training data. The larger the MD, the less trustworthy the prediction.
Mahalanobis Distance p-value: The p-value gives the fraction of training data with an MD greater than or equal to the one for the given sample, assuming normally distributed data. The smaller the p-value, the less trustworthy the prediciton. For highly non-normal X properties (e.g., fingerprints), the MD p-value is wildly inaccurate.

Model Applicability

Unknown features are fingerprint features in the query molecule, but not found in the training set.

1. All properties and OPS components are within expected ranges.
2. Unknown ECFP_2 feature: -955816473: [*]SCC(=[*])[*]
3. Unknown ECFP_2 feature: 1093109320: [*]S[c]1:o:[*]:[*]:n:1
4. Unknown ECFP_2 feature: 1427820655: [*]CS[c](:[*]):[*]
5. Unknown ECFP_2 feature: -1841325949: [*]:[c](:[*])C(C)[c](:[*]):[*]
6. Unknown ECFP_2 feature: 1092541557: [*]C([*])[c]1:o:[*]:[*]:n:1

Feature Contribution

Top features for positive contribution

Fingerprint	Bit/Smiles	Feature Structure	Score	Carcinogen in training set
ECFP_12	1203316083	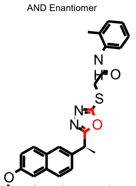 [*][c]1:[*]:[*]:[c]([*]):o:1	0.681	9 out of 13

ECFP_12	-1236483485	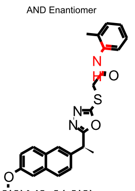<br>[*]C(=[*])N[c](:[*]):[*]	0.460	9 out of 17
ECFP_12	-1516972643	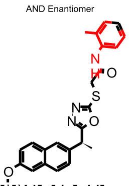<br>[*]C(=[*])N[c]1:[cH]:[cH]:[*]:[cH]:[c]:1C	0.421	1 out of 1
**Top Features for negative contribution**				
**Fingerprint**	**Bit/Smiles**	**Feature Structure**	**Score**	**Carcinogen in training set**
ECFP_12	1153577237	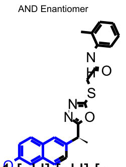<br>[*]O[c]1:[cH]:[cH]:[c]2:[cH]:[c]([*]):[*]:[cH]:[c]:2:[cH]:1	-0.485	0 out of 2
ECFP_12	168590984	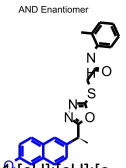<br>[*]O[c]1:[cH]:[cH]:[c]2:[cH]:[c]([*]):[cH]:[cH]:[c]:2:[cH]:1	-0.485	0 out of 2
ECFP_12	-1163815636	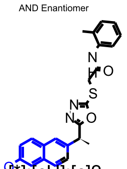<br>[*][c]1:[*]:[cH]:[c]2:[cH]:[c](OC):[cH]:[cH]:[c]:2:[cH]:1	-0.485	0 out of 2

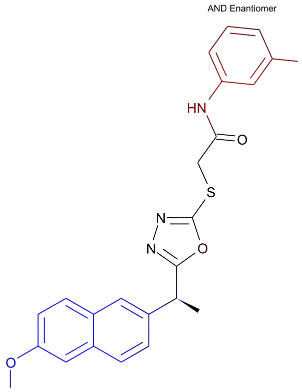

$C_{24}H_{23}N_3O_3S$
Molecular Weight: 433.52272
ALogP: 4.724
Rotatable Bonds: 7
Acceptors: 5
Donors: 1

Structural Similar Compounds

Name	Simvastatin	Lovastatin	Felodipine
Structure	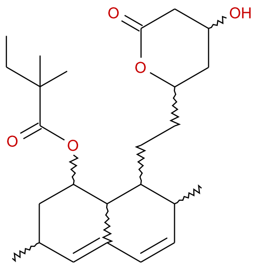	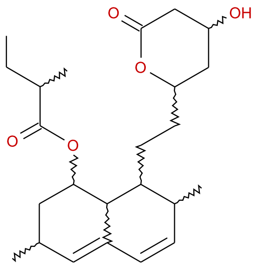	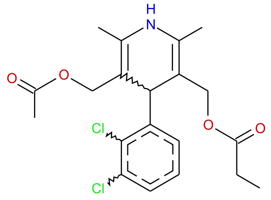
Actual Endpoint	Carcinogen	Non-Carcinogen	Non-Carcinogen
Predicted Endpoint	Carcinogen	Carcinogen	Non-Carcinogen
Distance	0.576	0.580	0.624
Reference	US FDA (Centre for Drug Eval.& Res./Off. Testing & Res.) Sept. 1997	US FDA (Centre for Drug Eval.& Res./Off. Testing & Res.) Sept. 1997	US FDA (Centre for Drug Eval.& Res./Off. Testing & Res.) Sept. 1997

Model Prediction

Prediction: Non-Carcinogen
Probability: 0.262
Enrichment: 0.815
Bayesian Score: -1.988
Mahalanobis Distance: 12.020
Mahalanobis Distance p-value: 0.00448

Prediction: Positive if the Bayesian score is above the estimated best cutoff value from minimizing the false positive and false negative rate.
Probability: The esimated probability that the sample is in the positive category. This assumes that the Bayesian score follows a normal distribution and is different from the prediction using a cutoff.
Enrichment: An estimate of enrichment, that is, the increased likelihood (versus random) of this sample being in the category.
Bayesian Score: The standard Laplacian-modified Bayesian score.
Mahalanobis Distance: The Mahalanobis distance (MD) is the distance to the center of the training data. The larger the MD, the less trustworthy the prediction.
Mahalanobis Distance p-value: The p-value gives the fraction of training data with an MD greater than or equal to the one for the given sample, assuming normally distributed data. The smaller the p-value, the less trustworthy the prediciton. For highly non-normal X properties (e.g., fingerprints), the MD p-value is wildly inaccurate.

Model Applicability

Unknown features are fingerprint features in the query molecule, but not found in the training set.

1. All properties and OPS components are within expected ranges.
2. Unknown ECFP_2 feature: -955816473: [*]SCC(=[*])[*]
3. Unknown ECFP_2 feature: 1093109320: [*]S[c]1:o:[*]:[*]:n:1
4. Unknown ECFP_2 feature: 1427820655: [*]CS[c](:[*]):[*]
5. Unknown ECFP_2 feature: -1841325949: [*]:[c](:[*])C(C)[c](:[*]):[*]
6. Unknown ECFP_2 feature: 1092541557: [*]C([*])[c]1:o:[*]:[*]:n:1

Feature Contribution

Top features for positive contribution

Fingerprint	Bit/Smiles	Feature Structure	Score	Carcinogen in training set
ECFP_12	1203316083	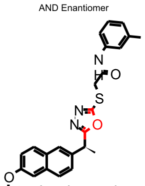 [*][c]1:[*]:[*]:[c]([*]):o:1	0.681	9 out of 13

ECFP_12	-177077903	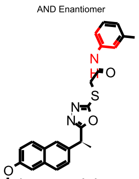 [*]N[c](:[cH]:[*]):[cH]:[*]	0.529	6 out of 10
ECFP_12	-1236483485	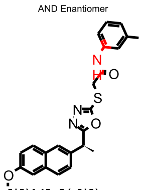 [*]C(=[*])N[c](:[*]):[*]	0.460	9 out of 17

Top Features for negative contribution

Fingerprint	Bit/Smiles	Feature Structure	Score	Carcinogen in training set
ECFP_12	-1145977934	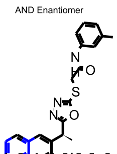 [*]O[c]1:[cH]:[*]:[c](:[*]):[c](:[cH]:[*]):[cH]:1	-0.485	0 out of 2
ECFP_12	2082242578	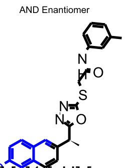 [*]:[cH]:[c]1:[cH]:[cH]:[c](OC):[cH]:[c]:1:[*]	-0.485	0 out of 2
ECFP_12	-1731425419	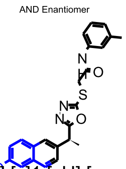 [*]:[cH]:[c]1:[cH]:[c](OC):[cH]:[cH]:[c]:1:[*]	-0.485	0 out of 2

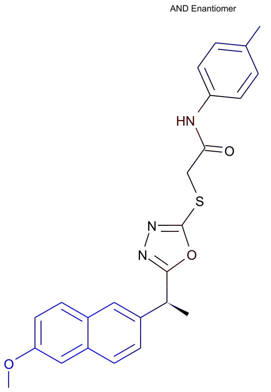

$C_{24}H_{23}N_3O_3S$
Molecular Weight: 433.52272
ALogP: 4.724
Rotatable Bonds: 7
Acceptors: 5
Donors: 1

Model Prediction

Prediction: Non-Carcinogen
Probability: 0.210
Enrichment: 0.653
Bayesian Score: -6.035
Mahalanobis Distance: 11.012
Mahalanobis Distance p-value: 0.0651

Prediction: Positive if the Bayesian score is above the estimated best cutoff value from minimizing the false positive and false negative rate.
Probability: The esimated probability that the sample is in the positive category. This assumes that the Bayesian score follows a normal distribution and is different from the prediction using a cutoff.
Enrichment: An estimate of enrichment, that is, the increased likelihood (versus random) of this sample being in the category.
Bayesian Score: The standard Laplacian-modified Bayesian score.
Mahalanobis Distance: The Mahalanobis distance (MD) is the distance to the center of the training data. The larger the MD, the less trustworthy the prediction.
Mahalanobis Distance p-value: The p-value gives the fraction of training data with an MD greater than or equal to the one for the given sample, assuming normally distributed data. The smaller the p-value, the less trustworthy the prediciton. For highly non-normal X properties (e.g., fingerprints), the MD p-value is wildly inaccurate.

Structural Similar Compounds

Name	Simvastatin	Lovastatin	Felodipine
Structure	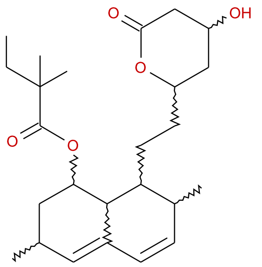	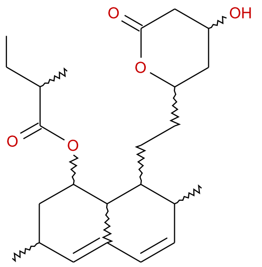	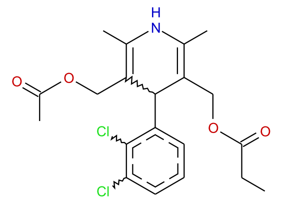
Actual Endpoint	Carcinogen	Non-Carcinogen	Non-Carcinogen
Predicted Endpoint	Carcinogen	Carcinogen	Non-Carcinogen
Distance	0.576	0.580	0.630
Reference	US FDA (Centre for Drug Eval.& Res./Off. Testing & Res.) Sept. 1997	US FDA (Centre for Drug Eval.& Res./Off. Testing & Res.) Sept. 1997	US FDA (Centre for Drug Eval.& Res./Off. Testing & Res.) Sept. 1997

Model Applicability

Unknown features are fingerprint features in the query molecule, but not found in the training set.

1. All properties and OPS components are within expected ranges.
2. Unknown ECFP_2 feature: -955816473: [*]SCC(=[*])[*]
3. Unknown ECFP_2 feature: 1093109320: [*]S[c]1:o:[*]:[*]:n:1
4. Unknown ECFP_2 feature: 1427820655: [*]CS[c](:[*]):[*]
5. Unknown ECFP_2 feature: -1841325949: [*]:[c](:[*])C(C)[c](:[*]):[*]
6. Unknown ECFP_2 feature: 1092541557: [*]C([*])[c]1:o:[*]:[*]:n:1

Feature Contribution

Top features for positive contribution

Fingerprint	Bit/Smiles	Feature Structure	Score	Carcinogen in training set
ECFP_12	1203316083	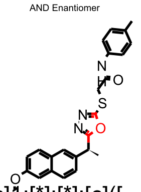 [*][c]1:[*]:[*]:[c]([*]):o:1	0.681	9 out of 13

ECFP_12	-177077903	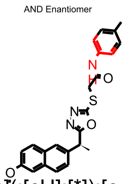<br>[*]N[c](:[cH]:[*]):[cH]:[*]	0.529	6 out of 10
ECFP_12	-1236483485	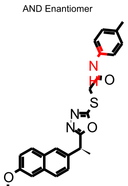<br>[*]C(=[*])N[c](:[*]):[*]	0.460	9 out of 17
**Top Features for negative contribution**				
**Fingerprint**	**Bit/Smiles**	**Feature Structure**	**Score**	**Carcinogen in training set**
ECFP_12	-533780882	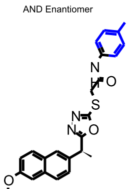<br>C[c]1:[cH]:[cH]:[*]:[cH]:[cH]:1	-1.056	0 out of 6
ECFP_12	-1926229349	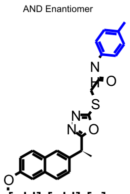<br>[*][c]1:[cH]:[cH]:[c](C):[cH]:[cH]:1	-1.056	0 out of 6
ECFP_12	-210573707	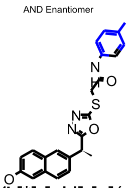<br>[*][c]1:[*]:[cH]:[c](C):[cH]:[cH]:1	-0.560	1 out of 8

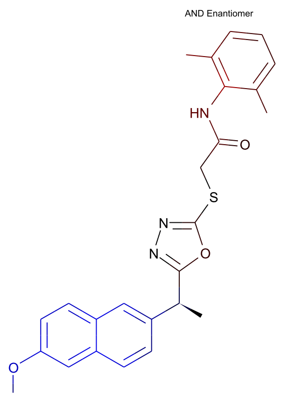

$C_{25}H_{25}N_3O_3S$
Molecular Weight: 447.5493
ALogP: 5.21
Rotatable Bonds: 7
Acceptors: 5
Donors: 1

Model Prediction

Prediction: Non-Carcinogen
Probability: 0.264
Enrichment: 0.819
Bayesian Score: -1.900
Mahalanobis Distance: 12.014
Mahalanobis Distance p-value: 0.00456

Prediction: Positive if the Bayesian score is above the estimated best cutoff value from minimizing the false positive and false negative rate.
Probability: The esimated probability that the sample is in the positive category. This assumes that the Bayesian score follows a normal distribution and is different from the prediction using a cutoff.
Enrichment: An estimate of enrichment, that is, the increased likelihood (versus random) of this sample being in the category.
Bayesian Score: The standard Laplacian-modified Bayesian score.
Mahalanobis Distance: The Mahalanobis distance (MD) is the distance to the center of the training data. The larger the MD, the less trustworthy the prediction.
Mahalanobis Distance p-value: The p-value gives the fraction of training data with an MD greater than or equal to the one for the given sample, assuming normally distributed data. The smaller the p-value, the less trustworthy the prediciton. For highly non-normal X properties (e.g., fingerprints), the MD p-value is wildly inaccurate.

Structural Similar Compounds

Name	Simvastatin	Lovastatin	Felodipine
Structure	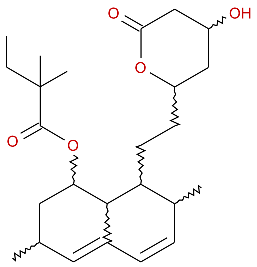	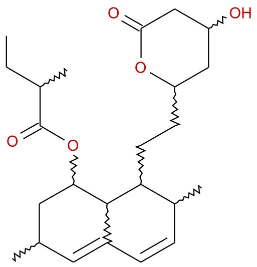	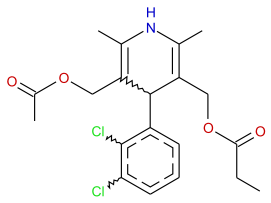
Actual Endpoint	Carcinogen	Non-Carcinogen	Non-Carcinogen
Predicted Endpoint	Carcinogen	Carcinogen	Non-Carcinogen
Distance	0.588	0.612	0.656
Reference	US FDA (Centre for Drug Eval.& Res./Off. Testing & Res.) Sept. 1997	US FDA (Centre for Drug Eval.& Res./Off. Testing & Res.) Sept. 1997	US FDA (Centre for Drug Eval.& Res./Off. Testing & Res.) Sept. 1997

Model Applicability

Unknown features are fingerprint features in the query molecule, but not found in the training set.

1. All properties and OPS components are within expected ranges.
2. Unknown ECFP_2 feature: -955816473: [*]SCC(=[*])[*]
3. Unknown ECFP_2 feature: 1093109320: [*]S[c]1:o:[*]:[*]:n:1
4. Unknown ECFP_2 feature: 1427820655: [*]CS[c](:[*]):[*]
5. Unknown ECFP_2 feature: -1841325949: [*]:[c](:[*])C(C)[c](:[*]):[*]
6. Unknown ECFP_2 feature: 1092541557: [*]C([*])[c]1:o:[*]:[*]:n:1

Feature Contribution

Top features for positive contribution				
**Fingerprint**	**Bit/Smiles**	**Feature Structure**	**Score**	**Carcinogen in training set**
ECFP_12	1203316083	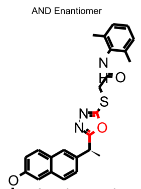 [*][c]1:[*]:[*]:[*]:[c]([*]):o:1	0.681	9 out of 13

ECFP_12	-1236483485	AND Enantiomer 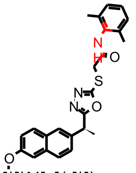 [*]C(=[*])N[c](:[*]):[*]	0.460	9 out of 17
ECFP_12	574399351	AND Enantiomer 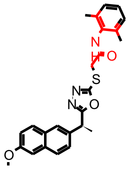 [*]CC(=O)N[c](:[c]([*]):[*]):[c]([*]):[*]	0.421	1 out of 1
**Top Features for negative contribution**				
**Fingerprint**	**Bit/Smiles**	**Feature Structure**	**Score**	**Carcinogen in training set**
ECFP_12	1264202888	AND Enantiomer 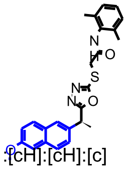 [*][c]1:[cH]:[cH]:[c]2:[cH]:[c](OC):[cH]:[cH]:[c]:2:[cH]:1	-0.485	0 out of 2
ECFP_12	-1163815636	AND Enantiomer 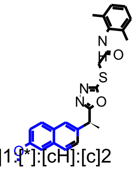 [*][c]1:[*]:[cH]:[c]2:[cH]:[c](OC):[cH]:[cH]:[c]:2:[cH]:1	-0.485	0 out of 2
ECFP_12	1153577237	AND Enantiomer 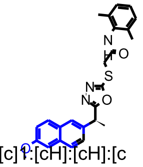 [*]O[c]1:[cH]:[cH]:[c]2:[cH]:[c]([*]):[*]:[cH]:[c]:2:[cH]:1	-0.485	0 out of 2

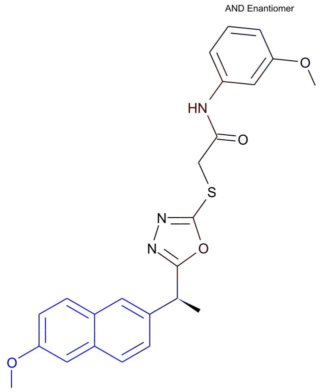

$C_{24}H_{23}N_3O_4S$
Molecular Weight: 449.52212
ALogP: 4.221
Rotatable Bonds: 8
Acceptors: 6
Donors: 1

Model Prediction

Prediction: Non-Carcinogen
Probability: 0.229
Enrichment: 0.711
Bayesian Score: -4.347
Mahalanobis Distance: 11.984
Mahalanobis Distance p-value: 0.00501

Prediction: Positive if the Bayesian score is above the estimated best cutoff value from minimizing the false positive and false negative rate.
Probability: The esimated probability that the sample is in the positive category. This assumes that the Bayesian score follows a normal distribution and is different from the prediction using a cutoff.
Enrichment: An estimate of enrichment, that is, the increased likelihood (versus random) of this sample being in the category.
Bayesian Score: The standard Laplacian-modified Bayesian score.
Mahalanobis Distance: The Mahalanobis distance (MD) is the distance to the center of the training data. The larger the MD, the less trustworthy the prediction.
Mahalanobis Distance p-value: The p-value gives the fraction of training data with an MD greater than or equal to the one for the given sample, assuming normally distributed data. The smaller the p-value, the less trustworthy the prediciton. For highly non-normal X properties (e.g., fingerprints), the MD p-value is wildly inaccurate.

Structural Similar Compounds

Name	Moricizine	Felodipine	Lovastatin
Structure	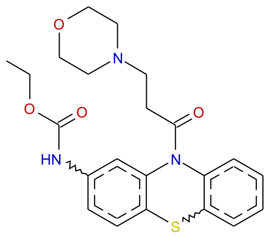	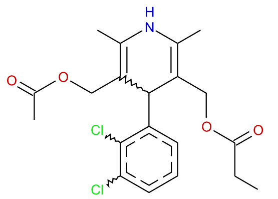	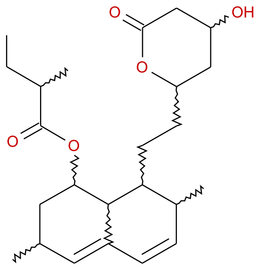
Actual Endpoint	Carcinogen	Non-Carcinogen	Non-Carcinogen
Predicted Endpoint	Carcinogen	Non-Carcinogen	Carcinogen
Distance	0.634	0.639	0.639
Reference	US FDA (Centre for Drug Eval.& Res./Off. Testing & Res.) Sept. 1997	US FDA (Centre for Drug Eval.& Res./Off. Testing & Res.) Sept. 1997	US FDA (Centre for Drug Eval.& Res./Off. Testing & Res.) Sept. 1997

Model Applicability

Unknown features are fingerprint features in the query molecule, but not found in the training set.

1. All properties and OPS components are within expected ranges.
2. Unknown ECFP_2 feature: -955816473: [*]SCC(=[*])[*]
3. Unknown ECFP_2 feature: 1093109320: [*]S[c]1:o:[*]:[*]:n:1
4. Unknown ECFP_2 feature: 1427820655: [*]CS[c](:[*]):[*]
5. Unknown ECFP_2 feature: -1841325949: [*]:[c](:[*])C(C)[c](:[*]):[*]
6. Unknown ECFP_2 feature: 1092541557: [*]C([*])[c]1:o:[*]:[*]:n:1

Feature Contribution

Top features for positive contribution				
**Fingerprint**	**Bit/Smiles**	**Feature Structure**	**Score**	**Carcinogen in training set**
ECFP_12	1203316083	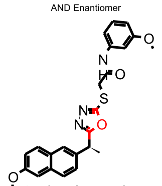 [*][c]1:[*]:[*]:[*]:[c]([*]):o:1	0.681	9 out of 13

ECFP_12	-177077903	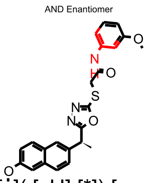 [*]N[c](:[cH]:[*]):[cH]:[*]	0.529	6 out of 10
ECFP_12	-1236483485	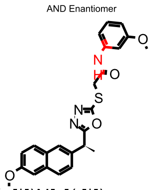 [*]C(=[*])N[c](:[*]):[*]	0.460	9 out of 17
**Top Features for negative contribution**				
**Fingerprint**	**Bit/Smiles**	**Feature Structure**	**Score**	**Carcinogen in training set**
ECFP_12	168590984	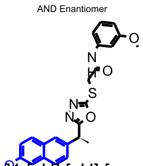 [*]O[c]1:[cH]:[cH]:[c]2:[cH]:[c]([*]):[cH]:[cH]:[c]:2:[cH]:1	-0.485	0 out of 2
ECFP_12	1264202888	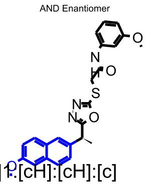 [*][c]1:[cH]:[cH]:[c]2:[cH]:[c](OC):[cH]:[cH]:[c]:2:[cH]:1	-0.485	0 out of 2
ECFP_12	-1145977934	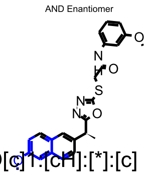 [*]O[c]1:[cH]:[*]:[c](:[*]):[c](:[cH]:[*]):[cH]:1	-0.485	0 out of 2

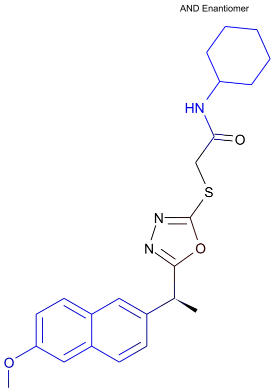

$C_{23}H_{27}N_3O_3S$

Molecular Weight: 425.54378

ALogP: 4.52

Rotatable Bonds: 7

Acceptors: 5

Donors: 1

Model Prediction

Prediction: Non-Carcinogen

Probability: 0.177

Enrichment: 0.551

Bayesian Score: -10.930

Mahalanobis Distance: 11.769

Mahalanobis Distance p-value: 0.00947

Prediction: Positive if the Bayesian score is above the estimated best cutoff value from minimizing the false positive and false negative rate.
Probability: The esimated probability that the sample is in the positive category. This assumes that the Bayesian score follows a normal distribution and is different from the prediction using a cutoff.
Enrichment: An estimate of enrichment, that is, the increased likelihood (versus random) of this sample being in the category.
Bayesian Score: The standard Laplacian-modified Bayesian score.
Mahalanobis Distance: The Mahalanobis distance (MD) is the distance to the center of the training data. The larger the MD, the less trustworthy the prediction.
Mahalanobis Distance p-value: The p-value gives the fraction of training data with an MD greater than or equal to the one for the given sample, assuming normally distributed data. The smaller the p-value, the less trustworthy the prediciton. For highly non-normal X properties (e.g., fingerprints), the MD p-value is wildly inaccurate.

Structural Similar Compounds

Name	Lovastatin	Simvastatin	Felodipine
Structure	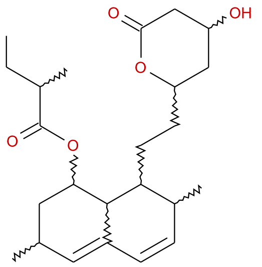	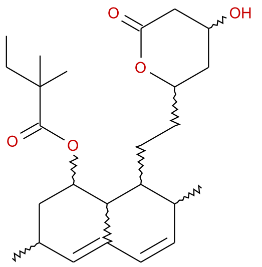	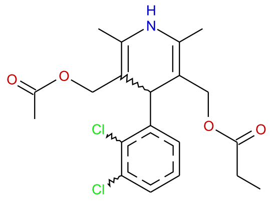
Actual Endpoint	Non-Carcinogen	Carcinogen	Non-Carcinogen
Predicted Endpoint	Carcinogen	Carcinogen	Non-Carcinogen
Distance	0.567	0.575	0.620
Reference	US FDA (Centre for Drug Eval.& Res./Off. Testing & Res.) Sept. 1997	US FDA (Centre for Drug Eval.& Res./Off. Testing & Res.) Sept. 1997	US FDA (Centre for Drug Eval.& Res./Off. Testing & Res.) Sept. 1997

Model Applicability

Unknown features are fingerprint features in the query molecule, but not found in the training set.

1. OPS PC31 out of range. Value: 4.4647. Training min, max, SD, explained variance: -2.785, 3.2518, 0.9521, 0.0095.
2. Unknown ECFP_2 feature: -955816473: [*]SCC(=[*])[*]
3. Unknown ECFP_2 feature: 1093109320: [*]S[c]1:o:[*]:[*]:n:1
4. Unknown ECFP_2 feature: 1427820655: [*]CS[c](:[*]):[*]
5. Unknown ECFP_2 feature: -1841325949: [*]:[c](:[*])C(C)[c](:[*]):[*]
6. Unknown ECFP_2 feature: 1092541557: [*]C([*])[c]1:o:[*]:[*]:n:1

Feature Contribution

Top features for positive contribution				
Fingerprint	Bit/Smiles	Feature Structure	Score	Carcinogen in training set

ECFP_12	1203316083	AND Enantiomer 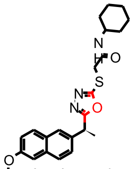 [*][c]1:[*]:[*]:[c]([*]):o:1	0.681	9 out of 13
ECFP_12	683445015	AND Enantiomer 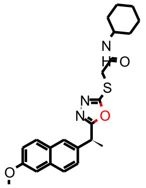 [*]:o:[*]	0.294	28 out of 66
ECFP_12	2055803015	AND Enantiomer 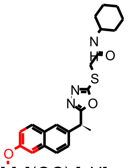 [*]:[cH]:[c](OC):[cH] :[*]	0.264	6 out of 14

Top Features for negative contribution

Fingerprint	Bit/Smiles	Feature Structure	Score	Carcinogen in training set
ECFP_12	-1051657476	AND Enantiomer 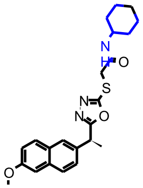 [*]C(=[*])NC1CC[*]CC1	-0.941	0 out of 5
ECFP_12	662850656	AND Enantiomer 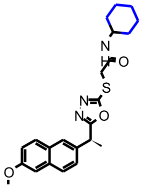 [*]1CCCCC1	-0.929	1 out of 13

ECFP_12	1701046020	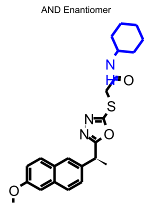<br>[*]C(=[*])NC1CCCCC1	-0.811	0 out of 4

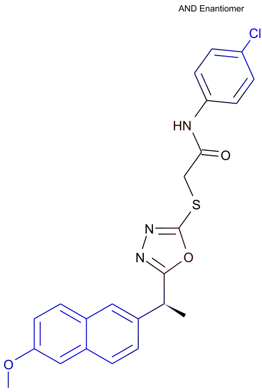

$C_{23}H_{20}ClN_3O_3S$
Molecular Weight: 453.9412
ALogP: 4.902
Rotatable Bonds: 7
Acceptors: 5
Donors: 1

Structural Similar Compounds

Name	Simvastatin	Lovastatin	Felodipine
Structure	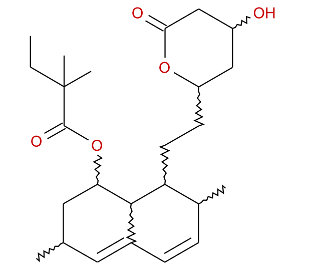	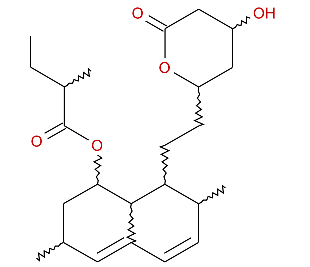	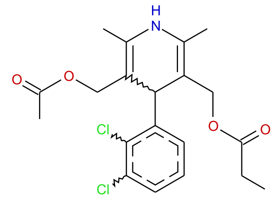
Actual Endpoint	Carcinogen	Non-Carcinogen	Non-Carcinogen
Predicted Endpoint	Carcinogen	Carcinogen	Non-Carcinogen
Distance	0.595	0.609	0.650
Reference	US FDA (Centre for Drug Eval.& Res./Off. Testing & Res.) Sept. 1997	US FDA (Centre for Drug Eval.& Res./Off. Testing & Res.) Sept. 1997	US FDA (Centre for Drug Eval.& Res./Off. Testing & Res.) Sept. 1997

Model Prediction

Prediction: Non-Carcinogen
Probability: 0.193
Enrichment: 0.600
Bayesian Score: -8.055
Mahalanobis Distance: 10.955
Mahalanobis Distance p-value: 0.0737

Prediction: Positive if the Bayesian score is above the estimated best cutoff value from minimizing the false positive and false negative rate.
Probability: The esimated probability that the sample is in the positive category. This assumes that the Bayesian score follows a normal distribution and is different from the prediction using a cutoff.
Enrichment: An estimate of enrichment, that is, the increased likelihood (versus random) of this sample being in the category.
Bayesian Score: The standard Laplacian-modified Bayesian score.
Mahalanobis Distance: The Mahalanobis distance (MD) is the distance to the center of the training data. The larger the MD, the less trustworthy the prediction.
Mahalanobis Distance p-value: The p-value gives the fraction of training data with an MD greater than or equal to the one for the given sample, assuming normally distributed data. The smaller the p-value, the less trustworthy the prediciton. For highly non-normal X properties (e.g., fingerprints), the MD p-value is wildly inaccurate.

Model Applicability

Unknown features are fingerprint features in the query molecule, but not found in the training set.

1. All properties and OPS components are within expected ranges.
2. Unknown ECFP_2 feature: -955816473: [*]SCC(=[*])[*]
3. Unknown ECFP_2 feature: 1093109320: [*]S[c]1:o:[*]:[*]:n:1
4. Unknown ECFP_2 feature: 1427820655: [*]CS[c](:[*]):[*]
5. Unknown ECFP_2 feature: -1841325949: [*]:[c](:[*])C(C)[c](:[*]):[*]
6. Unknown ECFP_2 feature: 1092541557: [*]C([*])[c]1:o:[*]:[*]:n:1

Feature Contribution

Top features for positive contribution				
**Fingerprint**	**Bit/Smiles**	**Feature Structure**	**Score**	**Carcinogen in training set**
ECFP_12	1203316083	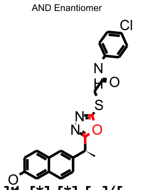 [*][c]1:[*]:[*]:[c]([*]):o:1	0.681	9 out of 13

ECFP_12	-177077903	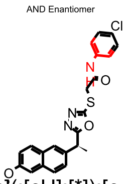 [*]N[c](:[cH]:[*]):[cH]:[*]	0.529	6 out of 10
ECFP_12	-1236483485	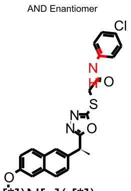 [*]C(=[*])N[c](:[*]):[*]	0.460	9 out of 17
**Top Features for negative contribution**				
**Fingerprint**	**Bit/Smiles**	**Feature Structure**	**Score**	**Carcinogen in training set**
ECFP_12	99947387	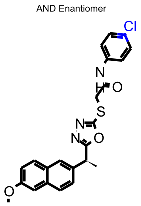 [*]:[c](:[*])Cl	-0.817	8 out of 62
ECFP_12	1854732111	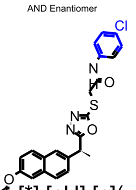 [*][c]1:[*]:[cH]:[c](Cl):[cH]:[cH]:1	-0.816	4 out of 33
ECFP_12	-769655848	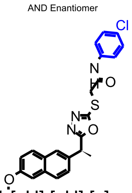 [*][c]1:[cH]:[cH]:[c](Cl):[cH]:[cH]:1	-0.797	1 out of 11

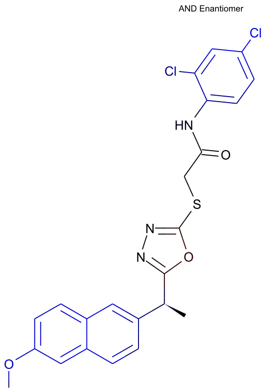

$C_{23}H_{19}Cl_2N_3O_3S$
Molecular Weight: 488.38626
ALogP: 5.567
Rotatable Bonds: 7
Acceptors: 5
Donors: 1

Structural Similar Compounds

Name	Simvastatin	Emetine	Lovastatin
Structure	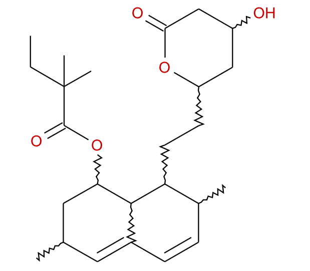	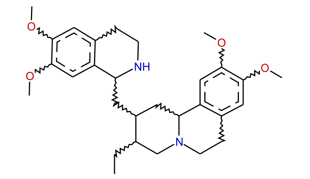	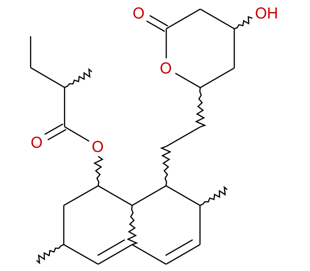
Actual Endpoint	Carcinogen	Non-Carcinogen	Non-Carcinogen
Predicted Endpoint	Carcinogen	Non-Carcinogen	Carcinogen
Distance	0.651	0.670	0.681
Reference	US FDA (Centre for Drug Eval.& Res./Off. Testing & Res.) Sept. 1997	US FDA (Centre for Drug Eval.& Res./Off. Testing & Res.) Sept. 1997	US FDA (Centre for Drug Eval.& Res./Off. Testing & Res.) Sept. 1997

Model Prediction

Prediction: Non-Carcinogen
Probability: 0.183
Enrichment: 0.567
Bayesian Score: -9.815
Mahalanobis Distance: 11.088
Mahalanobis Distance p-value: 0.0549

Prediction: Positive if the Bayesian score is above the estimated best cutoff value from minimizing the false positive and false negative rate.
Probability: The esimated probability that the sample is in the positive category. This assumes that the Bayesian score follows a normal distribution and is different from the prediction using a cutoff.
Enrichment: An estimate of enrichment, that is, the increased likelihood (versus random) of this sample being in the category.
Bayesian Score: The standard Laplacian-modified Bayesian score.
Mahalanobis Distance: The Mahalanobis distance (MD) is the distance to the center of the training data. The larger the MD, the less trustworthy the prediction.
Mahalanobis Distance p-value: The p-value gives the fraction of training data with an MD greater than or equal to the one for the given sample, assuming normally distributed data. The smaller the p-value, the less trustworthy the prediciton. For highly non-normal X properties (e.g., fingerprints), the MD p-value is wildly inaccurate.

Model Applicability

Unknown features are fingerprint features in the query molecule, but not found in the training set.

1. All properties and OPS components are within expected ranges.
2. Unknown ECFP_2 feature: -955816473: [*]SCC(=[*])[*]
3. Unknown ECFP_2 feature: 1093109320: [*]S[c]1:o:[*]:[*]:n:1
4. Unknown ECFP_2 feature: 1427820655: [*]CS[c](:[*]):[*]
5. Unknown ECFP_2 feature: -1841325949: [*]:[c](:[*])C(C)[c](:[*]):[*]
6. Unknown ECFP_2 feature: 1092541557: [*]C([*])[c]1:o:[*]:[*]:n:1

Feature Contribution

Top features for positive contribution

Fingerprint	Bit/Smiles	Feature Structure	Score	Carcinogen in training set
ECFP_12	1203316083	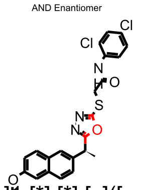 [*][c]1:[*]:[*]:[c]([*]):o:1	0.681	9 out of 13

ECFP_12	-1236483485	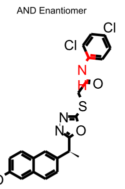 [*]C(=[*])N[c](:[*]):[*]	0.460	9 out of 17
ECFP_12	683445015	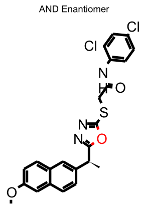 [*]:o:[*]	0.294	28 out of 66

Top Features for negative contribution

Fingerprint	Bit/Smiles	Feature Structure	Score	Carcinogen in training set
ECFP_12	1335691903	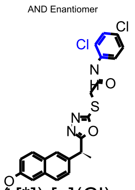 [*][c](:[*]):[c](Cl):[cH]:[*]	-1.112	2 out of 26
ECFP_12	99947387	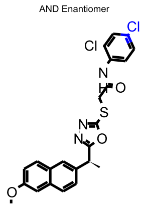 [*]:[c](:[*])Cl	-0.817	8 out of 62
ECFP_12	1854732111	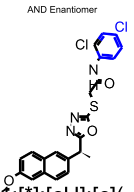 [*][c]1:[*]:[cH]:[c](Cl):[cH]:[cH]:1	-0.816	4 out of 33

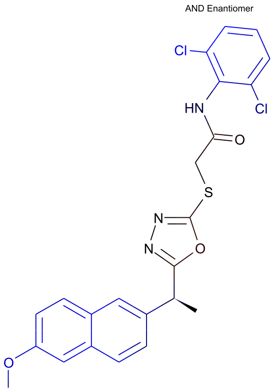

$C_{23}H_{19}Cl_2N_3O_3S$
Molecular Weight: 488.38626
ALogP: 5.567
Rotatable Bonds: 7
Acceptors: 5
Donors: 1

Model Prediction

Prediction: Non-Carcinogen
Probability: 0.181
Enrichment: 0.562
Bayesian Score: -10.102
Mahalanobis Distance: 12.395
Mahalanobis Distance p-value: 0.00133

Prediction: Positive if the Bayesian score is above the estimated best cutoff value from minimizing the false positive and false negative rate.
Probability: The esimated probability that the sample is in the positive category. This assumes that the Bayesian score follows a normal distribution and is different from the prediction using a cutoff.
Enrichment: An estimate of enrichment, that is, the increased likelihood (versus random) of this sample being in the category.
Bayesian Score: The standard Laplacian-modified Bayesian score.
Mahalanobis Distance: The Mahalanobis distance (MD) is the distance to the center of the training data. The larger the MD, the less trustworthy the prediction.
Mahalanobis Distance p-value: The p-value gives the fraction of training data with an MD greater than or equal to the one for the given sample, assuming normally distributed data. The smaller the p-value, the less trustworthy the prediciton. For highly non-normal X properties (e.g., fingerprints), the MD p-value is wildly inaccurate.

Structural Similar Compounds

Name	Simvastatin	Emetine	Astemizole
Structure	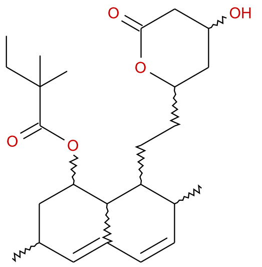	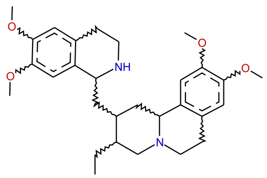	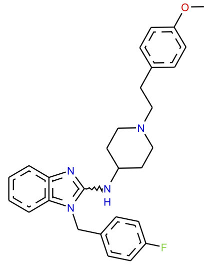
Actual Endpoint	Carcinogen	Non-Carcinogen	Non-Carcinogen
Predicted Endpoint	Carcinogen	Non-Carcinogen	Non-Carcinogen
Distance	0.650	0.670	0.679
Reference	US FDA (Centre for Drug Eval.& Res./Off. Testing & Res.) Sept. 1997	US FDA (Centre for Drug Eval.& Res./Off. Testing & Res.) Sept. 1997	US FDA (Centre for Drug Eval.& Res./Off. Testing & Res.) Sept. 1997

Model Applicability

Unknown features are fingerprint features in the query molecule, but not found in the training set.

1. All properties and OPS components are within expected ranges.
2. Unknown ECFP_2 feature: -955816473: [*]SCC(=[*])[*]
3. Unknown ECFP_2 feature: 1093109320: [*]S[c]1:o:[*]:[*]:n:1
4. Unknown ECFP_2 feature: 1427820655: [*]CS[c](:[*]):[*]
5. Unknown ECFP_2 feature: -1841325949: [*]:[c](:[*])C(C)[c](:[*]):[*]
6. Unknown ECFP_2 feature: 1092541557: [*]C([*])[c]1:o:[*]:[*]:n:1

Feature Contribution

Top features for positive contribution

Fingerprint	Bit/Smiles	Feature Structure	Score	Carcinogen in training set
ECFP_12	1203316083	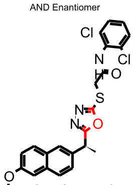 [*][c]1:[*]:[*]:[c]([*]):o:1	0.681	9 out of 13

ECFP_12	-1236483485	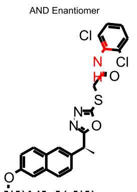 [*]C(=[*])N[c](:[*]):[*]	0.460	9 out of 17
ECFP_12	574399351	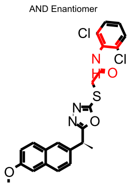 [*]CC(=O)N[c](:[c]([*]):[*]):[c]([*]):[*]	0.421	1 out of 1

Top Features for negative contribution

Fingerprint	Bit/Smiles	Feature Structure	Score	Carcinogen in training set
ECFP_12	1335691903	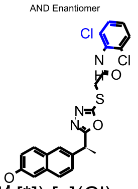 [*][c](:[*]):[c](Cl):[cH]:[*]	-1.112	2 out of 26
ECFP_12	-1354065290	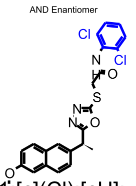 [*][c]1:[c](Cl):[cH]:[cH]:[cH]:[c]:1Cl	-0.941	0 out of 5
ECFP_12	1641317964	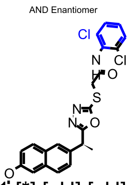 [*][c]1:[*]:[cH]:[cH]:[cH]:[c]:1Cl	-0.929	1 out of 13

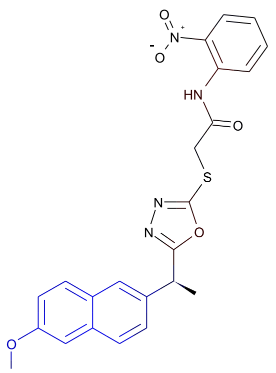

$C_{23}H_{20}N_4O_5S$
Molecular Weight: 464.4937
ALogP: 4.132
Rotatable Bonds: 8
Acceptors: 7
Donors: 1

Structural Similar Compounds

Name	Carbenicillin	Nimodipine	Nicardipine
Structure	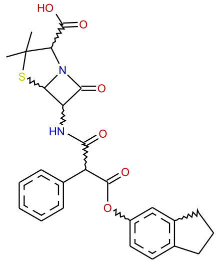	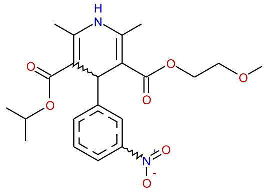	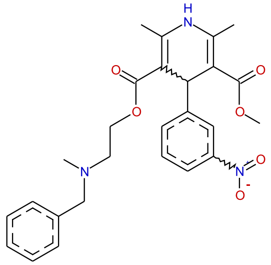
Actual Endpoint	Non-Carcinogen	Non-Carcinogen	Carcinogen
Predicted Endpoint	Non-Carcinogen	Non-Carcinogen	Carcinogen
Distance	0.642	0.657	0.668
Reference	US FDA (Centre for Drug Eval.& Res./Off. Testing & Res.) Sept. 1997	US FDA (Centre for Drug Eval.& Res./Off. Testing & Res.) Sept. 1997	US FDA (Centre for Drug Eval.& Res./Off. Testing & Res.) Sept. 1997

Model Prediction

Prediction: Non-Carcinogen
Probability: 0.233
Enrichment: 0.722
Bayesian Score: -4.048
Mahalanobis Distance: 11.693
Mahalanobis Distance p-value: 0.0118

Prediction: Positive if the Bayesian score is above the estimated best cutoff value from minimizing the false positive and false negative rate.
Probability: The esimated probability that the sample is in the positive category. This assumes that the Bayesian score follows a normal distribution and is different from the prediction using a cutoff.
Enrichment: An estimate of enrichment, that is, the increased likelihood (versus random) of this sample being in the category.
Bayesian Score: The standard Laplacian-modified Bayesian score.
Mahalanobis Distance: The Mahalanobis distance (MD) is the distance to the center of the training data. The larger the MD, the less trustworthy the prediction.
Mahalanobis Distance p-value: The p-value gives the fraction of training data with an MD greater than or equal to the one for the given sample, assuming normally distributed data. The smaller the p-value, the less trustworthy the prediciton. For highly non-normal X properties (e.g., fingerprints), the MD p-value is wildly inaccurate.

Model Applicability

Unknown features are fingerprint features in the query molecule, but not found in the training set.

1. All properties and OPS components are within expected ranges.
2. Unknown ECFP_2 feature: 1043790491: [*][N+](=[*])[*]
3. Unknown ECFP_2 feature: 781519895: [*][O-]
4. Unknown ECFP_2 feature: -955816473: [*]SCC(=[*])[*]
5. Unknown ECFP_2 feature: 1093109320: [*]S[c]1:o:[*]:[*]:n:1
6. Unknown ECFP_2 feature: 1427820655: [*]CS[c](:[*]):[*]
7. Unknown ECFP_2 feature: -1841325949: [*]:[c](:[*])C(C)[c](:[*]):[*]
8. Unknown ECFP_2 feature: 1092541557: [*]C([*])[c]1:o:[*]:[*]:n:1
9. Unknown ECFP_2 feature: -1956535100: [*][c](:[*]):[c]([N+](=[*])[*]):c:[*]
10. Unknown ECFP_2 feature: -215026467: [*]:[c](:[*])[N+](=O)[O-]
11. Unknown ECFP_2 feature: 2104376220: [*][N+](=O)[*]
12. Unknown ECFP_2 feature: -659271057: [*][N+](=[*])[O-]

Feature Contribution

Top features for positive contribution

Fingerprint	Bit/Smiles	Feature Structure	Score	Carcinogen in training set

ECFP_12	1203316083	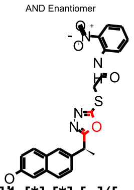 [*][c]1:[*]:[*]:[c]([*]):o:1	0.681	9 out of 13
ECFP_12	-1236483485	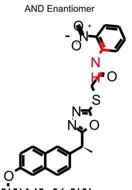 [*]C(=[*])N[c](:[*]): [*]	0.460	9 out of 17
ECFP_12	683445015	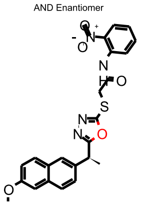 [*]:o:[*]	0.294	28 out of 66

Top Features for negative contribution

Fingerprint	Bit/Smiles	Feature Structure	Score	Carcinogen in training set
ECFP_12	1264202888	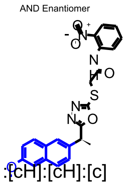 [*][c]1:[cH]:[cH]:[c] 2:[cH]:[c](OC):[cH]: [cH]:[c]:2:[cH]:1	-0.485	0 out of 2
ECFP_12	-1145977934	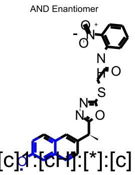 [*]O[c]1:[cH]:[*]:[c] (:[*]):[c](:[cH]:[*]):[cH]:1	-0.485	0 out of 2

ECFP_12	-1163815636	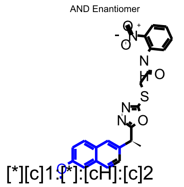 [*][c]1:[*]:[cH]:[c]2:[cH]:[c](OC):[cH]:[cH]:[c]:2:[cH]:1	-0.485	0 out of 2

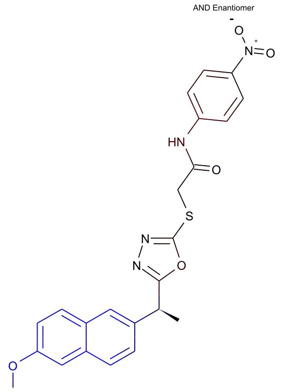

$C_{23}H_{20}N_4O_5S$
Molecular Weight: 464.4937
ALogP: 4.132
Rotatable Bonds: 8
Acceptors: 7
Donors: 1

Model Prediction

Prediction: Non-Carcinogen
Probability: 0.246
Enrichment: 0.763
Bayesian Score: -3.075
Mahalanobis Distance: 10.885
Mahalanobis Distance p-value: 0.0854

Prediction: Positive if the Bayesian score is above the estimated best cutoff value from minimizing the false positive and false negative rate.
Probability: The esimated probability that the sample is in the positive category. This assumes that the Bayesian score follows a normal distribution and is different from the prediction using a cutoff.
Enrichment: An estimate of enrichment, that is, the increased likelihood (versus random) of this sample being in the category.
Bayesian Score: The standard Laplacian-modified Bayesian score.
Mahalanobis Distance: The Mahalanobis distance (MD) is the distance to the center of the training data. The larger the MD, the less trustworthy the prediction.
Mahalanobis Distance p-value: The p-value gives the fraction of training data with an MD greater than or equal to the one for the given sample, assuming normally distributed data. The smaller the p-value, the less trustworthy the prediciton. For highly non-normal X properties (e.g., fingerprints), the MD p-value is wildly inaccurate.

Structural Similar Compounds

Name	Carbenicillin	Nimodipine	Nicardipine
Structure	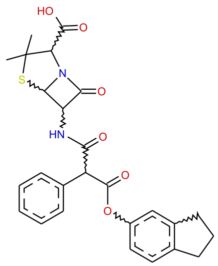	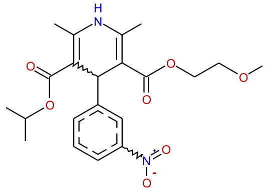	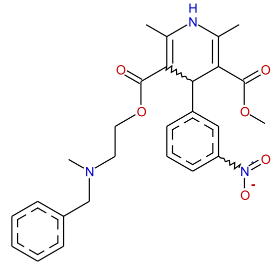
Actual Endpoint	Non-Carcinogen	Non-Carcinogen	Carcinogen
Predicted Endpoint	Non-Carcinogen	Non-Carcinogen	Carcinogen
Distance	0.646	0.660	0.672
Reference	US FDA (Centre for Drug Eval.& Res./Off. Testing & Res.) Sept. 1997	US FDA (Centre for Drug Eval.& Res./Off. Testing & Res.) Sept. 1997	US FDA (Centre for Drug Eval.& Res./Off. Testing & Res.) Sept. 1997

Model Applicability

Unknown features are fingerprint features in the query molecule, but not found in the training set.

1. All properties and OPS components are within expected ranges.
2. Unknown ECFP_2 feature: 1043790491: [*][N+](=[*])[*]
3. Unknown ECFP_2 feature: 781519895: [*][O-]
4. Unknown ECFP_2 feature: -955816473: [*]SCC(=[*])[*]
5. Unknown ECFP_2 feature: 1093109320: [*]S[c]1:o:[*]:[*]:n:1
6. Unknown ECFP_2 feature: 1427820655: [*]CS[c](:[*]):[*]
7. Unknown ECFP_2 feature: -1841325949: [*]:[c](:[*])C(C)[c](:[*]):[*]
8. Unknown ECFP_2 feature: 1092541557: [*]C([*])[c]1:o:[*]:[*]:n:1
9. Unknown ECFP_2 feature: -179073144: [*][N+](=[*])[c](:c:[*]):c:[*]
10. Unknown ECFP_2 feature: -215026467: [*]:[c](:[*])[N+](=O)[O-]
11. Unknown ECFP_2 feature: 2104376220: [*][N+](=O)[*]
12. Unknown ECFP_2 feature: -659271057: [*][N+](=[*])[O-]

Feature Contribution

Top features for positive contribution

Fingerprint	Bit/Smiles	Feature Structure	Score	Carcinogen in training set

ECFP_12	1203316083	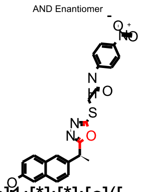 [*][c]1:[*]:[*]:[c]([*]):o:1	0.681	9 out of 13
ECFP_12	-177077903	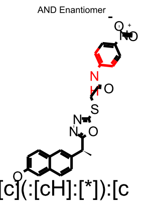 [*]N[c](:[cH]:[*]):[c H]:[*]	0.529	6 out of 10
ECFP_12	-1236483485	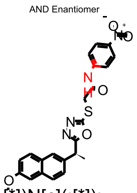 [*]C(=[*])N[c](:[*]): [*]	0.460	9 out of 17
**Top Features for negative contribution**				
**Fingerprint**	**Bit/Smiles**	**Feature Structure**	**Score**	**Carcinogen in training set**
ECFP_12	1153577237	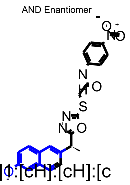 [*]O[c]1:[cH]:[cH]:[c]2:[cH]:[c]([*]):[*] :[cH]:[c]:2:[cH]:1	-0.485	0 out of 2
ECFP_12	1264202888	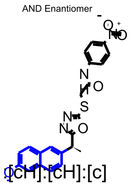 [*][c]1:[cH]:[cH]:[c] 2:[cH]:[c](OC):[cH]: [cH]:[c]:2:[cH]:1	-0.485	0 out of 2

ECFP_12	-1163815636	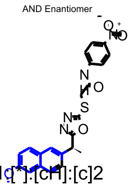 [*][c]1[c][*]:[cH]:[c]2:[cH]:[c](OC):[cH]:[cH]:[c]:2:[cH]:1	-0.485	0 out of 2

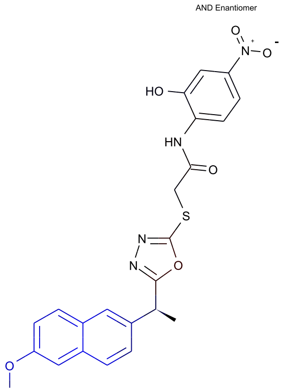

$C_{23}H_{20}N_4O_6S$

Molecular Weight: 480.4931

ALogP: 3.89

Rotatable Bonds: 8

Acceptors: 8

Donors: 2

Model Prediction

Prediction: Non-Carcinogen

Probability: 0.222

Enrichment: 0.690

Bayesian Score: -4.913

Mahalanobis Distance: 12.076

Mahalanobis Distance p-value: 0.00376

Prediction: Positive if the Bayesian score is above the estimated best cutoff value from minimizing the false positive and false negative rate.
Probability: The esimated probability that the sample is in the positive category. This assumes that the Bayesian score follows a normal distribution and is different from the prediction using a cutoff.
Enrichment: An estimate of enrichment, that is, the increased likelihood (versus random) of this sample being in the category.
Bayesian Score: The standard Laplacian-modified Bayesian score.
Mahalanobis Distance: The Mahalanobis distance (MD) is the distance to the center of the training data. The larger the MD, the less trustworthy the prediction.
Mahalanobis Distance p-value: The p-value gives the fraction of training data with an MD greater than or equal to the one for the given sample, assuming normally distributed data. The smaller the p-value, the less trustworthy the prediciton. For highly non-normal X properties (e.g., fingerprints), the MD p-value is wildly inaccurate.

Structural Similar Compounds

Name	Carbenicillin	Nimodipine	Sulfasalazine
Structure	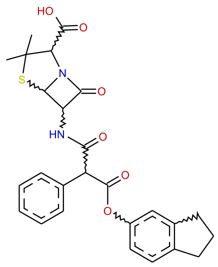	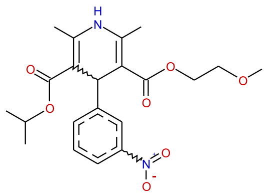	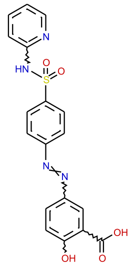
Actual Endpoint	Non-Carcinogen	Non-Carcinogen	Non-Carcinogen
Predicted Endpoint	Non-Carcinogen	Non-Carcinogen	Non-Carcinogen
Distance	0.612	0.707	0.719
Reference	US FDA (Centre for Drug Eval.& Res./Off. Testing & Res.) Sept. 1997	US FDA (Centre for Drug Eval.& Res./Off. Testing & Res.) Sept. 1997	US FDA (Centre for Drug Eval.& Res./Off. Testing & Res.) Sept. 1997

Model Applicability

Unknown features are fingerprint features in the query molecule, but not found in the training set.

1. All properties and OPS components are within expected ranges.
2. Unknown ECFP_2 feature: 1043790491: [*][N+](=[*])[*]
3. Unknown ECFP_2 feature: 781519895: [*][O-]
4. Unknown ECFP_2 feature: -955816473: [*]SCC(=[*])[*]
5. Unknown ECFP_2 feature: 1093109320: [*]S[c]1:o:[*]:[*]:n:1
6. Unknown ECFP_2 feature: 1427820655: [*]CS[c](:[*]):[*]
7. Unknown ECFP_2 feature: -1841325949: [*]:[c](:[*])C(C)[c](:[*]):[*]
8. Unknown ECFP_2 feature: 1092541557: [*]C([*])[c]1:o:[*]:[*]:n:1
9. Unknown ECFP_2 feature: -179073144: [*][N+](=[*])[c](:c:[*]):c:[*]
10. Unknown ECFP_2 feature: -215026467: [*]:[c](:[*])[N+](=O)[O-]
11. Unknown ECFP_2 feature: 2104376220: [*][N+](=O)[*]
12. Unknown ECFP_2 feature: -659271057: [*][N+](=[*])[O-]

Feature Contribution

Top features for positive contribution				
Fingerprint	Bit/Smiles	Feature Structure	Score	Carcinogen in training set

ECFP_12	1203316083	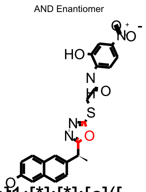 [*][c]1:[*]:[*]:[c]([*]):o:1	0.681	9 out of 13
ECFP_12	-1236483485	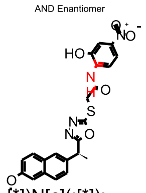 [*]C(=[*])N[c](:[*]):[*]	0.460	9 out of 17
ECFP_12	683445015	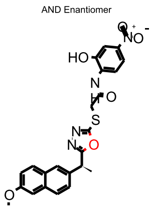 [*]:o:[*]	0.294	28 out of 66
**Top Features for negative contribution**				
**Fingerprint**	**Bit/Smiles**	**Feature Structure**	**Score**	**Carcinogen in training set**
ECFP_12	-1731425419	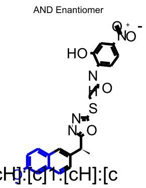 [*]:[cH]:[c]1:[cH]:[c](OC):[cH]:[cH]:[c]:1:[*]	-0.485	0 out of 2
ECFP_12	-1145977934	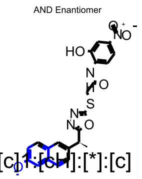 [*]O[c]1:[cH]:[*]:[c](:[*]):[c](:[cH]:[*]):[cH]:1	-0.485	0 out of 2

ECFP_12	2082242578	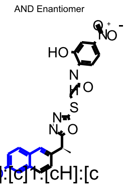 [*]:[cH]:[c]1:[cH]:[cH]:[c](OC):[cH]:[c]:1:[*]	-0.485	0 out of 2

Sorafenib

TOPKAT_Rat_Female_FDA_None_vs_Carcinogen

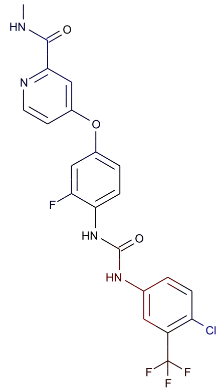

$C_{21}H_{15}ClF_4N_4O_3$

Molecular Weight: 482.81541

ALogP: 4.381

Rotatable Bonds: 6

Acceptors: 4

Donors: 3

Model Prediction

Prediction: Non-Carcinogen

Probability: 0.225

Enrichment: 0.699

Bayesian Score: -4.657

Mahalanobis Distance: 12.308

Mahalanobis Distance p-value: 0.00177

Prediction: Positive if the Bayesian score is above the estimated best cutoff value from minimizing the false positive and false negative rate.
Probability: The esimated probability that the sample is in the positive category. This assumes that the Bayesian score follows a normal distribution and is different from the prediction using a cutoff.
Enrichment: An estimate of enrichment, that is, the increased likelihood (versus random) of this sample being in the category.
Bayesian Score: The standard Laplacian-modified Bayesian score.
Mahalanobis Distance: The Mahalanobis distance (MD) is the distance to the center of the training data. The larger the MD, the less trustworthy the prediction.
Mahalanobis Distance p-value: The p-value gives the fraction of training data with an MD greater than or equal to the one for the given sample, assuming normally distributed data. The smaller the p-value, the less trustworthy the prediciton. For highly non-normal X properties (e.g., fingerprints), the MD p-value is wildly inaccurate.

Structural Similar Compounds

Name	Glimepride	Glyburide	Fluvastatin
Structure	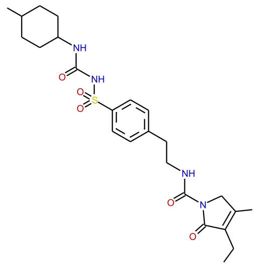	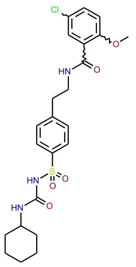	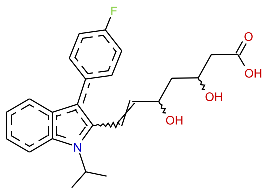
Actual Endpoint	Non-Carcinogen	Non-Carcinogen	Non-Carcinogen
Predicted Endpoint	Non-Carcinogen	Non-Carcinogen	Non-Carcinogen
Distance	0.623	0.633	0.654
Reference	US FDA (Centre for Drug Eval.& Res./Off. Testing & Res.) Sept. 1997	US FDA (Centre for Drug Eval.& Res./Off. Testing & Res.) Sept. 1997	US FDA (Centre for Drug Eval.& Res./Off. Testing & Res.) Sept. 1997

Model Applicability

Unknown features are fingerprint features in the query molecule, but not found in the training set.

1. All properties and OPS components are within expected ranges.

Feature Contribution

Top features for positive contribution				
**Fingerprint**	**Bit/Smiles**	**Feature Structure**	**Score**	**Carcinogen in training set**
ECFP_12	-970385855	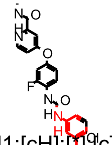 [*]N[c]1:[cH]:[c]:[c]([*]):[c](:[cH]:1)C([*])([*])[*]	0.613	2 out of 2

ECFP_12	-177077903	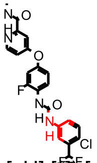 [*]N[c](:[cH]:[*]):[cH]:[*]	0.529	6 out of 10
ECFP_12	-1236483485	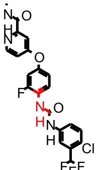 [*]C(=[*])N[c](:[*]):[*]	0.460	9 out of 17

Top Features for negative contribution

Fingerprint	Bit/Smiles	Feature Structure	Score	Carcinogen in training set
ECFP_12	1335691903	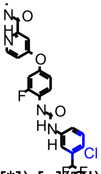 [*][c](:[*]):[c](Cl):[cH]:[*]	-1.112	2 out of 26
ECFP_12	99947387	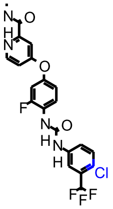 [*]:[c](:[*])Cl	-0.817	8 out of 62
ECFP_12	1413420509	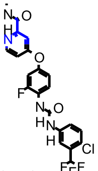 [*]C(=[*])[c](:[cH]:[*]):n:[*]	-0.661	0 out of 3

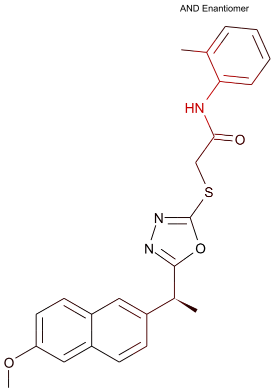

$C_{24}H_{23}N_3O_3S$
Molecular Weight: 433.52272
ALogP: 4.724
Rotatable Bonds: 7
Acceptors: 5
Donors: 1

Structural Similar Compounds

Name	Simvastatin	Moricizine	Diltiazem
Structure	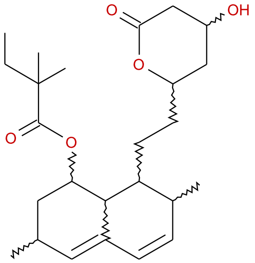	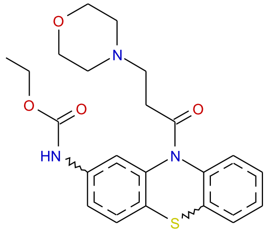	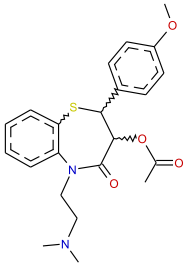
Actual Endpoint	Multiple-Carcinogen	Single-Carcinogen	Multiple-Carcinogen
Predicted Endpoint	Multiple-Carcinogen	Single-Carcinogen	Multiple-Carcinogen
Distance	0.554	0.595	0.615
Reference	US FDA (Centre for Drug Eval.& Res./Off. Testing & Res.) Sept. 1997	US FDA (Centre for Drug Eval.& Res./Off. Testing & Res.) Sept. 1997	US FDA (Centre for Drug Eval.& Res./Off. Testing & Res.) Sept. 1997

Model Prediction

Prediction: Multiple-Carcinogen
Probability: 0.753
Enrichment: 2.014
Bayesian Score: 6.878
Mahalanobis Distance: 13.652
Mahalanobis Distance p-value: 5.67e-005

Prediction: Positive if the Bayesian score is above the estimated best cutoff value from minimizing the false positive and false negative rate.
Probability: The esimated probability that the sample is in the positive category. This assumes that the Bayesian score follows a normal distribution and is different from the prediction using a cutoff.
Enrichment: An estimate of enrichment, that is, the increased likelihood (versus random) of this sample being in the category.
Bayesian Score: The standard Laplacian-modified Bayesian score.
Mahalanobis Distance: The Mahalanobis distance (MD) is the distance to the center of the training data. The larger the MD, the less trustworthy the prediction.
Mahalanobis Distance p-value: The p-value gives the fraction of training data with an MD greater than or equal to the one for the given sample, assuming normally distributed data. The smaller the p-value, the less trustworthy the prediciton. For highly non-normal X properties (e.g., fingerprints), the MD p-value is wildly inaccurate.

Model Applicability

Unknown features are fingerprint features in the query molecule, but not found in the training set.

1. OPS PC12 out of range. Value: 3.1709. Training min, max, SD, explained variance: -2.8991, 3.0113, 1.313, 0.0255.

Feature Contribution

Top features for positive contribution				
**Fingerprint**	**Bit/Smiles**	**Feature Structure**	**Score**	**Multiple-Carcinogen in training set**
SCFP_4	2097618059	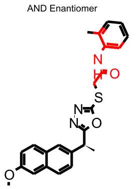 [*]CC(=O)N[c](:[cH]:[*]):[c]([*]):[*]	0.730	5 out of 6

SCFP_4	1792377291	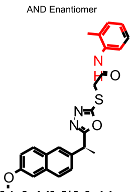 [*]N[c]1:[cH]:[*]:[cH]:[cH]:[c]:1C	0.610	2 out of 2
SCFP_4	1631845520	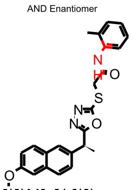 [*]C(=[*])N[c](:[*]): [*]	0.601	6 out of 9
**Top Features for negative contribution**				
**Fingerprint**	**Bit/Smiles**	**Feature Structure**	**Score**	**Multiple-Carcinogen in training set**
SCFP_4	112346096	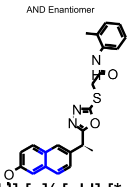 [*]:[cH]:[c](:[cH]:[*]):[c](:[*]):[*]	-0.730	1 out of 10
SCFP_4	-1379673609	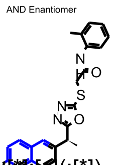 [*][c]1:[*]:[c](:[*]) :[c](:[cH]:[*]):[cH] :[cH]:1	-0.472	1 out of 7
SCFP_4	276283342	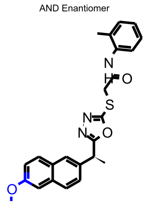 [*]:[c](:[*])OC	-0.106	5 out of 18

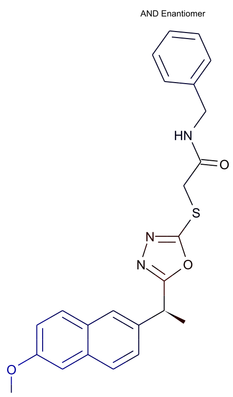

$C_{24}H_{23}N_3O_3S$
Molecular Weight: 433.52272
ALogP: 4.245
Rotatable Bonds: 8
Acceptors: 5
Donors: 1

Structural Similar Compounds

Name	Felodipine	Lovastatin	Simvastatin
Structure	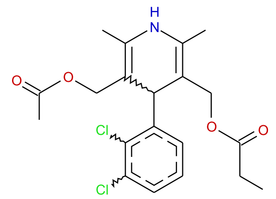	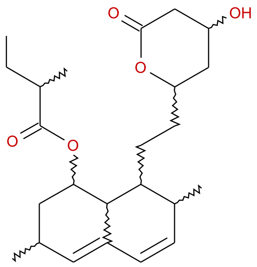	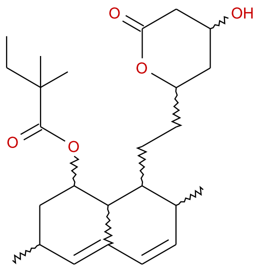
Actual Endpoint	Carcinogen	Carcinogen	Carcinogen
Predicted Endpoint	Carcinogen	Carcinogen	Carcinogen
Distance	0.570	0.588	0.596
Reference	US FDA (Centre for Drug Eval.& Res./Off. Testing & Res.) Sept. 1997	US FDA (Centre for Drug Eval.& Res./Off. Testing & Res.) Sept. 1997	US FDA (Centre for Drug Eval.& Res./Off. Testing & Res.) Sept. 1997

Model Prediction

Prediction: Non-Carcinogen
Probability: 0.295
Enrichment: 0.883
Bayesian Score: -2.321
Mahalanobis Distance: 13.816
Mahalanobis Distance p-value: 8.79e-005

Prediction: Positive if the Bayesian score is above the estimated best cutoff value from minimizing the false positive and false negative rate.
Probability: The esimated probability that the sample is in the positive category. This assumes that the Bayesian score follows a normal distribution and is different from the prediction using a cutoff.
Enrichment: An estimate of enrichment, that is, the increased likelihood (versus random) of this sample being in the category.
Bayesian Score: The standard Laplacian-modified Bayesian score.
Mahalanobis Distance: The Mahalanobis distance (MD) is the distance to the center of the training data. The larger the MD, the less trustworthy the prediction.
Mahalanobis Distance p-value: The p-value gives the fraction of training data with an MD greater than or equal to the one for the given sample, assuming normally distributed data. The smaller the p-value, the less trustworthy the prediciton. For highly non-normal X properties (e.g., fingerprints), the MD p-value is wildly inaccurate.

Model Applicability

Unknown features are fingerprint features in the query molecule, but not found in the training set.

1. All properties and OPS components are within expected ranges.

Feature Contribution

Top features for positive contribution

Fingerprint	Bit/Smiles	Feature Structure	Score	Carcinogen in training set
SCFP_6	794417578	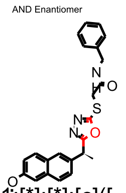 [*][c]1:[*]:[*]:[c]([*]):o:1	0.600	9 out of 14

SCFP_6	149212520	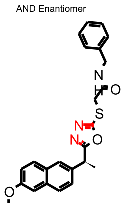 [*][c]1:[*]:[*]:n:n:1	0.543	9 out of 15
SCFP_6	-1379673609	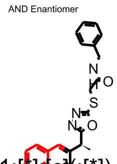 [*][c]1:[*]:[c](:[*]):[c](:[cH]:[*]):[cH]:[cH]:1	0.526	11 out of 19

Top Features for negative contribution

Fingerprint	Bit/Smiles	Feature Structure	Score	Carcinogen in training set
SCFP_6	-576781090	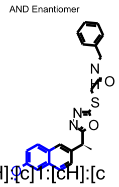 [*]:[cH]:[c]1:[cH]:[c](OC):[cH]:[cH]:[c]:1:[*]	-0.825	0 out of 4
SCFP_6	1653911926	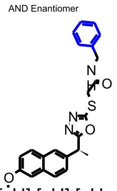 [*][c]1:[cH]:[cH]:[cH]:[cH]:[cH]:1	-0.504	12 out of 64
SCFP_6	2029059671	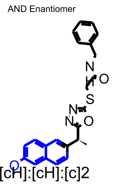 CO[c]1:[cH]:[cH]:[c]2:[cH]:[*]:[cH]:[cH]:[c]:2:[cH]:1	-0.496	0 out of 2

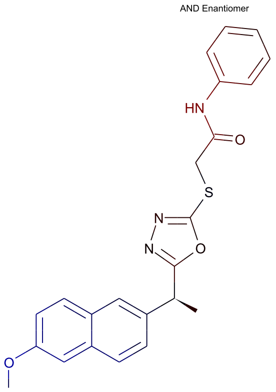

$C_{23}H_{21}N_{3}O_{3}S$
Molecular Weight: 419.49614
ALogP: 4.238
Rotatable Bonds: 7
Acceptors: 5
Donors: 1

Model Prediction

Prediction: Carcinogen
Probability: 0.377
Enrichment: 1.128
Bayesian Score: 0.705
Mahalanobis Distance: 14.198
Mahalanobis Distance p-value: 1.94e-005

Prediction: Positive if the Bayesian score is above the estimated best cutoff value from minimizing the false positive and false negative rate.
Probability: The esimated probability that the sample is in the positive category. This assumes that the Bayesian score follows a normal distribution and is different from the prediction using a cutoff.
Enrichment: An estimate of enrichment, that is, the increased likelihood (versus random) of this sample being in the category.
Bayesian Score: The standard Laplacian-modified Bayesian score.
Mahalanobis Distance: The Mahalanobis distance (MD) is the distance to the center of the training data. The larger the MD, the less trustworthy the prediction.
Mahalanobis Distance p-value: The p-value gives the fraction of training data with an MD greater than or equal to the one for the given sample, assuming normally distributed data. The smaller the p-value, the less trustworthy the prediciton. For highly non-normal X properties (e.g., fingerprints), the MD p-value is wildly inaccurate.

Structural Similar Compounds

Name	Lovastatin	Moricizine	Simvastatin
Structure	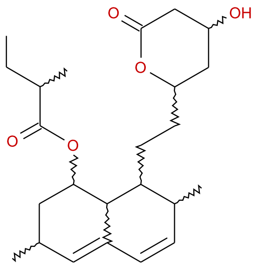	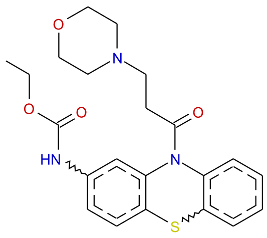	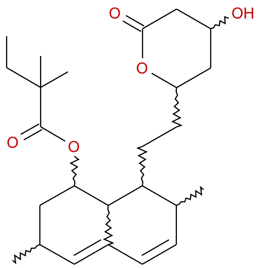
Actual Endpoint	Carcinogen	Carcinogen	Carcinogen
Predicted Endpoint	Carcinogen	Carcinogen	Carcinogen
Distance	0.572	0.585	0.589
Reference	US FDA (Centre for Drug Eval.& Res./Off. Testing & Res.) Sept. 1997	US FDA (Centre for Drug Eval.& Res./Off. Testing & Res.) Sept. 1997	US FDA (Centre for Drug Eval.& Res./Off. Testing & Res.) Sept. 1997

Model Applicability

Unknown features are fingerprint features in the query molecule, but not found in the training set.

1. All properties and OPS components are within expected ranges.

Feature Contribution

Top features for positive contribution

Fingerprint	Bit/Smiles	Feature Structure	Score	Carcinogen in training set
SCFP_6	-347048986	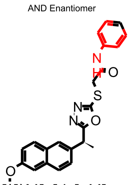 [*]C(=[*])N[c]1:[cH]:[cH]:[*]:[cH]:[cH]:1	0.615	5 out of 7

SCFP_6	814408713	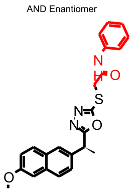 [*]CC(=O)N[c]1:[cH]:[cH]:[cH]:[cH]:[cH]:1	0.603	2 out of 2
SCFP_6	794417578	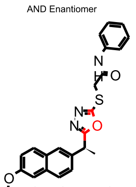 [*][c]1:[*]:[*]:[c]([*]):o:1	0.600	9 out of 14

Top Features for negative contribution

Fingerprint	Bit/Smiles	Feature Structure	Score	Carcinogen in training set
SCFP_6	-576781090	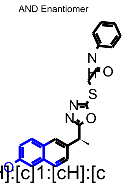 [*]:[cH]:[c]1:[cH]:[c](OC):[cH]:[cH]:[c]:1:[*]	-0.825	0 out of 4
SCFP_6	1653911926	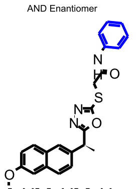 [*][c]1:[cH]:[cH]:[cH]:[cH]:[cH]:1	-0.504	12 out of 64
SCFP_6	2029059671	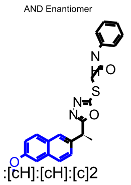 CO[c]1:[cH]:[cH]:[c]2:[cH]:[*]:[cH]:[cH]:[c]:2:[cH]:1	-0.496	0 out of 2

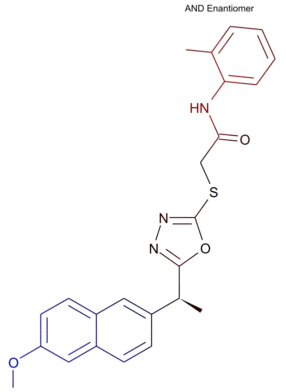

$C_{24}H_{23}N_{3}O_{3}S$
Molecular Weight: 433.52272
ALogP: 4.724
Rotatable Bonds: 7
Acceptors: 5
Donors: 1

Structural Similar Compounds

Name	Simvastatin	Lovastatin	Felodipine
Structure	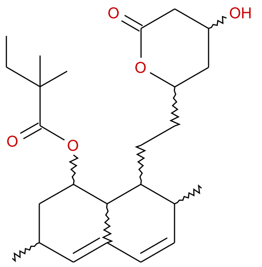	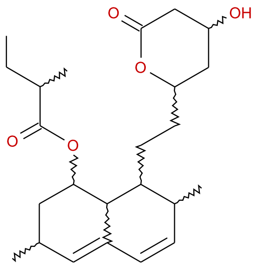	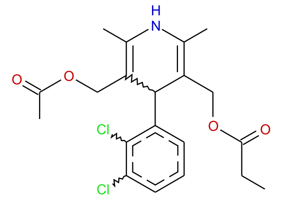
Actual Endpoint	Carcinogen	Carcinogen	Carcinogen
Predicted Endpoint	Carcinogen	Carcinogen	Carcinogen
Distance	0.572	0.581	0.607
Reference	US FDA (Centre for Drug Eval.& Res./Off. Testing & Res.) Sept. 1997	US FDA (Centre for Drug Eval.& Res./Off. Testing & Res.) Sept. 1997	US FDA (Centre for Drug Eval.& Res./Off. Testing & Res.) Sept. 1997

Model Prediction

Prediction: Carcinogen
Probability: 0.406
Enrichment: 1.215
Bayesian Score: 1.633
Mahalanobis Distance: 14.737
Mahalanobis Distance p-value: 1.98e-006

Prediction: Positive if the Bayesian score is above the estimated best cutoff value from minimizing the false positive and false negative rate.
Probability: The esimated probability that the sample is in the positive category. This assumes that the Bayesian score follows a normal distribution and is different from the prediction using a cutoff.
Enrichment: An estimate of enrichment, that is, the increased likelihood (versus random) of this sample being in the category.
Bayesian Score: The standard Laplacian-modified Bayesian score.
Mahalanobis Distance: The Mahalanobis distance (MD) is the distance to the center of the training data. The larger the MD, the less trustworthy the prediction.
Mahalanobis Distance p-value: The p-value gives the fraction of training data with an MD greater than or equal to the one for the given sample, assuming normally distributed data. The smaller the p-value, the less trustworthy the prediciton. For highly non-normal X properties (e.g., fingerprints), the MD p-value is wildly inaccurate.

Model Applicability

Unknown features are fingerprint features in the query molecule, but not found in the training set.

1. All properties and OPS components are within expected ranges.

Feature Contribution

Top features for positive contribution

Fingerprint	Bit/Smiles	Feature Structure	Score	Carcinogen in training set
SCFP_6	794417578	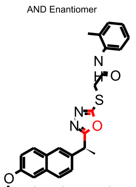 [*][c]1:[*]:[*]:[c]([*]):o:1	0.600	9 out of 14

SCFP_6	149212520	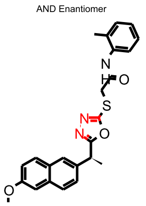 [*][c]1:[*]:[*]:n:n:1	0.543	9 out of 15
SCFP_6	-1379673609	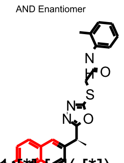 [*][c]1:[*]:[c](:[*]):[c](:[cH]:[*]):[cH]:[cH]:1	0.526	11 out of 19
**Top Features for negative contribution**				
**Fingerprint**	**Bit/Smiles**	**Feature Structure**	**Score**	**Carcinogen in training set**
SCFP_6	-576781090	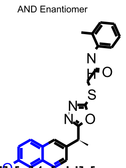 [*]:[cH]:[c]1:[cH]:[c](OC):[cH]:[cH]:[c]:1:[*]	-0.825	0 out of 4
SCFP_6	1469959550	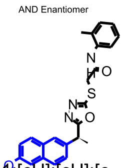 [*]O[c]1:[cH]:[cH]:[c]2:[cH]:[c]([*]):[cH]:[cH]:[c]:2:[cH]:1	-0.496	0 out of 2
SCFP_6	921696937	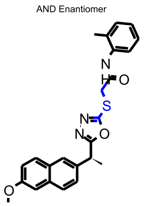 [*]CS[c](:[*]):[*]	-0.496	0 out of 2

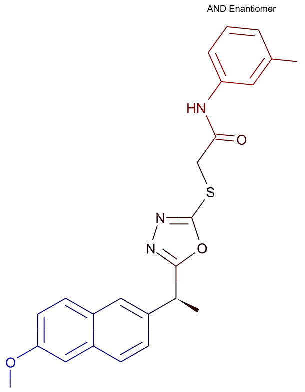

$C_{24}H_{23}N_3O_3S$
Molecular Weight: 433.52272
ALogP: 4.724
Rotatable Bonds: 7
Acceptors: 5
Donors: 1

Model Prediction

Prediction: Carcinogen
Probability: 0.416
Enrichment: 1.244
Bayesian Score: 1.932
Mahalanobis Distance: 13.769
Mahalanobis Distance p-value: 0.000105

Prediction: Positive if the Bayesian score is above the estimated best cutoff value from minimizing the false positive and false negative rate.
Probability: The esimated probability that the sample is in the positive category. This assumes that the Bayesian score follows a normal distribution and is different from the prediction using a cutoff.
Enrichment: An estimate of enrichment, that is, the increased likelihood (versus random) of this sample being in the category.
Bayesian Score: The standard Laplacian-modified Bayesian score.
Mahalanobis Distance: The Mahalanobis distance (MD) is the distance to the center of the training data. The larger the MD, the less trustworthy the prediction.
Mahalanobis Distance p-value: The p-value gives the fraction of training data with an MD greater than or equal to the one for the given sample, assuming normally distributed data. The smaller the p-value, the less trustworthy the prediciton. For highly non-normal X properties (e.g., fingerprints), the MD p-value is wildly inaccurate.

Structural Similar Compounds

Name	Simvastatin	Lovastatin	Felodipine
Structure	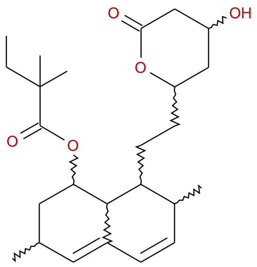	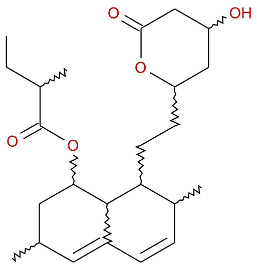	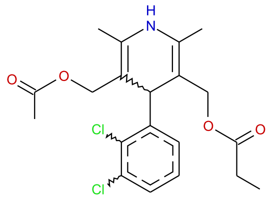
Actual Endpoint	Carcinogen	Carcinogen	Carcinogen
Predicted Endpoint	Carcinogen	Carcinogen	Carcinogen
Distance	0.572	0.581	0.608
Reference	US FDA (Centre for Drug Eval.& Res./Off. Testing & Res.) Sept. 1997	US FDA (Centre for Drug Eval.& Res./Off. Testing & Res.) Sept. 1997	US FDA (Centre for Drug Eval.& Res./Off. Testing & Res.) Sept. 1997

Model Applicability

Unknown features are fingerprint features in the query molecule, but not found in the training set.

1. All properties and OPS components are within expected ranges.

Feature Contribution

Top features for positive contribution

Fingerprint	Bit/Smiles	Feature Structure	Score	Carcinogen in training set
SCFP_6	-347048986	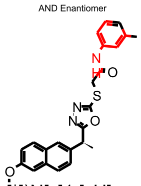 [*]C(=[*])N[c]1:[cH]:[cH]:[*]:[cH]:[cH]:1	0.615	5 out of 7

SCFP_6	-236487363	AND Enantiomer 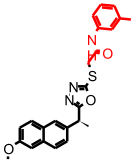 [*]CC(=O)N[c]1:[cH]:[cH]:[cH]:[c](C):[cH]:1	0.603	2 out of 2
SCFP_6	794417578	AND Enantiomer 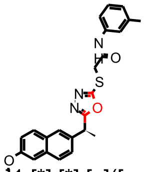 [*][c]1:[*]:[*]:[c]([*]):o:1	0.600	9 out of 14

Top Features for negative contribution

Fingerprint	Bit/Smiles	Feature Structure	Score	Carcinogen in training set
SCFP_6	-576781090	AND Enantiomer 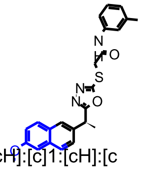 [*]:[cH]:[c]1:[cH]:[c](OC):[cH]:[cH]:[c]:1:[*]	-0.825	0 out of 4
SCFP_6	-1889730273	AND Enantiomer 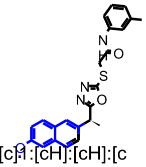 [*]O[c]1:[cH]:[cH]:[c]2:[cH]:[c]([*]):[*]:[cH]:[c]:2:[cH]:1	-0.496	0 out of 2
SCFP_6	1469959550	AND Enantiomer 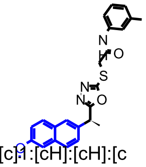 [*]O[c]1:[cH]:[cH]:[c]2:[cH]:[c]([*]):[cH]:[cH]:[c]:2:[cH]:1	-0.496	0 out of 2

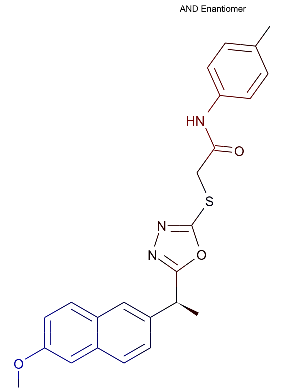

$C_{24}H_{23}N_3O_3S$
Molecular Weight: 433.52272
ALogP: 4.724
Rotatable Bonds: 7
Acceptors: 5
Donors: 1

Model Prediction

Prediction: Carcinogen
Probability: 0.382
Enrichment: 1.143
Bayesian Score: 0.864
Mahalanobis Distance: 13.906
Mahalanobis Distance p-value: 6.22e-005

Prediction: Positive if the Bayesian score is above the estimated best cutoff value from minimizing the false positive and false negative rate.
Probability: The esimated probability that the sample is in the positive category. This assumes that the Bayesian score follows a normal distribution and is different from the prediction using a cutoff.
Enrichment: An estimate of enrichment, that is, the increased likelihood (versus random) of this sample being in the category.
Bayesian Score: The standard Laplacian-modified Bayesian score.
Mahalanobis Distance: The Mahalanobis distance (MD) is the distance to the center of the training data. The larger the MD, the less trustworthy the prediction.
Mahalanobis Distance p-value: The p-value gives the fraction of training data with an MD greater than or equal to the one for the given sample, assuming normally distributed data. The smaller the p-value, the less trustworthy the prediciton. For highly non-normal X properties (e.g., fingerprints), the MD p-value is wildly inaccurate.

Structural Similar Compounds

Name	Simvastatin	Lovastatin	Felodipine
Structure	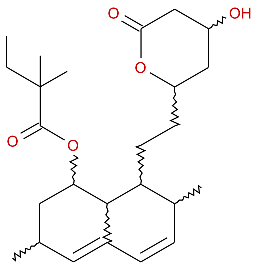	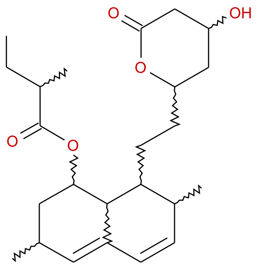	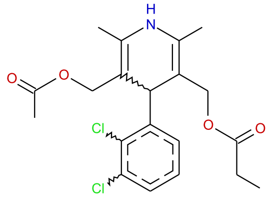
Actual Endpoint	Carcinogen	Carcinogen	Carcinogen
Predicted Endpoint	Carcinogen	Carcinogen	Carcinogen
Distance	0.572	0.580	0.611
Reference	US FDA (Centre for Drug Eval.& Res./Off. Testing & Res.) Sept. 1997	US FDA (Centre for Drug Eval.& Res./Off. Testing & Res.) Sept. 1997	US FDA (Centre for Drug Eval.& Res./Off. Testing & Res.) Sept. 1997

Model Applicability

Unknown features are fingerprint features in the query molecule, but not found in the training set.

1. All properties and OPS components are within expected ranges.

Feature Contribution

Top features for positive contribution				
**Fingerprint**	**Bit/Smiles**	**Feature Structure**	**Score**	**Carcinogen in training set**
SCFP_6	-347048986	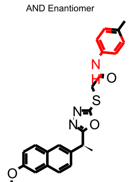 [*]C(=[*])N[c]1:[cH]:[cH]:[*]:[cH]:[cH]:1	0.615	5 out of 7

SCFP_6	814408713	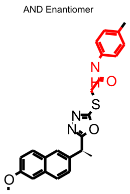 [*]CC(=O)N[c]1:[cH]:[cH]:[cH]:[cH]:[cH]:1	0.603	2 out of 2
SCFP_6	794417578	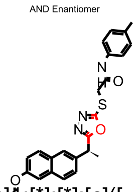 [*][c]1:[*]:[*]:[c]([*]):o:1	0.600	9 out of 14

Top Features for negative contribution

Fingerprint	Bit/Smiles	Feature Structure	Score	Carcinogen in training set
SCFP_6	-576781090	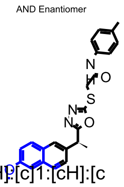 [*]:[cH]:[c]1:[cH]:[c](OC):[cH]:[cH]:[c]:1:[*]	-0.825	0 out of 4
SCFP_6	2029059671	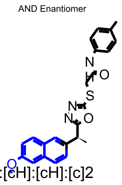 CO[c]1:[cH]:[cH]:[c]2:[cH]:[*]:[cH]:[cH]:[c]:2:[cH]:1	-0.496	0 out of 2
SCFP_6	1469959550	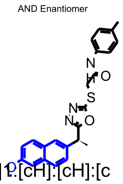 [*]O[c]1:[cH]:[cH]:[c]2:[cH]:[c]([*]):[cH]:[cH]:[c]:2:[cH]:1	-0.496	0 out of 2

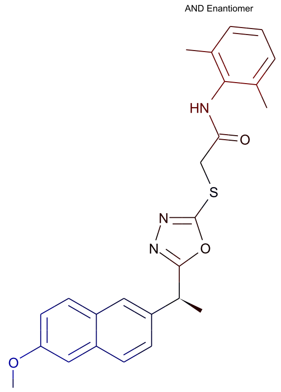

$C_{25}H_{25}N_3O_3S$
Molecular Weight: 447.5493
ALogP: 5.21
Rotatable Bonds: 7
Acceptors: 5
Donors: 1

Model Prediction

Prediction: Carcinogen
Probability: 0.409
Enrichment: 1.223
Bayesian Score: 1.710
Mahalanobis Distance: 14.747
Mahalanobis Distance p-value: 1.9e-006

Prediction: Positive if the Bayesian score is above the estimated best cutoff value from minimizing the false positive and false negative rate.
Probability: The esimated probability that the sample is in the positive category. This assumes that the Bayesian score follows a normal distribution and is different from the prediction using a cutoff.
Enrichment: An estimate of enrichment, that is, the increased likelihood (versus random) of this sample being in the category.
Bayesian Score: The standard Laplacian-modified Bayesian score.
Mahalanobis Distance: The Mahalanobis distance (MD) is the distance to the center of the training data. The larger the MD, the less trustworthy the prediction.
Mahalanobis Distance p-value: The p-value gives the fraction of training data with an MD greater than or equal to the one for the given sample, assuming normally distributed data. The smaller the p-value, the less trustworthy the prediciton. For highly non-normal X properties (e.g., fingerprints), the MD p-value is wildly inaccurate.

Structural Similar Compounds

Name	Simvastatin	Lovastatin	Felodipine
Structure	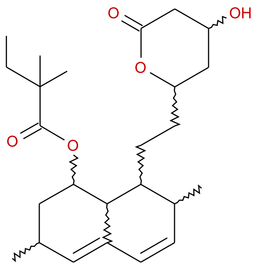	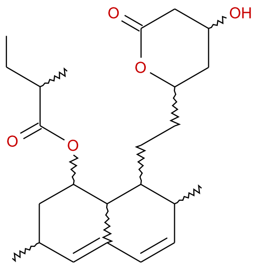	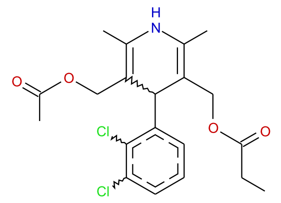
Actual Endpoint	Carcinogen	Carcinogen	Carcinogen
Predicted Endpoint	Carcinogen	Carcinogen	Carcinogen
Distance	0.585	0.613	0.638
Reference	US FDA (Centre for Drug Eval.& Res./Off. Testing & Res.) Sept. 1997	US FDA (Centre for Drug Eval.& Res./Off. Testing & Res.) Sept. 1997	US FDA (Centre for Drug Eval.& Res./Off. Testing & Res.) Sept. 1997

Model Applicability

Unknown features are fingerprint features in the query molecule, but not found in the training set.

1. All properties and OPS components are within expected ranges.

Feature Contribution

Top features for positive contribution

Fingerprint	Bit/Smiles	Feature Structure	Score	Carcinogen in training set
SCFP_6	794417578	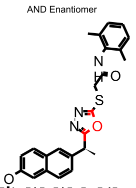 [*][c]1:[*]:[*]:[c]([*]):o:1	0.600	9 out of 14

SCFP_6	149212520	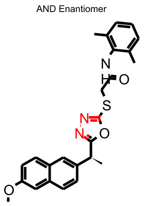<br>[*][c]1:[*]:[*]:n:n:1	0.543	9 out of 15
SCFP_6	-1379673609	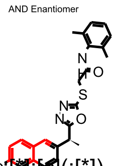<br>[*][c]1:[*]:[c](:[*])<br>:[c](:[cH]:[*]):[cH]<br>:[cH]:1	0.526	11 out of 19
**Top Features for negative contribution**				
**Fingerprint**	**Bit/Smiles**	**Feature Structure**	**Score**	**Carcinogen in training set**
SCFP_6	-576781090	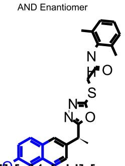<br>[*]:[cH]:[c]1:[cH]:[c<br>](OC):[cH]:[cH]:[c]:<br>1:[*]	-0.825	0 out of 4
SCFP_6	921696937	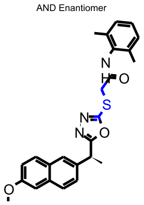<br>[*]CS[c](:[*]):[*]	-0.496	0 out of 2
SCFP_6	-1889730273	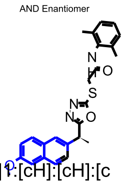<br>[*]O[c]1:[cH]:[cH]:[c<br>]2:[cH]:[c]([*]):[*]<br>:[cH]:[c]:2:[cH]:1	-0.496	0 out of 2

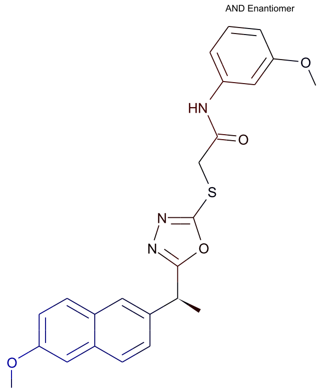

$C_{24}H_{23}N_3O_4S$
Molecular Weight: 449.52212
ALogP: 4.221
Rotatable Bonds: 8
Acceptors: 6
Donors: 1

Model Prediction

Prediction: Non-Carcinogen
Probability: 0.332
Enrichment: 0.993
Bayesian Score: -0.873
Mahalanobis Distance: 13.669
Mahalanobis Distance p-value: 0.000153

Prediction: Positive if the Bayesian score is above the estimated best cutoff value from minimizing the false positive and false negative rate.
Probability: The esimated probability that the sample is in the positive category. This assumes that the Bayesian score follows a normal distribution and is different from the prediction using a cutoff.
Enrichment: An estimate of enrichment, that is, the increased likelihood (versus random) of this sample being in the category.
Bayesian Score: The standard Laplacian-modified Bayesian score.
Mahalanobis Distance: The Mahalanobis distance (MD) is the distance to the center of the training data. The larger the MD, the less trustworthy the prediction.
Mahalanobis Distance p-value: The p-value gives the fraction of training data with an MD greater than or equal to the one for the given sample, assuming normally distributed data. The smaller the p-value, the less trustworthy the prediciton. For highly non-normal X properties (e.g., fingerprints), the MD p-value is wildly inaccurate.

Structural Similar Compounds

Name	Moricizine	Diltiazem	Felodipine
Structure	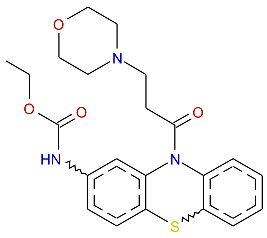	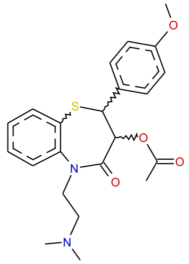	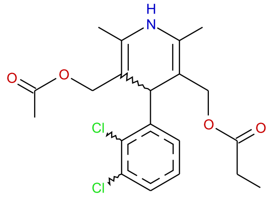
Actual Endpoint	Carcinogen	Non-Carcinogen	Carcinogen
Predicted Endpoint	Carcinogen	Non-Carcinogen	Carcinogen
Distance	0.605	0.614	0.618
Reference	US FDA (Centre for Drug Eval.& Res./Off. Testing & Res.) Sept. 1997	US FDA (Centre for Drug Eval.& Res./Off. Testing & Res.) Sept. 1997	US FDA (Centre for Drug Eval.& Res./Off. Testing & Res.) Sept. 1997

Model Applicability

Unknown features are fingerprint features in the query molecule, but not found in the training set.

1. All properties and OPS components are within expected ranges.

Feature Contribution

Top features for positive contribution

Fingerprint	Bit/Smiles	Feature Structure	Score	Carcinogen in training set
SCFP_6	-347048986	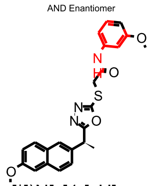 [*]C(=[*])N[c]1:[cH]:[cH]:[*]:[cH]:[cH]:1	0.615	5 out of 7

SCFP_6	794417578	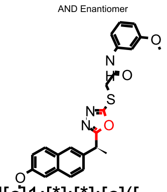 [*][c]1:[*]:[*]:[c]([*]):o:1	0.600	9 out of 14
SCFP_6	149212520	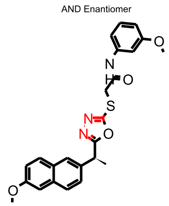 [*][c]1:[*]:[*]:n:n:1	0.543	9 out of 15

Top Features for negative contribution

Fingerprint	Bit/Smiles	Feature Structure	Score	Carcinogen in training set
SCFP_6	-576781090	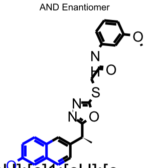 [*]:[cH]:[c]1:[cH]:[c](OC):[cH]:[cH]:[c]:1:[*]	-0.825	0 out of 4
SCFP_6	-1889730273	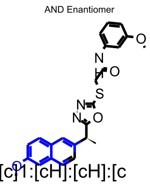 [*]O[c]1:[cH]:[cH]:[c]2:[cH]:[c]([*]):[*]:[cH]:[c]:2:[cH]:1	-0.496	0 out of 2
SCFP_6	1469959550	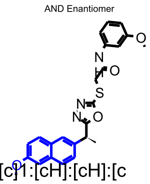 [*]O[c]1:[cH]:[cH]:[c]2:[cH]:[c]([*]):[cH]:[cH]:[c]:2:[cH]:1	-0.496	0 out of 2

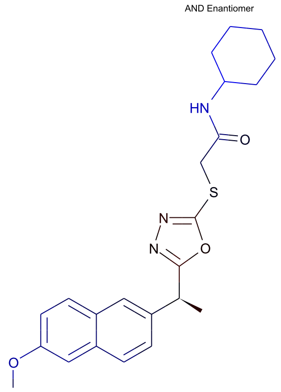

$C_{23}H_{27}N_3O_3S$
Molecular Weight: 425.54378
ALogP: 4.52
Rotatable Bonds: 7
Acceptors: 5
Donors: 1

Model Prediction

Prediction: Non-Carcinogen
Probability: 0.222
Enrichment: 0.665
Bayesian Score: -5.923
Mahalanobis Distance: 13.799
Mahalanobis Distance p-value: 9.39e-005

Prediction: Positive if the Bayesian score is above the estimated best cutoff value from minimizing the false positive and false negative rate.
Probability: The esimated probability that the sample is in the positive category. This assumes that the Bayesian score follows a normal distribution and is different from the prediction using a cutoff.
Enrichment: An estimate of enrichment, that is, the increased likelihood (versus random) of this sample being in the category.
Bayesian Score: The standard Laplacian-modified Bayesian score.
Mahalanobis Distance: The Mahalanobis distance (MD) is the distance to the center of the training data. The larger the MD, the less trustworthy the prediction.
Mahalanobis Distance p-value: The p-value gives the fraction of training data with an MD greater than or equal to the one for the given sample, assuming normally distributed data. The smaller the p-value, the less trustworthy the prediciton. For highly non-normal X properties (e.g., fingerprints), the MD p-value is wildly inaccurate.

Structural Similar Compounds

Name	Lovastatin	Simvastatin	Felodipine
Structure	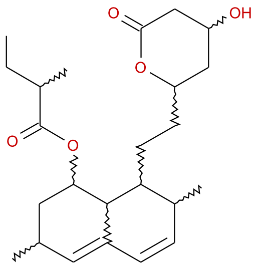	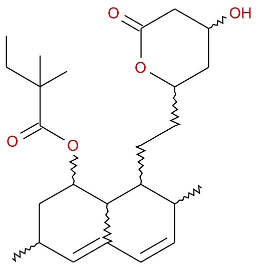	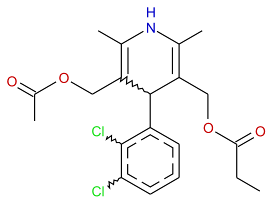
Actual Endpoint	Carcinogen	Carcinogen	Carcinogen
Predicted Endpoint	Carcinogen	Carcinogen	Carcinogen
Distance	0.568	0.571	0.602
Reference	US FDA (Centre for Drug Eval.& Res./Off. Testing & Res.) Sept. 1997	US FDA (Centre for Drug Eval.& Res./Off. Testing & Res.) Sept. 1997	US FDA (Centre for Drug Eval.& Res./Off. Testing & Res.) Sept. 1997

Model Applicability

Unknown features are fingerprint features in the query molecule, but not found in the training set.

1. All properties and OPS components are within expected ranges.

Feature Contribution

Top features for positive contribution

Fingerprint	Bit/Smiles	Feature Structure	Score	Carcinogen in training set
SCFP_6	794417578	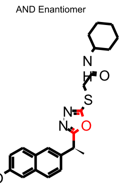 [*][c]1:[*]:[*]:[c]([*]):o:1	0.600	9 out of 14

SCFP_6	149212520	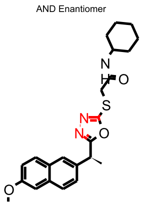 [*][c]1:[*]:[*]:n:n:1	0.543	9 out of 15
SCFP_6	-1379673609	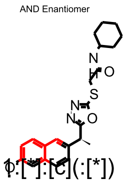 [*][c]1:[*]:[c](:[*]):[c](:[cH]:[*]):[cH]:[cH]:1	0.526	11 out of 19

Top Features for negative contribution

Fingerprint	Bit/Smiles	Feature Structure	Score	Carcinogen in training set
SCFP_6	-1530429459	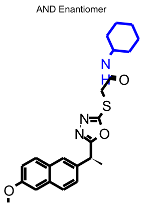 [*]NC1CCCCC1	-0.957	0 out of 5
SCFP_6	911934960	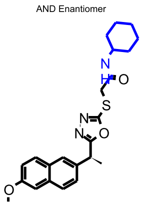 [*]C(=[*])NC1CCCCC1	-0.825	0 out of 4
SCFP_6	-576781090	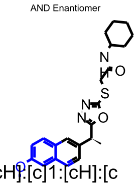 [*]:[cH]:[c]1:[cH]:[c](OC):[cH]:[cH]:[c]:1:[*]	-0.825	0 out of 4

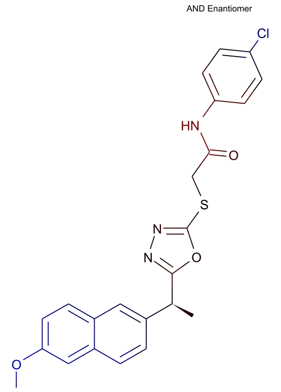

$C_{23}H_{20}ClN_3O_3S$
Molecular Weight: 453.9412
ALogP: 4.902
Rotatable Bonds: 7
Acceptors: 5
Donors: 1

Model Prediction

Prediction: Non-Carcinogen
Probability: 0.331
Enrichment: 0.991
Bayesian Score: -0.905
Mahalanobis Distance: 14.152
Mahalanobis Distance p-value: 2.34e-005

Prediction: Positive if the Bayesian score is above the estimated best cutoff value from minimizing the false positive and false negative rate.
Probability: The esimated probability that the sample is in the positive category. This assumes that the Bayesian score follows a normal distribution and is different from the prediction using a cutoff.
Enrichment: An estimate of enrichment, that is, the increased likelihood (versus random) of this sample being in the category.
Bayesian Score: The standard Laplacian-modified Bayesian score.
Mahalanobis Distance: The Mahalanobis distance (MD) is the distance to the center of the training data. The larger the MD, the less trustworthy the prediction.
Mahalanobis Distance p-value: The p-value gives the fraction of training data with an MD greater than or equal to the one for the given sample, assuming normally distributed data. The smaller the p-value, the less trustworthy the prediciton. For highly non-normal X properties (e.g., fingerprints), the MD p-value is wildly inaccurate.

Structural Similar Compounds

Name	Simvastatin	Lovastatin	Felodipine
Structure	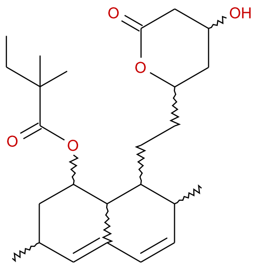	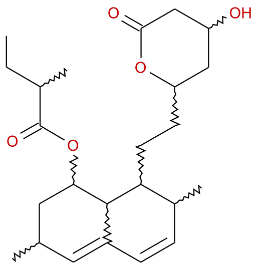	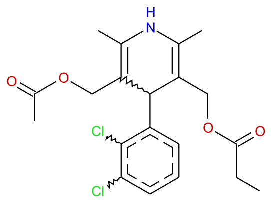
Actual Endpoint	Carcinogen	Carcinogen	Carcinogen
Predicted Endpoint	Carcinogen	Carcinogen	Carcinogen
Distance	0.591	0.611	0.616
Reference	US FDA (Centre for Drug Eval.& Res./Off. Testing & Res.) Sept. 1997	US FDA (Centre for Drug Eval.& Res./Off. Testing & Res.) Sept. 1997	US FDA (Centre for Drug Eval.& Res./Off. Testing & Res.) Sept. 1997

Model Applicability

Unknown features are fingerprint features in the query molecule, but not found in the training set.

1. All properties and OPS components are within expected ranges.

Feature Contribution

Top features for positive contribution

Fingerprint	Bit/Smiles	Feature Structure	Score	Carcinogen in training set
SCFP_6	-347048986	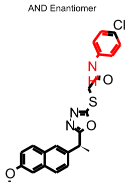 [*]C(=[*])N[c]1:[cH]:[cH]:[*]:[cH]:[cH]:1	0.615	5 out of 7

SCFP_6	814408713	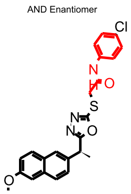<br>[*]CC(=O)N[c]1:[cH]:[<br>cH]:[cH]:[cH]:[cH]:1	0.603	2 out of 2
SCFP_6	794417578	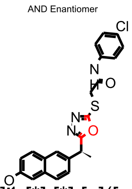<br>[*][c]1:[*]:[*]:[c]([<br>*]):o:1	0.600	9 out of 14
**Top Features for negative contribution**				
**Fingerprint**	**Bit/Smiles**	**Feature Structure**	**Score**	**Carcinogen in training set**
SCFP_6	-576781090	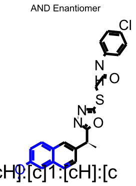<br>[*]:[cH]:[c]1:[cH]:[c<br>](OC):[cH]:[cH]:[c]:<br>1:[*]	-0.825	0 out of 4
SCFP_6	1469959550	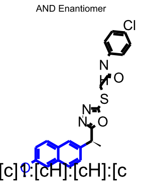<br>[*]O[c]1:[cH]:[cH]:[c<br>]2:[cH]:[c]([*]):[cH<br>]:[cH]:[c]:2:[cH]:1	-0.496	0 out of 2
SCFP_6	2029059671	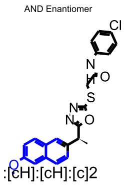<br>CO[c]1:[cH]:[cH]:[c]2<br>:[cH]:[*]:[cH]:[cH]:<br>[c]:2:[cH]:1	-0.496	0 out of 2

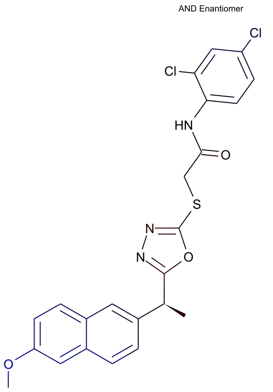

$C_{23}H_{19}Cl_2N_3O_3S$
Molecular Weight: 488.38626
ALogP: 5.567
Rotatable Bonds: 7
Acceptors: 5
Donors: 1

Model Prediction

Prediction: Non-Carcinogen
Probability: 0.303
Enrichment: 0.908
Bayesian Score: -1.981
Mahalanobis Distance: 14.152
Mahalanobis Distance p-value: 2.34e-005

Prediction: Positive if the Bayesian score is above the estimated best cutoff value from minimizing the false positive and false negative rate.
Probability: The esimated probability that the sample is in the positive category. This assumes that the Bayesian score follows a normal distribution and is different from the prediction using a cutoff.
Enrichment: An estimate of enrichment, that is, the increased likelihood (versus random) of this sample being in the category.
Bayesian Score: The standard Laplacian-modified Bayesian score.
Mahalanobis Distance: The Mahalanobis distance (MD) is the distance to the center of the training data. The larger the MD, the less trustworthy the prediction.
Mahalanobis Distance p-value: The p-value gives the fraction of training data with an MD greater than or equal to the one for the given sample, assuming normally distributed data. The smaller the p-value, the less trustworthy the prediciton. For highly non-normal X properties (e.g., fingerprints), the MD p-value is wildly inaccurate.

Structural Similar Compounds

Name	Emetine	Simvastatin	Astemizole
Structure	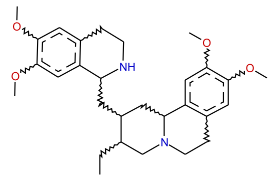	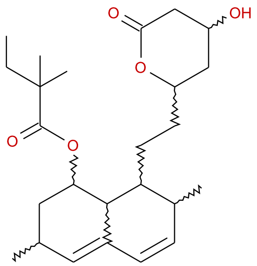	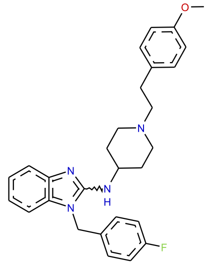
Actual Endpoint	Non-Carcinogen	Carcinogen	Non-Carcinogen
Predicted Endpoint	Non-Carcinogen	Carcinogen	Non-Carcinogen
Distance	0.645	0.648	0.660
Reference	US FDA (Centre for Drug Eval.& Res./Off. Testing & Res.) Sept. 1997	US FDA (Centre for Drug Eval.& Res./Off. Testing & Res.) Sept. 1997	US FDA (Centre for Drug Eval.& Res./Off. Testing & Res.) Sept. 1997

Model Applicability

Unknown features are fingerprint features in the query molecule, but not found in the training set.

1. All properties and OPS components are within expected ranges.

Feature Contribution

Top features for positive contribution				
**Fingerprint**	**Bit/Smiles**	**Feature Structure**	**Score**	**Carcinogen in training set**
SCFP_6	794417578	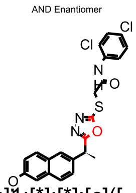 [*][c]1:[*]:[*]:[c]([*]):o:1	0.600	9 out of 14

SCFP_6	149212520	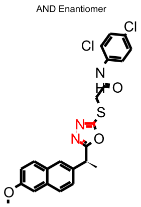 [*][c]1:[*]:[*]:n:n:1	0.543	9 out of 15
SCFP_6	-1379673609	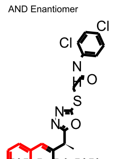 [*][c]1:[*]:[*]:[c](:[*]):[c](:[cH]:[*]):[cH]:[cH]:1	0.526	11 out of 19

Top Features for negative contribution

Fingerprint	Bit/Smiles	Feature Structure	Score	Carcinogen in training set
SCFP_6	-576781090	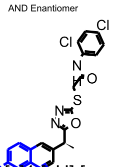 [*]:[cH]:[c]1:[cH]:[c](OC):[cH]:[cH]:[c]:1:[*]	-0.825	0 out of 4
SCFP_6	2029059671	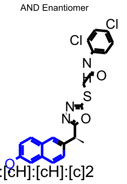 CO[c]1:[cH]:[cH]:[c]2:[cH]:[*]:[cH]:[cH]:[c]:2:[cH]:1	-0.496	0 out of 2
SCFP_6	-345817764	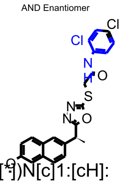 [*]C(=[*])N[c]1:[cH]:[cH]:[*]:[cH]:[c]:1Cl	-0.496	0 out of 2

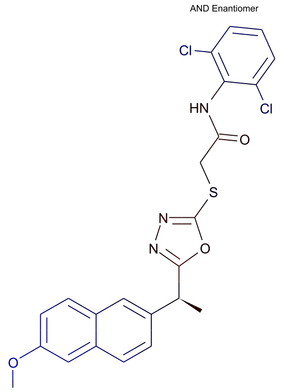

$C_{23}H_{19}Cl_2N_3O_3S$
Molecular Weight: 488.38626
ALogP: 5.567
Rotatable Bonds: 7
Acceptors: 5
Donors: 1

Model Prediction

Prediction: Non-Carcinogen
Probability: 0.285
Enrichment: 0.853
Bayesian Score: -2.758
Mahalanobis Distance: 16.140
Mahalanobis Distance p-value: 2.61e-009

Prediction: Positive if the Bayesian score is above the estimated best cutoff value from minimizing the false positive and false negative rate.
Probability: The esimated probability that the sample is in the positive category. This assumes that the Bayesian score follows a normal distribution and is different from the prediction using a cutoff.
Enrichment: An estimate of enrichment, that is, the increased likelihood (versus random) of this sample being in the category.
Bayesian Score: The standard Laplacian-modified Bayesian score.
Mahalanobis Distance: The Mahalanobis distance (MD) is the distance to the center of the training data. The larger the MD, the less trustworthy the prediction.
Mahalanobis Distance p-value: The p-value gives the fraction of training data with an MD greater than or equal to the one for the given sample, assuming normally distributed data. The smaller the p-value, the less trustworthy the prediciton. For highly non-normal X properties (e.g., fingerprints), the MD p-value is wildly inaccurate.

Structural Similar Compounds

Name	Emetine	Simvastatin	Astemizole
Structure	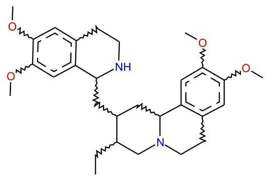	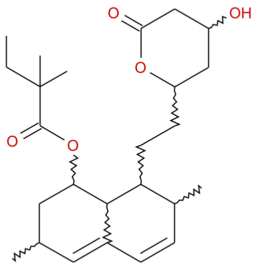	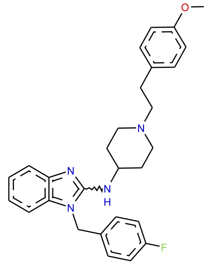
Actual Endpoint	Non-Carcinogen	Carcinogen	Non-Carcinogen
Predicted Endpoint	Non-Carcinogen	Carcinogen	Non-Carcinogen
Distance	0.644	0.647	0.655
Reference	US FDA (Centre for Drug Eval.& Res./Off. Testing & Res.) Sept. 1997	US FDA (Centre for Drug Eval.& Res./Off. Testing & Res.) Sept. 1997	US FDA (Centre for Drug Eval.& Res./Off. Testing & Res.) Sept. 1997

Model Applicability

Unknown features are fingerprint features in the query molecule, but not found in the training set.

1. All properties and OPS components are within expected ranges.

Feature Contribution

Top features for positive contribution

Fingerprint	Bit/Smiles	Feature Structure	Score	Carcinogen in training set
SCFP_6	794417578	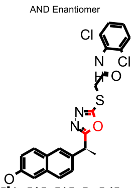 [*][c]1:[*]:[*]:[c]([*]):o:1	0.600	9 out of 14

SCFP_6	149212520	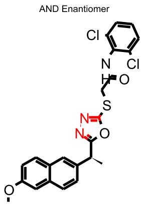 [*][c]1:[*]:[*]:n:n:1	0.543	9 out of 15
SCFP_6	-1379673609	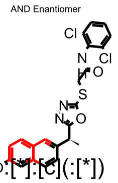 [*][c]1:[*]:[c](:[*]):[c](:[cH]:[*]):[cH]:[cH]:1	0.526	11 out of 19
**Top Features for negative contribution**				
**Fingerprint**	**Bit/Smiles**	**Feature Structure**	**Score**	**Carcinogen in training set**
SCFP_6	1062412764	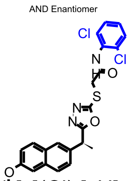 [*][c]1:[c](Cl):[cH]:[cH]:[cH]:[c]:1Cl	-0.957	0 out of 5
SCFP_6	-576781090	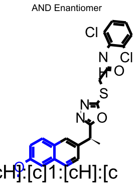 [*]:[cH]:[c]1:[cH]:[c](OC):[cH]:[cH]:[c]:1:[*]	-0.825	0 out of 4
SCFP_6	1469959550	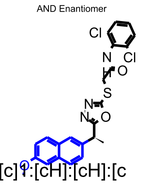 [*]O[c]1:[cH]:[cH]:[c]2:[cH]:[c]([*]):[cH]:[cH]:[c]:2:[cH]:1	-0.496	0 out of 2

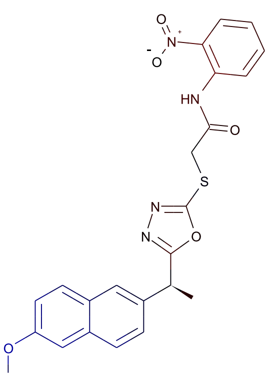

$C_{23}H_{20}N_4O_5S$
Molecular Weight: 464.4937
ALogP: 4.132
Rotatable Bonds: 8
Acceptors: 7
Donors: 1

Model Prediction

Prediction: Carcinogen
Probability: 0.385
Enrichment: 1.153
Bayesian Score: 0.975
Mahalanobis Distance: 19.265
Mahalanobis Distance p-value: 7.69e-017

Prediction: Positive if the Bayesian score is above the estimated best cutoff value from minimizing the false positive and false negative rate.
Probability: The esimated probability that the sample is in the positive category. This assumes that the Bayesian score follows a normal distribution and is different from the prediction using a cutoff.
Enrichment: An estimate of enrichment, that is, the increased likelihood (versus random) of this sample being in the category.
Bayesian Score: The standard Laplacian-modified Bayesian score.
Mahalanobis Distance: The Mahalanobis distance (MD) is the distance to the center of the training data. The larger the MD, the less trustworthy the prediction.
Mahalanobis Distance p-value: The p-value gives the fraction of training data with an MD greater than or equal to the one for the given sample, assuming normally distributed data. The smaller the p-value, the less trustworthy the prediciton. For highly non-normal X properties (e.g., fingerprints), the MD p-value is wildly inaccurate.

Structural Similar Compounds

Name	Carbenicillin	Nisoldipine	Moricizine
Structure	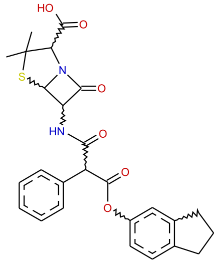	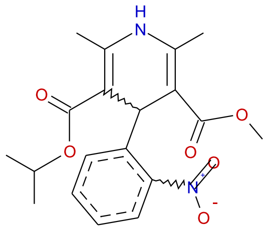	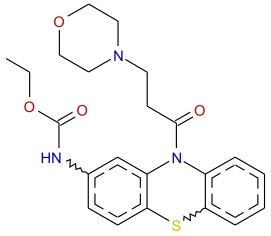
Actual Endpoint	Non-Carcinogen	Non-Carcinogen	Carcinogen
Predicted Endpoint	Non-Carcinogen	Carcinogen	Carcinogen
Distance	0.615	0.663	0.668
Reference	US FDA (Centre for Drug Eval.& Res./Off. Testing & Res.) Sept. 1997	US FDA (Centre for Drug Eval.& Res./Off. Testing & Res.) Sept. 1997	US FDA (Centre for Drug Eval.& Res./Off. Testing & Res.) Sept. 1997

Model Applicability

Unknown features are fingerprint features in the query molecule, but not found in the training set.

1. All properties and OPS components are within expected ranges.

Feature Contribution

Top features for positive contribution				
**Fingerprint**	**Bit/Smiles**	**Feature Structure**	**Score**	**Carcinogen in training set**
SCFP_6	794417578	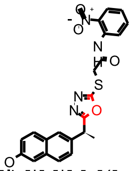 AND Enantiomer [*][c]1:[*]:[*]:[c]([*]):o:1	0.600	9 out of 14

SCFP_6	149212520	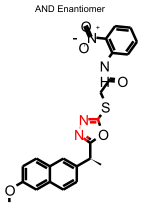 [*][c]1:[*]:[*]:n:n:1	0.543	9 out of 15
SCFP_6	-1379673609	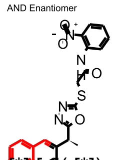 [*][c]1:[*]:[c](:[*]):[c](:[cH]:[*]):[cH]:[cH]:1	0.526	11 out of 19

Top Features for negative contribution

Fingerprint	Bit/Smiles	Feature Structure	Score	Carcinogen in training set
SCFP_6	-576781090	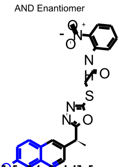 [*]:[cH]:[c]1:[cH]:[c](OC):[cH]:[cH]:[c]:1:[*]	-0.825	0 out of 4
SCFP_6	921696937	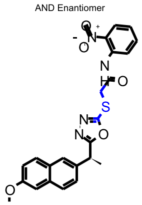 [*]CS[c](:[*]):[*]	-0.496	0 out of 2
SCFP_6	2029059671	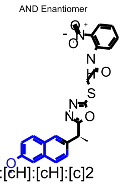 CO[c]1:[cH]:[cH]:[c]2:[cH]:[*]:[cH]:[cH]:[c]:2:[cH]:1	-0.496	0 out of 2

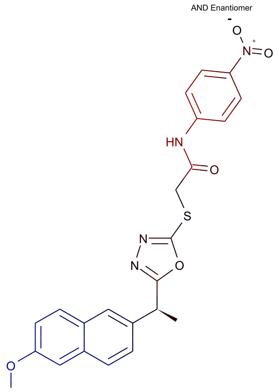

$C_{23}H_{20}N_4O_5S$
Molecular Weight: 464.4937
ALogP: 4.132
Rotatable Bonds: 8
Acceptors: 7
Donors: 1

Model Prediction

Prediction: Carcinogen
Probability: 0.424
Enrichment: 1.268
Bayesian Score: 2.170
Mahalanobis Distance: 19.060
Mahalanobis Distance p-value: 2.58e-016

Prediction: Positive if the Bayesian score is above the estimated best cutoff value from minimizing the false positive and false negative rate.
Probability: The esimated probability that the sample is in the positive category. This assumes that the Bayesian score follows a normal distribution and is different from the prediction using a cutoff.
Enrichment: An estimate of enrichment, that is, the increased likelihood (versus random) of this sample being in the category.
Bayesian Score: The standard Laplacian-modified Bayesian score.
Mahalanobis Distance: The Mahalanobis distance (MD) is the distance to the center of the training data. The larger the MD, the less trustworthy the prediction.
Mahalanobis Distance p-value: The p-value gives the fraction of training data with an MD greater than or equal to the one for the given sample, assuming normally distributed data. The smaller the p-value, the less trustworthy the prediciton. For highly non-normal X properties (e.g., fingerprints), the MD p-value is wildly inaccurate.

Structural Similar Compounds

Name	Carbenicillin	Moricizine	Nisoldipine
Structure	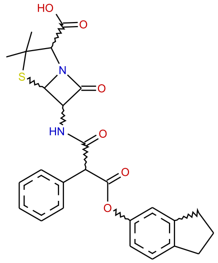	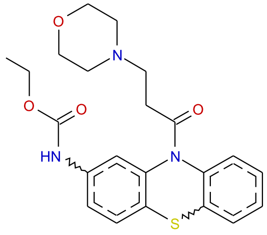	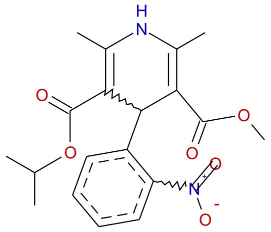
Actual Endpoint	Non-Carcinogen	Carcinogen	Non-Carcinogen
Predicted Endpoint	Non-Carcinogen	Carcinogen	Carcinogen
Distance	0.618	0.671	0.672
Reference	US FDA (Centre for Drug Eval.& Res./Off. Testing & Res.) Sept. 1997	US FDA (Centre for Drug Eval.& Res./Off. Testing & Res.) Sept. 1997	US FDA (Centre for Drug Eval.& Res./Off. Testing & Res.) Sept. 1997

Model Applicability

Unknown features are fingerprint features in the query molecule, but not found in the training set.

1. All properties and OPS components are within expected ranges.

Feature Contribution

Top features for positive contribution

Fingerprint	Bit/Smiles	Feature Structure	Score	Carcinogen in training set
SCFP_6	-347048986	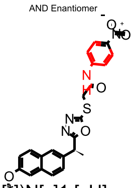 [*]C(=[*])N[c]1:[cH]:[cH]:[*]:[cH]:[cH]:1	0.615	5 out of 7

SCFP_6	814408713	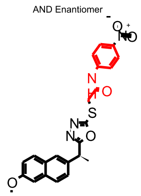 [*]CC(=O)N[c]1:[cH]:[cH]:[cH]:[cH]:[cH]:1	0.603	2 out of 2
SCFP_6	794417578	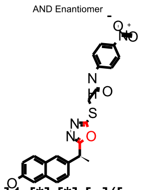 [*][c]1:[*]:[*]:[c]([*]):o:1	0.600	9 out of 14
**Top Features for negative contribution**				
**Fingerprint**	**Bit/Smiles**	**Feature Structure**	**Score**	**Carcinogen in training set**
SCFP_6	-576781090	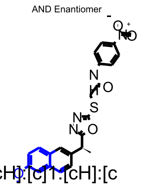 [*]:[cH]:[c]1:[cH]:[c](OC):[cH]:[cH]:[c]:1:[*]	-0.825	0 out of 4
SCFP_6	-1889730273	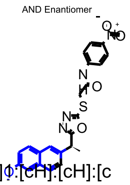 [*]O[c]1:[cH]:[cH]:[c]2:[cH]:[c]([*]):[*]:[cH]:[c]:2:[cH]:1	-0.496	0 out of 2
SCFP_6	1469959550	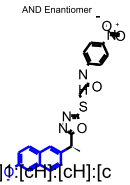 [*]O[c]1:[cH]:[cH]:[c]2:[cH]:[c]([*]):[cH]:[cH]:[c]:2:[cH]:1	-0.496	0 out of 2

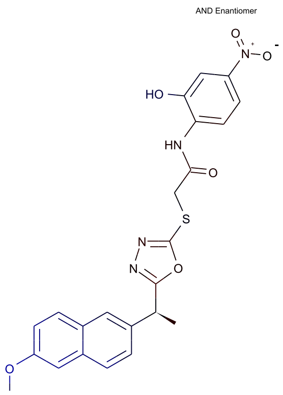

$C_{23}H_{20}N_4O_6S$
Molecular Weight: 480.4931
ALogP: 3.89
Rotatable Bonds: 8
Acceptors: 8
Donors: 2

Model Prediction

Prediction: Non-Carcinogen
Probability: 0.322
Enrichment: 0.963
Bayesian Score: -1.260
Mahalanobis Distance: 20.089
Mahalanobis Distance p-value: 5.38e-019

Prediction: Positive if the Bayesian score is above the estimated best cutoff value from minimizing the false positive and false negative rate.
Probability: The esimated probability that the sample is in the positive category. This assumes that the Bayesian score follows a normal distribution and is different from the prediction using a cutoff.
Enrichment: An estimate of enrichment, that is, the increased likelihood (versus random) of this sample being in the category.
Bayesian Score: The standard Laplacian-modified Bayesian score.
Mahalanobis Distance: The Mahalanobis distance (MD) is the distance to the center of the training data. The larger the MD, the less trustworthy the prediction.
Mahalanobis Distance p-value: The p-value gives the fraction of training data with an MD greater than or equal to the one for the given sample, assuming normally distributed data. The smaller the p-value, the less trustworthy the prediciton. For highly non-normal X properties (e.g., fingerprints), the MD p-value is wildly inaccurate.

Structural Similar Compounds

Name	Carbenicillin	Sulfasalazine	Nimodipine
Structure	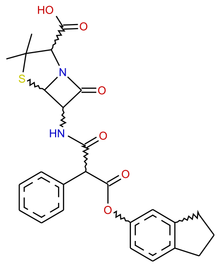	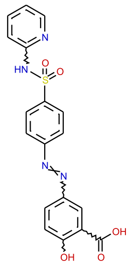	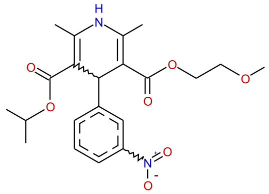
Actual Endpoint	Non-Carcinogen	Carcinogen	Carcinogen
Predicted Endpoint	Non-Carcinogen	Carcinogen	Carcinogen
Distance	0.588	0.703	0.726
Reference	US FDA (Centre for Drug Eval.& Res./Off. Testing & Res.) Sept. 1997	US FDA (Centre for Drug Eval.& Res./Off. Testing & Res.) Sept. 1997	US FDA (Centre for Drug Eval.& Res./Off. Testing & Res.) Sept. 1997

Model Applicability

Unknown features are fingerprint features in the query molecule, but not found in the training set.

1. All properties and OPS components are within expected ranges.

Feature Contribution

Top features for positive contribution				
**Fingerprint**	**Bit/Smiles**	**Feature Structure**	**Score**	**Carcinogen in training set**
SCFP_6	794417578	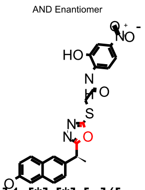 [*][c]1:[*]:[*]:[c]([*]):o:1	0.600	9 out of 14

SCFP_6	149212520	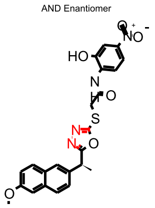 [*][c]1:[*]:[*]:n:n:1	0.543	9 out of 15
SCFP_6	-1379673609	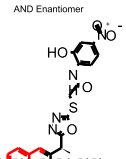 [*][c]1:[*]:[c](:[*]):[c](:[cH]:[*]):[cH]:[cH]:1	0.526	11 out of 19
**Top Features for negative contribution**				
**Fingerprint**	**Bit/Smiles**	**Feature Structure**	**Score**	**Carcinogen in training set**
SCFP_6	-576781090	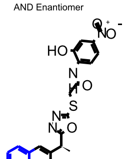 [*]:[cH]:[c]1:[cH]:[c](OC):[cH]:[cH]:[c]:1:[*]	-0.825	0 out of 4
SCFP_6	2029059671	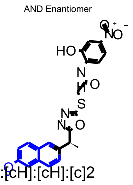 CO[c]1:[cH]:[cH]:[c]2:[cH]:[*]:[cH]:[cH]:[c]:2:[cH]:1	-0.496	0 out of 2
SCFP_6	124239044	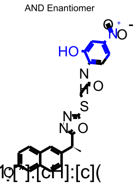 [*][c]1:[*]:[cH]:[c](:[cH]:[c]:1O)[N+](=[*])[*]	-0.496	0 out of 2

Sorafenib

TOPKAT_Rat_Male_FDA_None_vs_Carcinogen

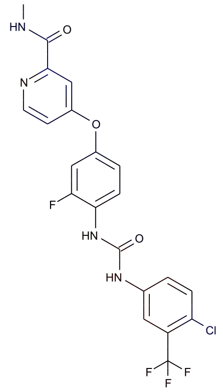

$C_{21}H_{15}ClF_4N_4O_3$

Molecular Weight: 482.81541

ALogP: 4.381

Rotatable Bonds: 6

Acceptors: 4

Donors: 3

Model Prediction

Prediction: Non-Carcinogen

Probability: 0.299

Enrichment: 0.894

Bayesian Score: -2.178

Mahalanobis Distance: 17.633

Mahalanobis Distance p-value: 9.36e-013

Prediction: Positive if the Bayesian score is above the estimated best cutoff value from minimizing the false positive and false negative rate.
Probability: The esimated probability that the sample is in the positive category. This assumes that the Bayesian score follows a normal distribution and is different from the prediction using a cutoff.
Enrichment: An estimate of enrichment, that is, the increased likelihood (versus random) of this sample being in the category.
Bayesian Score: The standard Laplacian-modified Bayesian score.
Mahalanobis Distance: The Mahalanobis distance (MD) is the distance to the center of the training data. The larger the MD, the less trustworthy the prediction.
Mahalanobis Distance p-value: The p-value gives the fraction of training data with an MD greater than or equal to the one for the given sample, assuming normally distributed data. The smaller the p-value, the less trustworthy the prediciton. For highly non-normal X properties (e.g., fingerprints), the MD p-value is wildly inaccurate.

Structural Similar Compounds

Name	Glyburide	Glimepride	Fluvastatin
Structure	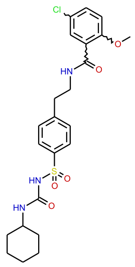	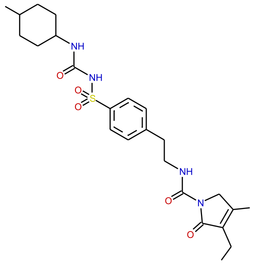	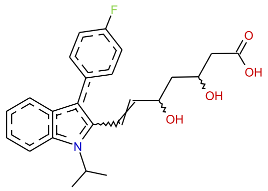
Actual Endpoint	Non-Carcinogen	Non-Carcinogen	Carcinogen
Predicted Endpoint	Non-Carcinogen	Non-Carcinogen	Carcinogen
Distance	0.594	0.605	0.629
Reference	US FDA (Centre for Drug Eval.& Res./Off. Testing & Res.) Sept. 1997	US FDA (Centre for Drug Eval.& Res./Off. Testing & Res.) Sept. 1997	US FDA (Centre for Drug Eval.& Res./Off. Testing & Res.) Sept. 1997

Model Applicability

Unknown features are fingerprint features in the query molecule, but not found in the training set.

1. All properties and OPS components are within expected ranges.

Feature Contribution

Top features for positive contribution

Fingerprint	Bit/Smiles	Feature Structure	Score	Carcinogen in training set
SCFP_6	-347048986	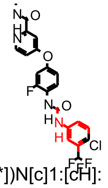 [*]C(=[*])N[c]1:[cH]:[cH]:[*]:[cH]:[cH]:1	0.615	5 out of 7

SCFP_6	-754059116	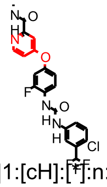 [*]O[c]1:[cH]:[*]:n:[cH]:[cH]:1	0.415	1 out of 1
SCFP_6	-347281112	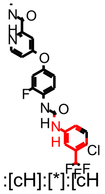 [*]N[c]1:[cH]:[*]:[cH]:[c](C):[cH]:1	0.273	2 out of 4

Top Features for negative contribution

Fingerprint	Bit/Smiles	Feature Structure	Score	Carcinogen in training set
SCFP_6	-827073191	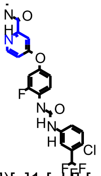 [*]C(=[*])[c]1:[cH]:[*]:[cH]:[cH]:n:1	-0.674	0 out of 3
SCFP_6	-488587948	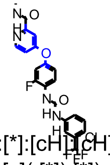 [*][c]1:[*]:[cH]:[cH]:[c](O[c](:[*]):[*]):[cH]:1	-0.496	0 out of 2
SCFP_6	1257084377	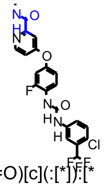 [*]NC(=O)[c](:[*]):[*]	-0.436	4 out of 21

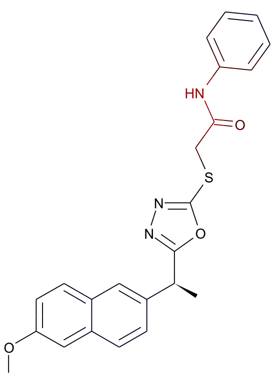

$C_{23}H_{21}N_3O_3S$
Molecular Weight: 419.49614
ALogP: 4.238
Rotatable Bonds: 7
Acceptors: 5
Donors: 1

Model Prediction

Prediction: Multiple-Carcinogen
Probability: 0.568
Enrichment: 1.372
Bayesian Score: 0.762
Mahalanobis Distance: 16.953
Mahalanobis Distance p-value: 1.75e-007

Prediction: Positive if the Bayesian score is above the estimated best cutoff value from minimizing the false positive and false negative rate.
Probability: The esimated probability that the sample is in the positive category. This assumes that the Bayesian score follows a normal distribution and is different from the prediction using a cutoff.
Enrichment: An estimate of enrichment, that is, the increased likelihood (versus random) of this sample being in the category.
Bayesian Score: The standard Laplacian-modified Bayesian score.
Mahalanobis Distance: The Mahalanobis distance (MD) is the distance to the center of the training data. The larger the MD, the less trustworthy the prediction.
Mahalanobis Distance p-value: The p-value gives the fraction of training data with an MD greater than or equal to the one for the given sample, assuming normally distributed data. The smaller the p-value, the less trustworthy the prediciton. For highly non-normal X properties (e.g., fingerprints), the MD p-value is wildly inaccurate.

Structural Similar Compounds

Name	Lovastatin	Simvastatin	Felodipine
Structure	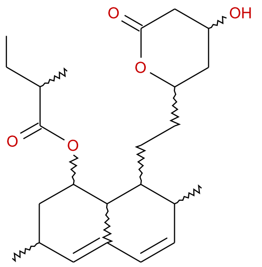	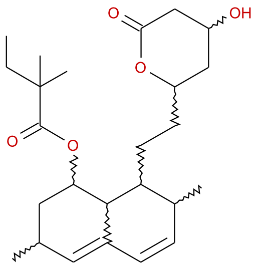	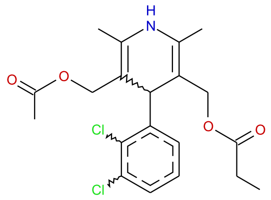
Actual Endpoint	Single-Carcinogen	Multiple-Carcinogen	Single-Carcinogen
Predicted Endpoint	Single-Carcinogen	Multiple-Carcinogen	Single-Carcinogen
Distance	0.573	0.590	0.599
Reference	US FDA (Centre for Drug Eval.& Res./Off. Testing & Res.) Sept. 1997	US FDA (Centre for Drug Eval.& Res./Off. Testing & Res.) Sept. 1997	US FDA (Centre for Drug Eval.& Res./Off. Testing & Res.) Sept. 1997

Model Applicability

Unknown features are fingerprint features in the query molecule, but not found in the training set.

1. All properties and OPS components are within expected ranges.

Feature Contribution

Top features for positive contribution

Fingerprint	Bit/Smiles	Feature Structure	Score	Multiple-Carcinogen in training set
SCFP_8	2097618059	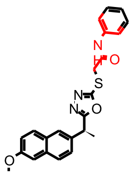 AND Enantiomer [*]CC(=O)N[c](:[cH]:[*]):[cH]:[*]	0.681	6 out of 7

SCFP_8	-347048986	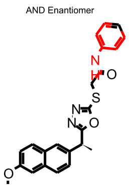 [*]C(=[*])N[c]1:[cH]:[cH]:[*]:[cH]:[cH]:1	0.574	4 out of 5
SCFP_8	814408713	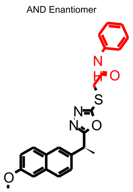 [*]CC(=O)N[c]1:[cH]:[cH]:[cH]:[cH]:[cH]:1	0.553	2 out of 2
**Top Features for negative contribution**				
**Fingerprint**	**Bit/Smiles**	**Feature Structure**	**Score**	**Multiple-Carcinogen in training set**
SCFP_8	1653911926	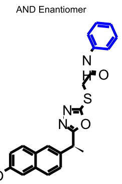 [*][c]1:[cH]:[cH]:[cH]:[cH]:[cH]:1	-0.985	1 out of 12
SCFP_8	136239834	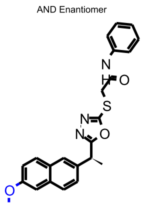 [*]OC	-0.358	3 out of 13
SCFP_8	-1186786545	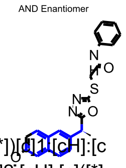 [*]C([*])[c]1:[cH]:[cH]:[c]2:[cH]:[c]([*]):[*]:[cH]:[c]:2:[cH]:1	-0.310	0 out of 1

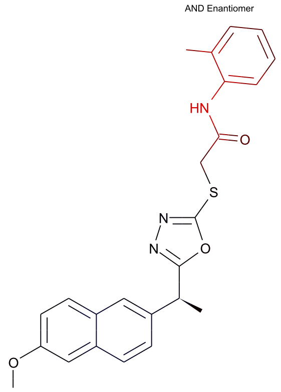

$C_{24}H_{23}N_3O_3S$
Molecular Weight: 433.52272
ALogP: 4.724
Rotatable Bonds: 7
Acceptors: 5
Donors: 1

Structural Similar Compounds

Name	Simvastatin	Lovastatin	Felodipine
Structure	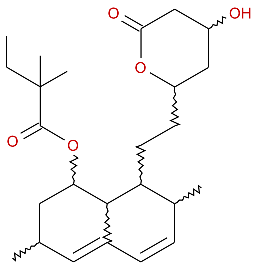	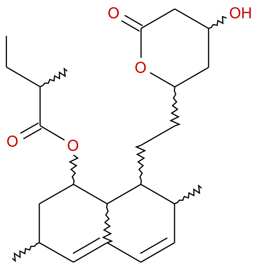	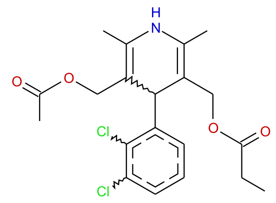
Actual Endpoint	Multiple-Carcinogen	Single-Carcinogen	Single-Carcinogen
Predicted Endpoint	Multiple-Carcinogen	Single-Carcinogen	Single-Carcinogen
Distance	0.573	0.586	0.621
Reference	US FDA (Centre for Drug Eval.& Res./Off. Testing & Res.) Sept. 1997	US FDA (Centre for Drug Eval.& Res./Off. Testing & Res.) Sept. 1997	US FDA (Centre for Drug Eval.& Res./Off. Testing & Res.) Sept. 1997

Model Prediction

Prediction: Multiple-Carcinogen
Probability: 0.558
Enrichment: 1.347
Bayesian Score: 4.046
Mahalanobis Distance: 17.078
Mahalanobis Distance p-value: 1.34e-007

Prediction: Positive if the Bayesian score is above the estimated best cutoff value from minimizing the false positive and false negative rate.
Probability: The esimated probability that the sample is in the positive category. This assumes that the Bayesian score follows a normal distribution and is different from the prediction using a cutoff.
Enrichment: An estimate of enrichment, that is, the increased likelihood (versus random) of this sample being in the category.
Bayesian Score: The standard Laplacian-modified Bayesian score.
Mahalanobis Distance: The Mahalanobis distance (MD) is the distance to the center of the training data. The larger the MD, the less trustworthy the prediction.
Mahalanobis Distance p-value: The p-value gives the fraction of training data with an MD greater than or equal to the one for the given sample, assuming normally distributed data. The smaller the p-value, the less trustworthy the prediciton. For highly non-normal X properties (e.g., fingerprints), the MD p-value is wildly inaccurate.

Model Applicability

Unknown features are fingerprint features in the query molecule, but not found in the training set.

1. All properties and OPS components are within expected ranges.

Feature Contribution

Top features for positive contribution				
**Fingerprint**	**Bit/Smiles**	**Feature Structure**	**Score**	**Multiple-Carcinogen in training set**
SCFP_8	2097618059	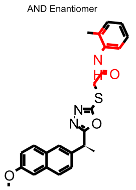 [*]CC(=O)N[c](:[cH]:[*]):[cH]:[*]	0.681	6 out of 7

SCFP_8	1792377291	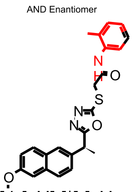 [*]N[c]1:[cH]:[*]:[cH]:[cH]:[c]:1C	0.553	2 out of 2
SCFP_8	1269778311	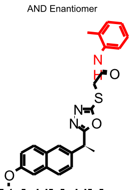 [*]N[c]1:[cH]:[cH]:[c H]:[cH]:[c]:1C	0.553	2 out of 2
**Top Features for negative contribution**				
**Fingerprint**	**Bit/Smiles**	**Feature Structure**	**Score**	**Multiple-Carcinogen in training set**
SCFP_8	136239834	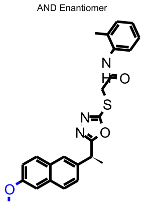 [*]OC	-0.358	3 out of 13
SCFP_8	470284964	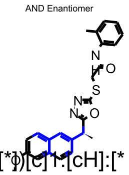 [*]C([*])[c]1:[cH]:[*]:[c](:[*]):[c](:[cH]:[*]):[cH]:1	-0.310	0 out of 1
SCFP_8	-699162599	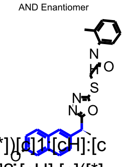 [*]C([*])[c]1:[cH]:[c H]:[c]2:[cH]:[c]([*]):[cH]:[cH]:[c]:2:[c H]:1	-0.310	0 out of 1

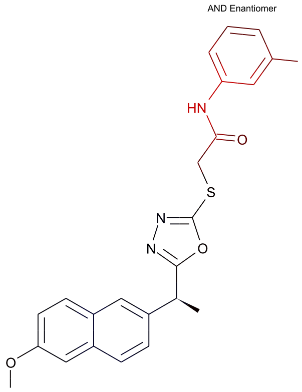

$C_{24}H_{23}N_3O_3S$
Molecular Weight: 433.52272
ALogP: 4.724
Rotatable Bonds: 7
Acceptors: 5
Donors: 1

Model Prediction

Prediction: Multiple-Carcinogen
Probability: 0.565
Enrichment: 1.365
Bayesian Score: 4.337
Mahalanobis Distance: 16.122
Mahalanobis Distance p-value: 1.02e-006

Prediction: Positive if the Bayesian score is above the estimated best cutoff value from minimizing the false positive and false negative rate.
Probability: The esimated probability that the sample is in the positive category. This assumes that the Bayesian score follows a normal distribution and is different from the prediction using a cutoff.
Enrichment: An estimate of enrichment, that is, the increased likelihood (versus random) of this sample being in the category.
Bayesian Score: The standard Laplacian-modified Bayesian score.
Mahalanobis Distance: The Mahalanobis distance (MD) is the distance to the center of the training data. The larger the MD, the less trustworthy the prediction.
Mahalanobis Distance p-value: The p-value gives the fraction of training data with an MD greater than or equal to the one for the given sample, assuming normally distributed data. The smaller the p-value, the less trustworthy the prediciton. For highly non-normal X properties (e.g., fingerprints), the MD p-value is wildly inaccurate.

Structural Similar Compounds

Name	Simvastatin	Lovastatin	Felodipine
Structure	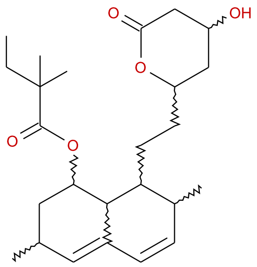	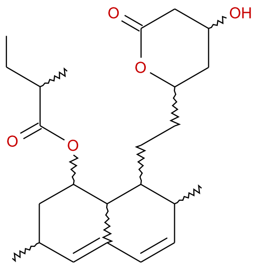	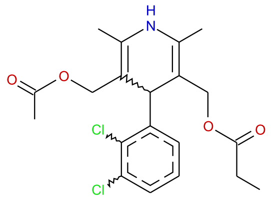
Actual Endpoint	Multiple-Carcinogen	Single-Carcinogen	Single-Carcinogen
Predicted Endpoint	Multiple-Carcinogen	Single-Carcinogen	Single-Carcinogen
Distance	0.573	0.586	0.621
Reference	US FDA (Centre for Drug Eval.& Res./Off. Testing & Res.) Sept. 1997	US FDA (Centre for Drug Eval.& Res./Off. Testing & Res.) Sept. 1997	US FDA (Centre for Drug Eval.& Res./Off. Testing & Res.) Sept. 1997

Model Applicability

Unknown features are fingerprint features in the query molecule, but not found in the training set.

1. All properties and OPS components are within expected ranges.

Feature Contribution

Top features for positive contribution

Fingerprint	Bit/Smiles	Feature Structure	Score	Multiple-Carcinogen in training set
SCFP_8	2097618059	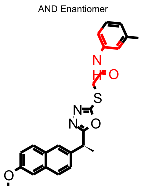 [*]CC(=O)N[c](:[cH]:[*]):[cH]:[*]	0.681	6 out of 7

SCFP_8	-347048986	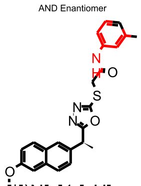 [*]C(=[*])N[c]1:[cH]:[cH]:[*]:[cH]:[cH]:1	0.574	4 out of 5
SCFP_8	-236487363	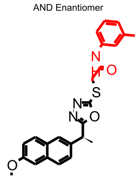 [*]CC(=O)N[c]1:[cH]:[cH]:[cH]:[c](C):[cH]:1	0.553	2 out of 2
**Top Features for negative contribution**				
**Fingerprint**	**Bit/Smiles**	**Feature Structure**	**Score**	**Multiple-Carcinogen in training set**
SCFP_8	136239834	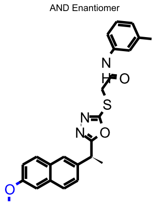 [*]OC	-0.358	3 out of 13
SCFP_8	-699162599	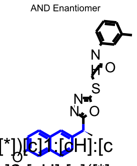 [*]C([*])[c]1:[cH]:[cH]:[c]2:[cH]:[c]([*]):[cH]:[cH]:[c]:2:[cH]:1	-0.310	0 out of 1
SCFP_8	470284964	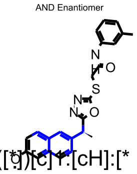 [*]C([*])[c]1:[cH]:[*]:[c](:[*]):[c](:[cH]:[*]):[cH]:1	-0.310	0 out of 1

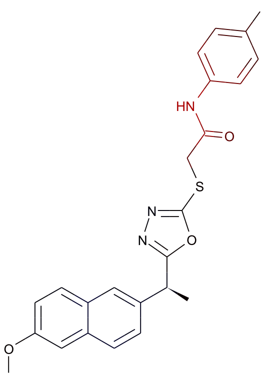

$C_{24}H_{23}N_{3}O_{3}S$
Molecular Weight: 433.52272
ALogP: 4.724
Rotatable Bonds: 7
Acceptors: 5
Donors: 1

Model Prediction

Prediction: Multiple-Carcinogen
Probability: 0.558
Enrichment: 1.347
Bayesian Score: 3.366
Mahalanobis Distance: 15.949
Mahalanobis Distance p-value: 1.47e-006

Prediction: Positive if the Bayesian score is above the estimated best cutoff value from minimizing the false positive and false negative rate.
Probability: The esimated probability that the sample is in the positive category. This assumes that the Bayesian score follows a normal distribution and is different from the prediction using a cutoff.
Enrichment: An estimate of enrichment, that is, the increased likelihood (versus random) of this sample being in the category.
Bayesian Score: The standard Laplacian-modified Bayesian score.
Mahalanobis Distance: The Mahalanobis distance (MD) is the distance to the center of the training data. The larger the MD, the less trustworthy the prediction.
Mahalanobis Distance p-value: The p-value gives the fraction of training data with an MD greater than or equal to the one for the given sample, assuming normally distributed data. The smaller the p-value, the less trustworthy the prediciton. For highly non-normal X properties (e.g., fingerprints), the MD p-value is wildly inaccurate.

Structural Similar Compounds

Name	Simvastatin	Lovastatin	Felodipine
Structure	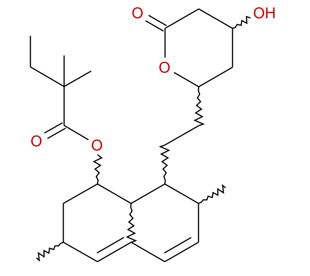	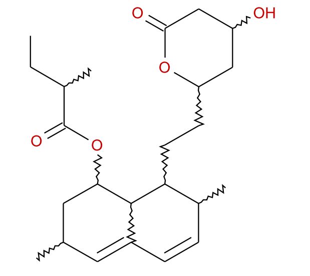	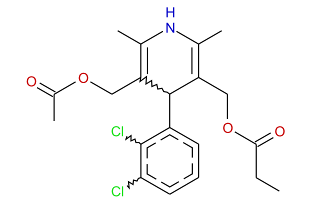
Actual Endpoint	Multiple-Carcinogen	Single-Carcinogen	Single-Carcinogen
Predicted Endpoint	Multiple-Carcinogen	Single-Carcinogen	Single-Carcinogen
Distance	0.573	0.585	0.624
Reference	US FDA (Centre for Drug Eval.& Res./Off. Testing & Res.) Sept. 1997	US FDA (Centre for Drug Eval.& Res./Off. Testing & Res.) Sept. 1997	US FDA (Centre for Drug Eval.& Res./Off. Testing & Res.) Sept. 1997

Model Applicability

Unknown features are fingerprint features in the query molecule, but not found in the training set.

1. All properties and OPS components are within expected ranges.

Feature Contribution

Top features for positive contribution

Fingerprint	Bit/Smiles	Feature Structure	Score	Multiple-Carcinogen in training set
SCFP_8	2097618059	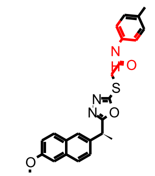 AND Enantiomer [*]CC(=O)N[c](:[cH]:[*]):[cH]:[*]	0.681	6 out of 7

SCFP_8	-347048986	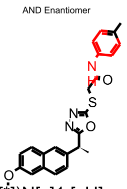<br>[*]C(=[*])N[c]1:[cH]:<br>[cH]:[*]:[cH]:[cH]:1	0.574	4 out of 5
SCFP_8	814408713	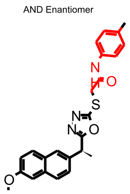<br>[*]CC(=O)N[c]1:[cH]:[<br>cH]:[cH]:[cH]:[cH]:1	0.553	2 out of 2
**Top Features for negative contribution**				
**Fingerprint**	**Bit/Smiles**	**Feature Structure**	**Score**	**Multiple-Carcinogen in training set**
SCFP_8	136239834	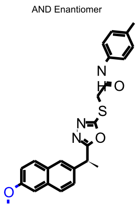<br>[*]OC	-0.358	3 out of 13
SCFP_8	470284964	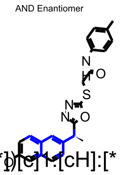<br>[*]C([*])[c]1:[cH]:[*<br>]:[c](:[*]):[c](:[cH<br>]:[*]):[cH]:1	-0.310	0 out of 1
SCFP_8	795925860	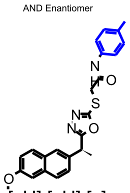<br>[*][c]1:[cH]:[cH]:[c]<br>(C):[cH]:[cH]:1	-0.310	0 out of 1

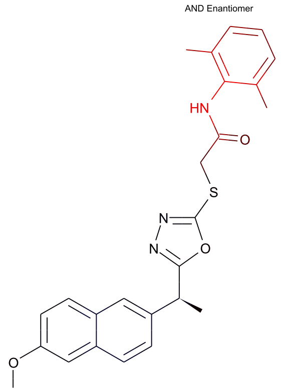

$C_{25}H_{25}N_3O_3S$
Molecular Weight: 447.5493
ALogP: 5.21
Rotatable Bonds: 7
Acceptors: 5
Donors: 1

Model Prediction

Prediction: Multiple-Carcinogen
Probability: 0.589
Enrichment: 1.422
Bayesian Score: 5.257
Mahalanobis Distance: 19.961
Mahalanobis Distance p-value: 3.29e-010

Prediction: Positive if the Bayesian score is above the estimated best cutoff value from minimizing the false positive and false negative rate.
Probability: The esimated probability that the sample is in the positive category. This assumes that the Bayesian score follows a normal distribution and is different from the prediction using a cutoff.
Enrichment: An estimate of enrichment, that is, the increased likelihood (versus random) of this sample being in the category.
Bayesian Score: The standard Laplacian-modified Bayesian score.
Mahalanobis Distance: The Mahalanobis distance (MD) is the distance to the center of the training data. The larger the MD, the less trustworthy the prediction.
Mahalanobis Distance p-value: The p-value gives the fraction of training data with an MD greater than or equal to the one for the given sample, assuming normally distributed data. The smaller the p-value, the less trustworthy the prediciton. For highly non-normal X properties (e.g., fingerprints), the MD p-value is wildly inaccurate.

Structural Similar Compounds

Name	Simvastatin	Lovastatin	Felodipine
Structure	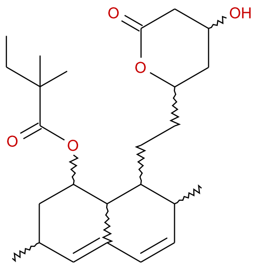	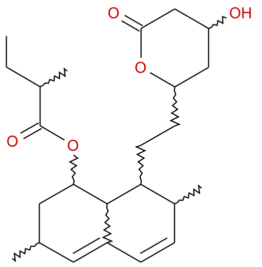	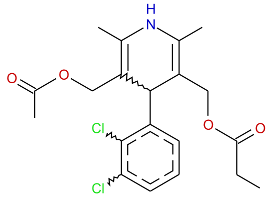
Actual Endpoint	Multiple-Carcinogen	Single-Carcinogen	Single-Carcinogen
Predicted Endpoint	Multiple-Carcinogen	Single-Carcinogen	Single-Carcinogen
Distance	0.590	0.623	0.658
Reference	US FDA (Centre for Drug Eval.& Res./Off. Testing & Res.) Sept. 1997	US FDA (Centre for Drug Eval.& Res./Off. Testing & Res.) Sept. 1997	US FDA (Centre for Drug Eval.& Res./Off. Testing & Res.) Sept. 1997

Model Applicability

Unknown features are fingerprint features in the query molecule, but not found in the training set.

1. All properties and OPS components are within expected ranges.

Feature Contribution

Top features for positive contribution

Fingerprint	Bit/Smiles	Feature Structure	Score	Multiple-Carcinogen in training set
SCFP_8	2097618059	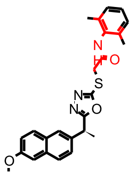 AND Enantiomer [*]CC(=O)N[c](:[cH]:[*]):[cH]:[*]	0.681	6 out of 7

SCFP_8	1792377291	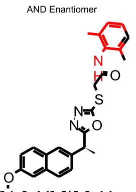 [*]N[c]1:[cH]:[*]:[cH]:[cH]:[c]:1C	0.553	2 out of 2
SCFP_8	1269778311	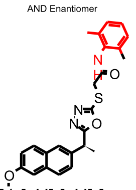 [*]N[c]1:[cH]:[cH]:[cH]:[cH]:[c]:1C	0.553	2 out of 2
**Top Features for negative contribution**				
**Fingerprint**	**Bit/Smiles**	**Feature Structure**	**Score**	**Multiple-Carcinogen in training set**
SCFP_8	136239834	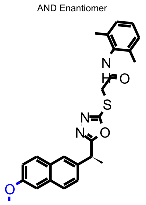 [*]OC	-0.358	3 out of 13
SCFP_8	470284964	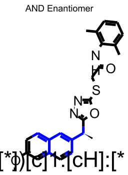 [*]C([*])[c]1:[cH]:[*]:[c](:[*]):[c](:[cH]:[*]):[cH]:1	-0.310	0 out of 1
SCFP_8	-1186786545	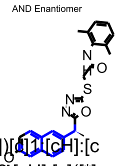 [*]C([*])[c]1:[cH]:[cH]:[c]2:[cH]:[c]([*]):[*]:[cH]:[c]:2:[cH]:1	-0.310	0 out of 1

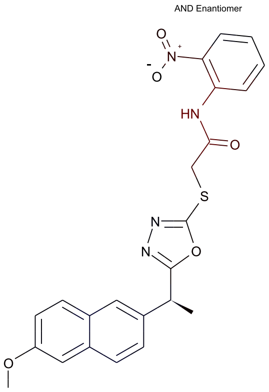

$C_{23}H_{20}N_4O_5S$
Molecular Weight: 464.4937
ALogP: 4.132
Rotatable Bonds: 8
Acceptors: 7
Donors: 1

Model Prediction

Prediction: Multiple-Carcinogen
Probability: 0.568
Enrichment: 1.372
Bayesian Score: 2.018
Mahalanobis Distance: 18.354
Mahalanobis Distance p-value: 9.15e-009

Prediction: Positive if the Bayesian score is above the estimated best cutoff value from minimizing the false positive and false negative rate.
Probability: The esimated probability that the sample is in the positive category. This assumes that the Bayesian score follows a normal distribution and is different from the prediction using a cutoff.
Enrichment: An estimate of enrichment, that is, the increased likelihood (versus random) of this sample being in the category.
Bayesian Score: The standard Laplacian-modified Bayesian score.
Mahalanobis Distance: The Mahalanobis distance (MD) is the distance to the center of the training data. The larger the MD, the less trustworthy the prediction.
Mahalanobis Distance p-value: The p-value gives the fraction of training data with an MD greater than or equal to the one for the given sample, assuming normally distributed data. The smaller the p-value, the less trustworthy the prediciton. For highly non-normal X properties (e.g., fingerprints), the MD p-value is wildly inaccurate.

Structural Similar Compounds

Name	Moricizine	Nimodipine	Nicardipine
Structure	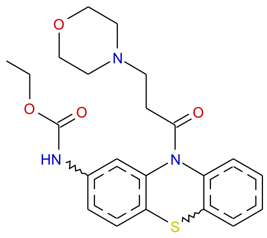	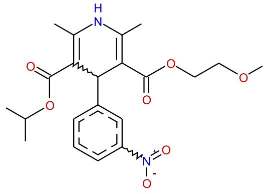	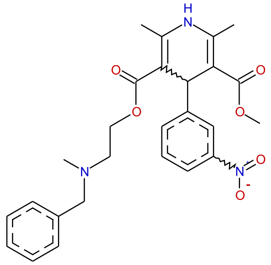
Actual Endpoint	Single-Carcinogen	Single-Carcinogen	Single-Carcinogen
Predicted Endpoint	Single-Carcinogen	Single-Carcinogen	Single-Carcinogen
Distance	0.689	0.714	0.715
Reference	US FDA (Centre for Drug Eval.& Res./Off. Testing & Res.) Sept. 1997	US FDA (Centre for Drug Eval.& Res./Off. Testing & Res.) Sept. 1997	US FDA (Centre for Drug Eval.& Res./Off. Testing & Res.) Sept. 1997

Model Applicability

Unknown features are fingerprint features in the query molecule, but not found in the training set.

1. All properties and OPS components are within expected ranges.

Feature Contribution

Top features for positive contribution				
**Fingerprint**	**Bit/Smiles**	**Feature Structure**	**Score**	**Multiple-Carcinogen in training set**
SCFP_8	2097618059	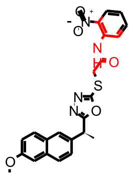 AND Enantiomer [*]CC(=O)N[c](:[cH]:[*]):[cH]:[*]	0.681	6 out of 7

SCFP_8	1631845520	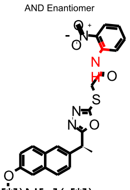 [*]C(=[*])N[c](:[*]):[*]	0.495	6 out of 9
SCFP_8	1311339974	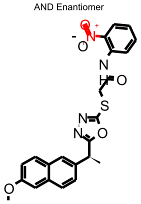 [*][N+](=O)[*]	0.453	8 out of 13
**Top Features for negative contribution**				
**Fingerprint**	**Bit/Smiles**	**Feature Structure**	**Score**	**Multiple-Carcinogen in training set**
SCFP_8	136239834	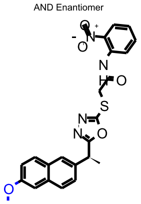 [*]OC	-0.358	3 out of 13
SCFP_8	-1186786545	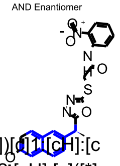 [*]C([*])[c]1[cH]:[cH]:[c]2:[cH]:[c]([*]):[*]:[cH]:[c]:2:[cH]:1	-0.310	0 out of 1
SCFP_8	-699162599	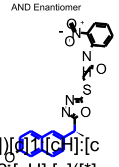 [*]C([*])[c]1[cH]:[cH]:[c]2:[cH]:[c]([*]):[cH]:[cH]:[c]:2:[cH]:1	-0.310	0 out of 1

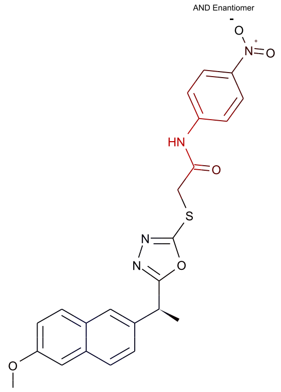

$C_{23}H_{20}N_4O_5S$
Molecular Weight: 464.4937
ALogP: 4.132
Rotatable Bonds: 8
Acceptors: 7
Donors: 1

Model Prediction

Prediction: Multiple-Carcinogen
Probability: 0.552
Enrichment: 1.334
Bayesian Score: 3.778
Mahalanobis Distance: 17.246
Mahalanobis Distance p-value: 9.41e-008

Prediction: Positive if the Bayesian score is above the estimated best cutoff value from minimizing the false positive and false negative rate.
Probability: The esimated probability that the sample is in the positive category. This assumes that the Bayesian score follows a normal distribution and is different from the prediction using a cutoff.
Enrichment: An estimate of enrichment, that is, the increased likelihood (versus random) of this sample being in the category.
Bayesian Score: The standard Laplacian-modified Bayesian score.
Mahalanobis Distance: The Mahalanobis distance (MD) is the distance to the center of the training data. The larger the MD, the less trustworthy the prediction.
Mahalanobis Distance p-value: The p-value gives the fraction of training data with an MD greater than or equal to the one for the given sample, assuming normally distributed data. The smaller the p-value, the less trustworthy the prediciton. For highly non-normal X properties (e.g., fingerprints), the MD p-value is wildly inaccurate.

Structural Similar Compounds

Name	Moricizine	Nimodipine	Nicardipine
Structure	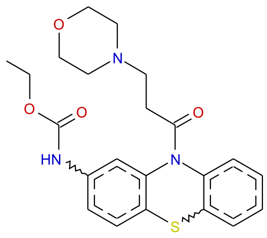	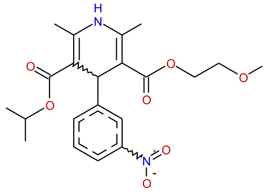	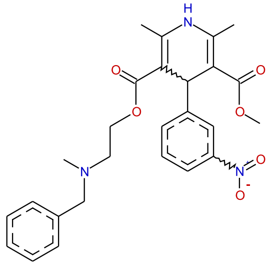
Actual Endpoint	Single-Carcinogen	Single-Carcinogen	Single-Carcinogen
Predicted Endpoint	Single-Carcinogen	Single-Carcinogen	Single-Carcinogen
Distance	0.692	0.717	0.718
Reference	US FDA (Centre for Drug Eval.& Res./Off. Testing & Res.) Sept. 1997	US FDA (Centre for Drug Eval.& Res./Off. Testing & Res.) Sept. 1997	US FDA (Centre for Drug Eval.& Res./Off. Testing & Res.) Sept. 1997

Model Applicability

Unknown features are fingerprint features in the query molecule, but not found in the training set.

1. All properties and OPS components are within expected ranges.

Feature Contribution

Top features for positive contribution

Fingerprint	Bit/Smiles	Feature Structure	Score	Multiple-Carcinogen in training set
SCFP_8	2097618059	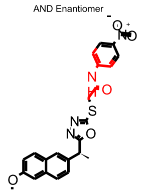 [*]CC(=O)N[c](:[cH]:[*]):[cH]:[*]	0.681	6 out of 7

SCFP_8	-347048986	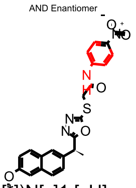 [*]C(=[*])N[c]1:[cH]:[cH]:[*]:[cH]:[cH]:1	0.574	4 out of 5
SCFP_8	814408713	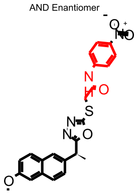 [*]CC(=O)N[c]1:[cH]:[cH]:[cH]:[cH]:[cH]:1	0.553	2 out of 2

Top Features for negative contribution

Fingerprint	Bit/Smiles	Feature Structure	Score	Multiple-Carcinogen in training set
SCFP_8	136239834	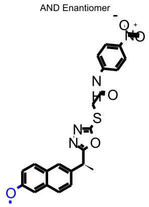 [*]OC	-0.358	3 out of 13
SCFP_8	-699162599	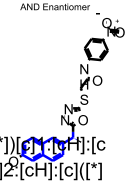 [*]C([*])[c]1:[cH]:[cH]:[cH]:[c]2:[cH]:[c]([*]):[cH]:[cH]:[c]:2:[cH]:1	-0.310	0 out of 1
SCFP_8	125474664	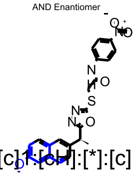 [*]O[c]1:[cH]:[*]:[c](:[*]):[c](:[cH]:[*]):[cH]:1	-0.310	0 out of 1

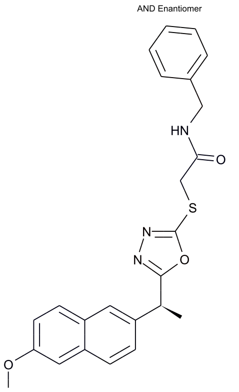

$C_{24}H_{23}N_3O_3S$

Molecular Weight: 433.52272

ALogP: 4.245

Rotatable Bonds: 8

Acceptors: 5

Donors: 1

Structural Similar Compounds

Name	Benzenesulfonic acid, 5-(2H-naphtho(1,2-d)triazol-2-yl)-2-(2-phenyl ethenyl)-, sodium salt	Pregna-1,4-diene-3,20-dione, 21-(acetyloxy)-11-hydroxy-6-methyl-17- (1-oxopropoxy)-, (6-alpha,11-beta)-	Anthraquinone, 1,1'-iminodi-
Structure	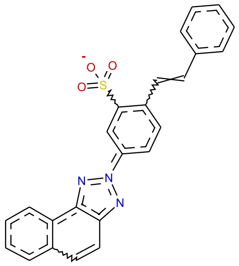	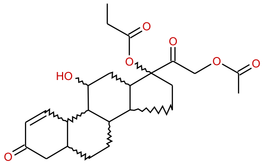	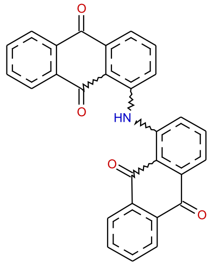
Actual Endpoint	Irritant	Irritant	Irritant
Predicted Endpoint	Irritant	Irritant	Non-Irritant
Distance	0.751	0.773	0.777
Reference	MVCRB3 MVC-Report. (Stockholm, Sweden) No.1-2, 1972-73. Discontinued. Volu me(issue)/page/year: 2,193,1973	YACHDS Yakuri to Chiryo. Pharmacology and Therapeutics. (Raifu Saiensu Shup pan K.K., 2-5-13, Yaesu, Chuo-ku, Tokyo 104, Japan) V.1-1972- Volume(issue) /page/year: 19,3103,1991	85JCAE "Prehled Prumyslove Toxikologie; Organicke Latky," Marhold, J., Prague , Czechoslovakia, Avicenum, 1986 Volume(issue)/page/year: -,735,1986

Model Prediction

Prediction: Non-Irritant

Probability: 0.971

Enrichment: 1.054

Bayesian Score: -0.946

Mahalanobis Distance: 10.604

Mahalanobis Distance p-value: 0.0104

Prediction: Positive if the Bayesian score is above the estimated best cutoff value from minimizing the false positive and false negative rate.
Probability: The esimated probability that the sample is in the positive category. This assumes that the Bayesian score follows a normal distribution and is different from the prediction using a cutoff.
Enrichment: An estimate of enrichment, that is, the increased likelihood (versus random) of this sample being in the category.
Bayesian Score: The standard Laplacian-modified Bayesian score.
Mahalanobis Distance: The Mahalanobis distance (MD) is the distance to the center of the training data. The larger the MD, the less trustworthy the prediction.
Mahalanobis Distance p-value: The p-value gives the fraction of training data with an MD greater than or equal to the one for the given sample, assuming normally distributed data. The smaller the p-value, the less trustworthy the prediciton. For highly non-normal X properties (e.g., fingerprints), the MD p-value is wildly inaccurate.

Model Applicability

Unknown features are fingerprint features in the query molecule, but not found in the training set.

1. OPS PC22 out of range. Value: 3.8034. Training min, max, SD, explained variance: -2.9623, 3.7863, 1.016, 0.0131.

Feature Contribution

Top features for positive contribution

Fingerprint	Bit/Smiles	Feature Structure	Score	Irritant in training set

FCFP_12	907096426	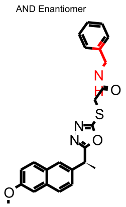<br>[*]NC[c](:[*]):[*]	0.077	7 out of 7
FCFP_12	427906732	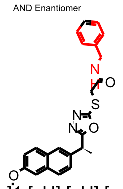<br>[*]NC[c]1:[cH]:[cH]:[*]:[cH]:[cH]:1	0.076	6 out of 6
FCFP_12	-1410079687	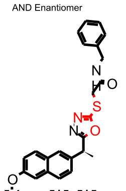<br>[*]S[c]1:o:[*]:[*]:n:1	0.076	6 out of 6
**Top Features for negative contribution**				
**Fingerprint**	**Bit/Smiles**	**Feature Structure**	**Score**	**Irritant in training set**
FCFP_12	566058135	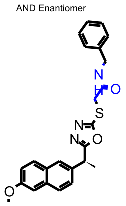<br>[*]CC(=O)N[*]	-0.367	13 out of 21
FCFP_12	346218766	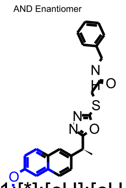<br>[*]:[c]1:[*]:[cH]:[cH]:[c](OC):[cH]:1	-0.091	35 out of 42

FCFP_12	-1320007763	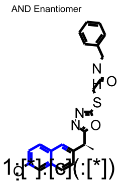 [*][c]1:[*]:[c](:[*]):[c](:[cH]:[*]):[cH]:[cH]:1	-0.089	20 out of 24

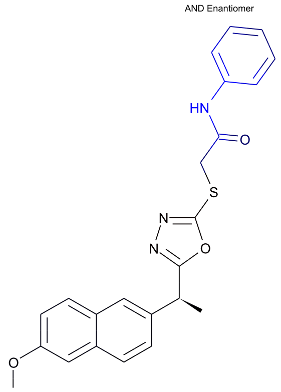

$C_{23}H_{21}N_3O_3S$
Molecular Weight: 419.49614
ALogP: 4.238
Rotatable Bonds: 7
Acceptors: 5
Donors: 1

Model Prediction

Prediction: Non-Irritant
Probability: 0.079
Enrichment: 0.086
Bayesian Score: -6.007
Mahalanobis Distance: 8.791
Mahalanobis Distance p-value: 0.534

Prediction: Positive if the Bayesian score is above the estimated best cutoff value from minimizing the false positive and false negative rate.
Probability: The esimated probability that the sample is in the positive category. This assumes that the Bayesian score follows a normal distribution and is different from the prediction using a cutoff.
Enrichment: An estimate of enrichment, that is, the increased likelihood (versus random) of this sample being in the category.
Bayesian Score: The standard Laplacian-modified Bayesian score.
Mahalanobis Distance: The Mahalanobis distance (MD) is the distance to the center of the training data. The larger the MD, the less trustworthy the prediction.
Mahalanobis Distance p-value: The p-value gives the fraction of training data with an MD greater than or equal to the one for the given sample, assuming normally distributed data. The smaller the p-value, the less trustworthy the prediciton. For highly non-normal X properties (e.g., fingerprints), the MD p-value is wildly inaccurate.

Structural Similar Compounds

Name	Benzenesulfonic acid, 5-(2H-naphtho(1,2-d)triazol-2-yl)-2-(2-phenyl ethenyl)-, sodium salt	Anthraquinone, 1,1'-iminodi-	Pregna-1,4-diene-3,20-dione, 21-(acetyloxy)-11-hydroxy-6-methyl-17- (1-oxopropoxy)-, (6-alpha,11-beta)-
Structure	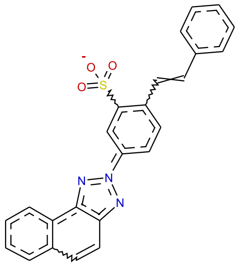	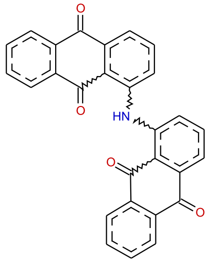	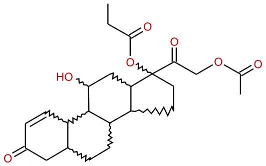
Actual Endpoint	Irritant	Irritant	Irritant
Predicted Endpoint	Irritant	Non-Irritant	Irritant
Distance	0.728	0.738	0.769
Reference	MVCRB3 MVC-Report. (Stockholm, Sweden) No.1-2, 1972-73. Discontinued. Volu me(issue)/page/year: 2,193,1973	85JCAE "Prehled Prumyslove Toxikologie; Organicke Latky," Marhold, J., Prague , Czechoslovakia, Avicenum, 1986 Volume(issue)/page/year: -,735,1986	YACHDS Yakuri to Chiryo. Pharmacology and Therapeutics. (Raifu Saiensu Shup pan K.K., 2-5-13, Yaesu, Chuo-ku, Tokyo 104, Japan) V.1-1972- Volume(issue) /page/year: 19,3103,1991

Model Applicability

Unknown features are fingerprint features in the query molecule, but not found in the training set.

1. All properties and OPS components are within expected ranges.

Feature Contribution

Top features for positive contribution				
Fingerprint	Bit/Smiles	Feature Structure	Score	Irritant in training set

FCFP_12	-1410079687	AND Enantiomer 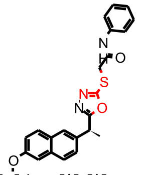 [*]S[c]1:o:[*]:[*]:n:1	0.076	6 out of 6
FCFP_12	4427049	AND Enantiomer 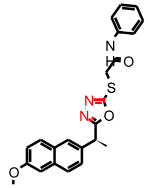 [*][c]1:[*]:[*]:n:n:1	0.073	5 out of 5
FCFP_12	-1539162406	AND Enantiomer 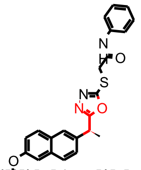 [*]C([*])[c]1:o:[*]:[*]:n:1	0.058	2 out of 2

Top Features for negative contribution

Fingerprint	Bit/Smiles	Feature Structure	Score	Irritant in training set
FCFP_12	1175665944	AND Enantiomer 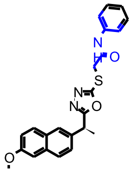 [*]CC(=O)N[c](:[cH]:[*]):[cH]:[*]	-1.020	2 out of 8
FCFP_12	-1838187238	AND Enantiomer 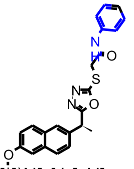 [*]C(=[*])N[c]1:[cH]:[cH]:[*]:[cH]:[cH]:1	-0.692	5 out of 12

FCFP_12	-451043714	AND Enantiomer 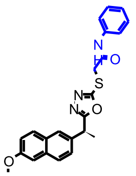 [*]CC(=O)N[c]1:[cH]:[cH]:[cH]:[cH]:[cH]:1	-0.650	0 out of 1

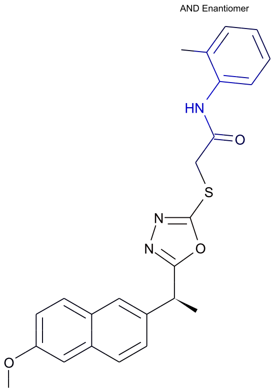

$C_{24}H_{23}N_3O_3S$

Molecular Weight: 433.52272

ALogP: 4.724

Rotatable Bonds: 7

Acceptors: 5

Donors: 1

Model Prediction

Prediction: Non-Irritant

Probability: 0.481

Enrichment: 0.522

Bayesian Score: -4.601

Mahalanobis Distance: 8.900

Mahalanobis Distance p-value: 0.474

Prediction: Positive if the Bayesian score is above the estimated best cutoff value from minimizing the false positive and false negative rate.
Probability: The esimated probability that the sample is in the positive category. This assumes that the Bayesian score follows a normal distribution and is different from the prediction using a cutoff.
Enrichment: An estimate of enrichment, that is, the increased likelihood (versus random) of this sample being in the category.
Bayesian Score: The standard Laplacian-modified Bayesian score.
Mahalanobis Distance: The Mahalanobis distance (MD) is the distance to the center of the training data. The larger the MD, the less trustworthy the prediction.
Mahalanobis Distance p-value: The p-value gives the fraction of training data with an MD greater than or equal to the one for the given sample, assuming normally distributed data. The smaller the p-value, the less trustworthy the prediciton. For highly non-normal X properties (e.g., fingerprints), the MD p-value is wildly inaccurate.

Structural Similar Compounds

Name	Anthraquinone, 1,1'-iminodi-	Benzenesulfonic acid, 5-(2H-naphtho(1,2-d)triazol-2-yl)-2-(2-phenyl ethenyl)-, sodium salt	Phosphorothioic acid, O-ethyl S-propyl O-(2,4,6-trichlorophenyl) es ter
Structure	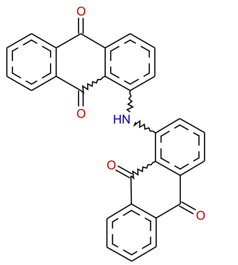	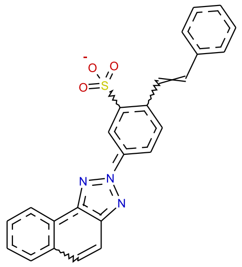	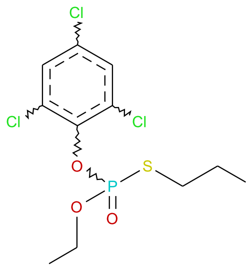
Actual Endpoint	Irritant	Irritant	Irritant
Predicted Endpoint	Non-Irritant	Irritant	Irritant
Distance	0.719	0.749	0.794
Reference	85JCAE "Prehled Prumyslove Toxikologie; Organicke Latky," Marhold, J., Prague , Czechoslovakia, Avicenum, 1986 Volume(issue)/page/year: -,735,1986	MVCRB3 MVC-Report. (Stockholm, Sweden) No.1-2, 1972-73. Discontinued. Volu me(issue)/page/year: 2,193,1973	NTIS** National Technical Information Service. (Springfield, VA 22161) Forme rly U.S. Clearinghouse for Scientific & Technical Information. Volume(issue)/pag e/year: OTS0535844

Model Applicability

Unknown features are fingerprint features in the query molecule, but not found in the training set.

1. All properties and OPS components are within expected ranges.

Feature Contribution

Top features for positive contribution				
Fingerprint	Bit/Smiles	Feature Structure	Score	Irritant in training set

FCFP_12	-1410079687	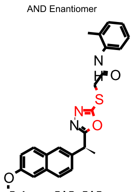 [*]S[c]1:o:[*]:[*]:n:1	0.076	6 out of 6
FCFP_12	4427049	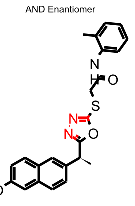 [*][c]1:[*]:[*]:n:n:1	0.073	5 out of 5
FCFP_12	-1371139928	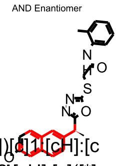 [*]C([*])[c]1:[cH]:[cH]:[c]2:[cH]:[c]([*]):[*]:[cH]:[c]:2:[cH]:1	0.058	2 out of 2

Top Features for negative contribution

Fingerprint	Bit/Smiles	Feature Structure	Score	Irritant in training set
FCFP_12	1175665944	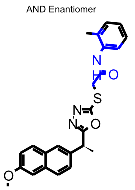 [*]CC(=O)N[c](:[cH]:[*]):[cH]:[*]	-1.020	2 out of 8
FCFP_12	1294255210	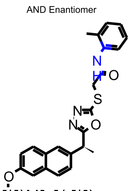 [*]C(=[*])N[c](:[*]):[*]	-0.486	12 out of 22

FCFP_12	-1724769936	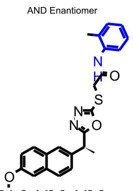 [*]N[c]1:[cH]:[cH]:[cH]:[cH]:1	-0.475	11 out of 20

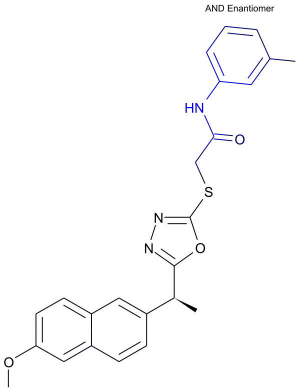

$C_{24}H_{23}N_3O_3S$
Molecular Weight: 433.52272
ALogP: 4.724
Rotatable Bonds: 7
Acceptors: 5
Donors: 1

Model Prediction

Prediction: Non-Irritant
Probability: 0.139
Enrichment: 0.151
Bayesian Score: -5.663
Mahalanobis Distance: 8.900
Mahalanobis Distance p-value: 0.474

Prediction: Positive if the Bayesian score is above the estimated best cutoff value from minimizing the false positive and false negative rate.
Probability: The esimated probability that the sample is in the positive category. This assumes that the Bayesian score follows a normal distribution and is different from the prediction using a cutoff.
Enrichment: An estimate of enrichment, that is, the increased likelihood (versus random) of this sample being in the category.
Bayesian Score: The standard Laplacian-modified Bayesian score.
Mahalanobis Distance: The Mahalanobis distance (MD) is the distance to the center of the training data. The larger the MD, the less trustworthy the prediction.
Mahalanobis Distance p-value: The p-value gives the fraction of training data with an MD greater than or equal to the one for the given sample, assuming normally distributed data. The smaller the p-value, the less trustworthy the prediciton. For highly non-normal X properties (e.g., fingerprints), the MD p-value is wildly inaccurate.

Structural Similar Compounds

Name	Anthraquinone, 1,1'-iminodi-	Benzenesulfonic acid, 5-(2H-naphtho(1,2-d)triazol-2-yl)-2-(2-phenyl ethenyl)-, sodium salt	Phosphorothioic acid, O-ethyl S-propyl O-(2,4,6-trichlorophenyl) es ter
Structure	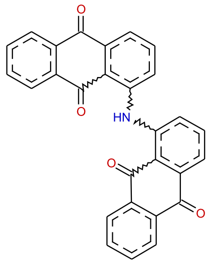	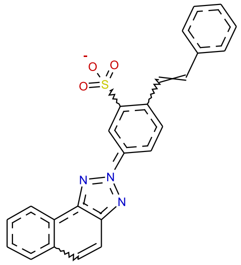	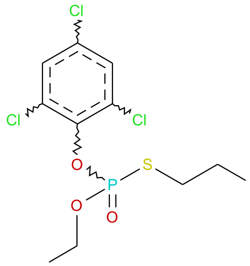
Actual Endpoint	Irritant	Irritant	Irritant
Predicted Endpoint	Non-Irritant	Irritant	Irritant
Distance	0.723	0.752	0.794
Reference	85JCAE "Prehled Prumyslove Toxikologie; Organicke Latky," Marhold, J., Prague , Czechoslovakia, Avicenum, 1986 Volume(issue)/page/year: -,735,1986	MVCRB3 MVC-Report. (Stockholm, Sweden) No.1-2, 1972-73. Discontinued. Volu me(issue)/page/year: 2,193,1973	NTIS** National Technical Information Service. (Springfield, VA 22161) Forme rly U.S. Clearinghouse for Scientific & Technical Information. Volume(issue)/pag e/year: OTS0535844

Model Applicability

Unknown features are fingerprint features in the query molecule, but not found in the training set.

1. All properties and OPS components are within expected ranges.

Feature Contribution

Top features for positive contribution				
Fingerprint	Bit/Smiles	Feature Structure	Score	Irritant in training set

FCFP_12	-1410079687	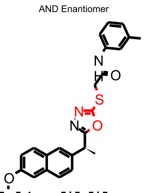<br><br>[*]S[c]1:o:[*]:[*]:n:1	0.076	6 out of 6
FCFP_12	4427049	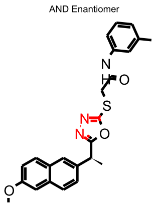<br><br>[*][c]1:[*]:[*]:n:n:1	0.073	5 out of 5
FCFP_12	630418361	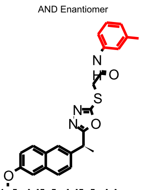<br><br>[*][c]1:[cH]:[cH]:[cH]:[c](C):[cH]:1	0.073	5 out of 5

Top Features for negative contribution

Fingerprint	Bit/Smiles	Feature Structure	Score	Irritant in training set
FCFP_12	1175665944	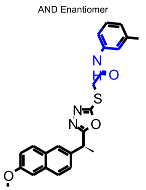<br><br>[*]CC(=O)N[c](:[cH]:[*]):[cH]:[*]	-1.020	2 out of 8
FCFP_12	-1838187238	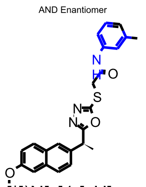<br><br>[*]C(=[*])N[c]1:[cH]:[cH]:[*]:[cH]:[cH]:1	-0.692	5 out of 12

FCFP_12	-453277354	AND Enantiomer<br>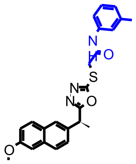<br>[*]CC(=O)N[c]1:[cH]:[cH]:[cH]:[c](C):[cH]:1	-0.650	0 out of 1

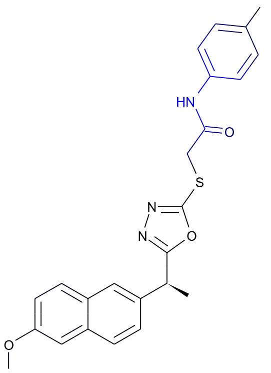

$C_{24}H_{23}N_3O_3S$
Molecular Weight: 433.52272
ALogP: 4.724
Rotatable Bonds: 7
Acceptors: 5
Donors: 1

Model Prediction

Prediction: Non-Irritant
Probability: 0.287
Enrichment: 0.312
Bayesian Score: -5.132
Mahalanobis Distance: 8.900
Mahalanobis Distance p-value: 0.474

Prediction: Positive if the Bayesian score is above the estimated best cutoff value from minimizing the false positive and false negative rate.
Probability: The esimated probability that the sample is in the positive category. This assumes that the Bayesian score follows a normal distribution and is different from the prediction using a cutoff.
Enrichment: An estimate of enrichment, that is, the increased likelihood (versus random) of this sample being in the category.
Bayesian Score: The standard Laplacian-modified Bayesian score.
Mahalanobis Distance: The Mahalanobis distance (MD) is the distance to the center of the training data. The larger the MD, the less trustworthy the prediction.
Mahalanobis Distance p-value: The p-value gives the fraction of training data with an MD greater than or equal to the one for the given sample, assuming normally distributed data. The smaller the p-value, the less trustworthy the prediciton. For highly non-normal X properties (e.g., fingerprints), the MD p-value is wildly inaccurate.

Structural Similar Compounds

Name	Anthraquinone, 1,1'-iminodi-	Benzenesulfonic acid, 5-(2H-naphtho(1,2-d)triazol-2-yl)-2-(2-phenyl ethenyl)-, sodium salt	Phosphorothioic acid, O-ethyl S-propyl O-(2,4,6-trichlorophenyl) es ter
Structure	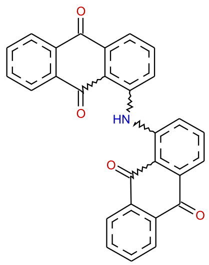	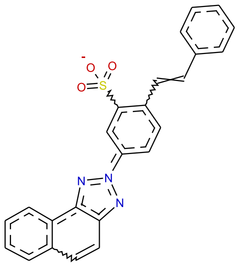	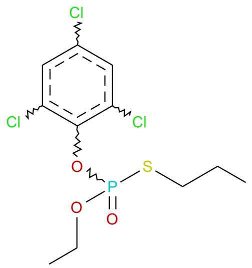
Actual Endpoint	Irritant	Irritant	Irritant
Predicted Endpoint	Non-Irritant	Irritant	Irritant
Distance	0.727	0.755	0.794
Reference	85JCAE "Prehled Prumyslove Toxikologie; Organicke Latky," Marhold, J., Prague , Czechoslovakia, Avicenum, 1986 Volume(issue)/page/year: -,735,1986	MVCRB3 MVC-Report. (Stockholm, Sweden) No.1-2, 1972-73. Discontinued. Volu me(issue)/page/year: 2,193,1973	NTIS** National Technical Information Service. (Springfield, VA 22161) Forme rly U.S. Clearinghouse for Scientific & Technical Information. Volume(issue)/pag e/year: OTS0535844

Model Applicability

Unknown features are fingerprint features in the query molecule, but not found in the training set.

1. All properties and OPS components are within expected ranges.

Feature Contribution

Top features for positive contribution				
**Fingerprint**	**Bit/Smiles**	**Feature Structure**	**Score**	**Irritant in training set**

FCFP_12	-1410079687	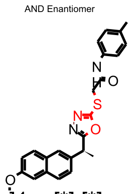 [*]S[c]1:o:[*]:[*]:n:1	0.076	6 out of 6
FCFP_12	4427049	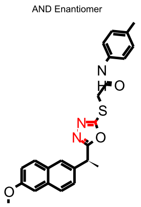 [*][c]1:[*]:[*]:n:n:1	0.073	5 out of 5
FCFP_12	-1539162406	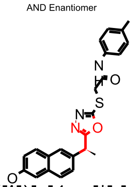 [*]C([*])[c]1:o:[*]:[*]:n:1	0.058	2 out of 2

Top Features for negative contribution

Fingerprint	Bit/Smiles	Feature Structure	Score	Irritant in training set
FCFP_12	1175665944	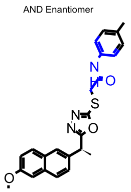 [*]CC(=O)N[c](:[cH]:[*]):[cH]:[*]	-1.020	2 out of 8
FCFP_12	-1838187238	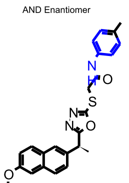 [*]C(=[*])N[c]1:[cH]:[cH]:[*]:[cH]:[cH]:1	-0.692	5 out of 12

FCFP_12	-451043714	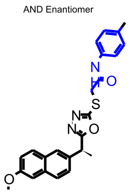 [*]CC(=O)N[c]1:[cH]:[cH]:[cH]:[cH]:1	-0.650	0 out of 1

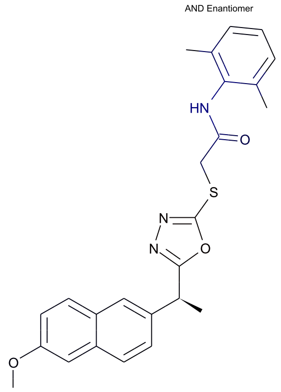

$C_{25}H_{25}N_3O_3S$
Molecular Weight: 447.5493
ALogP: 5.21
Rotatable Bonds: 7
Acceptors: 5
Donors: 1

Model Prediction

Prediction: Non-Irritant
Probability: 0.844
Enrichment: 0.917
Bayesian Score: -3.244
Mahalanobis Distance: 9.025
Mahalanobis Distance p-value: 0.407

Prediction: Positive if the Bayesian score is above the estimated best cutoff value from minimizing the false positive and false negative rate.
Probability: The esimated probability that the sample is in the positive category. This assumes that the Bayesian score follows a normal distribution and is different from the prediction using a cutoff.
Enrichment: An estimate of enrichment, that is, the increased likelihood (versus random) of this sample being in the category.
Bayesian Score: The standard Laplacian-modified Bayesian score.
Mahalanobis Distance: The Mahalanobis distance (MD) is the distance to the center of the training data. The larger the MD, the less trustworthy the prediction.
Mahalanobis Distance p-value: The p-value gives the fraction of training data with an MD greater than or equal to the one for the given sample, assuming normally distributed data. The smaller the p-value, the less trustworthy the prediciton. For highly non-normal X properties (e.g., fingerprints), the MD p-value is wildly inaccurate.

Structural Similar Compounds

Name	Anthraquinone, 1,1'-iminodi-	Benzenesulfonic acid, 2,2'-(4,4'-biphenylylenedivinylene)di-, disod ium salt	Benzenesulfonic acid, 5-(2H-naphtho(1,2-d)triazol-2-yl)-2-(2-phenyl ethenyl)-, sodium salt
Structure	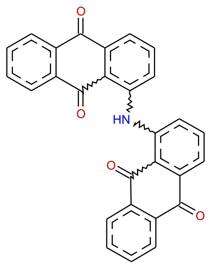	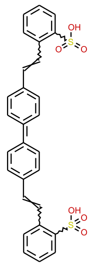	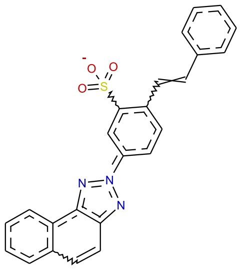
Actual Endpoint	Irritant	Irritant	Irritant
Predicted Endpoint	Non-Irritant	Non-Irritant	Irritant
Distance	0.722	0.779	0.780
Reference	85JCAE "Prehled Prumyslove Toxikologie; Organicke Latky," Marhold, J., Prague , Czechoslovakia, Avicenum, 1986 Volume(issue)/page/year: -,735,1986	MVCRB3 MVC-Report. (Stockholm, Sweden) No.1-2, 1972-73. Discontinued. Volu me(issue)/page/year: 2,193,1973	MVCRB3 MVC-Report. (Stockholm, Sweden) No.1-2, 1972-73. Discontinued. Volu me(issue)/page/year: 2,193,1973

Model Applicability

Unknown features are fingerprint features in the query molecule, but not found in the training set.

1. All properties and OPS components are within expected ranges.

Feature Contribution

Top features for positive contribution				
Fingerprint	Bit/Smiles	Feature Structure	Score	Irritant in training set

FCFP_12	-1410079687	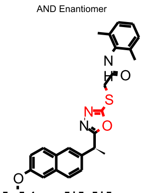 [*]S[c]1:o:[*]:[*]:n:1	0.076	6 out of 6
FCFP_12	4427049	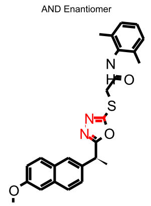 [*][c]1:[*]:[*]:n:n:1	0.073	5 out of 5
FCFP_12	1396506317	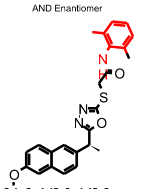 [*]N[c]1:[cH]:[cH]:[cH]:[cH]:[c]:1C	0.058	2 out of 2

Top Features for negative contribution

Fingerprint	Bit/Smiles	Feature Structure	Score	Irritant in training set
FCFP_12	1175665944	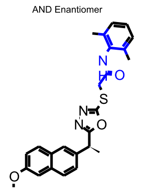 [*]CC(=O)N[c](:[cH]:[*]):[cH]:[*]	-1.020	2 out of 8
FCFP_12	1294255210	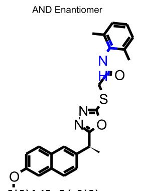 [*]C(=[*])N[c](:[*]):[*]	-0.486	12 out of 22

FCFP_12	590925877	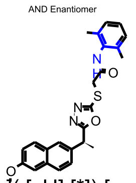<br>[*]N[c](:[cH]:[*]):[cH]:[*]	-0.434	56 out of 95

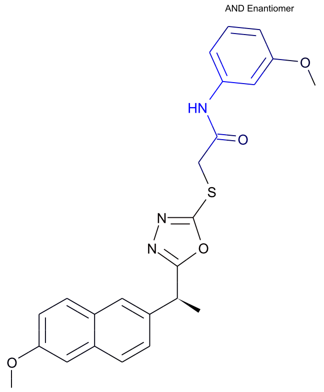

$C_{24}H_{23}N_3O_4S$

Molecular Weight: 449.52212

ALogP: 4.221

Rotatable Bonds: 8

Acceptors: 6

Donors: 1

Model Prediction

Prediction: Non-Irritant

Probability: 0.099

Enrichment: 0.107

Bayesian Score: -5.879

Mahalanobis Distance: 8.758

Mahalanobis Distance p-value: 0.552

Prediction: Positive if the Bayesian score is above the estimated best cutoff value from minimizing the false positive and false negative rate.
Probability: The esimated probability that the sample is in the positive category. This assumes that the Bayesian score follows a normal distribution and is different from the prediction using a cutoff.
Enrichment: An estimate of enrichment, that is, the increased likelihood (versus random) of this sample being in the category.
Bayesian Score: The standard Laplacian-modified Bayesian score.
Mahalanobis Distance: The Mahalanobis distance (MD) is the distance to the center of the training data. The larger the MD, the less trustworthy the prediction.
Mahalanobis Distance p-value: The p-value gives the fraction of training data with an MD greater than or equal to the one for the given sample, assuming normally distributed data. The smaller the p-value, the less trustworthy the prediciton. For highly non-normal X properties (e.g., fingerprints), the MD p-value is wildly inaccurate.

Structural Similar Compounds

Name	Pregna-1,4-diene-3,20-dione, 21-(acetyloxy)-11-hydroxy-6-methyl-17- (1-oxopropoxy)-, (6-alpha,11-beta)-	Benzenesulfonic acid, 2,2'-(4,4'-biphenylylenedivinylene)di-, disod ium salt	Benzenesulfonic acid, 5-(2H-naphtho(1,2-d)triazol-2-yl)-2-(2-phenyl ethenyl)-, sodium salt
Structure	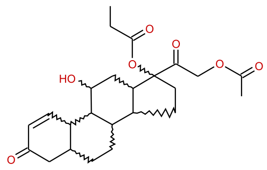	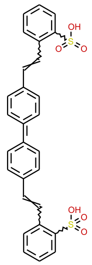	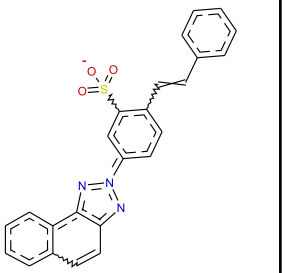
Actual Endpoint	Irritant	Irritant	Irritant
Predicted Endpoint	Irritant	Non-Irritant	Irritant
Distance	0.718	0.781	0.784
Reference	YACHDS Yakuri to Chiryo. Pharmacology and Therapeutics. (Raifu Saiensu Shup pan K.K., 2-5-13, Yaesu, Chuo-ku, Tokyo 104, Japan) V.1-1972- Volume(issue) /page/year: 19,3103,1991	MVCRB3 MVC-Report. (Stockholm, Sweden) No.1-2, 1972-73. Discontinued. Volu me(issue)/page/year: 2,193,1973	MVCRB3 MVC-Report. (Stockholm, Sweden) No.1-2, 1972-73. Discontinued. Volu me(issue)/page/year: 2,193,1973

Model Applicability

Unknown features are fingerprint features in the query molecule, but not found in the training set.

1. All properties and OPS components are within expected ranges.

Feature Contribution

Top features for positive contribution				
Fingerprint	Bit/Smiles	Feature Structure	Score	Irritant in training set

FCFP_12	-1410079687	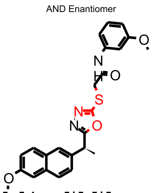 [*]S[c]1:o:[*]:[*]:n:1	0.076	6 out of 6
FCFP_12	4427049	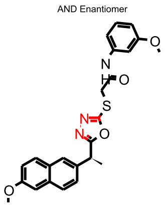 [*][c]1:[*]:[*]:n:n:1	0.073	5 out of 5
FCFP_12	-1539162406	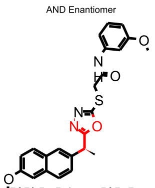 [*]C([*])[c]1:o:[*]:[*]:n:1	0.058	2 out of 2
**Top Features for negative contribution**				
**Fingerprint**	**Bit/Smiles**	**Feature Structure**	**Score**	**Irritant in training set**
FCFP_12	1175665944	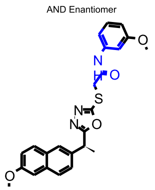 [*]CC(=O)N[c](:[cH]:[*]):[cH]:[*]	-1.020	2 out of 8
FCFP_12	-1838187238	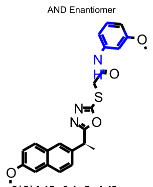 [*]C(=[*])N[c]1:[cH]:[cH]:[*]:[cH]:[cH]:1	-0.692	5 out of 12

FCFP_12	-792685140	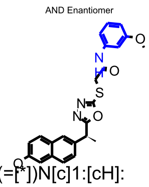 [*]C(=[*])N[c]1:[cH]:[cH]:[cH]:[cH]:[cH]:1	-0.650	0 out of 1

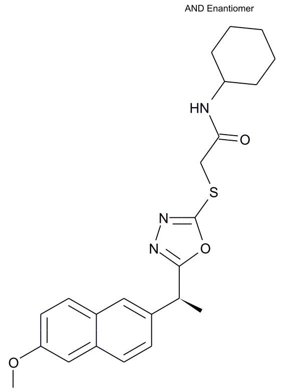

$C_{23}H_{27}N_3O_3S$
Molecular Weight: 425.54378
ALogP: 4.52
Rotatable Bonds: 7
Acceptors: 5
Donors: 1

Model Prediction

Prediction: Non-Irritant
Probability: 0.971
Enrichment: 1.054
Bayesian Score: -0.965
Mahalanobis Distance: 12.627
Mahalanobis Distance p-value: 8.31e-007

Prediction: Positive if the Bayesian score is above the estimated best cutoff value from minimizing the false positive and false negative rate.
Probability: The esimated probability that the sample is in the positive category. This assumes that the Bayesian score follows a normal distribution and is different from the prediction using a cutoff.
Enrichment: An estimate of enrichment, that is, the increased likelihood (versus random) of this sample being in the category.
Bayesian Score: The standard Laplacian-modified Bayesian score.
Mahalanobis Distance: The Mahalanobis distance (MD) is the distance to the center of the training data. The larger the MD, the less trustworthy the prediction.
Mahalanobis Distance p-value: The p-value gives the fraction of training data with an MD greater than or equal to the one for the given sample, assuming normally distributed data. The smaller the p-value, the less trustworthy the prediciton. For highly non-normal X properties (e.g., fingerprints), the MD p-value is wildly inaccurate.

Structural Similar Compounds

Name	Anthraquinone, 1,1'-iminodi-	Benzenesulfonic acid, 5-(2H-naphtho(1,2-d)triazol-2-yl)-2-(2-phenyl ethenyl)-, sodium salt	Pregna-1,4-diene-3,20-dione, 21-(acetyloxy)-11-hydroxy-6-methyl-17- (1-oxopropoxy)-, (6-alpha,11-beta)-
Structure	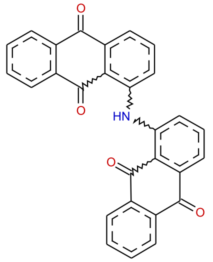	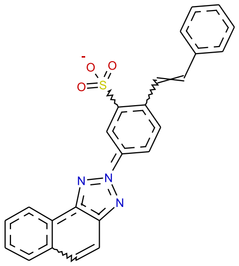	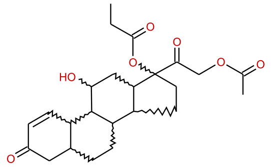
Actual Endpoint	Irritant	Irritant	Irritant
Predicted Endpoint	Non-Irritant	Irritant	Irritant
Distance	0.739	0.744	0.783
Reference	85JCAE "Prehled Prumyslove Toxikologie; Organicke Latky," Marhold, J., Prague , Czechoslovakia, Avicenum, 1986 Volume(issue)/page/year: -,735,1986	MVCRB3 MVC-Report. (Stockholm, Sweden) No.1-2, 1972-73. Discontinued. Volu me(issue)/page/year: 2,193,1973	YACHDS Yakuri to Chiryo. Pharmacology and Therapeutics. (Raifu Saiensu Shup pan K.K., 2-5-13, Yaesu, Chuo-ku, Tokyo 104, Japan) V.1-1972- Volume(issue) /page/year: 19,3103,1991

Model Applicability

Unknown features are fingerprint features in the query molecule, but not found in the training set.

1. OPS PC22 out of range. Value: 4.2266. Training min, max, SD, explained variance: -2.9623, 3.7863, 1.016, 0.0131.

Feature Contribution

Top features for positive contribution				
**Fingerprint**	**Bit/Smiles**	**Feature Structure**	**Score**	**Irritant in training set**

FCFP_12	-1410079687	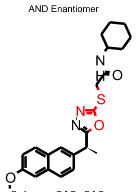 [*]S[c]1:o:[*]:[*]:n:1	0.076	6 out of 6
FCFP_12	4427049	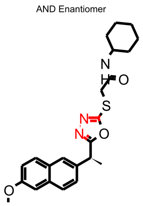 [*][c]1:[*]:[*]:n:n:1	0.073	5 out of 5
FCFP_12	-551279842	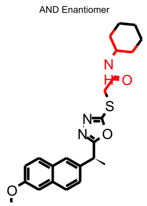 [*]CC(C[*])NC(=O)C[*]	0.066	3 out of 3

Top Features for negative contribution

Fingerprint	Bit/Smiles	Feature Structure	Score	Irritant in training set
FCFP_12	566058135	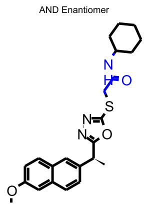 [*]CC(=O)N[*]	-0.367	13 out of 21
FCFP_12	346218766	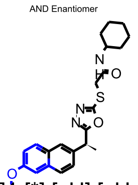 [*]:[c]1:[*]:[cH]:[cH]:[c](OC):[cH]:1	-0.091	35 out of 42

FCFP_12	-1320007763	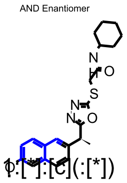 [*][c]1:[*]:[c](:[*]):[c](:[cH]:[*]):[cH]:[cH]:1	-0.089	20 out of 24

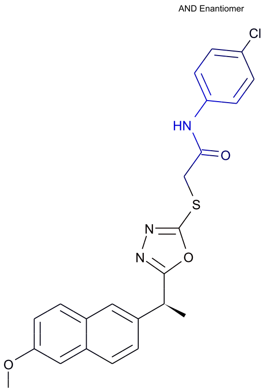

$C_{23}H_{20}ClN_3O_3S$
Molecular Weight: 453.9412
ALogP: 4.902
Rotatable Bonds: 7
Acceptors: 5
Donors: 1

Model Prediction

Prediction: Non-Irritant
Probability: 0.265
Enrichment: 0.288
Bayesian Score: -5.200
Mahalanobis Distance: 9.163
Mahalanobis Distance p-value: 0.337

Prediction: Positive if the Bayesian score is above the estimated best cutoff value from minimizing the false positive and false negative rate.
Probability: The esimated probability that the sample is in the positive category. This assumes that the Bayesian score follows a normal distribution and is different from the prediction using a cutoff.
Enrichment: An estimate of enrichment, that is, the increased likelihood (versus random) of this sample being in the category.
Bayesian Score: The standard Laplacian-modified Bayesian score.
Mahalanobis Distance: The Mahalanobis distance (MD) is the distance to the center of the training data. The larger the MD, the less trustworthy the prediction.
Mahalanobis Distance p-value: The p-value gives the fraction of training data with an MD greater than or equal to the one for the given sample, assuming normally distributed data. The smaller the p-value, the less trustworthy the prediciton. For highly non-normal X properties (e.g., fingerprints), the MD p-value is wildly inaccurate.

Structural Similar Compounds

Name	Anthraquinone, 1,1'-iminodi-	Benzenesulfonic acid, 5-(2H-naphtho(1,2-d)triazol-2-yl)-2-(2-phenyl ethenyl)-, sodium salt	Benzenesulfonic acid, 2,2'-(4,4'-biphenylylenedivinylene)di-, disod ium salt
Structure	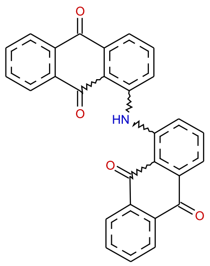	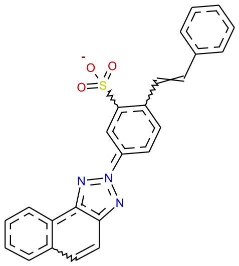	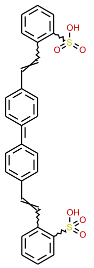
Actual Endpoint	Irritant	Irritant	Irritant
Predicted Endpoint	Non-Irritant	Irritant	Non-Irritant
Distance	0.729	0.769	0.783
Reference	85JCAE "Prehled Prumyslove Toxikologie; Organicke Latky," Marhold, J., Prague , Czechoslovakia, Avicenum, 1986 Volume(issue)/page/year: -,735,1986	MVCRB3 MVC-Report. (Stockholm, Sweden) No.1-2, 1972-73. Discontinued. Volu me(issue)/page/year: 2,193,1973	MVCRB3 MVC-Report. (Stockholm, Sweden) No.1-2, 1972-73. Discontinued. Volu me(issue)/page/year: 2,193,1973

Model Applicability

Unknown features are fingerprint features in the query molecule, but not found in the training set.

1. All properties and OPS components are within expected ranges.

Feature Contribution

Top features for positive contribution				
**Fingerprint**	**Bit/Smiles**	**Feature Structure**	**Score**	**Irritant in training set**

FCFP_12	-1410079687	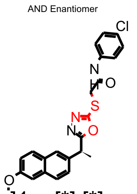 [*]S[c]1:o:[*]:[*]:n:1	0.076	6 out of 6
FCFP_12	4427049	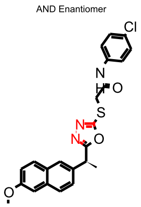 [*][c]1:[*]:[*]:n:n:1	0.073	5 out of 5
FCFP_12	-1539162406	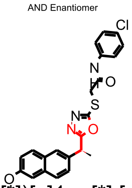 [*]C([*])[c]1:o:[*]:[*]:n:1	0.058	2 out of 2
**Top Features for negative contribution**				
**Fingerprint**	**Bit/Smiles**	**Feature Structure**	**Score**	**Irritant in training set**
FCFP_12	1175665944	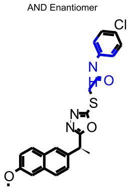 [*]CC(=O)N[c](:[cH]:[*]):[cH]:[*]	-1.020	2 out of 8
FCFP_12	-1838187238	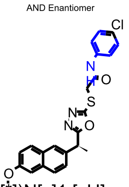 [*]C(=[*])N[c]1:[cH]:[cH]:[*]:[cH]:[cH]:1	-0.692	5 out of 12

FCFP_12	-451043714	AND Enantiomer 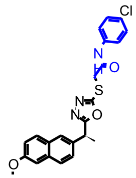 [*]CC(=O)N[c]1:[cH]:[cH]:[cH]:[cH]:1	-0.650	0 out of 1

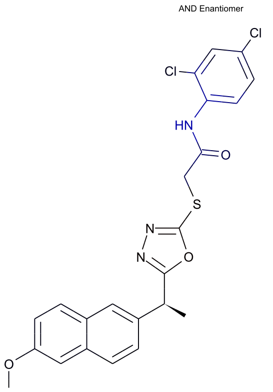

$C_{23}H_{19}Cl_2N_3O_3S$

Molecular Weight: 488.38626

ALogP: 5.567

Rotatable Bonds: 7

Acceptors: 5

Donors: 1

Model Prediction

Prediction: Non-Irritant

Probability: 0.609

Enrichment: 0.662

Bayesian Score: -4.238

Mahalanobis Distance: 9.313

Mahalanobis Distance p-value: 0.267

Prediction: Positive if the Bayesian score is above the estimated best cutoff value from minimizing the false positive and false negative rate.
Probability: The esimated probability that the sample is in the positive category. This assumes that the Bayesian score follows a normal distribution and is different from the prediction using a cutoff.
Enrichment: An estimate of enrichment, that is, the increased likelihood (versus random) of this sample being in the category.
Bayesian Score: The standard Laplacian-modified Bayesian score.
Mahalanobis Distance: The Mahalanobis distance (MD) is the distance to the center of the training data. The larger the MD, the less trustworthy the prediction.
Mahalanobis Distance p-value: The p-value gives the fraction of training data with an MD greater than or equal to the one for the given sample, assuming normally distributed data. The smaller the p-value, the less trustworthy the prediciton. For highly non-normal X properties (e.g., fingerprints), the MD p-value is wildly inaccurate.

Structural Similar Compounds

Name	Benzenesulfonic acid, 2,2'-(4,4'-biphenylylenedivinylene)di-, disod ium salt	Anthraquinone, 1,1'-iminodi-	Benzenesulfonic acid, 5-(2H-naphtho(1,2-d)triazol-2-yl)-2-(2-phenyl ethenyl)-, sodium salt
Structure	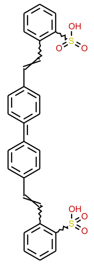	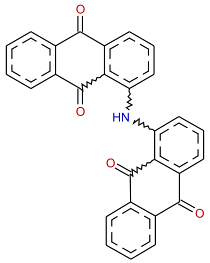	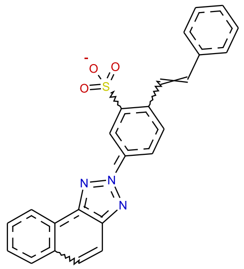
Actual Endpoint	Irritant	Irritant	Irritant
Predicted Endpoint	Non-Irritant	Non-Irritant	Irritant
Distance	0.747	0.749	0.827
Reference	MVCRB3 MVC-Report. (Stockholm, Sweden) No.1-2, 1972-73. Discontinued. Volu me(issue)/page/year: 2,193,1973	85JCAE "Prehled Prumyslove Toxikologie; Organicke Latky," Marhold, J., Prague , Czechoslovakia, Avicenum, 1986 Volume(issue)/page/year: -,735,1986	MVCRB3 MVC-Report. (Stockholm, Sweden) No.1-2, 1972-73. Discontinued. Volu me(issue)/page/year: 2,193,1973

Model Applicability

Unknown features are fingerprint features in the query molecule, but not found in the training set.

1. All properties and OPS components are within expected ranges.

Feature Contribution

Top features for positive contribution

Fingerprint	Bit/Smiles	Feature Structure	Score	Irritant in training set

FCFP_12	-1410079687	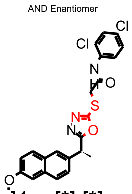 [*]S[c]1:o:[*]:[*]:n:1	0.076	6 out of 6
FCFP_12	4427049	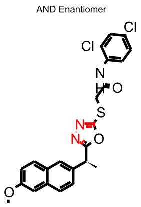 [*][c]1:[*]:[*]:n:n:1	0.073	5 out of 5
FCFP_12	73264552	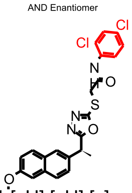 [*][c]1:[cH]:[cH]:[c](Cl):[cH]:[c]:1Cl	0.066	3 out of 3

Top Features for negative contribution

Fingerprint	Bit/Smiles	Feature Structure	Score	Irritant in training set
FCFP_12	1175665944	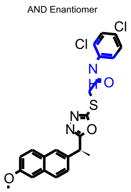 [*]CC(=O)N[c](:[cH]:[*]):[cH]:[*]	-1.020	2 out of 8
FCFP_12	1783756416	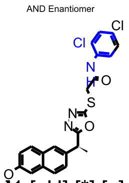 [*]N[c]1:[cH]:[*]:[c]([*]):[cH]:[c]:1Cl	-0.509	4 out of 8

FCFP_12	1294255210	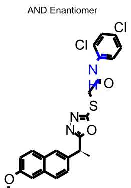 [*]C(=[*])N[c](:[*]):[*]	-0.486	12 out of 22

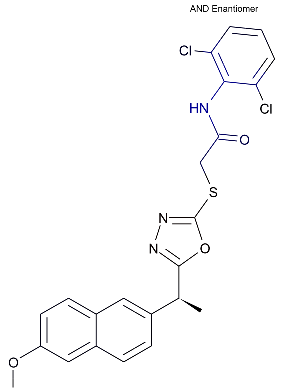

$C_{23}H_{19}Cl_2N_3O_3S$
Molecular Weight: 488.38626
ALogP: 5.567
Rotatable Bonds: 7
Acceptors: 5
Donors: 1

Model Prediction

Prediction: Non-Irritant
Probability: 0.731
Enrichment: 0.794
Bayesian Score: -3.822
Mahalanobis Distance: 9.313
Mahalanobis Distance p-value: 0.267

Prediction: Positive if the Bayesian score is above the estimated best cutoff value from minimizing the false positive and false negative rate.
Probability: The esimated probability that the sample is in the positive category. This assumes that the Bayesian score follows a normal distribution and is different from the prediction using a cutoff.
Enrichment: An estimate of enrichment, that is, the increased likelihood (versus random) of this sample being in the category.
Bayesian Score: The standard Laplacian-modified Bayesian score.
Mahalanobis Distance: The Mahalanobis distance (MD) is the distance to the center of the training data. The larger the MD, the less trustworthy the prediction.
Mahalanobis Distance p-value: The p-value gives the fraction of training data with an MD greater than or equal to the one for the given sample, assuming normally distributed data. The smaller the p-value, the less trustworthy the prediciton. For highly non-normal X properties (e.g., fingerprints), the MD p-value is wildly inaccurate.

Structural Similar Compounds

Name	Benzenesulfonic acid, 2,2'-(4,4'-biphenylylenedivinylene)di-, disod ium salt	Anthraquinone, 1,1'-iminodi-	Benzenesulfonic acid, 5-(2H-naphtho(1,2-d)triazol-2-yl)-2-(2-phenyl ethenyl)-, sodium salt
Structure	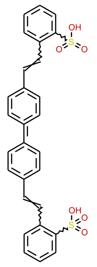	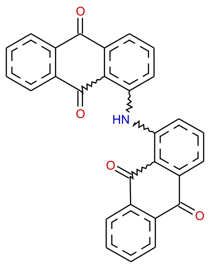	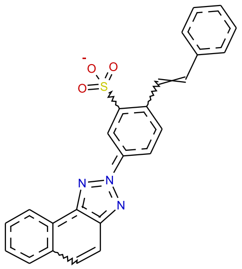
Actual Endpoint	Irritant	Irritant	Irritant
Predicted Endpoint	Non-Irritant	Non-Irritant	Irritant
Distance	0.743	0.748	0.823
Reference	MVCRB3 MVC-Report. (Stockholm, Sweden) No.1-2, 1972-73. Discontinued. Volu me(issue)/page/year: 2,193,1973	85JCAE "Prehled Prumyslove Toxikologie; Organicke Latky," Marhold, J., Prague , Czechoslovakia, Avicenum, 1986 Volume(issue)/page/year: -,735,1986	MVCRB3 MVC-Report. (Stockholm, Sweden) No.1-2, 1972-73. Discontinued. Volu me(issue)/page/year: 2,193,1973

Model Applicability

Unknown features are fingerprint features in the query molecule, but not found in the training set.

1. All properties and OPS components are within expected ranges.

Feature Contribution

Top features for positive contribution				
**Fingerprint**	**Bit/Smiles**	**Feature Structure**	**Score**	**Irritant in training set**

FCFP_12	-1410079687	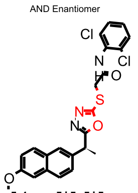 [*]S[c]1:o:[*]:[*]:n:1	0.076	6 out of 6
FCFP_12	4427049	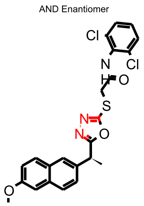 [*][c]1:[*]:[*]:n:n:1	0.073	5 out of 5
FCFP_12	1161767339	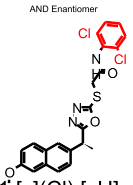 [*][c]1:[c](Cl):[cH]:[cH]:[cH]:[c]:1Cl	0.058	2 out of 2

Top Features for negative contribution

Fingerprint	Bit/Smiles	Feature Structure	Score	Irritant in training set
FCFP_12	1175665944	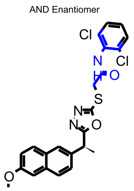 [*]CC(=O)N[c](:[cH]:[*]):[cH]:[*]	-1.020	2 out of 8
FCFP_12	1783756416	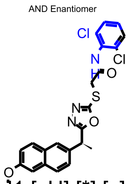 [*]N[c]1:[cH]:[*]:[c]([*]):[cH]:[c]:1Cl	-0.509	4 out of 8

FCFP_12	1294255210	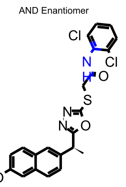<br>[*]C(=[*])N[c](:[*]):<br>[*]	-0.486	12 out of 22

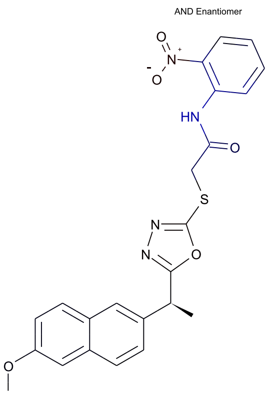

$C_{23}H_{20}N_4O_5S$

Molecular Weight: 464.4937

ALogP: 4.132

Rotatable Bonds: 8

Acceptors: 7

Donors: 1

Model Prediction

Prediction: Non-Irritant

Probability: 0.804

Enrichment: 0.873

Bayesian Score: -3.484

Mahalanobis Distance: 9.768

Mahalanobis Distance p-value: 0.11

Prediction: Positive if the Bayesian score is above the estimated best cutoff value from minimizing the false positive and false negative rate.
Probability: The esimated probability that the sample is in the positive category. This assumes that the Bayesian score follows a normal distribution and is different from the prediction using a cutoff.
Enrichment: An estimate of enrichment, that is, the increased likelihood (versus random) of this sample being in the category.
Bayesian Score: The standard Laplacian-modified Bayesian score.
Mahalanobis Distance: The Mahalanobis distance (MD) is the distance to the center of the training data. The larger the MD, the less trustworthy the prediction.
Mahalanobis Distance p-value: The p-value gives the fraction of training data with an MD greater than or equal to the one for the given sample, assuming normally distributed data. The smaller the p-value, the less trustworthy the prediciton. For highly non-normal X properties (e.g., fingerprints), the MD p-value is wildly inaccurate.

Structural Similar Compounds

Name	Pregna-1,4-diene-3,20-dione, 21-(acetyloxy)-11-hydroxy-6-methyl-17- (1-oxopropoxy)-, (6-alpha,11-beta)-	Benzenesulfonic acid, 2,2'-(4,4'-biphenylylenedivinylene)di-, disod ium salt	2-Anthracenesulfonic acid, 1-amino-9,10-dihydro-9,10-dioxo-4-(2,4,6 -trimethylanilino)-, monosodium salt
Structure	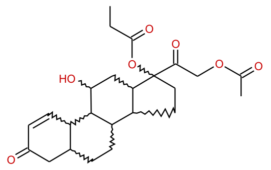	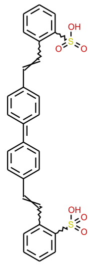	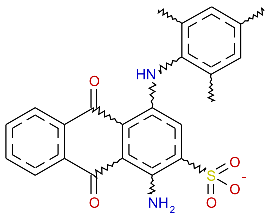
Actual Endpoint	Irritant	Irritant	Irritant
Predicted Endpoint	Irritant	Non-Irritant	Non-Irritant
Distance	0.803	0.813	0.831
Reference	YACHDS Yakuri to Chiryo. Pharmacology and Therapeutics. (Raifu Saiensu Shup pan K.K., 2-5-13, Yaesu, Chuo-ku, Tokyo 104, Japan) V.1-1972- Volume(issue) /page/year: 19,3103,1991	MVCRB3 MVC-Report. (Stockholm, Sweden) No.1-2, 1972-73. Discontinued. Volu me(issue)/page/year: 2,193,1973	85JCAE "Prehled Prumyslove Toxikologie; Organicke Latky," Marhold, J., Prague , Czechoslovakia, Avicenum, 1986 Volume(issue)/page/year: -,1327,1986

Model Applicability

Unknown features are fingerprint features in the query molecule, but not found in the training set.

1. All properties and OPS components are within expected ranges.

Feature Contribution

Top features for positive contribution				
**Fingerprint**	**Bit/Smiles**	**Feature Structure**	**Score**	**Irritant in training set**

FCFP_12	5	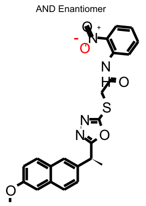 [*][O-]	0.085	27 out of 27
FCFP_12	8	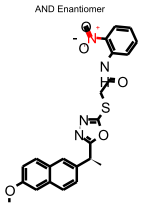 [*][N+](=[*])[*]	0.084	20 out of 20
FCFP_12	-828984032	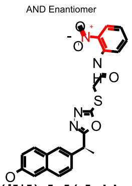 [*][c](:[*]):[c](:[cH]:[*])[N+](=[*])[*]	0.079	9 out of 9
**Top Features for negative contribution**				
**Fingerprint**	**Bit/Smiles**	**Feature Structure**	**Score**	**Irritant in training set**
FCFP_12	1175665944	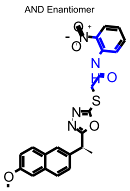 [*]CC(=O)N[c](:[cH]:[*]):[cH]:[*]	-1.020	2 out of 8
FCFP_12	1294255210	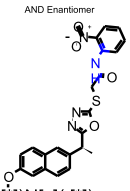 [*]C(=[*])N[c](:[*]): [*]	-0.486	12 out of 22

FCFP_12	-1724769936	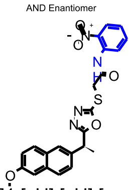 [*]N[c]1:[cH]:[cH]:[cH]:[cH]:1	-0.475	11 out of 20

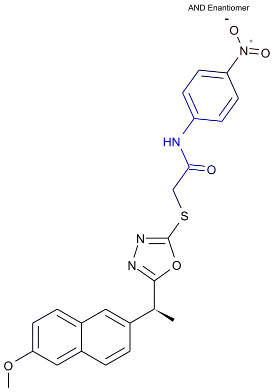

$C_{23}H_{20}N_4O_5S$

Molecular Weight: 464.4937

ALogP: 4.132

Rotatable Bonds: 8

Acceptors: 7

Donors: 1

Model Prediction

Prediction: Non-Irritant

Probability: 0.623

Enrichment: 0.677

Bayesian Score: -4.196

Mahalanobis Distance: 9.768

Mahalanobis Distance p-value: 0.11

Prediction: Positive if the Bayesian score is above the estimated best cutoff value from minimizing the false positive and false negative rate.
Probability: The esimated probability that the sample is in the positive category. This assumes that the Bayesian score follows a normal distribution and is different from the prediction using a cutoff.
Enrichment: An estimate of enrichment, that is, the increased likelihood (versus random) of this sample being in the category.
Bayesian Score: The standard Laplacian-modified Bayesian score.
Mahalanobis Distance: The Mahalanobis distance (MD) is the distance to the center of the training data. The larger the MD, the less trustworthy the prediction.
Mahalanobis Distance p-value: The p-value gives the fraction of training data with an MD greater than or equal to the one for the given sample, assuming normally distributed data. The smaller the p-value, the less trustworthy the prediciton. For highly non-normal X properties (e.g., fingerprints), the MD p-value is wildly inaccurate.

Structural Similar Compounds

Name	Pregna-1,4-diene-3,20-dione, 21-(acetyloxy)-11-hydroxy-6-methyl-17- (1-oxopropoxy)-, (6-alpha,11-beta)-	Benzenesulfonic acid, 2,2'-(4,4'-biphenylylenedivinylene)di-, disod ium salt	2-Anthracenesulfonic acid, 1-amino-9,10-dihydro-9,10-dioxo-4-(2,4,6 -trimethylanilino)-, monosodium salt
Structure	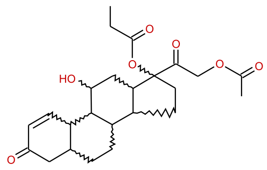	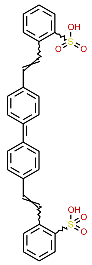	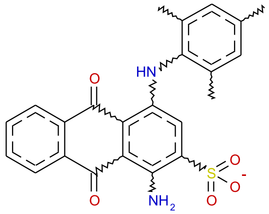
Actual Endpoint	Irritant	Irritant	Irritant
Predicted Endpoint	Irritant	Non-Irritant	Non-Irritant
Distance	0.803	0.817	0.834
Reference	YACHDS Yakuri to Chiryo. Pharmacology and Therapeutics. (Raifu Saiensu Shup pan K.K., 2-5-13, Yaesu, Chuo-ku, Tokyo 104, Japan) V.1- 1972- Volume(issue) /page/year: 19,3103,1991	MVCRB3 MVC-Report. (Stockholm, Sweden) No.1-2, 1972-73. Discontinued. Volu me(issue)/page/year: 2,193,1973	85JCAE "Prehled Prumyslove Toxikologie; Organicke Latky," Marhold, J., Prague , Czechoslovakia, Avicenum, 1986 Volume(issue)/page/year: -,1327,1986

Model Applicability

Unknown features are fingerprint features in the query molecule, but not found in the training set.

1. All properties and OPS components are within expected ranges.

Feature Contribution

Top features for positive contribution				
**Fingerprint**	**Bit/Smiles**	**Feature Structure**	**Score**	**Irritant in training set**

FCFP_12	5	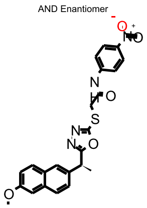 [*][O-]	0.085	27 out of 27
FCFP_12	8	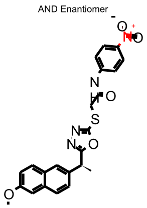 [*][N+](=[*])[*]	0.084	20 out of 20
FCFP_12	-828984032	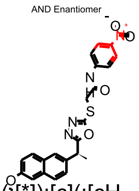 [*][c](:[*]):[c](:[cH]:[*])[N+](=[*])[*]	0.079	9 out of 9

Top Features for negative contribution

**Fingerprint**	**Bit/Smiles**	**Feature Structure**	**Score**	**Irritant in training set**
FCFP_12	1175665944	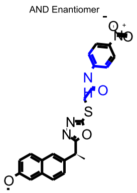 [*]CC(=O)N[c](:[cH]:[*]):[cH]:[*]	-1.020	2 out of 8
FCFP_12	-1838187238	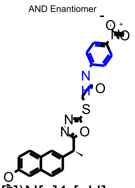 [*]C(=[*])N[c]1:[cH]:[cH]:[*]:[cH]:[cH]:1	-0.692	5 out of 12

FCFP_12	-451043714	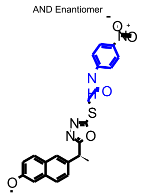 [*]CC(=O)N[c]1:[cH]:[cH]:[cH]:[cH]:[cH]:1	-0.650	0 out of 1

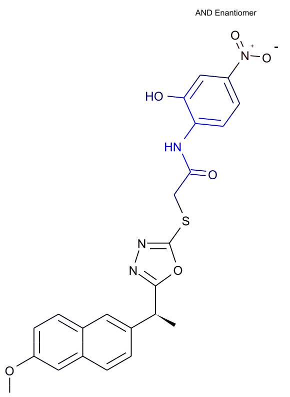

$C_{23}H_{20}N_4O_6S$

Molecular Weight: 480.4931

ALogP: 3.89

Rotatable Bonds: 8

Acceptors: 8

Donors: 2

Structural Similar Compounds

Name	2,2'-Stilbenedisulfonic acid, 4,4'-dinitro-	2-Anthracenesulfonic acid, 1-amino-9,10-dihydro-9,10-dioxo-4-(2,4,6 -trimethylanilino)-, monosodium salt	Urea, 1,3-bis(2-benzothiazolylthiomethyl)-
Structure	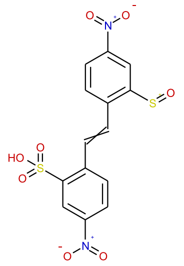	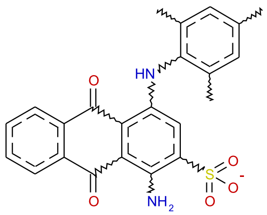	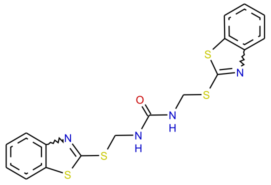
Actual Endpoint	Irritant	Irritant	Irritant
Predicted Endpoint	Irritant	Non-Irritant	Irritant
Distance	0.766	0.822	0.837
Reference	85JCAE "Prehled Prumyslove Toxikologie; Organicke Latky," Marhold, J., Prague , Czechoslovakia, Avicenum, 1986 Volume(issue)/page/year: -,1062,1986	85JCAE "Prehled Prumyslove Toxikologie; Organicke Latky," Marhold, J., Prague , Czechoslovakia, Avicenum, 1986 Volume(issue)/page/year: -,1327,1986	AMIHBC AMA Archives of Industrial Hygiene and Occupational Medicine. (Chicago , IL) V.2-10, 1950-54. For publisher information, see AEHLAU. Volume(issue)/pag e/year: 5,311,1952

Model Prediction

Prediction: Non-Irritant

Probability: 0.202

Enrichment: 0.219

Bayesian Score: -5.402

Mahalanobis Distance: 11.068

Mahalanobis Distance p-value: 0.0019

Prediction: Positive if the Bayesian score is above the estimated best cutoff value from minimizing the false positive and false negative rate.
Probability: The esimated probability that the sample is in the positive category. This assumes that the Bayesian score follows a normal distribution and is different from the prediction using a cutoff.
Enrichment: An estimate of enrichment, that is, the increased likelihood (versus random) of this sample being in the category.
Bayesian Score: The standard Laplacian-modified Bayesian score.
Mahalanobis Distance: The Mahalanobis distance (MD) is the distance to the center of the training data. The larger the MD, the less trustworthy the prediction.
Mahalanobis Distance p-value: The p-value gives the fraction of training data with an MD greater than or equal to the one for the given sample, assuming normally distributed data. The smaller the p-value, the less trustworthy the prediciton. For highly non-normal X properties (e.g., fingerprints), the MD p-value is wildly inaccurate.

Model Applicability

Unknown features are fingerprint features in the query molecule, but not found in the training set.

1. All properties and OPS components are within expected ranges.

Feature Contribution

Top features for positive contribution				
**Fingerprint**	**Bit/Smiles**	**Feature Structure**	**Score**	**Irritant in training set**

FCFP_12	5	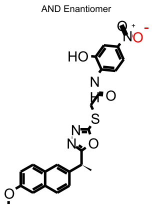 [*][O-]	0.085	27 out of 27
FCFP_12	8	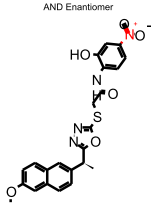 [*][N+](=[*])[*]	0.084	20 out of 20
FCFP_12	-828984032	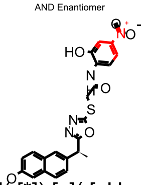 [*][c](:[*]):[c](:[cH]:[*])[N+](=[*])[*]	0.079	9 out of 9
**Top Features for negative contribution**				
**Fingerprint**	**Bit/Smiles**	**Feature Structure**	**Score**	**Irritant in training set**
FCFP_12	1175665944	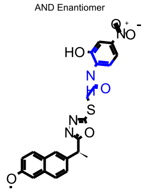 [*]CC(=O)N[c](:[cH]:[*]):[cH]:[*]	-1.020	2 out of 8
FCFP_12	-1924607822	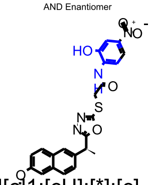 [*]N[c]1:[cH]:[*]:[c] ([*]):[cH]:[c]:1O	-0.650	0 out of 1

FCFP_12	-1883332927	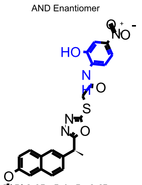<br>[*]C(=[*])N[c]1:[cH]:[cH]:[*]:[cH]:[c]:1O	-0.650	0 out of 1

Sorafenib

TOPKAT_Skin_Irritancy_None_vs_Irritant

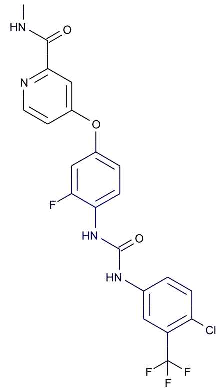

$C_{21}H_{15}ClF_4N_4O_3$
Molecular Weight: 482.81541
ALogP: 4.381
Rotatable Bonds: 6
Acceptors: 4
Donors: 3

Model Prediction

Prediction: Non-Irritant
Probability: 0.622
Enrichment: 0.676
Bayesian Score: -4.199
Mahalanobis Distance: 8.385
Mahalanobis Distance p-value: 0.742

Prediction: Positive if the Bayesian score is above the estimated best cutoff value from minimizing the false positive and false negative rate.
Probability: The esimated probability that the sample is in the positive category. This assumes that the Bayesian score follows a normal distribution and is different from the prediction using a cutoff.
Enrichment: An estimate of enrichment, that is, the increased likelihood (versus random) of this sample being in the category.
Bayesian Score: The standard Laplacian-modified Bayesian score.
Mahalanobis Distance: The Mahalanobis distance (MD) is the distance to the center of the training data. The larger the MD, the less trustworthy the prediction.
Mahalanobis Distance p-value: The p-value gives the fraction of training data with an MD greater than or equal to the one for the given sample, assuming normally distributed data. The smaller the p-value, the less trustworthy the prediciton. For highly non-normal X properties (e.g., fingerprints), the MD p-value is wildly inaccurate.

Structural Similar Compounds

Name	Benzenesulfonic acid, 2,2'-(4,4'-biphenylylenedivinylene)di-, disod ium salt	5-Norbornene-2,3-dicarboxylic acid, 1,4,5,6,7,7-hexachloro-	Sulfide, bis(4-t-butyl-m-cresyl)-
Structure	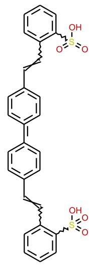	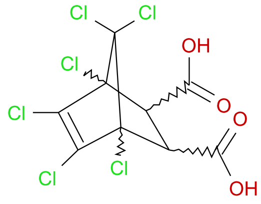	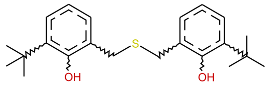
Actual Endpoint	Irritant	Irritant	Irritant
Predicted Endpoint	Non-Irritant	Irritant	Irritant
Distance	0.856	0.871	0.897
Reference	MVCRB3 MVC-Report. (Stockholm, Sweden) No.1-2, 1972-73. Discontinued. Volu me(issue)/page/year: 2,193,1973	85JCAE "Prehled Prumyslove Toxikologie; Organicke Latky," Marhold, J., Prague , Czechoslovakia, Avicenum, 1986 Volume(issue)/page/year: -,581,1986	AMIHBC AMA Archives of Industrial Hygiene and Occupational Medicine. (Chicago , IL) V.2-10, 1950-54. For publisher information, see AEHLAU. Volume(issue)/pag e/year: 5,311,1952

Model Applicability

Unknown features are fingerprint features in the query molecule, but not found in the training set.

1. All properties and OPS components are within expected ranges.

Feature Contribution

Top features for positive contribution				
Fingerprint	Bit/Smiles	Feature Structure	Score	Irritant in training set

FCFP_12	-124655670	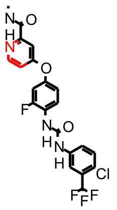 [*]:[cH]:[cH]:n:[*]	0.082	13 out of 13
FCFP_12	-1539132615	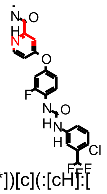 [*]C(=[*])[c](:[cH]:[*]):n:[*]	0.079	9 out of 9
FCFP_12	-1695756380	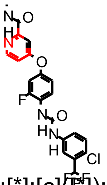 [*][c]1:[*]:[c]([*]):n:[cH]:[cH]:1	0.077	7 out of 7
**Top Features for negative contribution**				
**Fingerprint**	**Bit/Smiles**	**Feature Structure**	**Score**	**Irritant in training set**
FCFP_12	-1838187238	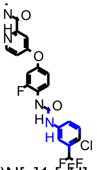 [*]C(=[*])N[c]1:[cH]:[cH]:[*]:[cH]:[cH]:1	-0.692	5 out of 12
FCFP_12	1783756416	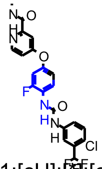 [*]N[c]1:[cH]:[*]:[c]([*]):[cH]:[c]:1Cl	-0.509	4 out of 8

FCFP_12	1294255210	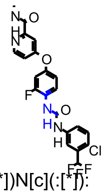 [*]C(=[*])N[c](:[*]):[*]	-0.486	12 out of 22

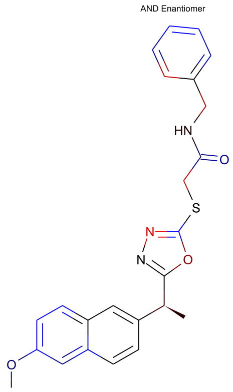

$C_{24}H_{23}N_3O_3S$
Molecular Weight: 433.52272
ALogP: 4.245
Rotatable Bonds: 8
Acceptors: 5
Donors: 1

Model Prediction

Prediction: 15.349
Unit: mg/kg_body_weight/day
Mahalanobis Distance: 14.115
Mahalanobis Distance p-value: 1.57e-010

Mahalanobis Distance: The Mahalanobis distance (MD) is a generalization of the Euclidean distance that accounts for correlations among the X properties. It is calculated as the distance to the center of the training data. The larger the MD, the less trustworthy the prediction.
Mahalanobis Distance p-value: The p-value gives the fraction of training data with an MD greater than or equal to the one for the given sample, assuming normally distributed data. The smaller the p-value, the less trustworthy the prediciton. For highly non-normal X properties (e.g., fingerprints), the MD p-value is wildly inaccurate.

Structural Similar Compounds

Name	646	Tamoxifen citrate	Phenolphthalein
Structure	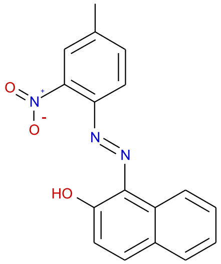	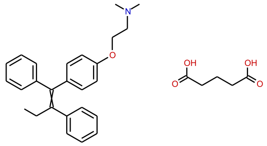	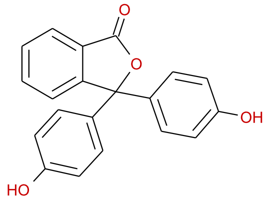
Actual Endpoint (-log C)	0.937339	5.05965	2.43468
Predicted Endpoint (-log C)	3.26294	4.24168	3.66084
Distance	0.812	0.856	0.868
Reference	CPDB	CPDB	CPDB

Model Applicability

Unknown features are fingerprint features in the query molecule, but not found in the training set.

1. All properties and OPS components are within expected ranges.
2. Unknown ECFP_2 feature: 1093109320: [*]S[c]1:o:[*]:[*]:n:1
3. Unknown ECFP_2 feature: -1841325949: [*]:[c](:[*])C(C)[c](:[*]):[*]
4. Unknown ECFP_2 feature: 1092541557: [*]C([*])[c]1:o:[*]:[*]:n:1

Feature Contribution

Top features for positive contribution			
**Fingerprint**	**Bit/Smiles**	**Feature Structure**	**Score**
ECFP_6	655739385	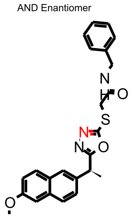 [*]:n:[*]	0.229

ECFP_6	1559650422	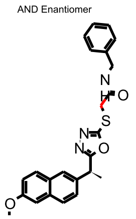 [*]C[*]	0.203
ECFP_6	-2024255407	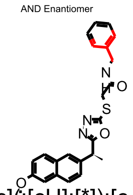 [*]C[c](:[cH]:[*]):[cH]:[*]	0.172
**Top Features for negative contribution**			
**Fingerprint**	**Bit/Smiles**	**Feature Structure**	**Score**
ECFP_6	1996767644	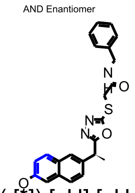 [*][c](:[*]):[cH]:[cH]:[*]	-0.251
ECFP_6	642810091	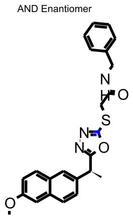 [*][c](:[*]):[*]	-0.247
ECFP_6	-182236392	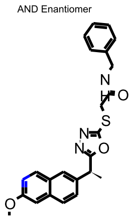 [*]:[cH]:[*]	-0.232

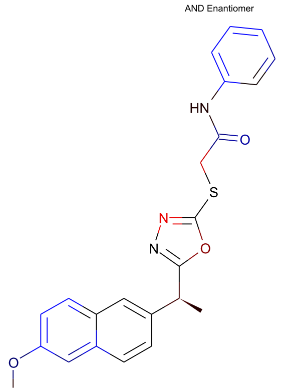

$C_{23}H_{21}N_3O_3S$
Molecular Weight: 419.49614
ALogP: 4.238
Rotatable Bonds: 7
Acceptors: 5
Donors: 1

Model Prediction

Prediction: 20.304
Unit: mg/kg_body_weight/day
Mahalanobis Distance: 14.265
Mahalanobis Distance p-value: 6.06e-011

Mahalanobis Distance: The Mahalanobis distance (MD) is a generalization of the Euclidean distance that accounts for correlations among the X properties. It is calculated as the distance to the center of the training data. The larger the MD, the less trustworthy the prediction.
Mahalanobis Distance p-value: The p-value gives the fraction of training data with an MD greater than or equal to the one for the given sample, assuming normally distributed data. The smaller the p-value, the less trustworthy the prediciton. For highly non-normal X properties (e.g., fingerprints), the MD p-value is wildly inaccurate.

Structural Similar Compounds

Name	646	Phenolphthalein	Acifluorfen
Structure	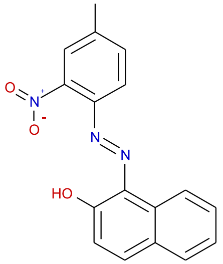	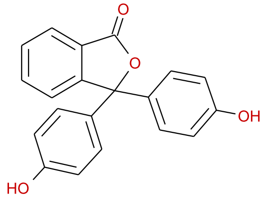	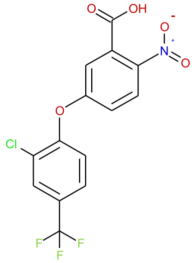
Actual Endpoint (-log C)	0.937339	2.43468	3.40908
Predicted Endpoint (-log C)	3.26294	3.66084	3.10974
Distance	0.773	0.829	0.869
Reference	CPDB	CPDB	CPDB

Model Applicability

Unknown features are fingerprint features in the query molecule, but not found in the training set.

1. All properties and OPS components are within expected ranges.
2. Unknown ECFP_2 feature: 1093109320: [*]S[c]1:o:[*]:[*]:n:1
3. Unknown ECFP_2 feature: -1841325949: [*]:[c](:[*])C(C)[c](:[*]):[*]
4. Unknown ECFP_2 feature: 1092541557: [*]C([*])[c]1:o:[*]:[*]:n:1

Feature Contribution

Top features for positive contribution			
**Fingerprint**	**Bit/Smiles**	**Feature Structure**	**Score**
ECFP_6	655739385	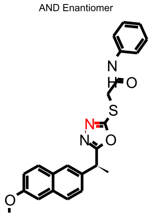 [*]:n:[*]	0.229

ECFP_6	1559650422	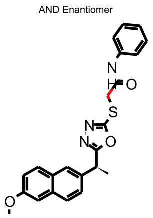 [*]C[*]	0.203
ECFP_6	683445015	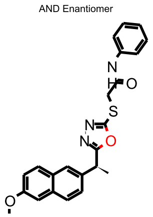 [*]:o:[*]	0.136

Top Features for negative contribution

Fingerprint	Bit/Smiles	Feature Structure	Score
ECFP_6	1996767644	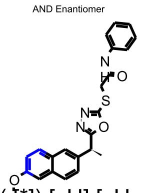 [*][c](:[*]):[cH]:[cH]:[*]	-0.251
ECFP_6	642810091	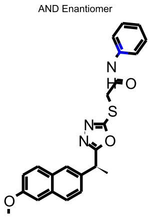 [*][c](:[*]):[*]	-0.247
ECFP_6	-182236392	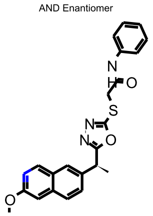 [*]:[cH]:[*]	-0.232

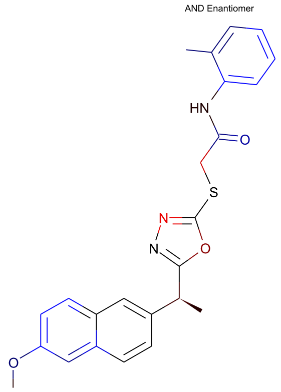

$C_{24}H_{23}N_3O_3S$
Molecular Weight: 433.52272
ALogP: 4.724
Rotatable Bonds: 7
Acceptors: 5
Donors: 1

Model Prediction

Prediction: 23.695
Unit: mg/kg_body_weight/day
Mahalanobis Distance: 16.091
Mahalanobis Distance p-value: 1.76e-016

Mahalanobis Distance: The Mahalanobis distance (MD) is a generalization of the Euclidean distance that accounts for correlations among the X properties. It is calculated as the distance to the center of the training data. The larger the MD, the less trustworthy the prediction.
Mahalanobis Distance p-value: The p-value gives the fraction of training data with an MD greater than or equal to the one for the given sample, assuming normally distributed data. The smaller the p-value, the less trustworthy the prediciton. For highly non-normal X properties (e.g., fingerprints), the MD p-value is wildly inaccurate.

Structural Similar Compounds

Name	646	Phenolphthalein	[4-Chloro-6-(2,3-xylidino)-2-pyri-midinylthio]acetic acid s
Structure	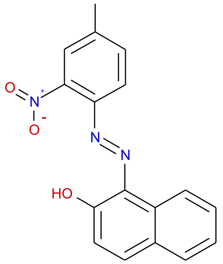	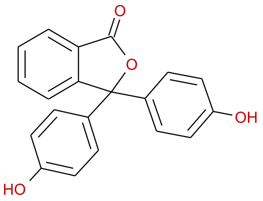	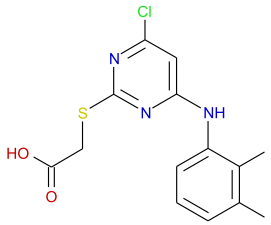
Actual Endpoint (-log C)	0.937339	2.43468	4.47685
Predicted Endpoint (-log C)	3.26294	3.66084	3.8529
Distance	0.772	0.839	0.861
Reference	CPDB	CPDB	CPDB

Model Applicability

Unknown features are fingerprint features in the query molecule, but not found in the training set.

1. All properties and OPS components are within expected ranges.
2. Unknown ECFP_2 feature: 1093109320: [*]S[c]1:o:[*]:[*]:n:1
3. Unknown ECFP_2 feature: -1841325949: [*]:[c](:[*])C(C)[c](:[*]):[*]
4. Unknown ECFP_2 feature: 1092541557: [*]C([*])[c]1:o:[*]:[*]:n:1

Feature Contribution

Top features for positive contribution

Fingerprint	Bit/Smiles	Feature Structure	Score
ECFP_6	655739385	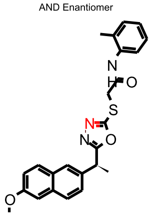 [*]:n:[*]	0.229

ECFP_6	1559650422	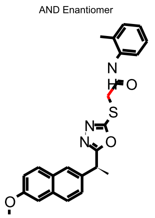 [*]C[*]	0.203
ECFP_6	683445015	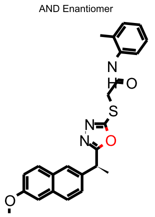 [*]:o:[*]	0.136
**Top Features for negative contribution**			
**Fingerprint**	**Bit/Smiles**	**Feature Structure**	**Score**
ECFP_6	1996767644	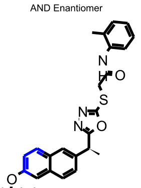 [*][c](:[*]):[cH]:[cH]:[*]	-0.251
ECFP_6	642810091	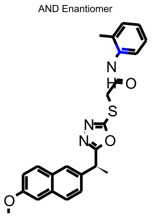 [*][c](:[*]):[*]	-0.247
ECFP_6	-182236392	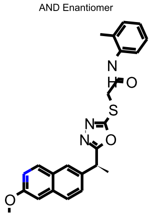 [*]:[cH]:[*]	-0.232

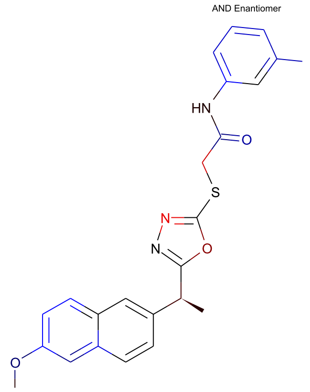

$C_{24}H_{23}N_3O_3S$
Molecular Weight: 433.52272
ALogP: 4.724
Rotatable Bonds: 7
Acceptors: 5
Donors: 1

Model Prediction

Prediction: 18.535
Unit: mg/kg_body_weight/day
Mahalanobis Distance: 15.784
Mahalanobis Distance p-value: 1.7e-015

Mahalanobis Distance: The Mahalanobis distance (MD) is a generalization of the Euclidean distance that accounts for correlations among the X properties. It is calculated as the distance to the center of the training data. The larger the MD, the less trustworthy the prediction.
Mahalanobis Distance p-value: The p-value gives the fraction of training data with an MD greater than or equal to the one for the given sample, assuming normally distributed data. The smaller the p-value, the less trustworthy the prediciton. For highly non-normal X properties (e.g., fingerprints), the MD p-value is wildly inaccurate.

Structural Similar Compounds

Name	646	Phenolphthalein	Acifluorfen
Structure	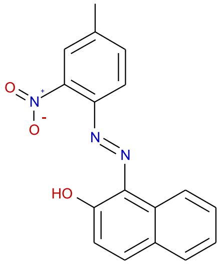	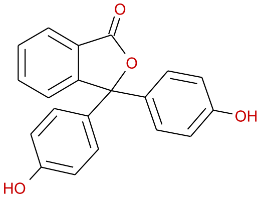	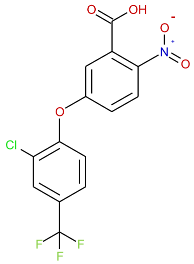
Actual Endpoint (-log C)	0.937339	2.43468	3.40908
Predicted Endpoint (-log C)	3.26294	3.66084	3.10974
Distance	0.768	0.843	0.873
Reference	CPDB	CPDB	CPDB

Model Applicability

Unknown features are fingerprint features in the query molecule, but not found in the training set.

1. All properties and OPS components are within expected ranges.
2. Unknown ECFP_2 feature: 1093109320: [*]S[c]1:o:[*]:[*]:n:1
3. Unknown ECFP_2 feature: -1841325949: [*]:[c](:[*])C(C)[c](:[*]):[*]
4. Unknown ECFP_2 feature: 1092541557: [*]C([*])[c]1:o:[*]:[*]:n:1

Feature Contribution

Top features for positive contribution			
Fingerprint	Bit/Smiles	Feature Structure	Score
ECFP_6	655739385	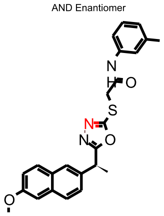 [*]:n:[*]	0.229

ECFP_6	1559650422	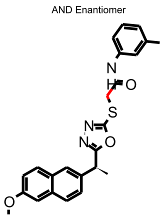 [*]C[*]	0.203
ECFP_6	683445015	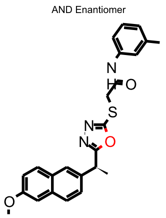 [*]:o:[*]	0.136
**Top Features for negative contribution**			
**Fingerprint**	**Bit/Smiles**	**Feature Structure**	**Score**
ECFP_6	1996767644	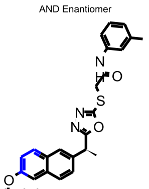 [*][c](:[*]):[cH]:[cH]:[*]	-0.251
ECFP_6	642810091	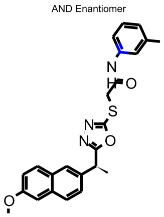 [*][c](:[*]):[*]	-0.247
ECFP_6	-182236392	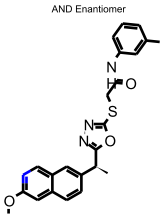 [*]:[cH]:[*]	-0.232

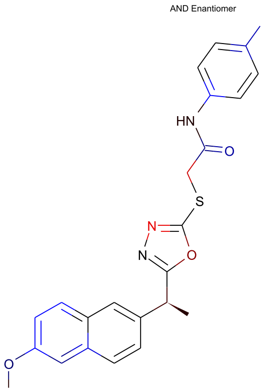

$C_{24}H_{23}N_3O_3S$
Molecular Weight: 433.52272
ALogP: 4.724
Rotatable Bonds: 7
Acceptors: 5
Donors: 1

Model Prediction

Prediction: 11.722
Unit: mg/kg_body_weight/day
Mahalanobis Distance: 15.585
Mahalanobis Distance p-value: 7.22e-015

Mahalanobis Distance: The Mahalanobis distance (MD) is a generalization of the Euclidean distance that accounts for correlations among the X properties. It is calculated as the distance to the center of the training data. The larger the MD, the less trustworthy the prediction.
Mahalanobis Distance p-value: The p-value gives the fraction of training data with an MD greater than or equal to the one for the given sample, assuming normally distributed data. The smaller the p-value, the less trustworthy the prediciton. For highly non-normal X properties (e.g., fingerprints), the MD p-value is wildly inaccurate.

Structural Similar Compounds

Name	646	Phenolphthalein	Acifluorfen
Structure	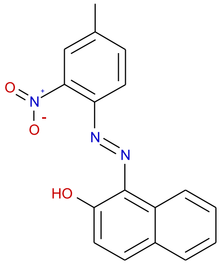	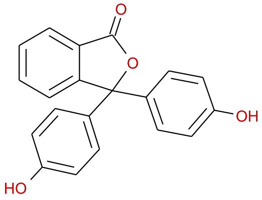	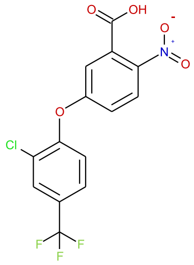
Actual Endpoint (-log C)	0.937339	2.43468	3.40908
Predicted Endpoint (-log C)	3.26294	3.66084	3.10974
Distance	0.771	0.847	0.872
Reference	CPDB	CPDB	CPDB

Model Applicability

Unknown features are fingerprint features in the query molecule, but not found in the training set.

1. All properties and OPS components are within expected ranges.
2. Unknown ECFP_2 feature: 1093109320: [*]S[c]1:o:[*]:[*]:n:1
3. Unknown ECFP_2 feature: -1841325949: [*]:[c](:[*])C(C)[c](:[*]):[*]
4. Unknown ECFP_2 feature: 1092541557: [*]C([*])[c]1:o:[*]:[*]:n:1

Feature Contribution

Top features for positive contribution			
Fingerprint	Bit/Smiles	Feature Structure	Score
ECFP_6	655739385	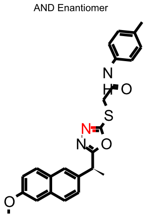 [*]:n:[*]	0.229

ECFP_6	1559650422	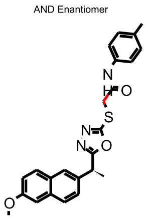 [*]C[*]	0.203
ECFP_6	683445015	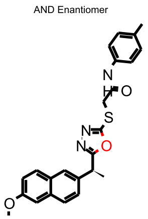 [*]:o:[*]	0.136
**Top Features for negative contribution**			
**Fingerprint**	**Bit/Smiles**	**Feature Structure**	**Score**
ECFP_6	1996767644	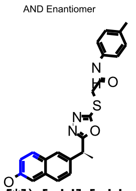 [*][c](:[*]):[cH]:[cH]:[*]	-0.251
ECFP_6	642810091	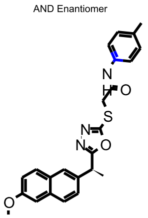 [*][c](:[*]):[*]	-0.247
ECFP_6	-182236392	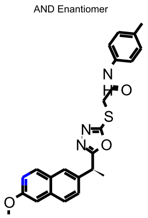 [*]:[cH]:[*]	-0.232

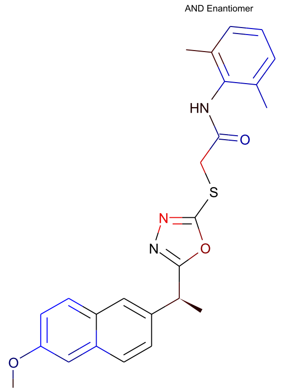

$C_{25}H_{25}N_3O_3S$
Molecular Weight: 447.5493
ALogP: 5.21
Rotatable Bonds: 7
Acceptors: 5
Donors: 1

Model Prediction

Prediction: 13.868
Unit: mg/kg_body_weight/day
Mahalanobis Distance: 16.006
Mahalanobis Distance p-value: 3.3e-016

Mahalanobis Distance: The Mahalanobis distance (MD) is a generalization of the Euclidean distance that accounts for correlations among the X properties. It is calculated as the distance to the center of the training data. The larger the MD, the less trustworthy the prediction.
Mahalanobis Distance p-value: The p-value gives the fraction of training data with an MD greater than or equal to the one for the given sample, assuming normally distributed data. The smaller the p-value, the less trustworthy the prediciton. For highly non-normal X properties (e.g., fingerprints), the MD p-value is wildly inaccurate.

Structural Similar Compounds

Name	646	Phenolphthalein	[4-Chloro-6-(2,3-xylidino)-2-pyri-midinylthio]acetic acid s
Structure	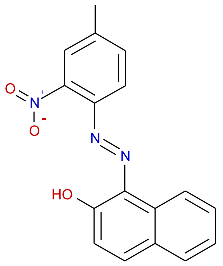	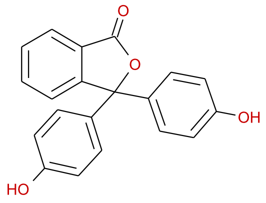	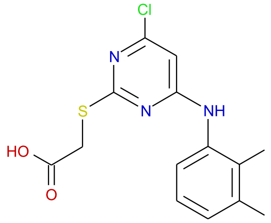
Actual Endpoint (-log C)	0.937339	2.43468	4.47685
Predicted Endpoint (-log C)	3.26294	3.66084	3.8529
Distance	0.782	0.855	0.872
Reference	CPDB	CPDB	CPDB

Model Applicability

Unknown features are fingerprint features in the query molecule, but not found in the training set.

1. All properties and OPS components are within expected ranges.
2. Unknown ECFP_2 feature: -1660205591: [*]N[c](:[c]([*]):[*]):[c]([*]):[*]
3. Unknown ECFP_2 feature: 1093109320: [*]S[c]1:o:[*]:[*]:n:1
4. Unknown ECFP_2 feature: -1841325949: [*]:[c](:[*])C(C)[c](:[*]):[*]
5. Unknown ECFP_2 feature: 1092541557: [*]C([*])[c]1:o:[*]:[*]:n:1

Feature Contribution

Top features for positive contribution			
Fingerprint	Bit/Smiles	Feature Structure	Score
ECFP_6	655739385	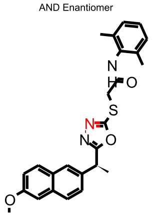 [*]:n:[*]	0.229

ECFP_6	1559650422	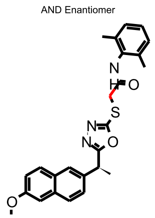 [*]C[*]	0.203
ECFP_6	683445015	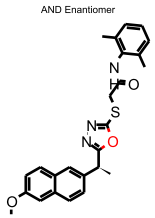 [*]:o:[*]	0.136

Top Features for negative contribution

Fingerprint	Bit/Smiles	Feature Structure	Score
ECFP_6	1996767644	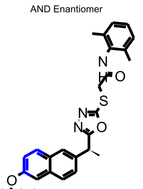 [*][c](:[*]):[cH]:[cH]:[*]	-0.251
ECFP_6	642810091	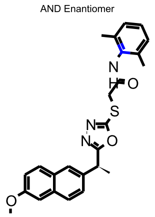 [*][c](:[*]):[*]	-0.247
ECFP_6	-182236392	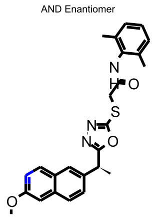 [*]:[cH]:[*]	-0.232

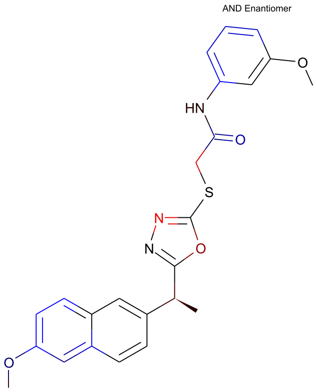

$C_{24}H_{23}N_3O_4S$
Molecular Weight: 449.52212
ALogP: 4.221
Rotatable Bonds: 8
Acceptors: 6
Donors: 1

Model Prediction

Prediction: 14.625
Unit: mg/kg_body_weight/day
Mahalanobis Distance: 14.185
Mahalanobis Distance p-value: 1.01e-010

Mahalanobis Distance: The Mahalanobis distance (MD) is a generalization of the Euclidean distance that accounts for correlations among the X properties. It is calculated as the distance to the center of the training data. The larger the MD, the less trustworthy the prediction.
Mahalanobis Distance p-value: The p-value gives the fraction of training data with an MD greater than or equal to the one for the given sample, assuming normally distributed data. The smaller the p-value, the less trustworthy the prediciton. For highly non-normal X properties (e.g., fingerprints), the MD p-value is wildly inaccurate.

Structural Similar Compounds

Name	646	Tamoxifen citrate	[4-Chloro-6-(2,3-xylidino)-2-pyri-midinylthio]acetic acid s
Structure	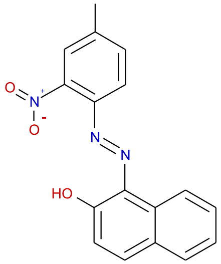	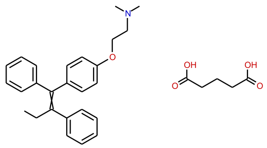	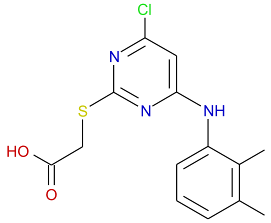
Actual Endpoint (-log C)	0.937339	5.05965	4.47685
Predicted Endpoint (-log C)	3.26294	4.24168	3.8529
Distance	0.835	0.864	0.894
Reference	CPDB	CPDB	CPDB

Model Applicability

Unknown features are fingerprint features in the query molecule, but not found in the training set.

1. All properties and OPS components are within expected ranges.
2. Unknown ECFP_2 feature: 1093109320: [*]S[c]1:o:[*]:[*]:n:1
3. Unknown ECFP_2 feature: -1841325949: [*]:[c](:[*])C(C)[c](:[*]):[*]
4. Unknown ECFP_2 feature: 1092541557: [*]C([*])[c]1:o:[*]:[*]:n:1

Feature Contribution

Top features for positive contribution

Fingerprint	Bit/Smiles	Feature Structure	Score
ECFP_6	655739385	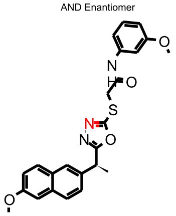 [*]:n:[*]	0.229

ECFP_6	1559650422	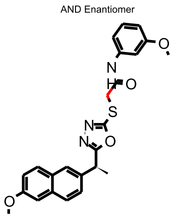 [*]C[*]	0.203
ECFP_6	683445015	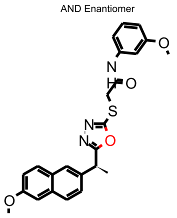 [*]:o:[*]	0.136

Top Features for negative contribution

Fingerprint	Bit/Smiles	Feature Structure	Score
ECFP_6	1996767644	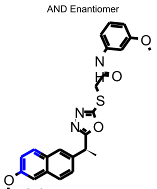 [*][c](:[*]):[cH]:[cH]:[*]	-0.251
ECFP_6	642810091	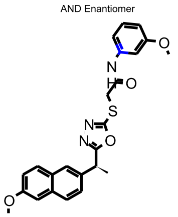 [*][c](:[*]):[*]	-0.247
ECFP_6	-182236392	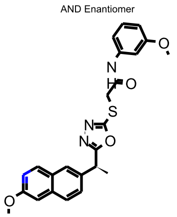 [*]:[cH]:[*]	-0.232

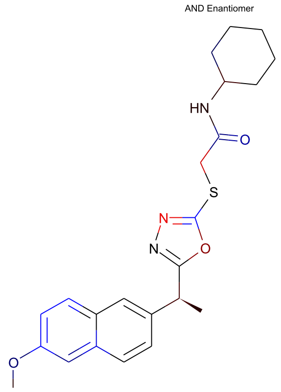

$C_{23}H_{27}N_3O_3S$

Molecular Weight: 425.54378

ALogP: 4.52

Rotatable Bonds: 7

Acceptors: 5

Donors: 1

Model Prediction

Prediction: 9.203

Unit: mg/kg_body_weight/day

Mahalanobis Distance: 13.886

Mahalanobis Distance p-value: 6.66e-010

Mahalanobis Distance: The Mahalanobis distance (MD) is a generalization of the Euclidean distance that accounts for correlations among the X properties. It is calculated as the distance to the center of the training data. The larger the MD, the less trustworthy the prediction.
Mahalanobis Distance p-value: The p-value gives the fraction of training data with an MD greater than or equal to the one for the given sample, assuming normally distributed data. The smaller the p-value, the less trustworthy the prediciton. For highly non-normal X properties (e.g., fingerprints), the MD p-value is wildly inaccurate.

Structural Similar Compounds

Name	646	Acifluorfen	C.I. pigment red 3
Structure	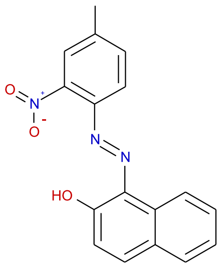	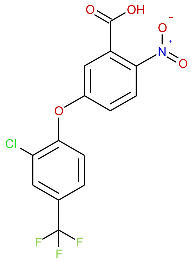	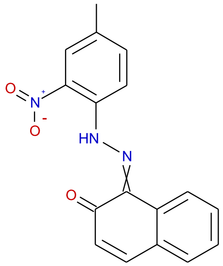
Actual Endpoint (-log C)	0.937339	3.40908	0.937339
Predicted Endpoint (-log C)	3.26294	3.10974	3.17837
Distance	0.739	0.766	0.786
Reference	CPDB	CPDB	CPDB

Model Applicability

Unknown features are fingerprint features in the query molecule, but not found in the training set.

1. All properties and OPS components are within expected ranges.
2. Unknown ECFP_2 feature: 1093109320: [*]S[c]1:o:[*]:[*]:n:1
3. Unknown ECFP_2 feature: -1841325949: [*]:[c](:[*])C(C)[c](:[*]):[*]
4. Unknown ECFP_2 feature: 1092541557: [*]C([*])[c]1:o:[*]:[*]:n:1

Feature Contribution

Top features for positive contribution

Fingerprint	Bit/Smiles	Feature Structure	Score
ECFP_6	655739385	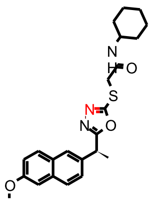 AND Enantiomer [*]:n:[*]	0.229

ECFP_6	1559650422	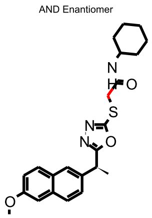 [*]C[*]	0.203
ECFP_6	683445015	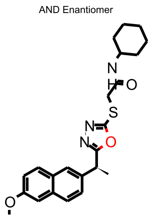 [*]:o:[*]	0.136
**Top Features for negative contribution**			
**Fingerprint**	**Bit/Smiles**	**Feature Structure**	**Score**
ECFP_6	1996767644	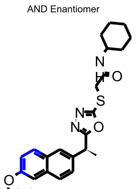 [*][c](:[*]):[cH]:[cH]:[*]	-0.251
ECFP_6	642810091	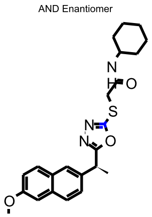 [*][c](:[*]):[*]	-0.247
ECFP_6	-182236392	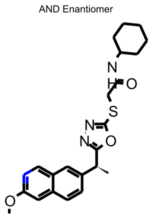 [*]:[cH]:[*]	-0.232

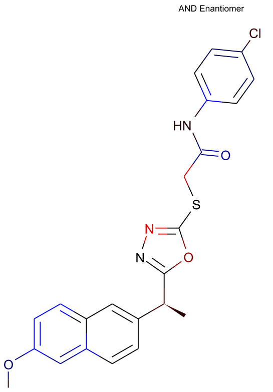

$C_{23}H_{20}ClN_3O_3S$

Molecular Weight: 453.9412

ALogP: 4.902

Rotatable Bonds: 7

Acceptors: 5

Donors: 1

Model Prediction

Prediction: 6.298

Unit: mg/kg_body_weight/day

Mahalanobis Distance: 14.892

Mahalanobis Distance p-value: 9.4e-013

Mahalanobis Distance: The Mahalanobis distance (MD) is a generalization of the Euclidean distance that accounts for correlations among the X properties. It is calculated as the distance to the center of the training data. The larger the MD, the less trustworthy the prediction.
Mahalanobis Distance p-value: The p-value gives the fraction of training data with an MD greater than or equal to the one for the given sample, assuming normally distributed data. The smaller the p-value, the less trustworthy the prediciton. For highly non-normal X properties (e.g., fingerprints), the MD p-value is wildly inaccurate.

Structural Similar Compounds

Name	646	Phenolphthalein	Acifluorfen
Structure	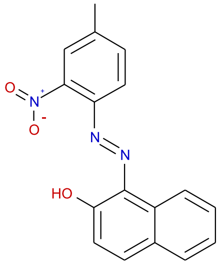	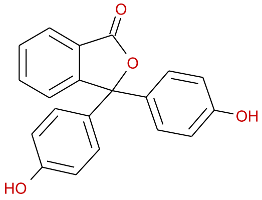	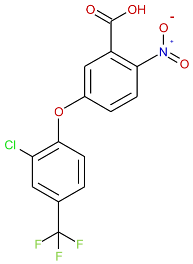
Actual Endpoint (-log C)	0.937339	2.43468	3.40908
Predicted Endpoint (-log C)	3.26294	3.66084	3.10974
Distance	0.795	0.859	0.870
Reference	CPDB	CPDB	CPDB

Model Applicability

Unknown features are fingerprint features in the query molecule, but not found in the training set.

1. All properties and OPS components are within expected ranges.
2. Unknown ECFP_2 feature: 1093109320: [*]S[c]1:o:[*]:[*]:n:1
3. Unknown ECFP_2 feature: -1841325949: [*]:[c](:[*])C(C)[c](:[*]):[*]
4. Unknown ECFP_2 feature: 1092541557: [*]C([*])[c]1:o:[*]:[*]:n:1

Feature Contribution

Top features for positive contribution			
**Fingerprint**	**Bit/Smiles**	**Feature Structure**	**Score**
ECFP_6	655739385	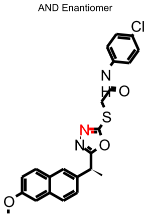 [*]:n:[*]	0.229

ECFP_6	1559650422	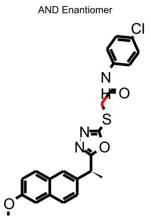 [*]C[*]	0.203
ECFP_6	683445015	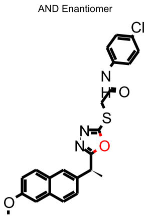 [*]:o:[*]	0.136
**Top Features for negative contribution**			
**Fingerprint**	**Bit/Smiles**	**Feature Structure**	**Score**
ECFP_6	1996767644	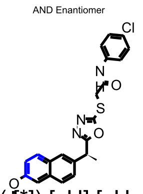 [*][c](:[*]):[cH]:[cH<br>]:[*]	-0.251
ECFP_6	642810091	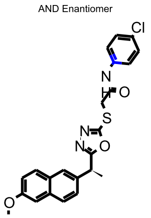 [*][c](:[*]):[*]	-0.247
ECFP_6	-182236392	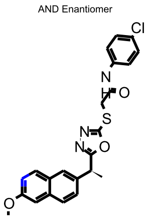 [*]:[cH]:[*]	-0.232

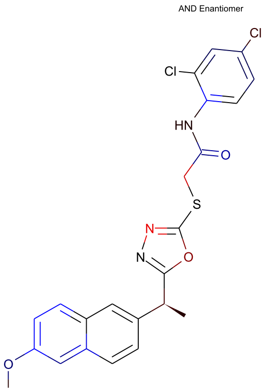

$C_{23}H_{19}Cl_2N_3O_3S$
Molecular Weight: 488.38626
ALogP: 5.567
Rotatable Bonds: 7
Acceptors: 5
Donors: 1

Model Prediction

Prediction: 4.964
Unit: mg/kg_body_weight/day
Mahalanobis Distance: 14.864
Mahalanobis Distance p-value: 1.13e-012

Mahalanobis Distance: The Mahalanobis distance (MD) is a generalization of the Euclidean distance that accounts for correlations among the X properties. It is calculated as the distance to the center of the training data. The larger the MD, the less trustworthy the prediction.
Mahalanobis Distance p-value: The p-value gives the fraction of training data with an MD greater than or equal to the one for the given sample, assuming normally distributed data. The smaller the p-value, the less trustworthy the prediciton. For highly non-normal X properties (e.g., fingerprints), the MD p-value is wildly inaccurate.

Structural Similar Compounds

Name	646	Acifluorfen	Phenolphthalein
Structure	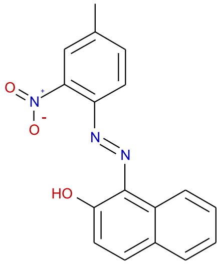	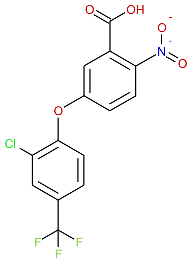	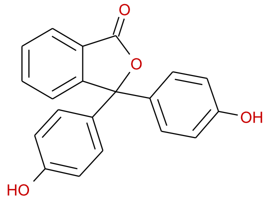
Actual Endpoint (-log C)	0.937339	3.40908	2.43468
Predicted Endpoint (-log C)	3.26294	3.10974	3.66084
Distance	0.819	0.885	0.888
Reference	CPDB	CPDB	CPDB

Model Applicability

Unknown features are fingerprint features in the query molecule, but not found in the training set.

1. All properties and OPS components are within expected ranges.
2. Unknown ECFP_2 feature: 1093109320: [*]S[c]1:o:[*]:[*]:n:1
3. Unknown ECFP_2 feature: -1841325949: [*]:[c](:[*])C(C)[c](:[*]):[*]
4. Unknown ECFP_2 feature: 1092541557: [*]C([*])[c]1:o:[*]:[*]:n:1

Feature Contribution

Top features for positive contribution			
**Fingerprint**	**Bit/Smiles**	**Feature Structure**	**Score**
ECFP_6	655739385	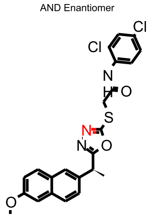 [*]:n:[*]	0.229

ECFP_6	1559650422	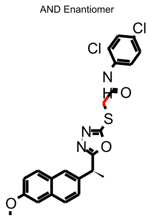 [*]C[*]	0.203
ECFP_6	683445015	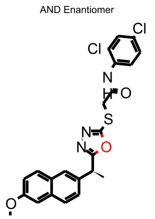 [*]:o:[*]	0.136
**Top Features for negative contribution**			
**Fingerprint**	**Bit/Smiles**	**Feature Structure**	**Score**
ECFP_6	1996767644	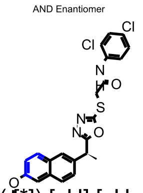 [*][c](:[*]):[cH]:[cH]:[*]	-0.251
ECFP_6	642810091	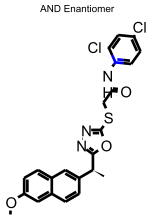 [*][c](:[*]):[*]	-0.247
ECFP_6	-182236392	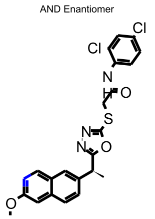 [*]:[cH]:[*]	-0.232

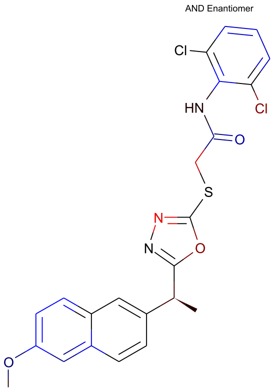

$C_{23}H_{19}Cl_2N_3O_3S$

Molecular Weight: 488.38626

ALogP: 5.567

Rotatable Bonds: 7

Acceptors: 5

Donors: 1

Model Prediction

Prediction: 6.520

Unit: mg/kg_body_weight/day

Mahalanobis Distance: 14.543

Mahalanobis Distance p-value: 9.8e-012

Mahalanobis Distance: The Mahalanobis distance (MD) is a generalization of the Euclidean distance that accounts for correlations among the X properties. It is calculated as the distance to the center of the training data. The larger the MD, the less trustworthy the prediction.

Mahalanobis Distance p-value: The p-value gives the fraction of training data with an MD greater than or equal to the one for the given sample, assuming normally distributed data. The smaller the p-value, the less trustworthy the prediciton. For highly non-normal X properties (e.g., fingerprints), the MD p-value is wildly inaccurate.

Structural Similar Compounds

Name	646	Phenolphthalein	Acifluorfen
Structure	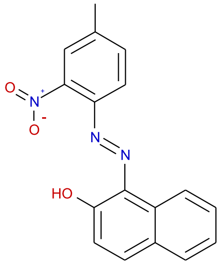	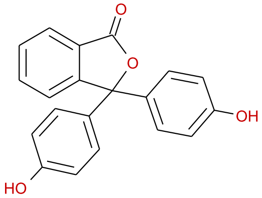	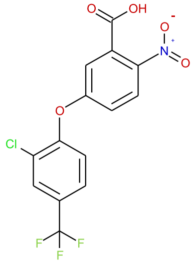
Actual Endpoint (-log C)	0.937339	2.43468	3.40908
Predicted Endpoint (-log C)	3.26294	3.66084	3.10974
Distance	0.814	0.883	0.884
Reference	CPDB	CPDB	CPDB

Model Applicability

Unknown features are fingerprint features in the query molecule, but not found in the training set.

1. All properties and OPS components are within expected ranges.
2. Unknown ECFP_2 feature: -1660205591: [*]N[c](:[c]([*]):[*]):[c]([*]):[*]
3. Unknown ECFP_2 feature: 1093109320: [*]S[c]1:o:[*]:[*]:n:1
4. Unknown ECFP_2 feature: -1841325949: [*]:[c](:[*])C(C)[c](:[*]):[*]
5. Unknown ECFP_2 feature: 1092541557: [*]C([*])[c]1:o:[*]:[*]:n:1

Feature Contribution

Top features for positive contribution

Fingerprint	Bit/Smiles	Feature Structure	Score
ECFP_6	655739385	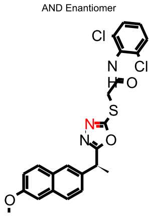 [*]:n:[*]	0.229

ECFP_6	1559650422	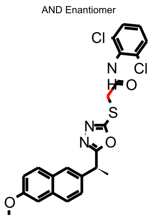 [*]C[*]	0.203
ECFP_6	683445015	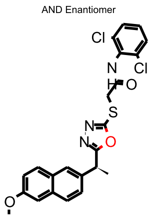 [*]:o:[*]	0.136

Top Features for negative contribution

Fingerprint	Bit/Smiles	Feature Structure	Score
ECFP_6	1996767644	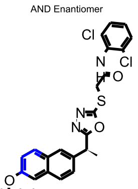 [*][c](:[*]):[cH]:[cH]:[*]	-0.251
ECFP_6	642810091	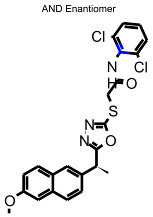 [*][c](:[*]):[*]	-0.247
ECFP_6	-182236392	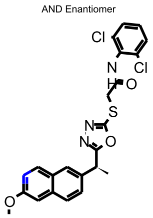 [*]:[cH]:[*]	-0.232

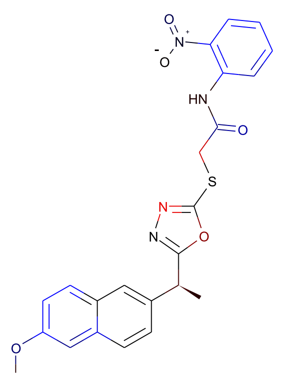

$C_{23}H_{20}N_4O_5S$
Molecular Weight: 464.4937
ALogP: 4.132
Rotatable Bonds: 8
Acceptors: 7
Donors: 1

Model Prediction

Prediction: 22.826
Unit: mg/kg_body_weight/day
Mahalanobis Distance: 14.734
Mahalanobis Distance p-value: 2.73e-012

Mahalanobis Distance: The Mahalanobis distance (MD) is a generalization of the Euclidean distance that accounts for correlations among the X properties. It is calculated as the distance to the center of the training data. The larger the MD, the less trustworthy the prediction.
Mahalanobis Distance p-value: The p-value gives the fraction of training data with an MD greater than or equal to the one for the given sample, assuming normally distributed data. The smaller the p-value, the less trustworthy the prediciton. For highly non-normal X properties (e.g., fingerprints), the MD p-value is wildly inaccurate.

Structural Similar Compounds

Name	470	646	Azathioprine s
Structure	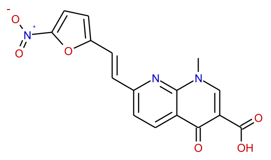	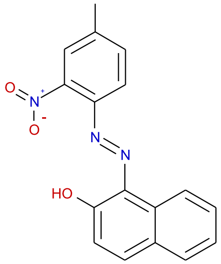	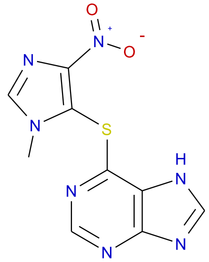
Actual Endpoint (-log C)	4.62839	0.937339	4.49253
Predicted Endpoint (-log C)	3.93264	3.26294	4.28607
Distance	0.881	0.890	0.893
Reference	CPDB	CPDB	CPDB

Model Applicability

Unknown features are fingerprint features in the query molecule, but not found in the training set.

1. All properties and OPS components are within expected ranges.
2. Unknown ECFP_2 feature: 1093109320: [*]S[c]1:o:[*]:[*]:n:1
3. Unknown ECFP_2 feature: -1841325949: [*]:[c](:[*])C(C)[c](:[*]):[*]
4. Unknown ECFP_2 feature: 1092541557: [*]C([*])[c]1:o:[*]:[*]:n:1

Feature Contribution

Top features for positive contribution			
Fingerprint	Bit/Smiles	Feature Structure	Score
ECFP_6	655739385	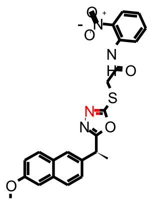 AND Enantiomer [*]:n:[*]	0.229

ECFP_6	1559650422	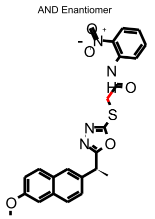 [*]C[*]	0.203
ECFP_6	683445015	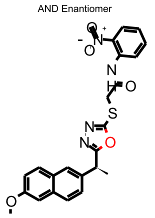 [*]:o:[*]	0.136

Top Features for negative contribution

Fingerprint	Bit/Smiles	Feature Structure	Score
ECFP_6	1996767644	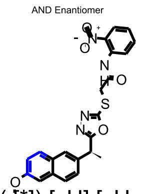 [*][c](:[*]):[cH]:[cH]:[*]	-0.251
ECFP_6	642810091	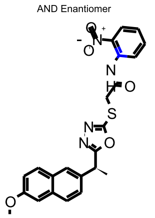 [*][c](:[*]):[*]	-0.247
ECFP_6	-182236392	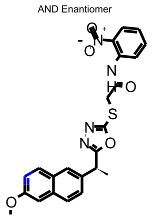 [*]:[cH]:[*]	-0.232

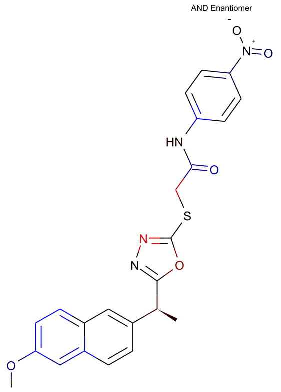

$C_{23}H_{20}N_4O_5S$
Molecular Weight: 464.4937
ALogP: 4.132
Rotatable Bonds: 8
Acceptors: 7
Donors: 1

Model Prediction

Prediction: 9.752
Unit: mg/kg_body_weight/day
Mahalanobis Distance: 14.331
Mahalanobis Distance p-value: 3.94e-011

Mahalanobis Distance: The Mahalanobis distance (MD) is a generalization of the Euclidean distance that accounts for correlations among the X properties. It is calculated as the distance to the center of the training data. The larger the MD, the less trustworthy the prediction.
Mahalanobis Distance p-value: The p-value gives the fraction of training data with an MD greater than or equal to the one for the given sample, assuming normally distributed data. The smaller the p-value, the less trustworthy the prediciton. For highly non-normal X properties (e.g., fingerprints), the MD p-value is wildly inaccurate.

Structural Similar Compounds

Name	470	Azathioprine s	223
Structure	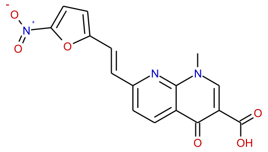	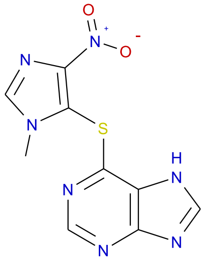	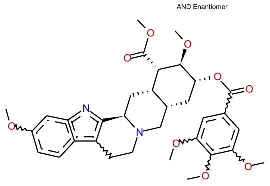
Actual Endpoint (-log C)	4.62839	4.49253	5.08368
Predicted Endpoint (-log C)	3.93264	4.28607	5.08273
Distance	0.879	0.892	0.897
Reference	CPDB	CPDB	CPDB

Model Applicability

Unknown features are fingerprint features in the query molecule, but not found in the training set.

1. All properties and OPS components are within expected ranges.
2. Unknown ECFP_2 feature: 1093109320: [*]S[c]1:o:[*]:[*]:n:1
3. Unknown ECFP_2 feature: -1841325949: [*]:[c](:[*])C(C)[c](:[*]):[*]
4. Unknown ECFP_2 feature: 1092541557: [*]C([*])[c]1:o:[*]:[*]:n:1

Feature Contribution

Top features for positive contribution

Fingerprint	Bit/Smiles	Feature Structure	Score
ECFP_6	655739385	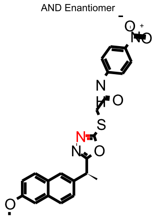 [*]:n:[*]	0.229

ECFP_6	1559650422	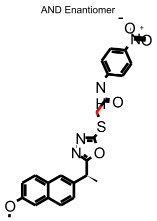 [*]C[*]	0.203
ECFP_6	683445015	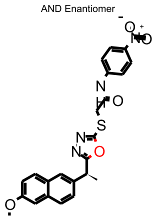 [*]:o:[*]	0.136

Top Features for negative contribution

Fingerprint	Bit/Smiles	Feature Structure	Score
ECFP_6	1996767644	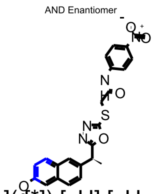 [*][c](:[*]):[cH]:[cH]:[*]	-0.251
ECFP_6	642810091	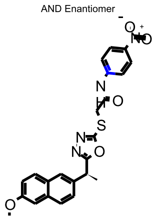 [*][c](:[*]):[*]	-0.247
ECFP_6	-182236392	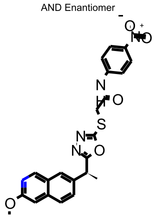 [*]:[cH]:[*]	-0.232

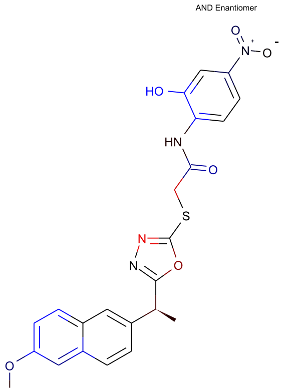

$C_{23}H_{20}N_4O_6S$
Molecular Weight: 480.4931
ALogP: 3.89
Rotatable Bonds: 8
Acceptors: 8
Donors: 2

Model Prediction

Prediction: 21.184
Unit: mg/kg_body_weight/day
Mahalanobis Distance: 14.635
Mahalanobis Distance p-value: 5.3e-012

Mahalanobis Distance: The Mahalanobis distance (MD) is a generalization of the Euclidean distance that accounts for correlations among the X properties. It is calculated as the distance to the center of the training data. The larger the MD, the less trustworthy the prediction.
Mahalanobis Distance p-value: The p-value gives the fraction of training data with an MD greater than or equal to the one for the given sample, assuming normally distributed data. The smaller the p-value, the less trustworthy the prediciton. For highly non-normal X properties (e.g., fingerprints), the MD p-value is wildly inaccurate.

Structural Similar Compounds

Name	420	Salicylazosulfapyridine	470
Structure	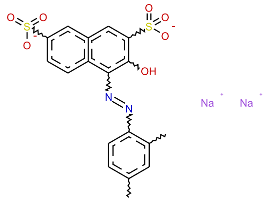	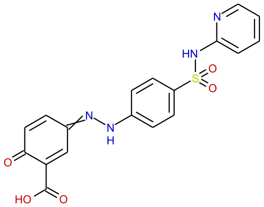	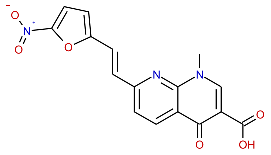
Actual Endpoint (-log C)	2.78302	2.5034	4.62839
Predicted Endpoint (-log C)	3.31546	3.54214	3.93264
Distance	0.880	0.882	0.923
Reference	CPDB	CPDB	CPDB

Model Applicability

Unknown features are fingerprint features in the query molecule, but not found in the training set.

1. All properties and OPS components are within expected ranges.
2. Unknown ECFP_2 feature: 1093109320: [*]S[c]1:o:[*]:[*]:n:1
3. Unknown ECFP_2 feature: -1841325949: [*]:[c](:[*])C(C)[c](:[*]):[*]
4. Unknown ECFP_2 feature: 1092541557: [*]C([*])[c]1:o:[*]:[*]:n:1

Feature Contribution

Top features for positive contribution			
Fingerprint	Bit/Smiles	Feature Structure	Score
ECFP_6	655739385	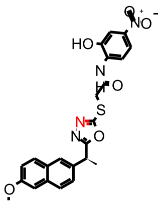 AND Enantiomer [*]:n:[*]	0.229

ECFP_6	1559650422	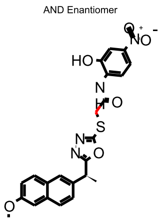 [*]C[*]	0.203
ECFP_6	683445015	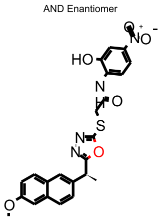 [*]:o:[*]	0.136

Top Features for negative contribution

Fingerprint	Bit/Smiles	Feature Structure	Score
ECFP_6	2019062761	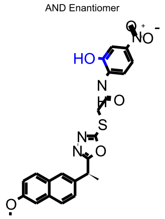 [*]:[c](:[*])O	-0.258
ECFP_6	1996767644	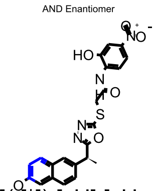 [*][c](:[*]):[cH]:[cH]:[*]	-0.251
ECFP_6	642810091	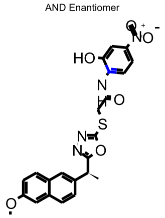 [*][c](:[*]):[*]	-0.247

Sorafenib

TOPKAT_Carcinogenic_Potency_TD50_Mouse

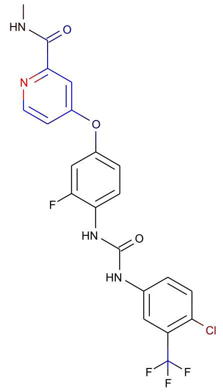

$C_{21}H_{15}ClF_{4}N_{4}O_{3}$
Molecular Weight: 482.81541
ALogP: 4.381
Rotatable Bonds: 6
Acceptors: 4
Donors: 3

Structural Similar Compounds

Name	Ochratoxin A	542	4-Chloro-6-(2,3-xylidino)-2-pyri-mi-dinylthio(N-b-hydroxy-ethyl) acetamide
Structure	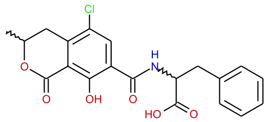	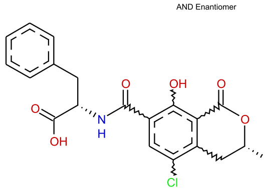	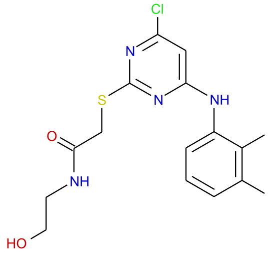
Actual Endpoint (-log C)	4.79932	4.79932	3.91517
Predicted Endpoint (-log C)	3.6353	3.6353	3.92186
Distance	0.731	0.731	0.748
Reference	CPDB	CPDB	CPDB

Model Prediction

Prediction: 17.535
Unit: mg/kg_body_weight/day
Mahalanobis Distance: 12.448
Mahalanobis Distance p-value: 2.31e-006

Mahalanobis Distance: The Mahalanobis distance (MD) is a generalization of the Euclidean distance that accounts for correlations among the X properties. It is calculated as the distance to the center of the training data. The larger the MD, the less trustworthy the prediction.
Mahalanobis Distance p-value: The p-value gives the fraction of training data with an MD greater than or equal to the one for the given sample, assuming normally distributed data. The smaller the p-value, the less trustworthy the prediciton. For highly non-normal X properties (e.g., fingerprints), the MD p-value is wildly inaccurate.

Model Applicability

Unknown features are fingerprint features in the query molecule, but not found in the training set.

1. All properties and OPS components are within expected ranges.
2. Unknown ECFP_2 feature: 1413420509: [*]C(=[*])[c](:n:[*]):c:[*]
3. Unknown ECFP_2 feature: 1338334141: [*]C(=[*])NC
4. Unknown ECFP_2 feature: -1311285389: [*][c](:[*]):[c](F):c:[*]

Feature Contribution

Top features for positive contribution

Fingerprint	Bit/Smiles	Feature Structure	Score
ECFP_6	655739385	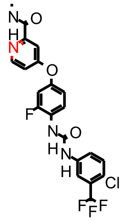 [*]:n:[*]	0.229

ECFP_6	-817402818	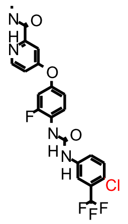 [*]Cl	0.129
ECFP_6	-176455838	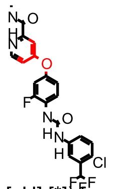 [*]O[c](:[cH]:[*]):[cH]:[*]	0.082

Top Features for negative contribution

Fingerprint	Bit/Smiles	Feature Structure	Score
ECFP_6	1996767644	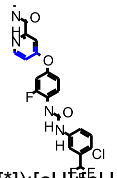 [*][c](:[*]):[cH]:[cH]:[*]	-0.251
ECFP_6	642810091	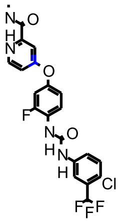 [*][c](:[*]):[*]	-0.247
ECFP_6	-182236392	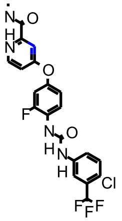 [*]:[cH]:[*]	-0.232

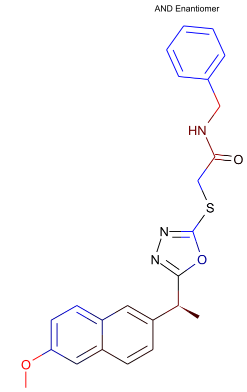

$C_{24}H_{23}N_3O_3S$
Molecular Weight: 433.52272
ALogP: 4.245
Rotatable Bonds: 8
Acceptors: 5
Donors: 1

Model Prediction

Prediction: 8.014
Unit: mg/kg_body_weight/day
Mahalanobis Distance: 15.196
Mahalanobis Distance p-value: 9.02e-012

Mahalanobis Distance: The Mahalanobis distance (MD) is a generalization of the Euclidean distance that accounts for correlations among the X properties. It is calculated as the distance to the center of the training data. The larger the MD, the less trustworthy the prediction.
Mahalanobis Distance p-value: The p-value gives the fraction of training data with an MD greater than or equal to the one for the given sample, assuming normally distributed data. The smaller the p-value, the less trustworthy the prediciton. For highly non-normal X properties (e.g., fingerprints), the MD p-value is wildly inaccurate.

Structural Similar Compounds

Name	Omeprazole	Indomethacin	C.I. direct brown 95
Structure	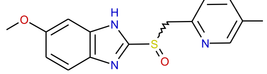	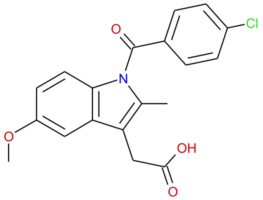	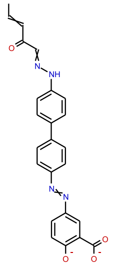
Actual Endpoint (-log C)	3.4628	5.49293	5.31387
Predicted Endpoint (-log C)	4.7324	4.9569	4.30266
Distance	0.701	0.707	0.722
Reference	CPDB	CPDB	CPDB

Model Applicability

Unknown features are fingerprint features in the query molecule, but not found in the training set.

1. All properties and OPS components are within expected ranges.

Feature Contribution

Top features for positive contribution

Fingerprint	Bit/Smiles	Feature Structure	Score
FCFP_6	136627117	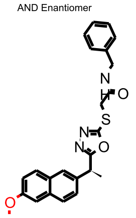 [*]OC	0.690

FCFP_6	1	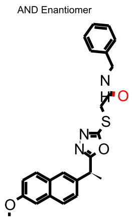<br>[*]=O	0.234
FCFP_6	-885550502	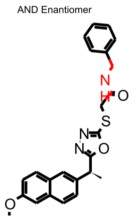<br>[*]CNC(=[*])[*]	0.229

Top Features for negative contribution

Fingerprint	Bit/Smiles	Feature Structure	Score
FCFP_6	991735244	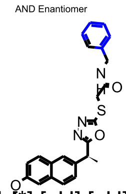<br>[*][c]1:[*]:[cH]:[cH]:[cH]:[cH]:1	-0.422
FCFP_6	-2093839777	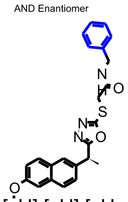<br>[*][c]1:[cH]:[cH]:[cH]:[cH]:[cH]:1	-0.378
FCFP_6	16	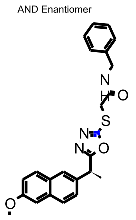<br>[*][c](:[*]):[*]	-0.354

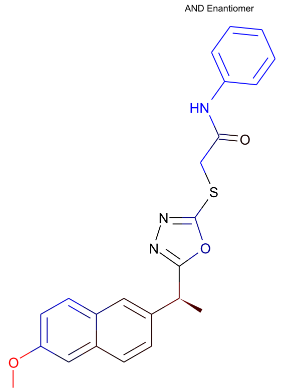

$C_{23}H_{21}N_3O_3S$
Molecular Weight: 419.49614
ALogP: 4.238
Rotatable Bonds: 7
Acceptors: 5
Donors: 1

Model Prediction

Prediction: 21.830
Unit: mg/kg_body_weight/day
Mahalanobis Distance: 15.310
Mahalanobis Distance p-value: 4.08e-012

Mahalanobis Distance: The Mahalanobis distance (MD) is a generalization of the Euclidean distance that accounts for correlations among the X properties. It is calculated as the distance to the center of the training data. The larger the MD, the less trustworthy the prediction.
Mahalanobis Distance p-value: The p-value gives the fraction of training data with an MD greater than or equal to the one for the given sample, assuming normally distributed data. The smaller the p-value, the less trustworthy the prediciton. For highly non-normal X properties (e.g., fingerprints), the MD p-value is wildly inaccurate.

Structural Similar Compounds

Name	Omeprazole	Indomethacin	C.I. direct brown 95
Structure	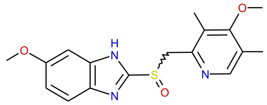	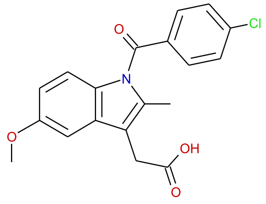	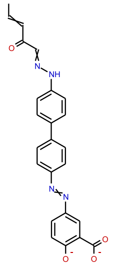
Actual Endpoint (-log C)	3.4628	5.49293	5.31387
Predicted Endpoint (-log C)	4.7324	4.9569	4.30266
Distance	0.672	0.674	0.717
Reference	CPDB	CPDB	CPDB

Model Applicability

Unknown features are fingerprint features in the query molecule, but not found in the training set.

1. All properties and OPS components are within expected ranges.

Feature Contribution

Top features for positive contribution			
**Fingerprint**	**Bit/Smiles**	**Feature Structure**	**Score**
FCFP_6	136627117	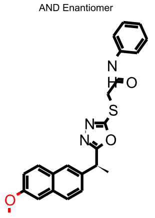 [*]OC	0.690

FCFP_6	1	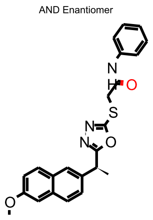 [*]=O	0.234
FCFP_6	203677720	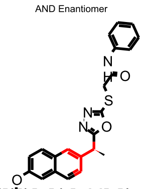 [*]C([*])[c](:[cH]:[*]):[cH]:[*]	0.137

Top Features for negative contribution

Fingerprint	Bit/Smiles	Feature Structure	Score
FCFP_6	991735244	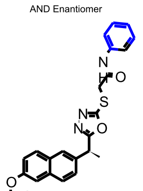 [*][c]1:[*]:[cH]:[cH] :[cH]:[cH]:1	-0.422
FCFP_6	-2093839777	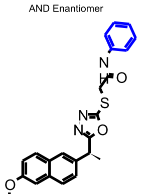 [*][c]1:[cH]:[cH]:[cH]:[cH]:[cH]:1	-0.378
FCFP_6	16	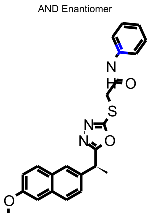 [*][c](:[*]):[*]	-0.354

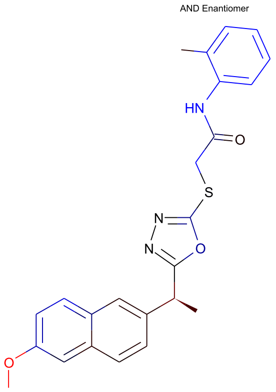

$C_{24}H_{23}N_3O_3S$
Molecular Weight: 433.52272
ALogP: 4.724
Rotatable Bonds: 7
Acceptors: 5
Donors: 1

Model Prediction

Prediction: 7.765
Unit: mg/kg_body_weight/day
Mahalanobis Distance: 15.707
Mahalanobis Distance p-value: 2.4e-013

Mahalanobis Distance: The Mahalanobis distance (MD) is a generalization of the Euclidean distance that accounts for correlations among the X properties. It is calculated as the distance to the center of the training data. The larger the MD, the less trustworthy the prediction.
Mahalanobis Distance p-value: The p-value gives the fraction of training data with an MD greater than or equal to the one for the given sample, assuming normally distributed data. The smaller the p-value, the less trustworthy the prediciton. For highly non-normal X properties (e.g., fingerprints), the MD p-value is wildly inaccurate.

Structural Similar Compounds

Name	Indomethacin	Omeprazole	C.I. direct brown 95
Structure	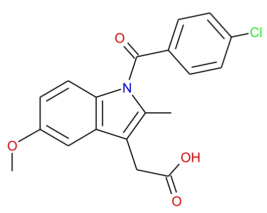	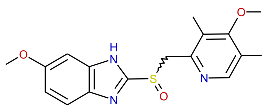	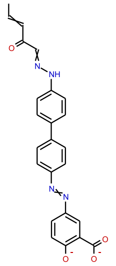
Actual Endpoint (-log C)	5.49293	3.4628	5.31387
Predicted Endpoint (-log C)	4.9569	4.7324	4.30266
Distance	0.682	0.693	0.723
Reference	CPDB	CPDB	CPDB

Model Applicability

Unknown features are fingerprint features in the query molecule, but not found in the training set.

1. All properties and OPS components are within expected ranges.

Feature Contribution

Top features for positive contribution			
**Fingerprint**	**Bit/Smiles**	**Feature Structure**	**Score**
FCFP_6	136627117	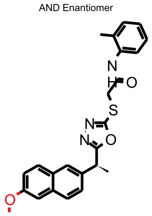 [*]OC	0.690

FCFP_6	1	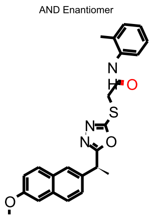 [*]=O	0.234
FCFP_6	203677720	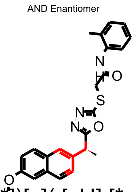 [*]C([*])[c](:[cH]:[*]):[cH]:[*]	0.137

Top Features for negative contribution

Fingerprint	Bit/Smiles	Feature Structure	Score
FCFP_6	991735244	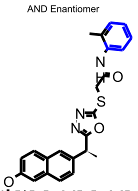 [*][c]1:[*]:[cH]:[cH]:[cH]:[cH]:1	-0.422
FCFP_6	16	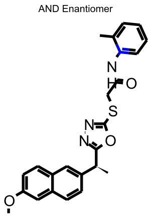 [*][c](:[*]):[*]	-0.354
FCFP_6	590925877	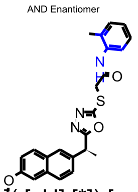 [*]N[c](:[cH]:[*]):[cH]:[*]	-0.323

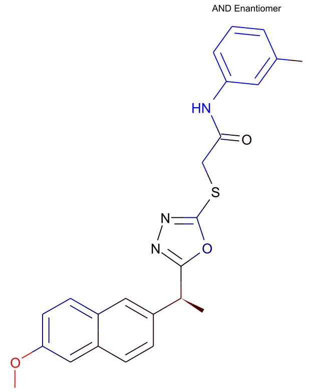

$C_{24}H_{23}N_3O_3S$
Molecular Weight: 433.52272
ALogP: 4.724
Rotatable Bonds: 7
Acceptors: 5
Donors: 1

Model Prediction

Prediction: 7.471
Unit: mg/kg_body_weight/day
Mahalanobis Distance: 14.987
Mahalanobis Distance p-value: 3.77e-011

Mahalanobis Distance: The Mahalanobis distance (MD) is a generalization of the Euclidean distance that accounts for correlations among the X properties. It is calculated as the distance to the center of the training data. The larger the MD, the less trustworthy the prediction.
Mahalanobis Distance p-value: The p-value gives the fraction of training data with an MD greater than or equal to the one for the given sample, assuming normally distributed data. The smaller the p-value, the less trustworthy the prediciton. For highly non-normal X properties (e.g., fingerprints), the MD p-value is wildly inaccurate.

Structural Similar Compounds

Name	Indomethacin	Omeprazole	C.I. direct brown 95
Structure	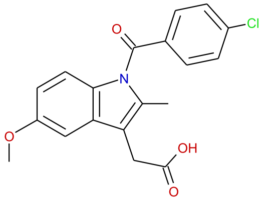	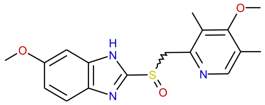	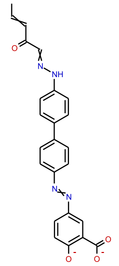
Actual Endpoint (-log C)	5.49293	3.4628	5.31387
Predicted Endpoint (-log C)	4.9569	4.7324	4.30266
Distance	0.682	0.693	0.723
Reference	CPDB	CPDB	CPDB

Model Applicability

Unknown features are fingerprint features in the query molecule, but not found in the training set.

1. All properties and OPS components are within expected ranges.

Feature Contribution

Top features for positive contribution

Fingerprint	Bit/Smiles	Feature Structure	Score
FCFP_6	136627117	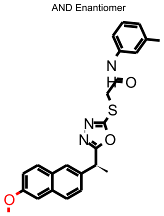 [*]OC	0.690

FCFP_6	1	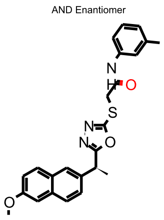<br>[*]=O	0.234
FCFP_6	203677720	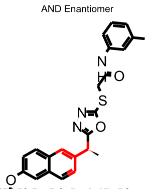<br>[*]C([*])[c](:[cH]:[*]):[cH]:[*]	0.137

Top Features for negative contribution

Fingerprint	Bit/Smiles	Feature Structure	Score
FCFP_6	991735244	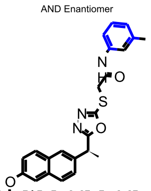<br>[*][c]1:[*]:[cH]:[cH]:[cH]:[cH]:1	-0.422
FCFP_6	16	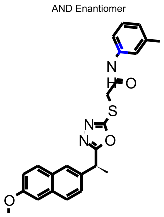<br>[*][c](:[*]):[*]	-0.354
FCFP_6	590925877	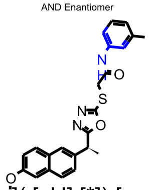<br>[*]N[c](:[cH]:[*]):[cH]:[*]	-0.323

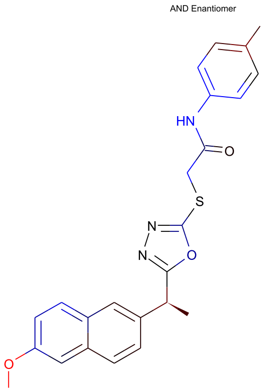

$C_{24}H_{23}N_{3}O_{3}S$
Molecular Weight: 433.52272
ALogP: 4.724
Rotatable Bonds: 7
Acceptors: 5
Donors: 1

Model Prediction

Prediction: 2.830
Unit: mg/kg_body_weight/day
Mahalanobis Distance: 15.223
Mahalanobis Distance p-value: 7.48e-012

Mahalanobis Distance: The Mahalanobis distance (MD) is a generalization of the Euclidean distance that accounts for correlations among the X properties. It is calculated as the distance to the center of the training data. The larger the MD, the less trustworthy the prediction.
Mahalanobis Distance p-value: The p-value gives the fraction of training data with an MD greater than or equal to the one for the given sample, assuming normally distributed data. The smaller the p-value, the less trustworthy the prediciton. For highly non-normal X properties (e.g., fingerprints), the MD p-value is wildly inaccurate.

Structural Similar Compounds

Name	Indomethacin	Omeprazole	C.I. direct brown 95
Structure	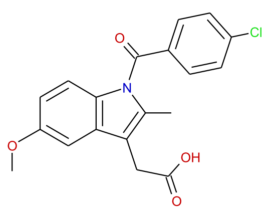	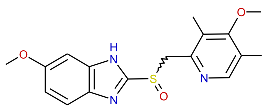	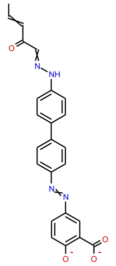
Actual Endpoint (-log C)	5.49293	3.4628	5.31387
Predicted Endpoint (-log C)	4.9569	4.7324	4.30266
Distance	0.680	0.692	0.722
Reference	CPDB	CPDB	CPDB

Model Applicability

Unknown features are fingerprint features in the query molecule, but not found in the training set.

1. All properties and OPS components are within expected ranges.

Feature Contribution

Top features for positive contribution

Fingerprint	Bit/Smiles	Feature Structure	Score
FCFP_6	136627117	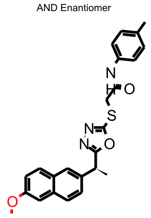 [*]OC	0.690

FCFP_6	1	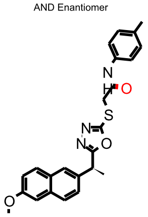 [*]=O	0.234
FCFP_6	203677720	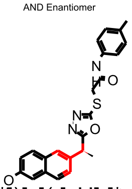 [*]C([*])[c](:[cH]:[*]):[cH]:[*]	0.137
**Top Features for negative contribution**			
**Fingerprint**	**Bit/Smiles**	**Feature Structure**	**Score**
FCFP_6	16	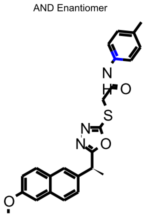 [*][c](:[*]):[*]	-0.354
FCFP_6	590925877	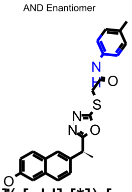 [*]N[c](:[cH]:[*]):[cH]:[*]	-0.323
FCFP_6	1674451008	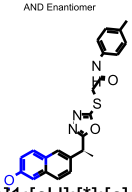 [*]O[c]1:[cH]:[*]:[c](:[*]):[cH]:[cH]:1	-0.233

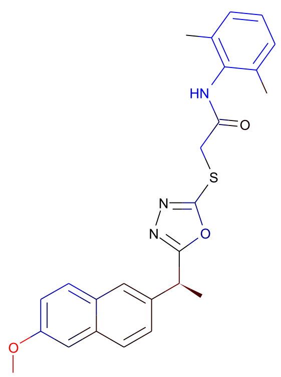

$C_{25}H_{25}N_{3}O_{3}S$
Molecular Weight: 447.5493
ALogP: 5.21
Rotatable Bonds: 7
Acceptors: 5
Donors: 1

Model Prediction

Prediction: 7.949
Unit: mg/kg_body_weight/day
Mahalanobis Distance: 16.512
Mahalanobis Distance p-value: 5.61e-016

Mahalanobis Distance: The Mahalanobis distance (MD) is a generalization of the Euclidean distance that accounts for correlations among the X properties. It is calculated as the distance to the center of the training data. The larger the MD, the less trustworthy the prediction.
Mahalanobis Distance p-value: The p-value gives the fraction of training data with an MD greater than or equal to the one for the given sample, assuming normally distributed data. The smaller the p-value, the less trustworthy the prediciton. For highly non-normal X properties (e.g., fingerprints), the MD p-value is wildly inaccurate.

Structural Similar Compounds

Name	Indomethacin	Omeprazole	FD & C violet no. 1
Structure	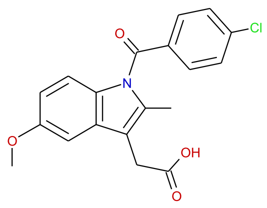	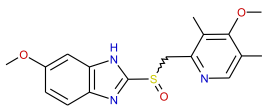	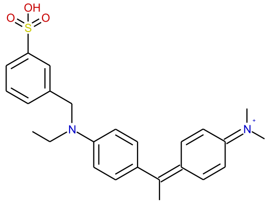
Actual Endpoint (-log C)	5.49293	3.4628	2.8543
Predicted Endpoint (-log C)	4.9569	4.7324	3.40838
Distance	0.696	0.719	0.735
Reference	CPDB	CPDB	CPDB

Model Applicability

Unknown features are fingerprint features in the query molecule, but not found in the training set.

1. All properties and OPS components are within expected ranges.

Feature Contribution

Top features for positive contribution

Fingerprint	Bit/Smiles	Feature Structure	Score
FCFP_6	136627117	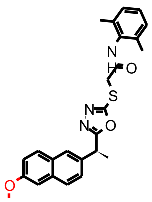 AND Enantiomer [*]OC	0.690

FCFP_6	1	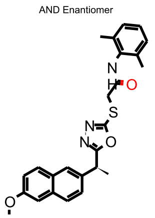 [*]=O	0.234
FCFP_6	203677720	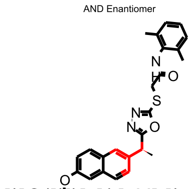 [*]C([*])[c](:[cH]:[*]):[cH]:[*]	0.137

Top Features for negative contribution

**Fingerprint**	**Bit/Smiles**	**Feature Structure**	**Score**
FCFP_6	991735244	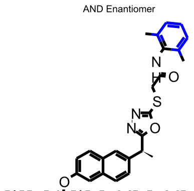 [*][c]1:[*]:[cH]:[cH]:[cH]:[cH]:1	-0.422
FCFP_6	16	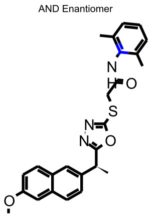 [*][c](:[*]):[*]	-0.354
FCFP_6	590925877	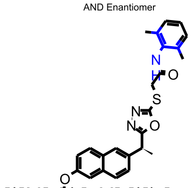 [*]N[c](:[cH]:[*]):[cH]:[*]	-0.323

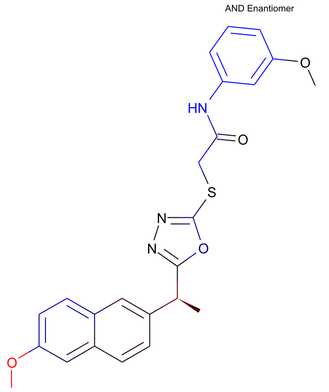

$C_{24}H_{23}N_3O_4S$
Molecular Weight: 449.52212
ALogP: 4.221
Rotatable Bonds: 8
Acceptors: 6
Donors: 1

Model Prediction

Prediction: 8.860
Unit: mg/kg_body_weight/day
Mahalanobis Distance: 14.508
Mahalanobis Distance p-value: 8.87e-010

Mahalanobis Distance: The Mahalanobis distance (MD) is a generalization of the Euclidean distance that accounts for correlations among the X properties. It is calculated as the distance to the center of the training data. The larger the MD, the less trustworthy the prediction.
Mahalanobis Distance p-value: The p-value gives the fraction of training data with an MD greater than or equal to the one for the given sample, assuming normally distributed data. The smaller the p-value, the less trustworthy the prediciton. For highly non-normal X properties (e.g., fingerprints), the MD p-value is wildly inaccurate.

Structural Similar Compounds

Name	C.I. direct brown 95	Omeprazole	Indomethacin
Structure	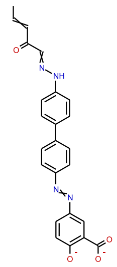	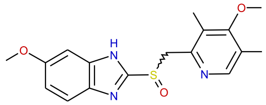	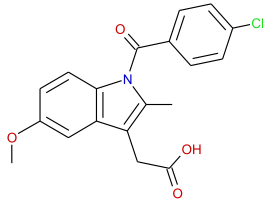
Actual Endpoint (-log C)	5.31387	3.4628	5.49293
Predicted Endpoint (-log C)	4.30266	4.7324	4.9569
Distance	0.666	0.725	0.749
Reference	CPDB	CPDB	CPDB

Model Applicability

Unknown features are fingerprint features in the query molecule, but not found in the training set.

1. All properties and OPS components are within expected ranges.

Feature Contribution

Top features for positive contribution			
**Fingerprint**	**Bit/Smiles**	**Feature Structure**	**Score**
FCFP_6	136627117	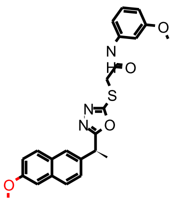 AND Enantiomer [*]OC	0.690

FCFP_6	1	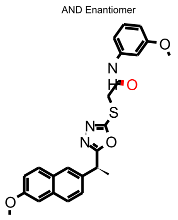 [*]=O	0.234
FCFP_6	203677720	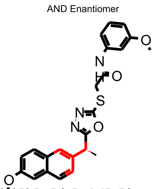 [*]C([*])[c](:[cH]:[*]):[cH]:[*]	0.137
**Top Features for negative contribution**			
**Fingerprint**	**Bit/Smiles**	**Feature Structure**	**Score**
FCFP_6	991735244	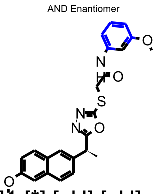 [*][c]1:[*]:[cH]:[cH]:[cH]:[cH]:1	-0.422
FCFP_6	16	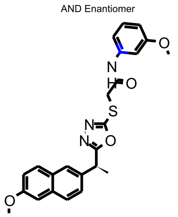 [*][c](:[*]):[*]	-0.354
FCFP_6	590925877	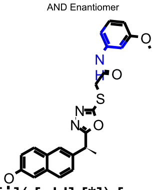 [*]N[c](:[cH]:[*]):[cH]:[*]	-0.323

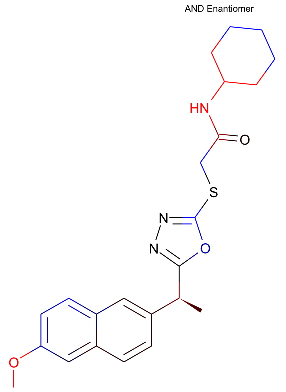

$C_{23}H_{27}N_3O_3S$
Molecular Weight: 425.54378
ALogP: 4.52
Rotatable Bonds: 7
Acceptors: 5
Donors: 1

Model Prediction

Prediction: 0.210
Unit: mg/kg_body_weight/day
Mahalanobis Distance: 16.688
Mahalanobis Distance p-value: 1.42e-016

Mahalanobis Distance: The Mahalanobis distance (MD) is a generalization of the Euclidean distance that accounts for correlations among the X properties. It is calculated as the distance to the center of the training data. The larger the MD, the less trustworthy the prediction.
Mahalanobis Distance p-value: The p-value gives the fraction of training data with an MD greater than or equal to the one for the given sample, assuming normally distributed data. The smaller the p-value, the less trustworthy the prediciton. For highly non-normal X properties (e.g., fingerprints), the MD p-value is wildly inaccurate.

Structural Similar Compounds

Name	Indomethacin	Omeprazole	FD & C violet no. 1
Structure	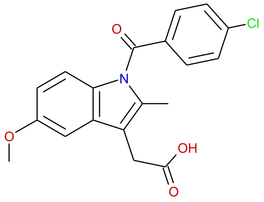	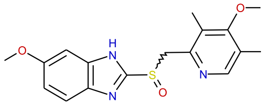	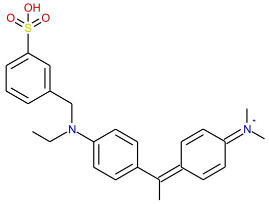
Actual Endpoint (-log C)	5.49293	3.4628	2.8543
Predicted Endpoint (-log C)	4.9569	4.7324	3.40838
Distance	0.643	0.646	0.650
Reference	CPDB	CPDB	CPDB

Model Applicability

Unknown features are fingerprint features in the query molecule, but not found in the training set.

1. All properties and OPS components are within expected ranges.

Feature Contribution

Top features for positive contribution			
**Fingerprint**	**Bit/Smiles**	**Feature Structure**	**Score**
FCFP_6	-1043250487	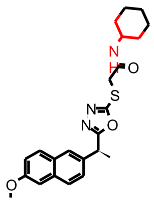 AND Enantiomer [*]CC(C[*])N[*]	1.153

FCFP_6	136627117	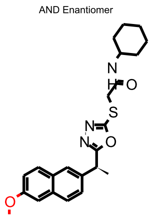 [*]OC	0.690
FCFP_6	1	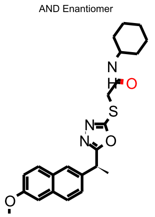 [*]=O	0.234
**Top Features for negative contribution**			
**Fingerprint**	**Bit/Smiles**	**Feature Structure**	**Score**
FCFP_6	1175638033	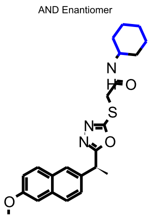 [*]C1[*]CCCC1	-0.512
FCFP_6	16	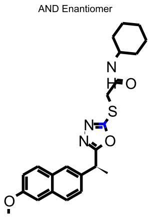 [*][c](:[*]):[*]	-0.354
FCFP_6	1674451008	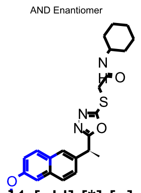 [*]O[c]1:[cH]:[*]:[c](:[*]):[cH]:[cH]:1	-0.233

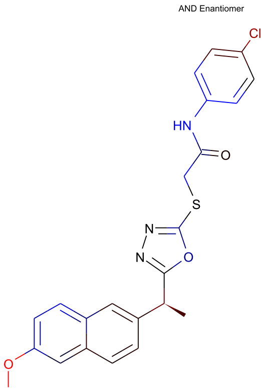

$C_{23}H_{20}ClN_3O_3S$
Molecular Weight: 453.9412
ALogP: 4.902
Rotatable Bonds: 7
Acceptors: 5
Donors: 1

Model Prediction

Prediction: 2.204
Unit: mg/kg_body_weight/day
Mahalanobis Distance: 15.321
Mahalanobis Distance p-value: 3.78e-012

Mahalanobis Distance: The Mahalanobis distance (MD) is a generalization of the Euclidean distance that accounts for correlations among the X properties. It is calculated as the distance to the center of the training data. The larger the MD, the less trustworthy the prediction.
Mahalanobis Distance p-value: The p-value gives the fraction of training data with an MD greater than or equal to the one for the given sample, assuming normally distributed data. The smaller the p-value, the less trustworthy the prediciton. For highly non-normal X properties (e.g., fingerprints), the MD p-value is wildly inaccurate.

Structural Similar Compounds

Name	Indomethacin	Omeprazole	C.I. direct brown 95
Structure	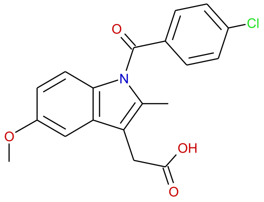	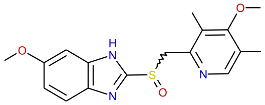	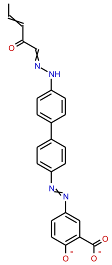
Actual Endpoint (-log C)	5.49293	3.4628	5.31387
Predicted Endpoint (-log C)	4.9569	4.7324	4.30266
Distance	0.663	0.711	0.727
Reference	CPDB	CPDB	CPDB

Model Applicability

Unknown features are fingerprint features in the query molecule, but not found in the training set.

1. OPS PC18 out of range. Value: 5.2005. Training min, max, SD, explained variance: -4.1023, 4.8669, 1.305, 0.0164.

Feature Contribution

Top features for positive contribution			
**Fingerprint**	**Bit/Smiles**	**Feature Structure**	**Score**
FCFP_6	136627117	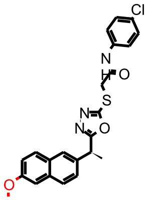 AND Enantiomer [*]OC	0.690

FCFP_6	1	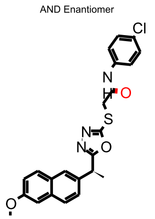 [*]=O	0.234
FCFP_6	32	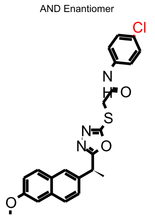 [*]Cl	0.154

Top Features for negative contribution

**Fingerprint**	**Bit/Smiles**	**Feature Structure**	**Score**
FCFP_6	16	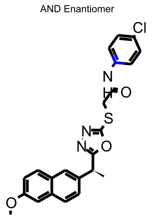 [*][c](:[*]):[*]	-0.354
FCFP_6	590925877	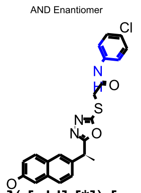 [*]N[c](:[cH]:[*]):[cH]:[*]	-0.323
FCFP_6	1674451008	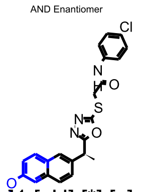 [*]O[c]1:[cH]:[*]:[c](:[*]):[cH]:[cH]:1	-0.233

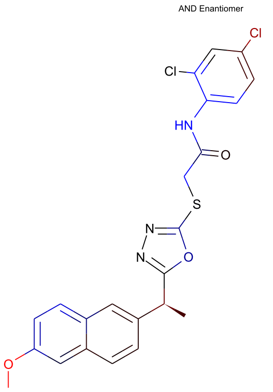

$C_{23}H_{19}Cl_2N_3O_3S$
Molecular Weight: 488.38626
ALogP: 5.567
Rotatable Bonds: 7
Acceptors: 5
Donors: 1

Model Prediction

Prediction: 1.999
Unit: mg/kg_body_weight/day
Mahalanobis Distance: 15.298
Mahalanobis Distance p-value: 4.45e-012

Mahalanobis Distance: The Mahalanobis distance (MD) is a generalization of the Euclidean distance that accounts for correlations among the X properties. It is calculated as the distance to the center of the training data. The larger the MD, the less trustworthy the prediction.
Mahalanobis Distance p-value: The p-value gives the fraction of training data with an MD greater than or equal to the one for the given sample, assuming normally distributed data. The smaller the p-value, the less trustworthy the prediciton. For highly non-normal X properties (e.g., fingerprints), the MD p-value is wildly inaccurate.

Structural Similar Compounds

Name	Indomethacin	C.I. direct brown 95	FD & C violet no. 1
Structure	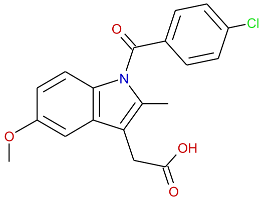	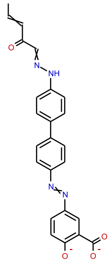	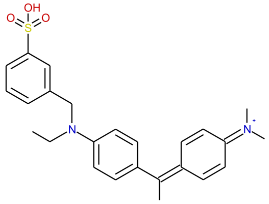
Actual Endpoint (-log C)	5.49293	5.31387	2.8543
Predicted Endpoint (-log C)	4.9569	4.30266	3.40838
Distance	0.701	0.748	0.754
Reference	CPDB	CPDB	CPDB

Model Applicability

Unknown features are fingerprint features in the query molecule, but not found in the training set.

1. OPS PC18 out of range. Value: 5.1982. Training min, max, SD, explained variance: -4.1023, 4.8669, 1.305, 0.0164.

Feature Contribution

Top features for positive contribution			
**Fingerprint**	**Bit/Smiles**	**Feature Structure**	**Score**
FCFP_6	136627117	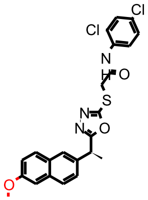 AND Enantiomer [*]OC	0.690

FCFP_6	1	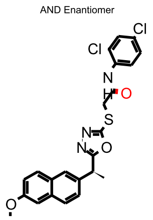 [*]=O	0.234
FCFP_6	32	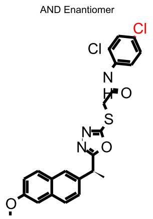 [*]Cl	0.154

Top Features for negative contribution

Fingerprint	Bit/Smiles	Feature Structure	Score
FCFP_6	16	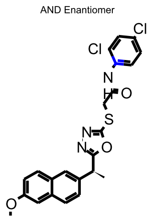 [*][c](:[*]):[*]	-0.354
FCFP_6	590925877	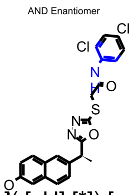 [*]N[c](:[cH]:[*]):[cH]:[*]	-0.323
FCFP_6	1674451008	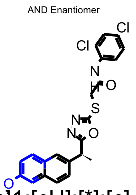 [*]O[c]1:[cH]:[*]:[c](:[*]):[cH]:[cH]:1	-0.233

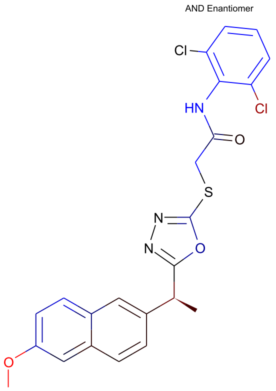

$C_{23}H_{19}Cl_2N_3O_3S$
Molecular Weight: 488.38626
ALogP: 5.567
Rotatable Bonds: 7
Acceptors: 5
Donors: 1

Model Prediction

Prediction: 6.077
Unit: mg/kg_body_weight/day
Mahalanobis Distance: 16.970
Mahalanobis Distance p-value: 1.5e-017

Mahalanobis Distance: The Mahalanobis distance (MD) is a generalization of the Euclidean distance that accounts for correlations among the X properties. It is calculated as the distance to the center of the training data. The larger the MD, the less trustworthy the prediction.
Mahalanobis Distance p-value: The p-value gives the fraction of training data with an MD greater than or equal to the one for the given sample, assuming normally distributed data. The smaller the p-value, the less trustworthy the prediciton. For highly non-normal X properties (e.g., fingerprints), the MD p-value is wildly inaccurate.

Structural Similar Compounds

Name	Indomethacin	FD & C violet no. 1	C.I. direct brown 95
Structure	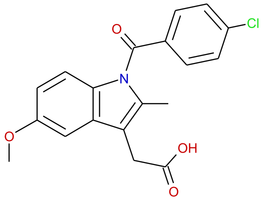	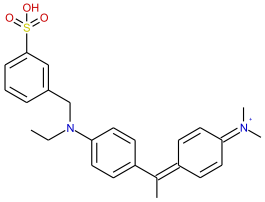	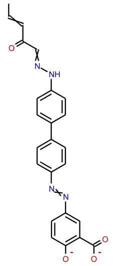
Actual Endpoint (-log C)	5.49293	2.8543	5.31387
Predicted Endpoint (-log C)	4.9569	3.40838	4.30266
Distance	0.703	0.748	0.752
Reference	CPDB	CPDB	CPDB

Model Applicability

Unknown features are fingerprint features in the query molecule, but not found in the training set.

1. OPS PC18 out of range. Value: 5.4834. Training min, max, SD, explained variance: -4.1023, 4.8669, 1.305, 0.0164.

Feature Contribution

Top features for positive contribution			
Fingerprint	Bit/Smiles	Feature Structure	Score
FCFP_6	136627117	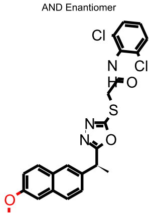 [*]OC	0.690

FCFP_6	1	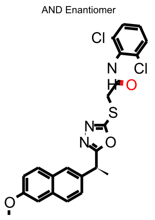 [*]=O	0.234
FCFP_6	32	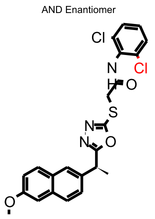 [*]Cl	0.154

Top Features for negative contribution

**Fingerprint**	**Bit/Smiles**	**Feature Structure**	**Score**
FCFP_6	991735244	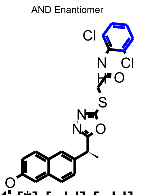 [*][c]1:[*]:[cH]:[cH]:[cH]:[cH]:1	-0.422
FCFP_6	16	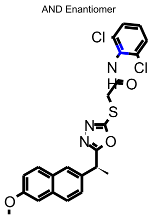 [*][c](:[*]):[*]	-0.354
FCFP_6	590925877	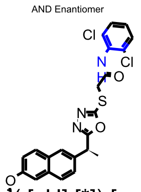 [*]N[c](:[cH]:[*]):[cH]:[*]	-0.323

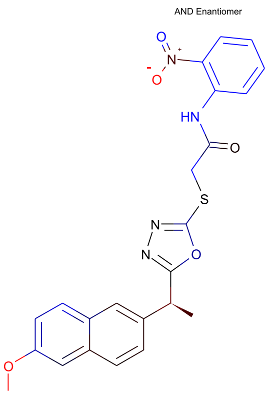

$C_{23}H_{20}N_4O_5S$

Molecular Weight: 464.4937

ALogP: 4.132

Rotatable Bonds: 8

Acceptors: 7

Donors: 1

Model Prediction

Prediction: 4.598

Unit: mg/kg_body_weight/day

Mahalanobis Distance: 15.752

Mahalanobis Distance p-value: 1.73e-013

Mahalanobis Distance: The Mahalanobis distance (MD) is a generalization of the Euclidean distance that accounts for correlations among the X properties. It is calculated as the distance to the center of the training data. The larger the MD, the less trustworthy the prediction.

Mahalanobis Distance p-value: The p-value gives the fraction of training data with an MD greater than or equal to the one for the given sample, assuming normally distributed data. The smaller the p-value, the less trustworthy the prediciton. For highly non-normal X properties (e.g., fingerprints), the MD p-value is wildly inaccurate.

Structural Similar Compounds

Name	C.I. direct brown 95	4-Bis(2-hydroxyethyl)amino-2-(5-nitro-2-thienyl)quinazoline	623
Structure	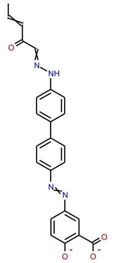	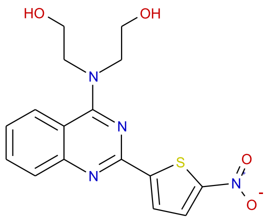	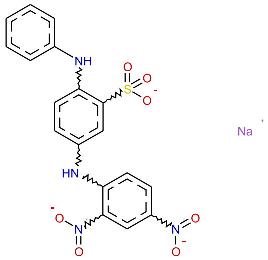
Actual Endpoint (-log C)	5.31387	5.05984	2.39985
Predicted Endpoint (-log C)	4.30266	4.23808	3.4177
Distance	0.642	0.718	0.746
Reference	CPDB	CPDB	CPDB

Model Applicability

Unknown features are fingerprint features in the query molecule, but not found in the training set.

1. OPS PC21 out of range. Value: -5.8098. Training min, max, SD, explained variance: -5.5407, 3.6894, 1.173, 0.0132.

Feature Contribution

Top features for positive contribution			
**Fingerprint**	**Bit/Smiles**	**Feature Structure**	**Score**
FCFP_6	136627117	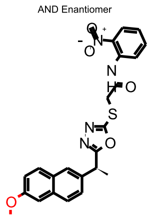 [*]OC	0.690

FCFP_6	5	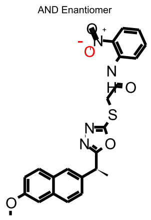 [*][O-]	0.431
FCFP_6	8	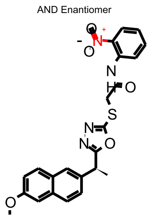 [*][N+](=[*])[*]	0.336
**Top Features for negative contribution**			
**Fingerprint**	**Bit/Smiles**	**Feature Structure**	**Score**
FCFP_6	991735244	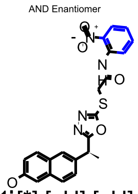 [*][c]1:[*]:[cH]:[cH]:[cH]:[cH]:1	-0.422
FCFP_6	16	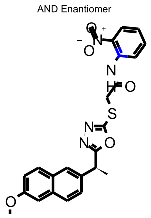 [*][c](:[*]):[*]	-0.354
FCFP_6	590925877	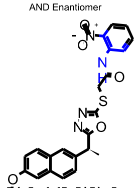 [*]N[c](:[cH]:[*]):[cH]:[*]	-0.323

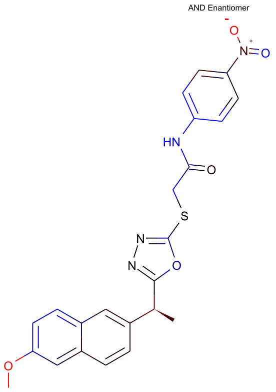

$C_{23}H_{20}N_4O_5S$
Molecular Weight: 464.4937
ALogP: 4.132
Rotatable Bonds: 8
Acceptors: 7
Donors: 1

Model Prediction

Prediction: 1.742
Unit: mg/kg_body_weight/day
Mahalanobis Distance: 15.877
Mahalanobis Distance p-value: 6.92e-014

Mahalanobis Distance: The Mahalanobis distance (MD) is a generalization of the Euclidean distance that accounts for correlations among the X properties. It is calculated as the distance to the center of the training data. The larger the MD, the less trustworthy the prediction.
Mahalanobis Distance p-value: The p-value gives the fraction of training data with an MD greater than or equal to the one for the given sample, assuming normally distributed data. The smaller the p-value, the less trustworthy the prediciton. For highly non-normal X properties (e.g., fingerprints), the MD p-value is wildly inaccurate.

Structural Similar Compounds

Name	C.I. direct brown 95	4-Bis(2-hydroxyethyl)amino-2-(5-nitro-2-thienyl)quinazoline	623
Structure	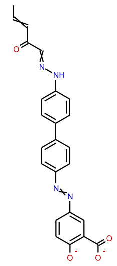	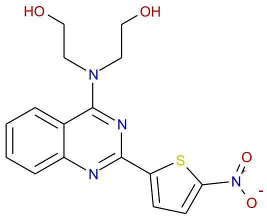	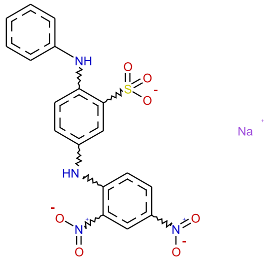
Actual Endpoint (-log C)	5.31387	5.05984	2.39985
Predicted Endpoint (-log C)	4.30266	4.23808	3.4177
Distance	0.641	0.721	0.749
Reference	CPDB	CPDB	CPDB

Model Applicability

Unknown features are fingerprint features in the query molecule, but not found in the training set.

1. All properties and OPS components are within expected ranges.

Feature Contribution

Top features for positive contribution			
Fingerprint	Bit/Smiles	Feature Structure	Score
FCFP_6	136627117	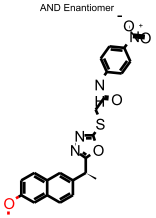 [*]OC	0.690

FCFP_6	5	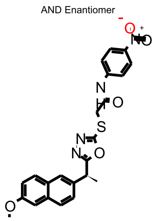 [*][O-]	0.431
FCFP_6	8	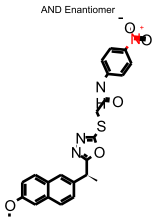 [*][N+](=[*])[*]	0.336

Top Features for negative contribution

Fingerprint	Bit/Smiles	Feature Structure	Score
FCFP_6	16	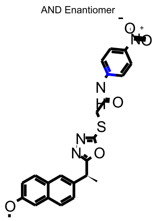 [*][c](:[*]):[*]	-0.354
FCFP_6	590925877	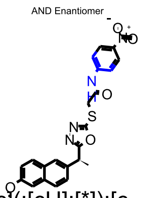 [*]N[c](:[cH]:[*]):[cH]:[*]	-0.323
FCFP_6	1674451008	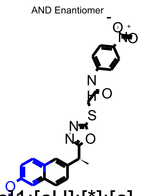 [*]O[c]1:[cH]:[*]:[c](:[*]):[cH]:[cH]:1	-0.233

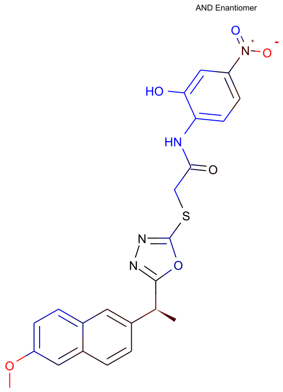

$C_{23}H_{20}N_4O_6S$

Molecular Weight: 480.4931

ALogP: 3.89

Rotatable Bonds: 8

Acceptors: 8

Donors: 2

Model Prediction

Prediction: 6.640

Unit: mg/kg_body_weight/day

Mahalanobis Distance: 18.009

Mahalanobis Distance p-value: 2.7e-021

Mahalanobis Distance: The Mahalanobis distance (MD) is a generalization of the Euclidean distance that accounts for correlations among the X properties. It is calculated as the distance to the center of the training data. The larger the MD, the less trustworthy the prediction.
Mahalanobis Distance p-value: The p-value gives the fraction of training data with an MD greater than or equal to the one for the given sample, assuming normally distributed data. The smaller the p-value, the less trustworthy the prediciton. For highly non-normal X properties (e.g., fingerprints), the MD p-value is wildly inaccurate.

Structural Similar Compounds

Name	623	C.I. direct brown 95	4-Bis(2-hydroxyethyl)amino-2-(5-nitro-2-thienyl)quinazoline
Structure	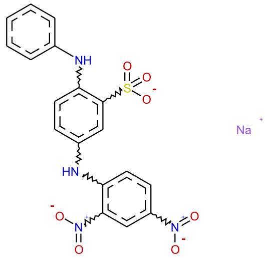	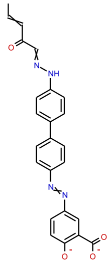	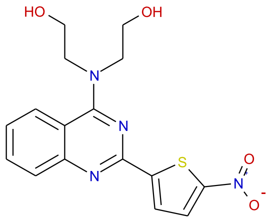
Actual Endpoint (-log C)	2.39985	5.31387	5.05984
Predicted Endpoint (-log C)	3.4177	4.30266	4.23808
Distance	0.671	0.721	0.731
Reference	CPDB	CPDB	CPDB

Model Applicability

Unknown features are fingerprint features in the query molecule, but not found in the training set.

1. OPS PC21 out of range. Value: -5.6721. Training min, max, SD, explained variance: -5.5407, 3.6894, 1.173, 0.0132.

Feature Contribution

Top features for positive contribution			
**Fingerprint**	**Bit/Smiles**	**Feature Structure**	**Score**
FCFP_6	136627117	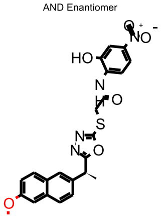 [*]OC	0.690

FCFP_6	5	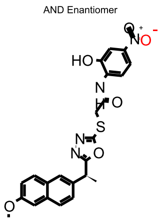 [*][O-]	0.431
FCFP_6	8	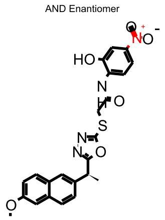 [*][N+](=[*])[*]	0.336

Top Features for negative contribution

Fingerprint	Bit/Smiles	Feature Structure	Score
FCFP_6	7	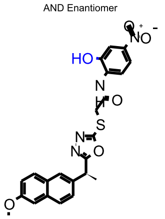 [*]O	-0.372
FCFP_6	16	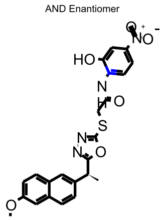 [*][c](:[*]):[*]	-0.354
FCFP_6	590925877	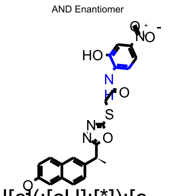 [*]N[c](:[cH]:[*]):[cH]:[*]	-0.323

Sorafenib

TOPKAT_Carcinogenic_Potency_TD50_Rat

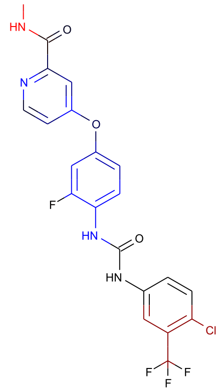

$C_{21}H_{15}ClF_4N_4O_3$

Molecular Weight: 482.81541

ALogP: 4.381

Rotatable Bonds: 6

Acceptors: 4

Donors: 3

Model Prediction

Prediction: 13.905

Unit: mg/kg_body_weight/day

Mahalanobis Distance: 20.390

Mahalanobis Distance p-value: 1.16e-030

Mahalanobis Distance: The Mahalanobis distance (MD) is a generalization of the Euclidean distance that accounts for correlations among the X properties. It is calculated as the distance to the center of the training data. The larger the MD, the less trustworthy the prediction.
Mahalanobis Distance p-value: The p-value gives the fraction of training data with an MD greater than or equal to the one for the given sample, assuming normally distributed data. The smaller the p-value, the less trustworthy the prediciton. For highly non-normal X properties (e.g., fingerprints), the MD p-value is wildly inaccurate.

Structural Similar Compounds

Name	Fluvastatin	913	Ochratoxin A
Structure	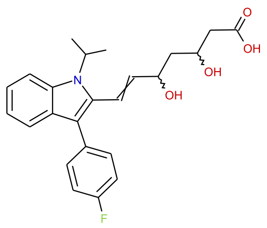	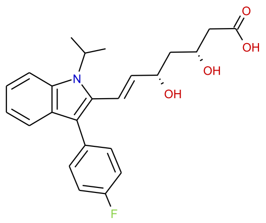	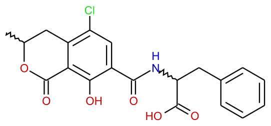
Actual Endpoint (-log C)	3.51742	3.51742	6.47264
Predicted Endpoint (-log C)	5.41573	5.41573	5.06501
Distance	0.611	0.611	0.682
Reference	CPDB	CPDB	CPDB

Model Applicability

Unknown features are fingerprint features in the query molecule, but not found in the training set.

1. All properties and OPS components are within expected ranges.
2. Unknown FCFP_2 feature: -1029533685: [*]:[c](:[*])C(F)(F)F

Feature Contribution

Top features for positive contribution

Fingerprint	Bit/Smiles	Feature Structure	Score
FCFP_6	1	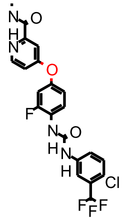 [*]=O	0.234

FCFP_6	-885550502	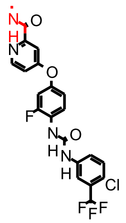 [*]CNC(=[*])[*]	0.229
FCFP_6	32	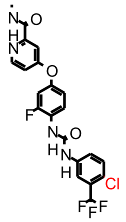 [*]Cl	0.154
**Top Features for negative contribution**			
**Fingerprint**	**Bit/Smiles**	**Feature Structure**	**Score**
FCFP_6	16	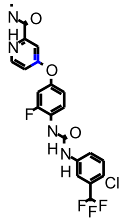 [*][c](:[*]):[*]	-0.354
FCFP_6	590925877	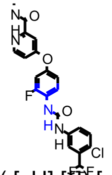 [*]N[c](:[cH]:[*]):[cH]:[*]	-0.323
FCFP_6	1674451008	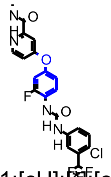 [*]O[c]1:[cH]:[*]:[c](:[*]):[cH]:[cH]:1	-0.233

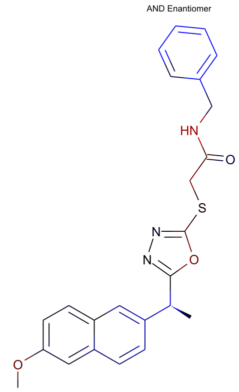

$C_{24}H_{23}N_3O_3S$
Molecular Weight: 433.52272
ALogP: 4.245
Rotatable Bonds: 8
Acceptors: 5
Donors: 1

Model Prediction

Prediction: 0.032
Unit: g/kg_body_weight
Mahalanobis Distance: 36.074
Mahalanobis Distance p-value: 1.65e-035

Mahalanobis Distance: The Mahalanobis distance (MD) is a generalization of the Euclidean distance that accounts for correlations among the X properties. It is calculated as the distance to the center of the training data. The larger the MD, the less trustworthy the prediction.
Mahalanobis Distance p-value: The p-value gives the fraction of training data with an MD greater than or equal to the one for the given sample, assuming normally distributed data. The smaller the p-value, the less trustworthy the prediciton. For highly non-normal X properties (e.g., fingerprints), the MD p-value is wildly inaccurate.

Structural Similar Compounds

Name	ASSURE	FLUVALINATE	DILTIAZEM
Structure	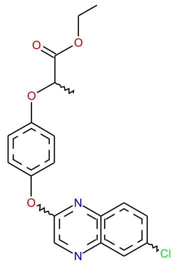	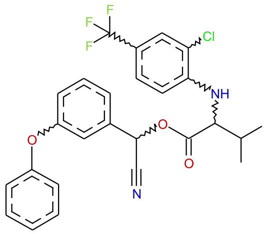	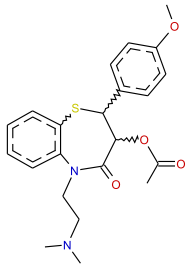
Actual Endpoint (-log C)	5.00328	5.30356	4.21961
Predicted Endpoint (-log C)	4.27671	4.89944	4.005
Distance	0.688	0.765	0.767
Reference	EPA COVER SHEET 0335;891001;(1)	EPA COVER SHEET 0281;880630;(1)	NDA-18602

Model Applicability

Unknown features are fingerprint features in the query molecule, but not found in the training set.

1. All properties and OPS components are within expected ranges.
2. Unknown ECFP_6 feature: -830332112: [*]S[*]
3. Unknown ECFP_6 feature: -955816473: [*]SCC(=[*])[*]
4. Unknown ECFP_6 feature: 1731843802: [*]CC(=O)N[*]
5. Unknown ECFP_6 feature: 497523368: [*]CNC(=[*])[*]
6. Unknown ECFP_6 feature: 769925792: [*]NC[c](:[*]):[*]
7. Unknown ECFP_6 feature: 1203316083: [*][c]1:[*]:[*]:[c]([*]):o:1
8. Unknown ECFP_6 feature: 1093109320: [*]S[c]1:o:[*]:[*]:n:1
9. Unknown ECFP_6 feature: 911256832: [*][c]1:[*]:[*]:n:n:1
10. Unknown ECFP_6 feature: 1427820655: [*]CS[c](:[*]):[*]
11. Unknown ECFP_6 feature: -178525456: [*]:[cH]:[c](:[cH]:[*]):[c](:[*]):[*]
12. Unknown ECFP_6 feature: -176846085: [*]C([*])[c](:[cH]:[*]):[cH]:[*]
13. Unknown ECFP_6 feature: 1307307440: [*]:[c](:[*])OC
14. Unknown ECFP_6 feature: -1841325949: [*]:[c](:[*])C(C)[c](:[*]):[*]
15. Unknown ECFP_6 feature: 1092541557: [*]C([*])[c]1:o:[*]:[*]:n:1
16. Unknown ECFP_6 feature: 1997021792: [*]:[cH]:[cH]:[cH]:[*]

Feature Contribution

Top features for positive contribution

Fingerprint	Bit/Smiles	Feature Structure	Score
ECFP_6	1559650422	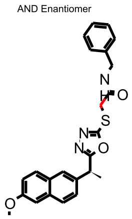 [*]C[*]	0.129
ECFP_6	-176455838	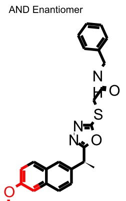 [*]O[c](:[cH]:[*]):[cH]:[*]	0.106
FCFP_6	3	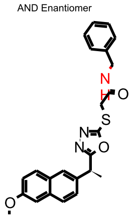 [*]N[*]	0.092
**Top Features for negative contribution**			
**Fingerprint**	**Bit/Smiles**	**Feature Structure**	**Score**
FCFP_6	991735244	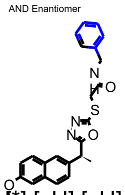 [*][c]1:[*]:[cH]:[cH]:[cH]:[cH]:1	-0.134

ECFP_6	1564392544	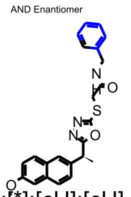<br>[*][c]1:[*]:[cH]:[cH]:[cH]:[cH]:1	-0.133
FCFP_6	1	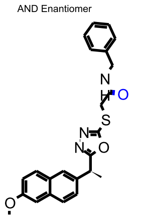<br>[*]=O	-0.102

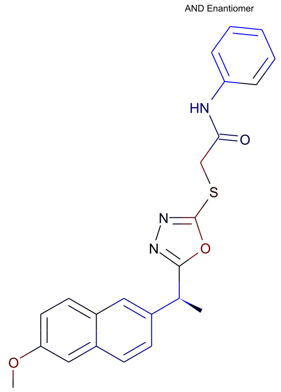

$C_{23}H_{21}N_3O_3S$
Molecular Weight: 419.49614
ALogP: 4.238
Rotatable Bonds: 7
Acceptors: 5
Donors: 1

Model Prediction

Prediction: 0.031
Unit: g/kg_body_weight
Mahalanobis Distance: 34.196
Mahalanobis Distance p-value: 3.24e-032

Mahalanobis Distance: The Mahalanobis distance (MD) is a generalization of the Euclidean distance that accounts for correlations among the X properties. It is calculated as the distance to the center of the training data. The larger the MD, the less trustworthy the prediction.
Mahalanobis Distance p-value: The p-value gives the fraction of training data with an MD greater than or equal to the one for the given sample, assuming normally distributed data. The smaller the p-value, the less trustworthy the prediciton. For highly non-normal X properties (e.g., fingerprints), the MD p-value is wildly inaccurate.

Structural Similar Compounds

Name	ASSURE	C.I. PIGMENT RED 3	D & C RED 9
Structure	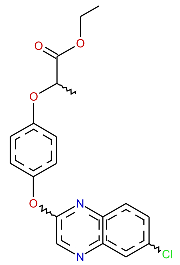	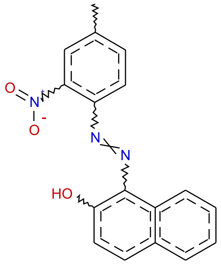	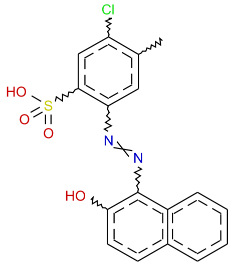
Actual Endpoint (-log C)	5.00328	3.0252	3.87715
Predicted Endpoint (-log C)	4.27671	3.34768	3.6546
Distance	0.671	0.745	0.745
Reference	EPA COVER SHEET 0335;891001;(1)	NTP REPORT # 407	NTP REPORT # 225

Model Applicability

Unknown features are fingerprint features in the query molecule, but not found in the training set.

1. All properties and OPS components are within expected ranges.
2. Unknown ECFP_6 feature: -830332112: [*]S[*]
3. Unknown ECFP_6 feature: -955816473: [*]SCC(=[*])[*]
4. Unknown ECFP_6 feature: 1731843802: [*]CC(=O)N[*]
5. Unknown ECFP_6 feature: -177077903: [*]N[c](:[cH]:[*]):[cH]:[*]
6. Unknown ECFP_6 feature: 1203316083: [*][c]1:[*]:[*]:[c]([*]):o:1
7. Unknown ECFP_6 feature: 1093109320: [*]S[c]1:o:[*]:[*]:n:1
8. Unknown ECFP_6 feature: 911256832: [*][c]1:[*]:[*]:n:n:1
9. Unknown ECFP_6 feature: 1427820655: [*]CS[c](:[*]):[*]
10. Unknown ECFP_6 feature: -178525456: [*]:[cH]:[c](:[cH]:[*]):[c](:[*]):[*]
11. Unknown ECFP_6 feature: -176846085: [*]C([*])[c](:[cH]:[*]):[cH]:[*]
12. Unknown ECFP_6 feature: 1307307440: [*]:[c](:[*])OC
13. Unknown ECFP_6 feature: -1841325949: [*]:[c](:[*])C(C)[c](:[*]):[*]
14. Unknown ECFP_6 feature: 1092541557: [*]C([*])[c]1:o:[*]:[*]:n:1
15. Unknown ECFP_6 feature: 1997021792: [*]:[cH]:[cH]:[cH]:[*]

Feature Contribution

Top features for positive contribution			
Fingerprint	Bit/Smiles	Feature Structure	Score

ECFP_6	1559650422	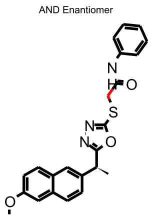 [*]C[*]	0.129
ECFP_6	-176455838	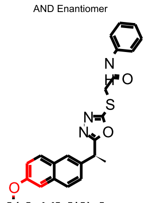 [*]O[c](:[cH]:[*]):[cH]:[*]	0.106
FCFP_6	3	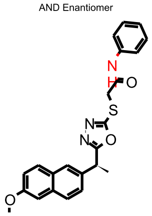 [*]N[*]	0.092
**Top Features for negative contribution**			
**Fingerprint**	**Bit/Smiles**	**Feature Structure**	**Score**
FCFP_6	991735244	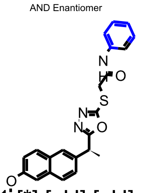 [*][c]1:[*]:[cH]:[cH]:[cH]:[cH]:1	-0.134
ECFP_6	1564392544	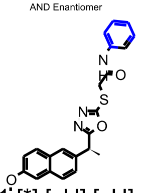 [*][c]1:[*]:[cH]:[cH]:[cH]:[cH]:1	-0.133

FCFP_6	1	AND Enantiomer 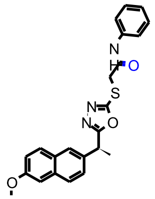 [*]=O	-0.102

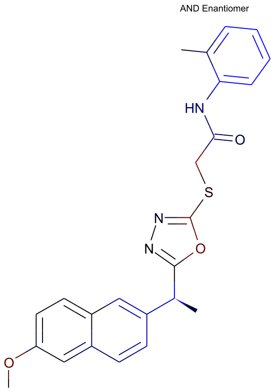

$C_{24}H_{23}N_{3}O_{3}S$
Molecular Weight: 433.52272
ALogP: 4.724
Rotatable Bonds: 7
Acceptors: 5
Donors: 1

Model Prediction

Prediction: 0.041
Unit: g/kg_body_weight
Mahalanobis Distance: 36.108
Mahalanobis Distance p-value: 1.45e-035

Mahalanobis Distance: The Mahalanobis distance (MD) is a generalization of the Euclidean distance that accounts for correlations among the X properties. It is calculated as the distance to the center of the training data. The larger the MD, the less trustworthy the prediction.
Mahalanobis Distance p-value: The p-value gives the fraction of training data with an MD greater than or equal to the one for the given sample, assuming normally distributed data. The smaller the p-value, the less trustworthy the prediciton. For highly non-normal X properties (e.g., fingerprints), the MD p-value is wildly inaccurate.

Structural Similar Compounds

Name	ASSURE	D & C RED 9	FLUVALINATE
Structure	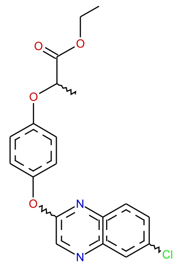	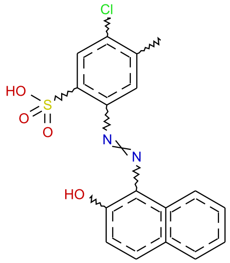	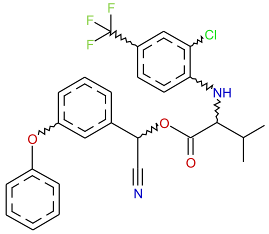
Actual Endpoint (-log C)	5.00328	3.87715	5.30356
Predicted Endpoint (-log C)	4.27671	3.6546	4.89944
Distance	0.684	0.739	0.744
Reference	EPA COVER SHEET 0335;891001;(1)	NTP REPORT # 225	EPA COVER SHEET 0281;880630;(1)

Model Applicability

Unknown features are fingerprint features in the query molecule, but not found in the training set.

1. All properties and OPS components are within expected ranges.
2. Unknown ECFP_6 feature: -830332112: [*]S[*]
3. Unknown ECFP_6 feature: -955816473: [*]SCC(=[*])[*]
4. Unknown ECFP_6 feature: 1731843802: [*]CC(=O)N[*]
5. Unknown ECFP_6 feature: 1335108269: [*]N[c](:[cH]:[*]):[c]([*]):[*]
6. Unknown ECFP_6 feature: 1203316083: [*][c]1:[*]:[*]:[c]([*]):o:1
7. Unknown ECFP_6 feature: 1093109320: [*]S[c]1:o:[*]:[*]:n:1
8. Unknown ECFP_6 feature: 911256832: [*][c]1:[*]:[*]:n:n:1
9. Unknown ECFP_6 feature: 1427820655: [*]CS[c](:[*]):[*]
10. Unknown ECFP_6 feature: -178525456: [*]:[cH]:[c](:[cH]:[*]):[c](:[*]):[*]
11. Unknown ECFP_6 feature: -176846085: [*]C([*])[c](:[cH]:[*]):[cH]:[*]
12. Unknown ECFP_6 feature: 1307307440: [*]:[c](:[*])OC
13. Unknown ECFP_6 feature: -1841325949: [*]:[c](:[*])C(C)[c](:[*]):[*]
14. Unknown ECFP_6 feature: 1092541557: [*]C([*])[c]1:o:[*]:[*]:n:1
15. Unknown ECFP_6 feature: 1997021792: [*]:[cH]:[cH]:[cH]:[*]

Feature Contribution

Top features for positive contribution			
Fingerprint	Bit/Smiles	Feature Structure	Score

ECFP_6	1559650422	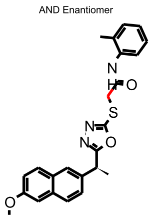 [*]C[*]	0.129
ECFP_6	-176455838	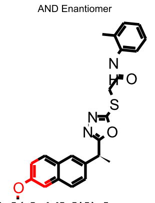 [*]O[c](:[cH]:[*]):[cH]:[*]	0.106
ECFP_6	2147419938	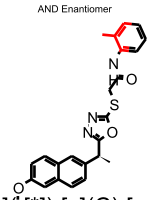 [*][c](:[*]):[c](C):[cH]:[*]	0.098
**Top Features for negative contribution**			
**Fingerprint**	**Bit/Smiles**	**Feature Structure**	**Score**
FCFP_6	991735244	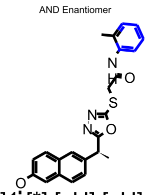 [*][c]1:[*]:[cH]:[cH]:[cH]:[cH]:1	-0.134
ECFP_6	1564392544	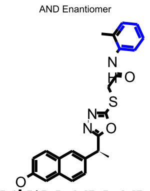 [*][c]1:[*]:[cH]:[cH]:[cH]:[cH]:1	-0.133

FCFP_6	1	AND Enantiomer 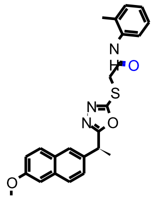 [*]=O	-0.102

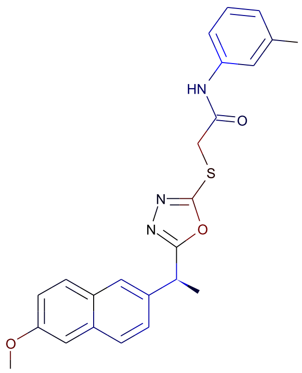

$C_{24}H_{23}N_3O_3S$
Molecular Weight: 433.52272
ALogP: 4.724
Rotatable Bonds: 7
Acceptors: 5
Donors: 1

Model Prediction

Prediction: 0.020
Unit: g/kg_body_weight
Mahalanobis Distance: 34.838
Mahalanobis Distance p-value: 2.39e-033

Mahalanobis Distance: The Mahalanobis distance (MD) is a generalization of the Euclidean distance that accounts for correlations among the X properties. It is calculated as the distance to the center of the training data. The larger the MD, the less trustworthy the prediction.
Mahalanobis Distance p-value: The p-value gives the fraction of training data with an MD greater than or equal to the one for the given sample, assuming normally distributed data. The smaller the p-value, the less trustworthy the prediciton. For highly non-normal X properties (e.g., fingerprints), the MD p-value is wildly inaccurate.

Structural Similar Compounds

Name	ASSURE	D & C RED 9	C.I. PIGMENT RED 3
Structure	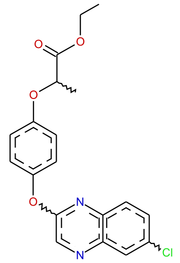	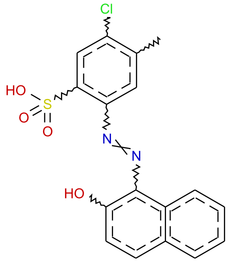	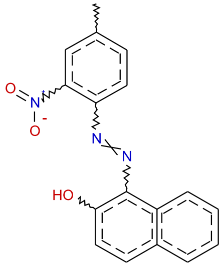
Actual Endpoint (-log C)	5.00328	3.87715	3.0252
Predicted Endpoint (-log C)	4.27671	3.6546	3.34768
Distance	0.682	0.746	0.750
Reference	EPA COVER SHEET 0335;891001;(1)	NTP REPORT # 225	NTP REPORT # 407

Model Applicability

Unknown features are fingerprint features in the query molecule, but not found in the training set.

1. All properties and OPS components are within expected ranges.
2. Unknown ECFP_6 feature: -830332112: [*]S[*]
3. Unknown ECFP_6 feature: -955816473: [*]SCC(=[*])[*]
4. Unknown ECFP_6 feature: 1731843802: [*]CC(=O)N[*]
5. Unknown ECFP_6 feature: -177077903: [*]N[c](:[cH]:[*]):[cH]:[*]
6. Unknown ECFP_6 feature: 1203316083: [*][c]1:[*]:[*]:[c]([*]):o:1
7. Unknown ECFP_6 feature: 1093109320: [*]S[c]1:o:[*]:[*]:n:1
8. Unknown ECFP_6 feature: 911256832: [*][c]1:[*]:[*]:n:n:1
9. Unknown ECFP_6 feature: 1427820655: [*]CS[c](:[*]):[*]
10. Unknown ECFP_6 feature: -178525456: [*]:[cH]:[c](:[cH]:[*]):[c](:[*]):[*]
11. Unknown ECFP_6 feature: -176846085: [*]C([*])[c](:[cH]:[*]):[cH]:[*]
12. Unknown ECFP_6 feature: 1307307440: [*]:[c](:[*])OC
13. Unknown ECFP_6 feature: -1841325949: [*]:[c](:[*])C(C)[c](:[*]):[*]
14. Unknown ECFP_6 feature: 1092541557: [*]C([*])[c]1:o:[*]:[*]:n:1
15. Unknown ECFP_6 feature: 1997021792: [*]:[cH]:[cH]:[cH]:[*]
16. Unknown ECFP_6 feature: -179515162: [*]:[cH]:[c](C):[cH]:[*]

Feature Contribution

Top features for positive contribution

Fingerprint	Bit/Smiles	Feature Structure	Score
ECFP_6	1559650422	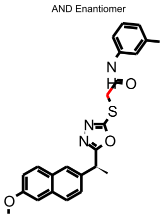 [*]C[*]	0.129
ECFP_6	-176455838	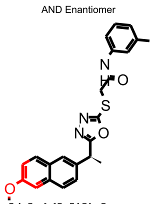 [*]O[c](:[cH]:[*]):[cH]:[*]	0.106
FCFP_6	3	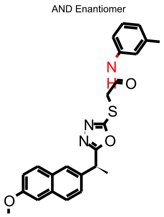 [*]N[*]	0.092
**Top Features for negative contribution**			
**Fingerprint**	**Bit/Smiles**	**Feature Structure**	**Score**
FCFP_6	991735244	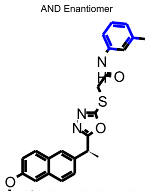 [*][c]1:[*]:[cH]:[cH]:[cH]:[cH]:1	-0.134

FCFP_6	1	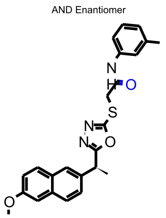 [*]=O	-0.102
FCFP_6	-453677277	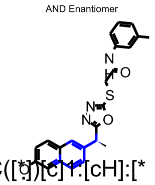 [*]C([*])[c]1:[cH]:[*]:[c](:[*]):[cH]:[cH]:1	-0.091

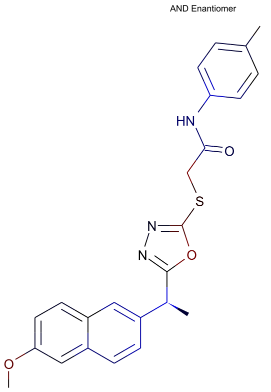

$C_{24}H_{23}N_3O_3S$
Molecular Weight: 433.52272
ALogP: 4.724
Rotatable Bonds: 7
Acceptors: 5
Donors: 1

Model Prediction

Prediction: 0.017
Unit: g/kg_body_weight
Mahalanobis Distance: 35.392
Mahalanobis Distance p-value: 2.55e-034

Mahalanobis Distance: The Mahalanobis distance (MD) is a generalization of the Euclidean distance that accounts for correlations among the X properties. It is calculated as the distance to the center of the training data. The larger the MD, the less trustworthy the prediction.
Mahalanobis Distance p-value: The p-value gives the fraction of training data with an MD greater than or equal to the one for the given sample, assuming normally distributed data. The smaller the p-value, the less trustworthy the prediciton. For highly non-normal X properties (e.g., fingerprints), the MD p-value is wildly inaccurate.

Structural Similar Compounds

Name	ASSURE	D & C RED 9	C.I. PIGMENT RED 3
Structure	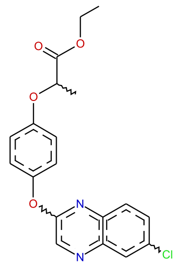	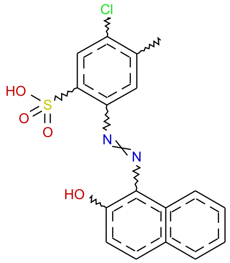	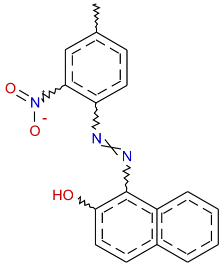
Actual Endpoint (-log C)	5.00328	3.87715	3.0252
Predicted Endpoint (-log C)	4.27671	3.6546	3.34768
Distance	0.681	0.748	0.752
Reference	EPA COVER SHEET 0335;891001;(1)	NTP REPORT # 225	NTP REPORT # 407

Model Applicability

Unknown features are fingerprint features in the query molecule, but not found in the training set.

1. All properties and OPS components are within expected ranges.
2. Unknown ECFP_6 feature: -830332112: [*]S[*]
3. Unknown ECFP_6 feature: -955816473: [*]SCC(=[*])[*]
4. Unknown ECFP_6 feature: 1731843802: [*]CC(=O)N[*]
5. Unknown ECFP_6 feature: -177077903: [*]N[c](:[cH]:[*]):[cH]:[*]
6. Unknown ECFP_6 feature: 1203316083: [*][c]1:[*]:[*]:[c]([*]):o:1
7. Unknown ECFP_6 feature: 1093109320: [*]S[c]1:o:[*]:[*]:n:1
8. Unknown ECFP_6 feature: 911256832: [*][c]1:[*]:[*]:n:n:1
9. Unknown ECFP_6 feature: 1427820655: [*]CS[c](:[*]):[*]
10. Unknown ECFP_6 feature: -178525456: [*]:[cH]:[c](:[cH]:[*]):[c](:[*]):[*]
11. Unknown ECFP_6 feature: -176846085: [*]C([*])[c](:[cH]:[*]):[cH]:[*]
12. Unknown ECFP_6 feature: 1307307440: [*]:[c](:[*])OC
13. Unknown ECFP_6 feature: -1841325949: [*]:[c](:[*])C(C)[c](:[*]):[*]
14. Unknown ECFP_6 feature: 1092541557: [*]C([*])[c]1:o:[*]:[*]:n:1
15. Unknown ECFP_6 feature: -179515162: [*]:[cH]:[c](C):[cH]:[*]

Feature Contribution

Top features for positive contribution

Fingerprint	Bit/Smiles	Feature Structure	Score

ECFP_6	1559650422	AND Enantiomer 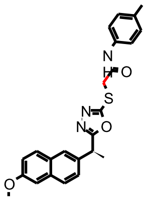 [*]C[*]	0.129
ECFP_6	-176455838	AND Enantiomer 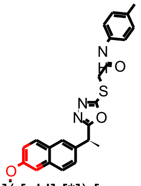 [*]O[c](:[cH]:[*]):[cH]:[*]	0.106
FCFP_6	3	AND Enantiomer 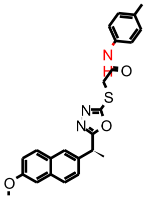 [*]N[*]	0.092

Top Features for negative contribution

Fingerprint	Bit/Smiles	Feature Structure	Score
FCFP_6	1	AND Enantiomer 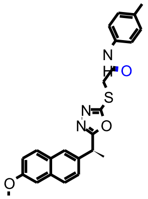 [*]=O	-0.102
FCFP_6	-453677277	AND Enantiomer 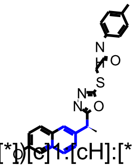 [*]C([*])[c]1:[cH]:[*]:[c](:[*]):[cH]:[cH]:1	-0.091

FCFP_6	136597326	AND Enantiomer 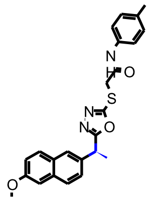 [*]C([*])C	-0.081

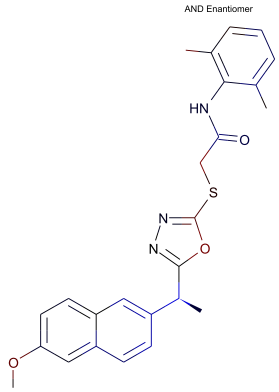

$C_{25}H_{25}N_3O_3S$
Molecular Weight: 447.5493
ALogP: 5.21
Rotatable Bonds: 7
Acceptors: 5
Donors: 1

Model Prediction

Prediction: 0.016
Unit: g/kg_body_weight
Mahalanobis Distance: 34.715
Mahalanobis Distance p-value: 3.93e-033

Mahalanobis Distance: The Mahalanobis distance (MD) is a generalization of the Euclidean distance that accounts for correlations among the X properties. It is calculated as the distance to the center of the training data. The larger the MD, the less trustworthy the prediction.
Mahalanobis Distance p-value: The p-value gives the fraction of training data with an MD greater than or equal to the one for the given sample, assuming normally distributed data. The smaller the p-value, the less trustworthy the prediciton. For highly non-normal X properties (e.g., fingerprints), the MD p-value is wildly inaccurate.

Structural Similar Compounds

Name	ASSURE	FLUVALINATE	D & C RED 9
Structure	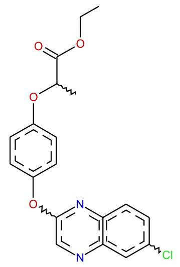	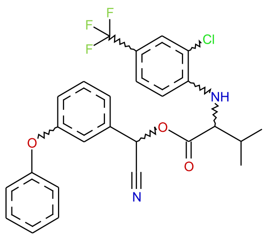	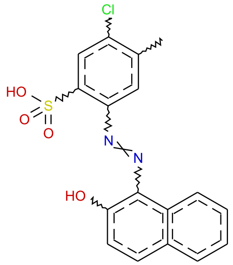
Actual Endpoint (-log C)	5.00328	5.30356	3.87715
Predicted Endpoint (-log C)	4.27671	4.89944	3.6546
Distance	0.700	0.715	0.749
Reference	EPA COVER SHEET 0335;891001;(1)	EPA COVER SHEET 0281;880630;(1)	NTP REPORT # 225

Model Applicability

Unknown features are fingerprint features in the query molecule, but not found in the training set.

1. All properties and OPS components are within expected ranges.
2. Unknown ECFP_6 feature: -830332112: [*]S[*]
3. Unknown ECFP_6 feature: -955816473: [*]SCC(=[*])[*]
4. Unknown ECFP_6 feature: 1731843802: [*]CC(=O)N[*]
5. Unknown ECFP_6 feature: -1660205591: [*]N[c](:[c]([*]):[*]):[c]([*]):[*]
6. Unknown ECFP_6 feature: 1203316083: [*][c]1:[*]:[*]:[c]([*]):o:1
7. Unknown ECFP_6 feature: 1093109320: [*]S[c]1:o:[*]:[*]:n:1
8. Unknown ECFP_6 feature: 911256832: [*][c]1:[*]:[*]:n:n:1
9. Unknown ECFP_6 feature: 1427820655: [*]CS[c](:[*]):[*]
10. Unknown ECFP_6 feature: -178525456: [*]:[cH]:[c](:[cH]:[*]):[c](:[*]):[*]
11. Unknown ECFP_6 feature: -176846085: [*]C([*])[c](:[cH]:[*]):[cH]:[*]
12. Unknown ECFP_6 feature: 1307307440: [*]:[c](:[*])OC
13. Unknown ECFP_6 feature: -1841325949: [*]:[c](:[*])C(C)[c](:[*]):[*]
14. Unknown ECFP_6 feature: 1092541557: [*]C([*])[c]1:o:[*]:[*]:n:1
15. Unknown ECFP_6 feature: 1997021792: [*]:[cH]:[cH]:[cH]:[*]

Feature Contribution

Top features for positive contribution			
Fingerprint	Bit/Smiles	Feature Structure	Score

ECFP_6	1559650422	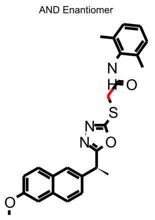 [*]C[*]	0.129
ECFP_6	-176455838	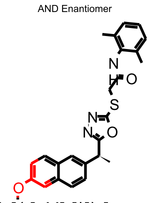 [*]O[c](:[cH]:[*]):[cH]:[*]	0.106
ECFP_6	2147419938	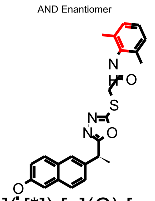 [*][c](:[*]):[c](C):[cH]:[*]	0.098
**Top Features for negative contribution**			
**Fingerprint**	**Bit/Smiles**	**Feature Structure**	**Score**
FCFP_6	991735244	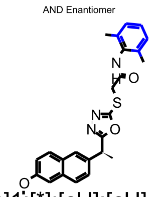 [*][c]1:[*]:[cH]:[cH]:[cH]:[cH]:1	-0.134
FCFP_6	1	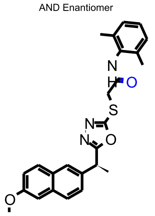 [*]=O	-0.102

FCFP_6	-453677277	AND Enantiomer 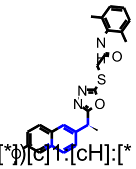 [*]C([*])[c]1:[cH]:[*]:[c](:[*]):[cH]:[cH]:1	-0.091

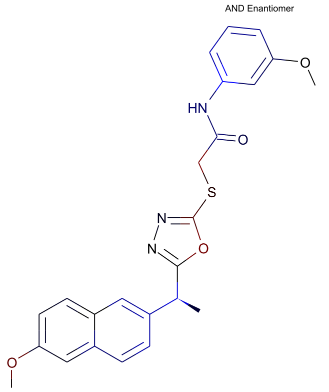

$C_{24}H_{23}N_3O_4S$

Molecular Weight: 449.52212

ALogP: 4.221

Rotatable Bonds: 8

Acceptors: 6

Donors: 1

Model Prediction

Prediction: 0.020

Unit: g/kg_body_weight

Mahalanobis Distance: 33.595

Mahalanobis Distance p-value: 3.77e-031

Mahalanobis Distance: The Mahalanobis distance (MD) is a generalization of the Euclidean distance that accounts for correlations among the X properties. It is calculated as the distance to the center of the training data. The larger the MD, the less trustworthy the prediction.
Mahalanobis Distance p-value: The p-value gives the fraction of training data with an MD greater than or equal to the one for the given sample, assuming normally distributed data. The smaller the p-value, the less trustworthy the prediciton. For highly non-normal X properties (e.g., fingerprints), the MD p-value is wildly inaccurate.

Structural Similar Compounds

Name	ASSURE	FLUVALINATE	DILTIAZEM
Structure	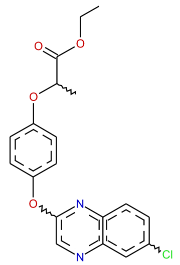	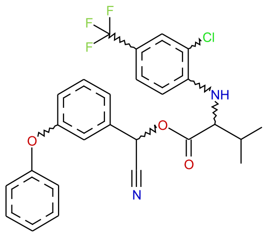	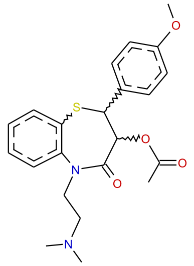
Actual Endpoint (-log C)	5.00328	5.30356	4.21961
Predicted Endpoint (-log C)	4.27671	4.89944	4.005
Distance	0.712	0.769	0.777
Reference	EPA COVER SHEET 0335;891001;(1)	EPA COVER SHEET 0281;880630;(1)	NDA-18602

Model Applicability

Unknown features are fingerprint features in the query molecule, but not found in the training set.

1. All properties and OPS components are within expected ranges.
2. Unknown ECFP_6 feature: -830332112: [*]S[*]
3. Unknown ECFP_6 feature: -955816473: [*]SCC(=[*])[*]
4. Unknown ECFP_6 feature: 1731843802: [*]CC(=O)N[*]
5. Unknown ECFP_6 feature: -177077903: [*]N[c](:[cH]:[*]):[cH]:[*]
6. Unknown ECFP_6 feature: 1203316083: [*][c]1:[*]:[*]:[c]([*]):o:1
7. Unknown ECFP_6 feature: 1093109320: [*]S[c]1:o:[*]:[*]:n:1
8. Unknown ECFP_6 feature: 911256832: [*][c]1:[*]:[*]:n:n:1
9. Unknown ECFP_6 feature: 1427820655: [*]CS[c](:[*]):[*]
10. Unknown ECFP_6 feature: -178525456: [*]:[cH]:[c](:[cH]:[*]):[c](:[*]):[*]
11. Unknown ECFP_6 feature: -176846085: [*]C([*])[c](:[cH]:[*]):[cH]:[*]
12. Unknown ECFP_6 feature: 1307307440: [*]:[c](:[*])OC
13. Unknown ECFP_6 feature: -1841325949: [*]:[c](:[*])C(C)[c](:[*]):[*]
14. Unknown ECFP_6 feature: 1092541557: [*]C([*])[c]1:o:[*]:[*]:n:1
15. Unknown ECFP_6 feature: 1997021792: [*]:[cH]:[cH]:[cH]:[*]

Feature Contribution

Top features for positive contribution			
Fingerprint	Bit/Smiles	Feature Structure	Score

ECFP_6	1559650422	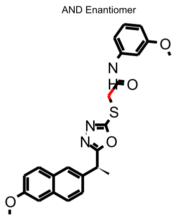 [*]C[*]	0.129
ECFP_6	-176455838	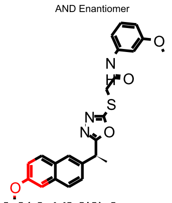 [*]O[c](:[cH]:[*]):[cH]:[*]	0.106
FCFP_6	3	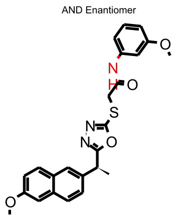 [*]N[*]	0.092
**Top Features for negative contribution**			
**Fingerprint**	**Bit/Smiles**	**Feature Structure**	**Score**
FCFP_6	991735244	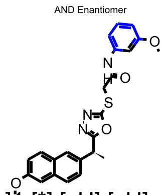 [*][c]1:[*]:[cH]:[cH]:[cH]:[cH]:1	-0.134
FCFP_6	1	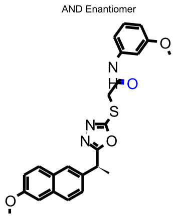 [*]=O	-0.102

FCFP_6	-453677277	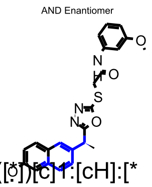 [*]C([*])[c]1:[cH]:[*]:[c](:[*]):[cH]:[cH]:1	-0.091

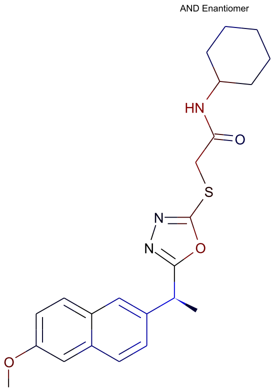

$C_{23}H_{27}N_3O_3S$
Molecular Weight: 425.54378
ALogP: 4.52
Rotatable Bonds: 7
Acceptors: 5
Donors: 1

Model Prediction

Prediction: 0.026
Unit: g/kg_body_weight
Mahalanobis Distance: 35.582
Mahalanobis Distance p-value: 1.19e-034

Mahalanobis Distance: The Mahalanobis distance (MD) is a generalization of the Euclidean distance that accounts for correlations among the X properties. It is calculated as the distance to the center of the training data. The larger the MD, the less trustworthy the prediction.
Mahalanobis Distance p-value: The p-value gives the fraction of training data with an MD greater than or equal to the one for the given sample, assuming normally distributed data. The smaller the p-value, the less trustworthy the prediciton. For highly non-normal X properties (e.g., fingerprints), the MD p-value is wildly inaccurate.

Structural Similar Compounds

Name	ASSURE	DILTIAZEM	ISOXABEN
Structure	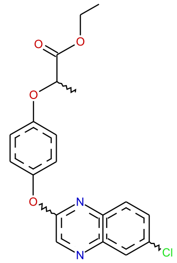	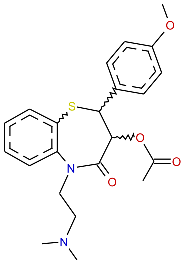	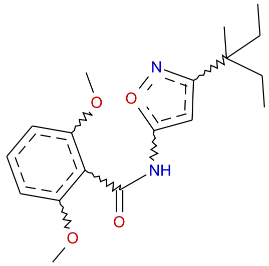
Actual Endpoint (-log C)	5.00328	4.21961	3.81665
Predicted Endpoint (-log C)	4.27671	4.005	4.42315
Distance	0.623	0.647	0.649
Reference	EPA COVER SHEET 0335;891001;(1)	NDA-18602	EPA COVER SHEET 0339;881201;(1)

Model Applicability

Unknown features are fingerprint features in the query molecule, but not found in the training set.

1. All properties and OPS components are within expected ranges.
2. Unknown ECFP_6 feature: -830332112: [*]S[*]
3. Unknown ECFP_6 feature: -955816473: [*]SCC(=[*])[*]
4. Unknown ECFP_6 feature: 1731843802: [*]CC(=O)N[*]
5. Unknown ECFP_6 feature: -2091181441: [*]C([*])NC(=[*])[*]
6. Unknown ECFP_6 feature: -859078569: [*]CC(C[*])N[*]
7. Unknown ECFP_6 feature: 1203316083: [*][c]1:[*]:[*]:[c]([*]):o:1
8. Unknown ECFP_6 feature: 1093109320: [*]S[c]1:o:[*]:[*]:n:1
9. Unknown ECFP_6 feature: 911256832: [*][c]1:[*]:[*]:n:n:1
10. Unknown ECFP_6 feature: 1427820655: [*]CS[c](:[*]):[*]
11. Unknown ECFP_6 feature: -178525456: [*]:[cH]:[c](:[cH]:[*]):[c](:[*]):[*]
12. Unknown ECFP_6 feature: -176846085: [*]C([*])[c](:[cH]:[*]):[cH]:[*]
13. Unknown ECFP_6 feature: 1307307440: [*]:[c](:[*])OC
14. Unknown ECFP_6 feature: -1841325949: [*]:[c](:[*])C(C)[c](:[*]):[*]
15. Unknown ECFP_6 feature: 1092541557: [*]C([*])[c]1:o:[*]:[*]:n:1
16. Unknown ECFP_6 feature: -1332781180: [*]CCC[*]

Feature Contribution

Top features for positive contribution

Fingerprint	Bit/Smiles	Feature Structure	Score
ECFP_6	-167460056	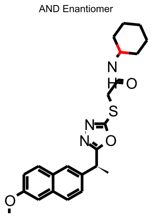 [*]C([*])[*]	0.136
ECFP_6	1559650422	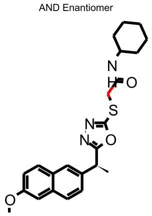 [*]C[*]	0.129
ECFP_6	-176455838	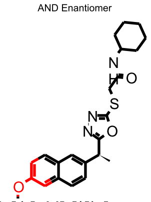 [*]O[c](:[cH]:[*]):[cH]:[*]	0.106

Top Features for negative contribution

Fingerprint	Bit/Smiles	Feature Structure	Score
FCFP_6	1	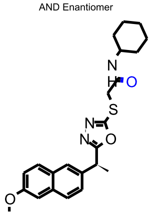 [*]=O	-0.102

FCFP_6	-453677277	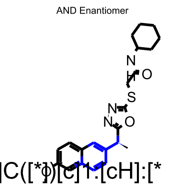<br>[*]C([*])[c]1:[cH]:[*]:[c](:[*]):[cH]:[cH]:1	-0.091
FCFP_6	136597326	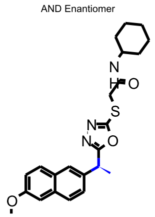<br>[*]C([*])C	-0.081

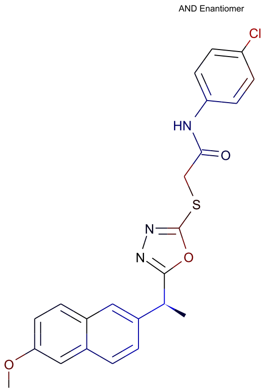

$C_{23}H_{20}ClN_3O_3S$

Molecular Weight: 453.9412

ALogP: 4.902

Rotatable Bonds: 7

Acceptors: 5

Donors: 1

Structural Similar Compounds

Name	ASSURE	FLUVALINATE	D & C RED 9
Structure	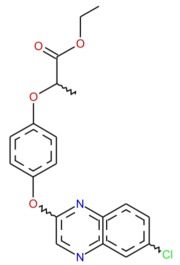	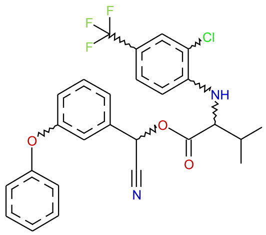	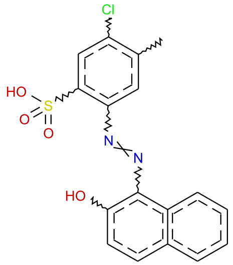
Actual Endpoint (-log C)	5.00328	5.30356	3.87715
Predicted Endpoint (-log C)	4.27671	4.89944	3.6546
Distance	0.667	0.722	0.741
Reference	EPA COVER SHEET 0335;891001;(1)	EPA COVER SHEET 0281;880630;(1)	NTP REPORT # 225

Model Prediction

Prediction: 0.012

Unit: g/kg_body_weight

Mahalanobis Distance: 35.232

Mahalanobis Distance p-value: 4.86e-034

Mahalanobis Distance: The Mahalanobis distance (MD) is a generalization of the Euclidean distance that accounts for correlations among the X properties. It is calculated as the distance to the center of the training data. The larger the MD, the less trustworthy the prediction.
Mahalanobis Distance p-value: The p-value gives the fraction of training data with an MD greater than or equal to the one for the given sample, assuming normally distributed data. The smaller the p-value, the less trustworthy the prediciton. For highly non-normal X properties (e.g., fingerprints), the MD p-value is wildly inaccurate.

Model Applicability

Unknown features are fingerprint features in the query molecule, but not found in the training set.

1. All properties and OPS components are within expected ranges.
2. Unknown ECFP_6 feature: -830332112: [*]S[*]
3. Unknown ECFP_6 feature: -955816473: [*]SCC(=[*])[*]
4. Unknown ECFP_6 feature: 1731843802: [*]CC(=O)N[*]
5. Unknown ECFP_6 feature: -177077903: [*]N[c](:[cH]:[*]):[cH]:[*]
6. Unknown ECFP_6 feature: 1203316083: [*][c]1:[*]:[*]:[c]([*]):o:1
7. Unknown ECFP_6 feature: 1093109320: [*]S[c]1:o:[*]:[*]:n:1
8. Unknown ECFP_6 feature: 911256832: [*][c]1:[*]:[*]:n:n:1
9. Unknown ECFP_6 feature: 1427820655: [*]CS[c](:[*]):[*]
10. Unknown ECFP_6 feature: -178525456: [*]:[cH]:[c](:[cH]:[*]):[c](:[*]):[*]
11. Unknown ECFP_6 feature: -176846085: [*]C([*])[c](:[cH]:[*]):[cH]:[*]
12. Unknown ECFP_6 feature: 1307307440: [*]:[c](:[*])OC
13. Unknown ECFP_6 feature: -1841325949: [*]:[c](:[*])C(C)[c](:[*]):[*]
14. Unknown ECFP_6 feature: 1092541557: [*]C([*])[c]1:o:[*]:[*]:n:1
15. Unknown ECFP_6 feature: -176494269: [*]:[cH]:[c](Cl):[cH]:[*]
16. Unknown ECFP_6 feature: 99947387: [*]:[c](:[*])Cl

Feature Contribution

Top features for positive contribution

Fingerprint	Bit/Smiles	Feature Structure	Score
ECFP_6	1559650422	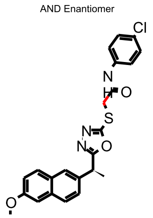 [*]C[*]	0.129
ECFP_6	-176455838	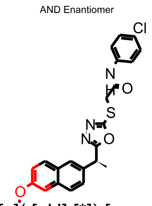 [*]O[c](:[cH]:[*]):[cH]:[*]	0.106
FCFP_6	32	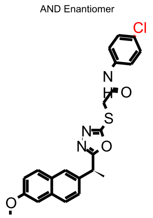 [*]Cl	0.101

Top Features for negative contribution

Fingerprint	Bit/Smiles	Feature Structure	Score
FCFP_6	1	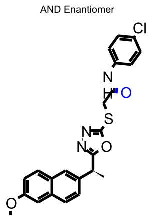 [*]=O	-0.102

FCFP_6	-453677277	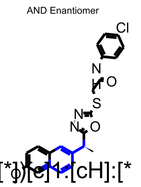 [*]C([*])[c]1:[cH]:[*]:[c](:[*]):[cH]:[cH]:1	-0.091
FCFP_6	136597326	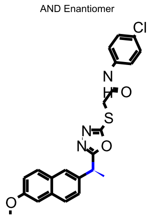 [*]C([*])C	-0.081

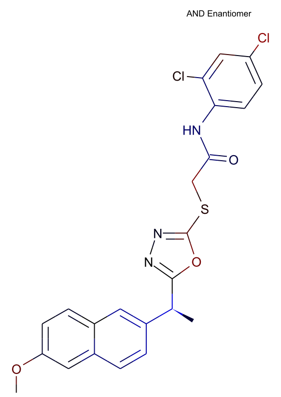

$C_{23}H_{19}Cl_2N_3O_3S$
Molecular Weight: 488.38626
ALogP: 5.567
Rotatable Bonds: 7
Acceptors: 5
Donors: 1

Model Prediction

Prediction: 0.014
Unit: g/kg_body_weight
Mahalanobis Distance: 34.961
Mahalanobis Distance p-value: 1.45e-033

Mahalanobis Distance: The Mahalanobis distance (MD) is a generalization of the Euclidean distance that accounts for correlations among the X properties. It is calculated as the distance to the center of the training data. The larger the MD, the less trustworthy the prediction.
Mahalanobis Distance p-value: The p-value gives the fraction of training data with an MD greater than or equal to the one for the given sample, assuming normally distributed data. The smaller the p-value, the less trustworthy the prediciton. For highly non-normal X properties (e.g., fingerprints), the MD p-value is wildly inaccurate.

Structural Similar Compounds

Name	FLUVALINATE	ASSURE	D & C RED 9
Structure	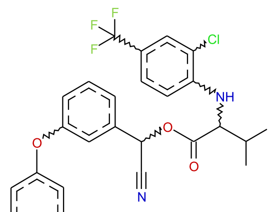	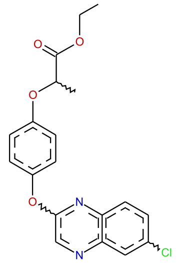	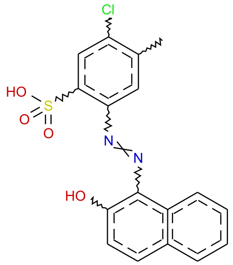
Actual Endpoint (-log C)	5.30356	5.00328	3.87715
Predicted Endpoint (-log C)	4.89944	4.27671	3.6546
Distance	0.671	0.704	0.759
Reference	EPA COVER SHEET 0281;880630;(1)	EPA COVER SHEET 0335;891001;(1)	NTP REPORT # 225

Model Applicability

Unknown features are fingerprint features in the query molecule, but not found in the training set.

1. All properties and OPS components are within expected ranges.
2. Unknown ECFP_6 feature: -830332112: [*]S[*]
3. Unknown ECFP_6 feature: -955816473: [*]SCC(=[*])[*]
4. Unknown ECFP_6 feature: 1731843802: [*]CC(=O)N[*]
5. Unknown ECFP_6 feature: 1335108269: [*]N[c](:[cH]:[*]):[c]([*]):[*]
6. Unknown ECFP_6 feature: 1203316083: [*][c]1:[*]:[*]:[c]([*]):o:1
7. Unknown ECFP_6 feature: 1093109320: [*]S[c]1:o:[*]:[*]:n:1
8. Unknown ECFP_6 feature: 911256832: [*][c]1:[*]:[*]:n:n:1
9. Unknown ECFP_6 feature: 1427820655: [*]CS[c](:[*]):[*]
10. Unknown ECFP_6 feature: -178525456: [*]:[cH]:[c](:[cH]:[*]):[c](:[*]):[*]
11. Unknown ECFP_6 feature: -176846085: [*]C([*])[c](:[cH]:[*]):[cH]:[*]
12. Unknown ECFP_6 feature: 1307307440: [*]:[c](:[*])OC
13. Unknown ECFP_6 feature: -1841325949: [*]:[c](:[*])C(C)[c](:[*]):[*]
14. Unknown ECFP_6 feature: 1092541557: [*]C([*])[c]1:o:[*]:[*]:n:1
15. Unknown ECFP_6 feature: -176494269: [*]:[cH]:[c](Cl):[cH]:[*]
16. Unknown ECFP_6 feature: 99947387: [*]:[c](:[*])Cl

Feature Contribution

Top features for positive contribution

Fingerprint	Bit/Smiles	Feature Structure	Score
ECFP_6	1559650422	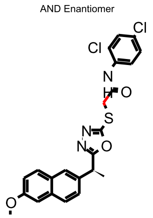 [*]C[*]	0.129
ECFP_6	-176455838	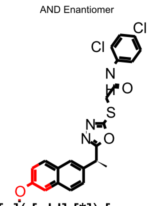 [*]O[c](:[cH]:[*]):[cH]:[*]	0.106
FCFP_6	32	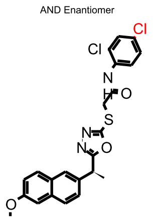 [*]Cl	0.101

Top Features for negative contribution

Fingerprint	Bit/Smiles	Feature Structure	Score
FCFP_6	1	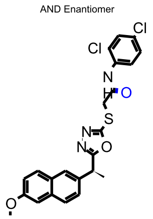 [*]=O	-0.102

FCFP_6	-453677277	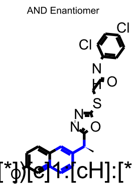 [*]C([*])[c]1:[cH]:[*]:[c](:[*]):[cH]:[cH]:1	-0.091
FCFP_6	136597326	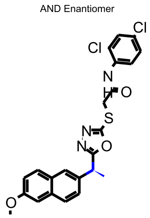 [*]C([*])C	-0.081

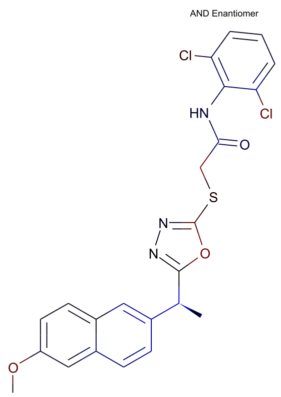

$C_{23}H_{19}Cl_2N_3O_3S$
Molecular Weight: 488.38626
ALogP: 5.567
Rotatable Bonds: 7
Acceptors: 5
Donors: 1

Model Prediction

Prediction: 0.015
Unit: g/kg_body_weight
Mahalanobis Distance: 35.428
Mahalanobis Distance p-value: 2.21e-034

Mahalanobis Distance: The Mahalanobis distance (MD) is a generalization of the Euclidean distance that accounts for correlations among the X properties. It is calculated as the distance to the center of the training data. The larger the MD, the less trustworthy the prediction.
Mahalanobis Distance p-value: The p-value gives the fraction of training data with an MD greater than or equal to the one for the given sample, assuming normally distributed data. The smaller the p-value, the less trustworthy the prediciton. For highly non-normal X properties (e.g., fingerprints), the MD p-value is wildly inaccurate.

Structural Similar Compounds

Name	FLUVALINATE	ASSURE	D & C RED 9
Structure	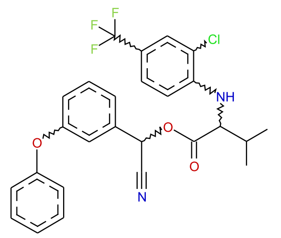	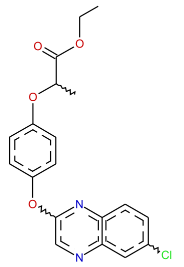	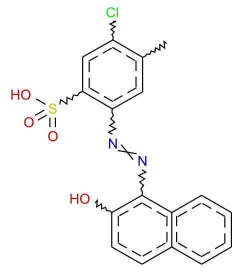
Actual Endpoint (-log C)	5.30356	5.00328	3.87715
Predicted Endpoint (-log C)	4.89944	4.27671	3.6546
Distance	0.673	0.709	0.755
Reference	EPA COVER SHEET 0281;880630;(1)	EPA COVER SHEET 0335;891001;(1)	NTP REPORT # 225

Model Applicability

Unknown features are fingerprint features in the query molecule, but not found in the training set.

1. All properties and OPS components are within expected ranges.
2. Unknown ECFP_6 feature: -830332112: [*]S[*]
3. Unknown ECFP_6 feature: -955816473: [*]SCC(=[*])[*]
4. Unknown ECFP_6 feature: 1731843802: [*]CC(=O)N[*]
5. Unknown ECFP_6 feature: -1660205591: [*]N[c](:[c]([*]):[*]):[c]([*]):[*]
6. Unknown ECFP_6 feature: 1203316083: [*][c]1:[*]:[*]:[c]([*]):o:1
7. Unknown ECFP_6 feature: 1093109320: [*]S[c]1:o:[*]:[*]:n:1
8. Unknown ECFP_6 feature: 911256832: [*][c]1:[*]:[*]:n:n:1
9. Unknown ECFP_6 feature: 1427820655: [*]CS[c](:[*]):[*]
10. Unknown ECFP_6 feature: -178525456: [*]:[cH]:[c](:[cH]:[*]):[c](:[*]):[*]
11. Unknown ECFP_6 feature: -176846085: [*]C([*])[c](:[cH]:[*]):[cH]:[*]
12. Unknown ECFP_6 feature: 1307307440: [*]:[c](:[*])OC
13. Unknown ECFP_6 feature: -1841325949: [*]:[c](:[*])C(C)[c](:[*]):[*]
14. Unknown ECFP_6 feature: 1092541557: [*]C([*])[c]1:o:[*]:[*]:n:1
15. Unknown ECFP_6 feature: 1997021792: [*]:[cH]:[cH]:[cH]:[*]
16. Unknown ECFP_6 feature: 99947387: [*]:[c](:[*])Cl

Feature Contribution

Top features for positive contribution

Fingerprint	Bit/Smiles	Feature Structure	Score
ECFP_6	1559650422	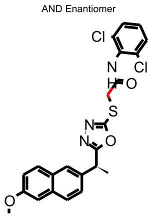 [*]C[*]	0.129
ECFP_6	-176455838	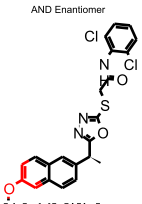 [*]O[c](:[cH]:[*]):[cH]:[*]	0.106
FCFP_6	32	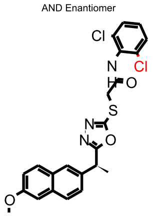 [*]Cl	0.101
**Top Features for negative contribution**			
**Fingerprint**	**Bit/Smiles**	**Feature Structure**	**Score**
FCFP_6	991735244	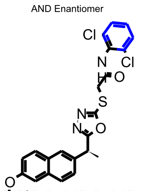 [*][c]1:[*]:[cH]:[cH]:[cH]:[cH]:1	-0.134

FCFP_6	1	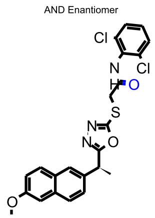<br>[*]=O	-0.102
FCFP_6	-453677277	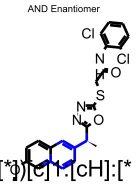<br>[*]C([*])[c]1:[cH]:[*]:[c](:[*]):[cH]:[cH]:1	-0.091

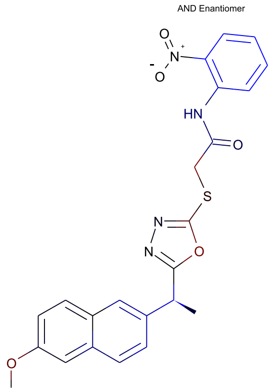

$C_{23}H_{20}N_4O_5S$
Molecular Weight: 464.4937
ALogP: 4.132
Rotatable Bonds: 8
Acceptors: 7
Donors: 1

Structural Similar Compounds

Name	C.I.PIGMENT RED 23	C.I. ACID RED 14	C.I. ACID ORANGE 3
Structure	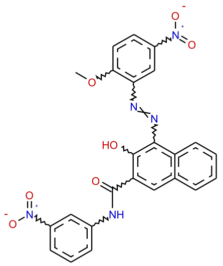	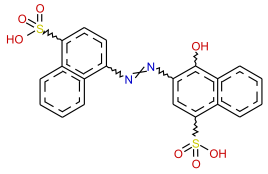	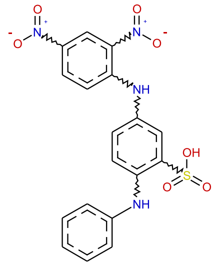
Actual Endpoint (-log C)	2.28997	2.8654	3.20573
Predicted Endpoint (-log C)	3.52921	3.29295	3.55956
Distance	0.622	0.784	0.799
Reference	NTP 411 146	NTP REPORT # 220	NTP REPORT # 335

Model Prediction

Prediction: 0.028
Unit: g/kg_body_weight
Mahalanobis Distance: 36.726
Mahalanobis Distance p-value: 1.24e-036

Mahalanobis Distance: The Mahalanobis distance (MD) is a generalization of the Euclidean distance that accounts for correlations among the X properties. It is calculated as the distance to the center of the training data. The larger the MD, the less trustworthy the prediction.
Mahalanobis Distance p-value: The p-value gives the fraction of training data with an MD greater than or equal to the one for the given sample, assuming normally distributed data. The smaller the p-value, the less trustworthy the prediciton. For highly non-normal X properties (e.g., fingerprints), the MD p-value is wildly inaccurate.

Model Applicability

Unknown features are fingerprint features in the query molecule, but not found in the training set.

1. All properties and OPS components are within expected ranges.
2. Unknown FCFP_2 feature: 5: [*][O-]
3. Unknown FCFP_2 feature: -828984032: [*][c](:[*]):[c]([N+](=[*])[*]):c:[*]
4. Unknown FCFP_2 feature: -1338588315: [*]:[c](:[*])[N+](=O)[O-]
5. Unknown FCFP_2 feature: 1872392852: [*][N+](=O)[*]
6. Unknown FCFP_2 feature: 260476081: [*][N+](=[*])[O-]
7. Unknown ECFP_6 feature: -830332112: [*]S[*]
8. Unknown ECFP_6 feature: 1043790491: [*][N+](=[*])[*]
9. Unknown ECFP_6 feature: 781519895: [*][O-]
10. Unknown ECFP_6 feature: -955816473: [*]SCC(=[*])[*]
11. Unknown ECFP_6 feature: 1731843802: [*]CC(=O)N[*]
12. Unknown ECFP_6 feature: 1335108269: [*]N[c](:[cH]:[*]):[c]([*]):[*]
13. Unknown ECFP_6 feature: 1203316083: [*][c]1:[*]:[*]:[c]([*]):o:1
14. Unknown ECFP_6 feature: 1093109320: [*]S[c]1:o:[*]:[*]:n:1
15. Unknown ECFP_6 feature: 911256832: [*][c]1:[*]:[*]:n:n:1
16. Unknown ECFP_6 feature: 1427820655: [*]CS[c](:[*]):[*]
17. Unknown ECFP_6 feature: -178525456: [*]:[cH]:[c](:[cH]:[*]):[c](:[*]):[*]
18. Unknown ECFP_6 feature: -176846085: [*]C([*])[c](:[cH]:[*]):[cH]:[*]
19. Unknown ECFP_6 feature: 1307307440: [*]:[c](:[*])OC
20. Unknown ECFP_6 feature: -1841325949: [*]:[c](:[*])C(C)[c](:[*]):[*]
21. Unknown ECFP_6 feature: 1092541557: [*]C([*])[c]1:o:[*]:[*]:n:1

22. Unknown ECFP_6 feature: -1956535100: [*][c](:[*]):[c](:[cH]:[*])[N+](=[*])[*]
23. Unknown ECFP_6 feature: 1997021792: [*]:[cH]:[cH]:[cH]:[*]
24. Unknown ECFP_6 feature: -215026467: [*]:[c](:[*])[N+](=O)[O-]
25. Unknown ECFP_6 feature: 2104376220: [*][N+](=O)[*]
26. Unknown ECFP_6 feature: -659271057: [*][N+](=[*])[O-]

Feature Contribution

Top features for positive contribution

Fingerprint	Bit/Smiles	Feature Structure	Score
ECFP_6	1559650422	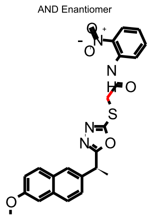 [*]C[*]	0.129
ECFP_6	-176455838	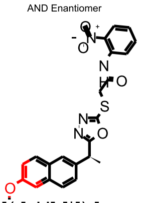 [*]O[c](:[cH]:[*]):[cH]:[*]	0.106
FCFP_6	3	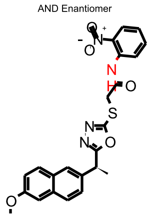 [*]N[*]	0.092

Top Features for negative contribution

Fingerprint	Bit/Smiles	Feature Structure	Score

FCFP_6	991735244	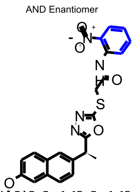 [*][c]1:[*]:[cH]:[cH]:[cH]:[cH]:1	-0.134
ECFP_6	1564392544	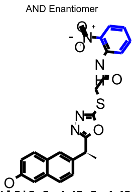 [*][c]1:[*]:[cH]:[cH]:[cH]:[cH]:1	-0.133
FCFP_6	1	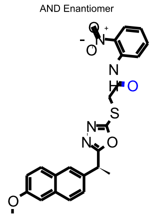 [*]=O	-0.102

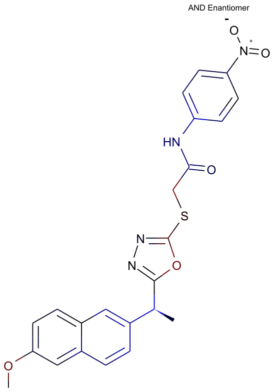

$C_{23}H_{20}N_4O_5S$

Molecular Weight: 464.4937

ALogP: 4.132

Rotatable Bonds: 8

Acceptors: 7

Donors: 1

Model Prediction

Prediction: 0.014

Unit: g/kg_body_weight

Mahalanobis Distance: 37.958

Mahalanobis Distance p-value: 9.66e-039

Mahalanobis Distance: The Mahalanobis distance (MD) is a generalization of the Euclidean distance that accounts for correlations among the X properties. It is calculated as the distance to the center of the training data. The larger the MD, the less trustworthy the prediction.
Mahalanobis Distance p-value: The p-value gives the fraction of training data with an MD greater than or equal to the one for the given sample, assuming normally distributed data. The smaller the p-value, the less trustworthy the prediciton. For highly non-normal X properties (e.g., fingerprints), the MD p-value is wildly inaccurate.

Structural Similar Compounds

Name	C.I.PIGMENT RED 23	C.I. ACID RED 14	C.I. ACID ORANGE 3
Structure	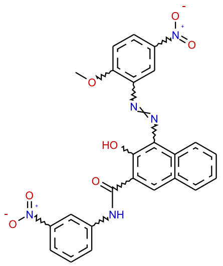	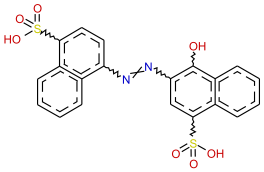	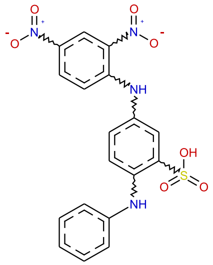
Actual Endpoint (-log C)	2.28997	2.8654	3.20573
Predicted Endpoint (-log C)	3.52921	3.29295	3.55956
Distance	0.628	0.793	0.810
Reference	NTP 411 146	NTP REPORT # 220	NTP REPORT # 335

Model Applicability

Unknown features are fingerprint features in the query molecule, but not found in the training set.

1. All properties and OPS components are within expected ranges.
2. Unknown FCFP_2 feature: 5: [*][O-]
3. Unknown FCFP_2 feature: -828984032: [*][c](:[*]):[c]([N+](=[*])[*]):c:[*]
4. Unknown FCFP_2 feature: -1338588315: [*]:[c](:[*])[N+](=O)[O-]
5. Unknown FCFP_2 feature: 1872392852: [*][N+](=O)[*]
6. Unknown FCFP_2 feature: 260476081: [*][N+](=[*])[O-]
7. Unknown ECFP_6 feature: -830332112: [*]S[*]
8. Unknown ECFP_6 feature: 1043790491: [*][N+](=[*])[*]
9. Unknown ECFP_6 feature: 781519895: [*][O-]
10. Unknown ECFP_6 feature: -955816473: [*]SCC(=[*])[*]
11. Unknown ECFP_6 feature: 1731843802: [*]CC(=O)N[*]
12. Unknown ECFP_6 feature: -177077903: [*]N[c](:[cH]:[*]):[cH]:[*]
13. Unknown ECFP_6 feature: 1203316083: [*][c]1:[*]:[*]:[c]([*]):o:1
14. Unknown ECFP_6 feature: 1093109320: [*]S[c]1:o:[*]:[*]:n:1
15. Unknown ECFP_6 feature: 911256832: [*][c]1:[*]:[*]:n:n:1
16. Unknown ECFP_6 feature: 1427820655: [*]CS[c](:[*]):[*]
17. Unknown ECFP_6 feature: -178525456: [*]:[cH]:[c](:[cH]:[*]):[c](:[*]):[*]
18. Unknown ECFP_6 feature: -176846085: [*]C([*])[c](:[cH]:[*]):[cH]:[*]
19. Unknown ECFP_6 feature: 1307307440: [*]:[c](:[*])OC
20. Unknown ECFP_6 feature: -1841325949: [*]:[c](:[*])C(C)[c](:[*]):[*]
21. Unknown ECFP_6 feature: 1092541557: [*]C([*])[c]1:o:[*]:[*]:n:1

22. Unknown ECFP_6 feature: -179073144: [*][N+](=[*])[c](:[cH]:[*]):[cH]:[*]
23. Unknown ECFP_6 feature: -215026467: [*]:[c](:[*])[N+](=O)[O-]
24. Unknown ECFP_6 feature: 2104376220: [*][N+](=O)[*]
25. Unknown ECFP_6 feature: -659271057: [*][N+](=[*])[O-]

Feature Contribution

Top features for positive contribution

Fingerprint	Bit/Smiles	Feature Structure	Score
ECFP_6	1559650422	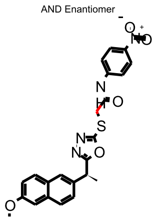 [*]C[*]	0.129
ECFP_6	-176455838	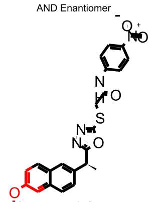 [*]O[c](:[cH]:[*]):[cH]:[*]	0.106
FCFP_6	3	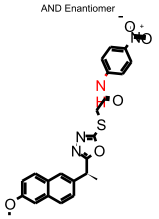 [*]N[*]	0.092

Top Features for negative contribution

Fingerprint	Bit/Smiles	Feature Structure	Score

FCFP_6	1	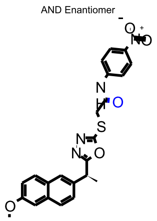 [*]=O	-0.102
FCFP_6	-453677277	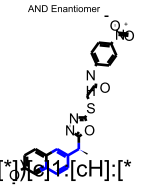 [*]C([*])[c]1:[cH]:[*]:[c](:[*]):[cH]:[cH]:1	-0.091
FCFP_6	136597326	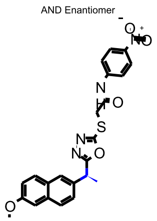 [*]C([*])C	-0.081

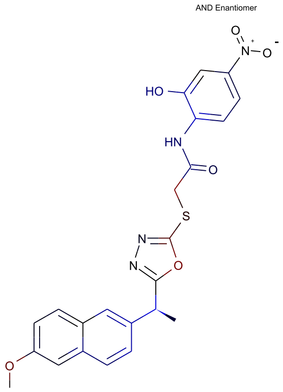

$C_{23}H_{20}N_4O_6S$

Molecular Weight: 480.4931

ALogP: 3.89

Rotatable Bonds: 8

Acceptors: 8

Donors: 2

Model Prediction

Prediction: 0.032

Unit: g/kg_body_weight

Mahalanobis Distance: 39.932

Mahalanobis Distance p-value: 4.72e-042

Mahalanobis Distance: The Mahalanobis distance (MD) is a generalization of the Euclidean distance that accounts for correlations among the X properties. It is calculated as the distance to the center of the training data. The larger the MD, the less trustworthy the prediction.
Mahalanobis Distance p-value: The p-value gives the fraction of training data with an MD greater than or equal to the one for the given sample, assuming normally distributed data. The smaller the p-value, the less trustworthy the prediciton. For highly non-normal X properties (e.g., fingerprints), the MD p-value is wildly inaccurate.

Structural Similar Compounds

Name	C.I.PIGMENT RED 23	C.I. ACID RED 14	C.I. ACID ORANGE 3
Structure	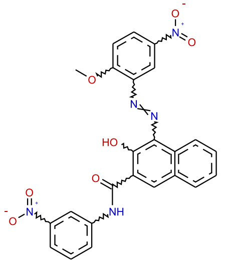	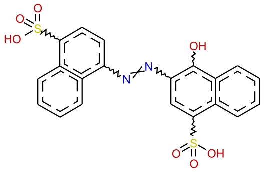	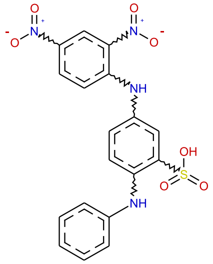
Actual Endpoint (-log C)	2.28997	2.8654	3.20573
Predicted Endpoint (-log C)	3.52921	3.29295	3.55956
Distance	0.505	0.657	0.671
Reference	NTP 411 146	NTP REPORT # 220	NTP REPORT # 335

Model Applicability

Unknown features are fingerprint features in the query molecule, but not found in the training set.

1. All properties and OPS components are within expected ranges.
2. Unknown FCFP_2 feature: 5: [*][O-]
3. Unknown FCFP_2 feature: -828984032: [*][c](:[*]):[c]([N+](=[*])[*]):c:[*]
4. Unknown FCFP_2 feature: -1338588315: [*]:[c](:[*])[N+](=O)[O-]
5. Unknown FCFP_2 feature: 1872392852: [*][N+](=O)[*]
6. Unknown FCFP_2 feature: 260476081: [*][N+](=[*])[O-]
7. Unknown ECFP_6 feature: -830332112: [*]S[*]
8. Unknown ECFP_6 feature: 1043790491: [*][N+](=[*])[*]
9. Unknown ECFP_6 feature: 781519895: [*][O-]
10. Unknown ECFP_6 feature: -955816473: [*]SCC(=[*])[*]
11. Unknown ECFP_6 feature: 1731843802: [*]CC(=O)N[*]
12. Unknown ECFP_6 feature: 1335108269: [*]N[c](:[cH]:[*]):[c]([*]):[*]
13. Unknown ECFP_6 feature: 1203316083: [*][c]1:[*]:[*]:[c]([*]):o:1
14. Unknown ECFP_6 feature: 1093109320: [*]S[c]1:o:[*]:[*]:n:1
15. Unknown ECFP_6 feature: 911256832: [*][c]1:[*]:[*]:n:n:1
16. Unknown ECFP_6 feature: 1427820655: [*]CS[c](:[*]):[*]
17. Unknown ECFP_6 feature: -178525456: [*]:[cH]:[c](:[cH]:[*]):[c](:[*]):[*]
18. Unknown ECFP_6 feature: -176846085: [*]C([*])[c](:[cH]:[*]):[cH]:[*]
19. Unknown ECFP_6 feature: 1307307440: [*]:[c](:[*])OC
20. Unknown ECFP_6 feature: -1841325949: [*]:[c](:[*])C(C)[c](:[*]):[*]
21. Unknown ECFP_6 feature: 1092541557: [*]C([*])[c]1:o:[*]:[*]:n:1

22. Unknown ECFP_6 feature: -179073144: [*][N+](=[*])[c](:[cH]:[*]):[cH]:[*]
23. Unknown ECFP_6 feature: 2019062761: [*]:[c](:[*])O
24. Unknown ECFP_6 feature: -215026467: [*]:[c](:[*])[N+](=O)[O-]
25. Unknown ECFP_6 feature: 2104376220: [*][N+](=O)[*]
26. Unknown ECFP_6 feature: -659271057: [*][N+](=[*])[O-]

Feature Contribution

Top features for positive contribution			
**Fingerprint**	**Bit/Smiles**	**Feature Structure**	**Score**
ECFP_6	1559650422	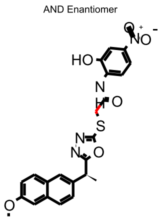 [*]C[*]	0.129
ECFP_6	-176455838	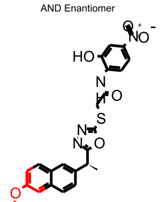 [*]O[c](:[cH]:[*]):[cH]:[*]	0.106
FCFP_6	3	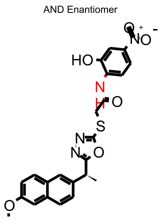 [*]N[*]	0.092

Top Features for negative contribution			
**Fingerprint**	**Bit/Smiles**	**Feature Structure**	**Score**

FCFP_6	1	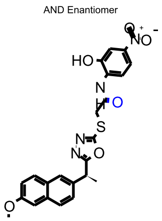 [*]=O	-0.102
FCFP_6	-453677277	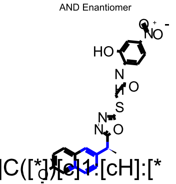 [*]C([*])[c]1:[cH]:[*<br>]:[c](:[*]):[cH]:[cH<br>]:1	-0.091
FCFP_6	136597326	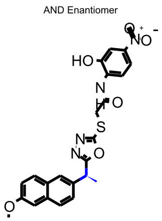 [*]C([*])C	-0.081

Sorafenib

TOPKAT_Chronic_LOAEL

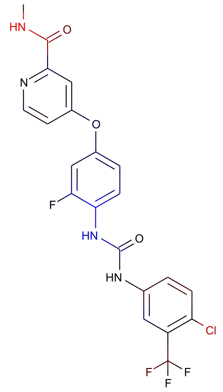

$C_{21}H_{15}ClF_4N_4O_3$

Molecular Weight: 482.81541

ALogP: 4.381

Rotatable Bonds: 6

Acceptors: 4

Donors: 3

Model Prediction

Prediction: 0.004

Unit: g/kg_body_weight

Mahalanobis Distance: 30.058

Mahalanobis Distance p-value: 9.03e-025

Mahalanobis Distance: The Mahalanobis distance (MD) is a generalization of the Euclidean distance that accounts for correlations among the X properties. It is calculated as the distance to the center of the training data. The larger the MD, the less trustworthy the prediction.
Mahalanobis Distance p-value: The p-value gives the fraction of training data with an MD greater than or equal to the one for the given sample, assuming normally distributed data. The smaller the p-value, the less trustworthy the prediciton. For highly non-normal X properties (e.g., fingerprints), the MD p-value is wildly inaccurate.

Structural Similar Compounds

Name	GLYBURIDE	D & C RED 9	FLUVALINATE
Structure	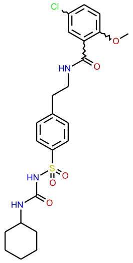	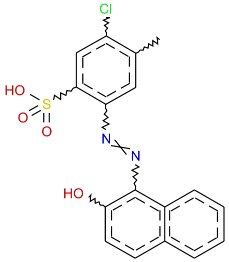	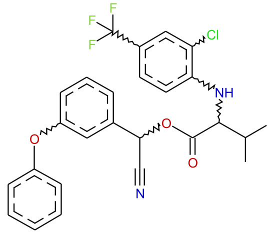
Actual Endpoint (-log C)	4.21661	3.87715	5.30356
Predicted Endpoint (-log C)	4.21035	3.6546	4.89944
Distance	0.635	0.738	0.755
Reference	UPJ-26452	NTP REPORT # 225	EPA COVER SHEET 0281;880630;(1)

Model Applicability

Unknown features are fingerprint features in the query molecule, but not found in the training set.

1. All properties and OPS components are within expected ranges.
2. Unknown ECFP_6 feature: -1046436026: [*]F
3. Unknown ECFP_6 feature: 1305253718: [*]:[c](:[*])O[c](:[*]):[*]
4. Unknown ECFP_6 feature: 1413420509: [*]C(=[*])[c](:[cH]:[*]):n:[*]
5. Unknown ECFP_6 feature: -677309799: [*][c](:[*]):n:[cH]:[*]
6. Unknown ECFP_6 feature: 1996163143: [*]:[cH]:[cH]:n:[*]
7. Unknown ECFP_6 feature: 1430169877: [*]NC(=O)[c](:[*]):[*]
8. Unknown ECFP_6 feature: 1338334141: [*]C(=[*])NC
9. Unknown ECFP_6 feature: 864287155: [*]NC
10. Unknown ECFP_6 feature: 1335108269: [*]N[c](:[cH]:[*]):[c]([*]):[*]
11. Unknown ECFP_6 feature: -1311285389: [*][c](:[*]):[c](F):[cH]:[*]
12. Unknown ECFP_6 feature: -649580166: [*]NC(=O)N[*]
13. Unknown ECFP_6 feature: -177077903: [*]N[c](:[cH]:[*]):[cH]:[*]
14. Unknown ECFP_6 feature: 1336678434: [*][c](:[*]):[c](:[cH]:[*])C([*])([*])[*]
15. Unknown ECFP_6 feature: 99947387: [*]:[c](:[*])Cl
16. Unknown ECFP_6 feature: 220735655: [*]:[c](:[*])F
17. Unknown ECFP_6 feature: -1952889961: [*]:[c](:[*])C(F)(F)F
18. Unknown ECFP_6 feature: 226796801: [*]C([*])([*])F

Feature Contribution

Top features for positive contribution

Fingerprint	Bit/Smiles	Feature Structure	Score
ECFP_6	-176455838	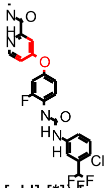 [*]O[c](:[cH]:[*]):[cH]:[*]	0.106
FCFP_6	32	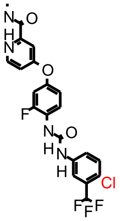 [*]Cl	0.101
FCFP_6	3	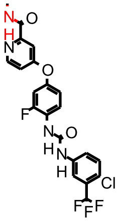 [*]N[*]	0.092

Top Features for negative contribution

Fingerprint	Bit/Smiles	Feature Structure	Score
FCFP_6	1	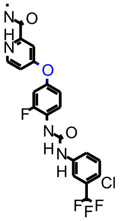 [*]=O	-0.102

ECFP_6	-1236483485	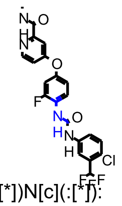 [*]C(=[*])N[c](:[*]):[*]	-0.075
FCFP_6	203677720	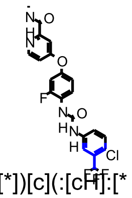 [*]C([*])[c](:[cH]:[*]):[cH]:[*]	-0.071

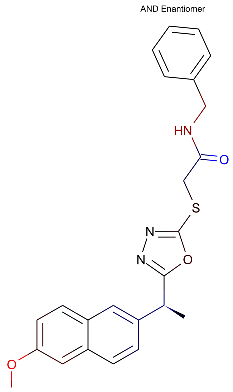

$C_{24}H_{23}N_3O_3S$

Molecular Weight: 433.52272

ALogP: 4.245

Rotatable Bonds: 8

Acceptors: 5

Donors: 1

Model Prediction

Prediction: 0.042

Unit: g/kg_body_weight

Mahalanobis Distance: 10.412

Mahalanobis Distance p-value: 9.29e-006

Mahalanobis Distance: The Mahalanobis distance (MD) is a generalization of the Euclidean distance that accounts for correlations among the X properties. It is calculated as the distance to the center of the training data. The larger the MD, the less trustworthy the prediction.
Mahalanobis Distance p-value: The p-value gives the fraction of training data with an MD greater than or equal to the one for the given sample, assuming normally distributed data. The smaller the p-value, the less trustworthy the prediciton. For highly non-normal X properties (e.g., fingerprints), the MD p-value is wildly inaccurate.

Structural Similar Compounds

Name	C.I.PIGMENT RED 3	PHENOLPHTHALEIN	ROTENONE
Structure	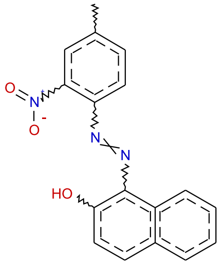	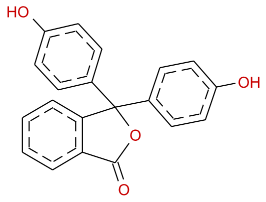	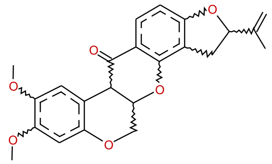
Actual Endpoint (-log C)	2.65635	2.20184	5.06769
Predicted Endpoint (-log C)	2.97957	2.8857	4.11907
Distance	0.776	0.889	0.891
Reference	NCI/NTP TR-407	NCI/NTP TR-465	NCI/NTP TR-320

Model Applicability

Unknown features are fingerprint features in the query molecule, but not found in the training set.

1. OPS PC9 out of range. Value: 4.1338. Training min, max, SD, explained variance: -2.8548, 3.3954, 1.263, 0.0360.
2. Unknown FCFP_2 feature: -1410079687: [*]S[c]1:o:[*]:[*]:n:1

Feature Contribution

Top features for positive contribution

Fingerprint	Bit/Smiles	Feature Structure	Score
FCFP_2	136627117	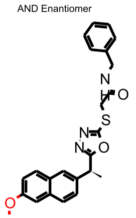 [*]OC	0.173

FCFP_2	-885550502	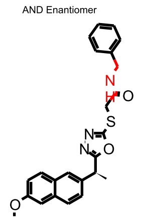 [*]CNC(=[*])[*]	0.115
FCFP_2	1036089772	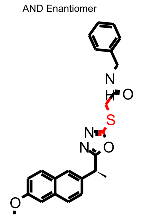 [*]CS[c](:[*]):[*]	0.075

Top Features for negative contribution

Fingerprint	Bit/Smiles	Feature Structure	Score
FCFP_2	1872154524	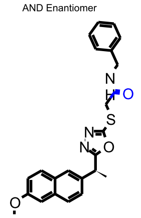 [*]C(=O)[*]	-0.105
FCFP_2	203677720	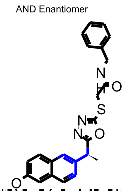 [*]C([*])[c](:[cH]:[*]):[cH]:[*]	-0.083
FCFP_2	1	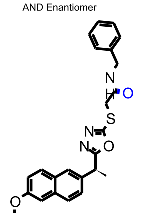 [*]=O	-0.080

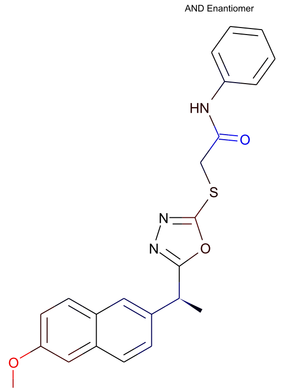

$C_{23}H_{21}N_3O_3S$

Molecular Weight: 419.49614

ALogP: 4.238

Rotatable Bonds: 7

Acceptors: 5

Donors: 1

Model Prediction

Prediction: 0.054

Unit: g/kg_body_weight

Mahalanobis Distance: 10.083

Mahalanobis Distance p-value: 3.26e-005

Mahalanobis Distance: The Mahalanobis distance (MD) is a generalization of the Euclidean distance that accounts for correlations among the X properties. It is calculated as the distance to the center of the training data. The larger the MD, the less trustworthy the prediction.
Mahalanobis Distance p-value: The p-value gives the fraction of training data with an MD greater than or equal to the one for the given sample, assuming normally distributed data. The smaller the p-value, the less trustworthy the prediciton. For highly non-normal X properties (e.g., fingerprints), the MD p-value is wildly inaccurate.

Structural Similar Compounds

Name	C.I.PIGMENT RED 3	PHENOLPHTHALEIN	DISPERSE YELLOW 3
Structure	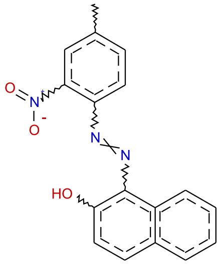	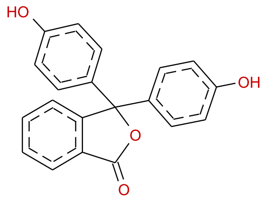	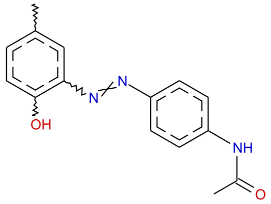
Actual Endpoint (-log C)	2.65635	2.20184	2.77703
Predicted Endpoint (-log C)	2.97957	2.8857	2.80195
Distance	0.730	0.844	0.860
Reference	NCI/NTP TR-407	NCI/NTP TR-465	NCI/NTP TR-222

Model Applicability

Unknown features are fingerprint features in the query molecule, but not found in the training set.

1. OPS PC9 out of range. Value: 4.8445. Training min, max, SD, explained variance: -2.8548, 3.3954, 1.263, 0.0360.
2. Unknown FCFP_2 feature: -1410079687: [*]S[c]1:o:[*]:[*]:n:1

Feature Contribution

Top features for positive contribution

Fingerprint	Bit/Smiles	Feature Structure	Score
FCFP_2	136627117	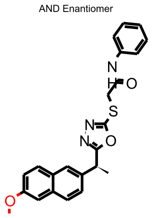 [*]OC	0.173

FCFP_2	1036089772	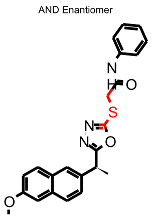 [*]CS[c](:[*]):[*]	0.075
FCFP_2	3	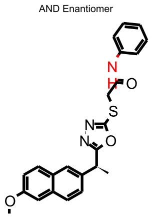 [*]N[*]	0.074

Top Features for negative contribution

**Fingerprint**	**Bit/Smiles**	**Feature Structure**	**Score**
FCFP_2	1872154524	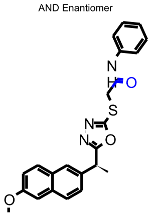 [*]C(=O)[*]	-0.105
FCFP_2	203677720	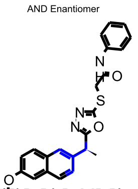 [*]C([*])[c](:[cH]:[*])):[cH]:[*]	-0.083
FCFP_2	1	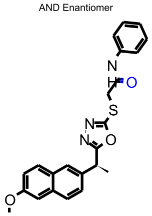 [*]=O	-0.080

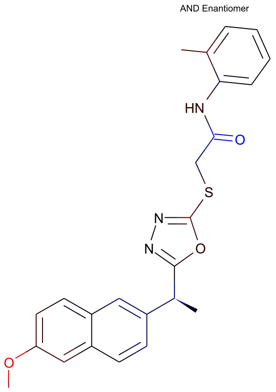

$C_{24}H_{23}N_3O_3S$

Molecular Weight: 433.52272

ALogP: 4.724

Rotatable Bonds: 7

Acceptors: 5

Donors: 1

Model Prediction

Prediction: 0.045

Unit: g/kg_body_weight

Mahalanobis Distance: 10.806

Mahalanobis Distance p-value: 1.95e-006

Mahalanobis Distance: The Mahalanobis distance (MD) is a generalization of the Euclidean distance that accounts for correlations among the X properties. It is calculated as the distance to the center of the training data. The larger the MD, the less trustworthy the prediction.

Mahalanobis Distance p-value: The p-value gives the fraction of training data with an MD greater than or equal to the one for the given sample, assuming normally distributed data. The smaller the p-value, the less trustworthy the prediciton. For highly non-normal X properties (e.g., fingerprints), the MD p-value is wildly inaccurate.

Structural Similar Compounds

Name	C.I.PIGMENT RED 3	DISPERSE YELLOW 3	PHENOLPHTHALEIN
Structure	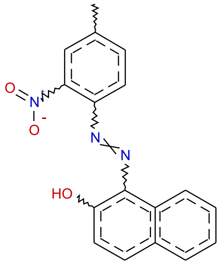	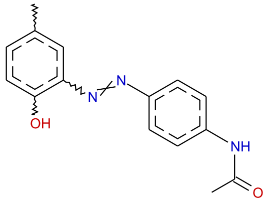	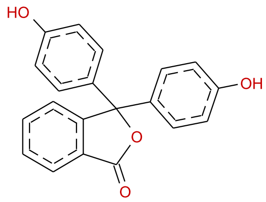
Actual Endpoint (-log C)	2.65635	2.77703	2.20184
Predicted Endpoint (-log C)	2.97957	2.80195	2.8857
Distance	0.716	0.858	0.861
Reference	NCI/NTP TR-407	NCI/NTP TR-222	NCI/NTP TR-465

Model Applicability

Unknown features are fingerprint features in the query molecule, but not found in the training set.

1. OPS PC9 out of range. Value: 4.0612. Training min, max, SD, explained variance: -2.8548, 3.3954, 1.263, 0.0360.
2. Unknown FCFP_2 feature: -1410079687: [*]S[c]1:o:[*]:[*]:n:1

Feature Contribution

Top features for positive contribution

Fingerprint	Bit/Smiles	Feature Structure	Score
FCFP_2	136627117	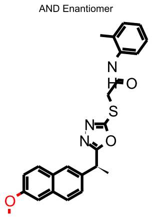 [*]OC	0.173

FCFP_2	1036089772	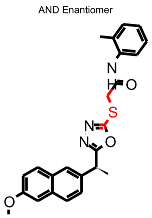 [*]CS[c](:[*]):[*]	0.075
FCFP_2	3	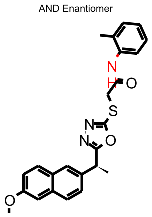 [*]N[*]	0.074

Top Features for negative contribution

**Fingerprint**	**Bit/Smiles**	**Feature Structure**	**Score**
FCFP_2	1872154524	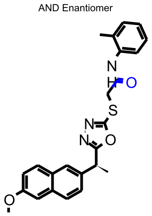 [*]C(=O)[*]	-0.105
FCFP_2	203677720	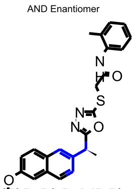 [*]C([*])[c](:[cH]:[*]):[cH]:[*]	-0.083
FCFP_2	1	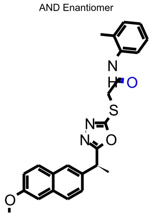 [*]=O	-0.080

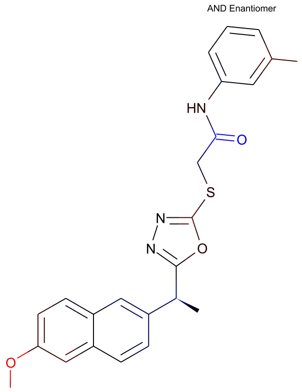

$C_{24}H_{23}N_3O_3S$
Molecular Weight: 433.52272
ALogP: 4.724
Rotatable Bonds: 7
Acceptors: 5
Donors: 1

Model Prediction

Prediction: 0.045
Unit: g/kg_body_weight
Mahalanobis Distance: 10.806
Mahalanobis Distance p-value: 1.95e-006

Mahalanobis Distance: The Mahalanobis distance (MD) is a generalization of the Euclidean distance that accounts for correlations among the X properties. It is calculated as the distance to the center of the training data. The larger the MD, the less trustworthy the prediction.
Mahalanobis Distance p-value: The p-value gives the fraction of training data with an MD greater than or equal to the one for the given sample, assuming normally distributed data. The smaller the p-value, the less trustworthy the prediciton. For highly non-normal X properties (e.g., fingerprints), the MD p-value is wildly inaccurate.

Structural Similar Compounds

Name	C.I.PIGMENT RED 3	DISPERSE YELLOW 3	PHENOLPHTHALEIN
Structure	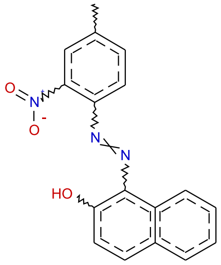	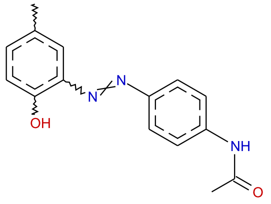	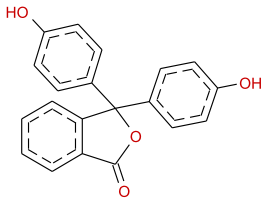
Actual Endpoint (-log C)	2.65635	2.77703	2.20184
Predicted Endpoint (-log C)	2.97957	2.80195	2.8857
Distance	0.716	0.858	0.861
Reference	NCI/NTP TR-407	NCI/NTP TR-222	NCI/NTP TR-465

Model Applicability

Unknown features are fingerprint features in the query molecule, but not found in the training set.

1. OPS PC9 out of range. Value: 4.0612. Training min, max, SD, explained variance: -2.8548, 3.3954, 1.263, 0.0360.
2. Unknown FCFP_2 feature: -1410079687: [*]S[c]1:o:[*]:[*]:n:1

Feature Contribution

Top features for positive contribution

Fingerprint	Bit/Smiles	Feature Structure	Score
FCFP_2	136627117	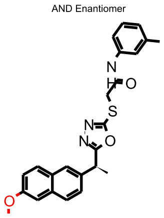 [*]OC	0.173

FCFP_2	1036089772	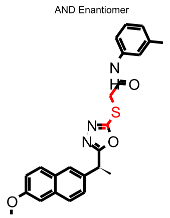 [*]CS[c](:[*]):[*]	0.075
FCFP_2	3	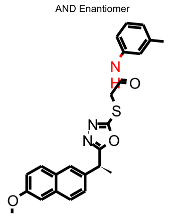 [*]N[*]	0.074
**Top Features for negative contribution**			
**Fingerprint**	**Bit/Smiles**	**Feature Structure**	**Score**
FCFP_2	1872154524	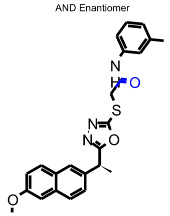 [*]C(=O)[*]	-0.105
FCFP_2	203677720	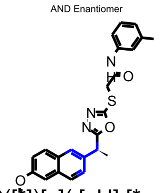 [*]C([*])[c](:[cH]:[*]):[cH]:[*]	-0.083
FCFP_2	1	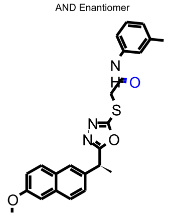 [*]=O	-0.080

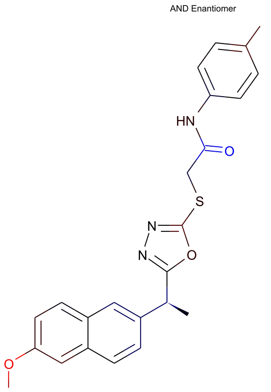

$C_{24}H_{23}N_3O_3S$
Molecular Weight: 433.52272
ALogP: 4.724
Rotatable Bonds: 7
Acceptors: 5
Donors: 1

Structural Similar Compounds

Name	C.I.PIGMENT RED 3	DISPERSE YELLOW 3	PHENOLPHTHALEIN
Structure	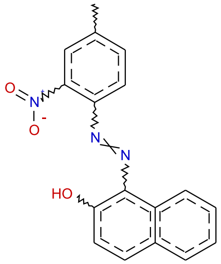	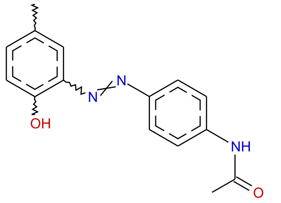	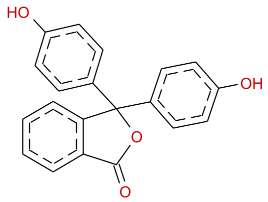
Actual Endpoint (-log C)	2.65635	2.77703	2.20184
Predicted Endpoint (-log C)	2.97957	2.80195	2.8857
Distance	0.716	0.858	0.861
Reference	NCI/NTP TR-407	NCI/NTP TR-222	NCI/NTP TR-465

Model Applicability

Unknown features are fingerprint features in the query molecule, but not found in the training set.

1. OPS PC9 out of range. Value: 4.0612. Training min, max, SD, explained variance: -2.8548, 3.3954, 1.263, 0.0360.
2. Unknown FCFP_2 feature: -1410079687: [*]S[c]1:o:[*]:[*]:n:1

Model Prediction

Prediction: 0.045
Unit: g/kg_body_weight
Mahalanobis Distance: 10.806
Mahalanobis Distance p-value: 1.95e-006

Mahalanobis Distance: The Mahalanobis distance (MD) is a generalization of the Euclidean distance that accounts for correlations among the X properties. It is calculated as the distance to the center of the training data. The larger the MD, the less trustworthy the prediction.
Mahalanobis Distance p-value: The p-value gives the fraction of training data with an MD greater than or equal to the one for the given sample, assuming normally distributed data. The smaller the p-value, the less trustworthy the prediciton. For highly non-normal X properties (e.g., fingerprints), the MD p-value is wildly inaccurate.

Feature Contribution

Top features for positive contribution			
**Fingerprint**	**Bit/Smiles**	**Feature Structure**	**Score**
FCFP_2	136627117	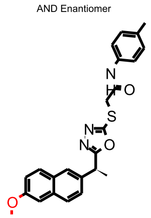 [*]OC	0.173

FCFP_2	1036089772	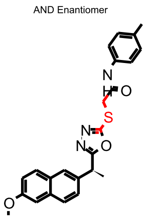 [*]CS[c](:[*]):[*]	0.075
FCFP_2	3	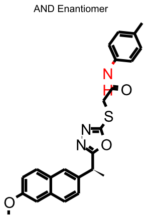 [*]N[*]	0.074

Top Features for negative contribution

Fingerprint	Bit/Smiles	Feature Structure	Score
FCFP_2	1872154524	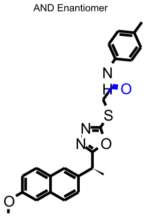 [*]C(=O)[*]	-0.105
FCFP_2	203677720	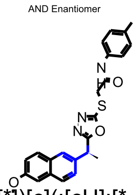 [*]C([*])[c](:[cH]:[*])):[cH]:[*]	-0.083
FCFP_2	1	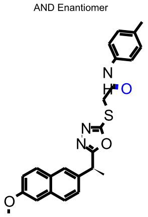 [*]=O	-0.080

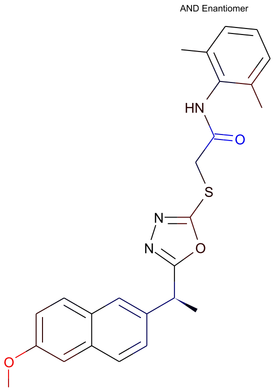

$C_{25}H_{25}N_3O_3S$
Molecular Weight: 447.5493
ALogP: 5.21
Rotatable Bonds: 7
Acceptors: 5
Donors: 1

Model Prediction

Prediction: 0.043
Unit: g/kg_body_weight
Mahalanobis Distance: 10.760
Mahalanobis Distance p-value: 2.34e-006

Mahalanobis Distance: The Mahalanobis distance (MD) is a generalization of the Euclidean distance that accounts for correlations among the X properties. It is calculated as the distance to the center of the training data. The larger the MD, the less trustworthy the prediction.
Mahalanobis Distance p-value: The p-value gives the fraction of training data with an MD greater than or equal to the one for the given sample, assuming normally distributed data. The smaller the p-value, the less trustworthy the prediciton. For highly non-normal X properties (e.g., fingerprints), the MD p-value is wildly inaccurate.

Structural Similar Compounds

Name	C.I.PIGMENT RED 3	C.I.PIGMENT RED 23	DISPERSE YELLOW 3
Structure	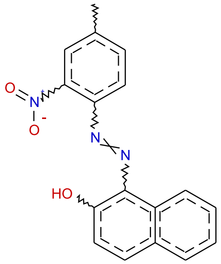	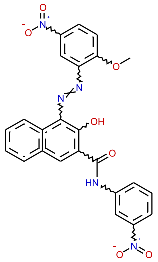	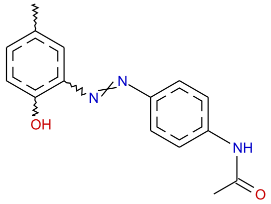
Actual Endpoint (-log C)	2.65635	2.30052	2.77703
Predicted Endpoint (-log C)	2.97957	3.55333	2.80195
Distance	0.727	0.864	0.878
Reference	NCI/NTP TR-407	NCI/NTP TR-411	NCI/NTP TR-222

Model Applicability

Unknown features are fingerprint features in the query molecule, but not found in the training set.

1. OPS PC9 out of range. Value: 4.0942. Training min, max, SD, explained variance: -2.8548, 3.3954, 1.263, 0.0360.
2. Unknown FCFP_2 feature: -1410079687: [*]S[c]1:o:[*]:[*]:n:1

Feature Contribution

Top features for positive contribution

Fingerprint	Bit/Smiles	Feature Structure	Score
FCFP_2	136627117	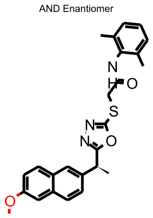 [*]OC	0.173

FCFP_2	1036089772	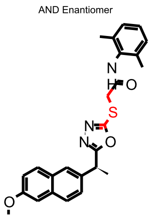 [*]CS[c](:[*]):[*]	0.075
FCFP_2	3	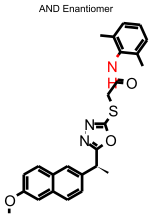 [*]N[*]	0.074

Top Features for negative contribution

Fingerprint	Bit/Smiles	Feature Structure	Score
FCFP_2	1872154524	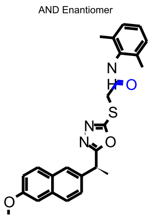 [*]C(=O)[*]	-0.105
FCFP_2	203677720	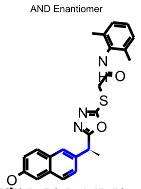 [*]C([*])[c](:[cH]:[*]):[cH]:[*]	-0.083
FCFP_2	1	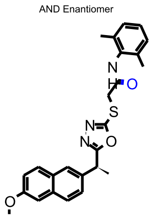 [*]=O	-0.080

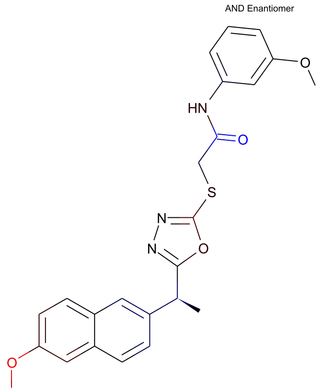

$C_{24}H_{23}N_3O_4S$
Molecular Weight: 449.52212
ALogP: 4.221
Rotatable Bonds: 8
Acceptors: 6
Donors: 1

Model Prediction

Prediction: 0.052
Unit: g/kg_body_weight
Mahalanobis Distance: 9.698
Mahalanobis Distance p-value: 0.000134

Mahalanobis Distance: The Mahalanobis distance (MD) is a generalization of the Euclidean distance that accounts for correlations among the X properties. It is calculated as the distance to the center of the training data. The larger the MD, the less trustworthy the prediction.
Mahalanobis Distance p-value: The p-value gives the fraction of training data with an MD greater than or equal to the one for the given sample, assuming normally distributed data. The smaller the p-value, the less trustworthy the prediciton. For highly non-normal X properties (e.g., fingerprints), the MD p-value is wildly inaccurate.

Structural Similar Compounds

Name	C.I.PIGMENT RED 23	C.I.PIGMENT RED 3	SALICYLAZOSULFAPYRIDINE
Structure	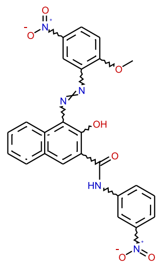	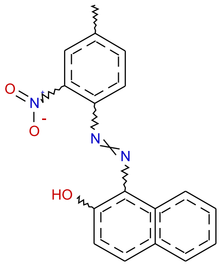	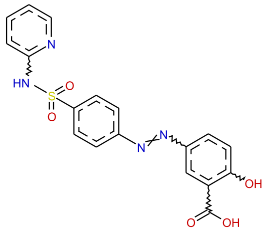
Actual Endpoint (-log C)	2.30052	2.65635	3.375
Predicted Endpoint (-log C)	3.55333	2.97957	2.80292
Distance	0.797	0.804	0.830
Reference	NCI/NTP TR-411	NCI/NTP TR-407	NCI/NTP TR-457

Model Applicability

Unknown features are fingerprint features in the query molecule, but not found in the training set.

1. OPS PC9 out of range. Value: 4.9278. Training min, max, SD, explained variance: -2.8548, 3.3954, 1.263, 0.0360.
2. Unknown FCFP_2 feature: -1410079687: [*]S[c]1:o:[*]:[*]:n:1

Feature Contribution

Top features for positive contribution			
**Fingerprint**	**Bit/Smiles**	**Feature Structure**	**Score**
FCFP_2	136627117	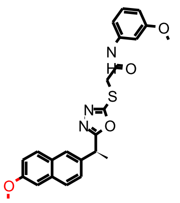 AND Enantiomer [*]OC	0.173

FCFP_2	1036089772	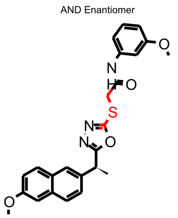 [*]CS[c](:[*]):[*]	0.075
FCFP_2	3	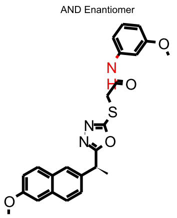 [*]N[*]	0.074

Top Features for negative contribution

Fingerprint	Bit/Smiles	Feature Structure	Score
FCFP_2	1872154524	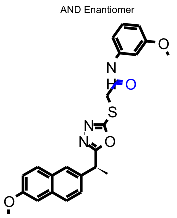 [*]C(=O)[*]	-0.105
FCFP_2	203677720	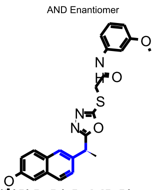 [*]C([*])[c](:[cH]:[*])):[cH]:[*]	-0.083
FCFP_2	1	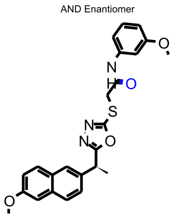 [*]=O	-0.080

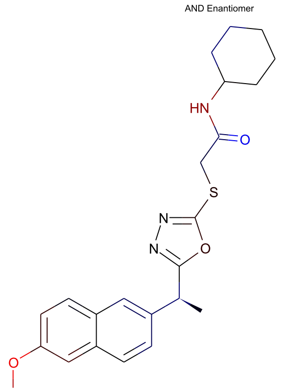

$C_{23}H_{27}N_3O_3S$
Molecular Weight: 425.54378
ALogP: 4.52
Rotatable Bonds: 7
Acceptors: 5
Donors: 1

Structural Similar Compounds

Name	C.I.PIGMENT RED 3	ROTENONE	3,3'-DIMETHOXYBENZIDINE-4,4'-DIISOCYANATE
Structure	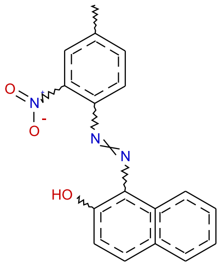	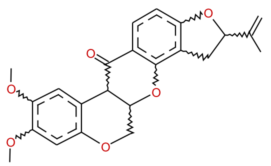	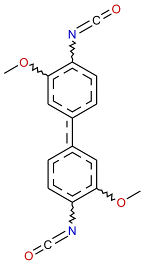
Actual Endpoint (-log C)	2.65635	5.06769	2.17504
Predicted Endpoint (-log C)	2.97957	4.11907	3.78717
Distance	0.670	0.742	0.759
Reference	NCI/NTP TR-407	NCI/NTP TR-320	NCI/NTP TR-128

Model Prediction

Prediction: 0.042
Unit: g/kg_body_weight
Mahalanobis Distance: 9.375
Mahalanobis Distance p-value: 0.000412

Mahalanobis Distance: The Mahalanobis distance (MD) is a generalization of the Euclidean distance that accounts for correlations among the X properties. It is calculated as the distance to the center of the training data. The larger the MD, the less trustworthy the prediction.
Mahalanobis Distance p-value: The p-value gives the fraction of training data with an MD greater than or equal to the one for the given sample, assuming normally distributed data. The smaller the p-value, the less trustworthy the prediciton. For highly non-normal X properties (e.g., fingerprints), the MD p-value is wildly inaccurate.

Model Applicability

Unknown features are fingerprint features in the query molecule, but not found in the training set.

1. OPS PC9 out of range. Value: 4.3841. Training min, max, SD, explained variance: -2.8548, 3.3954, 1.263, 0.0360.
2. Unknown FCFP_2 feature: -1410079687: [*]S[c]1:o:[*]:[*]:n:1

Feature Contribution

Top features for positive contribution

Fingerprint	Bit/Smiles	Feature Structure	Score
FCFP_2	136627117	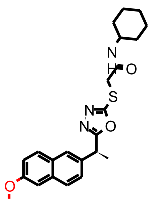 AND Enantiomer [*]OC	0.173

FCFP_2	-885550502	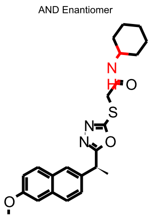 [*]CNC(=[*])[*]	0.115
FCFP_2	1036089772	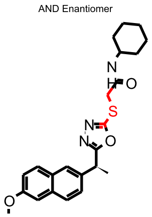 [*]CS[c](:[*]):[*]	0.075

Top Features for negative contribution

Fingerprint	Bit/Smiles	Feature Structure	Score
FCFP_2	-1272798659	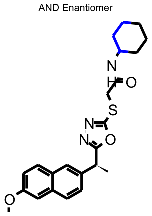 [*]CCC([*])[*]	-0.111
FCFP_2	1872154524	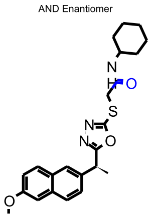 [*]C(=O)[*]	-0.105
FCFP_2	203677720	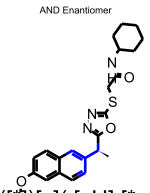 [*]C([*])[c](:[cH]:[*]):[cH]:[*]	-0.083

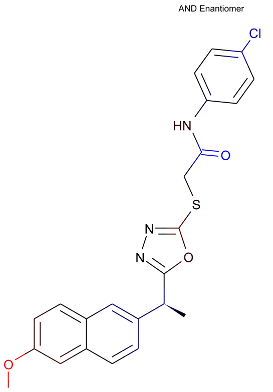

$C_{23}H_{20}ClN_3O_3S$
Molecular Weight: 453.9412
ALogP: 4.902
Rotatable Bonds: 7
Acceptors: 5
Donors: 1

Model Prediction

Prediction: 0.066
Unit: g/kg_body_weight
Mahalanobis Distance: 10.775
Mahalanobis Distance p-value: 2.21e-006

Mahalanobis Distance: The Mahalanobis distance (MD) is a generalization of the Euclidean distance that accounts for correlations among the X properties. It is calculated as the distance to the center of the training data. The larger the MD, the less trustworthy the prediction.
Mahalanobis Distance p-value: The p-value gives the fraction of training data with an MD greater than or equal to the one for the given sample, assuming normally distributed data. The smaller the p-value, the less trustworthy the prediciton. For highly non-normal X properties (e.g., fingerprints), the MD p-value is wildly inaccurate.

Structural Similar Compounds

Name	C.I.PIGMENT RED 3	CHLORBENZILATE	C.I.PIGMENT RED 23
Structure	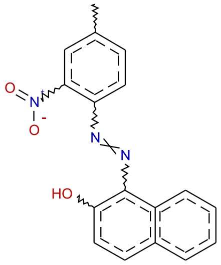	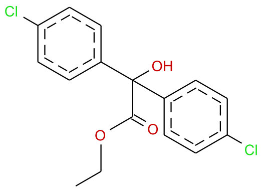	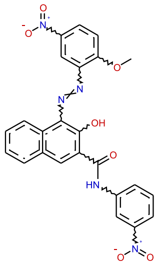
Actual Endpoint (-log C)	2.65635	3.38252	2.30052
Predicted Endpoint (-log C)	2.97957	3.27894	3.55333
Distance	0.766	0.879	0.879
Reference	NCI/NTP TR-407	NCI/NTP TR-75	NCI/NTP TR-411

Model Applicability

Unknown features are fingerprint features in the query molecule, but not found in the training set.

1. OPS PC9 out of range. Value: 4.8354. Training min, max, SD, explained variance: -2.8548, 3.3954, 1.263, 0.0360.
2. Unknown FCFP_2 feature: -1410079687: [*]S[c]1:o:[*]:[*]:n:1

Feature Contribution

Top features for positive contribution

Fingerprint	Bit/Smiles	Feature Structure	Score
FCFP_2	136627117	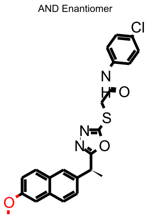 [*]OC	0.173

FCFP_2	1036089772	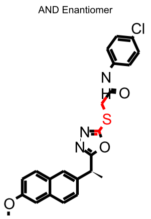 [*]CS[c](:[*]):[*]	0.075
FCFP_2	3	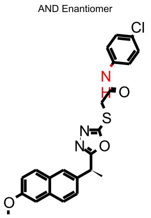 [*]N[*]	0.074

Top Features for negative contribution

Fingerprint	Bit/Smiles	Feature Structure	Score
FCFP_2	71476542	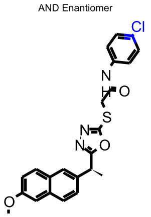 [*]:[c](:[*])Cl	-0.134
FCFP_2	1872154524	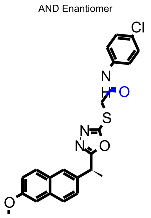 [*]C(=O)[*]	-0.105
FCFP_2	203677720	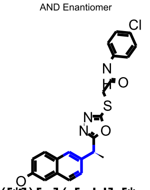 [*]C([*])[c](:[cH]:[*])):[cH]:[*]	-0.083

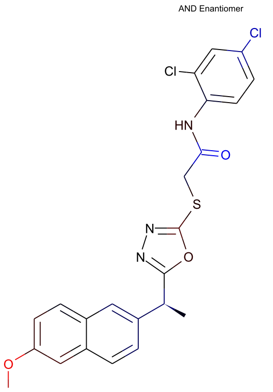

$C_{23}H_{19}Cl_2N_3O_3S$
Molecular Weight: 488.38626
ALogP: 5.567
Rotatable Bonds: 7
Acceptors: 5
Donors: 1

Model Prediction

Prediction: 0.053
Unit: g/kg_body_weight
Mahalanobis Distance: 10.723
Mahalanobis Distance p-value: 2.71e-006

Mahalanobis Distance: The Mahalanobis distance (MD) is a generalization of the Euclidean distance that accounts for correlations among the X properties. It is calculated as the distance to the center of the training data. The larger the MD, the less trustworthy the prediction.
Mahalanobis Distance p-value: The p-value gives the fraction of training data with an MD greater than or equal to the one for the given sample, assuming normally distributed data. The smaller the p-value, the less trustworthy the prediciton. For highly non-normal X properties (e.g., fingerprints), the MD p-value is wildly inaccurate.

Structural Similar Compounds

Name	C.I.PIGMENT RED 3	C.I.PIGMENT RED 23	CHLORBENZILATE
Structure	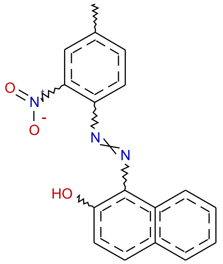	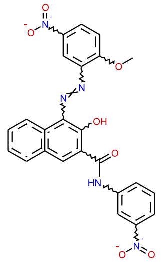	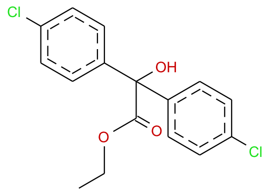
Actual Endpoint (-log C)	2.65635	2.30052	3.38252
Predicted Endpoint (-log C)	2.97957	3.55333	3.27894
Distance	0.804	0.874	0.910
Reference	NCI/NTP TR-407	NCI/NTP TR-411	NCI/NTP TR-75

Model Applicability

Unknown features are fingerprint features in the query molecule, but not found in the training set.

1. OPS PC9 out of range. Value: 4.9018. Training min, max, SD, explained variance: -2.8548, 3.3954, 1.263, 0.0360.
2. Unknown FCFP_2 feature: -1410079687: [*]S[c]1:o:[*]:[*]:n:1

Feature Contribution

Top features for positive contribution			
**Fingerprint**	**Bit/Smiles**	**Feature Structure**	**Score**
FCFP_2	136627117	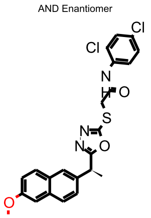 [*]OC	0.173

FCFP_2	1036089772	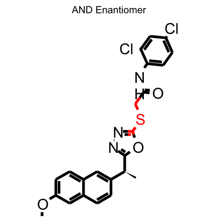 [*]CS[c](:[*]):[*]	0.075
FCFP_2	3	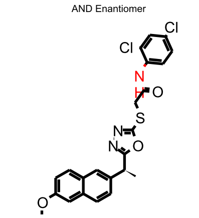 [*]N[*]	0.074

Top Features for negative contribution

Fingerprint	Bit/Smiles	Feature Structure	Score
FCFP_2	71476542	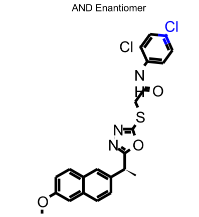 [*]:[c](:[*])Cl	-0.134
FCFP_2	1872154524	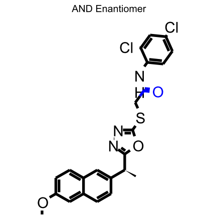 [*]C(=O)[*]	-0.105
FCFP_2	203677720	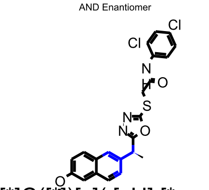 [*]C([*])[c](:[cH]:[*])):[cH]:[*]	-0.083

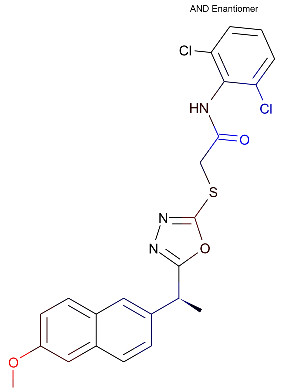

$C_{23}H_{19}Cl_2N_3O_3S$
Molecular Weight: 488.38626
ALogP: 5.567
Rotatable Bonds: 7
Acceptors: 5
Donors: 1

Model Prediction

Prediction: 0.053
Unit: g/kg_body_weight
Mahalanobis Distance: 10.723
Mahalanobis Distance p-value: 2.71e-006

Mahalanobis Distance: The Mahalanobis distance (MD) is a generalization of the Euclidean distance that accounts for correlations among the X properties. It is calculated as the distance to the center of the training data. The larger the MD, the less trustworthy the prediction.
Mahalanobis Distance p-value: The p-value gives the fraction of training data with an MD greater than or equal to the one for the given sample, assuming normally distributed data. The smaller the p-value, the less trustworthy the prediciton. For highly non-normal X properties (e.g., fingerprints), the MD p-value is wildly inaccurate.

Structural Similar Compounds

Name	C.I.PIGMENT RED 3	C.I.PIGMENT RED 23	CHLORBENZILATE
Structure	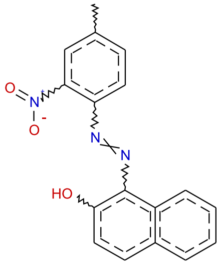	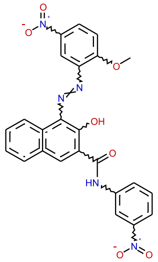	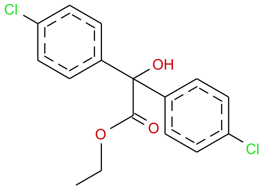
Actual Endpoint (-log C)	2.65635	2.30052	3.38252
Predicted Endpoint (-log C)	2.97957	3.55333	3.27894
Distance	0.804	0.874	0.910
Reference	NCI/NTP TR-407	NCI/NTP TR-411	NCI/NTP TR-75

Model Applicability

Unknown features are fingerprint features in the query molecule, but not found in the training set.

1. OPS PC9 out of range. Value: 4.9018. Training min, max, SD, explained variance: -2.8548, 3.3954, 1.263, 0.0360.
2. Unknown FCFP_2 feature: -1410079687: [*]S[c]1:o:[*]:[*]:n:1

Feature Contribution

Top features for positive contribution			
**Fingerprint**	**Bit/Smiles**	**Feature Structure**	**Score**
FCFP_2	136627117	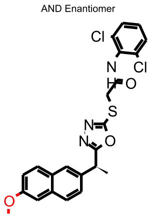 [*]OC	0.173

FCFP_2	1036089772	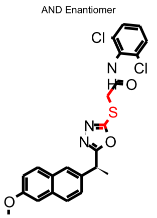 [*]CS[c](:[*]):[*]	0.075
FCFP_2	3	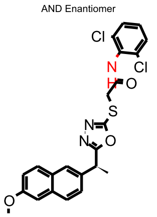 [*]N[*]	0.074

Top Features for negative contribution

Fingerprint	Bit/Smiles	Feature Structure	Score
FCFP_2	71476542	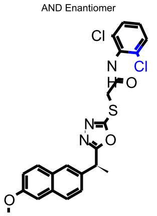 [*]:[c](:[*])Cl	-0.134
FCFP_2	1872154524	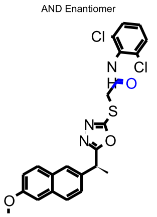 [*]C(=O)[*]	-0.105
FCFP_2	203677720	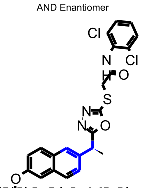 [*]C([*])[c](:[cH]:[*]):[cH]:[*]	-0.083

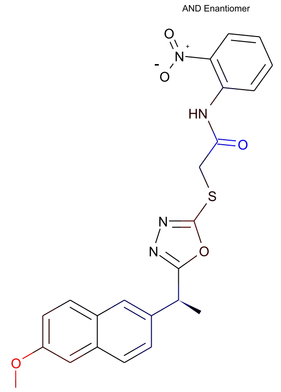

$C_{23}H_{20}N_4O_5S$

Molecular Weight: 464.4937

ALogP: 4.132

Rotatable Bonds: 8

Acceptors: 7

Donors: 1

Model Prediction

Prediction: 0.040

Unit: g/kg_body_weight

Mahalanobis Distance: 10.620

Mahalanobis Distance p-value: 4.09e-006

Mahalanobis Distance: The Mahalanobis distance (MD) is a generalization of the Euclidean distance that accounts for correlations among the X properties. It is calculated as the distance to the center of the training data. The larger the MD, the less trustworthy the prediction.

Mahalanobis Distance p-value: The p-value gives the fraction of training data with an MD greater than or equal to the one for the given sample, assuming normally distributed data. The smaller the p-value, the less trustworthy the prediciton. For highly non-normal X properties (e.g., fingerprints), the MD p-value is wildly inaccurate.

Structural Similar Compounds

Name	C.I.PIGMENT RED 23	SALICYLAZOSULFAPYRIDINE	RESERPINE
Structure	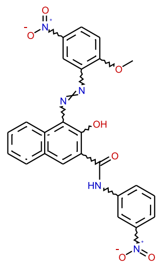	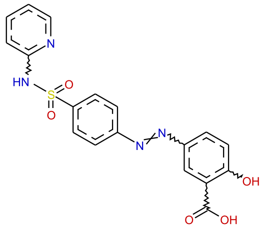	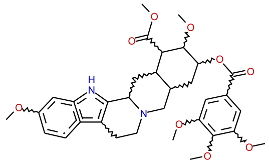
Actual Endpoint (-log C)	2.30052	3.375	6.13118
Predicted Endpoint (-log C)	3.55333	2.80292	4.38304
Distance	0.624	0.758	0.873
Reference	NCI/NTP TR-411	NCI/NTP TR-457	NCI/NTP TR-193

Model Applicability

Unknown features are fingerprint features in the query molecule, but not found in the training set.

1. OPS PC9 out of range. Value: 4.91. Training min, max, SD, explained variance: -2.8548, 3.3954, 1.263, 0.0360.
2. Unknown FCFP_2 feature: 8: [*][N+](=[*])[*]
3. Unknown FCFP_2 feature: 5: [*][O-]
4. Unknown FCFP_2 feature: -1410079687: [*]S[c]1:o:[*]:[*]:n:1
5. Unknown FCFP_2 feature: -828984032: [*][c](:[*]):[c]([N+](=[*])[*]):c:[*]
6. Unknown FCFP_2 feature: -1338588315: [*]:[c](:[*])[N+](=O)[O-]
7. Unknown FCFP_2 feature: 1872392852: [*][N+](=O)[*]
8. Unknown FCFP_2 feature: 260476081: [*][N+](=[*])[O-]

Feature Contribution

Top features for positive contribution

Fingerprint	Bit/Smiles	Feature Structure	Score

FCFP_2	136627117	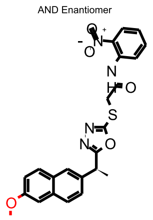 [*]OC	0.173
FCFP_2	1036089772	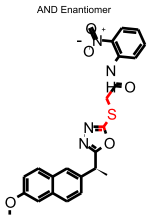 [*]CS[c](:[*]):[*]	0.075
FCFP_2	3	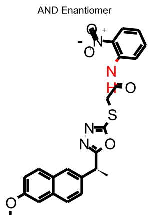 [*]N[*]	0.074
**Top Features for negative contribution**			
**Fingerprint**	**Bit/Smiles**	**Feature Structure**	**Score**
FCFP_2	1872154524	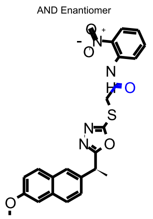 [*]C(=O)[*]	-0.105
FCFP_2	203677720	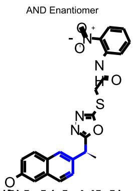 [*]C([*])[c](:[cH]:[*]):[cH]:[*]	-0.083

FCFP_2	1	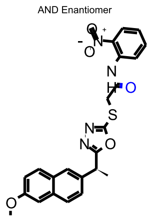<br>[*]=O	-0.080

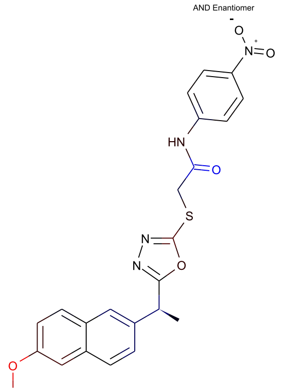

$C_{23}H_{20}N_4O_5S$
Molecular Weight: 464.4937
ALogP: 4.132
Rotatable Bonds: 8
Acceptors: 7
Donors: 1

Model Prediction

Prediction: 0.040
Unit: g/kg_body_weight
Mahalanobis Distance: 10.620
Mahalanobis Distance p-value: 4.09e-006

Mahalanobis Distance: The Mahalanobis distance (MD) is a generalization of the Euclidean distance that accounts for correlations among the X properties. It is calculated as the distance to the center of the training data. The larger the MD, the less trustworthy the prediction.
Mahalanobis Distance p-value: The p-value gives the fraction of training data with an MD greater than or equal to the one for the given sample, assuming normally distributed data. The smaller the p-value, the less trustworthy the prediciton. For highly non-normal X properties (e.g., fingerprints), the MD p-value is wildly inaccurate.

Structural Similar Compounds

Name	C.I.PIGMENT RED 23	SALICYLAZOSULFAPYRIDINE	RESERPINE
Structure	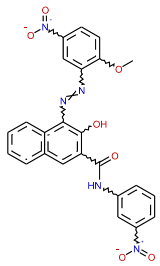	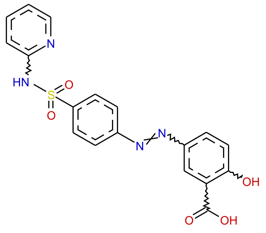	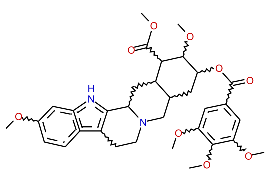
Actual Endpoint (-log C)	2.30052	3.375	6.13118
Predicted Endpoint (-log C)	3.55333	2.80292	4.38304
Distance	0.624	0.758	0.873
Reference	NCI/NTP TR-411	NCI/NTP TR-457	NCI/NTP TR-193

Model Applicability

Unknown features are fingerprint features in the query molecule, but not found in the training set.

1. OPS PC9 out of range. Value: 4.91. Training min, max, SD, explained variance: -2.8548, 3.3954, 1.263, 0.0360.
2. Unknown FCFP_2 feature: 8: [*][N+](=[*])[*]
3. Unknown FCFP_2 feature: 5: [*][O-]
4. Unknown FCFP_2 feature: -1410079687: [*]S[c]1:o:[*]:[*]:n:1
5. Unknown FCFP_2 feature: -828984032: [*][c](:[*]):[c]([N+](=[*])[*]):c:[*]
6. Unknown FCFP_2 feature: -1338588315: [*]:[c](:[*])[N+](=O)[O-]
7. Unknown FCFP_2 feature: 1872392852: [*][N+](=O)[*]
8. Unknown FCFP_2 feature: 260476081: [*][N+](=[*])[O-]

Feature Contribution

Top features for positive contribution

Fingerprint	Bit/Smiles	Feature Structure	Score

FCFP_2	136627117	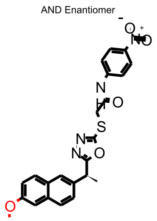<br>[*]OC	0.173
FCFP_2	1036089772	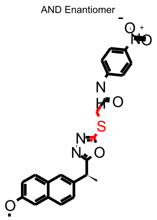<br>[*]CS[c](:[*]):[*]	0.075
FCFP_2	3	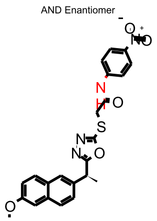<br>[*]N[*]	0.074

Top Features for negative contribution

**Fingerprint**	**Bit/Smiles**	**Feature Structure**	**Score**
FCFP_2	1872154524	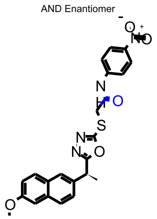<br>[*]C(=O)[*]	-0.105
FCFP_2	203677720	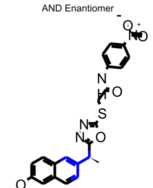<br>[*]C([*])[c](:[cH]:[*])):[cH]:[*]	-0.083

FCFP_2	1	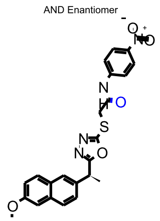 [*]=O	-0.080

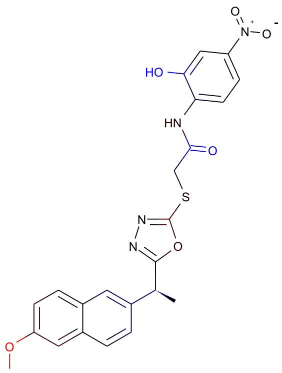

$C_{23}H_{20}N_4O_6S$

Molecular Weight: 480.4931

ALogP: 3.89

Rotatable Bonds: 8

Acceptors: 8

Donors: 2

Model Prediction

Prediction: 0.132

Unit: g/kg_body_weight

Mahalanobis Distance: 11.224

Mahalanobis Distance p-value: 3.49e-007

Mahalanobis Distance: The Mahalanobis distance (MD) is a generalization of the Euclidean distance that accounts for correlations among the X properties. It is calculated as the distance to the center of the training data. The larger the MD, the less trustworthy the prediction.
Mahalanobis Distance p-value: The p-value gives the fraction of training data with an MD greater than or equal to the one for the given sample, assuming normally distributed data. The smaller the p-value, the less trustworthy the prediciton. For highly non-normal X properties (e.g., fingerprints), the MD p-value is wildly inaccurate.

Structural Similar Compounds

Name	C.I.PIGMENT RED 23	SALICYLAZOSULFAPYRIDINE	RESERPINE
Structure	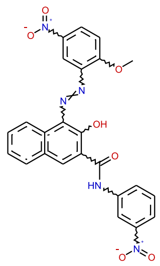	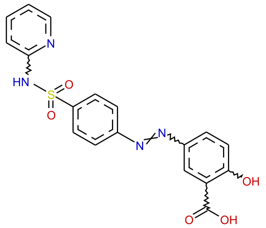	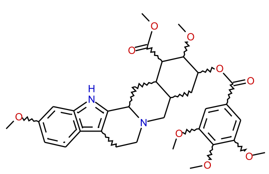
Actual Endpoint (-log C)	2.30052	3.375	6.13118
Predicted Endpoint (-log C)	3.55333	2.80292	4.38304
Distance	0.442	0.638	0.940
Reference	NCI/NTP TR-411	NCI/NTP TR-457	NCI/NTP TR-193

Model Applicability

Unknown features are fingerprint features in the query molecule, but not found in the training set.

1. OPS PC9 out of range. Value: 4.5479. Training min, max, SD, explained variance: -2.8548, 3.3954, 1.263, 0.0360.
2. Unknown FCFP_2 feature: 8: [*][N+](=[*])[*]
3. Unknown FCFP_2 feature: 5: [*][O-]
4. Unknown FCFP_2 feature: -1410079687: [*]S[c]1:o:[*]:[*]:n:1
5. Unknown FCFP_2 feature: -828984032: [*][c](:[*]):[c]([N+](=[*])[*]):c:[*]
6. Unknown FCFP_2 feature: -1338588315: [*]:[c](:[*])[N+](=O)[O-]
7. Unknown FCFP_2 feature: 1872392852: [*][N+](=O)[*]
8. Unknown FCFP_2 feature: 260476081: [*][N+](=[*])[O-]

Feature Contribution

Top features for positive contribution			
Fingerprint	Bit/Smiles	Feature Structure	Score

FCFP_2	136627117	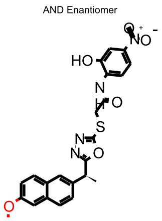 [*]OC	0.173
FCFP_2	1036089772	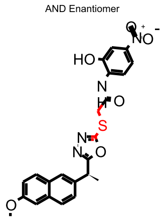 [*]CS[c](:[*]):[*]	0.075
FCFP_2	3	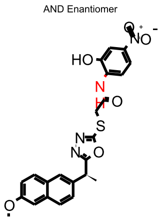 [*]N[*]	0.074
**Top Features for negative contribution**			
**Fingerprint**	**Bit/Smiles**	**Feature Structure**	**Score**
FCFP_2	7	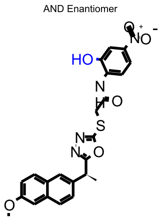 [*]O	-0.214
FCFP_2	-549108873	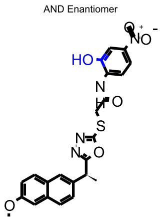 [*]:[c](:[*])O	-0.127

FCFP_2	1872154524	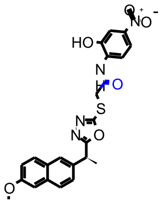 [*]C(=O)[*]	-0.105

Sorafenib TOPKAT_Rat_Maximum_Tolerated_Dose_Feed

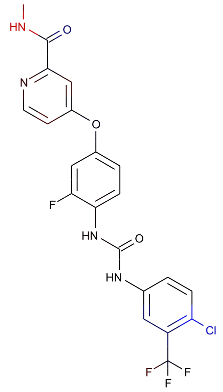

$C_{21}H_{15}ClF_4N_4O_3$

Molecular Weight: 482.81541

ALogP: 4.381

Rotatable Bonds: 6

Acceptors: 4

Donors: 3

Model Prediction

Prediction: 0.077

Unit: g/kg_body_weight

Mahalanobis Distance: 12.422

Mahalanobis Distance p-value: 1.91e-009

Mahalanobis Distance: The Mahalanobis distance (MD) is a generalization of the Euclidean distance that accounts for correlations among the X properties. It is calculated as the distance to the center of the training data. The larger the MD, the less trustworthy the prediction.
Mahalanobis Distance p-value: The p-value gives the fraction of training data with an MD greater than or equal to the one for the given sample, assuming normally distributed data. The smaller the p-value, the less trustworthy the prediciton. For highly non-normal X properties (e.g., fingerprints), the MD p-value is wildly inaccurate.

Structural Similar Compounds

Name	FUROSEMIDE	PHENOLPHTHALEIN	SALICYLAZOSULFAPYRIDINE
Structure	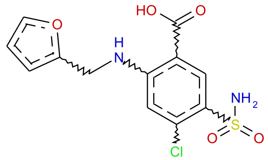	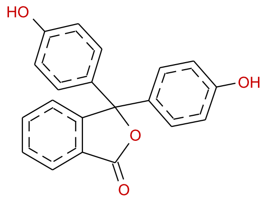	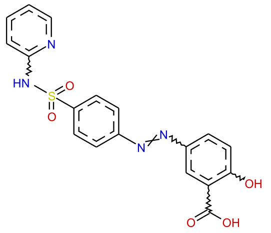
Actual Endpoint (-log C)	4.04236	2.20184	3.375
Predicted Endpoint (-log C)	2.8614	2.8857	2.80292
Distance	0.764	0.801	0.818
Reference	NCI/NTP TR-356	NCI/NTP TR-465	NCI/NTP TR-457

Model Applicability

Unknown features are fingerprint features in the query molecule, but not found in the training set.

1. All properties and OPS components are within expected ranges.

Feature Contribution

Top features for positive contribution

Fingerprint	Bit/Smiles	Feature Structure	Score
FCFP_2	-885550502	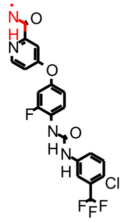 [*]CNC(=[*])[*]	0.115

FCFP_2	3	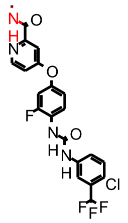<br>[*]N[*]	0.074
FCFP_2	332760439	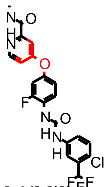<br>[*]O[c](:[cH]:[*]):[c H]:[*]	0.061

Top Features for negative contribution

Fingerprint	Bit/Smiles	Feature Structure	Score
FCFP_2	71476542	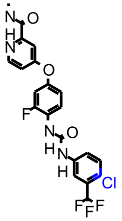<br>[*]:[c](:[*])Cl	-0.134
FCFP_2	1872154524	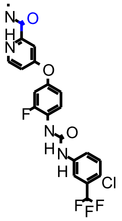<br>[*]C(=O)[*]	-0.105
FCFP_2	203677720	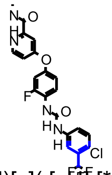<br>[*]C([*])[c](:[cH]:[*]):[cH]:[*]	-0.083

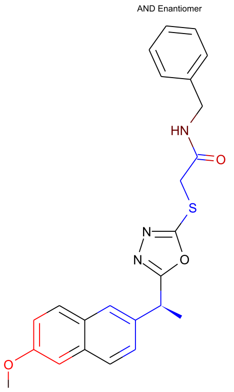

$C_{24}H_{23}N_3O_3S$

Molecular Weight: 433.52272

ALogP: 4.245

Rotatable Bonds: 8

Acceptors: 5

Donors: 1

Model Prediction

Prediction: 0.038

Unit: g/kg_body_weight

Mahalanobis Distance: 11.553

Mahalanobis Distance p-value: 3.81e-008

Mahalanobis Distance: The Mahalanobis distance (MD) is a generalization of the Euclidean distance that accounts for correlations among the X properties. It is calculated as the distance to the center of the training data. The larger the MD, the less trustworthy the prediction.
Mahalanobis Distance p-value: The p-value gives the fraction of training data with an MD greater than or equal to the one for the given sample, assuming normally distributed data. The smaller the p-value, the less trustworthy the prediciton. For highly non-normal X properties (e.g., fingerprints), the MD p-value is wildly inaccurate.

Structural Similar Compounds

Name	OCHRATOXIN	SULFISOOXAZOLE	PHENYLBUTAZONE
Structure	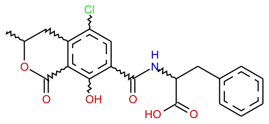	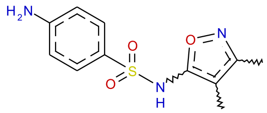	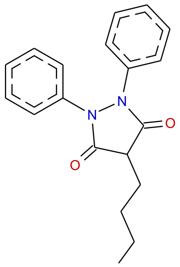
Actual Endpoint (-log C)	6.28396	2.82494	3.48909
Predicted Endpoint (-log C)	5.12358	3.0705	3.17333
Distance	1.109	1.177	1.202
Reference	NCI/NTP TR-358	NCI/NTP TR-138	NCI/NTP TR-367

Model Applicability

Unknown features are fingerprint features in the query molecule, but not found in the training set.

1. Num_AromaticRings out of range. Value: 4. Training min, max, mean, SD: 0, 2, 0.5625, 0.693.
2. OPS PC6 out of range. Value: -3.567. Training min, max, SD, explained variance: -2.4321, 2.9885, 1.256, 0.0488.
3. Unknown FCFP_2 feature: -1539162406: [*]C([*])[c]1:o:[*]:[*]:n:1

Feature Contribution

Top features for positive contribution

Fingerprint	Bit/Smiles	Feature Structure	Score
FCFP_2	332760439	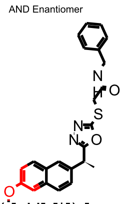 [*]O[c](:[cH]:[*]):[cH]:[*]	0.672

FCFP_2	1	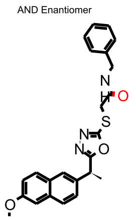 [*]=O	0.511
FCFP_2	3	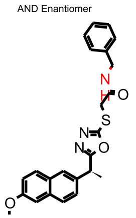 [*]N[*]	0.104

Top Features for negative contribution

Fingerprint	Bit/Smiles	Feature Structure	Score
FCFP_2	136597326	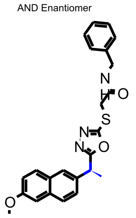 [*]C([*])C	-0.489
FCFP_2	203677720	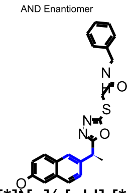 [*]C([*])[c](:[cH]:[*]):[cH]:[*]	-0.406
FCFP_2	1872154524	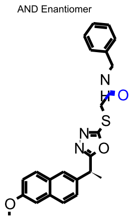 [*]C(=O)[*]	-0.307

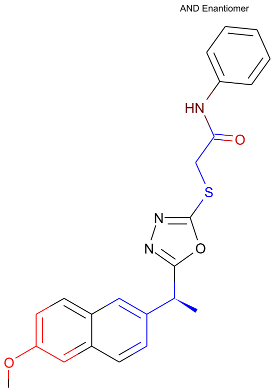

$C_{23}H_{21}N_3O_3S$
Molecular Weight: 419.49614
ALogP: 4.238
Rotatable Bonds: 7
Acceptors: 5
Donors: 1

Model Prediction

Prediction: 0.026
Unit: g/kg_body_weight
Mahalanobis Distance: 11.685
Mahalanobis Distance p-value: 2.48e-008

Mahalanobis Distance: The Mahalanobis distance (MD) is a generalization of the Euclidean distance that accounts for correlations among the X properties. It is calculated as the distance to the center of the training data. The larger the MD, the less trustworthy the prediction.
Mahalanobis Distance p-value: The p-value gives the fraction of training data with an MD greater than or equal to the one for the given sample, assuming normally distributed data. The smaller the p-value, the less trustworthy the prediciton. For highly non-normal X properties (e.g., fingerprints), the MD p-value is wildly inaccurate.

Structural Similar Compounds

Name	SULFISOOXAZOLE	OCHRATOXIN	PHENYLBUTAZONE
Structure	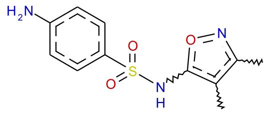	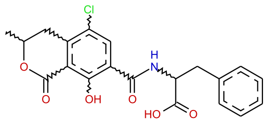	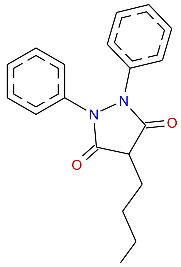
Actual Endpoint (-log C)	2.82494	6.28396	3.48909
Predicted Endpoint (-log C)	3.0705	5.12358	3.17333
Distance	1.094	1.110	1.181
Reference	NCI/NTP TR-138	NCI/NTP TR-358	NCI/NTP TR-367

Model Applicability

Unknown features are fingerprint features in the query molecule, but not found in the training set.

1. Num_AromaticRings out of range. Value: 4. Training min, max, mean, SD: 0, 2, 0.5625, 0.693.
2. OPS PC6 out of range. Value: -3.4935. Training min, max, SD, explained variance: -2.4321, 2.9885, 1.256, 0.0488.
3. Unknown FCFP_2 feature: -1539162406: [*]C([*])[c]1:o:[*]:[*]:n:1

Feature Contribution

Top features for positive contribution

Fingerprint	Bit/Smiles	Feature Structure	Score
FCFP_2	332760439	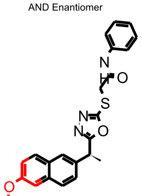 [*]O[c](:[cH]:[*]):[cH]:[*]	0.672

FCFP_2	1	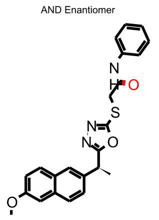 [*]=O	0.511
FCFP_2	3	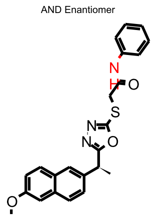 [*]N[*]	0.104

Top Features for negative contribution

Fingerprint	Bit/Smiles	Feature Structure	Score
FCFP_2	136597326	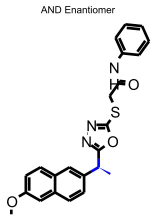 [*]C([*])C	-0.489
FCFP_2	203677720	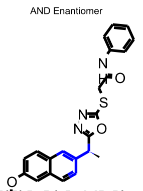 [*]C([*])[c](:[cH]:[*]):[cH]:[*]	-0.406
FCFP_2	1872154524	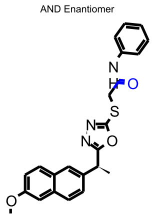 [*]C(=O)[*]	-0.307

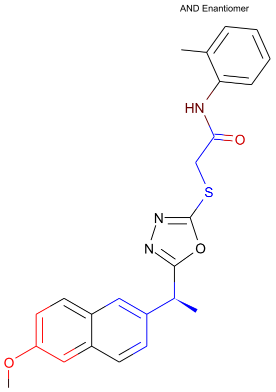

$C_{24}H_{23}N_3O_3S$

Molecular Weight: 433.52272

ALogP: 4.724

Rotatable Bonds: 7

Acceptors: 5

Donors: 1

Structural Similar Compounds

Name	SULFISOOXAZOLE	OCHRATOXIN	PHENYLBUTAZONE
Structure	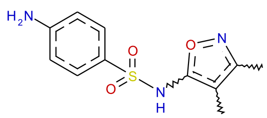	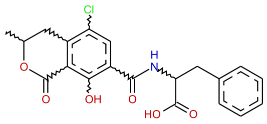	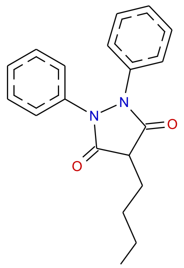
Actual Endpoint (-log C)	2.82494	6.28396	3.48909
Predicted Endpoint (-log C)	3.0705	5.12358	3.17333
Distance	1.112	1.124	1.198
Reference	NCI/NTP TR-138	NCI/NTP TR-358	NCI/NTP TR-367

Model Prediction

Prediction: 0.020

Unit: g/kg_body_weight

Mahalanobis Distance: 11.752

Mahalanobis Distance p-value: 2e-008

Mahalanobis Distance: The Mahalanobis distance (MD) is a generalization of the Euclidean distance that accounts for correlations among the X properties. It is calculated as the distance to the center of the training data. The larger the MD, the less trustworthy the prediction.
Mahalanobis Distance p-value: The p-value gives the fraction of training data with an MD greater than or equal to the one for the given sample, assuming normally distributed data. The smaller the p-value, the less trustworthy the prediciton. For highly non-normal X properties (e.g., fingerprints), the MD p-value is wildly inaccurate.

Model Applicability

Unknown features are fingerprint features in the query molecule, but not found in the training set.

1. Num_AromaticRings out of range. Value: 4. Training min, max, mean, SD: 0, 2, 0.5625, 0.693.
2. OPS PC6 out of range. Value: -3.5573. Training min, max, SD, explained variance: -2.4321, 2.9885, 1.256, 0.0488.
3. Unknown FCFP_2 feature: -1539162406: [*]C([*])[c]1:o:[*]:[*]:n:1

Feature Contribution

Top features for positive contribution

Fingerprint	Bit/Smiles	Feature Structure	Score
FCFP_2	332760439	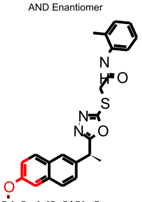 [*]O[c](:[cH]:[*]):[cH]:[*]	0.672

FCFP_2	1	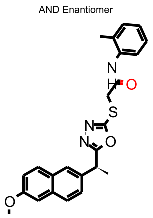 [*]=O	0.511
FCFP_2	3	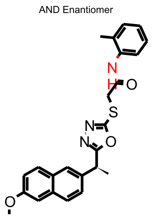 [*]N[*]	0.104

Top Features for negative contribution

Fingerprint	Bit/Smiles	Feature Structure	Score
FCFP_2	136597326	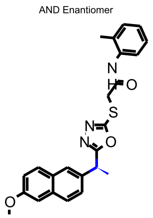 [*]C([*])C	-0.489
FCFP_2	203677720	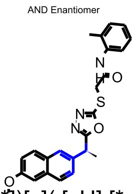 [*]C([*])[c](:[cH]:[*])):[cH]:[*]	-0.406
FCFP_2	1872154524	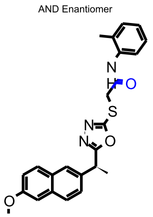 [*]C(=O)[*]	-0.307

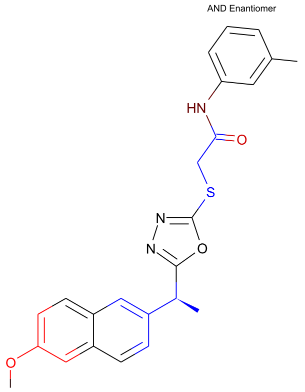

$C_{24}H_{23}N_3O_3S$

Molecular Weight: 433.52272

ALogP: 4.724

Rotatable Bonds: 7

Acceptors: 5

Donors: 1

Model Prediction

Prediction: 0.020

Unit: g/kg_body_weight

Mahalanobis Distance: 11.752

Mahalanobis Distance p-value: 2e-008

Mahalanobis Distance: The Mahalanobis distance (MD) is a generalization of the Euclidean distance that accounts for correlations among the X properties. It is calculated as the distance to the center of the training data. The larger the MD, the less trustworthy the prediction.
Mahalanobis Distance p-value: The p-value gives the fraction of training data with an MD greater than or equal to the one for the given sample, assuming normally distributed data. The smaller the p-value, the less trustworthy the prediciton. For highly non-normal X properties (e.g., fingerprints), the MD p-value is wildly inaccurate.

Structural Similar Compounds

Name	SULFISOOXAZOLE	OCHRATOXIN	PHENYLBUTAZONE
Structure	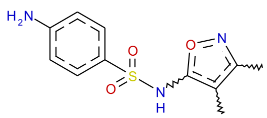	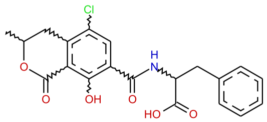	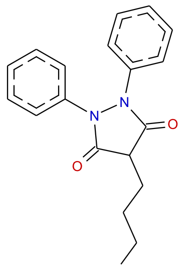
Actual Endpoint (-log C)	2.82494	6.28396	3.48909
Predicted Endpoint (-log C)	3.0705	5.12358	3.17333
Distance	1.112	1.124	1.198
Reference	NCI/NTP TR-138	NCI/NTP TR-358	NCI/NTP TR-367

Model Applicability

Unknown features are fingerprint features in the query molecule, but not found in the training set.

1. Num_AromaticRings out of range. Value: 4. Training min, max, mean, SD: 0, 2, 0.5625, 0.693.
2. OPS PC6 out of range. Value: -3.5573. Training min, max, SD, explained variance: -2.4321, 2.9885, 1.256, 0.0488.
3. Unknown FCFP_2 feature: -1539162406: [*]C([*])[c]1:o:[*]:[*]:n:1

Feature Contribution

Top features for positive contribution

Fingerprint	Bit/Smiles	Feature Structure	Score
FCFP_2	332760439	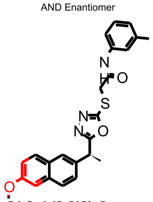 [*]O[c](:[cH]:[*]):[cH]:[*]	0.672

FCFP_2	1	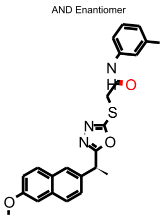 [*]=O	0.511
FCFP_2	3	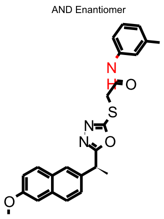 [*]N[*]	0.104

Top Features for negative contribution

Fingerprint	Bit/Smiles	Feature Structure	Score
FCFP_2	136597326	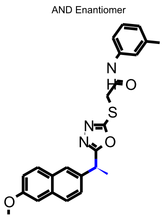 [*]C([*])C	-0.489
FCFP_2	203677720	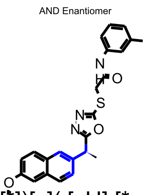 [*]C([*])[c](:[cH]:[*]):[cH]:[*]	-0.406
FCFP_2	1872154524	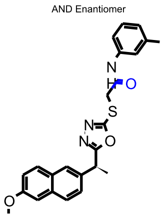 [*]C(=O)[*]	-0.307

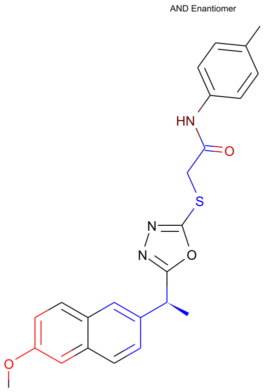

$C_{24}H_{23}N_3O_3S$
Molecular Weight: 433.52272
ALogP: 4.724
Rotatable Bonds: 7
Acceptors: 5
Donors: 1

Model Prediction

Prediction: 0.020
Unit: g/kg_body_weight
Mahalanobis Distance: 11.752
Mahalanobis Distance p-value: 2e-008

Mahalanobis Distance: The Mahalanobis distance (MD) is a generalization of the Euclidean distance that accounts for correlations among the X properties. It is calculated as the distance to the center of the training data. The larger the MD, the less trustworthy the prediction.
Mahalanobis Distance p-value: The p-value gives the fraction of training data with an MD greater than or equal to the one for the given sample, assuming normally distributed data. The smaller the p-value, the less trustworthy the prediciton. For highly non-normal X properties (e.g., fingerprints), the MD p-value is wildly inaccurate.

Structural Similar Compounds

Name	SULFISOOXAZOLE	OCHRATOXIN	PHENYLBUTAZONE
Structure	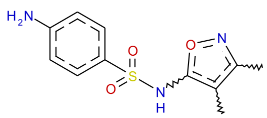	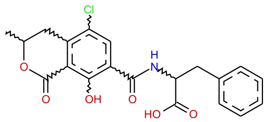	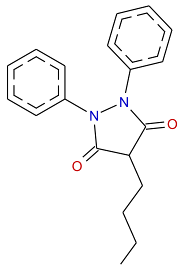
Actual Endpoint (-log C)	2.82494	6.28396	3.48909
Predicted Endpoint (-log C)	3.0705	5.12358	3.17333
Distance	1.112	1.124	1.198
Reference	NCI/NTP TR-138	NCI/NTP TR-358	NCI/NTP TR-367

Model Applicability

Unknown features are fingerprint features in the query molecule, but not found in the training set.

1. Num_AromaticRings out of range. Value: 4. Training min, max, mean, SD: 0, 2, 0.5625, 0.693.
2. OPS PC6 out of range. Value: -3.5573. Training min, max, SD, explained variance: -2.4321, 2.9885, 1.256, 0.0488.
3. Unknown FCFP_2 feature: -1539162406: [*]C([*])[c]1:o:[*]:[*]:n:1

Feature Contribution

Top features for positive contribution

Fingerprint	Bit/Smiles	Feature Structure	Score
FCFP_2	332760439	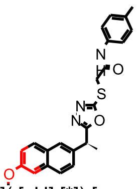 AND Enantiomer [*]O[c](:[cH]:[*]):[cH]:[*]	0.672

FCFP_2	1	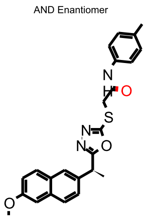 [*]=O	0.511
FCFP_2	3	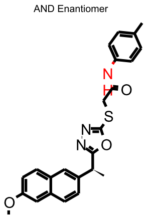 [*]N[*]	0.104

Top Features for negative contribution

Fingerprint	Bit/Smiles	Feature Structure	Score
FCFP_2	136597326	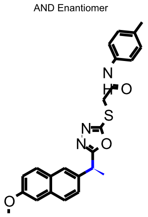 [*]C([*])C	-0.489
FCFP_2	203677720	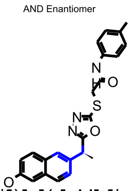 [*]C([*])[c](:[cH]:[*]):[cH]:[*]	-0.406
FCFP_2	1872154524	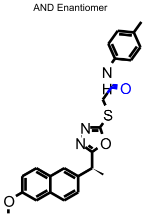 [*]C(=O)[*]	-0.307

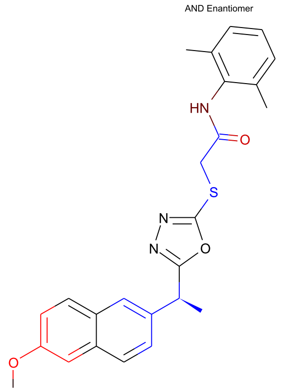

$C_{25}H_{25}N_3O_3S$
Molecular Weight: 447.5493
ALogP: 5.21
Rotatable Bonds: 7
Acceptors: 5
Donors: 1

Model Prediction

Prediction: 0.015
Unit: g/kg_body_weight
Mahalanobis Distance: 11.855
Mahalanobis Distance p-value: 1.42e-008

Mahalanobis Distance: The Mahalanobis distance (MD) is a generalization of the Euclidean distance that accounts for correlations among the X properties. It is calculated as the distance to the center of the training data. The larger the MD, the less trustworthy the prediction.
Mahalanobis Distance p-value: The p-value gives the fraction of training data with an MD greater than or equal to the one for the given sample, assuming normally distributed data. The smaller the p-value, the less trustworthy the prediciton. For highly non-normal X properties (e.g., fingerprints), the MD p-value is wildly inaccurate.

Structural Similar Compounds

Name	OCHRATOXIN	SULFISOOXAZOLE	PHENYLBUTAZONE
Structure	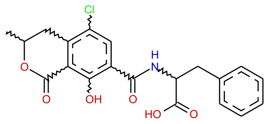	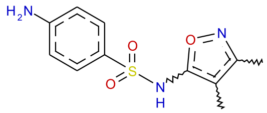	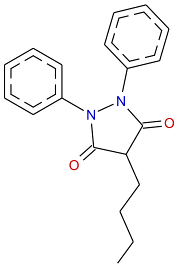
Actual Endpoint (-log C)	6.28396	2.82494	3.48909
Predicted Endpoint (-log C)	5.12358	3.0705	3.17333
Distance	1.140	1.152	1.215
Reference	NCI/NTP TR-358	NCI/NTP TR-138	NCI/NTP TR-367

Model Applicability

Unknown features are fingerprint features in the query molecule, but not found in the training set.

1. Molecular_Weight out of range. Value: 447.55. Training min, max, mean, SD: 68.074, 434.63, 171.13, 85.06.
2. Num_AromaticRings out of range. Value: 4. Training min, max, mean, SD: 0, 2, 0.5625, 0.693.
3. OPS PC6 out of range. Value: -3.6212. Training min, max, SD, explained variance: -2.4321, 2.9885, 1.256, 0.0488.
4. Unknown FCFP_2 feature: -1539162406: [*]C([*])[c]1:o:[*]:[*]:n:1

Feature Contribution

Top features for positive contribution

Fingerprint	Bit/Smiles	Feature Structure	Score
FCFP_2	332760439	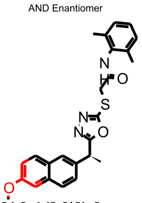 [*]O[c](:[cH]:[*]):[cH]:[*]	0.672

FCFP_2	1	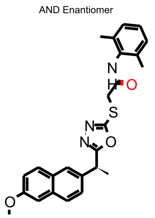 [*]=O	0.511
FCFP_2	3	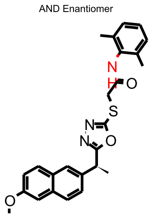 [*]N[*]	0.104
**Top Features for negative contribution**			
**Fingerprint**	**Bit/Smiles**	**Feature Structure**	**Score**
FCFP_2	136597326	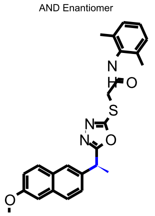 [*]C([*])C	-0.489
FCFP_2	203677720	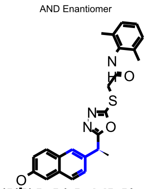 [*]C([*])[c](:[cH]:[*]):[cH]:[*]	-0.406
FCFP_2	1872154524	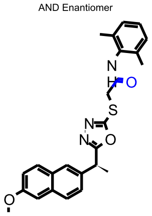 [*]C(=O)[*]	-0.307

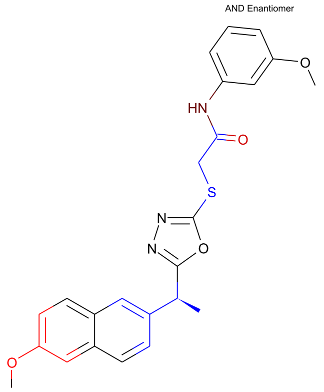

$C_{24}H_{23}N_3O_4S$

Molecular Weight: 449.52212

ALogP: 4.221

Rotatable Bonds: 8

Acceptors: 6

Donors: 1

Model Prediction

Prediction: 0.010

Unit: g/kg_body_weight

Mahalanobis Distance: 11.843

Mahalanobis Distance p-value: 1.48e-008

Mahalanobis Distance: The Mahalanobis distance (MD) is a generalization of the Euclidean distance that accounts for correlations among the X properties. It is calculated as the distance to the center of the training data. The larger the MD, the less trustworthy the prediction.
Mahalanobis Distance p-value: The p-value gives the fraction of training data with an MD greater than or equal to the one for the given sample, assuming normally distributed data. The smaller the p-value, the less trustworthy the prediciton. For highly non-normal X properties (e.g., fingerprints), the MD p-value is wildly inaccurate.

Structural Similar Compounds

Name	OCHRATOXIN	SULFISOOXAZOLE	PENICILLIN VK
Structure	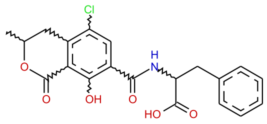	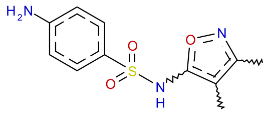	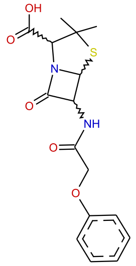
Actual Endpoint (-log C)	6.28396	2.82494	2.54455
Predicted Endpoint (-log C)	5.12358	3.0705	3.9702
Distance	1.115	1.160	1.260
Reference	NCI/NTP TR-358	NCI/NTP TR-138	NCI/NTP TR-336

Model Applicability

Unknown features are fingerprint features in the query molecule, but not found in the training set.

1. Molecular_Weight out of range. Value: 449.52. Training min, max, mean, SD: 68.074, 434.63, 171.13, 85.06.
2. Num_AromaticRings out of range. Value: 4. Training min, max, mean, SD: 0, 2, 0.5625, 0.693.
3. OPS PC6 out of range. Value: -3.5916. Training min, max, SD, explained variance: -2.4321, 2.9885, 1.256, 0.0488.
4. Unknown FCFP_2 feature: -1539162406: [*]C([*])[c]1:o:[*]:[*]:n:1

Feature Contribution

Top features for positive contribution

Fingerprint	Bit/Smiles	Feature Structure	Score
FCFP_2	332760439	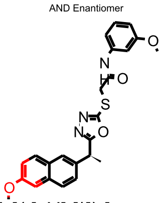 [*]O[c](:[cH]:[*]):[cH]:[*]	0.672

FCFP_2	1	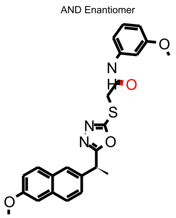 [*]=O	0.511
FCFP_2	3	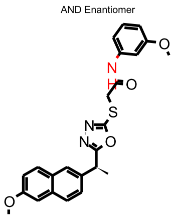 [*]N[*]	0.104

Top Features for negative contribution

**Fingerprint**	**Bit/Smiles**	**Feature Structure**	**Score**
FCFP_2	136597326	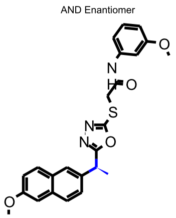 [*]C([*])C	-0.489
FCFP_2	203677720	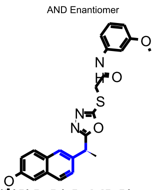 [*]C([*])[c](:[cH]:[*])):[cH]:[*]	-0.406
FCFP_2	1872154524	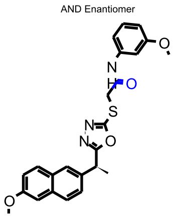 [*]C(=O)[*]	-0.307

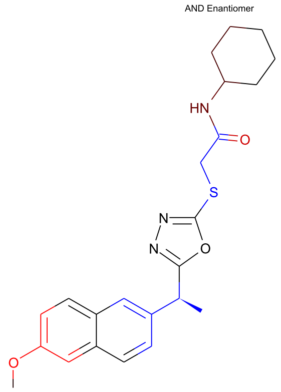

$C_{23}H_{27}N_3O_3S$
Molecular Weight: 425.54378
ALogP: 4.52
Rotatable Bonds: 7
Acceptors: 5
Donors: 1

Model Prediction

Prediction: 0.021
Unit: g/kg_body_weight
Mahalanobis Distance: 12.014
Mahalanobis Distance p-value: 8.45e-009

Mahalanobis Distance: The Mahalanobis distance (MD) is a generalization of the Euclidean distance that accounts for correlations among the X properties. It is calculated as the distance to the center of the training data. The larger the MD, the less trustworthy the prediction.
Mahalanobis Distance p-value: The p-value gives the fraction of training data with an MD greater than or equal to the one for the given sample, assuming normally distributed data. The smaller the p-value, the less trustworthy the prediciton. For highly non-normal X properties (e.g., fingerprints), the MD p-value is wildly inaccurate.

Structural Similar Compounds

Name	OCHRATOXIN	PROBENECID	PENICILLIN VK
Structure	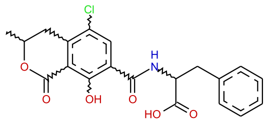	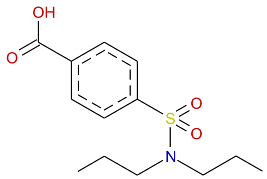	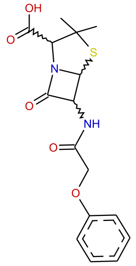
Actual Endpoint (-log C)	6.28396	2.85333	2.54455
Predicted Endpoint (-log C)	5.12358	2.4258	3.9702
Distance	0.933	1.022	1.032
Reference	NCI/NTP TR-358	NCI/NTP TR-395	NCI/NTP TR-336

Model Applicability

Unknown features are fingerprint features in the query molecule, but not found in the training set.

1. Num_AromaticRings out of range. Value: 3. Training min, max, mean, SD: 0, 2, 0.5625, 0.693.
2. OPS PC6 out of range. Value: -3.1611. Training min, max, SD, explained variance: -2.4321, 2.9885, 1.256, 0.0488.
3. Unknown FCFP_2 feature: -1539162406: [*]C([*])[c]1:o:[*]:[*]:n:1

Feature Contribution

Top features for positive contribution

Fingerprint	Bit/Smiles	Feature Structure	Score
FCFP_2	332760439	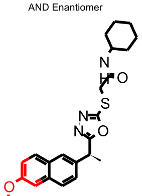 [*]O[c](:[cH]:[*]):[cH]:[*]	0.672

FCFP_2	1	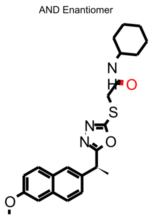 [*]=O	0.511
FCFP_2	3	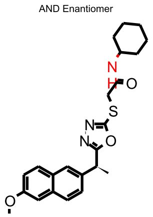 [*]N[*]	0.104

Top Features for negative contribution

Fingerprint	Bit/Smiles	Feature Structure	Score
FCFP_2	136597326	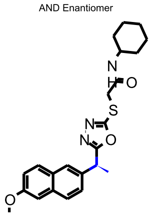 [*]C([*])C	-0.489
FCFP_2	203677720	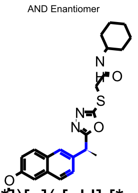 [*]C([*])[c](:[cH]:[*]):[cH]:[*]	-0.406
FCFP_2	1872154524	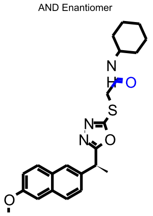 [*]C(=O)[*]	-0.307

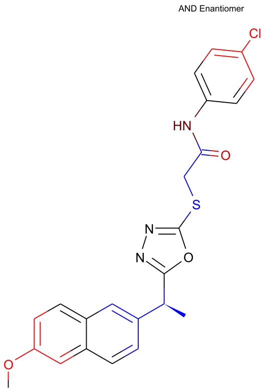

$C_{23}H_{20}ClN_3O_3S$
Molecular Weight: 453.9412
ALogP: 4.902
Rotatable Bonds: 7
Acceptors: 5
Donors: 1

Model Prediction

Prediction: 0.002
Unit: g/kg_body_weight
Mahalanobis Distance: 11.470
Mahalanobis Distance p-value: 5.01e-008

Mahalanobis Distance: The Mahalanobis distance (MD) is a generalization of the Euclidean distance that accounts for correlations among the X properties. It is calculated as the distance to the center of the training data. The larger the MD, the less trustworthy the prediction.
Mahalanobis Distance p-value: The p-value gives the fraction of training data with an MD greater than or equal to the one for the given sample, assuming normally distributed data. The smaller the p-value, the less trustworthy the prediciton. For highly non-normal X properties (e.g., fingerprints), the MD p-value is wildly inaccurate.

Structural Similar Compounds

Name	OCHRATOXIN	SULFISOOXAZOLE	PHENYLBUTAZONE
Structure	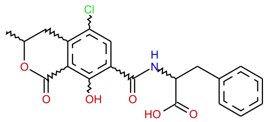	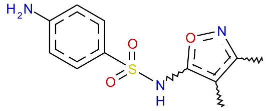	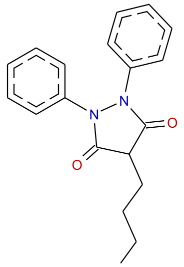
Actual Endpoint (-log C)	6.28396	2.82494	3.48909
Predicted Endpoint (-log C)	5.12358	3.0705	3.17333
Distance	1.090	1.179	1.224
Reference	NCI/NTP TR-358	NCI/NTP TR-138	NCI/NTP TR-367

Model Applicability

Unknown features are fingerprint features in the query molecule, but not found in the training set.

1. Molecular_Weight out of range. Value: 453.94. Training min, max, mean, SD: 68.074, 434.63, 171.13, 85.06.
2. Num_AromaticRings out of range. Value: 4. Training min, max, mean, SD: 0, 2, 0.5625, 0.693.
3. OPS PC5 out of range. Value: -4.0051. Training min, max, SD, explained variance: -3.4, 4.1587, 1.489, 0.0686.
4. Unknown FCFP_2 feature: -1539162406: [*]C([*])[c]1:o:[*]:[*]:n:1

Feature Contribution

Top features for positive contribution

Fingerprint	Bit/Smiles	Feature Structure	Score
FCFP_2	332760439	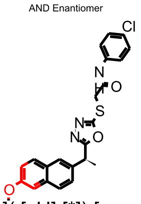 [*]O[c](:[cH]:[*]):[cH]:[*]	0.672

FCFP_2	32	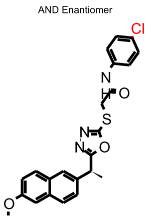 [*]Cl	0.526
FCFP_2	1	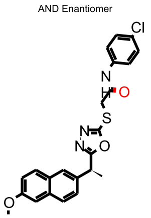 [*]=O	0.511
**Top Features for negative contribution**			
**Fingerprint**	**Bit/Smiles**	**Feature Structure**	**Score**
FCFP_2	136597326	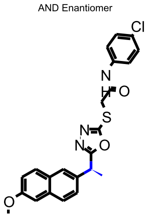 [*]C([*])C	-0.489
FCFP_2	203677720	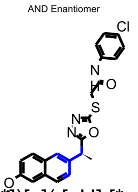 [*]C([*])[c](:[cH]:[*]):[cH]:[*]	-0.406
FCFP_2	1872154524	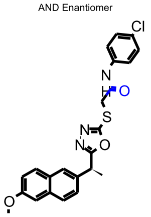 [*]C(=O)[*]	-0.307

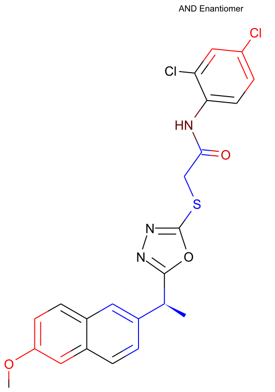

$C_{23}H_{19}Cl_2N_3O_3S$
Molecular Weight: 488.38626
ALogP: 5.567
Rotatable Bonds: 7
Acceptors: 5
Donors: 1

Model Prediction

Prediction: 0.001
Unit: g/kg_body_weight
Mahalanobis Distance: 11.501
Mahalanobis Distance p-value: 4.52e-008

Mahalanobis Distance: The Mahalanobis distance (MD) is a generalization of the Euclidean distance that accounts for correlations among the X properties. It is calculated as the distance to the center of the training data. The larger the MD, the less trustworthy the prediction.
Mahalanobis Distance p-value: The p-value gives the fraction of training data with an MD greater than or equal to the one for the given sample, assuming normally distributed data. The smaller the p-value, the less trustworthy the prediciton. For highly non-normal X properties (e.g., fingerprints), the MD p-value is wildly inaccurate.

Structural Similar Compounds

Name	OCHRATOXIN	SULFISOOXAZOLE	PHENYLBUTAZONE
Structure	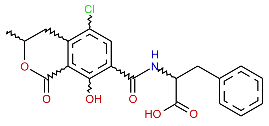	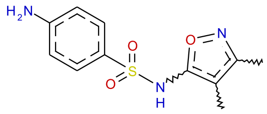	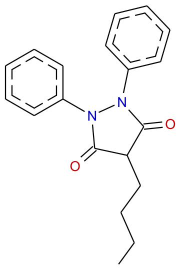
Actual Endpoint (-log C)	6.28396	2.82494	3.48909
Predicted Endpoint (-log C)	5.12358	3.0705	3.17333
Distance	1.124	1.251	1.264
Reference	NCI/NTP TR-358	NCI/NTP TR-138	NCI/NTP TR-367

Model Applicability

Unknown features are fingerprint features in the query molecule, but not found in the training set.

1. Molecular_Weight out of range. Value: 488.39. Training min, max, mean, SD: 68.074, 434.63, 171.13, 85.06.
2. Num_AromaticRings out of range. Value: 4. Training min, max, mean, SD: 0, 2, 0.5625, 0.693.
3. OPS PC5 out of range. Value: -4.0231. Training min, max, SD, explained variance: -3.4, 4.1587, 1.489, 0.0686.
4. OPS PC7 out of range. Value: -2.8101. Training min, max, SD, explained variance: -2.8003, 2.9332, 1.16, 0.0416.
5. Unknown FCFP_2 feature: -1539162406: [*]C([*])[c]1:o:[*]:[*]:n:1

Feature Contribution

Top features for positive contribution

Fingerprint	Bit/Smiles	Feature Structure	Score
FCFP_2	332760439	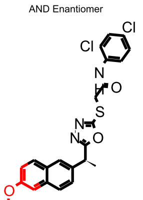 [*]O[c](:[cH]:[*]):[cH]:[*]	0.672

FCFP_2	32	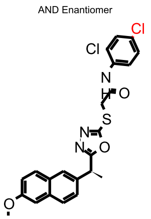 [*]Cl	0.526
FCFP_2	1	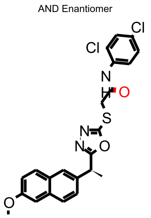 [*]=O	0.511
**Top Features for negative contribution**			
**Fingerprint**	**Bit/Smiles**	**Feature Structure**	**Score**
FCFP_2	136597326	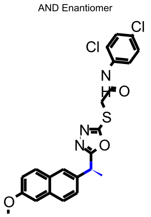 [*]C([*])C	-0.489
FCFP_2	203677720	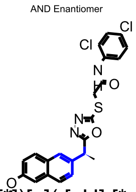 [*]C([*])[c](:[cH]:[*]):[cH]:[*]	-0.406
FCFP_2	1872154524	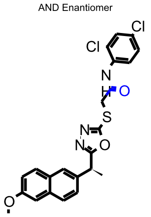 [*]C(=O)[*]	-0.307

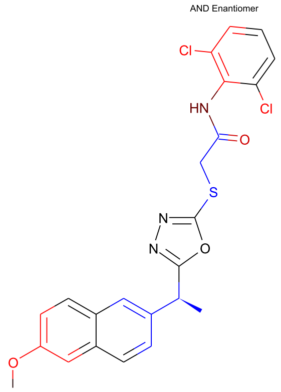

$C_{23}H_{19}Cl_2N_3O_3S$

Molecular Weight: 488.38626

ALogP: 5.567

Rotatable Bonds: 7

Acceptors: 5

Donors: 1

Model Prediction

Prediction: 0.001

Unit: g/kg_body_weight

Mahalanobis Distance: 11.501

Mahalanobis Distance p-value: 4.52e-008

Mahalanobis Distance: The Mahalanobis distance (MD) is a generalization of the Euclidean distance that accounts for correlations among the X properties. It is calculated as the distance to the center of the training data. The larger the MD, the less trustworthy the prediction.
Mahalanobis Distance p-value: The p-value gives the fraction of training data with an MD greater than or equal to the one for the given sample, assuming normally distributed data. The smaller the p-value, the less trustworthy the prediciton. For highly non-normal X properties (e.g., fingerprints), the MD p-value is wildly inaccurate.

Structural Similar Compounds

Name	OCHRATOXIN	SULFISOOXAZOLE	PHENYLBUTAZONE
Structure	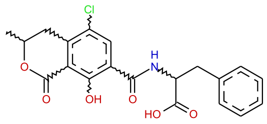	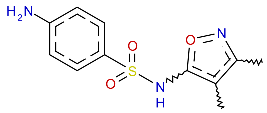	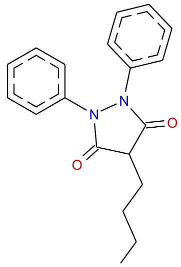
Actual Endpoint (-log C)	6.28396	2.82494	3.48909
Predicted Endpoint (-log C)	5.12358	3.0705	3.17333
Distance	1.124	1.251	1.264
Reference	NCI/NTP TR-358	NCI/NTP TR-138	NCI/NTP TR-367

Model Applicability

Unknown features are fingerprint features in the query molecule, but not found in the training set.

1. Molecular_Weight out of range. Value: 488.39. Training min, max, mean, SD: 68.074, 434.63, 171.13, 85.06.
2. Num_AromaticRings out of range. Value: 4. Training min, max, mean, SD: 0, 2, 0.5625, 0.693.
3. OPS PC5 out of range. Value: -4.0231. Training min, max, SD, explained variance: -3.4, 4.1587, 1.489, 0.0686.
4. OPS PC7 out of range. Value: -2.8101. Training min, max, SD, explained variance: -2.8003, 2.9332, 1.16, 0.0416.
5. Unknown FCFP_2 feature: -1539162406: [*]C([*])[c]1:o:[*]:[*]:n:1

Feature Contribution

Top features for positive contribution			
**Fingerprint**	**Bit/Smiles**	**Feature Structure**	**Score**
FCFP_2	332760439	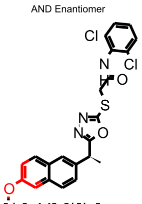 [*]O[c](:[cH]:[*]):[cH]:[*]	0.672

FCFP_2	32	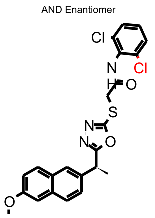 [*]Cl	0.526
FCFP_2	1	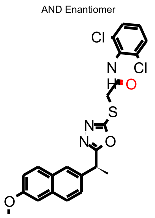 [*]=O	0.511

Top Features for negative contribution

Fingerprint	Bit/Smiles	Feature Structure	Score
FCFP_2	136597326	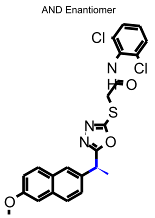 [*]C([*])C	-0.489
FCFP_2	203677720	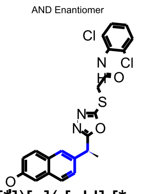 [*]C([*])[c](:[cH]:[*]):[cH]:[*]	-0.406
FCFP_2	1872154524	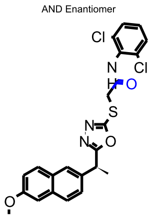 [*]C(=O)[*]	-0.307

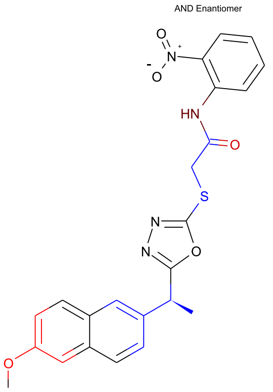

$C_{23}H_{20}N_4O_5S$
Molecular Weight: 464.4937
ALogP: 4.132
Rotatable Bonds: 8
Acceptors: 7
Donors: 1

Model Prediction

Prediction: 0.008
Unit: g/kg_body_weight
Mahalanobis Distance: 12.920
Mahalanobis Distance p-value: 4.34e-010

Mahalanobis Distance: The Mahalanobis distance (MD) is a generalization of the Euclidean distance that accounts for correlations among the X properties. It is calculated as the distance to the center of the training data. The larger the MD, the less trustworthy the prediction.
Mahalanobis Distance p-value: The p-value gives the fraction of training data with an MD greater than or equal to the one for the given sample, assuming normally distributed data. The smaller the p-value, the less trustworthy the prediciton. For highly non-normal X properties (e.g., fingerprints), the MD p-value is wildly inaccurate.

Structural Similar Compounds

Name	OCHRATOXIN	SULFISOOXAZOLE	PENICILLIN VK
Structure	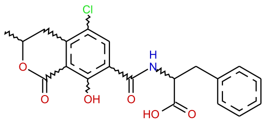	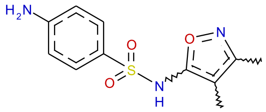	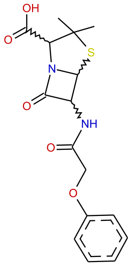
Actual Endpoint (-log C)	6.28396	2.82494	2.54455
Predicted Endpoint (-log C)	5.12358	3.0705	3.9702
Distance	1.157	1.266	1.307
Reference	NCI/NTP TR-358	NCI/NTP TR-138	NCI/NTP TR-336

Model Applicability

Unknown features are fingerprint features in the query molecule, but not found in the training set.

1. Molecular_Weight out of range. Value: 464.49. Training min, max, mean, SD: 68.074, 434.63, 171.13, 85.06.
2. Num_H_Acceptors out of range. Value: 7. Training min, max, mean, SD: 0, 6, 1.6146, 1.644.
3. Num_AromaticRings out of range. Value: 4. Training min, max, mean, SD: 0, 2, 0.5625, 0.693.
4. Molecular_PolarSurfaceArea out of range. Value: 148.37. Training min, max, mean, SD: 0, 138.03, 28.978, 32.1.
5. OPS PC6 out of range. Value: -3.5726. Training min, max, SD, explained variance: -2.4321, 2.9885, 1.256, 0.0488.
6. Unknown FCFP_2 feature: 8: [*][N+](=[*])[*]
7. Unknown FCFP_2 feature: 5: [*][O-]
8. Unknown FCFP_2 feature: -1539162406: [*]C([*])[c]1:o:[*]:[*]:n:1
9. Unknown FCFP_2 feature: -828984032: [*][c](:[*]):[c]([N+](=[*])[*]):c:[*]
10. Unknown FCFP_2 feature: -1338588315: [*]:[c](:[*])[N+](=O)[O-]
11. Unknown FCFP_2 feature: 1872392852: [*][N+](=O)[*]
12. Unknown FCFP_2 feature: 260476081: [*][N+](=[*])[O-]

Feature Contribution

Top features for positive contribution

Fingerprint	Bit/Smiles	Feature Structure	Score

FCFP_2	332760439	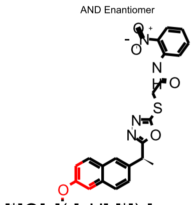 [*]O[c](:[cH]:[*]):[cH]:[*]	0.672
FCFP_2	1	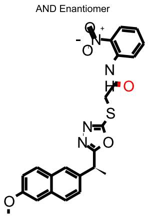 [*]=O	0.511
FCFP_2	3	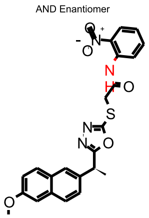 [*]N[*]	0.104
**Top Features for negative contribution**			
**Fingerprint**	**Bit/Smiles**	**Feature Structure**	**Score**
FCFP_2	136597326	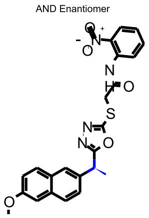 [*]C([*])C	-0.489
FCFP_2	203677720	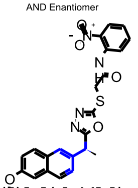 [*]C([*])[c](:[cH]:[*]):[cH]:[*]	-0.406

FCFP_2	1872154524	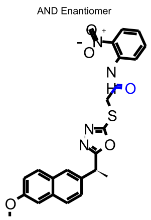 [*]C(=O)[*]	-0.307

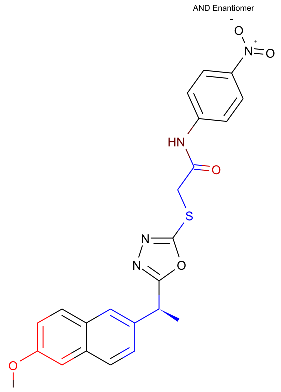

$C_{23}H_{20}N_4O_5S$
Molecular Weight: 464.4937
ALogP: 4.132
Rotatable Bonds: 8
Acceptors: 7
Donors: 1

Model Prediction

Prediction: 0.008
Unit: g/kg_body_weight
Mahalanobis Distance: 12.920
Mahalanobis Distance p-value: 4.34e-010

Mahalanobis Distance: The Mahalanobis distance (MD) is a generalization of the Euclidean distance that accounts for correlations among the X properties. It is calculated as the distance to the center of the training data. The larger the MD, the less trustworthy the prediction.
Mahalanobis Distance p-value: The p-value gives the fraction of training data with an MD greater than or equal to the one for the given sample, assuming normally distributed data. The smaller the p-value, the less trustworthy the prediciton. For highly non-normal X properties (e.g., fingerprints), the MD p-value is wildly inaccurate.

Structural Similar Compounds

Name	OCHRATOXIN	SULFISOOXAZOLE	PENICILLIN VK
Structure	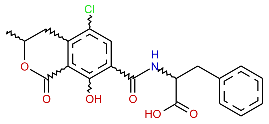	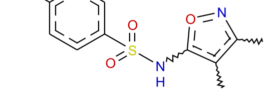	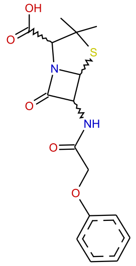
Actual Endpoint (-log C)	6.28396	2.82494	2.54455
Predicted Endpoint (-log C)	5.12358	3.0705	3.9702
Distance	1.157	1.266	1.307
Reference	NCI/NTP TR-358	NCI/NTP TR-138	NCI/NTP TR-336

Model Applicability

Unknown features are fingerprint features in the query molecule, but not found in the training set.

1. Molecular_Weight out of range. Value: 464.49. Training min, max, mean, SD: 68.074, 434.63, 171.13, 85.06.
2. Num_H_Acceptors out of range. Value: 7. Training min, max, mean, SD: 0, 6, 1.6146, 1.644.
3. Num_AromaticRings out of range. Value: 4. Training min, max, mean, SD: 0, 2, 0.5625, 0.693.
4. Molecular_PolarSurfaceArea out of range. Value: 148.37. Training min, max, mean, SD: 0, 138.03, 28.978, 32.1.
5. OPS PC6 out of range. Value: -3.5726. Training min, max, SD, explained variance: -2.4321, 2.9885, 1.256, 0.0488.
6. Unknown FCFP_2 feature: 8: [*][N+](=[*])[*]
7. Unknown FCFP_2 feature: 5: [*][O-]
8. Unknown FCFP_2 feature: -1539162406: [*]C([*])[c]1:o:[*]:[*]:n:1
9. Unknown FCFP_2 feature: -828984032: [*][c](:[*]):[c]([N+](=[*])[*]):c:[*]
10. Unknown FCFP_2 feature: -1338588315: [*]:[c](:[*])[N+](=O)[O-]
11. Unknown FCFP_2 feature: 1872392852: [*][N+](=O)[*]
12. Unknown FCFP_2 feature: 260476081: [*][N+](=[*])[O-]

Feature Contribution

Top features for positive contribution

Fingerprint	Bit/Smiles	Feature Structure	Score

FCFP_2	332760439	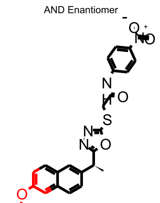 [*]O[c](:[cH]:[*]):[cH]:[*]	0.672
FCFP_2	1	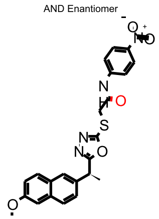 [*]=O	0.511
FCFP_2	3	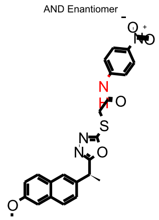 [*]N[*]	0.104
**Top Features for negative contribution**			
**Fingerprint**	**Bit/Smiles**	**Feature Structure**	**Score**
FCFP_2	136597326	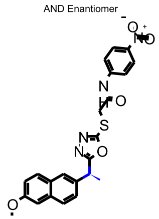 [*]C([*])C	-0.489
FCFP_2	203677720	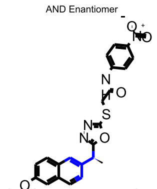 [*]C([*])[c](:[cH]:[*]):[cH]:[*]	-0.406

FCFP_2	1872154524	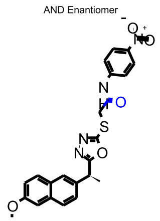<br>[*]C(=O)[*]	-0.307

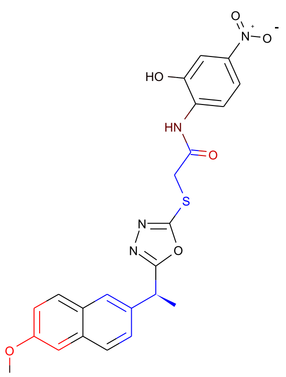

$C_{23}H_{20}N_4O_6S$
Molecular Weight: 480.4931
ALogP: 3.89
Rotatable Bonds: 8
Acceptors: 8
Donors: 2

Model Prediction

Prediction: 0.003
Unit: g/kg_body_weight
Mahalanobis Distance: 13.029
Mahalanobis Distance p-value: 3.04e-010

Mahalanobis Distance: The Mahalanobis distance (MD) is a generalization of the Euclidean distance that accounts for correlations among the X properties. It is calculated as the distance to the center of the training data. The larger the MD, the less trustworthy the prediction.
Mahalanobis Distance p-value: The p-value gives the fraction of training data with an MD greater than or equal to the one for the given sample, assuming normally distributed data. The smaller the p-value, the less trustworthy the prediciton. For highly non-normal X properties (e.g., fingerprints), the MD p-value is wildly inaccurate.

Structural Similar Compounds

Name	OCHRATOXIN	PENICILLIN VK	SULFISOOXAZOLE
Structure	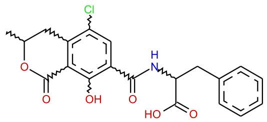	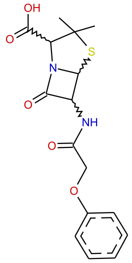	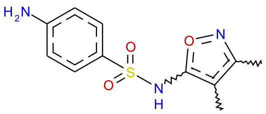
Actual Endpoint (-log C)	6.28396	2.54455	2.82494
Predicted Endpoint (-log C)	5.12358	3.9702	3.0705
Distance	1.115	1.339	1.355
Reference	NCI/NTP TR-358	NCI/NTP TR-336	NCI/NTP TR-138

Model Applicability

Unknown features are fingerprint features in the query molecule, but not found in the training set.

1. Molecular_Weight out of range. Value: 480.49. Training min, max, mean, SD: 68.074, 434.63, 171.13, 85.06.
2. Num_H_Acceptors out of range. Value: 8. Training min, max, mean, SD: 0, 6, 1.6146, 1.644.
3. Num_AromaticRings out of range. Value: 4. Training min, max, mean, SD: 0, 2, 0.5625, 0.693.
4. Molecular_PolarSASA out of range. Value: 246.84. Training min, max, mean, SD: 0, 223.97, 50.816, 55.15.
5. Molecular_PolarSurfaceArea out of range. Value: 168.6. Training min, max, mean, SD: 0, 138.03, 28.978, 32.1.
6. OPS PC1 out of range. Value: 10.247. Training min, max, SD, explained variance: -4.0008, 7.9165, 2.861, 0.2531.
7. OPS PC6 out of range. Value: -2.7251. Training min, max, SD, explained variance: -2.4321, 2.9885, 1.256, 0.0488.
8. OPS PC9 out of range. Value: 3.0427. Training min, max, SD, explained variance: -2.7086, 2.9267, 1.019, 0.0321.
9. Unknown FCFP_2 feature: 8: [*][N+](=[*])[*]
10. Unknown FCFP_2 feature: 5: [*][O-]
11. Unknown FCFP_2 feature: -1539162406: [*]C([*])[c]1:o:[*]:[*]:n:1
12. Unknown FCFP_2 feature: -828984032: [*][c](:[*]):[c]([N+](=[*])[*]):c:[*]
13. Unknown FCFP_2 feature: -1338588315: [*]:[c](:[*])[N+](=O)[O-]
14. Unknown FCFP_2 feature: 1872392852: [*][N+](=O)[*]
15. Unknown FCFP_2 feature: 260476081: [*][N+](=[*])[O-]

Feature Contribution

Top features for positive contribution			
**Fingerprint**	**Bit/Smiles**	**Feature Structure**	**Score**
FCFP_2	332760439	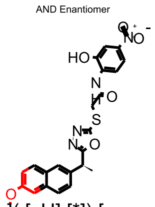 [*]O[c](:[cH]:[*]):[cH]:[*]	0.672
FCFP_2	1	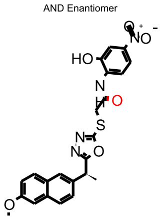 [*]=O	0.511
FCFP_2	3	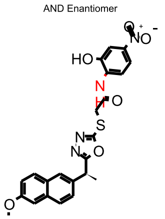 [*]N[*]	0.104

Top Features for negative contribution			
**Fingerprint**	**Bit/Smiles**	**Feature Structure**	**Score**
FCFP_2	136597326	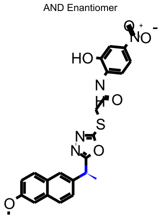 [*]C([*])C	-0.489

FCFP_2	203677720	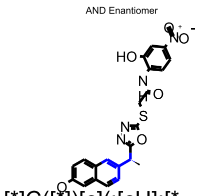 [*]C([*])[c](:[cH]:[*]):[cH]:[*]	-0.406
FCFP_2	1872154524	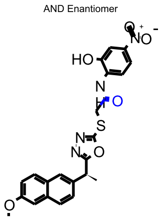 [*]C(=O)[*]	-0.307

Sorafenib

TOPKAT_Rat_Maximum_Tolerated_Dose_Gavage

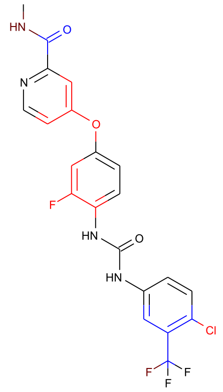

$C_{21}H_{15}ClF_4N_4O_3$
Molecular Weight: 482.81541
ALogP: 4.381
Rotatable Bonds: 6
Acceptors: 4
Donors: 3

Model Prediction

Prediction: 0.001
Unit: g/kg_body_weight
Mahalanobis Distance: 12.177
Mahalanobis Distance p-value: 4.95e-009

Mahalanobis Distance: The Mahalanobis distance (MD) is a generalization of the Euclidean distance that accounts for correlations among the X properties. It is calculated as the distance to the center of the training data. The larger the MD, the less trustworthy the prediction.
Mahalanobis Distance p-value: The p-value gives the fraction of training data with an MD greater than or equal to the one for the given sample, assuming normally distributed data. The smaller the p-value, the less trustworthy the prediciton. For highly non-normal X properties (e.g., fingerprints), the MD p-value is wildly inaccurate.

Structural Similar Compounds

Name	OCHRATOXIN	SULFISOOXAZOLE	PENICILLIN VK
Structure	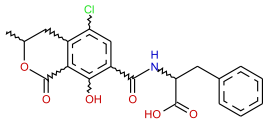	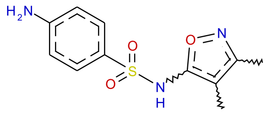	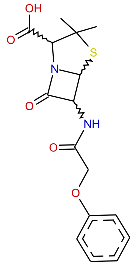
Actual Endpoint (-log C)	6.28396	2.82494	2.54455
Predicted Endpoint (-log C)	5.12358	3.0705	3.9702
Distance	0.776	1.031	1.182
Reference	NCI/NTP TR-358	NCI/NTP TR-138	NCI/NTP TR-336

Model Applicability

Unknown features are fingerprint features in the query molecule, but not found in the training set.

1. Molecular_Weight out of range. Value: 482.82. Training min, max, mean, SD: 68.074, 434.63, 171.13, 85.06.
2. Num_AromaticRings out of range. Value: 3. Training min, max, mean, SD: 0, 2, 0.5625, 0.693.
3. OPS PC5 out of range. Value: -3.5956. Training min, max, SD, explained variance: -3.4, 4.1587, 1.489, 0.0686.
4. OPS PC7 out of range. Value: -3.867. Training min, max, SD, explained variance: -2.8003, 2.9332, 1.16, 0.0416.
5. Unknown FCFP_2 feature: 136686699: [*]NC
6. Unknown FCFP_2 feature: 1499521844: [*]NC(=O)N[*]
7. Unknown FCFP_2 feature: -1029533685: [*]:[c](:[*])C(F)(F)F

Feature Contribution

Top features for positive contribution

Fingerprint	Bit/Smiles	Feature Structure	Score

FCFP_2	332760439	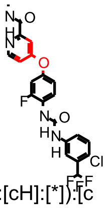 [*]O[c](:[cH]:[*]):[cH]:[*]	0.672
FCFP_2	32	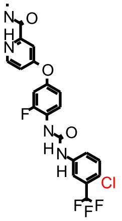 [*]Cl	0.526
FCFP_2	1	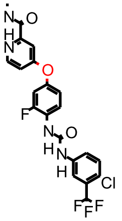 [*]=O	0.511

Top Features for negative contribution

Fingerprint	Bit/Smiles	Feature Structure	Score
FCFP_2	203677720	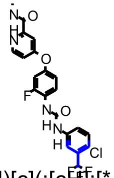 [*]C([*])[c](:[cH]:[*]):[cH]:[*]	-0.406
FCFP_2	1872154524	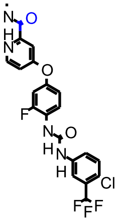 [*]C(=O)[*]	-0.307

FCFP_2	0	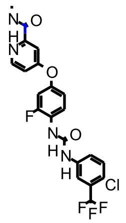 [*]C[*]	-0.290

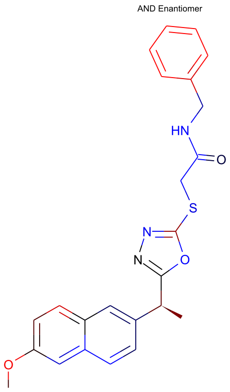

$C_{24}H_{23}N_3O_3S$
Molecular Weight: 433.52272
ALogP: 4.245
Rotatable Bonds: 8
Acceptors: 5
Donors: 1

Model Prediction

Prediction: 0.700
Unit: g/kg_body_weight
Mahalanobis Distance: 23.957
Mahalanobis Distance p-value: 1.77e-025

Mahalanobis Distance: The Mahalanobis distance (MD) is a generalization of the Euclidean distance that accounts for correlations among the X properties. It is calculated as the distance to the center of the training data. The larger the MD, the less trustworthy the prediction.
Mahalanobis Distance p-value: The p-value gives the fraction of training data with an MD greater than or equal to the one for the given sample, assuming normally distributed data. The smaller the p-value, the less trustworthy the prediciton. For highly non-normal X properties (e.g., fingerprints), the MD p-value is wildly inaccurate.

Structural Similar Compounds

Name	ACEMETACIN	bis-OXATIN ACETATE	TALNIFLUMATE
Structure	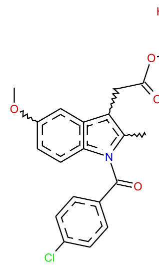	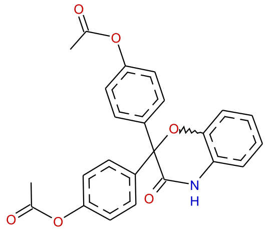	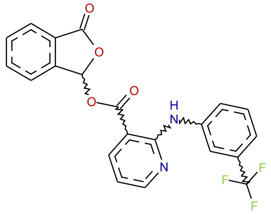
Actual Endpoint (-log C)	4.235	1.717	1.538
Predicted Endpoint (-log C)	3.39415	2.40947	2.82541
Distance	0.625	0.657	0.706
Reference	ARZNAD 30;1398;80	NIIRDN 6;609;82	FRPSAX 36;372;81

Model Applicability

Unknown features are fingerprint features in the query molecule, but not found in the training set.

1. All properties and OPS components are within expected ranges.
2. Unknown ECFP_2 feature: 1093109320: [*]S[c]1:o:[*]:[*]:n:1
3. Unknown ECFP_2 feature: 1092541557: [*]C([*])[c]1:o:[*]:[*]:n:1
4. Unknown FCFP_6 feature: 16: [*][c](:[*]):[*]
5. Unknown FCFP_6 feature: 907096426: [*]NC[c](:[*]):[*]
6. Unknown FCFP_6 feature: 1747237384: [*][c]1:[*]:[*]:[c]([*]):o:1
7. Unknown FCFP_6 feature: -1410079687: [*]S[c]1:o:[*]:[*]:n:1
8. Unknown FCFP_6 feature: 4427049: [*][c]1:[*]:[*]:n:n:1
9. Unknown FCFP_6 feature: 1618154665: [*][c](:[*]):[cH]:[cH]:[*]
10. Unknown FCFP_6 feature: -928857652: [*]:[c](:[*])C(C)[c](:[*]):[*]
11. Unknown FCFP_6 feature: -1539162406: [*]C([*])[c]1:o:[*]:[*]:n:1

Feature Contribution

Top features for positive contribution			
**Fingerprint**	**Bit/Smiles**	**Feature Structure**	**Score**

ECFP_6	642810091	AND Enantiomer 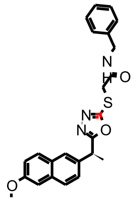 [*][c](:[*]):[*]	0.281
ECFP_6	-1897341097	AND Enantiomer 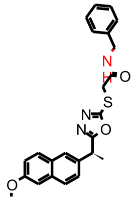 [*]N[*]	0.216
ECFP_6	1571214559	AND Enantiomer 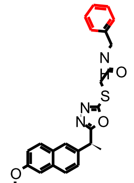 [*]1:[cH]:[cH]:[cH]:[cH]:[cH]:1	0.190

Top Features for negative contribution

Fingerprint	Bit/Smiles	Feature Structure	Score
ECFP_6	497523368	AND Enantiomer 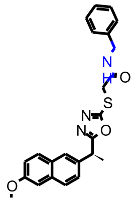 [*]CNC(=[*])[*]	-0.301
ECFP_6	683445015	AND Enantiomer 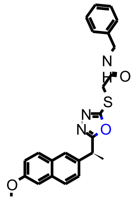 [*]:o:[*]	-0.266

ECFP_6	-176455838	AND Enantiomer 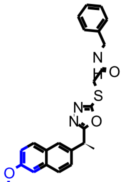 [*]O[c](:[cH]:[*]):[cH]:[*]	-0.257

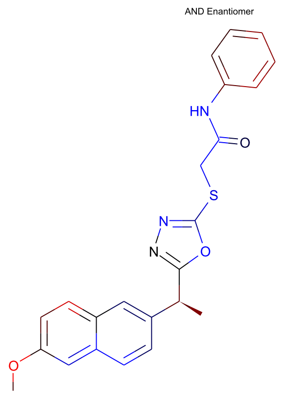

$C_{23}H_{21}N_3O_3S$
Molecular Weight: 419.49614
ALogP: 4.238
Rotatable Bonds: 7
Acceptors: 5
Donors: 1

Model Prediction

Prediction: 0.876
Unit: g/kg_body_weight
Mahalanobis Distance: 23.919
Mahalanobis Distance p-value: 2.78e-025

Mahalanobis Distance: The Mahalanobis distance (MD) is a generalization of the Euclidean distance that accounts for correlations among the X properties. It is calculated as the distance to the center of the training data. The larger the MD, the less trustworthy the prediction.
Mahalanobis Distance p-value: The p-value gives the fraction of training data with an MD greater than or equal to the one for the given sample, assuming normally distributed data. The smaller the p-value, the less trustworthy the prediciton. For highly non-normal X properties (e.g., fingerprints), the MD p-value is wildly inaccurate.

Structural Similar Compounds

Name	ACEMETACIN	bis-OXATIN ACETATE	TALNIFLUMATE
Structure	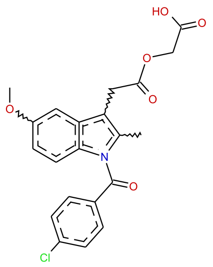	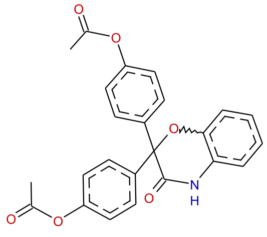	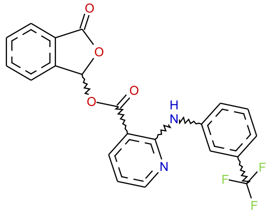
Actual Endpoint (-log C)	4.235	1.717	1.538
Predicted Endpoint (-log C)	3.39415	2.40947	2.82541
Distance	0.619	0.624	0.678
Reference	ARZNAD 30;1398;80	NIIRDN 6;609;82	FRPSAX 36;372;81

Model Applicability

Unknown features are fingerprint features in the query molecule, but not found in the training set.

1. All properties and OPS components are within expected ranges.
2. Unknown ECFP_2 feature: 1093109320: [*]S[c]1:o:[*]:[*]:n:1
3. Unknown ECFP_2 feature: 1092541557: [*]C([*])[c]1:o:[*]:[*]:n:1
4. Unknown FCFP_6 feature: 16: [*][c](:[*]):[*]
5. Unknown FCFP_6 feature: 1747237384: [*][c]1:[*]:[*]:[c]([*]):o:1
6. Unknown FCFP_6 feature: -1410079687: [*]S[c]1:o:[*]:[*]:n:1
7. Unknown FCFP_6 feature: 4427049: [*][c]1:[*]:[*]:n:n:1
8. Unknown FCFP_6 feature: 1618154665: [*][c](:[*]):[cH]:[cH]:[*]
9. Unknown FCFP_6 feature: -928857652: [*]:[c](:[*])C(C)[c](:[*]):[*]
10. Unknown FCFP_6 feature: -1539162406: [*]C([*])[c]1:o:[*]:[*]:n:1

Feature Contribution

Top features for positive contribution

Fingerprint	Bit/Smiles	Feature Structure	Score

ECFP_6	642810091	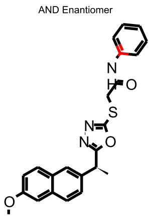 [*][c](:[*]):[*]	0.281
ECFP_6	-1897341097	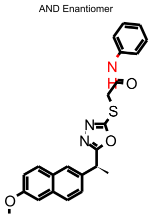 [*]N[*]	0.216
ECFP_6	1571214559	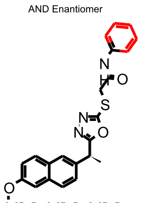 [*]1:[cH]:[cH]:[cH]:[cH]:[cH]:1	0.190

Top Features for negative contribution

Fingerprint	Bit/Smiles	Feature Structure	Score
ECFP_6	683445015	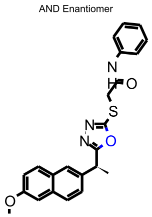 [*]:o:[*]	-0.266
ECFP_6	-176455838	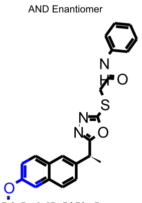 [*]O[c](:[cH]:[*]):[cH]:[*]	-0.257

ECFP_6	655739385	AND Enantiomer<br>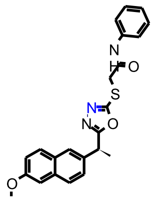<br>[*]:n:[*]	-0.239

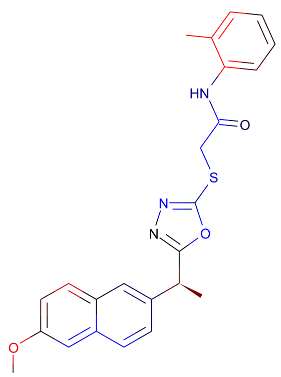

$C_{24}H_{23}N_3O_3S$
Molecular Weight: 433.52272
ALogP: 4.724
Rotatable Bonds: 7
Acceptors: 5
Donors: 1

Model Prediction

Prediction: 0.386
Unit: g/kg_body_weight
Mahalanobis Distance: 24.099
Mahalanobis Distance p-value: 3.22e-026

Mahalanobis Distance: The Mahalanobis distance (MD) is a generalization of the Euclidean distance that accounts for correlations among the X properties. It is calculated as the distance to the center of the training data. The larger the MD, the less trustworthy the prediction.
Mahalanobis Distance p-value: The p-value gives the fraction of training data with an MD greater than or equal to the one for the given sample, assuming normally distributed data. The smaller the p-value, the less trustworthy the prediciton. For highly non-normal X properties (e.g., fingerprints), the MD p-value is wildly inaccurate.

Structural Similar Compounds

Name	ACEMETACIN	bis-OXATIN ACETATE	TALNIFLUMATE
Structure	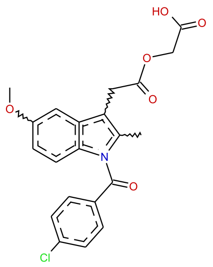	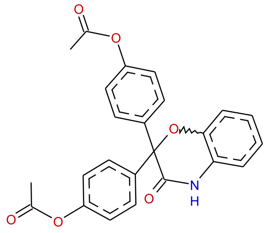	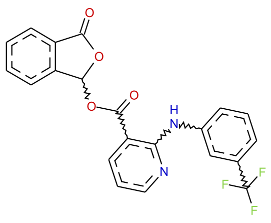
Actual Endpoint (-log C)	4.235	1.717	1.538
Predicted Endpoint (-log C)	3.39415	2.40947	2.82541
Distance	0.625	0.641	0.668
Reference	ARZNAD 30;1398;80	NIIRDN 6;609;82	FRPSAX 36;372;81

Model Applicability

Unknown features are fingerprint features in the query molecule, but not found in the training set.

1. All properties and OPS components are within expected ranges.
2. Unknown ECFP_2 feature: 1093109320: [*]S[c]1:o:[*]:[*]:n:1
3. Unknown ECFP_2 feature: 1092541557: [*]C([*])[c]1:o:[*]:[*]:n:1
4. Unknown FCFP_6 feature: 16: [*][c](:[*]):[*]
5. Unknown FCFP_6 feature: 1747237384: [*][c]1:[*]:[*]:[c]([*]):o:1
6. Unknown FCFP_6 feature: -1410079687: [*]S[c]1:o:[*]:[*]:n:1
7. Unknown FCFP_6 feature: 4427049: [*][c]1:[*]:[*]:n:n:1
8. Unknown FCFP_6 feature: 1618154665: [*][c](:[*]):[cH]:[cH]:[*]
9. Unknown FCFP_6 feature: -928857652: [*]:[c](:[*])C(C)[c](:[*]):[*]
10. Unknown FCFP_6 feature: -1539162406: [*]C([*])[c]1:o:[*]:[*]:n:1

Feature Contribution

Top features for positive contribution

Fingerprint	Bit/Smiles	Feature Structure	Score

ECFP_6	642810091	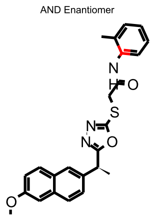 [*][c](:[*]):[*]	0.281
ECFP_6	2147419938	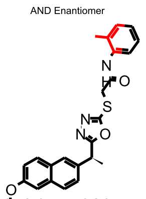 [*][c](:[*]):[c](C):[cH]:[*]	0.263
ECFP_6	-1897341097	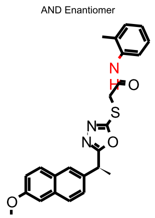 [*]N[*]	0.216

Top Features for negative contribution

**Fingerprint**	**Bit/Smiles**	**Feature Structure**	**Score**
ECFP_6	683445015	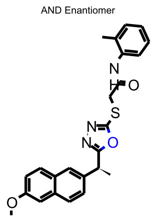 [*]:o:[*]	-0.266
ECFP_6	-176455838	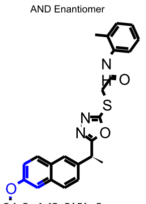 [*]O[c](:[cH]:[*]):[c H]:[*]	-0.257

ECFP_6	655739385	AND Enantiomer 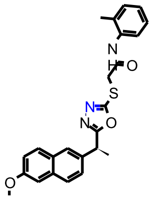 [*]:n:[*]	-0.239

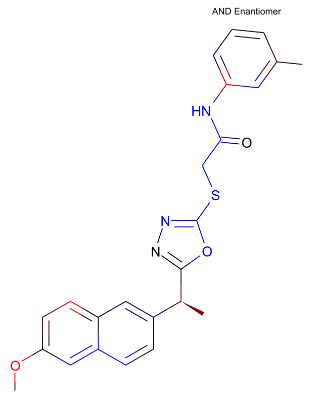

$C_{24}H_{23}N_3O_3S$

Molecular Weight: 433.52272

ALogP: 4.724

Rotatable Bonds: 7

Acceptors: 5

Donors: 1

Model Prediction

Prediction: 0.991

Unit: g/kg_body_weight

Mahalanobis Distance: 23.877

Mahalanobis Distance p-value: 4.58e-025

Mahalanobis Distance: The Mahalanobis distance (MD) is a generalization of the Euclidean distance that accounts for correlations among the X properties. It is calculated as the distance to the center of the training data. The larger the MD, the less trustworthy the prediction.

Mahalanobis Distance p-value: The p-value gives the fraction of training data with an MD greater than or equal to the one for the given sample, assuming normally distributed data. The smaller the p-value, the less trustworthy the prediciton. For highly non-normal X properties (e.g., fingerprints), the MD p-value is wildly inaccurate.

Structural Similar Compounds

Name	ACEMETACIN	bis-OXATIN ACETATE	TALNIFLUMATE
Structure	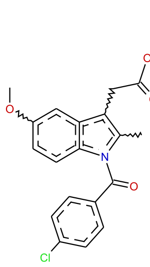	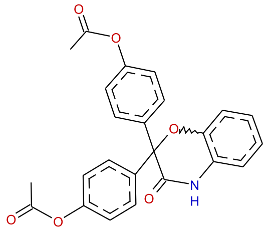	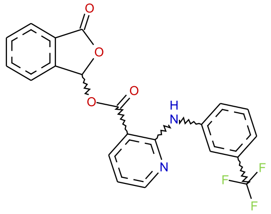
Actual Endpoint (-log C)	4.235	1.717	1.538
Predicted Endpoint (-log C)	3.39415	2.40947	2.82541
Distance	0.630	0.650	0.671
Reference	ARZNAD 30;1398;80	NIIRDN 6;609;82	FRPSAX 36;372;81

Model Applicability

Unknown features are fingerprint features in the query molecule, but not found in the training set.

1. All properties and OPS components are within expected ranges.
2. Unknown ECFP_2 feature: 1093109320: [*]S[c]1:o:[*]:[*]:n:1
3. Unknown ECFP_2 feature: 1092541557: [*]C([*])[c]1:o:[*]:[*]:n:1
4. Unknown FCFP_6 feature: 16: [*][c](:[*]):[*]
5. Unknown FCFP_6 feature: 1747237384: [*][c]1:[*]:[*]:[c]([*]):o:1
6. Unknown FCFP_6 feature: -1410079687: [*]S[c]1:o:[*]:[*]:n:1
7. Unknown FCFP_6 feature: 4427049: [*][c]1:[*]:[*]:n:n:1
8. Unknown FCFP_6 feature: 1618154665: [*][c](:[*]):[cH]:[cH]:[*]
9. Unknown FCFP_6 feature: -928857652: [*]:[c](:[*])C(C)[c](:[*]):[*]
10. Unknown FCFP_6 feature: -1539162406: [*]C([*])[c]1:o:[*]:[*]:n:1

Feature Contribution

Top features for positive contribution

Fingerprint	Bit/Smiles	Feature Structure	Score

ECFP_6	642810091	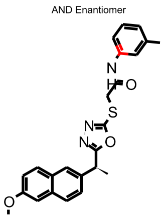<br>[*][c](:[*]):[*]	0.281
ECFP_6	-1897341097	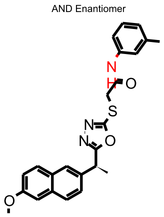<br>[*]N[*]	0.216
FCFP_6	136627117	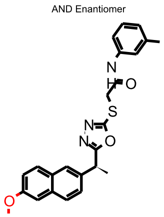<br>[*]OC	0.170

Top Features for negative contribution

Fingerprint	Bit/Smiles	Feature Structure	Score
ECFP_6	683445015	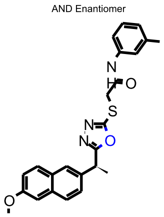<br>[*]:o:[*]	-0.266
ECFP_6	-176455838	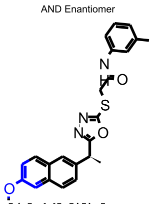<br>[*]O[c](:[cH]:[*]):[cH]:[*]	-0.257

ECFP_6	655739385	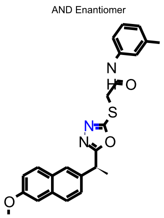<br>[*]:n:[*]	-0.239

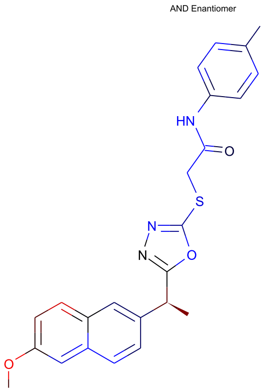

$C_{24}H_{23}N_3O_3S$
Molecular Weight: 433.52272
ALogP: 4.724
Rotatable Bonds: 7
Acceptors: 5
Donors: 1

Model Prediction

Prediction: 1.412
Unit: g/kg_body_weight
Mahalanobis Distance: 23.877
Mahalanobis Distance p-value: 4.58e-025

Mahalanobis Distance: The Mahalanobis distance (MD) is a generalization of the Euclidean distance that accounts for correlations among the X properties. It is calculated as the distance to the center of the training data. The larger the MD, the less trustworthy the prediction.
Mahalanobis Distance p-value: The p-value gives the fraction of training data with an MD greater than or equal to the one for the given sample, assuming normally distributed data. The smaller the p-value, the less trustworthy the prediciton. For highly non-normal X properties (e.g., fingerprints), the MD p-value is wildly inaccurate.

Structural Similar Compounds

Name	ACEMETACIN	bis-OXATIN ACETATE	TALNIFLUMATE
Structure	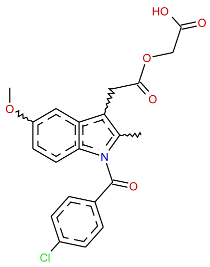	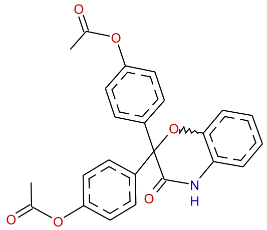	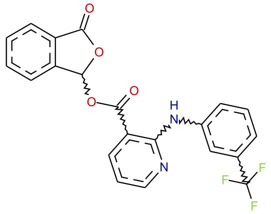
Actual Endpoint (-log C)	4.235	1.717	1.538
Predicted Endpoint (-log C)	3.39415	2.40947	2.82541
Distance	0.629	0.652	0.677
Reference	ARZNAD 30;1398;80	NIIRDN 6;609;82	FRPSAX 36;372;81

Model Applicability

Unknown features are fingerprint features in the query molecule, but not found in the training set.

1. All properties and OPS components are within expected ranges.
2. Unknown ECFP_2 feature: 1093109320: [*]S[c]1:o:[*]:[*]:n:1
3. Unknown ECFP_2 feature: 1092541557: [*]C([*])[c]1:o:[*]:[*]:n:1
4. Unknown FCFP_6 feature: 16: [*][c](:[*]):[*]
5. Unknown FCFP_6 feature: 1747237384: [*][c]1:[*]:[*]:[c]([*]):o:1
6. Unknown FCFP_6 feature: -1410079687: [*]S[c]1:o:[*]:[*]:n:1
7. Unknown FCFP_6 feature: 4427049: [*][c]1:[*]:[*]:n:n:1
8. Unknown FCFP_6 feature: 1618154665: [*][c](:[*]):[cH]:[cH]:[*]
9. Unknown FCFP_6 feature: -928857652: [*]:[c](:[*])C(C)[c](:[*]):[*]
10. Unknown FCFP_6 feature: -1539162406: [*]C([*])[c]1:o:[*]:[*]:n:1

Feature Contribution

Top features for positive contribution

Fingerprint	Bit/Smiles	Feature Structure	Score

ECFP_6	642810091	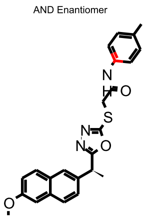 [*][c](:[*]):[*]	0.281
ECFP_6	-1897341097	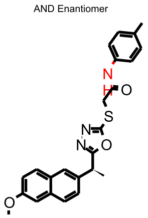 [*]N[*]	0.216
FCFP_6	136627117	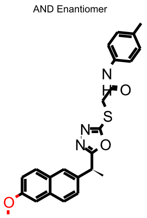 [*]OC	0.170
**Top Features for negative contribution**			
**Fingerprint**	**Bit/Smiles**	**Feature Structure**	**Score**
ECFP_6	683445015	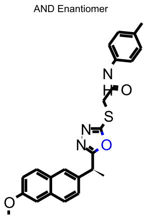 [*]:o:[*]	-0.266
ECFP_6	-176455838	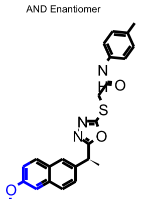 [*]O[c](:[cH]:[*]):[cH]:[*]	-0.257

ECFP_6	655739385	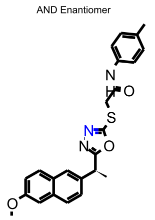 [*]:n:[*]	-0.239

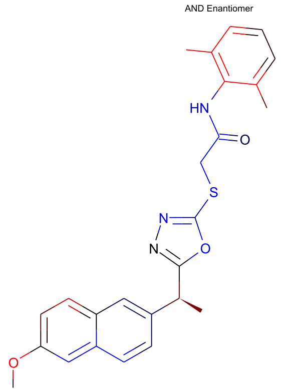

$C_{25}H_{25}N_3O_3S$
Molecular Weight: 447.5493
ALogP: 5.21
Rotatable Bonds: 7
Acceptors: 5
Donors: 1

Model Prediction

Prediction: 0.368
Unit: g/kg_body_weight
Mahalanobis Distance: 24.053
Mahalanobis Distance p-value: 5.63e-026

Mahalanobis Distance: The Mahalanobis distance (MD) is a generalization of the Euclidean distance that accounts for correlations among the X properties. It is calculated as the distance to the center of the training data. The larger the MD, the less trustworthy the prediction.
Mahalanobis Distance p-value: The p-value gives the fraction of training data with an MD greater than or equal to the one for the given sample, assuming normally distributed data. The smaller the p-value, the less trustworthy the prediciton. For highly non-normal X properties (e.g., fingerprints), the MD p-value is wildly inaccurate.

Structural Similar Compounds

Name	ACEMETACIN	TALNIFLUMATE	bis-OXATIN ACETATE
Structure	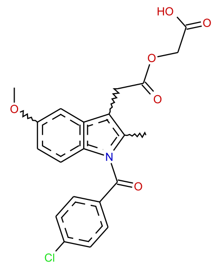	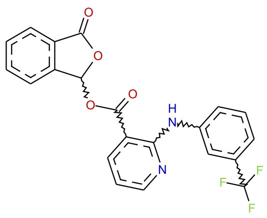	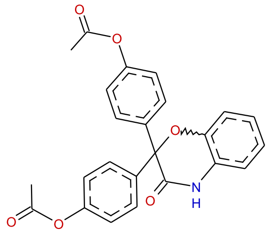
Actual Endpoint (-log C)	4.235	1.538	1.717
Predicted Endpoint (-log C)	3.39415	2.82541	2.40947
Distance	0.647	0.674	0.675
Reference	ARZNAD 30;1398;80	FRPSAX 36;372;81	NIIRDN 6;609;82

Model Applicability

Unknown features are fingerprint features in the query molecule, but not found in the training set.

1. All properties and OPS components are within expected ranges.
2. Unknown ECFP_2 feature: 1093109320: [*]S[c]1:o:[*]:[*]:n:1
3. Unknown ECFP_2 feature: 1092541557: [*]C([*])[c]1:o:[*]:[*]:n:1
4. Unknown FCFP_6 feature: 16: [*][c](:[*]):[*]
5. Unknown FCFP_6 feature: 1747237384: [*][c]1:[*]:[*]:[c]([*]):o:1
6. Unknown FCFP_6 feature: -1410079687: [*]S[c]1:o:[*]:[*]:n:1
7. Unknown FCFP_6 feature: 4427049: [*][c]1:[*]:[*]:n:n:1
8. Unknown FCFP_6 feature: 1618154665: [*][c](:[*]):[cH]:[cH]:[*]
9. Unknown FCFP_6 feature: -928857652: [*]:[c](:[*])C(C)[c](:[*]):[*]
10. Unknown FCFP_6 feature: -1539162406: [*]C([*])[c]1:o:[*]:[*]:n:1

Feature Contribution

Top features for positive contribution

Fingerprint	Bit/Smiles	Feature Structure	Score

ECFP_6	642810091	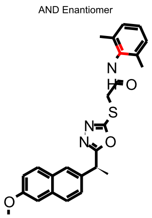 [*][c](:[*]):[*]	0.281
ECFP_6	2147419938	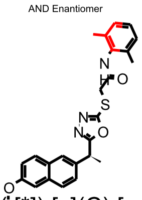 [*][c](:[*]):[c](C):[cH]:[*]	0.263
ECFP_6	-1897341097	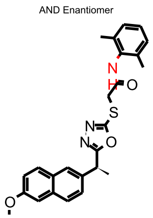 [*]N[*]	0.216
**Top Features for negative contribution**			
**Fingerprint**	**Bit/Smiles**	**Feature Structure**	**Score**
ECFP_6	683445015	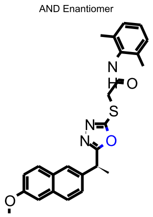 [*]:o:[*]	-0.266
ECFP_6	-176455838	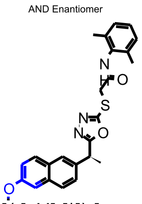 [*]O[c](:[cH]:[*]):[c H]:[*]	-0.257

ECFP_6	655739385	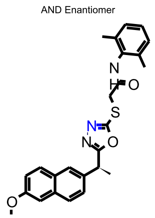 [*]:n:[*]	-0.239

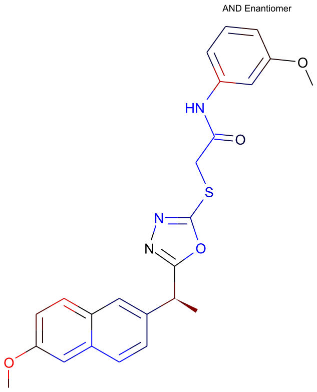

$C_{24}H_{23}N_3O_4S$
Molecular Weight: 449.52212
ALogP: 4.221
Rotatable Bonds: 8
Acceptors: 6
Donors: 1

Model Prediction

Prediction: 1.614
Unit: g/kg_body_weight
Mahalanobis Distance: 23.936
Mahalanobis Distance p-value: 2.28e-025

Mahalanobis Distance: The Mahalanobis distance (MD) is a generalization of the Euclidean distance that accounts for correlations among the X properties. It is calculated as the distance to the center of the training data. The larger the MD, the less trustworthy the prediction.
Mahalanobis Distance p-value: The p-value gives the fraction of training data with an MD greater than or equal to the one for the given sample, assuming normally distributed data. The smaller the p-value, the less trustworthy the prediciton. For highly non-normal X properties (e.g., fingerprints), the MD p-value is wildly inaccurate.

Structural Similar Compounds

Name	ACEMETACIN	bis-OXATIN ACETATE	TALNIFLUMATE
Structure	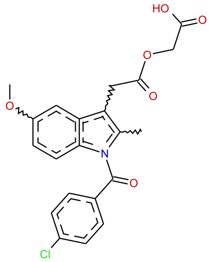	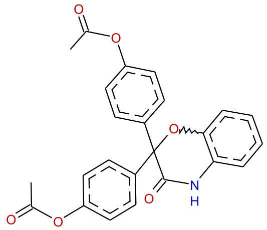	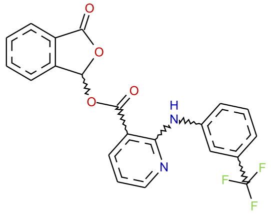
Actual Endpoint (-log C)	4.235	1.717	1.538
Predicted Endpoint (-log C)	3.39415	2.40947	2.82541
Distance	0.623	0.650	0.709
Reference	ARZNAD 30;1398;80	NIIRDN 6;609;82	FRPSAX 36;372;81

Model Applicability

Unknown features are fingerprint features in the query molecule, but not found in the training set.

1. All properties and OPS components are within expected ranges.
2. Unknown ECFP_2 feature: 1093109320: [*]S[c]1:o:[*]:[*]:n:1
3. Unknown ECFP_2 feature: 1092541557: [*]C([*])[c]1:o:[*]:[*]:n:1
4. Unknown FCFP_6 feature: 16: [*][c](:[*]):[*]
5. Unknown FCFP_6 feature: 1747237384: [*][c]1:[*]:[*]:[c]([*]):o:1
6. Unknown FCFP_6 feature: -1410079687: [*]S[c]1:o:[*]:[*]:n:1
7. Unknown FCFP_6 feature: 4427049: [*][c]1:[*]:[*]:n:n:1
8. Unknown FCFP_6 feature: 1618154665: [*][c](:[*]):[cH]:[cH]:[*]
9. Unknown FCFP_6 feature: -928857652: [*]:[c](:[*])C(C)[c](:[*]):[*]
10. Unknown FCFP_6 feature: -1539162406: [*]C([*])[c]1:o:[*]:[*]:n:1

Feature Contribution

Top features for positive contribution			
Fingerprint	Bit/Smiles	Feature Structure	Score

ECFP_6	642810091	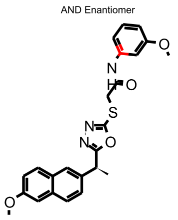 [*][c](:[*]):[*]	0.281
ECFP_6	-1897341097	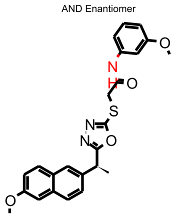 [*]N[*]	0.216
FCFP_6	136627117	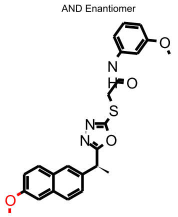 [*]OC	0.170

Top Features for negative contribution

Fingerprint	Bit/Smiles	Feature Structure	Score
ECFP_6	683445015	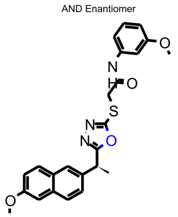 [*]:o:[*]	-0.266
ECFP_6	-176455838	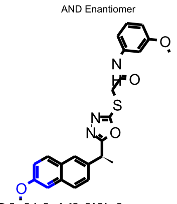 [*]O[c](:[cH]:[*]):[cH]:[*]	-0.257

ECFP_6	655739385	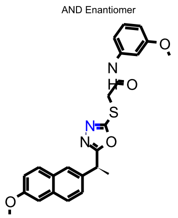 [*]:n:[*]	-0.239

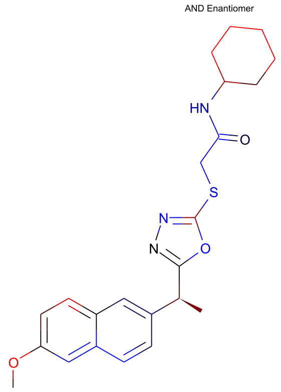

$C_{23}H_{27}N_3O_3S$
Molecular Weight: 425.54378
ALogP: 4.52
Rotatable Bonds: 7
Acceptors: 5
Donors: 1

Model Prediction

Prediction: 0.246
Unit: g/kg_body_weight
Mahalanobis Distance: 24.042
Mahalanobis Distance p-value: 6.37e-026

Mahalanobis Distance: The Mahalanobis distance (MD) is a generalization of the Euclidean distance that accounts for correlations among the X properties. It is calculated as the distance to the center of the training data. The larger the MD, the less trustworthy the prediction.
Mahalanobis Distance p-value: The p-value gives the fraction of training data with an MD greater than or equal to the one for the given sample, assuming normally distributed data. The smaller the p-value, the less trustworthy the prediciton. For highly non-normal X properties (e.g., fingerprints), the MD p-value is wildly inaccurate.

Structural Similar Compounds

Name	ACEMETACIN	bis-OXATIN ACETATE	TALNIFLUMATE
Structure	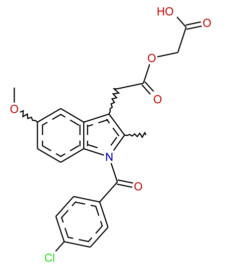	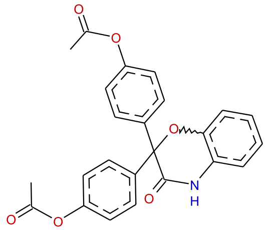	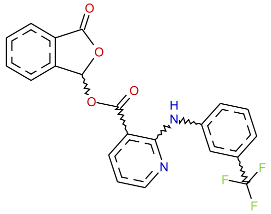
Actual Endpoint (-log C)	4.235	1.717	1.538
Predicted Endpoint (-log C)	3.39415	2.40947	2.82541
Distance	0.505	0.551	0.590
Reference	ARZNAD 30;1398;80	NIIRDN 6;609;82	FRPSAX 36;372;81

Model Applicability

Unknown features are fingerprint features in the query molecule, but not found in the training set.

1. All properties and OPS components are within expected ranges.
2. Unknown ECFP_2 feature: 1093109320: [*]S[c]1:o:[*]:[*]:n:1
3. Unknown ECFP_2 feature: 1092541557: [*]C([*])[c]1:o:[*]:[*]:n:1
4. Unknown FCFP_6 feature: 16: [*][c](:[*]):[*]
5. Unknown FCFP_6 feature: 1747237384: [*][c]1:[*]:[*]:[c]([*]):o:1
6. Unknown FCFP_6 feature: -1410079687: [*]S[c]1:o:[*]:[*]:n:1
7. Unknown FCFP_6 feature: 4427049: [*][c]1:[*]:[*]:n:n:1
8. Unknown FCFP_6 feature: 1618154665: [*][c](:[*]):[cH]:[cH]:[*]
9. Unknown FCFP_6 feature: -928857652: [*]:[c](:[*])C(C)[c](:[*]):[*]
10. Unknown FCFP_6 feature: -1539162406: [*]C([*])[c]1:o:[*]:[*]:n:1

Feature Contribution

Top features for positive contribution			
**Fingerprint**	**Bit/Smiles**	**Feature Structure**	**Score**

ECFP_6	642810091	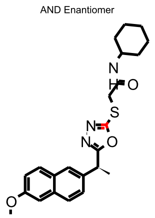 [*][c](:[*]):[*]	0.281
ECFP_6	-1897341097	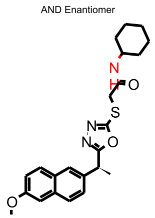 [*]N[*]	0.216
FCFP_6	136627117	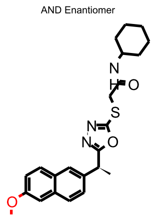 [*]OC	0.170
**Top Features for negative contribution**			
**Fingerprint**	**Bit/Smiles**	**Feature Structure**	**Score**
ECFP_6	683445015	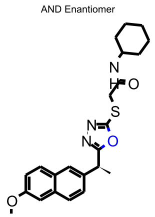 [*]:o:[*]	-0.266
ECFP_6	-176455838	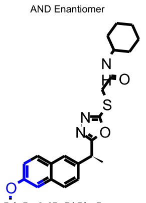 [*]O[c](:[cH]:[*]):[cH]:[*]	-0.257

ECFP_6	655739385	AND Enantiomer 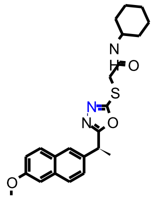 [*]:n:[*]	-0.239

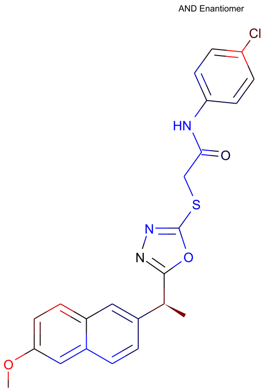

$C_{23}H_{20}ClN_3O_3S$
Molecular Weight: 453.9412
ALogP: 4.902
Rotatable Bonds: 7
Acceptors: 5
Donors: 1

Model Prediction

Prediction: 0.915
Unit: g/kg_body_weight
Mahalanobis Distance: 24.298
Mahalanobis Distance p-value: 2.9e-027

Mahalanobis Distance: The Mahalanobis distance (MD) is a generalization of the Euclidean distance that accounts for correlations among the X properties. It is calculated as the distance to the center of the training data. The larger the MD, the less trustworthy the prediction.
Mahalanobis Distance p-value: The p-value gives the fraction of training data with an MD greater than or equal to the one for the given sample, assuming normally distributed data. The smaller the p-value, the less trustworthy the prediciton. For highly non-normal X properties (e.g., fingerprints), the MD p-value is wildly inaccurate.

Structural Similar Compounds

Name	ACEMETACIN	bis-OXATIN ACETATE	TALNIFLUMATE
Structure	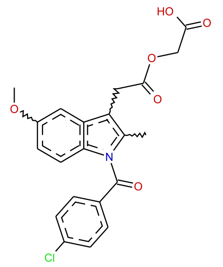	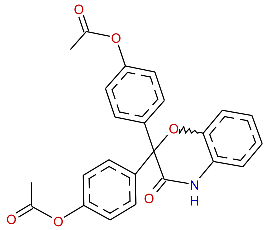	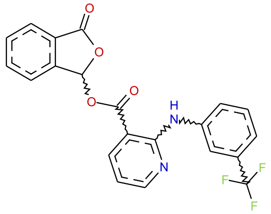
Actual Endpoint (-log C)	4.235	1.717	1.538
Predicted Endpoint (-log C)	3.39415	2.40947	2.82541
Distance	0.617	0.673	0.679
Reference	ARZNAD 30;1398;80	NIIRDN 6;609;82	FRPSAX 36;372;81

Model Applicability

Unknown features are fingerprint features in the query molecule, but not found in the training set.

1. All properties and OPS components are within expected ranges.
2. Unknown ECFP_2 feature: 1093109320: [*]S[c]1:o:[*]:[*]:n:1
3. Unknown ECFP_2 feature: 1092541557: [*]C([*])[c]1:o:[*]:[*]:n:1
4. Unknown FCFP_6 feature: 16: [*][c](:[*]):[*]
5. Unknown FCFP_6 feature: 1747237384: [*][c]1:[*]:[*]:[c]([*]):o:1
6. Unknown FCFP_6 feature: -1410079687: [*]S[c]1:o:[*]:[*]:n:1
7. Unknown FCFP_6 feature: 4427049: [*][c]1:[*]:[*]:n:n:1
8. Unknown FCFP_6 feature: 1618154665: [*][c](:[*]):[cH]:[cH]:[*]
9. Unknown FCFP_6 feature: -928857652: [*]:[c](:[*])C(C)[c](:[*]):[*]
10. Unknown FCFP_6 feature: -1539162406: [*]C([*])[c]1:o:[*]:[*]:n:1
11. Unknown FCFP_6 feature: 71476542: [*]:[c](:[*])Cl

Feature Contribution

Top features for positive contribution

Fingerprint	Bit/Smiles	Feature Structure	Score

ECFP_6	642810091	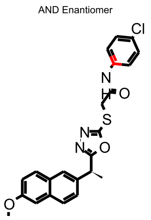 [*][c](:[*]):[*]	0.281
ECFP_6	-1897341097	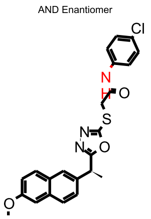 [*]N[*]	0.216
ECFP_6	99947387	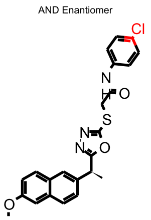 [*]:[c](:[*])Cl	0.181
**Top Features for negative contribution**			
**Fingerprint**	**Bit/Smiles**	**Feature Structure**	**Score**
ECFP_6	683445015	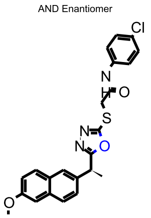 [*]:o:[*]	-0.266
ECFP_6	-817402818	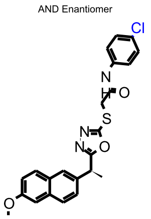 [*]Cl	-0.263

ECFP_6	-176455838	AND Enantiomer 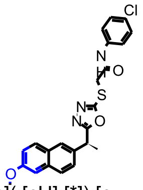 [*]O[c](:[cH]:[*]):[cH]:[*]	-0.257

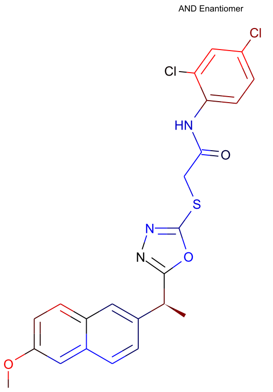

$C_{23}H_{19}Cl_2N_3O_3S$
Molecular Weight: 488.38626
ALogP: 5.567
Rotatable Bonds: 7
Acceptors: 5
Donors: 1

Model Prediction

Prediction: 0.473
Unit: g/kg_body_weight
Mahalanobis Distance: 24.281
Mahalanobis Distance p-value: 3.57e-027

Mahalanobis Distance: The Mahalanobis distance (MD) is a generalization of the Euclidean distance that accounts for correlations among the X properties. It is calculated as the distance to the center of the training data. The larger the MD, the less trustworthy the prediction.
Mahalanobis Distance p-value: The p-value gives the fraction of training data with an MD greater than or equal to the one for the given sample, assuming normally distributed data. The smaller the p-value, the less trustworthy the prediciton. For highly non-normal X properties (e.g., fingerprints), the MD p-value is wildly inaccurate.

Structural Similar Compounds

Name	ACEMETACIN	TALNIFLUMATE	bis-OXATIN ACETATE
Structure	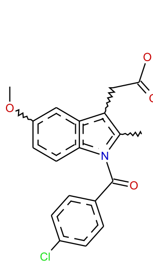	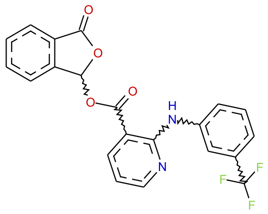	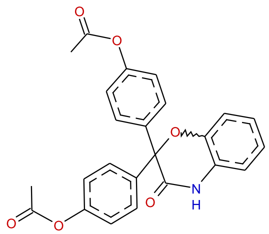
Actual Endpoint (-log C)	4.235	1.538	1.717
Predicted Endpoint (-log C)	3.39415	2.82541	2.40947
Distance	0.669	0.702	0.725
Reference	ARZNAD 30;1398;80	FRPSAX 36;372;81	NIIRDN 6;609;82

Model Applicability

Unknown features are fingerprint features in the query molecule, but not found in the training set.

1. All properties and OPS components are within expected ranges.
2. Unknown ECFP_2 feature: 1093109320: [*]S[c]1:o:[*]:[*]:n:1
3. Unknown ECFP_2 feature: 1092541557: [*]C([*])[c]1:o:[*]:[*]:n:1
4. Unknown FCFP_6 feature: 16: [*][c](:[*]):[*]
5. Unknown FCFP_6 feature: 1747237384: [*][c]1:[*]:[*]:[c]([*]):o:1
6. Unknown FCFP_6 feature: -1410079687: [*]S[c]1:o:[*]:[*]:n:1
7. Unknown FCFP_6 feature: 4427049: [*][c]1:[*]:[*]:n:n:1
8. Unknown FCFP_6 feature: 1618154665: [*][c](:[*]):[cH]:[cH]:[*]
9. Unknown FCFP_6 feature: -928857652: [*]:[c](:[*])C(C)[c](:[*]):[*]
10. Unknown FCFP_6 feature: -1539162406: [*]C([*])[c]1:o:[*]:[*]:n:1
11. Unknown FCFP_6 feature: 71476542: [*]:[c](:[*])Cl

Feature Contribution

Top features for positive contribution			
**Fingerprint**	**Bit/Smiles**	**Feature Structure**	**Score**

ECFP_6	642810091	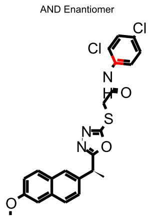 [*][c](:[*]):[*]	0.281
ECFP_6	-1897341097	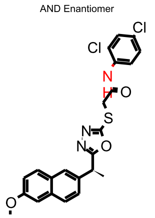 [*]N[*]	0.216
ECFP_6	577592657	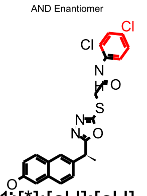 [*][c]1:[*]:[cH]:[cH]:[c](Cl):[cH]:1	0.194
**Top Features for negative contribution**			
**Fingerprint**	**Bit/Smiles**	**Feature Structure**	**Score**
ECFP_6	683445015	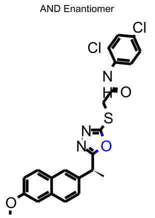 [*]:o:[*]	-0.266
ECFP_6	-817402818	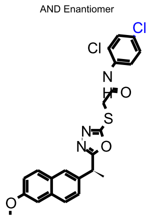 [*]Cl	-0.263

ECFP_6	-176455838	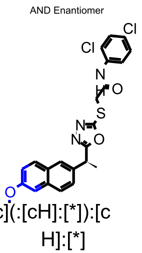<br>[*]O[c](:[cH]:[*]):[cH]:[*]	-0.257

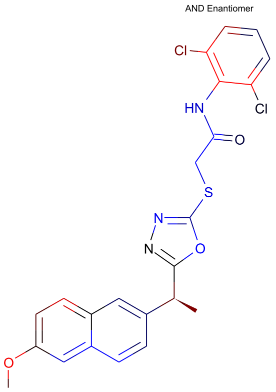

$C_{23}H_{19}Cl_2N_3O_3S$
Molecular Weight: 488.38626
ALogP: 5.567
Rotatable Bonds: 7
Acceptors: 5
Donors: 1

Structural Similar Compounds

Name	ACEMETACIN	TALNIFLUMATE	bis-OXATIN ACETATE
Structure	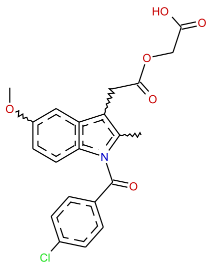	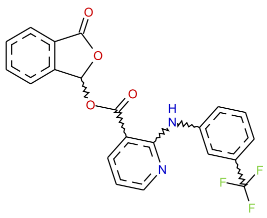	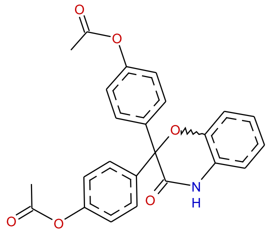
Actual Endpoint (-log C)	4.235	1.538	1.717
Predicted Endpoint (-log C)	3.39415	2.82541	2.40947
Distance	0.672	0.699	0.723
Reference	ARZNAD 30;1398;80	FRPSAX 36;372;81	NIIRDN 6;609;82

Model Prediction

Prediction: 0.660
Unit: g/kg_body_weight
Mahalanobis Distance: 24.281
Mahalanobis Distance p-value: 3.57e-027

Mahalanobis Distance: The Mahalanobis distance (MD) is a generalization of the Euclidean distance that accounts for correlations among the X properties. It is calculated as the distance to the center of the training data. The larger the MD, the less trustworthy the prediction.
Mahalanobis Distance p-value: The p-value gives the fraction of training data with an MD greater than or equal to the one for the given sample, assuming normally distributed data. The smaller the p-value, the less trustworthy the prediciton. For highly non-normal X properties (e.g., fingerprints), the MD p-value is wildly inaccurate.

Model Applicability

Unknown features are fingerprint features in the query molecule, but not found in the training set.

1. All properties and OPS components are within expected ranges.
2. Unknown ECFP_2 feature: 1093109320: [*]S[c]1:o:[*]:[*]:n:1
3. Unknown ECFP_2 feature: 1092541557: [*]C([*])[c]1:o:[*]:[*]:n:1
4. Unknown FCFP_6 feature: 16: [*][c](:[*]):[*]
5. Unknown FCFP_6 feature: 1747237384: [*][c]1:[*]:[*]:[c]([*]):o:1
6. Unknown FCFP_6 feature: -1410079687: [*]S[c]1:o:[*]:[*]:n:1
7. Unknown FCFP_6 feature: 4427049: [*][c]1:[*]:[*]:n:n:1
8. Unknown FCFP_6 feature: 1618154665: [*][c](:[*]):[cH]:[cH]:[*]
9. Unknown FCFP_6 feature: -928857652: [*]:[c](:[*])C(C)[c](:[*]):[*]
10. Unknown FCFP_6 feature: -1539162406: [*]C([*])[c]1:o:[*]:[*]:n:1
11. Unknown FCFP_6 feature: 71476542: [*]:[c](:[*])Cl

Feature Contribution

Top features for positive contribution

Fingerprint	Bit/Smiles	Feature Structure	Score

ECFP_6	642810091	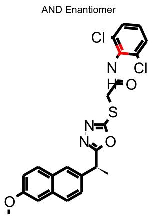 [*][c](:[*]):[*]	0.281
ECFP_6	-1897341097	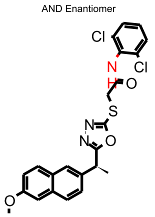 [*]N[*]	0.216
ECFP_6	99947387	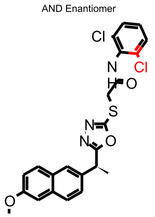 [*]:[c](:[*])Cl	0.181

Top Features for negative contribution

**Fingerprint**	**Bit/Smiles**	**Feature Structure**	**Score**
ECFP_6	683445015	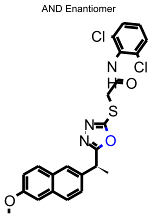 [*]:o:[*]	-0.266
ECFP_6	-817402818	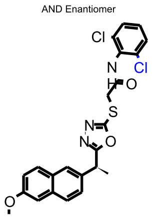 [*]Cl	-0.263

ECFP_6	-176455838	AND Enantiomer 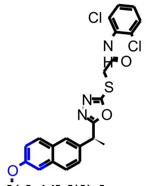 [*]O[c](:[cH]:[*]):[cH]:[*]	-0.257

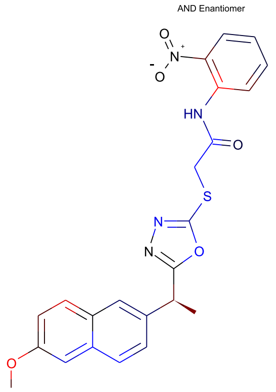

$C_{23}H_{20}N_4O_5S$
Molecular Weight: 464.4937
ALogP: 4.132
Rotatable Bonds: 8
Acceptors: 7
Donors: 1

Structural Similar Compounds

Name	BENZENESULFONIC ACID; 2;2'-(4;4'-BIPHENYLYLENEDIVINYLENE)DI-; DISODIUM SALT (Na STRIPPED)	ACEMETACIN	BENZOTHIAZOLE; 6-NITRO-2-(p-NITROBENZOYLAMINO)-
Structure	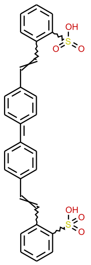	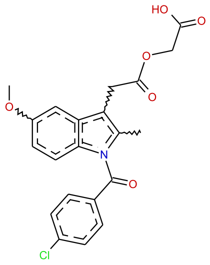	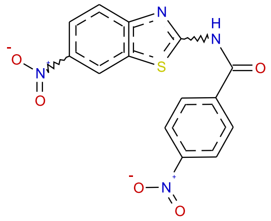
Actual Endpoint (-log C)	1.968	4.235	2.361
Predicted Endpoint (-log C)	1.72109	3.39415	2.96257
Distance	0.746	0.771	0.795
Reference	MVCRB3 2;193;73	ARZNAD 30;1398;80	JPETAB 90;260;47

Model Prediction

Prediction: 2.753
Unit: g/kg_body_weight
Mahalanobis Distance: 24.744
Mahalanobis Distance p-value: 1.11e-029

Mahalanobis Distance: The Mahalanobis distance (MD) is a generalization of the Euclidean distance that accounts for correlations among the X properties. It is calculated as the distance to the center of the training data. The larger the MD, the less trustworthy the prediction.
Mahalanobis Distance p-value: The p-value gives the fraction of training data with an MD greater than or equal to the one for the given sample, assuming normally distributed data. The smaller the p-value, the less trustworthy the prediciton. For highly non-normal X properties (e.g., fingerprints), the MD p-value is wildly inaccurate.

Model Applicability

Unknown features are fingerprint features in the query molecule, but not found in the training set.

1. All properties and OPS components are within expected ranges.
2. Unknown ECFP_2 feature: 1043790491: [*][N+](=[*])[*]
3. Unknown ECFP_2 feature: 781519895: [*][O-]
4. Unknown ECFP_2 feature: 1093109320: [*]S[c]1:o:[*]:[*]:n:1
5. Unknown ECFP_2 feature: 1092541557: [*]C([*])[c]1:o:[*]:[*]:n:1
6. Unknown ECFP_2 feature: -1956535100: [*][c](:[*]):[c]([N+](=[*])[*]):c:[*]
7. Unknown ECFP_2 feature: -215026467: [*]:[c](:[*])[N+](=O)[O-]
8. Unknown ECFP_2 feature: 2104376220: [*][N+](=O)[*]
9. Unknown ECFP_2 feature: -659271057: [*][N+](=[*])[O-]
10. Unknown FCFP_6 feature: 16: [*][c](:[*]):[*]
11. Unknown FCFP_6 feature: 8: [*][N+](=[*])[*]
12. Unknown FCFP_6 feature: 5: [*][O-]
13. Unknown FCFP_6 feature: 1747237384: [*][c]1:[*]:[*]:[c]([*]):o:1
14. Unknown FCFP_6 feature: -1410079687: [*]S[c]1:o:[*]:[*]:n:1
15. Unknown FCFP_6 feature: 4427049: [*][c]1:[*]:[*]:n:n:1
16. Unknown FCFP_6 feature: 1618154665: [*][c](:[*]):[cH]:[cH]:[*]
17. Unknown FCFP_6 feature: -928857652: [*]:[c](:[*])C(C)[c](:[*]):[*]
18. Unknown FCFP_6 feature: -1539162406: [*]C([*])[c]1:o:[*]:[*]:n:1

19. Unknown FCFP_6 feature: -828984032: [*][c](:[*]):[c](:[cH]:[*])[N+](=[*])[*]
20. Unknown FCFP_6 feature: -1338588315: [*]:[c](:[*])[N+](=O)[O-]
21. Unknown FCFP_6 feature: 1872392852: [*][N+](=O)[*]
22. Unknown FCFP_6 feature: 260476081: [*][N+](=[*])[O-]

Feature Contribution

Top features for positive contribution

Fingerprint	Bit/Smiles	Feature Structure	Score
ECFP_6	642810091	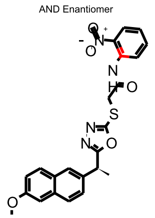 [*][c](:[*]):[*]	0.281
ECFP_6	-1897341097	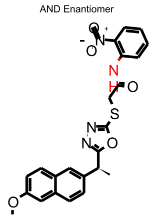 [*]N[*]	0.216
FCFP_6	136627117	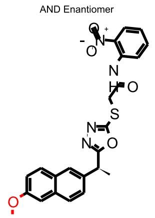 [*]OC	0.170

Top Features for negative contribution

Fingerprint	Bit/Smiles	Feature Structure	Score

ECFP_6	683445015	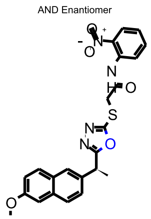 [*]:o:[*]	-0.266
ECFP_6	-176455838	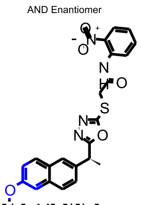 [*]O[c](:[cH]:[*]):[cH]:[*]	-0.257
ECFP_6	655739385	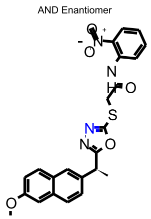 [*]:n:[*]	-0.239

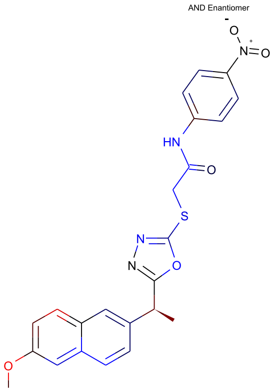

$C_{23}H_{20}N_4O_5S$

Molecular Weight: 464.4937

ALogP: 4.132

Rotatable Bonds: 8

Acceptors: 7

Donors: 1

Structural Similar Compounds

Name	BENZENESULFONIC ACID; 2;2'-(4;4'-BIPHENYLYLENEDIVINYLENE)DI-; DISODIUM SALT (Na STRIPPED)	ACEMETACIN	BENZOTHIAZOLE; 6-NITRO-2-(p-NITROBENZOYLAMINO)-
Structure	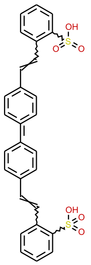	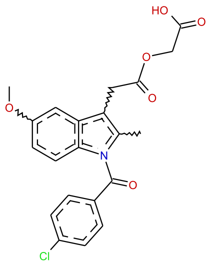	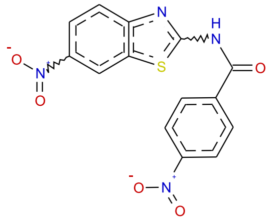
Actual Endpoint (-log C)	1.968	4.235	2.361
Predicted Endpoint (-log C)	1.72109	3.39415	2.96257
Distance	0.753	0.771	0.792
Reference	MVCRB3 2;193;73	ARZNAD 30;1398;80	JPETAB 90;260;47

Model Prediction

Prediction: 1.832

Unit: g/kg_body_weight

Mahalanobis Distance: 24.861

Mahalanobis Distance p-value: 2.47e-030

Mahalanobis Distance: The Mahalanobis distance (MD) is a generalization of the Euclidean distance that accounts for correlations among the X properties. It is calculated as the distance to the center of the training data. The larger the MD, the less trustworthy the prediction.
Mahalanobis Distance p-value: The p-value gives the fraction of training data with an MD greater than or equal to the one for the given sample, assuming normally distributed data. The smaller the p-value, the less trustworthy the prediciton. For highly non-normal X properties (e.g., fingerprints), the MD p-value is wildly inaccurate.

Model Applicability

Unknown features are fingerprint features in the query molecule, but not found in the training set.

1. All properties and OPS components are within expected ranges.
2. Unknown ECFP_2 feature: 1043790491: [*][N+](=[*])[*]
3. Unknown ECFP_2 feature: 781519895: [*][O-]
4. Unknown ECFP_2 feature: 1093109320: [*]S[c]1:o:[*]:[*]:n:1
5. Unknown ECFP_2 feature: 1092541557: [*]C([*])[c]1:o:[*]:[*]:n:1
6. Unknown ECFP_2 feature: -179073144: [*][N+](=[*])[c](:c:[*]):c:[*]
7. Unknown ECFP_2 feature: -215026467: [*]:[c](:[*])[N+](=O)[O-]
8. Unknown ECFP_2 feature: 2104376220: [*][N+](=O)[*]
9. Unknown ECFP_2 feature: -659271057: [*][N+](=[*])[O-]
10. Unknown FCFP_6 feature: 16: [*][c](:[*]):[*]
11. Unknown FCFP_6 feature: 8: [*][N+](=[*])[*]
12. Unknown FCFP_6 feature: 5: [*][O-]
13. Unknown FCFP_6 feature: 1747237384: [*][c]1:[*]:[*]:[c]([*]):o:1
14. Unknown FCFP_6 feature: -1410079687: [*]S[c]1:o:[*]:[*]:n:1
15. Unknown FCFP_6 feature: 4427049: [*][c]1:[*]:[*]:n:n:1
16. Unknown FCFP_6 feature: 1618154665: [*][c](:[*]):[cH]:[cH]:[*]
17. Unknown FCFP_6 feature: -928857652: [*]:[c](:[*])C(C)[c](:[*]):[*]
18. Unknown FCFP_6 feature: -1539162406: [*]C([*])[c]1:o:[*]:[*]:n:1

19. Unknown FCFP_6 feature: -828984032: [*][c](:[*]):[c](:[cH]:[*])[N+](=[*])[*]
20. Unknown FCFP_6 feature: -1338588315: [*]:[c](:[*])[N+](=O)[O-]
21. Unknown FCFP_6 feature: 1872392852: [*][N+](=O)[*]
22. Unknown FCFP_6 feature: 260476081: [*][N+](=[*])[O-]

Feature Contribution

Top features for positive contribution

Fingerprint	Bit/Smiles	Feature Structure	Score
ECFP_6	642810091	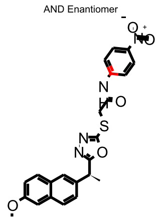 [*][c](:[*]):[*]	0.281
ECFP_6	-1897341097	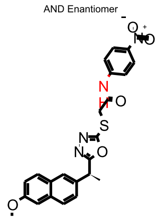 [*]N[*]	0.216
FCFP_6	136627117	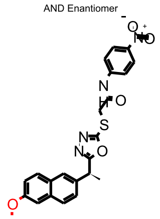 [*]OC	0.170

Top Features for negative contribution

Fingerprint	Bit/Smiles	Feature Structure	Score

ECFP_6	683445015	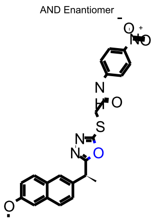 [*]:o:[*]	-0.266
ECFP_6	-176455838	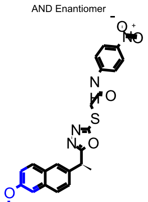 [*]O[c](:[cH]:[*]):[cH]:[*]	-0.257
ECFP_6	655739385	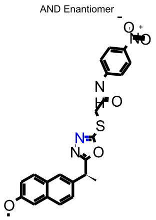 [*]:n:[*]	-0.239

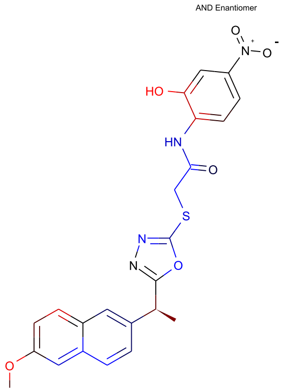

$C_{23}H_{20}N_4O_6S$

Molecular Weight: 480.4931

ALogP: 3.89

Rotatable Bonds: 8

Acceptors: 8

Donors: 2

Structural Similar Compounds

Name	BENZENESULFONIC ACID; 2;2'-(4;4'-BIPHENYLYLENEDIVINYLENE)DI-; DISODIUM SALT (Na STRIPPED)	BENZOTHIAZOLE; 6-NITRO-2-(p-NITROBENZOYLAMINO)-	FEBANTEL
Structure	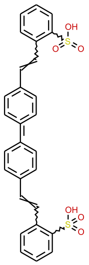	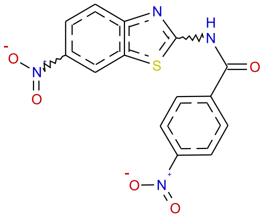	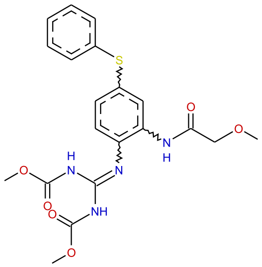
Actual Endpoint (-log C)	1.968	2.361	1.624
Predicted Endpoint (-log C)	1.72109	2.96257	2.37098
Distance	0.797	0.840	0.888
Reference	MVCRB3 2;193;73	JPETAB 90;260;47	ARZNAD 28;2193;78

Model Prediction

Prediction: 1.669

Unit: g/kg_body_weight

Mahalanobis Distance: 28.036

Mahalanobis Distance p-value: 4.29e-050

Mahalanobis Distance: The Mahalanobis distance (MD) is a generalization of the Euclidean distance that accounts for correlations among the X properties. It is calculated as the distance to the center of the training data. The larger the MD, the less trustworthy the prediction.
Mahalanobis Distance p-value: The p-value gives the fraction of training data with an MD greater than or equal to the one for the given sample, assuming normally distributed data. The smaller the p-value, the less trustworthy the prediciton. For highly non-normal X properties (e.g., fingerprints), the MD p-value is wildly inaccurate.

Model Applicability

Unknown features are fingerprint features in the query molecule, but not found in the training set.

1. All properties and OPS components are within expected ranges.
2. Unknown ECFP_2 feature: 1043790491: [*][N+](=[*])[*]
3. Unknown ECFP_2 feature: 781519895: [*][O-]
4. Unknown ECFP_2 feature: 1093109320: [*]S[c]1:o:[*]:[*]:n:1
5. Unknown ECFP_2 feature: 1092541557: [*]C([*])[c]1:o:[*]:[*]:n:1
6. Unknown ECFP_2 feature: -179073144: [*][N+](=[*])[c](:c:[*]):c:[*]
7. Unknown ECFP_2 feature: -215026467: [*]:[c](:[*])[N+](=O)[O-]
8. Unknown ECFP_2 feature: 2104376220: [*][N+](=O)[*]
9. Unknown ECFP_2 feature: -659271057: [*][N+](=[*])[O-]
10. Unknown FCFP_6 feature: 16: [*][c](:[*]):[*]
11. Unknown FCFP_6 feature: 8: [*][N+](=[*])[*]
12. Unknown FCFP_6 feature: 5: [*][O-]
13. Unknown FCFP_6 feature: 1747237384: [*][c]1:[*]:[*]:[c]([*]):o:1
14. Unknown FCFP_6 feature: -1410079687: [*]S[c]1:o:[*]:[*]:n:1
15. Unknown FCFP_6 feature: 4427049: [*][c]1:[*]:[*]:n:n:1
16. Unknown FCFP_6 feature: 1618154665: [*][c](:[*]):[cH]:[cH]:[*]
17. Unknown FCFP_6 feature: -928857652: [*]:[c](:[*])C(C)[c](:[*]):[*]
18. Unknown FCFP_6 feature: -1539162406: [*]C([*])[c]1:o:[*]:[*]:n:1

19. Unknown FCFP_6 feature: -828984032: [*][c](:[*]):[c](:[cH]:[*])[N+](=[*])[*]
20. Unknown FCFP_6 feature: 74595001: [*][c](:[*]):[c](O):[cH]:[*]
21. Unknown FCFP_6 feature: -549108873: [*]:[c](:[*])O
22. Unknown FCFP_6 feature: -1338588315: [*]:[c](:[*])[N+](=O)[O-]
23. Unknown FCFP_6 feature: 1872392852: [*][N+](=O)[*]
24. Unknown FCFP_6 feature: 260476081: [*][N+](=[*])[O-]

Feature Contribution

Top features for positive contribution

Fingerprint	Bit/Smiles	Feature Structure	Score
ECFP_6	642810091	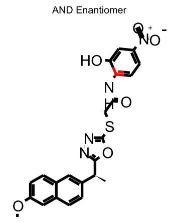 [*][c](:[*]):[*]	0.281
ECFP_6	-1897341097	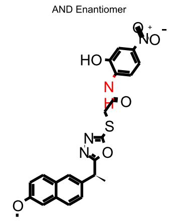 [*]N[*]	0.216
FCFP_6	136627117	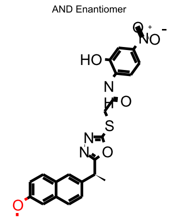 [*]OC	0.170

Top Features for negative contribution

Fingerprint	Bit/Smiles	Feature Structure	Score

ECFP_6	683445015	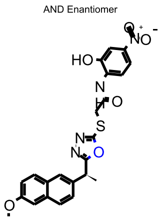 [*]:o:[*]	-0.266
ECFP_6	-176455838	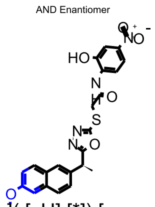 [*]O[c](:[cH]:[*]):[cH]:[*]	-0.257
ECFP_6	655739385	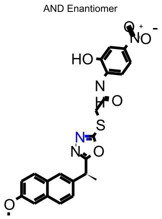 [*]:n:[*]	-0.239

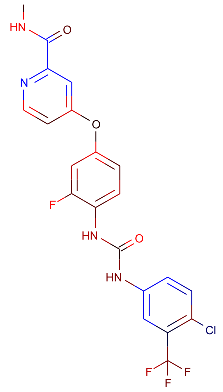

$C_{21}H_{15}ClF_4N_4O_3$
Molecular Weight: 482.81541
ALogP: 4.381
Rotatable Bonds: 6
Acceptors: 4
Donors: 3

Structural Similar Compounds

Name	PHOSPHORAMIDOTHIOIC ACID; ACETIMIDOYL-; O;O-bis-(p-CHLOROPHENYL)ESTER	FLUBENDAZOLE	OXYCLOZANIDE
Structure	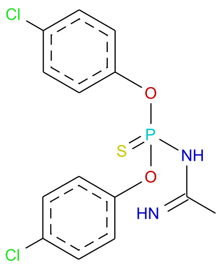	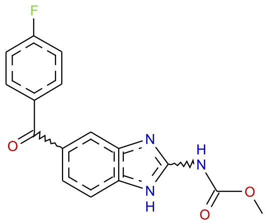	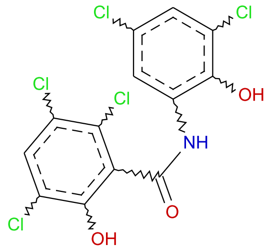
Actual Endpoint (-log C)	5.006	2.088	2.604
Predicted Endpoint (-log C)	3.23989	2.69288	2.94104
Distance	0.720	0.728	0.732
Reference	FMCHA2 -;C149;89	YRTMA6 9;11;78	NATUAS 210;744;66

Model Prediction

Prediction: 0.890
Unit: g/kg_body_weight
Mahalanobis Distance: 21.059
Mahalanobis Distance p-value: 1.49e-012

Mahalanobis Distance: The Mahalanobis distance (MD) is a generalization of the Euclidean distance that accounts for correlations among the X properties. It is calculated as the distance to the center of the training data. The larger the MD, the less trustworthy the prediction.
Mahalanobis Distance p-value: The p-value gives the fraction of training data with an MD greater than or equal to the one for the given sample, assuming normally distributed data. The smaller the p-value, the less trustworthy the prediciton. For highly non-normal X properties (e.g., fingerprints), the MD p-value is wildly inaccurate.

Model Applicability

Unknown features are fingerprint features in the query molecule, but not found in the training set.

1. All properties and OPS components are within expected ranges.
2. Unknown FCFP_6 feature: 16: [*][c](:[*]):[*]
3. Unknown FCFP_6 feature: 1618154665: [*][c](:[*]):[cH]:[cH]:[*]
4. Unknown FCFP_6 feature: 1747237384: [*][c]1:[*]:[*]:[c]([*]):o:1
5. Unknown FCFP_6 feature: 136686699: [*]NC
6. Unknown FCFP_6 feature: 71476542: [*]:[c](:[*])Cl

Feature Contribution

Top features for positive contribution			
**Fingerprint**	**Bit/Smiles**	**Feature Structure**	**Score**

FCFP_6	71953198	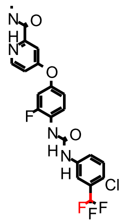 [*]C([*])([*])F	0.392
ECFP_6	-1046436026	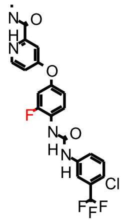 [*]F	0.349
ECFP_6	642810091	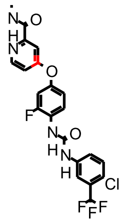 [*][c](:[*]):[*]	0.281
**Top Features for negative contribution**			
**Fingerprint**	**Bit/Smiles**	**Feature Structure**	**Score**
ECFP_6	226796801	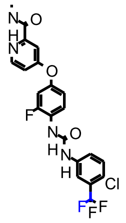 [*]C([*])([*])F	-0.320
ECFP_6	-817402818	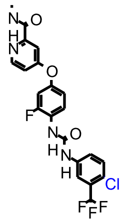 [*]Cl	-0.263

ECFP_6	-176455838	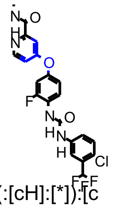 [*]O[c](:[cH]:[*]):[cH]:[*]	-0.257
